# Human parainfluenza virus 3 field strains undergo extracellular fusion protein cleavage to activate entry

**DOI:** 10.1128/mbio.02327-24

**Published:** 2024-10-09

**Authors:** Kyle Stearns, George Lampe, Rachel Hanan, Tara Marcink, Stefan Niewiesk, Samuel H. Sternberg, Alexander L. Greninger, Matteo Porotto, Anne Moscona

**Affiliations:** 1 Department of Pediatrics, Columbia University Vagelos College of Physicians and Surgeons, New York, New York, USA; 2 Center for Host–Pathogen Interaction, Columbia University Vagelos College of Physicians and Surgeons, New York, New York, USA; 3 Department of Physiology & Cellular Biophysics, Columbia University Vagelos College of Physicians and Surgeons, New York, New York, USA; 4 Department of Biochemistry and Molecular Biophysics, Columbia University Vagelos College of Physicians and Surgeons, New York, New York, USA; 5 Department of Veterinary Biosciences, College of Veterinary Medicine, The Ohio State University, Columbus, Ohio, USA; 6 Department of Laboratory Medicine and Pathology, University of Washington, Seattle, Washington, USA; 7 Vaccine and Infectious Disease Division, Fred Hutchinson Cancer Research Center, Seattle, Washington, USA; 8 Department of Experimental Medicine, University of Campania “Luigi Vanvitelli”, Caserta, Italy; 9 Department of Microbiology & Immunology, Columbia University Vagelos College of Physicians and Surgeons, New York, New York, USA; St. Jude Children's Research Hospital, Memphis, Tennessee, USA

**Keywords:** viral entry, membrane fusion, fusion protein, paramyxovirus, parainfluenza virus, proteases

## Abstract

Human parainfluenza virus 3 (HPIV3) infection is driven by the coordinated action of viral surface glycoproteins hemagglutinin-neuraminidase (HN) and fusion protein (F). Receptor-engaged HN activates F to insert into the target cell membrane and drive virion-cell membrane fusion. For F to mediate entry, its precursor (F0) must first be cleaved by host proteases. F0 cleavage has been thought to be executed during viral glycoprotein transit through the trans-Golgi network by the ubiquitously expressed furin because F0 proteins of laboratory-adapted viruses contain a furin recognition dibasic cleavage motif RXKR around residue 108. Here, we show that the F proteins of field strains have a different cleavage motif from laboratory-adapted strains and are cleaved by unidentified proteases expressed in only a narrow subset of cell types. We demonstrate that extracellular serine protease inhibitors block HPIV3 F0 cleavage for field strains, suggesting F0 cleavage occurs at the cell surface facilitated by transmembrane proteases. Candidate proteases that may process HPIV3 F *in vivo* were identified by a genome-wide CRISPRa screen in HEK293/dCas9-VP64 + MPH cells. The lung-expressed extracellular serine proteases TMPRSS2 and TMPRSS13 are both sufficient to cleave HPIV3 F and enable infectious virus release by otherwise non-permissive cells. Our findings support an alternative mechanism of F activation *in vivo*, reliant on extracellular membrane-bound serine proteases expressed in a narrow subset of cells. The proportion of HPIV3 F proteins cleaved and infectious virus release is determined by host cell expression of requisite proteases, allowing just-in-time activation of F and positioning F cleavage as another key regulator of HPIV3 spread.

**IMPORTANCE:**

Enveloped viruses cause a wide range of diseases in humans. At the first step of infection, these viruses must fuse their envelope with a cell membrane to initiate infection. This fusion is mediated by viral proteins that require a critical activating cleavage event. It was previously thought that for parainfluenza virus 3, an important cause of respiratory disease and a representative of a group of important pathogens, this cleavage event was mediated by furin in the cell secretory pathways prior to formation of the virions. We show that this is only true for laboratory strain viruses, and that clinical viruses that infect humans utilize extracellular proteases that are only made by a small subset of cells. These results highlight the importance of studying authentic clinical viruses that infect human tissues for understanding natural infection.

## INTRODUCTION

Acute respiratory infection is the leading cause of mortality in children under 5  years of age. Human parainfluenza viruses (HPIV) types 1, 2, 3, 4a, and 4b, human metapneumovirus, and respiratory syncytial virus are enveloped negative-sense RNA viruses (paramyxoviruses and pneumoviruses) that cause the majority of childhood croup, bronchiolitis, and pneumonia (1–4). No effective drugs or vaccines are available for the parainfluenza viruses. Like other paramyxoviruses, HPIV3 enters cells by fusing directly with the cell membrane (5–7). Viral entry into target cells is mediated by two viral surface glycoproteins, the dimeric (8, 9) multi-functional receptor binding protein (hemagglutinin-neuraminidase; HN) and the trimeric fusion protein (F) (10–15), that function in a complex on the surface of infectious virions (8, 9).

To initiate infection, the HPIV3 receptor binding protein HN binds to sialic acid bearing receptors on a target cell surface and triggers F to undergo a series of conformational changes, proceeding through a transient extended intermediate (10, 16, 17). F is synthesized as an inactive F0 precursor, which is cleaved by host cell proteases into its active form composed of two subunits, F1 and F2, linked by a disulfide bridge (18). Cleavage of F is essential for infectivity for HPIV3 as well as the other paramyxoviruses and pneumoviruses (11, 19–21). This cleavage exposes the hydrophobic fusion peptide at the newly exposed N-terminus of F1 that will insert into the target membrane. Refolding of the fusion protein then drives virion-cell membrane fusion resulting in the viral genome’s release into the target cell (5, 22–29). Cleavage of F0 has been thought to occur intracellularly during processing and transit to the cell surface and to depend on the intracellular protease furin, so that budded virions bear cleaved F1/F2 on their surfaces (30–33).

The HPIV HN-F fusion complexes have been honed by evolution to support viral spread within and between human hosts. These fusion complexes can adapt through a variety of mechanisms to mediate viral entry in artificial environments, such as monolayer cell cultures that lack the requisite host factors for infection with field strains of virus (34, 35). The study of parainfluenza viruses until recently was limited to culture-adapted strains. However, infectious viruses precisely fit their hosts, and the study of natural viral infection depends on host-specific mechanisms (12, 34–39). The HN-F fusion pairs of circulating HPIV3 viruses possess a balance of properties that enable circulating viruses to propagate more efficiently *in vivo* than in cultured cells (12, 34–38). The HN-F fusion complex as a unit adapts to permit viability in the different environments of cell cultures and human beings.

We have shown that circulating HPIV3 strains are genetically different from viruses grown in standard laboratory conditions and specifically differ at the F protease cleavage site (34). Here we show by sequence comparison of HPIV3 fusion proteins that current and historic laboratory-adapted strains bear the same dibasic cleavage site (R-X-K-R) in F (K108), consistent with furin cleavage, while field strains differ by a single residue (E108) and possess a highly conserved monobasic cleavage site (R-X-E-R). The furin-dependent strains bearing F with a dibasic cleavage site (R-X-K-R) are artifacts of rapid viral adaptation to cell culture (30, 34, 35, 40). Identifying which proteases are sufficient for HPIV3 F cleavage in the human lungs and the cellular location of cleavage is critical for understanding where and when the fusion protein becomes functional. We identify extracellular serine proteases sufficient for HPIV3 field strain cleavage and highlight HPIV3 F protease specificity as a key regulator of infectious HPIV3 release.

## RESULTS

### Human parainfluenza viruses in circulation have a different fusion protein cleavage motif than lab-adapted strains

**Fig 1:**
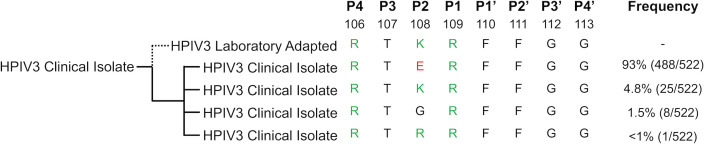
Human parainfluenza viruses in circulation have different fusion protein cleavage motifs than lab-adapted strains. HPIV3 fusion protein sequences were extracted with NCBI Protein Blast [NC_001796 fusion protein (NP_067151.1, accessed 5/18/22)]. Listed are cleavage motif sequence alignments of laboratory-adapted and patient-derived HPIV3. Reported are the observed sequence frequencies and the amino acid sequences of four amino acids upstream and downstream of the HPIV3 F1/F2 cleavage site. Green, positively charged amino acids; red, negatively charged amino acids.

We made the striking finding that the F proteins of a set of unpassaged clinical strains of HPIV3 were all different from laboratory-passaged strains at position 108 (34), the critical cleavage activation site residue of F. To expand the global sequence context at the HPIV3 F1/F2 junction, we first examined 522 HPIV3 F sequences in NCBI Protein. The vast majority of sequences (93.4%) had a glutamic acid at residue 108, in the P2 position of the cleavage site (Fig. 1). Alternative amino acids at residue 108 included lysine (4.8%) already known to be present in laboratory-adapted strains, as well as glycine (1.5%) and arginine (0.2%). Review of the manuscripts and history of isolates bearing the alternative amino acids at residue 108 indicated that indeed these generally could be documented to have a history of adaptation in culture and were not sequenced directly from clinical specimens (41–47), in contrast to the isolates bearing glutamic acid at residue 108 (34). Specifically, out of a total of 522 isolates sequenced (Fig. 1), 34 have alternate amino acids at residue 108 and 24 of those have published documentation of cell culture passage. Of the remaining 10, 6 had no information and only 4 were sequenced directly from patients. Taken together, these data confirmed that lysine at residue 108 is a hallmark of laboratory adaptation in HPIV3.

### Field strain HPIV3 infectivity is determined by *ex vivo* tissues and cell cultures abilities to cleave HPIV3 F

**Fig 2:**
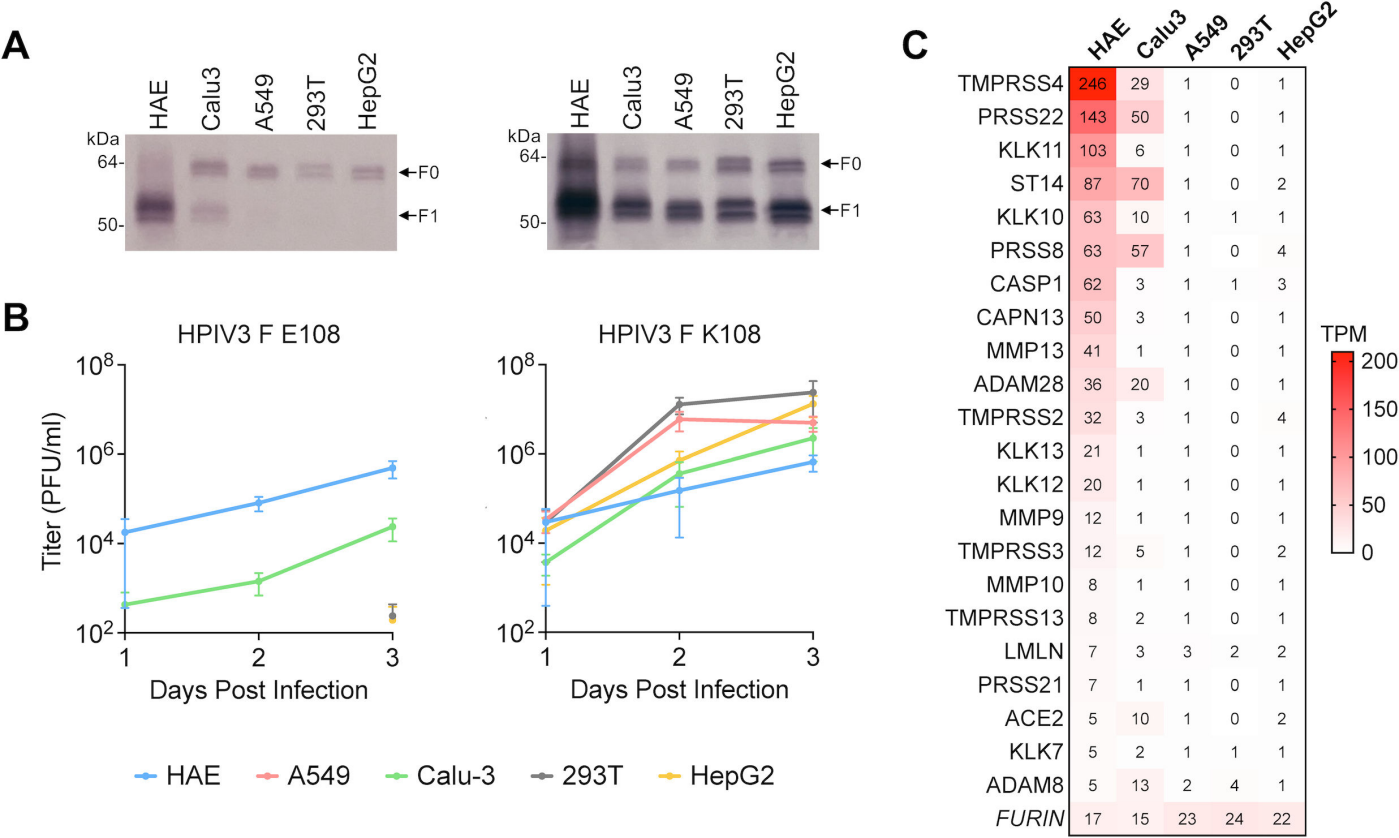
HPIV3 fusion protein cleavage and infectious virion production by *ex vivo* tissues and immortalized cell cultures. The indicated tissue (HAE) and immortalized cells were inoculated with HPIV3 F E108 (left) or HPIV3 F K108 (right) with the JS strain background. HPIV3 in cell culture media and HAE apical washes were collected 1–3 days after infection. (**A**) HPIV3-infected cell culture media and HAE apical wash resolved by reducing SDS-PAGE and immunoblotting with anti-HPIV3 F antibody. (**B**) HPIV3 titer in cell culture media and HAE apical washes 1–3 days after infection. (**C**) Proteases with transcripts per million (TPM) expression level >5 in HAE or Calu-3, and <5 in A549, HEK293T, and HepG2 cells determined by RNA-seq. Values are means and standard error of the means from three biological replicates.

To identify cells expressing proteases sufficient for infectious HPIV3 release, human airway epithelium (HAE) cultures as well as Calu-3, A549, HEK293T, and HepG2 cells were infected with either recombinant field strain (HPIV3 F E108) or laboratory-adapted (HPIV3 F K108) viruses with otherwise identical genetic backgrounds, and HPIV3 F cleavage and infectious virus release were compared. Protease expression in permissive and non-permissive cells was then compared to identify proteases that may be sufficient for F E108 cleavage. We first identified which of the immortalized cell culture models could release infectious HPIV3 F E108 and express a protease sufficient to cleave F E108. We tested Calu-3 and A549 cells because they are lung adenocarcinoma cells grown in monolayer cell culture and are frequently used to characterize respiratory viruses (48–51). We also infected HEK293T cells (derived from human embryonic kidney) and HepG2 cells (derived from liver hepatocellular carcinoma) to broaden the protease profiles included in the comparison (Fig. 2). HAE (MatTek) was used because we have shown that clinical strains of HPIV3 maintain their genomic sequence during growth in HAE, indicating that the HAE tissue environment does not exert selective pressure on the fusion mechanism—HPIV3 does not need to adapt to allow the HAE to support growth (34, 36, 37, 52)—and HAE expresses the proteases required for HPIV3 fitness in the human lung (34, 35). The proteases expressed in the HAE and permissive cells, but not in the non-permissive cells, were considered as candidates.

HAE, Calu-3, A549, HEK293T, and HepG2 cells were infected with HPIV3 bearing F proteins with either the E108 or K108 cleavage motif. The residue at 108 was the only difference between the viruses. All cultures were susceptible to primary infection and cleaved the F K108 due to their ubiquitous expression of furin (Fig. 2C). Only HAE and Calu-3 cells cleaved F E108 and released infectious virions (Fig. 2A). We compared the proteases expressed in the permissive HAE and Calu-3 cells to the proteases expressed in the non-permissive cells using RNA-seq data. This comparison revealed that the permissive cells were enriched in extracellular serine proteases. The restriction of HPIV3 F E108 cleavage and spread to HAE and Calu-3 cultures indicated that ubiquitously expressed furin is not sufficient for cleaving F E108 or for production of infectious field strain HPIV3, and that other proteases expressed in a narrower subset of cells are necessary for release of infectious virions.

### HPIV3 fusion proteins are cleaved by extracellular serine proteases

**Fig 3:**
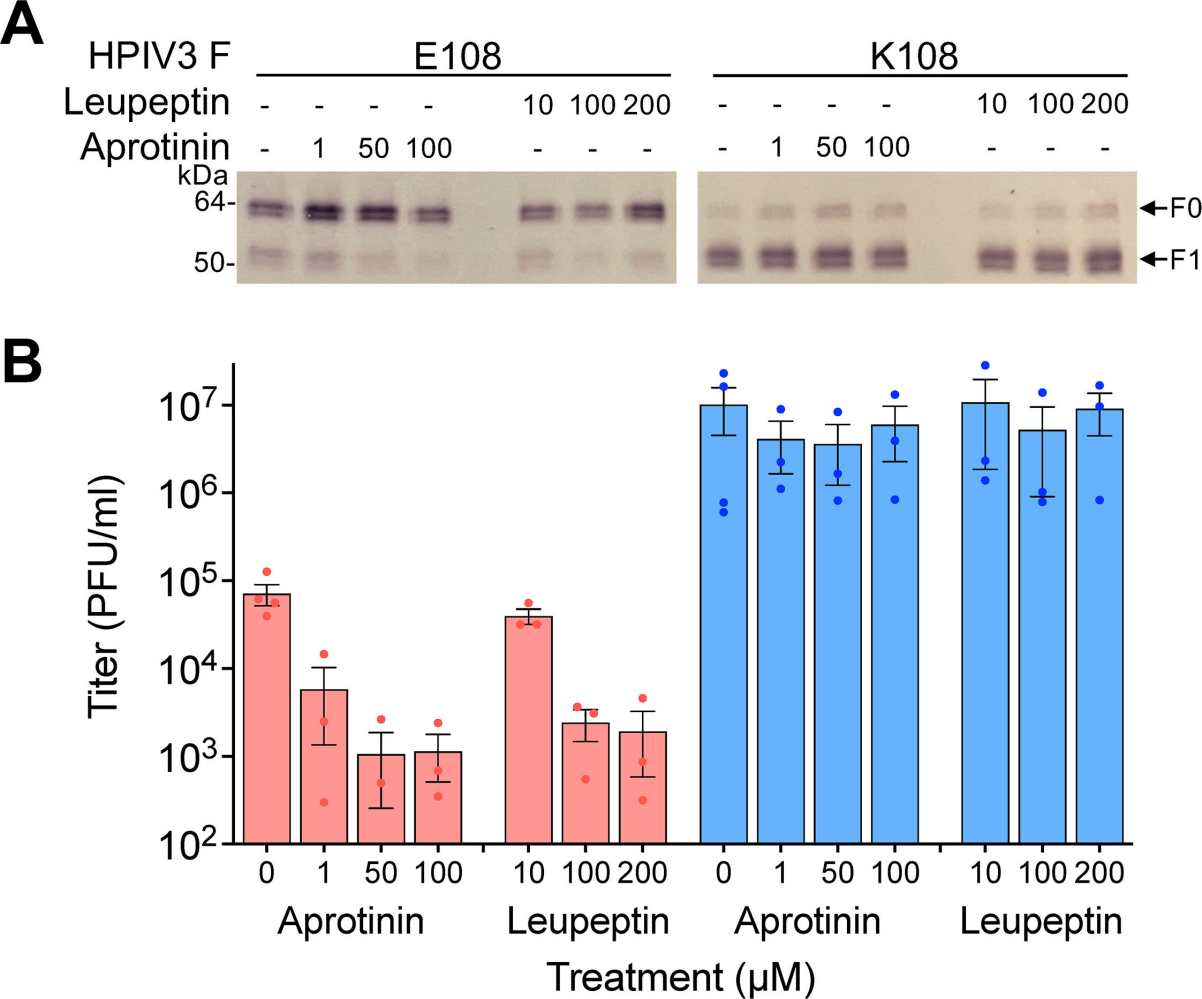
Inhibition of extracellular serine proteases with aprotinin or leupeptin blocks HPIV3 F (E108) cleavage and reduces the production of infectious HPIV3. Calu-3 cells were inoculated with HPIV3 F E108 (red) or HPIV3 F K108 (blue) with JS strain background. After inoculation, cells were treated with the indicated concentrations of aprotinin or leupeptin. (**A**) HPIV3-infected Calu-3 cell culture media were resolved by reducing SDS-PAGE and immunoblotted with anti-HPIV3 F antibody. (**B**) HPIV3 titer in cell culture media was assessed 3 days after infection. Values are means and SEM from three biological replicates.

Because many of the proteases uniquely expressed in permissive cells are extracellular serine proteases (Fig. 2C) and proteases from that family are required for cleavage of the fusion proteins of several respiratory viruses (53–55), we explored their sufficiency for HPIV3 F cleavage. Calu-3 cells were infected with HPIV3 (bearing field strain F, F E108, or laboratory strain F, F K108) and we tested if the cell-impermeable serine protease inhibitors aprotinin or leupeptin affect HPIV3 F cleavage and infectious virion release (Fig. 3). Aprotinin and leupeptin caused a dose-dependent reduction in the proportion of HPIV3 F E108 cleaved and the number of infectious viral particles released but had no effect on HPIV3 F K108 (Fig. 3). These results suggest that the HPIV3 F cleavage required for infectious virion production by field strains may occur at the cell surface, mediated by extracellular serine proteases.

**Fig 4:**
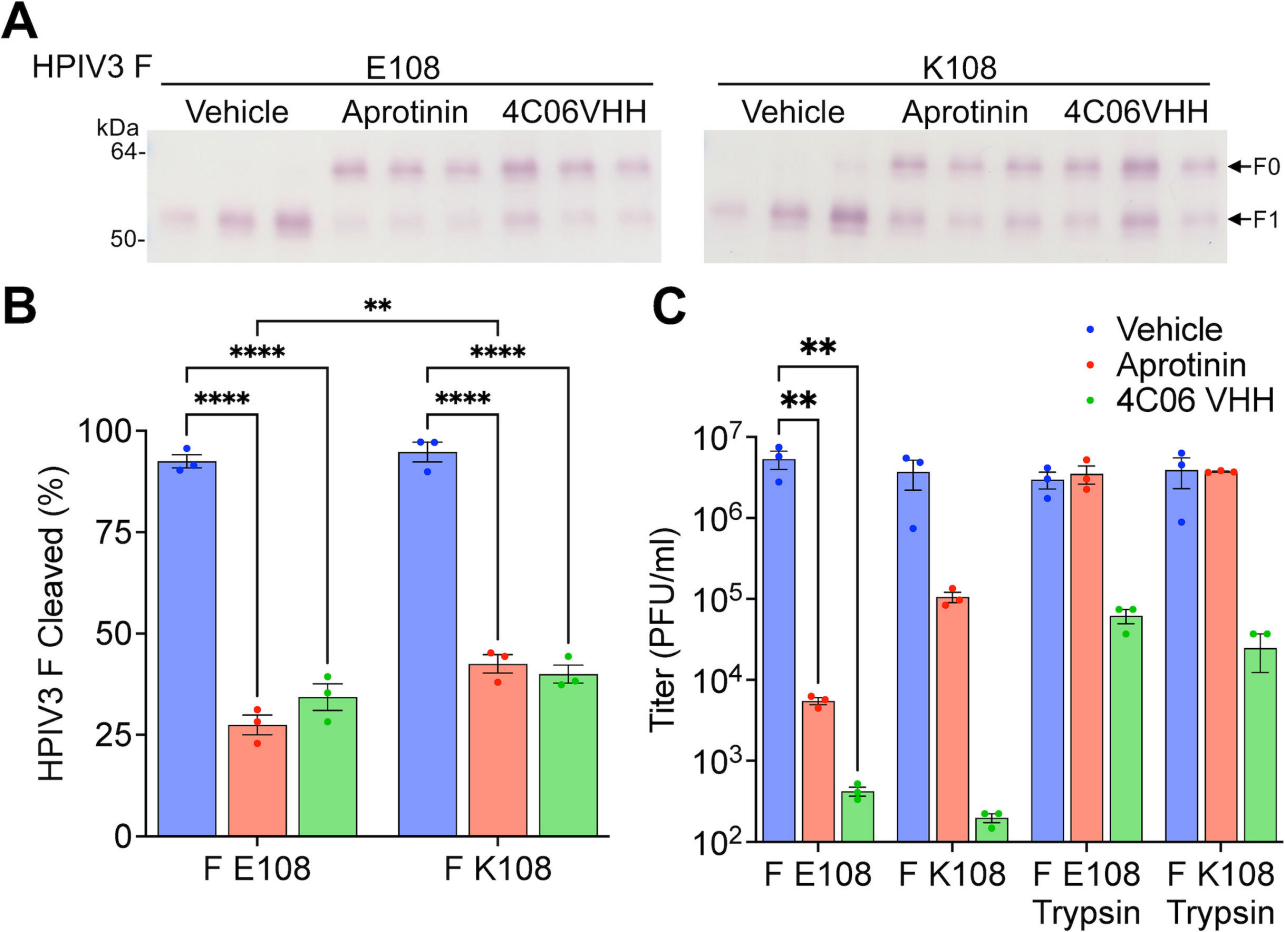
Inhibition of extracellular HPIV3 F (E108) cleavage by aprotinin or steric hindrance of the F cleavage site on HAE. HAE cells were inoculated with HPIV3 F E108 or HPIV3 F K108 with CI-1 background. After inoculation, cells were apically treated with vehicle, 1 mM aprotinin, or 2.6 µM 4C06 VHH-Fc. (**A**) HPIV3-infected HAE supernatants were resolved by reducing SDS-PAGE and immunoblotted with anti-HPIV3 F antibody. (**B**) Proportion of HPIV3 F cleaved. ****P ≤ 0.0001 by two-way analysis of variance (ANOVA) and Tukey’s multiple comparisons *post hoc* test. (**C**) HAE supernatant HPIV3 titered in Vero cells infected in the presence or absence of 1 µg/mL TPCK-treated trypsin. **P ≤ 0.01 by two-way ANOVA and Sidak’s *post hoc* test. Values are means and SEM from three biological replicates.

Since the Calu-3 cell results suggested extracellular proteases contribute to HPIV3 F E108 cleavage, we assessed this effect in the more authentic lung model, HAE (34, 36, 37, 52). We tested whether HPIV3 F is extracellularly cleaved on HPIV3 F E108- or HPIV3 F K108-infected HAE cells by directly inhibiting serine proteases with aprotinin or by blocking the HPIV3 F cleavage motif with an anti-HPIV3 F VHH-Fc that blocks the HPIV3 F cleavage site (56). Both treatments significantly decreased the proportion of HPIV3 F cleaved for HPIV3 F E108 and HPIV3 F K108 suggesting extracellular serine proteases cleave HPIV3 F on HAE (Fig. 4A and B). Furthermore, the reduction in HPIV3 F K108 cleavage suggests that furin or other intracellular proteases only cleave about half of the F proteins, and additional cleavage occurs at the cell surface. Determination of the viral titer in the presence of L-1-tosylamido-2-phenylethyl chloromethyl ketone (TPCK)-treated trypsin demonstrated that aprotinin only prevented HPIV3 F cleavage, without affecting overall virus viability (Fig. 4C). Titers of the VHH-Fc-treated virus could not be completely recovered with trypsin treatment because the VHH-Fc neutralizes the virus by binding directly to the F proteins and holding them in their uncleaved precursor state (56). These results suggest that extracellular cleavage of HPIV3 F by serine proteases is critical for infectious HPIV3 F release in authentic lung models, and that membrane-bound or secreted proteases may be significant for HPIV3 spread in the human lung.

### Protease candidates for HPIV3 F processing and HN-F mediated cell-cell fusion identified with human genome CRISPRa pooled lentiviral screen

**Fig 5:**
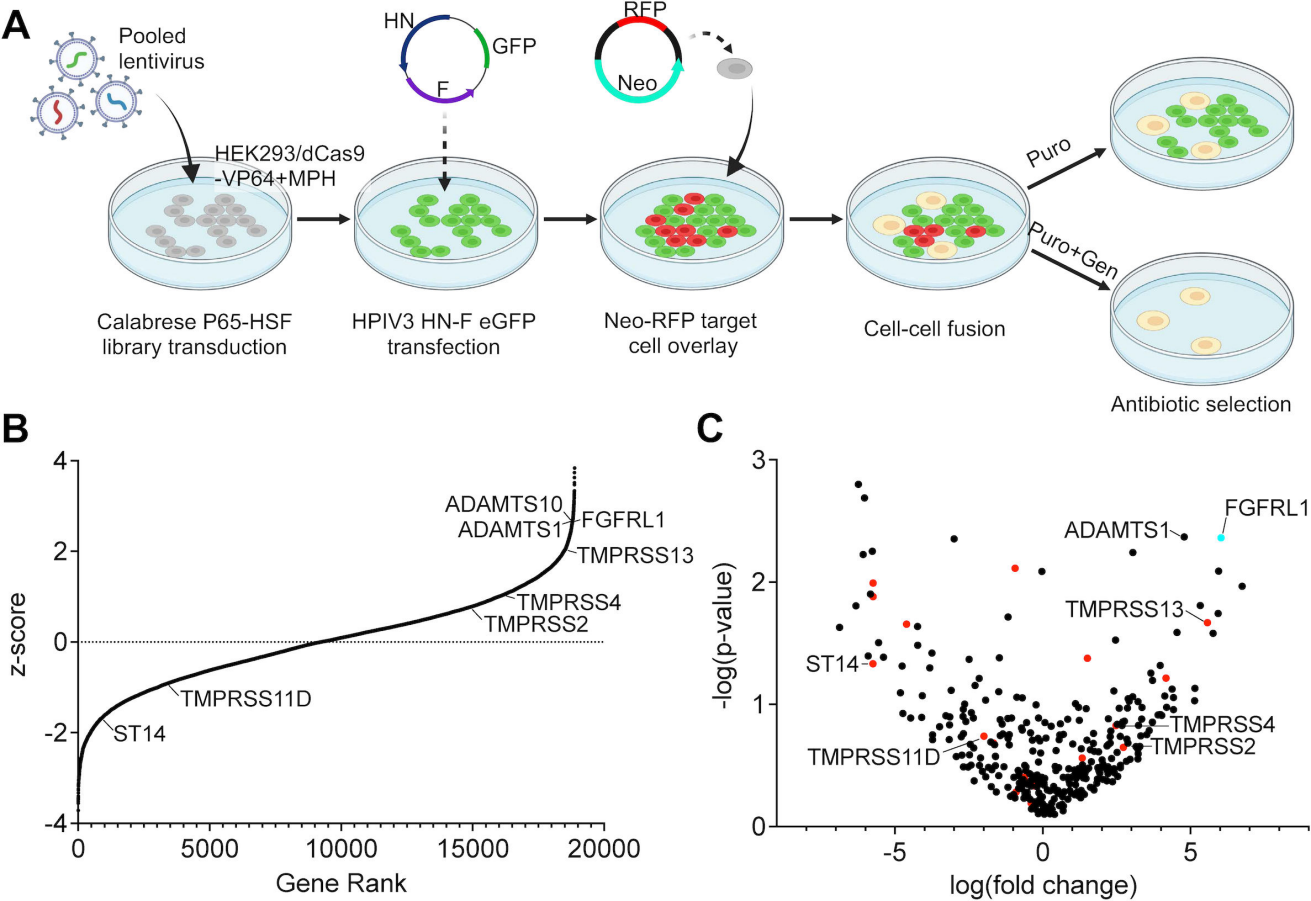
Whole human genome CRISPRa screen identified candidate proteases for HPIV3 F cleavage and infection. (**A**) Schematic of whole human genome lentiviral screen (created with BioRender.com). (**B**) Distribution of normalized z-scores (geneticin treated vs control) for all genes targeted by the sgRNA library. (**C**) Volcano plot of all human proteases expressed in human lung airway epithelium with serine proteases indicated in red. FGFRL1 was included as a positive control (turquoise).

To identify human proteases sufficient for HPIV3 F cleavage, we conducted a gain-of-function CRISPRa screen using antibiotic resistance gained via cell-cell fusion as a functional readout. The strategy is illustrated in Fig. 5A. HEK293/dCas9-VP64 + MPH cells [stably expressing the synergistic activation meditator system (57), GeneCopoeia Inc.] transduced with a pooled lentiviral human CRISPR activation library containing a puromycin resistance cassette and 56,762 single guide RNAs (sgRNAs; 3 sgRNAs/gene, targeting 18,885 genes) were transfected with HPIV3 HN, HPIV3 F E108, and enhanced green fluorescent protein (eGFP). The effector HPIV3 HN-F expressing cells were overlaid with target Neomycin-red fluorescent protein (RFP) expressing cells. The subset of effector cells bearing guide RNAs that induced the overexpression of an endogenous protease sufficient for HPIV3 F E108 cleavage fused with *Neo* expressing target cells and gained resistance to otherwise lethal concentrations of geneticin and puromycin.

Following antibiotic selection, syncytia were observed and enriched genes were identified with next-generation sequencing with 175× sgRNA coverage (Fig. 5A). Among the proteases identified to be uniquely expressed in permissive cells (Fig. 2C), TMPRSS13 was the most significantly enriched (Fig. 5B; Table S1). FGFRL1 was included in the volcano plot with all human proteases because overexpression of the gene has been previously shown to induce HEK cells syncytium formation independent of HN-F (58). Proteases enriched in the puromycin- and geneticin-treated group were considered to be potential proteases that cleave HPIV3 F E108 (Fig. 5C).

### Identification of proteases sufficient for HPIV3 F processing and HN-F-mediated cell-cell fusion

**Fig 6:**
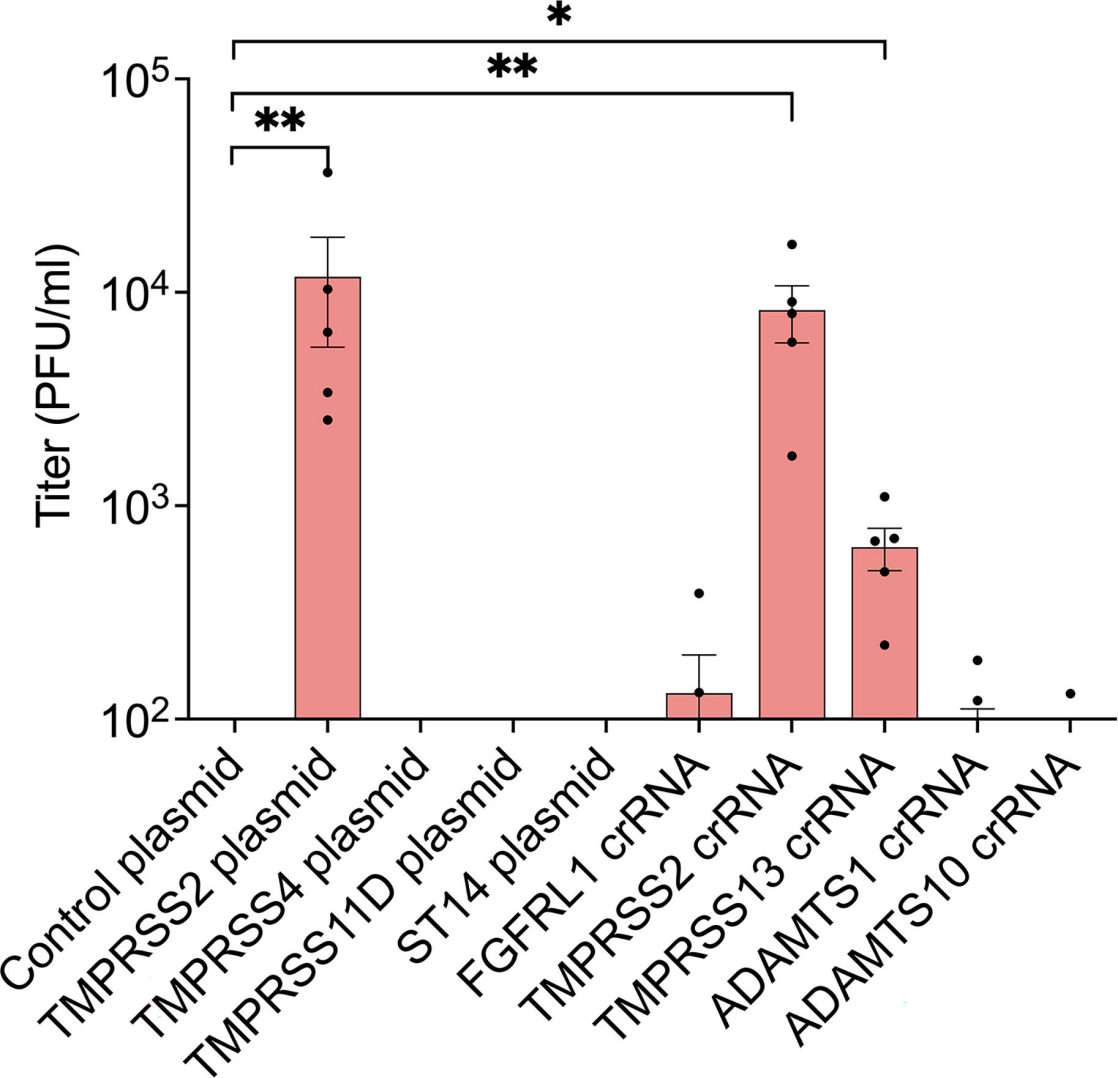
CRISPRa gain-of-function screen identifies TMPRSS2 and TMPRSS13 as sufficient for HPIV3 infection. HEK293/dCas9-VP64 + MPH cells were transfected with CRISPRa plasmids or tracrRNA:crRNA complexes for proteases significantly enriched in the lentiviral screen. Transfected cells were then infected with HPIV3 F E108 with JS strain background and infectious HPIV3 released by cells were titered. *P ≤ 0.05, **P ≤ 0.01 by two-way analysis of variance and Kruskal-Wallis *post hoc* test. Values are means and SEM from three biological replicates.

Based on the serine protease inhibitors’ inhibition of HPIV3 F E108 spread (Fig. 3 and 4), we evaluated serine proteases frequently exploited by respiratory viruses including TMPRSS11D (HAT) and Matriptase (ST14), as well as those enriched in the CRISPRa lentiviral screen, including TMPRSS2, TMPRSS4, and TMPRSS13 (54). Additionally, the lung protease ADAMTS1 and the related protease ADAMTS10 were enriched to similar levels to the positive control FGFRL1 and were therefore included. In the gain-of-function experiment, only TMPRSS2 or TMPRSS13 expression, conferred by their respective guide RNAs (gRNAs), were sufficient for infectious HPIV3 F E108 release by HEK293/dCas9-VP64 + MPH cells (Fig. 6). These results suggest that at least two extracellular serine proteases expressed in the human lung are sufficient for the spread of HPIV3 bearing the highly conserved cleavage motif observed in circulating strains.

### TMPRSS2 is sufficient for HPIV3 HN-F on the same cell to mediate cell-cell fusion

**Fig 7:**
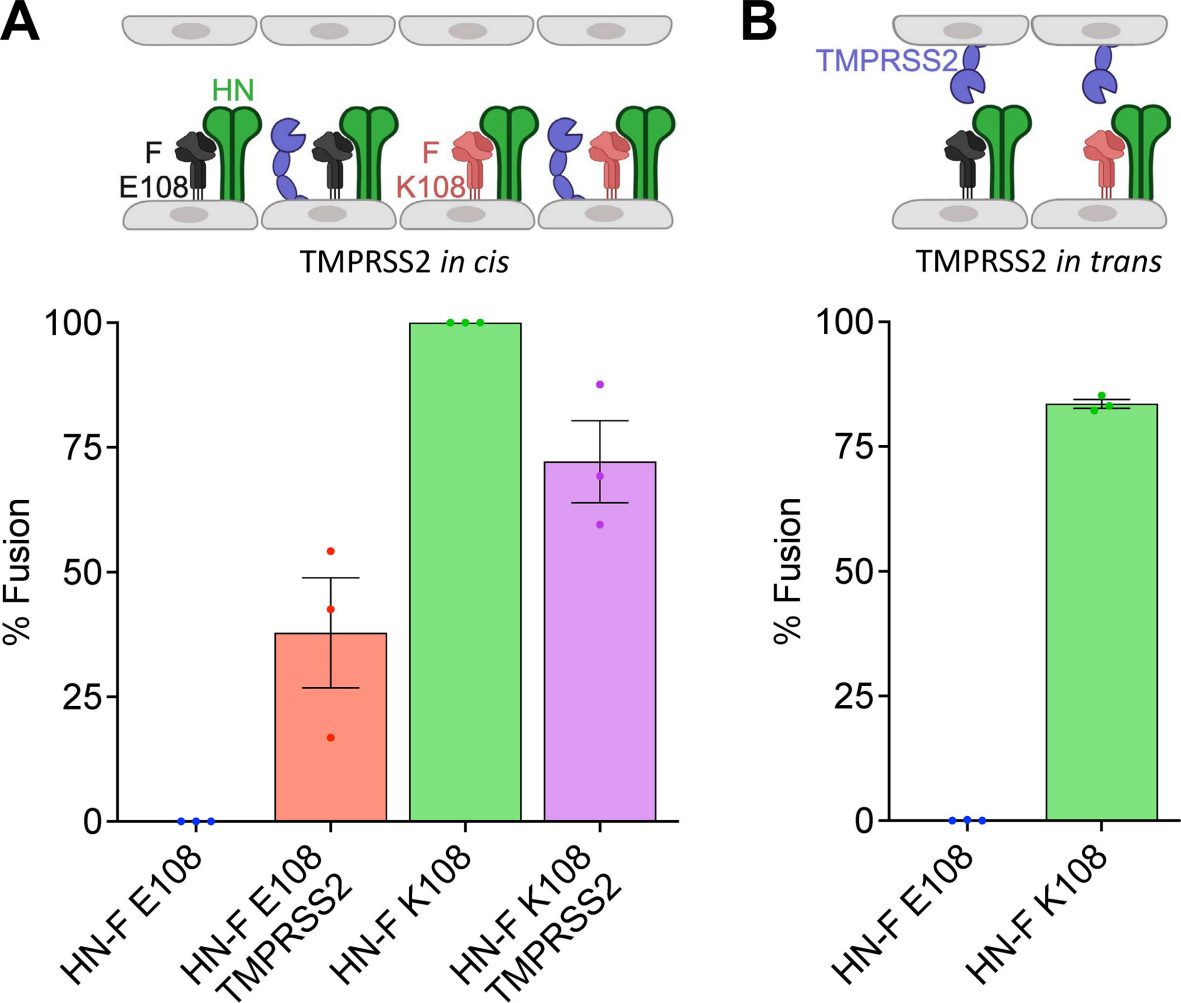
TMPRSS2 expression is sufficient for HPIV3 HN-F-mediated cell-cell fusion. Fusion activity measured with β-galactosidase complementation assay. HEK293T cells were transfected with the α subunit of β-galactosidase and HPIV3 F E108/HN (blue), HPIV3 F E108/HN/TMPRSS2 (red), HPIV3 F K108/HN (green), or HPIV3 F K108/HN/TMPRSS2 (purple). (**A**) Cells were incubated for 16 hours with HEK293T cells expressing the Ω subunit of β-galactosidase. (**B**) Cells were then incubated for 16 hours with HEK293T-TMPRSS2 (TMPRSS2 *in trans*) cells expressing the Ω subunit of β-galactosidase. Values are means and SEM from three biological replicates. Schematic created with BioRender.com.

To determine whether extracellular serine proteases are sufficient to enable HN-F E108-mediated cell-cell fusion, we examined the effect of TMPRSS2 expression on fusion promoted by HPIV3 HN and F E108 or F K108 with a β-galactosidase (β-Gal) complementation cell-cell fusion assay (13, 59). The assay measures the direct impact of proteases on fusion protein activity. HEK293T cells are not permissive to field strain HPIV3, do not constitutively express *TMPRSS2,* and do not support HN-F-mediated cell-cell fusion (Fig. 7). When their endogenous *TMPRSS2* gene was activated with CRISPRa, these cells gained the ability to cleave HPIV3 F E108 and fused with target cells (Fig. 7).

Cells bearing HPIV3 HN and F K108 fused without *TMPRSS2* expression, as expected since the F is cleaved by furin, and fused slightly less when *TMPRSS2* was expressed. TMPRSS2 expression only on the target cells (i.e., on adjacent cells in the culture) was not sufficient for HN-F E108-mediated cell-cell fusion (Fig. 7B). TMPRSS2 expressed on the HN-F expressing (Fig. 7A) or infected cells (Fig. 6) is sufficient for HN-F-mediated membrane fusion. However, TMPRSS2 on the surface of a target cell alone is not sufficient to permit entry by virions with uncleaved F proteins, as shown in Fig. S1, supporting the evidence in Fig. 3 that cleavage of F must occur upon release from infected or HN-F expressing cells.

### TMPRSS2 or TMPRSS13 are sufficient, but not required for infectious HPIV3 release by Calu-3 cells

**Fig 8:**
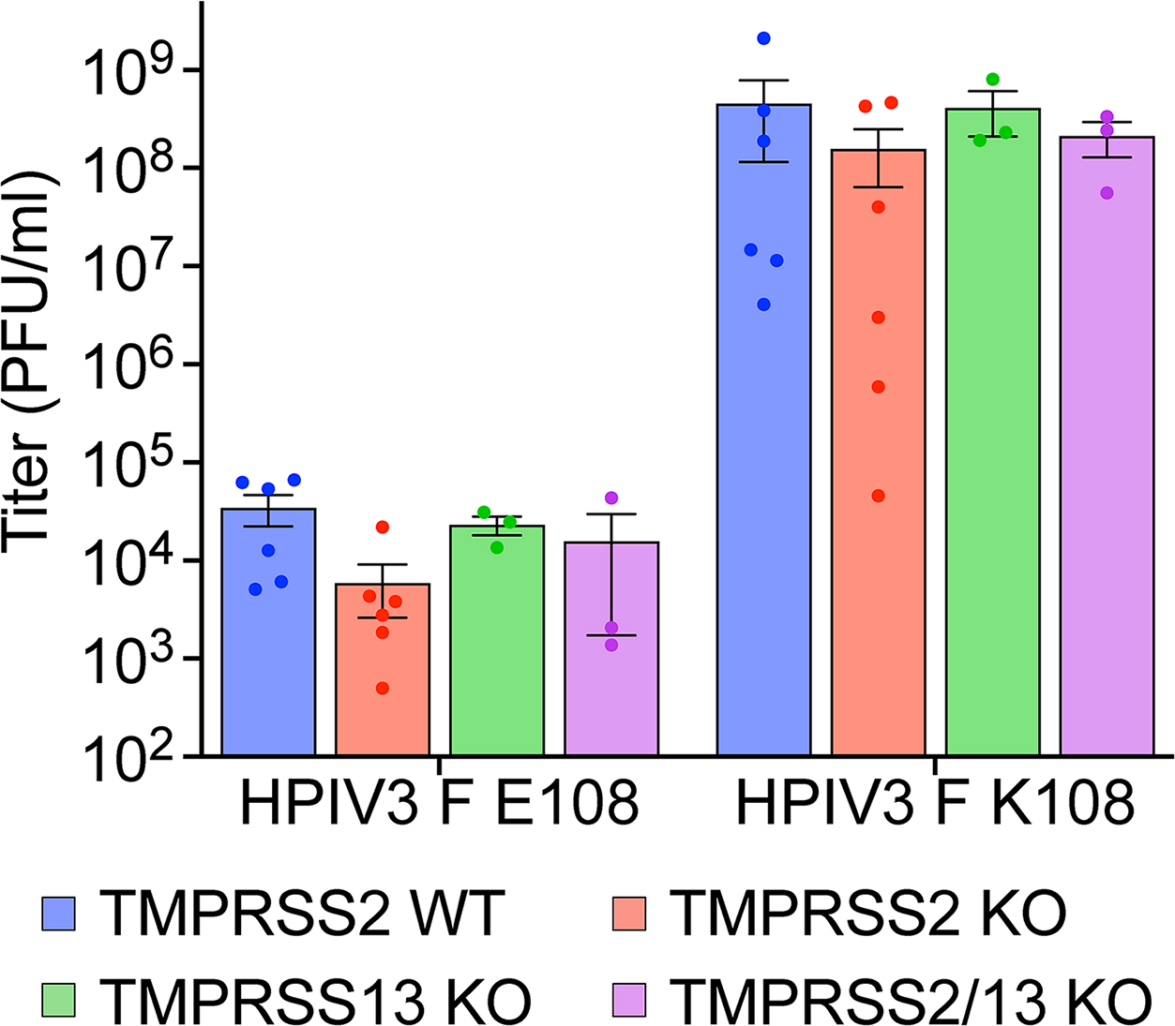
TMPRSS2 and TMPRSS13 are not necessary for infectious viral particle production by Calu-3 cells. WT, TMPRSS2 KO, TMPRSS13 KO, and TMPRSS2/13 KO Calu-3 cells were inoculated with HPIV3 F E108 or HPIV3 F K108 with JS strain background. HPIV3 titers in Calu-3 culture media. Values are means and SEM from three to six biological replicates.

To determine the extent to which TMPRSS2 and TMPRSS13 are necessary for HPIV3 F cleavage, we determined whether knocking out (KO) TMPRSS2 and TMPRSS13 in Calu-3 cells abrogated their ability to cleave HPIV3 F E108 and release infectious virions. Using commercial TMPRSS2 KO Calu-3 cells (Abcam), we excised 159 bp in TMPRSS13 exon 2 using CRISPR with two guides (Fig. S2), then infected the wild-type (WT) and KO cell lines with HPIV3 F E108 or HPIV3 F K108. The TMPRSS2, TMPRSS13, and TMPRSS2/13 KO cell lines had reductions in HPIV3 F E108 titers relative to the WT Calu-3 cell control but retained the ability to release infectious virions (Fig. 8), indicating that multiple proteases expressed in lung cells are sufficient to cleave HPIV3 bearing F E108.

## DISCUSSION

Among respiratory viruses with type I fusion proteins—including paramyxoviruses, influenza viruses, and coronaviruses—fusion protein cleavage by host proteases is a requirement for cell entry and these host-pathogen interactions are critical to the virus’ life cycles (33, 53, 60–63). The HPIV3 strains previously available to virologists for sequencing and study had a highly conserved dibasic R-X-K-R cleavage motif (Fig. 1) that was classically considered to be the minimal furin cleavage motif. Therefore, it was thought that the F of HPIV3—and the F of other viruses bearing dibasic cleavage motifs—was cleaved intracellularly by the ubiquitously expressed proprotein convertase furin (30, 34, 64). However, we show that the F of unpassaged field strains of HPIV3 in fact contain a monobasic cleavage site. When circulating strains of HPIV3 infect immortalized cell cultures, the virions that are released require exogenous trypsin to enter cells, and emerge as trypsin-independent escape mutants bearing F with R-X-K-R after passaging in immortalized monolayer cell culture (34, 53). Field strains that have never been passaged in cell culture virtually all bear R-X-E-R at their monobasic cleavage site (34)—not a target for furin cleavage.

The related morbilliviruses have until recently also been thought to all contain a polybasic furin cleavage signal in their F protein to permit processing and infectivity. However, a new feline morbillivirus (FeMV) was identified and found to lack a polybasic furin cleavage signal in its F protein and to possess instead a monobasic cleavage site processed by cysteine proteases, not by furin (65). For FeMV, the sequence and glycoproteins were obtained directly from a clinical sample, as we have done for field strains of HPIV3, and not from viruses that had been isolated in cell culture as was previously standard for morbilliviruses. While FeMV may employ a different activation strategy to all other morbilliviruses, it is also possible that this difference could instead relate to its analysis without laboratory adaptation. In this case, FeMV would parallel HPIV3 in challenging the pathogenetic relevance of the canonical furin processing pathway.

Multibasic cleavage site variations observed in other respiratory viruses, including the paramyxovirus Newcastle disease virus (NDV) and avian influenza viruses, are defining features of highly pathogenic strains (66, 67). It has been shown that monobasic or dibasic cleavage motifs regulate viral pathogenicity and impact the viral lifecycles and tissue tropism (62, 66–68). Dibasic or multibasic cleavage motifs enable intracellular fusion protein cleavage by furin or related proteases during transit through the trans-Golgi network, and influenza virus and NDV with multibasic cleavage motifs have been shown to have a dramatically expanded range of cells that cleave their fusion proteins, resulting in more severe infections in domestic poultry and other natural hosts including waterfowl (33, 66, 67, 69). In addition to its canonical avian host, highly pathogenic avian influenza bearing multibasic cleavage motifs can also be transmitted to humans and cause lethal infections (70–74). Our results suggest that a multibasic cleavage site in F is strongly negatively selected against *in vivo*; however, it remains unclear what consequences the emergence of such a variant would have on human parainfluenza virus infection outcomes. Identifying the host factors that interact with HPIV3 F’s monobasic cleavage motif may help to predict how antivirals including protease inhibitors direct viral evolution.

HPIV3 clinical isolates’ features are conserved when propagated in authentic lung models, including primary human airway epithelium grown at an air-liquid interface or lung organoids, suggesting that these systems faithfully recapitulate the virus’s natural environment and bear key host factors that the virus exploits to proliferate in human lungs (34, 35, 38, 52, 75). Comparison of the proteomes of authentic lung models and immortalized cell cultures revealed that extracellular proteases required by other trypsin-dependent respiratory viruses are enriched in authentic lung models (53–55, 61, 76, 77). Extracellular serine protease inhibitors significantly reduced the F processing and infectious virus release for field strains (bearing F E108). When cell surface proteases are overexpressed, the extracellular serine proteases TMPRSS2 and TMPRSS13 are each sufficient for the release of infectious HPIV3 bearing the circulating strain cleavage motif. These results suggest that HPIV3 F is not cleaved during intracellular trafficking but is instead cleaved later at the cell surface.

The sufficiency of TMPRSS2, TMPRSS13, and potentially other yet unidentified proteases for HPIV3 F cleavage highlights HPIV3’s exploitation of multiple cell surface proteases. This redundancy affords the virus multiple avenues of F activation, and the cleavage motif protease selectivity has been evolutionarily honed to achieve optimal F cleavage necessary for HPIV3 spread. Notably, the HPIV3 F cleavage site is highly specific and other proteases frequently exploited by respiratory viruses—including TMPRSS11D (HAT), Matriptase (ST14), and TMPRSS4 (55, 60, 78, 79)—are not sufficient for activation (Fig. 6). Moreover, circulating strains of both severe acute respiratory syndrome coronavirus 2 spike and highly pathogenic avian influenza hemagglutinin can also be cleaved by TMPRSS2 and TMPRSS13. Mutating the cleavage motifs of either virus from dibasic to monobasic residues has a minimal effect on TMPRSS2 cleavage, but significantly reduces cleavage by TMPRSS13, due to TMPRSS13’s increased catalytic activity with dibasic cleavage motifs (78–80). The combination of TMPRSS2 and TMPRSS13 activities may therefore be necessary for cleaving the optimal proportion of HPIV3 F necessary for viral spread in the human lung.

The HPIV3 fusion complex operates under specific constraints that govern viability. The several key functions of the complex exist in a relationship to each other that modulates entry. Receptor avidity, receptor cleavage, stabilization of F by HN prior to receptor engagement, and activation of F by HN upon receptor engagement cooperate to regulate the entry process in each environment (8, 12, 34, 35, 37, 39). This complex relationship also offers the possibility of adaptation to a range of environments and escape from inhibitors (8, 35). The specificity of F protein cleavage shifts the point at which the virus becomes infectious to the location of the requisite proteases. The conservation of HPIV3 F E108 in circulation suggests that cleavage specificity is also an important regulator of viral fitness that uniquely serves as a determinant of viral infectivity by contributing to the maintenance of the pre-fusion state of F. The stabilization of F by HN prior to receptor engagement complements selective cleavage of F to offer redundant approaches to the preservation of the pre-fusion F.

It remains unclear what the advantage of bearing F E108 is to viruses *in vivo* and thus why F E108-bearing strains, resistant to furin cleavage, are entirely dominant in circulation. One possibility is that the advantage relates to transmission, and this is a subject of ongoing study. The results presented here suggest that the fusion protein is critical to the fine tuning of the parainfluenza entry mechanism in a crucially overlooked way. We have previously described how HN facilitates infection under circumstances of low avidity and high neuraminidase activity to escape target mimics and receptors on the membrane of the parent cell (12, 34, 35, 37, 39). HN also stabilizes the activation-ready F so that the virus can traverse the airway without activating F in the wrong location (8, 81). The conservation of HPIV3 F E108 suggests that HPIV3 in circulation utilizes the timing of F’s cleavage as another mechanism for regulating the fusion complex, in an intimate interaction with the host. F E108 fundamentally alters which host proteases the virus exploits and when the virus becomes infectious.

This proposed specificity of F protein cleavage, as a binary determinant of viral infectivity, reveals a previously unexplored feature of paramyxovirus lifecycles. Our results subvert the accepted paradigm whereby HPIV3 F cleavage processing occurs intracellularly during transit to the cell surface, and show that the cleavage necessary for viral infectivity occurs at the cell surface. F cleavage serves as a determinant of F’s ability to function and the HPIV3 F cleavage site provides a “ticket” for activation at any site in the cell where a protease recognizing that cleavage motif can be found. Fusion proteins across enveloped viruses bear distinct cleavage motifs that allow them to selectively time and locate activation at the site of specific proteases. In response to host-specific environmental settings and/or to adapt to opportunities, HPIV3—and likely most respiroviruses—can adapt the cleavage motif at F residue 108 to utilize the proteases available. In humans, the virus exploits a subset of host cell surface proteases to allow for regulation of infectivity at the optimal location and permit viral spread.

## MATERIALS AND METHODS

### HPIV3 genome sequencing

Shotgun RNA sequencing metagenomic reads (82) were adapter- and Q20 quality-trimmed using Trimmomatic v.0.39 (83). Reads for the viral genome were aligned to the reference sequence for the HPIV3 expressing mCherry (GenBank accession no. OP821798) using bwa-mem v.0.7.17-r1188 (https://arxiv.org/abs/1303.3997), and variant allele frequencies were extracted using bcftools v.1.9 (84) and annotated via VarScan v.2.3 (85) (Bioproject PRJNA1132297).

### Cells and viruses

HEK293T [human kidney epithelial, American Type Culture Collection (ATCC)] cells were grown in Dulbecco’s modified Eagle’s medium (DMEM) (Gibco) supplemented with 10% fetal bovine serum (FBS) and 1% penicillin-streptomycin (P/S) (Gibco) at 37°C in 5% CO_2_. HEK293/dCas9-VP64 + MPH (Genecopoeia) cells were grown in DMEM (Gibco) supplemented with 10% FBS, 20 µg/mL blasticidin (Gibco), and 250 µg/mL hygromycin (Invitrogen) at 37°C in 5% CO_2_. A549 (adenocarcinoma human alveolar basal epithelial cell, ATCC) cells were grown in F-12K medium (ATCC) supplemented with 10% FBS and 1% P/S (Gibco) at 37°C in 5% CO_2_. Calu-3 (lung adenocarcinoma, ATCC), TMPRSS2 KO Calu-3 (Abcam), and HEPG2 (hepatocellular carcinoma, ATCC) cells were grown in Eagle’s minimum essential medium (ATCC) supplemented with 10% FBS and 1% P/S (Gibco) at 37°C in 5% CO_2_.

For infection and production of viruses, HEK293T, Calu-3, A549, and HEPG2 cells were grown to 70% confluency in 96-well plates and infected with 2,000 plaque forming units (PFU) of HPIV3 F E108 mCherry or HPIV3 F K108 mCherry in Opti-MEM for 90 minutes. Virions released by the cells were titered by limiting dilution infection of Vero cells (limit of detection 100 PFU/mL).

### Human airway epithelial culture

HAE EpiAirway AIR-100 (MatTek Corporation) cultures consist of pseudostratified, human-derived tracheo/bronchial mucociliary epithelium that recapitulate *in vivo* human airway tissue (34, 35, 38, 52). Upon receipt from the manufacturer, HAE cultures were transferred to six-well plates containing 1.5 mL of AIR-100-ASY assay medium (MatTek Corporation) per well with the apical surface remaining exposed to air and incubated at 37°C in 5% CO_2_ overnight prior to infection.

### Recombinant virus growth and purification

Recombinant viruses were generated by reverse genetics as previously described (35, 86) using an HPIV3 clinical isolate (CI-1)(34, 36, 38) or JS strain (44) background harboring single mutations in F (E108 or E108K) and a recombinant eGFP or mCherry cassette between genes P and M. Resulting viruses were propagated using the HAE EpiAirway AIR-100 system (MatTek Corporation). Viruses were titered by limiting dilution infection of Vero cells (ATCC), and infected cells were quantified using a Cytation5 (Agilent). All recombinant viruses were sequenced using metagenomic next-generation sequencing (82). All experiments comparing HPIV3 F E108 and HPIV3 F K108 were performed with viruses with identical genomes differing only at residue 108.

### RNA-seq

Total RNA was purified from HAE and HEK293T cells using the Direct-zol RNA MicroPrep kit (Zymo Research). mRNAs were enriched from total RNA samples with a poly-A pull-down, and the library was constructed using Illumina TruSeq chemistry. Libraries were sequenced using the Illumina NovaSeq 6000 (Illumina) at Columbia Genome Center. Samples were multiplexed in each lane, yielding a targeted number of single-end/pair-end 100 bp reads for each sample. Real-time analysis (RTA; Illumina) was used for base calling and bcl2fastq2 (version 2.19) converted BCL to fastq format and adaptor trimming. Pseudoalignment to a kallisto index was created from transcriptomes (Ensembl v.96, Human:GRCh38.p12; Mouse:GRCm38.p6) using kallisto (0.44.0).

The gene expression data for Calu-3, A549, and HEPG2 cells were extracted from the 22Q1 public data release from the DepMap at the Broad Institute Cancer Cell Line Encyclopedia expression from https://portals.broadinstitute.org/ccle, accessed on 2/7/2022) (Bioproject PRJNA1132297).

### β-Galactosidase complementation fusion assay

Cell-cell fusion was quantified by measuring β-Gal complementation as performed previously (13, 59). Receptor-bearing cells expressing the omega peptide of β-Gal were mixed with cells co-expressing envelope glycoproteins [HN T193A (87) and F (E108 or K108)], control or TMPRSS2 CRISPRa plasmid (Santa Cruz Biotechnology), and the α peptide of β-Gal. Cell-cell fusion was quantified by measuring galactosidase activity in fused cell lysates (Galacton-Star substrate; Applied Biosystems, T1012), and luminescence was read after 1 hour on a Tecan M1000 Pro. Percent fusion was quantified by comparing relative luminescence units (RLUs) of each condition to RLUs with HN-F K108.

### Cleavage of HPIV3 F and inhibition of cleavage by protease inhibitors

The effect of serine protease inhibitors on the release of infectious HPIV3 was assessed by titering HPIV3 F E108 or HPIV3 F K108 released by HPIV3-infected Calu-3 cells or HAE cultured in the presence of the extracellular serine protease inhibitors aprotinin or leupeptin. Viruses and treatments were added to Calu-3 cell culture media or the apical surface of HAE cultures. Cells were infected with HPIV3 F E108 or HPIV3 F K108 for 90 minutes at 37°C, followed by inoculum removal and incubation at 37°C for the remainder of the experiment. One day post infection, cells were treated with the listed concentrations of aprotinin (Abcam) or leupeptin (Tocris). Following overnight incubation with inhibitors, virions released into the cell culture media were titered by limiting dilution infection of Vero cells. The proportion of HPIV3 F cleaved on the released virions was determined by resolving the viral proteins on 4%–20% Tris-glycine SDS-PAGE (Novex) under reducing conditions, and then detecting F proteins with rabbit anti-HPIV3 F HRC antibody (Genscript) and WesternBreeze Chemiluminescent Kit (Invitrogen).

### Whole human genome CRISPRa lentiviral screen

Effector cells were prepared by transducing HEK293/dCas9-VP64 + MPH with the Human Calabrese CRISPR activation pooled library set A (88) (Addgene #92379) at a low multiplicity of infection (MOI;~0.5) for 16 hours in DMEM supplemented with 10% FBS, 1% P/S, and 5 µg/mL polybrene. Twenty-four hours post infection, selection for transduced cells was started with 1 µg/mL puromycin. Forty-eight hours post transfection, effector cells were transfected with plasmids carrying laboratory-adapted HPIV3 F bearing E108, laboratory-adapted HPIV3 HN, and eGFP. In parallel, target cells were transfected with a plasmid carrying *Neomycin* and *RFP* cassettes. Following transfection, the target cells were overlaid onto the effector cells. Forty-eight hours after overlaying the cells, control cells were cultured in DMEM supplemented with 10% FBS, 1% P/S, and 0.5 µg/mL puromycin, and fused cells were selected via culturing in DMEM supplemented with 10% FBS, 1% P/S, 0.5 µg/mL puromycin, and 1 mg/mL geneticin. After 96 hours of antibiotic selection, DNA was extracted from both puromycin only and puromycin + geneticin selected cell cultures with a QIAamp DNA mini kit (Qiagen).

Calabrese sgRNA template sequences were amplified from genomic DNA in 10 parallel 50 µL twenty cycle PCR reactions/sample. Each PCR reaction contained 1 µg genomic DNA, AmpliTaq Gold 360 Master Mix, staggered forward primers, and reverse primers. After ten cycles, samples were then bead-purified using Omega Mag-Bind beads (Omega Biotek) and eluted in 100 µL. Ten microliters of this was then taken over into a second PCR reaction in which oligos were used that annealed to the Read 1 and Read 2 sequences, and contained overhangs that included Illumina indexes and P5/P7 sequences. Ten cycles of PCR were performed. Samples were then pooled and resolved on a 1.5% agarose gel, purified, and sequenced on an Illumina NextSeq 500/550 with 75 cycles in the Read 1 orientation.

Sequencing reads from whole genome CRISPRa lentiviral screenings had barcodes removed with cutadapt (Galaxy version 4.8 + galaxy0). The abundance of each sgRNA was assessed and normalized among samples with the use of MAGeCK (Galaxy version 0.5.9.2.4). About 85% of reads contained sgRNA sequences that aligned to the Calabrese Set A Target Genes library (88). sgRNA read counts were input into MAGeCK (Galaxy version 0.5.9.2.1) to obtain *P*-values and log fold change for gene enrichment relative to the no geneticin control (89–91).

### Individual protease gain-of-function screen

Sufficiency of individual proteases for infectious HPIV3 release was determined by transfecting HEK293/dCas9-VP64 + MPH cells in 96-well poly-d-lysine biocoated culture wells with 0.15 µg of CRISPR activation plasmids (Santa Cruz Biotechnology) or CRISPRa MS2 tracrRNA:crRNA (Dharmacon), and 30 µL of Lipofectamine 2000 (Thermo Scientific). Twenty-four hours post transfection, cells were infected with HPIV3 F E108 or HPIV3 F K108 for 90 minutes at 37°C, followed by inoculum removal and incubation at 37°C for the remainder of the experiment. Three days post infection, virions in the cell supernatants were titered by limiting dilution infection of Vero cells.

### PCR

gDNA was isolated from cell lysates with a QIAamp DNA mini kit (Qiagen). DNA was amplified with AmpliTaq Gold 360 Master Mix (Applied Biosystems) following the manufacturer’s protocol. Ten microliters of gDNA was added to a 25 µL PCR reaction. Thermocycling conditions were as follows: 95°C for 3 minutes, 95°C for 30 seconds, annealing at 60°C for 30 seconds, 72°C for 30 seconds, and 72°C for 7 minutes, with steps 2–4 cycled 35 times. The annealing temperature was adjusted to 52°C for TMPRSS13 primers. PCR amplicons were resolved by 2% SeaKem LE Agarose (Lonza) gel electrophoresis and visualized by staining with SYBR Safe (Thermo Scientific).

### Statistics

Graphs were generated and statistical analysis was performed with GraphPad Prism 10. Results are means ± SEM unless otherwise stated. *P*-values less than 0.05 were considered statistically significant.

## SUPPLEMENTAL MATERIAL

Supplemental material mbio.02327-24-s0001.pdf Additional data in support of Figures 7 and 8.

Table S1 mbio.02327-24-s0002.pdf Whole human genome CRISPRa screen.

ASM does not own the copyrights to Supplemental Material that may be linked to, or accessed through, an article. The authors have granted ASM a non-exclusive, world-wide license to publish the Supplemental Material files. Please contact the corresponding author directly for reuse.
