# Supplementary material for: Human parainfluenza virus 3 field strains undergo extracellular fusion protein cleavage to activate entry
Source: mBio. 2024 Oct 9;15(11):e02327-24. doi: 10.1128/mbio.02327-24 (PMC11559058; doi:10.1128/mbio.02327-24)
Supplement: Supplemental material — Additional data in support of Figures 7 and 8. [file mbio.02327-24-s0001.pdf]

Supplementary materials for

Human parainfluenza virus 3 field strains undergo extracellular fusion protein activation to activate entry.

Kyle Stearns, George Lampe, Rachel Hanan, Tara Marcink, Stefan Niewiesk, Samuel H. Sternberg, Alexander L. Greninger, Matteo Porotto, Anne Moscona

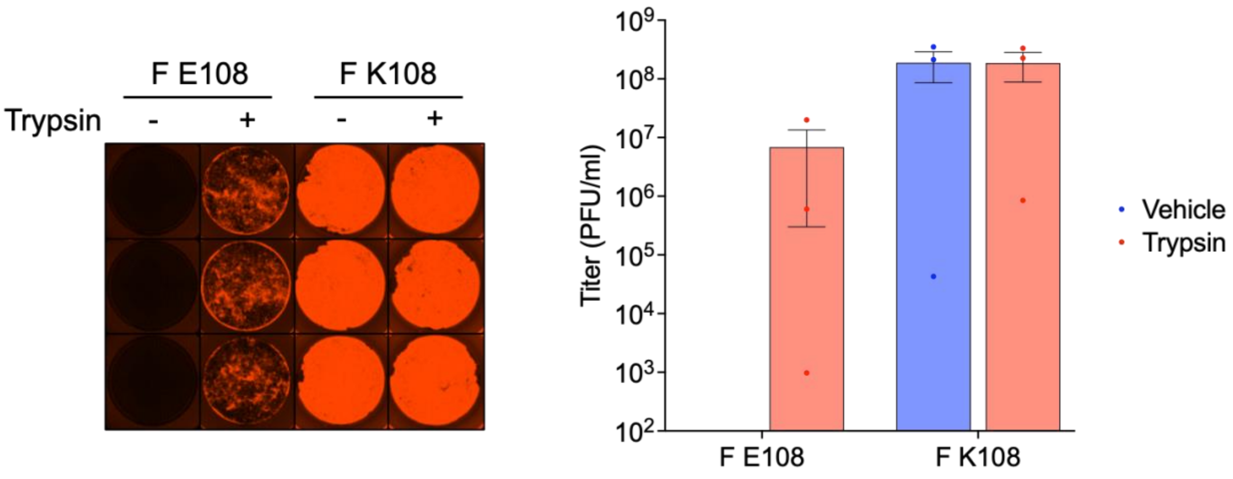

Fig S1: TMPRSS2 expression is not sufficient to permit entry by virions with uncleaved F proteins. Calu-3 cells were inoculated with HPIV3 F0 (uncleaved) E108 or HPIV3 F (cleaved) K108 in the presence of vehicle (blue) or exogenous TPCK-treated trypsin (red). (A) Representative images of Calu-3 cells 3 days post-inoculation. (B) Titer of infectious HPIV3 released by Calu-3 cells. Values are means and SEM from three biological replicates.

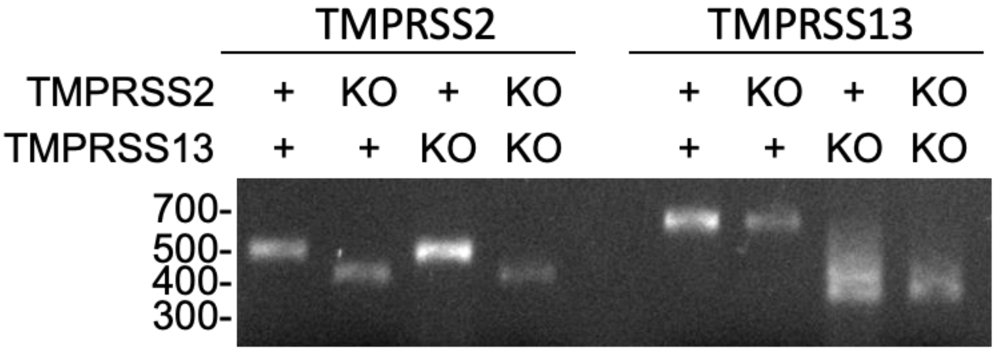

Fig S2: TMPRSS2 and TMPRSS13 gene deletions. PCR amplified genomic DNA from wild type, TMPRSS2 KO (77bp exon3 deletion), TMPRSS13 KO 159 bp exon 2 deletion) or TMPRSS2/TMPRSS13 KO Calu-3 cells. PCR reactions had forward and reverse primers flanking the deletion sites with a 472 bp predicted amplicon for TMPRSS2 exon 3 (left), or 620 bp predicted amplicon for TMPRSS13 exon 2 (right).

Table S1: whole human genome CRISPRa screen all genes table. Link to file: CRISPRa Screen pval lfc zscore.pdf
