# Supplementary material for: Human parainfluenza virus 3 field strains undergo extracellular fusion protein cleavage to activate entry
Source: mBio. 2024 Oct 9;15(11):e02327-24. doi: 10.1128/mbio.02327-24 (PMC11559058; doi:10.1128/mbio.02327-24)
Supplement: Table S1 — Whole human genome CRISPRa screen. [file mbio.02327-24-s0002.pdf]

Table S1

gene	pval	lfc	z-score	Gene Rank
**LRRIQ4**	0.00010151	-8.523	-3.715228707738740	1
**TREH**	0.00019016	-3.9635	-3.553376332283430	2
**NOX1**	0.00022269	-8.9571	-3.5116226107817300	3
**DDX28**	0.00025783	-3.5658	-3.4724870144989400	4
**PARD6A**	0.00028668	-8.0002	-3.4439131000829700	5
**IL20RB**	0.00029823	-8.7798	-3.433219072481850	6
**CCDC110**	0.000336	-6.8862	-3.400755129704050	7
**FBXL15**	0.00043881	-3.3523	-3.327077398583830	8
**IFT122**	0.0005453	-8.3522	-3.2660473991129000	9
**DCP1B**	0.00059094	-7.822	-3.2432173990792400	10
**LANCL1**	0.00059304	-5.3796	-3.242206630746440	11
**SOD3**	0.00073888	-5.5664	-3.179015886406270	12
**GPR148**	0.0007672	-3.6675	-3.1680976148991200	13
**CREBL2**	0.00078399	-5.0263	-3.1617985575526500	14
**NICN1**	0.00083592	-7.8609	-3.1430732603385100	15
**COL11A2**	0.00085901	-8.6712	-3.1350889812910100	16
**EME2**	0.0008632	-7.5461	-3.1336613208902700	17
**ALDH16A1**	0.00093297	-5.0306	-3.11077985503093	18
**MC2R**	0.00095186	-6.08	-3.1048555187840600	19
**H2AFY**	0.0011071	-1.2674	-3.059887709865830	20
**HIST1H4E**	0.0011187	-7.4673	-3.0567644907758200	21
**HMGB3**	0.0011879	-8.2354	-3.0387270629251500	22
**TXNIP**	0.0012503	-8.1794	-3.0232688365316700	23
**MAN1C1**	0.001274	-0.15375	-3.017582123481480	24
**RIMS4**	0.0013002	-8.3383	-3.011407063126570	25
**EPC1**	0.0013338	-8.7705	-3.0036523052184000	26
**DDX59**	0.0013988	-5.6848	-2.9891442803320500	27
**CNGB3**	0.0014035	-7.2087	-2.988119267250490	28
**PADI4**	0.0014256	-6.1306	-2.983341153770360	29

**C12orf65**	0.001436	-3.5868	-2.9811159887517200	30
**CLEC12A**	0.0014555	-7.0013	-2.9769831673100600	31
**CDYL**	0.0015011	-4.9153	-2.967512584628060	32
**ADGRG6**	0.0015515	-3.8391	-2.9573457165964100	33
**PMVK**	0.0015714	-8.4345	-2.9534141682332200	34
**SLC2A9**	0.0015787	-7.5172	-2.951983313617750	35
**SPPL2A**	0.0015866	-6.2331	-2.950441633495450	36
**ADAD2**	0.0015981	-6.191	-2.9482098748109900	37
**KANSL2**	0.001616	-2.1548	-2.9447650454385200	38
**KREMEN1**	0.001616	-3.8971	-2.9447650454385200	39
**METTL7B**	0.0016244	-7.1033	-2.943160448662360	40
**CALR**	0.0017041	-6.1778	-2.928300937645660	41
**DZANK1**	0.0018384	-8.0116	-2.904636368235890	42
**APOM**	0.0020509	-4.6019	-2.87022485862863	43
**TASP1**	0.0020509	-6.0297	-2.87022485862863	44
**PRELID1**	0.0020624	-1.3751	-2.868456437253050	45
**IFNA21**	0.0020881	-5.0301	-2.8645365193802800	46
**ZNF136**	0.0021012	-7.0469	-2.8625552418387100	47
**SECISBP2**	0.0022145	-7.4025	-2.845871958298470	48
**FILIP1L**	0.0022271	-3.6822	-2.844064716932210	50
**USP17L2**	0.0022271	-1.9262	-2.844064716932210	49
**SPHK1**	0.002331	-4.7758	-2.829505491712840	51
**SOST**	0.0023572	-6.3293	-2.825927045684050	52
**SPRR2A**	0.0023782	-1.5176	-2.823084727018990	53
**ORMDL2**	0.00246	-7.9519	-2.812225484010700	54
**LGALS3BP**	0.0024868	-3.8507	-2.8087386768335	55
**VSIG2**	0.002491	-8.0342	-2.808195319881560	56
**WDR20**	0.0025387	-2.7587	-2.8020817848858	57
**ZNF705A**	0.0025623	-7.9706	-2.7990953639281700	58
**C1orf122**	0.002619	-4.4111	-2.79202072991945	59
**LRRK1**	0.002619	-6.2869	-2.79202072991945	60
**WDR92**	0.0027066	-2.7432	-2.781358035730000	61

**IKBIP**	0.0027344	-7.0954	-2.7780393175420200	62
**PRRG2**	0.0027407	-7.8752	-2.777291468909220	63
**KLB**	0.0027737	-6.8749	-2.7733993344886100	64
**RTP5**	0.0028566	-2.5838	-2.7638031153393800	65
**SF1**	0.0029253	-5.4059	-2.756039111345890	66
**MCRS1**	0.0029337	-6.0272	-2.7551011081245100	67
**PLK2**	0.0029862	-2.1578	-2.7492928463101800	68
**PXMP2**	0.0030119	-7.4676	-2.7464830380806300	69
**WDR64**	0.0030229	-3.6685	-2.7452869928543400	70
**GPSM3**	0.0030276	-3.0446	-2.7447771504934200	71
**LAMP3**	0.003111	-7.4795	-2.735846718611210	72
**OR5M3**	0.0031257	-8.0469	-2.7342950412220900	73
**PHOSPHO1**	0.0032081	-3.399	-2.7257169166738200	74
**FAM171A1**	0.0032789	-5.7906	-2.718503302060540	76
**FMO4**	0.0032789	-4.145	-2.718503302060540	75
**ZFP37**	0.0034179	-6.5681	-2.704739360481120	77
**CCDC151**	0.0034775	-7.2126	-2.698991266472070	78
**AGAP6**	0.0035407	-8.0717	-2.6929918424476500	79
**B3GNT5**	0.0035564	-8.0839	-2.6915163974952200	80
**ZDHHC6**	0.0036855	-9.2023	-2.679600821990250	81
**TMEM198**	0.0037825	-3.9256	-2.6708919138384900	82
**RLN1**	0.0038045	-2.3592	-2.668944588982440	83
**SLC46A3**	0.003836	-6.922	-2.666173874950130	84
**ZBTB6**	0.0038785	-6.1778	-2.6624677508038600	85
**SPATA32**	0.0039514	-3.1173	-2.6561945724503000	86
**DDX18**	0.0041119	-2.6713	-2.6427406433246600	87
**OR10K1**	0.0041324	-6.7259	-2.6410562234897300	88
**PTGR2**	0.0041324	-7.1186	-2.6410562234897300	89
**ZNF426**	0.0041392	-5.8495	-2.640499140321050	90
**MBD3L1**	0.0041765	-1.5267	-2.637457861353340	91
**MICA**	0.0041765	-2.4077	-2.637457861353340	92
**MAPT**	0.0042772	-3.7673	-2.6293669337221900	93

**GNG10**	0.0042929	-5.7337	-2.628120868295620	95
**MAP1A**	0.0042929	-6.309	-2.628120868295620	96
**XG**	0.0042929	-5.2845	-2.628120868295620	94
**VEPH1**	0.0043758	-8.0151	-2.6216080988381200	97
**SPRTN**	0.0044409	-2.9959	-2.616570567378930	98
**GMPPA**	0.0044487	-4.9341	-2.615971425014600	100
**LOC100130[illegible]**	0.0044487	-0.1287	-2.615971425014600	99
**SEC23A**	0.0044592	-7.1841	-2.6151663672311200	101
**CPA2**	0.0045599	-7.1596	-2.607530282127590	102
**DSE**	0.0046234	-0.04611[illegible]	-2.602792115458320	103
**RGPD6**	0.0046413	-5.0066	-2.6014669693517300	104
**DCAF12L1**	0.0046643	-8.5866	-2.5997709459067200	105
**SPATA31E1**	0.0046701	-7.9216	-2.59934443130791	106
**OR8G5**	0.0047315	-8.1091	-2.594858008218690	107
**ZNF575**	0.0047803	-1.5995	-2.591329137755980	108
**GNG7**	0.0049376	-2.1897	-2.5801688956901600	109
**DVL1**	0.0049397	-7.6976	-2.580022058809970	110
**ZNF81**	0.0050079	-3.1369	-2.575283327965060	111
**APOBEC3G**	0.0050347	-1.731	-2.5734369064436900	112
**OR7C2**	0.0050976	-1.1572	-2.5691374693663300	113
**PRPS1L1**	0.0052062	-7.5634	-2.5618242154640500	115
**ZNF491**	0.0052062	-5.467	-2.5618242154640500	114
**ARHGEF16**	0.005255	-7.9705	-2.558582082864360	117
**SNW1**	0.005255	-7.7039	-2.558582082864360	116
**INPP5A**	0.0052563	-0.15678	-2.5584960814261200	118
**BTF3L4**	0.0053353	-6.1153	-2.553305016781290	119
**NKX1-2**	0.0054103	-3.7833	-2.5484396559488800	120
**PAFAH1B3**	0.0055677	-6.5675	-2.53842079430947	121
**PIP4K2B**	0.0055677	-7.3834	-2.53842079430947	122
**ERAL1**	0.0055708	-8.0261	-2.5382260118010700	123
**DHRS7C**	0.0055734	-5.3343	-2.538062720048720	124
**PSMA5**	0.0055918	-5.7666	-2.536909046742910	125

**SIX3**	0.0056857	-4.1717	-2.531073534853720	126
**SYPL2**	0.0056857	-5.4134	-2.531073534853720	127
**ACSM4**	0.0057214	-4.9755	-2.5288773518834800	128
**ABRACL**	0.0058436	-6.7687	-2.521450835381920	129
**OR5M8**	0.0058436	-6.842	-2.521450835381920	130
**MOG**	0.0059417	-0.14737	-2.515588001345190	131
**YOD1**	0.0059417	-6.0766	-2.515588001345190	132
**AGAP5**	0.00597	-7.3574	-2.5139126395560500	133
**FOXD4L1**	0.0061717	-5.7255	-2.502171811675150	134
**CDK5RAP2**	0.0061741	-2.1365	-2.502034168167340	135
**PTGIR**	0.0063719	-6.8317	-2.4908497260669400	136
**CT45A5**	0.0063844	-4.2952	-2.4901532969090100	137
**BUB1B**	0.0064862	-2.9276	-2.4845260743828100	138
**HIST1H4J**	0.0065135	-6.4952	-2.4830302961524800	139
**KLHL6**	0.0065497	-4.427	-2.481055411457950	140
**ANKRD13D**	0.0066714	-7.0678	-2.4744861126295000	141
**OR10Z1**	0.006706	-5.281	-2.4726377676003100	142
**SLC4A1**	0.0067186	-3.6467	-2.4719667639249100	143
**STK17B**	0.0068555	-4.2787	-2.464747045539950	144
**CFC1B**	0.0069499	-3.5479	-2.4598424770477300	145
**CLTCL1**	0.0069552	-6.2875	-2.4595688622016600	146
**KLRC4-KLR**	0.0069646	-7.1915	-2.459084035465700	147
**P4HA1**	0.0069646	-7.2276	-2.459084035465700	148
**HEPH**	0.0069846	-7.5478	-2.458054408330430	149
**SHC1**	0.0070213	-2.1495	-2.456171793310600	150
**FAM156B**	0.0070223	-5.5289	-2.4561206175629100	151
**NKAIN2**	0.0070842	-5.3853	-2.4529652909132400	152
**PTGER3**	0.0070958	-4.9255	-2.452376694244570	153
**THG1L**	0.0072033	-7.1264	-2.446962044904190	154
**PYHIN1**	0.007261	-2.7303	-2.444085092355260	155
**E2F3**	0.0074399	-5.4402	-2.4352914079101500	156
**C21orf91**	0.0075469	-6.1297	-2.430120563851150	158

**VDAC1**	0.0075469	-3.5169	-2.430120563851150	157
**HTRA2**	0.0077153	-0.93485	-2.422111832846280	159
**PSMB11**	0.0077153	-5.9793	-2.422111832846280	160
**KLRC4**	0.0077237	-6.7822	-2.421716390773740	161
**RBX1**	0.0077756	-7.5661	-2.419281483884620	162
**UQCC2**	0.0077914	-5.9023	-2.4185430609266400	163
**CEP78**	0.007868	-6.1386	-2.4149816810619300	164
**C3orf58**	0.0078758	-0.16229	-2.414620747171280	165
**CECR2**	0.0078926	-6.4264	-2.4138444177825300	166
**RIPOR1**	0.0078926	-6.7781	-2.4138444177825300	167
**SPANXN4**	0.0080432	-0.91045	-2.4069493404997400	168
**PCDHB10**	0.0081932	-5.6884	-2.4001936341534800	170
**TMEM174**	0.0081932	-3.2238	-2.4001936341534800	169
**ADAM17**	0.0081985	-0.028303	-2.3999569264315000	171
**LAMP1**	0.0081985	-4.162	-2.3999569264315000	172
**VWA1**	0.0083548	-2.5693	-2.393036005781470	173
**PNISR**	0.0084356	-6.8559	-2.3895026699828100	174
**CD8A**	0.0085672	-7.4105	-2.3838109673207200	175
**GTF2IRD1**	0.0086575	-5.585	-2.379949702136080	176
**FAM199X**	0.0088857	-2.7033	-2.370346921250820	177
**LRMDA**	0.0090388	-5.5023	-2.3640248209025600	178
**REM1**	0.0090808	-7.0578	-2.3623068758418800	179
**OR1J1**	0.0091642	-4.1693	-2.358916052594370	180
**ZNF429**	0.0091994	-8.8324	-2.357493015500090	181
**CDK19**	0.0092004	-3.7679	-2.3574526579802300	182
**RCCD1**	0.0092602	-2.5001	-2.3550462295725100	183
**FADS1**	0.0093399	-4.6766	-2.3518600521897800	185
**TMC8**	0.0093399	-3.5665	-2.3518600521897800	184
**C11orf45**	0.0094039	-5.7216	-2.3493186894796300	186
**GRIA1**	0.0094391	-5.5298	-2.34792738166801	188
**ZCCHC4**	0.0094391	-5.4682	-2.34792738166801	187
**AKAP4**	0.0094973	-0.5283	-2.3456369026878000	189

**A4GALT**	0.0095666	-7.4718	-2.3429255324791700	190
**OR51B6**	0.0096893	-8.1727	-2.3381667132365000	191
**KCTD14**	0.0096935	-5.6222	-2.338004753846540	192
**KL**	0.0097129	-4.1056	-2.3372574504899900	193
**PRSS56**	0.0097748	-1.6415	-2.334881700818050	194
**MYH15**	0.0098294	-7.4876	-2.332797012148730	195
**CALHM2**	0.0098304	-3.6881	-2.332758925447160	196
**PKMYT1**	0.0098892	-5.9373	-2.3305253540162300	197
**IL18R1**	0.0099516	-4.429	-2.3281676807569200	198
**DCAF8**	0.0099663	-6.0817	-2.3276141462213000	199
**GVQW2**	0.0099684	-7.5924	-2.3275351280498400	200
**RIBC2**	0.010017	-0.93642	-2.3257104690533900	201
**TLCD2**	0.010073	-5.8764	-2.323617537736140	203
**TOM1L2**	0.010073	-6.6963	-2.323617537736140	204
**VCX3B**	0.010073	-3.3794	-2.323617537736140	202
**DBR1**	0.010174	-0.71799	-2.3198683229085200	205
**MUM1**	0.010176	-7.9801	-2.3197944094729900	206
**PRSS16**	0.010185	-5.74	-2.3194619557521300	207
**C15orf40**	0.010286	-5.9148	-2.3157485537441400	208
**HOXB2**	0.010286	-6.3003	-2.3157485537441400	209
**IGF2BP2**	0.010286	-6.3809	-2.3157485537441400	210
**ATP6V0E2**	0.010299	-4.0746	-2.3152729034355700	211
**NDUFAF5**	0.010477	-3.5482	-2.3088122542123100	212
**CYP51A1**	0.010508	-5.3944	-2.3076968800399800	213
**TMED8**	0.010508	-6.4654	-2.3076968800399800	215
**UROC1**	0.010508	-5.5817	-2.3076968800399800	214
**MAPK8**	0.010539	-6.3633	-2.3065843694118800	217
**ZBTB37**	0.010539	-5.7534	-2.3065843694118800	216
**OR2T5**	0.01074	-6.4849	-2.2994393825029100	218
**SLC16A14**	0.01074	-6.6785	-2.2994393825029100	219
**FAM71F1**	0.010816	-5.2431	-2.296768107589350	220
**ERC1**	0.010857	-4.8425	-2.2953338030782400	222

**GPR183**	0.010857	-2.8646	-2.2953338030782400	221
**A3GALT2**	0.010948	-3.5108	-2.2921671136943200	223
**TIMM8B**	0.010962	-5.6547	-2.291681965213960	224
**MESDC1**	0.010968	-7.5763	-2.2914742094494500	225
**NPC1**	0.011115	-0.34041	-2.286414824416110	226
**HIC1**	0.011124	-6.3309	-2.286106960563410	227
**PMP22**	0.011247	-4.3666	-2.281921048411050	228
**FABP1**	0.011257	-7.6259	-2.2815824823316500	229
**INCA1**	0.011298	-5.1202	-2.2801970888355700	231
**PXN**	0.011298	-5.0956	-2.2801970888355700	230
**MOV10**	0.011388	-4.1639	-2.277171238350970	232
**EIF4G3**	0.011396	-3.038	-2.2769032805463300	233
**P2RY4**	0.011466	-2.8166	-2.274565595179460	234
**PRORY**	0.011559	-4.2575	-2.2714789145041300	235
**NHLRC3**	0.011593	-5.9418	-2.2703558326760900	236
**TMEM19**	0.011685	-5.5087	-2.267331181414850	237
**TPRG1**	0.011712	-1.9479	-2.266447436067300	238
**NSMCE2**	0.011729	-5.6622	-2.265891910609090	239
**MBOAT7**	0.01175	-6.2045	-2.265206637289430	240
**IL27**	0.011798	-2.2058	-2.263644280733780	242
**PAIP1**	0.011798	-0.012771	-2.263644280733780	241
**LEPROT**	0.011836	-5.5874	-2.2624113220847200	243
**NMRK1**	0.011836	-7.3093	-2.2624113220847200	244
**ORC3**	0.011873	-1.7405	-2.2612141054209000	245
**CD247**	0.011877	-3.165	-2.2610848705422200	246
**AUNIP**	0.012034	-4.0904	-2.256041981165670	247
**MYB**	0.012093	-0.099724	-2.2541616229793600	248
**PTPRF**	0.012167	-6.4564	-2.2518144184910600	249
**ZXDB**	0.012178	-4.8214	-2.251466566750060	250
**ALLC**	0.012389	-4.9465	-2.2448462861185700	251
**EREG**	0.012407	-5.7766	-2.244286055228290	252
**CBR3**	0.01248	-5.1333	-2.242021199527150	253

**DISC1**	0.012529	-3.2578	-2.240507379776090	254
**ABHD12**	0.012545	-5.8275	-2.2400141813825600	255
**ABI2**	0.012579	-5.95	-2.2389679403013400	256
**ELP5**	0.012583	-5.133	-2.2388450141087100	258
**MUC12**	0.012583	-3.4591	-2.2388450141087100	257
**TMED1**	0.012703	-0.743	-2.2351728639663300	259
**HECTD2**	0.012722	-6.1724	-2.2345941954698800	260
**QSER1**	0.012777	-3.8131	-2.232923307553660	261
**CLN5**	0.012832	-4.5037	-2.231258630500360	262
**GZMK**	0.01285	-3.9029	-2.2307151674988300	263
**DNAL4**	0.013051	-5.949	-2.2246907995038300	264
**ASXL1**	0.013082	-3.2618	-2.2237688183024500	266
**CLCF1**	0.013082	-3.0359	-2.2237688183024500	265
**C1QTNF4**	0.013139	-6.2555	-2.2220784795699600	267
**FOXD4L6**	0.013172	-5.883	-2.2211027568079300	268
**CXorf65**	0.013175	-5.1411	-2.221014159517720	269
**PRSS12**	0.013175	-5.7364	-2.221014159517720	270
**TYROBP**	0.013175	-5.9215	-2.221014159517720	271
**FAM114A1**	0.013252	-7.3098	-2.218746106298140	272
**FMN1**	0.013277	-6.3249	-2.2180121735587500	273
**CHFR**	0.013358	-3.0772	-2.215642402809950	274
**SLC25A23**	0.013367	-5.7411	-2.2153798616152600	275
**C4orf48**	0.01337	-1.2887	-2.2152923818072900	276
**LIPM**	0.01337	-3.592	-2.2152923818072900	277
**SNX2**	0.013446	-5.9974	-2.2130818608435700	278
**MOBP**	0.013461	-4.5294	-2.2126468490493000	279
**TMED7-TIC/**	0.013466	-5.4845	-2.2125019381190600	280
**TRIM10**	0.01359	-5.4966	-2.208922923257710	281
**PSMD1**	0.013601	-2.8067	-2.2086067927896200	282
**ATF2**	0.013706	-5.4164	-2.2056002368372300	283
**LOC1027242**	0.013746	-2.6377	-2.2044601075771500	284
**SERF1A**	0.013752	-8.6312	-2.2042893351104500	285

**ID1**	0.013799	-4.5959	-2.2029538364793500	286
**ACSBG1**	0.013848	-5.0639	-2.201565678913500	288
**PLA2G3**	0.013848	-4.8172	-2.201565678913500	287
**TCTN1**	0.013848	-5.3238	-2.201565678913500	289
**CRYL1**	0.013906	-5.4365	-2.19992801744192	290
**WDR78**	0.014006	-8.6198	-2.197118242838470	291
**SUMF1**	0.014013	-5.581	-2.1969222069548800	292
**CCDC178**	0.014064	-0.98694	-2.1954964877048800	293
**ARHGEF3**	0.014159	-4.4902	-2.1928525745311000	294
**PTCHD4**	0.014161	-7.6372	-2.1927970776854900	295
**TAF6L**	0.014192	-4.8903	-2.191937738969990	296
**DLAT**	0.014268	-6.0538	-2.1898377951104000	297
**LIMD2**	0.014269	-5.7128	-2.189810228555880	298
**HGF**	0.014374	-1.2485	-2.1869249577518000	299
**ANO1**	0.014418	-5.1796	-2.1857212854131200	300
**TBX20**	0.014418	-6.2559	-2.1857212854131200	301
**GTPBP10**	0.014502	-5.359	-2.183432120636240	302
**SSX2B**	0.01456	-3.435	-2.1818581588231600	303
**C2orf81**	0.014596	-8.5967	-2.1808839289864300	304
**PRPF8**	0.014675	-5.073	-2.1787532620196600	305
**MYOZ2**	0.014709	-6.1654	-2.1778393024072700	306
**FGFR4**	0.014788	-4.6319	-2.1757226870848000	307
**SLC5A8**	0.014886	-3.0745	-2.1731104851313900	309
**SUGP1**	0.014886	-2.6765	-2.1731104851313900	308
**CNGA2**	0.014946	-5.5416	-2.1715184667672500	311
**HLA-A**	0.014946	-4.6763	-2.1715184667672500	310
**PRKDC**	0.015102	-3.4641	-2.1674047842068800	312
**LENG9**	0.015113	-6.0219	-2.1671160972782500	313
**SORBS3**	0.01514	-3.4545	-2.1664082668155200	314
**RCOR2**	0.01526	-4.3671	-2.1632754150593600	315
**CLDND2**	0.015321	-3.4217	-2.161690986777240	316
**POTEE**	0.015351	-5.5947	-2.1609137462293000	317

**ZG16B**	0.015351	-6.461	-2.1609137462293000	318
**CRY2**	0.015414	-5.4956	-2.1592857773170100	319
**CDK2AP2**	0.015529	-6.1487	-2.156328762467440	321
**KDM4C**	0.015529	-5.2955	-2.156328762467440	320
**MAPKBP1**	0.015548	-3.3718	-2.155842022666230	322
**OR4F4**	0.015622	-8.5654	-2.153951151684260	323
**UCHL5**	0.015639	-6.318	-2.153517848316340	324
**SLC5A9**	0.015706	-0.34509	-2.151814048605200	325
**ENTPD4**	0.015752	-5.1563	-2.15064788209519	326
**TAF11**	0.015777	-1.1343	-2.1500153206266700	327
**SLC2A13**	0.01578	-2.8997	-2.1499394710347300	328
**ACPT**	0.015781	-6.2281	-2.149914190585850	329
**ELAVL4**	0.015787	-5.0727	-2.149762536739430	330
**ELOA3D**	0.015787	-6.4103	-2.149762536739430	331
**MFSD12**	0.016067	-5.7631	-2.142739733105590	332
**LRP2**	0.016078	-4.2367	-2.142465985262180	333
**MTF1**	0.016078	-5.1255	-2.142465985262180	334
**RPA2**	0.016172	-4.9473	-2.140133208486240	335
**BCO2**	0.016235	-4.0477	-2.1385762456169100	336
**FAM47A**	0.016235	-5.4034	-2.1385762456169100	338
**SMAD7**	0.016235	-5.2065	-2.1385762456169100	337
**TNRC6C**	0.01627	-5.4051	-2.1377135013341000	339
**SKI**	0.016301	-3.4751	-2.136950682939220	340
**CSE1L**	0.01632	-7.5023	-2.13648376299072	341
**DHPS**	0.016327	-5.0728	-2.136311857203100	342
**FAM3A**	0.016437	-4.3327	-2.1336187353696300	343
**RDH11**	0.016661	-1.411	-2.12818192178989	344
**SYNE4**	0.016661	-2.5891	-2.12818192178989	345
**PPAN**	0.016682	-5.183	-2.127675433471030	346
**SLC25A35**	0.016708	-3.5367	-2.127049107955940	347
**GNAI2**	0.01678	-5.6181	-2.1253190094293300	348
**OR6C74**	0.016893	-7.8022	-2.122616476138660	349

**KDM1B**	0.016974	-5.3068	-2.120688758218390	350
**KRTCAP2**	0.017072	-5.4009	-2.1183669432337300	351
**OR2M2**	0.017165	-4.8384	-2.116174099135310	352
**OR10A2**	0.017185	-2.359	-2.1157038466563400	353
**ZNF165**	0.017289	-4.144	-2.1132660449745400	354
**HSPBAP1**	0.01733	-7.4013	-2.112308430261030	355
**GUCY1B3**	0.017426	-2.2809	-2.110073757639220	356
**MAU2**	0.017426	-3.5554	-2.110073757639220	357
**RAB6C**	0.01748	-6.1329	-2.108821370197750	358
**TAOK3**	0.017627	-4.61	-2.105428750836040	359
**GATAD1**	0.01764	-1.6046	-2.105129886878850	360
**SULT1C2**	0.017795	-7.49	-2.101580912563280	361
**UBASH3A**	0.017811	-5.2399	-2.101216070287320	362
**MED8**	0.017996	-3.3511	-2.0970177581182200	364
**TMEM81**	0.017996	-1.4189	-2.0970177581182200	363
**DACH2**	0.018046	-7.2116	-2.0958893984304300	365
**UNC80**	0.018077	-4.6861	-2.0951911532626400	366
**POMC**	0.018091	-5.3384	-2.0948761512769900	367
**F8A3**	0.018095	-4.4861	-2.0947861888774300	368
**MAEL**	0.01811	-4.0845	-2.0944489807855700	369
**C5orf67**	0.018308	-2.7365	-2.0900199968694900	370
**CNTN2**	0.018308	-5.8001	-2.0900199968694900	372
**SCGB1D1**	0.018308	-3.5343	-2.0900199968694900	371
**TERF2IP**	0.018384	-3.3142	-2.088330816834340	373
**SLFN14**	0.018449	-4.0258	-2.08689083599862	374
**IFNL4**	0.018456	-3.6595	-2.086736018977760	376
**KCNK1**	0.018456	-0.96892	-2.086736018977760	375
**CD8B**	0.01854	-5.3243	-2.0848821044150500	378
**UBE2D1**	0.01854	-0.96318	-2.0848821044150500	377
**MC5R**	0.018662	-4.1388	-2.082202210128700	379
**JMJD1C**	0.018758	-5.6411	-2.080103904979460	380
**GIMAP4**	0.018765	-1.5728	-2.079951261326370	381

**LOC1001328**	0.018765	-4.2148	-2.079951261326370	382
**MRPL37**	0.018791	-6.1591	-2.07938472307907	383
**MED14**	0.018835	-5.8586	-2.0784274837707300	384
**BRWD1**	0.018889	-5.7179	-2.0772552869540600	386
**MRPS10**	0.018889	-3.4426	-2.0772552869540600	385
**SP2**	0.018988	-2.7297	-2.075113643453570	387
**COL13A1**	0.019006	-6.7892	-2.074725274523970	388
**KIN**	0.019036	-4.0913	-2.074078687541370	389
**PRR23A**	0.019057	-5.4079	-2.073626592053130	390
**LILRA5**	0.019073	-5.9296	-2.0732824226035300	391
**C6orf52**	0.019096	-6.4775	-2.0727881088679300	392
**GPR143**	0.019127	-4.9698	-2.072122660252370	393
**PHF21A**	0.019145	-3.5967	-2.0717366914099700	394
**KRT10**	0.019205	-3.9391	-2.0704523527358700	395
**TNPO2**	0.019209	-6.8775	-2.070366851490740	396
**CELSR2**	0.019276	-1.1695	-2.0689369505430700	397
**MRPS36**	0.019277	-3.7532	-2.068915640752330	398
**TMEM245**	0.019284	-5.6744	-2.0687664985170700	399
**KIFC1**	0.01933	-4.6365	-2.0677875639318800	401
**SEMA3E**	0.01933	-4.1741	-2.0677875639318800	400
**CACNB1**	0.019475	-4.114	-2.064714691849190	402
**SMR3A**	0.019496	-6.0466	-2.0642712677442800	403
**GPC6**	0.019497	-7.1419	-2.064250162432190	404
**VSIG10**	0.019587	-0.76065	-2.062354438644380	405
**AMY2B**	0.019785	-6.0046	-2.0582097436896900	406
**UGT2B7**	0.019815	-7.1899	-2.057584834357980	408
**VPS13A**	0.019815	-7.1792	-2.057584834357980	407
**TMEM39A**	0.019881	-3.4525	-2.0562128553167100	409
**FRAS1**	0.019937	-4.7532	-2.055051779554030	410
**HIST1H3E**	0.019947	-3.6006	-2.0548447358560900	413
**POLR2B**	0.019947	-1.239	-2.0548447358560900	411
**RNF121**	0.019947	-2.3298	-2.0548447358560900	412

**GPX6**	0.020066	-5.4225	-2.052387650696220	414
**POLR2H**	0.020114	-2.5477	-2.0514000547182500	415
**STOM**	0.020175	-3.7994	-2.0501478648594500	416
**ZNF547**	0.020178	-4.8713	-2.0500863646246400	417
**MRVI1**	0.020248	-2.8794	-2.0486535555312400	418
**GSX2**	0.020263	-6.5491	-2.04834707152627	420
**MYH7**	0.020263	-5.046	-2.04834707152627	419
**HYOU1**	0.020382	-4.6822	-2.045922423472390	421
**RAB1B**	0.020446	-6.7711	-2.044623368830280	422
**THAP6**	0.020458	-4.4305	-2.0443801798092100	424
**VPS16**	0.020458	-2.9047	-2.0443801798092100	423
**C7orf26**	0.020488	-5.3414	-2.04377273566572	425
**CDK5R2**	0.020636	-4.0584	-2.0407869946842100	426
**THAP10**	0.02065	-3.8738	-2.04050549974147	427
**VWA7**	0.020721	-4.9862	-2.0390804017447000	428
**SSTR2**	0.020784	-7.3513	-2.0378193365179200	429
**EGLN2**	0.020793	-3.8803	-2.0376394486464300	430
**PCDHGA10**	0.020836	-2.7819	-2.0367808931962300	432
**TCHH**	0.020836	-1.6145	-2.0367808931962300	431
**TRAPPC10**	0.020877	-4.525	-2.0359636664279100	433
**WISP2**	0.021015	-5.1123	-2.0332229455175100	434
**DCD**	0.021096	-3.6886	-2.0316213472644300	435
**LCT**	0.021113	-4.9259	-2.031285870123940	436
**GOLGA8N**	0.02115	-8.3929	-2.0305565032343100	437
**DTD2**	0.021176	-5.5401	-2.03004462063713	438
**TM7SF2**	0.021287	-5.0852	-2.0278652373008200	439
**PCDHB8**	0.021288	-6.6408	-2.0278456469438700	440
**OR2W3**	0.021299	-5.3506	-2.0276302043647000	444
**PARP14**	0.021299	-5.1437	-2.0276302043647000	443
**SIRPA**	0.021299	-3.3209	-2.0276302043647000	442
**STK36**	0.021299	-3.2867	-2.0276302043647000	441
**OR56A3**	0.021406	-3.8091	-2.025539429793960	445

**CST7**	0.021501	-4.4936	-2.0236905264417400	446
**SEPT14**	0.021551	-7.4423	-2.0227201913662500	447
**GHR**	0.021611	-5.1605	-2.0215582975484800	448
**SKAP2**	0.021724	-0.41837	-2.019377445295750	449
**UHRF1BP1L**	0.021736	-5.8712	-2.0191464136684400	450
**NFU1**	0.021883	-5.8124	-2.016324984462320	452
**ZNF778**	0.021883	-4.5897	-2.016324984462320	451
**PRSS8**	0.022072	-4.6062	-2.0127208560113700	453
**APPBP2**	0.022089	-3.112	-2.0123979537727100	455
**MAP2**	0.022089	-2.939	-2.0123979537727100	454
**CITED4**	0.022092	-5.8936	-2.012340992800380	456
**TMEM229B**	0.0222	-5.1131	-2.01029473269187	457
**METTL3**	0.022204	-4.384	-2.0102191067476300	458
**SERF1B**	0.022239	-2.743	-2.009557869648270	459
**KCNK13**	0.022363	-4.8265	-2.0072222446807600	460
**SFT2D3**	0.022375	-1.6468	-2.0069967966756500	461
**SASS6**	0.022453	-3.0348	-2.005533865370920	462
**MCCC1**	0.022529	-2.9634	-2.004112561791430	463
**RBM7**	0.022577	-4.8459	-2.003216978021070	464
**TMEM31**	0.022577	-5.7593	-2.003216978021070	465
**MAP3K7**	0.022637	-4.1405	-2.0020997529581200	466
**ZNF502**	0.022678	-4.54	-2.001337750659860	467
**LMBR1**	0.022751	-7.1255	-1.999983884416880	469
**NOSTRIN**	0.022751	-6.6493	-1.999983884416880	468
**ALDH7A1**	0.022879	-5.0433	-1.9976187939354900	471
**SEZ6L2**	0.022879	-4.8145	-1.9976187939354900	470
**H1FNT**	0.022883	-2.7035	-1.997545064675280	472
**TMEM220**	0.022926	-4.0904	-1.9967531600818600	473
**PREB**	0.023069	-7.0438	-1.994128584426810	474
**PSMB8**	0.02307	-4.2329	-1.9941102790515200	475
**DGKI**	0.023221	-5.1818	-1.991353804006310	476
**VNN 3.00**	0.023231	-4.7625	-1.9911717893077600	477

**CHRM1**	0.023234	-1.2316	-1.9911171977594000	478
**GCG**	0.023234	-3.1422	-1.9911171977594000	479
**SEC24B**	0.023234	-3.3413	-1.9911171977594000	480
**SSBP2**	0.023404	-5.2673	-1.9880333273289800	481
**RSPH6A**	0.023428	-5.4304	-1.987599477080950	482
**STAMBP**	0.023465	-6.8715	-1.9869313566999000	483
**CLMP**	0.023485	-7.4618	-1.9865705795140300	484
**DNAJC28**	0.023516	-2.7149	-1.986011885362760	485
**BBS10**	0.023721	-3.1602	-1.9823328095347800	486
**HSP90AB1**	0.023865	-4.6091	-1.9797644365206200	487
**YAF2**	0.024041	-5.3201	-1.9766429446760300	488
**KRTAP9-1**	0.02418	-1.2992	-1.9741912172769700	489
**C14orf166**	0.024263	-7.3568	-1.972732875700070	490
**C9orf3**	0.024346	-5.1967	-1.97127871762314	492
**POFUT1**	0.024346	-3.99	-1.97127871762314	491
**ESRRB**	0.024552	-1.2258	-1.9676875054731800	493
**CTRB1**	0.024647	-5.8667	-1.9660398781552800	494
**FUT10**	0.024728	-5.323	-1.9646392622966600	497
**INTS2**	0.024728	-3.8865	-1.9646392622966600	495
**NIPAL1**	0.024728	-4.9721	-1.9646392622966600	496
**IQUB**	0.024776	-4.3477	-1.9638110829135400	499
**LAMA2**	0.024776	-3.3008	-1.9638110829135400	498
**EIF4H**	0.024789	-5.8963	-1.9635870159585700	500
**POMT2**	0.024928	-3.534	-1.9611973630915100	501
**AJAP1**	0.025031	-3.4883	-1.959433810094330	502
**FUZ**	0.025035	-6.981	-1.9593654454091300	504
**TRNP1**	0.025035	-6.4966	-1.9593654454091300	503
**RAD54B**	0.025066	-3.7822	-1.9588359293440700	505
**GRAP**	0.025216	-2.784	-1.9562814820694800	506
**IL1RL2**	0.025277	-4.4127	-1.9552463145663000	507
**MANBA**	0.02531	-4.1509	-1.9546871780858600	508
**PYCR3**	0.02531	-5.7999	-1.9546871780858600	509

**IFI44L**	0.025404	-6.4953	-1.953097827108980	510
**TRIM49B**	0.025404	-7.4859	-1.953097827108980	511
**CYP4V2**	0.02548	-4.4161	-1.9518164173384400	512
**NAA10**	0.02549	-3.2753	-1.9516480491761800	515
**PHYHIPL**	0.02549	-2.7753	-1.9516480491761800	514
**PRH2**	0.02549	-2.541	-1.9516480491761800	513
**UCN**	0.02549	-3.6136	-1.9516480491761800	516
**MLNR**	0.025589	-3.5754	-1.9499841818883600	517
**YIPF7**	0.025594	-5.1427	-1.9499002912645600	518
**ZSWIM2**	0.025781	-4.9793	-1.9467725902987100	519
**CCNB1IP1**	0.025782	-6.6919	-1.9467559157403600	520
**DBT**	0.025782	-7.326	-1.9467559157403600	521
**CCDC89**	0.025885	-5.8778	-1.9450413279988200	524
**NCS1**	0.025885	-5.5218	-1.9450413279988200	523
**SLC25A25**	0.025885	-5.3876	-1.9450413279988200	522
**CCDC177**	0.025924	-5.9755	-1.9443936049664400	525
**RNF41**	0.026028	-5.0825	-1.9426703204074600	526
**BICD1**	0.026069	-5.0299	-1.9419925312430400	527
**AAED1**	0.026151	-6.7948	-1.9406396231918000	529
**BTNL10**	0.026151	-6.3872	-1.9406396231918000	530
**SAP30**	0.026151	-5.0951	-1.9406396231918000	528
**KHSRP**	0.026152	-2.7985	-1.9406231462216100	531
**RPL41**	0.026274	-5.3259	-1.9386168972587200	533
**ZNF77**	0.026274	-2.7628	-1.9386168972587200	532
**AMBN**	0.02629	-5.0566	-1.9383543606445700	534
**MTTP**	0.02635	-4.9092	-1.9373710363745200	535
**OR5J2**	0.026367	-3.9872	-1.9370927681096900	536
**ARHGAP15**	0.026436	-3.2693	-1.9359648635042000	537
**MEIG1**	0.026436	-5.7766	-1.9359648635042000	539
**RLN3**	0.026436	-3.7318	-1.9359648635042000	538
**UBD**	0.026859	-5.3136	-1.9291035810754100	540
**KRT27**	0.026867	-1.4455	-1.9289746889133500	541

**PSG1**	0.026867	-1.9807	-1.9289746889133500	542
**PDCD2L**	0.026876	-5.8031	-1.9288297235261000	543
**NHS**	0.026946	-3.6517	-1.9277035962639700	544
**METTL27**	0.026986	-5.6163	-1.9270611909580300	545
**LOC105377**	0.027055	-4.1099	-1.9259549073601400	546
**RRP7A**	0.027165	-2.0645	-1.9241961259675500	547
**RPS3**	0.02723	-6.9565	-1.9231596372851600	548
**GKN2**	0.027358	-4.4214	-1.9211245713447700	549
**MAT2B**	0.027375	-0.97414	-1.9208548868677400	550
**SLC25A38**	0.027478	-4.5184	-1.9192238961865200	551
**METTL17**	0.02753	-5.8442	-1.9184024189925600	552
**EIF5A**	0.027598	-4.2214	-1.917330129167070	553
**CELA2B**	0.0276	-3.0907	-1.9172986245863300	554
**MOGAT1**	0.0276	-3.1316	-1.9172986245863300	555
**FCGR2C**	0.027601	-3.8058	-1.9172828730095600	556
**CPB2**	0.027729	-4.2438	-1.9152705870851500	557
**CTSE**	0.027758	-1.4631	-1.9148157544374900	558
**CDCA8**	0.027839	-5.6191	-1.9135474538711400	559
**AKIP1**	0.02785	-3.6161	-1.9133754527148400	560
**VWDE**	0.027884	-4.3704	-1.9128441703544600	561
**IFNE**	0.027894	-3.4328	-1.9126880135413500	562
**WARS**	0.027908	-5.5474	-1.9124694723290800	563
**FGF4**	0.028052	-5.1006	-1.9102269035731500	564
**C6orf10**	0.028102	-3.3626	-1.9094504759639300	566
**NLRP8**	0.028102	-2.8654	-1.9094504759639300	565
**PPA2**	0.02823	-5.0365	-1.907468049676050	567
**OR14A16**	0.028458	-5.0116	-1.9039553107254100	568
**DDX60**	0.028469	-1.7152	-1.9037864294469100	569
**HSD17B11**	0.028469	-1.8044	-1.9037864294469100	570
**ENG**	0.028476	-4.111	-1.903678987806530	571
**CYHR1**	0.028499	-6.7321	-1.9033261199129600	572
**PCID2**	0.028528	-4.7602	-1.9028815370489200	573

**KRTAP9-7**	0.028611	-0.063406	-1.9016111846771900	574
**C3orf79**	0.028775	-4.3948	-1.8991100755403800	575
**MRGPRD**	0.028852	-3.8184	-1.8979398610245600	576
**PCDHGA8**	0.028875	-4.7351	-1.8975908200212700	577
**SPTLC2**	0.028921	-2.8748	-1.8968934307561300	578
**SLC6A20**	0.028928	-5.2526	-1.896787387141040	579
**ERMARD**	0.028985	-4.2567	-1.8959246821008500	581
**NDNF**	0.028985	-3.0784	-1.8959246821008500	580
**ZNF720**	0.028999	-4.5633	-1.895713005334380	582
**ARMCX5**	0.029102	-3.7867	-1.894158274329730	583
**F8**	0.029279	-4.2522	-1.8914971980285100	584
**TUBGCP6**	0.029279	-5.1068	-1.8914971980285100	585
**STAT3**	0.029296	-7.1967	-1.8912423183277600	587
**XKRY2**	0.029296	-6.563	-1.8912423183277600	586
**RAB22A**	0.029426	-0.49607	-1.8892972885552100	588
**AS3MT**	0.029475	-4.1013	-1.8885660131175500	589
**PRSS42**	0.029539	-5.4731	-1.8876123962722900	590
**PCP2**	0.029674	-5.7045	-1.8856064714745500	591
**ARL 2.00**	0.029684	-5.8876	-1.8854581859369300	595
**C16orf54**	0.029684	-5.3318	-1.8854581859369300	593
**C1QTNF9**	0.029684	-4.4587	-1.8854581859369300	592
**XYLT2**	0.029684	-5.5507	-1.8854581859369300	594
**APLF**	0.02982	-6.7495	-1.883445606499340	596
**LGR4**	0.029902	-5.5098	-1.8822358163243200	597
**LAT**	0.030101	-3.9947	-1.8793112630434800	598
**CATSPER3**	0.030161	-3.2885	-1.8784326338760900	599
**PRAMEF1**	0.030277	-5.2287	-1.8767380511741000	600
**IGHMBP2**	0.030387	-3.7358	-1.8751360824845300	601
**CCDC102B**	0.030391	-0.54435	-1.875077919680750	602
**H2AFB1**	0.030452	-2.0452	-1.8741917218738600	603
**CSRP1**	0.030551	-4.5043	-1.8727565916266000	604
**CCDC171**	0.030555	-5.2162	-1.8726986875776300	605

**RSC1A1**	0.030614	-5.4565	-1.8718453312866200	606
**C19orf53**	0.03074	-2.1068	-1.8700274597587000	607
**SLC17A9**	0.030766	-3.6306	-1.8696531115458300	608
**PWWP2A**	0.030789	-5.4776	-1.8693221756218600	609
**MBIP**	0.030792	-0.86453	-1.8692790251566000	610
**WI2-2373I1.**	0.030792	-1.0533	-1.8692790251566000	611
**ADRM1**	0.030953	-5.4729	-1.8669683721938900	612
**CHP2**	0.031011	-4.2391	-1.8661383999545700	614
**SEC63**	0.031011	-3.7392	-1.8661383999545700	613
**BTG1**	0.03102	-3.442	-1.8660097263085700	615
**CASP12**	0.031044	-4.1704	-1.8656667475476000	616
**TAAR6**	0.031111	-5.2624	-1.864710424952890	617
**SERPINB2**	0.031146	-7.3451	-1.8642115308040200	618
**CD40**	0.031221	-3.5623	-1.8631440316274500	619
**C10orf131**	0.031292	-3.4433	-1.8621354183377100	621
**SNX24**	0.031292	-2.1056	-1.8621354183377100	620
**RARS2**	0.031307	-5.3234	-1.861922573225640	622
**OTUD5**	0.031335	-5.5539	-1.861525487886510	623
**CP**	0.031433	-3.7558	-1.8601379957100500	624
**ATAT1**	0.031617	-6.4654	-1.857542540550220	625
**PAPLN**	0.03172	-5.2756	-1.8560950953156000	626
**ATN1**	0.031723	-3.6344	-1.85605299495706	628
**CRIP2**	0.031723	-2.0538	-1.85605299495706	627
**DHX37**	0.031736	-4.7267	-1.8558705980743400	629
**TDRKH**	0.031764	-5.4293	-1.8554779528211300	630
**MTHFR**	0.031858	-0.008906	-1.8541618745328000	631
**GSDMA**	0.031867	-3.42	-1.8540360353732100	632
**C15orf32**	0.032027	-5.3228	-1.8518037795381900	633
**C14orf28**	0.032157	-3.0166	-1.849996842157100	636
**NIPBL**	0.032157	-2.0076	-1.849996842157100	634
**REEP4**	0.032157	-5.5012	-1.849996842157100	637
**SLC19A2**	0.032157	-2.3045	-1.849996842157100	635

**NEGR1**	0.032163	-5.7521	-1.849913590739650	638
**RASA4**	0.032249	-3.4561	-1.8487217269274300	639
**MPND**	0.032313	-3.8789	-1.8478364598848700	640
**ACTN3**	0.032408	-4.4124	-1.8465250558444400	641
**CMPK1**	0.032428	-4.7653	-1.8462493750445800	643
**CRYBA2**	0.032428	-4.9554	-1.8462493750445800	644
**FCRLA**	0.032428	-5.0565	-1.8462493750445800	645
**RGSL1**	0.032428	-4.2249	-1.8462493750445800	642
**SEPT2**	0.032576	-2.1417	-1.8442136850759500	646
**C9orf85**	0.03278	-8.0743	-1.8414202009414600	647
**HLA-DPA1**	0.032802	-5.2291	-1.8411197998411300	648
**CFB**	0.032907	-4.2293	-1.8396883511835400	649
**WSCD2**	0.033021	-4.6599	-1.838138463105470	650
**EIF4ENIF1**	0.033116	-5.8089	-1.8368502542919100	652
**PTPN4**	0.033116	-4.9841	-1.8368502542919100	651
**GALNT12**	0.033141	-4.6996	-1.8365117580798800	653
**C17orf112**	0.033395	-2.2469	-1.8330845071839100	655
**FDX1**	0.033395	-3.296	-1.8330845071839100	657
**HLA-DQA2**	0.033395	-3.5513	-1.8330845071839100	658
**SMARCAD1**	0.033395	-1.1586	-1.8330845071839100	654
**UBASH3B**	0.033395	-2.4631	-1.8330845071839100	656
**DMRT1**	0.033472	-5.1214	-1.8320497780037100	659
**CD1C**	0.033545	-6.2184	-1.831070609011360	660
**ANXA13**	0.033577	-4.7585	-1.8306419371400900	662
**HGSNAT**	0.033577	-3.8074	-1.8306419371400900	661
**HMGCS2**	0.033687	-5.4825	-1.829170937516670	663
**PHACTR4**	0.033796	-6.1778	-1.827717204441740	664
**SYNGAP1**	0.033822	-3.8158	-1.8273710123549500	665
**DPY19L3**	0.033915	-4.2933	-1.8261344992515800	667
**MED22**	0.033915	-3.3923	-1.8261344992515800	666
**GPR17**	0.03394	-4.3753	-1.8258025789478700	668
**SSX4**	0.034003	-6.8105	-1.824967030757230	669

**CYP2A6**	0.034014	-3.8341	-1.8248212719681200	670
**ISCA2**	0.034014	-4.5264	-1.8248212719681200	671
**COPA**	0.034076	-4.8958	-1.82400044663761	672
**SNTB2**	0.034182	-6.0695	-1.8225999401535700	673
**CACNA1D**	0.034411	-3.3388	-1.8195864630551700	674
**OR14C36**	0.034411	-5.8537	-1.8195864630551700	676
**PRB3**	0.034411	-4.7005	-1.8195864630551700	675
**CABLES1**	0.034597	-4.7633	-1.817150939582330	679
**EPHA10**	0.034597	-1.7925	-1.817150939582330	677
**LRRC2**	0.034597	-3.7068	-1.817150939582330	678
**THOC5**	0.034597	-4.7811	-1.817150939582330	680
**OR10R2**	0.034846	-4.6876	-1.8139072608829200	681
**ERVH48-1**	0.034953	-3.6417	-1.8125192342771400	682
**TSPAN16**	0.034974	-3.3023	-1.8122472273765300	683
**PPP2R5B**	0.035056	-3.7295	-1.8111863871366100	684
**CEP55**	0.035163	-3.0193	-1.8098051778912100	685
**POLR3G**	0.035163	-5.3666	-1.8098051778912100	686
**PARP3**	0.03519	-5.4565	-1.8094571934099400	687
**ETV7**	0.035234	-3.4322	-1.8088905766353800	688
**DUT**	0.035262	-4.0797	-1.8085303044765800	689
**LFNG**	0.035279	-0.94938	-1.8083116822823000	690
**MS4A8**	0.035279	-4.847	-1.8083116822823000	691
**SH3BP5**	0.035379	-3.3232	-1.8070274156188700	692
**KCNQ5**	0.03558	-8	-1.8044550185095200	693
**B3GNTL1**	0.035716	-5.0536	-1.802721240395600	697
**DPF1**	0.035716	-0.27072	-1.802721240395600	694
**DYRK2**	0.035716	-0.78266	-1.802721240395600	695
**OR51E2**	0.035716	-2.7642	-1.802721240395600	696
**HIST1H2BJ**	0.035751	-7.9941	-1.8022759230678000	698
**PTPRZ1**	0.035771	-4.0192	-1.8020216164046000	699
**GATA6**	0.035823	-3.6059	-1.8013609639312900	700
**ZNF57**	0.035861	-5.1068	-1.8008786761689900	701

**GDF5**	0.035956	-6.9232	-1.7996747859732700	702
**SLC24A5**	0.035957	-3.2938	-1.7996621273104600	703
**THY1**	0.036017	-3.7335	-1.7989031347217300	704
**ATP6AP2**	0.036162	-2.5069	-1.797073168773140	705
**ORMDL1**	0.036162	-3.0467	-1.797073168773140	706
**ZNF705D**	0.036216	-7.9823	-1.7963931995200700	707
**GMIP**	0.036469	-6.2695	-1.7932184277694000	708
**SEL1L3**	0.036469	-7.1713	-1.7932184277694000	709
**C2orf48**	0.036507	-4.8772	-1.792743142735640	712
**HAO1**	0.036507	-4.8667	-1.792743142735640	711
**MYADM**	0.036507	-4.6575	-1.792743142735640	710
**DAB2IP**	0.036613	-3.2735	-1.791419483727050	715
**TNS1**	0.036613	-3.2545	-1.791419483727050	714
**YKT6**	0.036613	-2.7356	-1.791419483727050	713
**EXOC8**	0.036737	-3.3358	-1.7898750252181000	716
**STOML2**	0.036769	-5.3373	-1.7894771475299600	717
**NDUFAF1**	0.036951	-4.4148	-1.7872195879694400	718
**KLHL34**	0.036979	-4.6436	-1.7868730784460000	719
**OR12D2**	0.036979	-5.5203	-1.7868730784460000	720
**C1orf226**	0.037015	-0.8785	-1.7864278812175800	721
**NEDD1**	0.037018	-3.5462	-1.786390797427200	722
**SH2D2A**	0.037129	-3.1113	-1.785020420930840	723
**DNAJC7**	0.0373	-4.9085	-1.7829158375651300	724
**SP3**	0.037374	-5.0762	-1.7820075251907200	725
**OR5T1**	0.037442	-2.1973	-1.7811741540377700	726
**CHD4**	0.037451	-5.5007	-1.7810639475668100	728
**INHBA**	0.037451	-5.3892	-1.7810639475668100	727
**GFRAL**	0.037497	-1.3267	-1.7805010076030200	729
**CRYBA4**	0.037512	-4.1462	-1.7803175621598500	730
**FADS3**	0.037565	-6.3116	-1.7796698676239900	731
**AGTR2**	0.037601	-0.72492	-1.779230349582200	732
**TMEM70**	0.037653	-4.1085	-1.7785960963396100	733

**ORC1**	0.037656	-7.9523	-1.7785595266294900	734
**FKBP8**	0.03773	-3.0652	-1.7776582257794600	735
**RBL2**	0.03773	-3.4361	-1.7776582257794600	736
**JUNB**	0.037967	-5.2953	-1.7747813038579100	737
**FBLIM1**	0.03808	-7.9401	-1.773414762778900	738
**ABHD10**	0.038093	-3.7495	-1.773257762339540	739
**VEGFB**	0.038234	-3.3876	-1.7715577127081700	740
**LAMP2**	0.038262	-4.421	-1.771220722835660	741
**ERP27**	0.038483	-3.5089	-1.7685679425127700	742
**FOXO4**	0.038483	-5.3284	-1.7685679425127700	745
**OR4B1**	0.038483	-4.4091	-1.7685679425127700	743
**PHIP**	0.038483	-5.3455	-1.7685679425127700	746
**SLPI**	0.038483	-4.8481	-1.7685679425127700	744
**KDF1**	0.038596	-3.8681	-1.767216338312050	747
**ANKRD45**	0.038663	-3.4942	-1.7664164667572800	748
**ALG1**	0.038892	-4.7337	-1.7636910755725900	749
**BCL2L1**	0.03893	-4.7484	-1.7632400923815500	750
**RNF138**	0.039083	-4.1767	-1.7614279107986700	751
**CCNB2**	0.039094	-6.7564	-1.761297845924430	754
**LGALS9B**	0.039094	-7.0794	-1.761297845924430	755
**MLKL**	0.039094	-6.6601	-1.761297845924430	753
**TMEM186**	0.039094	-6.1637	-1.761297845924430	752
**CSF3**	0.039129	-5.3438	-1.7608842012480800	756
**ST13**	0.039205	-3.02	-1.7599870373648300	757
**TNP1**	0.039205	-3.273	-1.7599870373648300	758
**EIF5AL1**	0.039281	-5.213	-1.7590912878681600	759
**AP1G1**	0.039477	-3.4516	-1.7567876880663300	760
**CEACAM21**	0.039486	-0.84028	-1.7566821341539100	761
**ITFG1**	0.03954	-5.3569	-1.7560492213267500	762
**FEM1B**	0.039604	-6.016	-1.755300012161140	763
**TCERG1L**	0.039612	-1.6873	-1.7552064302632800	764
**DEFB104B**	0.039624	-2.6478	-1.7550660862312200	765

**GABPB2**	0.039745	-4.3667	-1.7536528783979400	766
**COG7**	0.039767	-4.3217	-1.753396307460560	767
**NKIRAS1**	0.039767	-5.4954	-1.753396307460560	769
**PLS1**	0.039767	-4.8485	-1.753396307460560	768
**KCTD2**	0.040014	-4.8908	-1.7505236065257800	770
**PRR33**	0.040048	-3.5938	-1.750129303250380	771
**ZNF124**	0.040102	-0.94108	-1.7495036155780400	772
**C4BPA**	0.040178	-5.7905	-1.74862417674279	773
**MMP15**	0.040178	-5.9035	-1.74862417674279	774
**ZNF277**	0.0402	-3.7428	-1.7483698545748800	775
**LACC1**	0.040298	-7.8904	-1.7472383365068900	776
**EBPL**	0.040306	-4.5655	-1.7471460663906900	777
**TMEM91**	0.04038	-2.8704	-1.746293272134730	778
**PCSK1**	0.040385	-2.6456	-1.7462356966870000	779
**TCOF1**	0.040385	-3.9759	-1.7462356966870000	780
**OTUD4**	0.04041	-4.026	-1.7459479062424000	781
**CACNG2**	0.040423	-5.377	-1.7457983123411900	782
**BAK1**	0.040511	-2.0795	-1.7447867025636700	783
**DYRK1B**	0.040597	-4.9926	-1.743799806058440	784
**HLA-DRB3**	0.040597	-5.5683	-1.743799806058440	785
**DFNB59**	0.040641	-1.3	-1.7432955383277700	786
**RXRA**	0.040641	-2.0363	-1.7432955383277700	787
**BCHE**	0.040862	-1.5125	-1.7407694198058400	788
**HEYL**	0.040932	-4.6521	-1.7399716034588800	789
**RAB3D**	0.041011	-1.301	-1.7390725389536300	790
**IFNA7**	0.041117	-5.5844	-1.7378684033553000	791
**ADD1**	0.041148	-2.8349	-1.7375167262641600	792
**PREP**	0.041148	-5.3881	-1.7375167262641600	793
**PTRH1**	0.041212	-0.96119	-1.7367913622578900	794
**TAF6**	0.041212	-2.1024	-1.7367913622578900	795
**CYP2C18**	0.041341	-6.2476	-1.7353320714508400	796
**MCTS1**	0.041457	-4.5533	-1.7340229892061100	797

**LCE1E**	0.041567	-2.85	-1.7327843571569400	799
**TMEM82**	0.041567	-0.93163	-1.7327843571569400	798
**USP5**	0.041663	-1.4657	-1.7317055374577700	800
**C8orf74**	0.041688	-4.9533	-1.7314249253345300	802
**MASP2**	0.041688	-2.779	-1.7314249253345300	801
**RESP18**	0.041695	-5.1203	-1.7313463783665600	803
**ZNF638**	0.041773	-3.1009	-1.7304718625049600	804
**GPR160**	0.041975	-1.4774	-1.7282132215481500	805
**OR8B2**	0.042121	-1.9504	-1.7265862107105300	806
**ERCC2**	0.042151	-3.8551	-1.726252458875620	807
**SPCS1**	0.042229	-4.171	-1.7253856028493000	808
**MAPKAPK2**	0.04246	-3.8684	-1.7228259523297900	809
**APOL1**	0.042545	-3.8378	-1.721886924132630	810
**MRPS30**	0.042549	-6.3409	-1.721842771961080	811
**SLC51B**	0.042549	-6.7031	-1.721842771961080	812
**CCND1**	0.042559	-3.7937	-1.7217324062148600	814
**DYDC1**	0.042559	-2.1141	-1.7217324062148600	813
**SLC35E1**	0.042559	-4.1082	-1.7217324062148600	815
**SYDE1**	0.042559	-4.7921	-1.7217324062148600	816
**ZFY**	0.04266	-5.8201	-1.720618885944760	817
**IL26**	0.042686	-3.4238	-1.7203325821749300	818
**RANBP6**	0.042686	-5.0656	-1.7203325821749300	819
**MEST**	0.042808	-2.4929	-1.7189910364702200	820
**SLC27A5**	0.042918	-0.019668	-1.717784092704790	821
**WDR91**	0.042993	-4.6302	-1.7169626089661000	822
**PARP12**	0.04301	-5.4337	-1.716776566975730	823
**LTBP1**	0.043168	-4.1703	-1.7150503065867000	824
**C1QTNF12**	0.043189	-3.5424	-1.7148212513309100	825
**MB21D2**	0.043234	-2.9712	-1.7143307213765100	826
**RD3**	0.04334	-2.7766	-1.7131768782500400	827
**DDHD1**	0.043456	-1.5156	-1.7119167902215300	828
**LYRM7**	0.043456	-3.9357	-1.7119167902215300	829

**MMP11**	0.043493	-4.0078	-1.7115154367980900	830
**SLC25A48**	0.043561	-4.573	-1.7107785324260400	831
**FAM168A**	0.043658	-6.4406	-1.7097289650871500	832
**YIPF4**	0.043667	-2.087	-1.7096316779828900	833
**CCDC25**	0.043767	-3.6381	-1.7085517972913400	834
**C9orf72**	0.043861	-4.3466	-1.7075385229371200	836
**RPL22L1**	0.043861	-3.953	-1.7075385229371200	835
**AP3M2**	0.043922	-4.508	-1.7068819093771300	837
**EFNB1**	0.044152	-1.5975	-1.7044127483810200	838
**TBC1D2B**	0.044207	-4.4087	-1.7038238337719600	839
**CHST11**	0.044244	-5.5525	-1.703427987083620	841
**KRTDAP**	0.044244	-4.1802	-1.703427987083620	840
**TTL**	0.044246	-5.1004	-1.7034065975679400	842
**BATF3**	0.044348	-2.0161	-1.7023167641510300	843
**ZNF407**	0.044404	-3.4344	-1.7017192829610800	844
**USP17L3**	0.044422	-1.7761	-1.7015273643875400	845
**FGG**	0.04449	-5.2162	-1.7008029036750400	846
**AXL**	0.044525	-5.4755	-1.7004303671884900	847
**C6orf99**	0.044558	-5.3689	-1.7000793345195300	848
**LY86**	0.044568	-3.5855	-1.6999730023317300	849
**OR6C70**	0.044596	-5.4729	-1.6996753744160900	850
**PLXNA3**	0.044715	-4.2424	-1.6984121324713500	851
**SEC22A**	0.044741	-3.8541	-1.698136490501740	852
**CNIH2**	0.044751	-2.9778	-1.6980305087061900	853
**KCNH7**	0.044751	-4.6837	-1.6980305087061900	855
**MYCL**	0.044751	-4.3987	-1.6980305087061900	854
**CAPN5**	0.044914	-3.3251	-1.696305688309960	857
**IFT27**	0.044914	-0.78927	-1.696305688309960	856
**DNAL1**	0.045028	-6.7804	-1.6951023644765000	858
**AASS**	0.045044	-3.4009	-1.694933673209460	859
**DNAJC13**	0.04509	-4.3779	-1.6944489542963600	860
**KIAA1549L**	0.045136	-4.6329	-1.6939646331715900	861

**ZNHIT2**	0.045198	-4.6434	-1.6933124805662800	862
**RALGPS1**	0.045314	-5.276	-1.6920942546896300	864
**STS**	0.045314	-5.1983	-1.6920942546896300	863
**SH3BP5L**	0.045366	-5.6281	-1.69154896764914	865
**ZC2HC1B**	0.045377	-3.9199	-1.6914336828960500	866
**WFDC10A**	0.045415	-1.4808	-1.691035599344760	868
**ZNF80**	0.045415	-0.22856	-1.691035599344760	867
**OR14I1**	0.045428	-2.6522	-1.6908994743668100	869
**ZSCAN10**	0.045437	-4.6688	-1.6908052523487400	870
**COL15A1**	0.04549	-6.7945	-1.690250693593950	873
**LHFPL1**	0.04549	-6.1346	-1.690250693593950	871
**TAAR1**	0.04549	-6.4	-1.690250693593950	872
**BICRA**	0.045619	-4.9189	-1.6889030865279200	874
**PACS1**	0.045694	-0.088595	-1.6881210018763600	875
**BCL2L13**	0.045698	-1.8777	-1.6880793196882500	876
**PTPN14**	0.045915	-3.0001	-1.685822443189910	877
**RPE**	0.04593	-3.6983	-1.6856667548666100	878
**RAB3B**	0.045938	-2.0323	-1.6855837377980500	879
**DESI1**	0.046086	-7.7648	-1.6840500130980800	880
**CHMP4C**	0.046089	-4.7587	-1.684018965022660	882
**CLEC18B**	0.046089	-4.7564	-1.684018965022660	881
**RAB7A**	0.046089	-4.8136	-1.684018965022660	883
**TUBGCP5**	0.04612	-3.6523	-1.683698229925740	884
**LYPD6B**	0.046206	-3.0149	-1.6828093542714000	885
**OR4D10**	0.046244	-4.8902	-1.6824170184464800	886
**CEP104**	0.046255	-5.9424	-1.6823034958684000	887
**KCNIP3**	0.046256	-1.8888	-1.6822931767090300	888
**ISCU**	0.046264	-2.7459	-1.6822106298824000	889
**SPRY2**	0.046264	-4.5785	-1.6822106298824000	890
**CEMP1**	0.046404	-4.2827	-1.6807679123320100	891
**SLC38A2**	0.04644	-5.3234	-1.680397492704700	892
**LOC1001291**	0.046587	-3.1756	-1.6788873337620800	893

**ENTPD7**	0.046594	-2.4409	-1.6788155168742500	895
**PPM1H**	0.046594	-2.0806	-1.6788155168742500	894
**ST14**	0.046611	-5.7445	-1.6786411404719500	896
**TTC5**	0.046622	-3.8864	-1.678528335875670	897
**ZDHHC18**	0.046661	-5.5829	-1.678128564317660	898
**XPO7**	0.046777	-4.617	-1.6769410826738800	899
**C8orf88**	0.046937	-5.2327	-1.6753070471671300	901
**PSMG1**	0.046937	-2.5161	-1.6753070471671300	900
**PXDN**	0.046937	-5.4708	-1.6753070471671300	902
**IFITM10**	0.046938	-6.4652	-1.6752968485028500	904
**PCDHB2**	0.046938	-6.4427	-1.6752968485028500	903
**TMED5**	0.046944	-3.6902	-1.6752356601761800	905
**TRIM32**	0.04711	-4.7673	-1.6735452647949800	906
**BAG6**	0.047166	-7.7439	-1.6729760881912900	907
**PDCD7**	0.047305	-5.4991	-1.6715656479518700	908
**ALDH3B1**	0.047393	-0.66077	-1.6706744235137700	909
**TMEM169**	0.047466	-4.6642	-1.6699361180125900	910
**GPR78**	0.047509	-4.3835	-1.6695016513889300	911
**ADARB1**	0.047533	-2.9967	-1.6692592953559000	912
**ALDH1A1**	0.047556	-3.1929	-1.669027129458510	913
**FOXN2**	0.047632	-5.2459	-1.668260611653440	914
**HSFX1**	0.047632	-6.6502	-1.668260611653440	915
**IGSF9B**	0.047808	-2.0284	-1.6664892712898500	916
**PHF3**	0.047808	-3.3529	-1.6664892712898500	918
**ZNF232**	0.047808	-2.7666	-1.6664892712898500	917
**IL23A**	0.047811	-3.22	-1.6664591232815900	919
**KRTAP13-4**	0.047925	-4.3591	-1.665314619628150	920
**ARMC2**	0.048075	-4.4501	-1.6638120093102200	922
**GRM4**	0.048075	-4.8457	-1.6638120093102200	923
**PAX8**	0.048075	-3.7416	-1.6638120093102200	921
**PPM1E**	0.048075	-5.3382	-1.6638120093102200	924
**SEBOX**	0.048075	-5.3794	-1.6638120093102200	925

**SAMHD1**	0.048207	-4.1485	-1.6624928126888100	926
**TLR1**	0.048346	-5.2251	-1.6611067789574000	927
**CNTN6**	0.04858	-4.4639	-1.6587806384916500	928
**RAB20**	0.048605	-3.2137	-1.6585326492923700	929
**C18orf54**	0.04866	-2.9892	-1.6579874317982700	930
**SENP1**	0.04866	-4.7541	-1.6579874317982700	931
**IMPDH2**	0.048686	-3.1568	-1.6577298640917500	932
**KIF17**	0.049019	-4.823	-1.6544406978996200	933
**CBFA2T2**	0.049067	-2.6816	-1.6539680571920600	936
**CCDC34**	0.049067	-1.6671	-1.6539680571920600	934
**CTDSPL**	0.049067	-2.5486	-1.6539680571920600	935
**NETO1**	0.049067	-3.4364	-1.6539680571920600	938
**RASGRF1**	0.049067	-3.4235	-1.6539680571920600	937
**CNN1**	0.049092	-1.5747	-1.6537220364179300	939
**CCDC144NI**	0.049181	-4.2493	-1.6528470139051900	941
**RORC**	0.049181	-4.5608	-1.6528470139051900	942
**ZNF726**	0.049181	-2.8753	-1.6528470139051900	940
**P3H1**	0.049338	-5.8853	-1.6513065130243300	944
**SELENOS**	0.049338	-5.5773	-1.6513065130243300	943
**GLT8D2**	0.049395	-3.4394	-1.6507481913112200	945
**LRRC17**	0.049432	-1.1204	-1.6503860471929400	946
**SLC22A17**	0.049486	-2.2261	-1.6498579006813100	947
**OMG**	0.049605	-2.7861	-1.6486956440452500	949
**PRRT4**	0.049605	-2.1787	-1.6486956440452500	948
**OR6K3**	0.049795	-3.527	-1.646844543915810	950
**SMCO4**	0.049877	-2.9333	-1.6460473883903300	951
**LRIT1**	0.049881	-4.7982	-1.6460085295027000	952
**PITPNM3**	0.049943	-0.47564	-1.645406534324350	954
**STON2**	0.049943	-0.011427	-1.645406534324350	953
**AGAP2**	0.050056	-3.6465	-1.644310880840330	955
**IQCH**	0.050134	-5.4743	-1.643555739010640	956
**SLC7A6OS**	0.050207	-2.9877	-1.6428498517379000	957

**TSNARE1**	0.050272	-2.8868	-1.6422220101920300	958
**DHTKD1**	0.050316	-3.8546	-1.6417973768921600	959
**LMLN**	0.050327	-3.8217	-1.641691264810030	960
**ZNF544**	0.050537	-2.8693	-1.6396690242884500	961
**GCHFR**	0.050541	-4.9732	-1.6396305704498800	963
**OR2T11**	0.050541	-3.8	-1.6396305704498800	962
**PCDHAC2**	0.050546	-5.0095	-1.639582506560900	964
**OBP2B**	0.050731	-1.7725	-1.637806799228800	965
**CDYL2**	0.050801	-2.8238	-1.637136254678510	966
**TNRC6B**	0.050863	-4.9921	-1.636542957901440	967
**BABAM1**	0.050934	-1.9523	-1.635864244201630	968
**C17orf62**	0.050966	-4.0976	-1.6355585912696400	969
**CABP7**	0.051128	-4.2492	-1.634013563196680	970
**TMEM258**	0.051151	-3.661	-1.633794523307670	971
**ARPC3**	0.051236	-5.4394	-1.6329857074481400	973
**NDFIP1**	0.051236	-3.3303	-1.6329857074481400	972
**HOXB9**	0.051262	-5.1659	-1.632738518181730	974
**BUD23**	0.051401	-2.4991	-1.6314186956880000	976
**OPA1**	0.051401	-2.0843	-1.6314186956880000	975
**CAMK1G**	0.051423	-7.6573	-1.6312100632190100	978
**URB2**	0.051423	-7.6573	-1.6312100632190100	977
**RXFP2**	0.051557	-3.7847	-1.629940832499040	979
**OR6X1**	0.05164	-5.3825	-1.6291559821980500	980
**MED14OS**	0.05177	-2.8949	-1.6279287117147600	981
**SLC8A1**	0.05177	-3.2892	-1.6279287117147600	982
**DRP2**	0.051781	-5.9259	-1.627824978204270	984
**NDUFB7**	0.051781	-5.0839	-1.627824978204270	983
**CD59**	0.051907	-0.21945	-1.626638005495990	985
**DYNC2H1**	0.052095	-4.1604	-1.6248712162309700	986
**GPI**	0.052095	-4.9179	-1.6248712162309700	988
**MIEF2**	0.052095	-4.3753	-1.6248712162309700	987
**GFOD1**	0.052169	-6.1148	-1.624177166989180	989

**ANP32D**	0.052174	-5.1804	-1.624130299979300	990
**NEO1**	0.052215	-0.61767	-1.6237461249930800	991
**NAP1L1**	0.052399	-2.4875	-1.6220249667687200	992
**SIGLEC8**	0.052441	-2.828	-1.6216327665506700	993
**ARL 3.00**	0.052641	-0.10787	-1.6197685598577300	994
**C12orf80**	0.052641	-2.3308	-1.6197685598577300	995
**POLR2C**	0.052641	-2.4601	-1.6197685598577300	996
**KIAA1958**	0.052746	-6.0469	-1.6187921005245400	997
**AP3S1**	0.052837	-5.1454	-1.6179470825069700	1000
**ARHGEF35**	0.052837	-4.4719	-1.6179470825069700	998
**HSD3B2**	0.052837	-5.1482	-1.6179470825069700	1001
**TTLL11**	0.052837	-5.0514	-1.6179470825069700	999
**CNOT8**	0.052913	-3.7265	-1.6172422374703200	1002
**LTBR**	0.052916	-1.9578	-1.6172144311191300	1003
**BRD3**	0.053087	-4.1943	-1.6156315318281500	1004
**DHRS13**	0.053189	-1.9139	-1.6146892704222500	1005
**NIPSNAP3A**	0.053189	-3.9927	-1.6146892704222500	1006
**CALML4**	0.053229	-4.1458	-1.6143201471739000	1007
**PDCD4**	0.053306	-5.6855	-1.6136102035216800	1008
**CROCC**	0.053343	-1.6284	-1.6132693508677400	1009
**AKR1A1**	0.053463	-5.1768	-1.6121651705282900	1012
**LUZP2**	0.053463	-3.1808	-1.6121651705282900	1011
**SCN1A**	0.053463	-2.4843	-1.6121651705282900	1010
**PLA2G2E**	0.053477	-2.8697	-1.612036477456220	1013
**IL2**	0.053515	-5.2893	-1.6116873022049900	1014
**HSPA14**	0.053521	-1.8617	-1.611632187230060	1015
**PANX3**	0.053763	-7.612	-1.609413285664650	1016
**TNFRSF6B**	0.053817	-3.3322	-1.6089192387507600	1017
**CXCL16**	0.053857	-2.1694	-1.6085535311009700	1018
**MYLIP**	0.053906	-2.6211	-1.6081058322105100	1019
**PEX5L**	0.054037	-2.9602	-1.6069105030164600	1020
**OR6C2**	0.054056	-3.1708	-1.606737325194080	1021

**C8orf76**	0.054081	-5.1372	-1.6065095330278100	1025
**DNAJC8**	0.054081	-2.6326	-1.6065095330278100	1022
**IGFL3**	0.054081	-4.3459	-1.6065095330278100	1023
**TXN2**	0.054081	-5.0225	-1.6065095330278100	1024
**ZNF419**	0.054095	-3.8665	-1.6063820058179600	1026
**ICK**	0.05411	-5.0819	-1.6062453985060600	1027
**TTC3**	0.054265	-1.6808	-1.6048355415831100	1028
**RRH**	0.05432	-3.1866	-1.604336035706290	1029
**SENP8**	0.05432	-5.4015	-1.604336035706290	1031
**VPS33B**	0.05432	-4.1858	-1.604336035706290	1030
**ADCY4**	0.054335	-3.521	-1.604199876279490	1032
**UBAP1**	0.054374	-3.3849	-1.6038460008881000	1033
**SLC41A1**	0.054414	-3.9734	-1.6034832602850000	1034
**PLAC4**	0.054439	-5.0073	-1.603256654506510	1035
**POU4F3**	0.054444	-3.542	-1.6032113432288100	1036
**AMMECR1L**	0.054659	-0.35232	-1.6012660643465000	1037
**NQO1**	0.0547	-5.6877	-1.6008957913570000	1038
**PGA3**	0.054721	-3.0979	-1.6007062243075000	1039
**NMT2**	0.054835	-1.9165	-1.5996781483700400	1040
**IGFBP2**	0.054862	-1.6319	-1.5994349041842900	1041
**B4GALT7**	0.0549	-5.2165	-1.5990927207541100	1045
**GBP1**	0.0549	-4.4435	-1.5990927207541100	1044
**IFT43**	0.0549	-4.3854	-1.5990927207541100	1043
**PTDSS1**	0.0549	-2.9117	-1.5990927207541100	1042
**PCDHGA1**	0.054915	-4.5386	-1.5989576998749200	1046
**MTG2**	0.054944	-4.2666	-1.5986967421338900	1047
**ERN 1.00**	0.055057	-1.6868	-1.5976809439645100	1048
**NRXN3**	0.055095	-5.2571	-1.5973397182436900	1049
**CRTAP**	0.055205	-3.147	-1.5963530065725800	1051
**PRKRA**	0.055205	-0.7471	-1.5963530065725800	1050
**SLC39A10**	0.055205	-5.2487	-1.5963530065725800	1052
**VPS37C**	0.055345	-5.1134	-1.595099435241290	1053

**BUD31**	0.055357	-3.0112	-1.5949921028408000	1054
**CFAP221**	0.055364	-2.2618	-1.5949295007584200	1055
**WNT11**	0.055594	-4.6921	-1.5928760427699500	1056
**MYH9**	0.055639	-3.895	-1.5924750639524900	1058
**PPM1D**	0.055639	-2.6781	-1.5924750639524900	1057
**TMPRSS12**	0.055639	-4.8396	-1.5924750639524900	1060
**TRPC3**	0.055639	-4.6403	-1.5924750639524900	1059
**ACOT9**	0.055672	-1.4718	-1.5921811754685000	1062
**CCDC166**	0.055672	-3.8126	-1.5921811754685000	1063
**CROCC2**	0.055672	-0.42159	-1.5921811754685000	1061
**UPP1**	0.055709	-3.5754	-1.591851827584930	1064
**ZNF205**	0.055846	-5.8342	-1.5906338511138200	1065
**ALOXE3**	0.05591	-4.7865	-1.5900656771415900	1066
**NNMT**	0.056019	-3.0797	-1.5890991857539700	1067
**DOCK10**	0.05611	-5.6072	-1.5882934338698200	1068
**NDUFC1**	0.056143	-1.1109	-1.5880014927464000	1069
**TCTA**	0.056143	-4.3046	-1.5880014927464000	1070
**FAM212B**	0.056289	-3.2696	-1.5867114956493600	1071
**FZD7**	0.056298	-3.1781	-1.5866320616361900	1072
**TKTL2**	0.056411	-2.595	-1.58563557432062	1073
**SYCP2L**	0.056421	-4.1049	-1.585547465404130	1074
**GIPR**	0.056425	-0.21229	-1.5855122252837800	1075
**ANXA4**	0.056477	-4.6202	-1.5850542827916900	1079
**ARNTL**	0.056477	-5.3953	-1.5850542827916900	1081
**FIBCD1**	0.056477	-3.17	-1.5850542827916900	1078
**FRMD4B**	0.056477	-4.6288	-1.5850542827916900	1080
**MOGAT3**	0.056477	-2.5226	-1.5850542827916900	1076
**RAVER2**	0.056477	-2.6225	-1.5850542827916900	1077
**FGFBP3**	0.05654	-3.2077	-1.584499912787370	1082
**FZD4**	0.056585	-4.6477	-1.584104232148680	1083
**PINX1**	0.056744	-3.896	-1.5827081431044500	1084
**TNFAIP1**	0.056786	-3.9963	-1.582339879447120	1085

**S100A5**	0.056819	-3.1255	-1.5820506799022600	1086
**ZNF594**	0.056819	-3.5341	-1.5820506799022600	1087
**NRG4**	0.056973	-5.3745	-1.5807028284099200	1088
**CCDC22**	0.057216	-5.6508	-1.5785818462831100	1093
**KTI12**	0.057216	-4.6464	-1.5785818462831100	1091
**TACC3**	0.057216	-4.7368	-1.5785818462831100	1092
**TDRD15**	0.057216	-4.1906	-1.5785818462831100	1090
**TUBB3**	0.057216	-3.3115	-1.5785818462831100	1089
**ESYT3**	0.057342	-1.8018	-1.5774848677654700	1095
**HOXA6**	0.057342	-2.2675	-1.5774848677654700	1096
**TRIM49**	0.057342	-4.5398	-1.5774848677654700	1097
**ZNF17**	0.057342	-1.3688	-1.5774848677654700	1094
**TM2D1**	0.057344	-3.0488	-1.5774674707073200	1098
**BAG5**	0.057412	-3.2739	-1.576876254589350	1099
**NDUFB2**	0.057601	-5.6252	-1.5752359099540300	1100
**EPM2AIP1**	0.057719	-3.4908	-1.5742139247956200	1101
**PAPD5**	0.057719	-4.2969	-1.5742139247956200	1102
**CNGB1**	0.057807	-1.2118	-1.5734528353116100	1103
**NKX6-3**	0.057807	-1.3526	-1.5734528353116100	1105
**TMPRSS11A**	0.057807	-1.3337	-1.5734528353116100	1104
**HPX**	0.057906	-2.8509	-1.5725976976575900	1106
**TLE6**	0.057906	-4.493	-1.5725976976575900	1107
**AIFM1**	0.058085	-1.9665	-1.5710544525828700	1108
**GJB3**	0.058222	-3.3194	-1.569875832482100	1109
**HDAC11**	0.058222	-4.6121	-1.569875832482100	1110
**ATP6V0A4**	0.058223	-4.891	-1.5698672374298200	1111
**B3GNT7**	0.058225	-3.0229	-1.5698500476731600	1112
**TMEM150B**	0.058252	-2.5722	-1.5696180313462100	1113
**UGT2B17**	0.058349	-4.1641	-1.5687851874830800	1114
**LAMTOR4**	0.058372	-1.4222	-1.5685878685580300	1116
**SLC9A9**	0.058372	-0.64097	-1.5685878685580300	1115
**FN3KRP**	0.058409	-4.7303	-1.568270570550780	1117

**TMEM43**	0.058512	-6.1068	-1.5673881120476400	1118
**FUBP1**	0.058555	-4.2249	-1.5670200679210500	1119
**LAG3**	0.058615	-3.0128	-1.5665068724361800	1120
**ZNHIT3**	0.058615	-4.6176	-1.5665068724361800	1121
**SLC25A53**	0.058747	-2.6789	-1.5653792923297400	1122
**BEX5**	0.058781	-2.7925	-1.5650891771468700	1124
**CCT8**	0.058781	-0.6671	-1.5650891771468700	1123
**LBR**	0.058913	-4.6933	-1.5639640941971900	1125
**SPATC1**	0.058979	-4.4934	-1.5634022943230900	1126
**PPCS**	0.059013	-3.7122	-1.5631130747364100	1127
**ALG10B**	0.059034	-2.9088	-1.562934504407970	1128
**CA10**	0.059444	-1.7452	-1.559458072399120	1129
**MX2**	0.059456	-4.9505	-1.559356606712900	1132
**OR4F16**	0.059456	-2.6735	-1.559356606712900	1130
**TANGO2**	0.059456	-4.8561	-1.559356606712900	1131
**PSMG4**	0.059526	-1.7099	-1.5587650432618900	1133
**GRM8**	0.059532	-2.6199	-1.5587143631988200	1135
**TOMM22**	0.059532	-0.26285	-1.5587143631988200	1134
**MYZAP**	0.059707	-3.2619	-1.5572379526766000	1136
**SH2B1**	0.059844	-2.7075	-1.556084498522800	1137
**C1orf137**	0.059963	-2.4111	-1.555084270554610	1138
**PPIA**	0.059987	-2.4449	-1.554882732328490	1139
**GBX1**	0.060044	-3.4935	-1.5544043319907600	1142
**LOC284108**	0.060044	-2.9104	-1.5544043319907600	1140
**RTN2**	0.060044	-4.8534	-1.5544043319907600	1143
**TXNDC15**	0.060044	-3.3687	-1.5544043319907600	1141
**NANOG**	0.060122	-3.8471	-1.5537502548371200	1144
**RRP12**	0.060274	-5.0435	-1.5524775493484100	1145
**GRIK1**	0.060325	-2.6321	-1.5520510860648600	1146
**CBWD3**	0.060406	-7.4752	-1.5513743416347300	1147
**ITGA4**	0.060545	-3.9606	-1.550214667821360	1148
**YWHAB**	0.060545	-5.6997	-1.550214667821360	1149

**TMEM88**	0.060566	-2.0884	-1.5500396464770100	1150
**VWA3A**	0.060601	-4.0792	-1.5497480497070200	1151
**C17orf107**	0.060758	-3.8593	-1.5484416484188000	1152
**ZNF282**	0.060863	-3.3737	-1.5475694130870400	1153
**ZNF423**	0.060869	-7.4668	-1.5475196066183100	1154
**LRSAM1**	0.060912	-4.7271	-1.5471627725488200	1155
**ZNF518B**	0.060968	-4.1531	-1.5466983535510700	1156
**TMEM187**	0.061078	-0.19347	-1.5457870719365900	1157
**HDAC6**	0.061144	-3.3051	-1.5452409185443300	1158
**EVL**	0.061216	-1.6725	-1.544645640045420	1160
**R3HDM1**	0.061216	-1.156	-1.544645640045420	1159
**DPP3**	0.061367	-2.1485	-1.5433989833652200	1161
**TAF13**	0.061383	-3.6195	-1.5432670277657300	1162
**KIF1BP**	0.061546	-3.4823	-1.5419242588036900	1163
**UBC**	0.061604	-4.245	-1.541447133673400	1164
**SSPO**	0.061612	-3.2116	-1.5413813508430000	1165
**EXPH5**	0.061659	-3.5346	-1.5409950113527200	1166
**OR2V1**	0.061757	-2.7367	-1.5401901913795600	1167
**OR5R1**	0.061757	-5.5452	-1.5401901913795600	1169
**ZNF660**	0.061757	-5.0893	-1.5401901913795600	1168
**FNDC4**	0.061802	-1.4443	-1.5398209651790900	1170
**TXNRD2**	0.061976	-3.6364	-1.5383952619448500	1171
**ZPBP**	0.062063	-2.6252	-1.5376835814172100	1172
**MAPK8IP1**	0.062078	-3.9958	-1.5375609565648900	1173
**COQ9**	0.062183	-2.9811	-1.5367032292483900	1176
**SERAC1**	0.062183	-1.276	-1.5367032292483900	1175
**SLC22A11**	0.062183	-0.63817	-1.5367032292483900	1174
**TBC1D12**	0.062183	-3.1441	-1.5367032292483900	1177
**TMEM126A**	0.062198	-4.627	-1.5365807890267500	1178
**MVK**	0.062253	-6.4554	-1.5361320385147500	1179
**RPRD2**	0.062299	-0.24184	-1.5357569573167200	1180
**HAS2**	0.062519	-3.8192	-1.5339660714201700	1181

**FOLR1**	0.062546	-4.1349	-1.5337466195586700	1182
**KRTAP23-1**	0.06267	-0.40128	-1.532739713899260	1183
**UBE2T**	0.062693	-4.0022	-1.5325531199010000	1185
**ZNF385D**	0.062693	-1.6592	-1.5325531199010000	1184
**MRPL18**	0.062701	-5.2208	-1.5324882301467300	1187
**SMOC2**	0.062701	-3.9696	-1.5324882301467300	1186
**MAK**	0.062729	-2.48	-1.5322611668074800	1188
**DNAH3**	0.062751	-3.1426	-1.532082815303030	1189
**NADSYN1**	0.062926	-2.3542	-1.5306658426796500	1190
**RNF214**	0.063053	-5.8499	-1.5296394466188900	1191
**LAMP5**	0.063107	-5.4696	-1.5292035141001400	1192
**JAK1**	0.063241	-7.4237	-1.5281230090952400	1193
**ARL5C**	0.063472	-1.5704	-1.526264525228500	1194
**EXOSC9**	0.063612	-5.1624	-1.525140732063420	1195
**SVIL**	0.063706	-3.8204	-1.5243872646543400	1196
**ETV5**	0.06392	-1.174	-1.5226751442106600	1197
**ANKRD33B**	0.064116	-1.7658	-1.521110940553010	1198
**TMTC1**	0.064143	-4.3107	-1.520895754967890	1199
**ZNF33B**	0.06415	-7.4061	-1.5208399776069500	1200
**ZNF485**	0.06415	-7.4061	-1.5208399776069500	1201
**BMS1**	0.06429	-1.0916	-1.5197254226241900	1203
**HEMGN**	0.06429	-1.0639	-1.5197254226241900	1202
**RRAGA**	0.06429	-3.4578	-1.5197254226241900	1204
**PTCHD3**	0.064434	-5.3787	-1.5185809893387600	1205
**MMP21**	0.064446	-4.9076	-1.518485709636300	1206
**TUBGCP4**	0.064506	-3.1343	-1.5180095177743700	1207
**PICALM**	0.06452	-4.7252	-1.5178984558561000	1209
**RIC3**	0.06452	-5.23	-1.5178984558561000	1210
**RNF103-CH**	0.06452	-4.1291	-1.5178984558561000	1208
**C5orf49**	0.064767	-0.54204	-1.5159420778962900	1211
**F12**	0.064768	-5.2114	-1.51593416912382	1212
**ZNF25**	0.064771	-2.8379	-1.5159104433753100	1213

**OR5B17**	0.064882	-3.0083	-1.515033189915340	1214
**TBC1D10A**	0.06489	-1.7243	-1.5149700094696100	1215
**SIN3B**	0.064934	-2.6634	-1.5146226250674800	1216
**FAM90A1**	0.065029	-3.0726	-1.5138732132716100	1217
**RPL17-C18c**	0.065141	-0.25579	-1.5129907868383900	1218
**ODF3L2**	0.065197	-4.1275	-1.512550015070730	1219
**CHRNA3**	0.065222	-5.1945	-1.5123533368013700	1220
**BAHCC1**	0.065287	-4.4732	-1.511842246900350	1222
**HNRNPA2B1**	0.065287	-5.2626	-1.511842246900350	1224
**NDUFB10**	0.065287	-3.4257	-1.511842246900350	1221
**SHISA9**	0.065287	-4.6693	-1.511842246900350	1223
**TBL2**	0.065356	-4.0516	-1.5113001370768600	1225
**STRN**	0.065397	-3.0484	-1.5109782240980800	1226
**GALR3**	0.065505	-4.773	-1.510131006813730	1227
**C3**	0.065518	-3.0308	-1.5100291000183800	1228
**DEFB114**	0.065518	-3.1469	-1.5100291000183800	1229
**SYT1**	0.065582	-4.5692	-1.50952763351515	1230
**RAET1G**	0.065731	-2.6821	-1.5083616251230600	1231
**MAN2C1**	0.065852	-3.3604	-1.507416239040480	1232
**CH25H**	0.065913	-3.2461	-1.506940149812700	1234
**CTNNA3**	0.065913	-1.2416	-1.506940149812700	1233
**SLC35A4**	0.066018	-3.7378	-1.5061214510347000	1235
**RAB19**	0.066062	-3.7337	-1.5057786772162900	1236
**UBE2V2**	0.066333	-4.3659	-1.5036713921821600	1237
**RALGAPB**	0.066354	-3.3981	-1.503508375573750	1238
**TMEM209**	0.066379	-3.479	-1.5033143602633800	1239
**EME1**	0.066406	-5.5003	-1.5031048872677200	1240
**C8orf33**	0.066431	-1.8477	-1.5029109895801700	1241
**CD300C**	0.066504	-0.51011	-1.5023451314744300	1243
**HOOK2**	0.066504	-0.26434	-1.5023451314744300	1242
**RUNX2**	0.066504	-2.6633	-1.5023451314744300	1244
**SAPCD2**	0.066504	-4.116	-1.5023451314744300	1245

**ODF4**	0.066733	-1.9796	-1.5005731565122500	1246
**DOK1**	0.066742	-3.9018	-1.5005036117449300	1247
**APOA2**	0.066811	-4.1823	-1.4999706761323900	1248
**DIDO1**	0.066811	-4.9597	-1.4999706761323900	1252
**HLA-DQA1**	0.066811	-5.062	-1.4999706761323900	1253
**LCE3A**	0.066811	-4.4757	-1.4999706761323900	1250
**NUDT6**	0.066811	-4.3906	-1.4999706761323900	1249
**WDR60**	0.066811	-4.57	-1.4999706761323900	1251
**CHRNA2**	0.066832	-1.3838	-1.499808562868190	1254
**SLC6A11**	0.066843	-1.0491	-1.4997236621247900	1255
**PEX11G**	0.067111	-4.3551	-1.4976585028767500	1257
**PUS3**	0.067111	-0.92128	-1.4976585028767500	1256
**SDR42E2**	0.067111	-4.6542	-1.4976585028767500	1258
**SYTL1**	0.067177	-0.38239	-1.4971508979065400	1259
**IGDCC4**	0.067406	-3.139	-1.4953926469266900	1260
**AKAP8**	0.067515	-5.0097	-1.494557371631620	1262
**KRTAP10-5**	0.067515	-3.5366	-1.494557371631620	1261
**ACBD6**	0.067588	-4.0004	-1.493998549564720	1264
**DEGS2**	0.067588	-1.7468	-1.493998549564720	1263
**IFIT2**	0.067772	-4.4262	-1.492592077898960	1265
**WRNIP1**	0.067888	-3.5807	-1.4917069044776600	1266
**ABHD17C**	0.067961	-1.2744	-1.491150454345430	1267
**ATP11B**	0.068063	-1.6216	-1.4903737211121400	1268
**TAS2R9**	0.068072	-3.9146	-1.4903052289833100	1269
**C4orf17**	0.068139	-5.0099	-1.48979556274552	1270
**C15orf57**	0.068163	-3.2126	-1.4896130898403900	1272
**RNF111**	0.068163	-2.6173	-1.4896130898403900	1271
**SCML1**	0.068214	-4.405	-1.4892254995143500	1273
**PAFAH2**	0.068255	-3.7546	-1.4889140694341000	1274
**ENTPD3**	0.068266	-1.3499	-1.4888305395868400	1275
**KCNT2**	0.068281	-4.8064	-1.488716651987830	1276
**RASSF2**	0.06839	-4.7424	-1.4878896481308000	1277

**IPPK**	0.068404	-2.4818	-1.4877835011713900	1278
**DAZ1**	0.068531	-7.3147	-1.4868213614519500	1279
**LLPH**	0.068547	-0.86672	-1.4867002445826000	1280
**SLC2A4RG**	0.068547	-1.7269	-1.4867002445826000	1281
**HDGFRP3**	0.068554	-5.0417	-1.486647262809450	1282
**GTSCR1**	0.068639	-3.895	-1.4860042454400500	1283
**PLEKHA3**	0.068839	-4.9665	-1.4844936821930800	1284
**IMPACT**	0.068911	-4.3553	-1.483950707755050	1285
**ETV1**	0.068998	-2.1178	-1.4832951968060300	1286
**MAPK14**	0.069007	-2.4279	-1.4832274216966100	1288
**MGEA5**	0.069007	-0.77638	-1.4832274216966100	1287
**MYOM2**	0.069007	-3.4179	-1.4832274216966100	1289
**CCR3**	0.069033	-2.028	-1.4830316651977200	1290
**RNASE8**	0.069079	-1.7033	-1.4826854659291400	1291
**YIPF2**	0.069219	-2.0769	-1.4816329079283300	1292
**TRIM55**	0.06923	-3.2148	-1.4815502764689600	1293
**UTP6**	0.069276	-3.2351	-1.481204836285010	1294
**TRIM67**	0.069357	-4.1992	-1.4805969904613700	1295
**RSPO3**	0.069474	-1.7971	-1.4797199555271000	1296
**CLEC1A**	0.069509	-2.8194	-1.479457815695610	1297
**GYS1**	0.069509	-5.1607	-1.479457815695610	1300
**HES3**	0.069509	-4.1478	-1.479457815695610	1298
**ICAM4**	0.069509	-4.6815	-1.479457815695610	1299
**LRP1**	0.069577	-3.9193	-1.4789488058875300	1302
**PTCH2**	0.069577	-2.9985	-1.4789488058875300	1301
**OCLN**	0.069664	-4.954	-1.4782981309729800	1303
**ABCC1**	0.069688	-0.71974	-1.4781187445445000	1304
**CYP4X1**	0.069688	-1.6233	-1.4781187445445000	1305
**LRPPRC**	0.069688	-4.0187	-1.4781187445445000	1307
**SPATA8**	0.069688	-1.6328	-1.4781187445445000	1306
**ATP6AP1L**	0.069716	-5.3176	-1.4779095204768600	1308
**RBM17**	0.069743	-1.763	-1.4777078299494800	1309

**TRIT1**	0.069839	-3.8202	-1.4769911944572100	1310
**WDR44**	0.06988	-3.7965	-1.476685362409400	1311
**GAS2L1**	0.069992	-3.4871	-1.4758506219872800	1312
**NFE2L1**	0.070032	-2.3856	-1.4755527494828100	1313
**OR2AG1**	0.070057	-4.3178	-1.4753666456315600	1314
**PSMB4**	0.07006	-2.2888	-1.4753443166029900	1315
**HAPLN3**	0.070169	-1.0558	-1.474533526963310	1316
**PCBD2**	0.070173	-7.2862	-1.4745037916489700	1317
**FAM167B**	0.070311	-3.298	-1.4734787206870400	1318
**C2orf82**	0.070407	-1.5932	-1.472766539978430	1321
**CSAG3**	0.070407	-1.31	-1.472766539978430	1320
**PRSS37**	0.070407	-0.99526	-1.472766539978430	1319
**FBXO5**	0.070432	-1.6215	-1.4725811987951400	1323
**KIF18B**	0.070432	-0.15264	-1.4725811987951400	1322
**FAM169B**	0.07051	-4.7714	-1.4720032591813800	1327
**MCAT**	0.07051	-3.7277	-1.4720032591813800	1324
**PDLIM2**	0.07051	-4.0596	-1.4720032591813800	1325
**ZNF346**	0.07051	-4.7661	-1.4720032591813800	1326
**KLRF2**	0.070546	-4.1672	-1.471736683572030	1328
**COL3A1**	0.070559	-3.1295	-1.4716404458552500	1329
**FAM124A**	0.070674	-4.668	-1.4707897051680300	1330
**ZNF563**	0.070698	-2.5153	-1.4706122934862300	1331
**NPIPB8**	0.070705	-2.8628	-1.4705605571304800	1332
**PTPRE**	0.07075	-3.2684	-1.4702280602194100	1333
**CT45A6**	0.070925	-4.1665	-1.4689365592671300	1334
**SPATA16**	0.071079	-3.7535	-1.4678020617335700	1335
**CKAP2**	0.07108	-2.7458	-1.4677947010381300	1337
**MYO5B**	0.07108	-4.8078	-1.4677947010381300	1340
**RNF19B**	0.07108	-2.021	-1.4677947010381300	1336
**SCO2**	0.07108	-3.2334	-1.4677947010381300	1338
**TMEM138**	0.07108	-3.2608	-1.4677947010381300	1339
**KAT14**	0.071222	-2.9243	-1.4667502886838400	1341

**OPN4**	0.071544	-0.29712	-1.4643878823204600	1342
**SLAIN2**	0.071544	-0.38066	-1.4643878823204600	1343
**CEACAM7**	0.071638	-2.6836	-1.4636997746671500	1344
**HNRNPUL1**	0.071638	-4.9773	-1.4636997746671500	1346
**RBM10**	0.071638	-5.4602	-1.4636997746671500	1347
**ZNRD1**	0.071638	-4.374	-1.4636997746671500	1345
**ESR2**	0.071653	-3.6322	-1.4635900343549300	1348
**ELF1**	0.071733	-1.4763	-1.463005050154070	1349
**RPS12**	0.071749	-2.4616	-1.4628881133743	1350
**AMPD3**	0.071758	-4.589	-1.4628223452254700	1353
**CDKL2**	0.071758	-3.4595	-1.4628223452254700	1352
**EIF3L**	0.071758	-2.7784	-1.4628223452254700	1351
**CLCNKA**	0.071818	-1.4665	-1.4623840525112500	1354
**TMX1**	0.071991	-3.9753	-1.46112187870332	1355
**GLB1L2**	0.072015	-2.1146	-1.4609469632049400	1356
**SEC14L6**	0.072068	-1.7714	-1.460560849744010	1357
**ERG**	0.072197	-2.9889	-1.4596219730072600	1358
**FNDC9**	0.072234	-2.1004	-1.4593529200883800	1359
**DDAH2**	0.072282	-5.2782	-1.4590040358079500	1362
**HELB**	0.072282	-3.8808	-1.4590040358079500	1361
**ZNF410**	0.072282	-2.7632	-1.4590040358079500	1360
**EPHA5**	0.072326	-4.1771	-1.4586843811518100	1363
**DPH1**	0.072361	-2.9543	-1.4584302167960700	1364
**CEP290**	0.072394	-1.5171	-1.4581906623795600	1365
**OR4D9**	0.072573	-3.2244	-1.4568927167777400	1366
**MAGOH**	0.072601	-2.1264	-1.4566899080425100	1367
**DSC2**	0.072693	-2.8511	-1.4560239579802600	1368
**SRPRB**	0.072693	-4.9613	-1.4560239579802600	1369
**DNAJC24**	0.072718	-4.6095	-1.4558431048225200	1370
**DDX10**	0.072735	-3.4094	-1.4557201518691100	1372
**PINLYP**	0.072735	-4.2538	-1.4557201518691100	1373
**SOD2**	0.072735	-1.7286	-1.4557201518691100	1371

**C1orf61**	0.072758	-2.8235	-1.455553838775620	1374
**LRGUK**	0.072867	-4.5379	-1.4547662063072400	1375
**EVI2B**	0.072986	-7.2273	-1.4539073431905700	1376
**PITPNM2**	0.073117	-4.3887	-1.4529631104978000	1377
**PTPRO**	0.073146	-3.5549	-1.4527542569294600	1378
**ASAP2**	0.073203	-2.6575	-1.4523439362208500	1379
**MFSD9**	0.073203	-4.6341	-1.4523439362208500	1380
**ACOX3**	0.073324	-3.5691	-1.4514737140917700	1381
**LONRF3**	0.073514	-4.0438	-1.4501094635893200	1382
**DSC1**	0.073654	-2.5195	-1.4491059507132700	1384
**MAZ**	0.073654	-4.8758	-1.4491059507132700	1385
**PRDX1**	0.073654	-1.7118	-1.4491059507132700	1383
**LRFN5**	0.073665	-4.5359	-1.4490271650858100	1389
**SCO1**	0.073665	-3.4243	-1.4490271650858100	1388
**SGK2**	0.073665	-1.3545	-1.4490271650858100	1386
**THBS4**	0.073665	-1.3612	-1.4490271650858100	1387
**C9orf163**	0.073715	-4.5517	-1.4486691619053000	1391
**LHFPL2**	0.073715	-4.3332	-1.4486691619053000	1390
**PPP1R12B**	0.073715	-4.6788	-1.4486691619053000	1392
**CERK**	0.073836	-4.3895	-1.4478035616859200	1393
**SPECC1**	0.07422	-2.1862	-1.4450636918327400	1394
**TREML2**	0.074277	-2.1012	-1.444657915947010	1395
**EPSTI1**	0.074364	-0.40327	-1.444039032026090	1397
**IL6ST**	0.074364	-0.024866	-1.444039032026090	1396
**SERPINA1**	0.074364	-3.6635	-1.444039032026090	1400
**TRAPPC3**	0.074364	-0.7323	-1.444039032026090	1398
**YPEL2**	0.074364	-2.4273	-1.444039032026090	1399
**GDE1**	0.074372	-1.9519	-1.4439821509214700	1401
**DRD3**	0.074653	-4.9747	-1.4419871591258200	1402
**ARHGEF2**	0.074711	-1.6384	-1.4415760952973900	1403
**GNS**	0.074711	-4.7502	-1.4415760952973900	1407
**OSR1**	0.074711	-4.2968	-1.4415760952973900	1405

**PODXL2**	0.074711	-4.312	-1.4415760952973900	1406
**SULT6B1**	0.074711	-2.6383	-1.4415760952973900	1404
**KCNT1**	0.074793	-4.4329	-1.4409953513963400	1408
**POTED**	0.074932	-3.4817	-1.4400120283498100	1409
**RAB11FIP5**	0.074994	-2.2886	-1.4395738725231200	1410
**DUSP1**	0.075039	-3.4775	-1.4392560292037800	1411
**AGPAT4**	0.075071	-4.4877	-1.4390300957176400	1412
**OR6B1**	0.075153	-3.6429	-1.4384514761990900	1413
**AASDH**	0.075171	-1.4642	-1.4383245266003200	1414
**ETV4**	0.075244	-4.3124	-1.4378099129308900	1415
**PGBD4**	0.075301	-4.68	-1.4374083558704800	1416
**CCL16**	0.075327	-4.0978	-1.4372252664420700	1417
**INPP4A**	0.075573	-3.3715	-1.435495338159860	1418
**ACTG1**	0.075781	-4.0461	-1.43403597912354	1421
**CSN1S1**	0.075781	-3.2088	-1.43403597912354	1419
**NSG1**	0.075781	-3.6242	-1.43403597912354	1420
**ZNF639**	0.075781	-4.1205	-1.43403597912354	1422
**PPP1R11**	0.075876	-2.5877	-1.4333704597814200	1423
**B3GALT1**	0.075909	-0.91668	-1.433139427881780	1424
**BSN**	0.075909	-3.3458	-1.433139427881780	1427
**GPR157**	0.075909	-3.6475	-1.433139427881780	1430
**NTAN1**	0.075909	-3.8218	-1.433139427881780	1431
**RBM14-RBM**	0.075909	-3.507	-1.433139427881780	1429
**SULF2**	0.075909	-2.0682	-1.433139427881780	1425
**ZG16**	0.075909	-3.4958	-1.433139427881780	1428
**ZNF711**	0.075909	-2.1122	-1.433139427881780	1426
**FDXACB1**	0.075926	-3.8614	-1.4330204412960900	1432
**ZNF141**	0.076179	-4.3271	-1.4312520335866200	1433
**CAPN3**	0.07648	-1.9064	-1.429153930913220	1434
**LRRC59**	0.076504	-3.469	-1.4289869109523700	1435
**CCDC70**	0.076662	-1.5255	-1.427888356582130	1436
**PDGFA**	0.076741	-4.6464	-1.4273397250072600	1437

**VCAN**	0.076839	-7.1448	-1.4266597405470000	1438
**C1RL**	0.076926	-3.0935	-1.426056633135450	1441
**KRTAP19-1**	0.076926	-2.9341	-1.426056633135450	1440
**SLIRP**	0.076926	-2.6633	-1.426056633135450	1439
**ZNF645**	0.076926	-4.8141	-1.426056633135450	1442
**CRYGB**	0.076978	-2.5945	-1.4256964026719400	1444
**NPPC**	0.076978	-2.4934	-1.4256964026719400	1443
**SLC28A1**	0.077033	-2.1745	-1.4253155908984300	1445
**HS6ST3**	0.077147	-1.5717	-1.4245269294901800	1446
**YWHAG**	0.077276	-2.3575	-1.4236355640969600	1447
**CDKL5**	0.077581	-4.1518	-1.421532558869170	1448
**CPEB4**	0.077749	-1.8682	-1.4203768624113100	1451
**PTGS1**	0.077749	-3.1922	-1.4203768624113100	1452
**SERPINA12**	0.077749	-1.6776	-1.4203768624113100	1450
**TNFRSF13B**	0.077749	-0.79594	-1.4203768624113100	1449
**CDC20B**	0.0778	-2.0558	-1.4200264010952900	1453
**OXLD1**	0.0778	-4.3838	-1.4200264010952900	1455
**VDAC2**	0.0778	-2.7021	-1.4200264010952900	1454
**UBAP2L**	0.077996	-2.4506	-1.4186811505944900	1456
**CCR9**	0.078191	-2.7426	-1.4173453065569000	1457
**GTF2F1**	0.078269	-2.9202	-1.416811676454110	1458
**GPSM2**	0.078321	-0.44834	-1.41645614707037	1459
**EHHADH**	0.078381	-4.9463	-1.4160461432098900	1460
**ARHGAP31**	0.078398	-1.3929	-1.4159300187166200	1461
**ABCA5**	0.078409	-3.3849	-1.4158548895119300	1462
**CLRN2**	0.078547	-2.1497	-1.4149130377494600	1463
**JTB**	0.078547	-4.6477	-1.4149130377494600	1465
**TTYH3**	0.078547	-3.8844	-1.4149130377494600	1464
**PHOX2A**	0.078557	-2.1034	-1.41484483637874	1466
**GRWD1**	0.078625	-3.8714	-1.414381241489040	1467
**CPD**	0.078789	-1.4878	-1.4132644085415300	1468
**BYSL**	0.078891	-0.89227	-1.4125706810953000	1470

**NUP133**	0.078891	-2.3242	-1.4125706810953000	1471
**RNASEH2A**	0.078891	-0.76246	-1.4125706810953000	1469
**KRT17**	0.078937	-5.0668	-1.4122580459231200	1472
**SLC16A5**	0.078976	-3.3161	-1.4119930937521600	1473
**CYP4F2**	0.079052	-2.6517	-1.411477061297140	1474
**FASTKD5**	0.079071	-3.7352	-1.4113481118957300	1475
**METTL4**	0.079104	-4.1096	-1.4111242029062400	1476
**PWP2**	0.079148	-1.6607	-1.4108257675934600	1477
**TRMT10C**	0.079336	-2.1632	-1.4095520480913500	1478
**C8orf46**	0.079414	-3.106	-1.4090242605952700	1479
**OR2L5**	0.079414	-4.2756	-1.4090242605952700	1480
**HMG20B**	0.079512	-5.322	-1.408361698830650	1481
**TEX12**	0.079572	-4.0129	-1.4079563537140400	1482
**ARL6IP6**	0.079591	-1.6728	-1.4078280426419700	1483
**H2AFB2**	0.079601	-4.6486	-1.4077605198075200	1484
**MSH6**	0.079769	-1.2697	-1.4066270945827700	1485
**ASXL2**	0.079802	-2.5961	-1.4064046697109900	1486
**NRIP1**	0.079842	-0.4687	-1.4061351570543600	1487
**STK11**	0.079845	-3.7894	-1.406114947722180	1488
**SEC22C**	0.079896	-7.0907	-1.405771476904280	1489
**TBC1D3H**	0.079896	-7.0907	-1.405771476904280	1491
**TDGF1**	0.079896	-7.0907	-1.405771476904280	1490
**NCOA4**	0.079981	-3.8149	-1.4051993938020100	1492
**AGAP11**	0.080106	-1.5028	-1.4043589296324200	1493
**MPZL2**	0.080243	-3.124	-1.4034389188839000	1496
**PLCG2**	0.080243	-0.87357	-1.4034389188839000	1495
**PLCH2**	0.080243	-3.841	-1.4034389188839000	1497
**ZNF718**	0.080243	-0.60953	-1.4034389188839000	1494
**EIF4EBP2**	0.080306	-4.5541	-1.4030162466954100	1502
**EXOC1**	0.080306	-2.519	-1.4030162466954100	1498
**GLRA3**	0.080306	-4.2087	-1.4030162466954100	1499
**MMADHC**	0.080306	-4.983	-1.4030162466954100	1503

**RND2**	0.080306	-4.5227	-1.4030162466954100	1501
**TPTE2**	0.080306	-4.4045	-1.4030162466954100	1500
**SPAST**	0.080326	-1.6936	-1.4028821174513000	1504
**AFG3L2**	0.080366	-4.8116	-1.4026139346532500	1505
**USP17L24**	0.080449	-7.0797	-1.4020577769525700	1506
**PIAS3**	0.080671	-3.7709	-1.4005723498612000	1507
**SAMD8**	0.080695	-4.2161	-1.400411948156080	1508
**VPS33A**	0.080717	-2.8183	-1.4002649449051600	1509
**ITPA**	0.080774	-4.4903	-1.3998842135421400	1510
**KCNK7**	0.080779	-3.7916	-1.3998508257337400	1511
**PTCD2**	0.080801	-0.30432	-1.3997039379119200	1512
**DEFA6**	0.080885	-0.41465	-1.399143371100300	1513
**FAM185A**	0.080885	-0.83197	-1.399143371100300	1514
**IGSF9**	0.080913	-3.5047	-1.3989566131648000	1515
**ZNF85**	0.080925	-2.1096	-1.3988765889843500	1516
**ECSIT**	0.081143	-0.092669	-1.397424373296660	1517
**P2RX3**	0.081143	-0.25332	-1.397424373296660	1518
**RAB21**	0.081206	-4.0119	-1.397005244737160	1520
**TENM4**	0.081206	-1.2653	-1.397005244737160	1519
**INTU**	0.081314	-3.8088	-1.3962873090557300	1521
**AMZ1**	0.081354	-5.1353	-1.3960215895025600	1525
**CDK20**	0.081354	-4.6417	-1.3960215895025600	1523
**PRC1**	0.081354	-2.7515	-1.3960215895025600	1522
**SSH1**	0.081354	-4.7569	-1.3960215895025600	1524
**NEIL1**	0.081386	-5.0689	-1.3958090848076100	1526
**H3F3B**	0.081616	-1.912	-1.3942835585693600	1527
**ACTC1**	0.081634	-2.4846	-1.3941643064012200	1530
**OR52N4**	0.081634	-2.3999	-1.3941643064012200	1529
**SRGN**	0.081634	-3.148	-1.3941643064012200	1531
**ZFHX4**	0.081634	-3.7523	-1.3941643064012200	1532
**ZNF606**	0.081634	-1.2679	-1.3941643064012200	1528
**PPP2R5C**	0.081682	-5.1694	-1.3938463975105700	1533

**CNKSR3**	0.081703	-4.8884	-1.3937073566577200	1534
**NBPF12**	0.081756	-1.4848	-1.3933565637869300	1535
**OR4A16**	0.081814	-2.4571	-1.3929728736321400	1536
**GPR63**	0.082182	-0.47395	-1.3905431901243	1537
**ARHGAP5**	0.082329	-3.1536	-1.389574927834660	1538
**CPS1**	0.082329	-4.6565	-1.389574927834660	1540
**GPRC5A**	0.082329	-4.031	-1.389574927834660	1539
**INHBC**	0.082376	-4.5472	-1.3892656220706700	1541
**ST3GAL1**	0.082395	-4.5169	-1.3891406212875700	1542
**ASB18**	0.082571	-4.8381	-1.3879837496444500	1548
**CASKIN2**	0.082571	-0.51832	-1.3879837496444500	1543
**CPN1**	0.082571	-3.2895	-1.3879837496444500	1544
**HIST3H2BB**	0.082571	-3.6506	-1.3879837496444500	1546
**RNF43**	0.082571	-3.39	-1.3879837496444500	1545
**SCAI**	0.082571	-3.6649	-1.3879837496444500	1547
**TRIM73**	0.082571	-5.0266	-1.3879837496444500	1549
**UBN2**	0.082598	-3.7522	-1.3878064392871200	1550
**HCN3**	0.082669	-4.0969	-1.3873403868173300	1551
**CCL28**	0.082787	-3.9718	-1.386566486647130	1552
**LRRC52**	0.082901	-2.2142	-1.3858196082553800	1553
**CASP5**	0.082918	-5.2815	-1.3857082978684700	1554
**DBP**	0.083004	-5.1342	-1.3851454612035800	1555
**SBSN**	0.083066	-4.8249	-1.3847399672726100	1556
**GTF2H2C**	0.083111	-4.3338	-1.3844457996999500	1558
**PTGER1**	0.083111	-1.9503	-1.3844457996999500	1557
**FKBP4**	0.083338	-4.1844	-1.3829637108959500	1560
**FSIP1**	0.083338	-4.7748	-1.3829637108959500	1561
**MOGAT2**	0.083338	-4.1664	-1.3829637108959500	1559
**PLEKHG4B**	0.08334	-2.9246	-1.3829506663405000	1562
**CRHR2**	0.083463	-2.8408	-1.3821488780192900	1563
**CLDN9**	0.083591	-0.81431	-1.3813154390446100	1564
**FAM205A**	0.083591	-4.0068	-1.3813154390446100	1565

**IWS1**	0.083603	-4.7659	-1.3812373533198900	1566
**PRRX1**	0.083645	-3.944	-1.3809641195838600	1567
**APOBEC4**	0.08369	-3.6965	-1.380671483514090	1568
**POFUT2**	0.083721	-3.0801	-1.3804699585372800	1569
**LOC1001304**	0.083962	-0.16197	-1.3789051729351500	1570
**ARHGAP22**	0.084215	-1.5234	-1.3772660972352700	1571
**BPGM**	0.084215	-3.0396	-1.3772660972352700	1573
**CD300LB**	0.084215	-3.2814	-1.3772660972352700	1574
**OR5AC2**	0.084215	-1.8068	-1.3772660972352700	1572
**TCTE1**	0.084264	-3.2784	-1.3769490751903400	1575
**TCF7**	0.084308	-5.1138	-1.3766645202128200	1576
**UCN3**	0.084347	-1.5629	-1.376412394184890	1577
**IGBP1**	0.084385	-3.2273	-1.3761668170570400	1578
**DLG1**	0.084397	-4.5315	-1.3760892836251500	1582
**EFHC1**	0.084397	-2.2993	-1.3760892836251500	1581
**ERCC4**	0.084397	-1.7116	-1.3760892836251500	1580
**GREB1**	0.084397	-4.8671	-1.3760892836251500	1583
**OR4N5**	0.084397	-1.4366	-1.3760892836251500	1579
**GDF11**	0.084543	-1.7518	-1.375146622054530	1584
**STK38L**	0.084605	-4.2858	-1.3747466831105300	1585
**DNAJC3**	0.08465	-2.6452	-1.374456542505250	1587
**MTRNR2L1(**	0.08465	-1.5576	-1.374456542505250	1586
**ZNF10**	0.084679	-3.5544	-1.3742696242957400	1588
**XPNPEP1**	0.085015	-4.0757	-1.3721074432603000	1589
**EXTL1**	0.085054	-4.8633	-1.3718568909943800	1590
**HRNR**	0.08507	-3.3166	-1.3717541252300400	1591
**BTN3A1**	0.085081	-2.3555	-1.3716834821696800	1593
**CD300E**	0.085081	-0.49299	-1.3716834821696800	1592
**OR52B2**	0.085081	-4.302	-1.3716834821696800	1594
**TTC39A**	0.0851	-4.8338	-1.371561478458870	1595
**SLAMF1**	0.08511	-6.988	-1.3714972741788200	1596
**CCDC122**	0.085319	-1.3639	-1.3701566965504600	1597

**ITGAE**	0.085466	-1.8399	-1.3692152752336200	1598
**EXOSC5**	0.08554	-4.3124	-1.3687418213155300	1599
**ISX**	0.085569	-3.5277	-1.3685563622169200	1600
**MBNL1**	0.085581	-3.2588	-1.368479634288030	1601
**HGS**	0.085644	-6.9761	-1.3680769447743500	1602
**MLH3**	0.085665	-2.0386	-1.3679427642236500	1605
**NASP**	0.085665	-0.46697	-1.3679427642236500	1603
**RASSF4**	0.085665	-1.4072	-1.3679427642236500	1604
**PTPN6**	0.08569	-1.1033	-1.3677830575786300	1606
**ATP4A**	0.085715	-2.3147	-1.3676233858129700	1607
**NUDT8**	0.085715	-3.1288	-1.3676233858129700	1608
**OR2L3**	0.085715	-4.1692	-1.3676233858129700	1610
**ZNF48**	0.085715	-3.9458	-1.3676233858129700	1609
**CES5A**	0.08583	-3.5494	-1.3668893443903400	1611
**DENND1A**	0.085902	-4.0005	-1.3664301452794300	1612
**TRMT61B**	0.086103	-3.3447	-1.365149736968390	1613
**KITLG**	0.086128	-3.635	-1.3649906386284400	1614
**NOL3**	0.086159	-2.5543	-1.3647934046588300	1615
**IMP4**	0.086168	-4.0344	-1.364736153127130	1616
**CLRN1**	0.08623	-2.9658	-1.364341875180600	1617
**SP8**	0.086304	-1.3695	-1.3638715628419300	1618
**LRP12**	0.086428	-3.5061	-1.363084147515510	1621
**SHPRH**	0.086428	-0.68134	-1.363084147515510	1619
**TUBGCP2**	0.086428	-0.91292	-1.363084147515510	1620
**AHR**	0.086531	-2.3114	-1.3624307267639400	1622
**SLC30A2**	0.086606	-2.259	-1.3619553007004900	1623
**CAMKMT**	0.08668	-4.5182	-1.3614865151713700	1624
**MXD4**	0.086775	-2.1112	-1.3608851341742100	1625
**RSPH4A**	0.086916	-1.8372	-1.3599934644721400	1626
**EIF3J**	0.086934	-1.9435	-1.3598797121026100	1627
**KRT36**	0.087015	-0.59187	-1.3593680440605000	1628
**LTK**	0.087015	-2.1645	-1.3593680440605000	1629

**LZIC**	0.087016	-0.3943	-1.3593617293948000	1630
**COL4A2**	0.08705	-4.2849	-1.3591470630047100	1632
**KCTD12**	0.08705	-3.8286	-1.3591470630047100	1631
**HHAT**	0.087178	-3.731	-1.3583394683713500	1633
**RBPMS**	0.087343	-1.8454	-1.3572997336959300	1634
**AKR1D1**	0.087405	-5.0069	-1.3569094245259800	1636
**RCE1**	0.087405	-0.87913	-1.3569094245259800	1635
**CCDC127**	0.087526	-6.9399	-1.3561482869207400	1637
**SLC25A12**	0.087595	-3.9084	-1.355714601294440	1638
**SLC39A8**	0.087775	-3.6817	-1.354584446044140	1639
**ANKRD23**	0.087839	-3.0427	-1.3541830297204700	1641
**SCIN**	0.087839	-1.9368	-1.3541830297204700	1640
**FANCM**	0.087884	-2.7337	-1.3539009144668900	1642
**TBCCD1**	0.087916	-3.7862	-1.3537003647145600	1643
**ABCB7**	0.087967	-4.4592	-1.353380851035210	1644
**NKAPL**	0.088124	-1.314	-1.352398116409880	1645
**NSD1**	0.088178	-1.6236	-1.3520604074695300	1646
**MAGEL2**	0.088198	-3.5243	-1.351935369207300	1647
**TRIB3**	0.088229	-6.9276	-1.351741601662330	1648
**CHRFAM7A**	0.088355	-2.5296	-1.3509545522213600	1651
**COL6A5**	0.088355	-4.1631	-1.3509545522213600	1653
**RASAL1**	0.088355	-1.3663	-1.3509545522213600	1649
**TOX2**	0.088355	-4.694	-1.3509545522213600	1654
**UBE2O**	0.088355	-1.8486	-1.3509545522213600	1650
**UPF2**	0.088355	-3.7252	-1.3509545522213600	1652
**ZNF425**	0.088355	-4.7683	-1.3509545522213600	1655
**ADAM9**	0.088388	-0.68845	-1.3507485584581300	1657
**FGFR1**	0.088388	-0.75523	-1.3507485584581300	1658
**IFI27L1**	0.088388	-0.13674	-1.3507485584581300	1656
**TPH2**	0.088388	-2.989	-1.3507485584581300	1660
**ZNF701**	0.088388	-2.3931	-1.3507485584581300	1659
**CCL8**	0.088472	-4.3613	-1.350224469167430	1661

**PGC**	0.088712	-2.0507	-1.3487291110194600	1662
**INHA**	0.088849	-2.2069	-1.347876861162580	1663
**B4GALT5**	0.08892	-3.0906	-1.3474355691225900	1664
**FITM2**	0.088926	-0.58239	-1.347398288860110	1665
**HMGN3**	0.088926	-2.6744	-1.347398288860110	1667
**STYXL1**	0.088926	-1.1001	-1.347398288860110	1666
**ANO6**	0.088965	-3.944	-1.347156012786570	1668
**IDO2**	0.089119	-4.6012	-1.3462001046285	1669
**VPS9D1**	0.08931	-2.0739	-1.3450162365058200	1670
**SPATA20**	0.089339	-1.2464	-1.3448366516608200	1671
**HP1BP3**	0.089418	-4.4247	-1.344347657647310	1672
**LCN9**	0.089434	-1.4879	-1.3442486600219700	1673
**ATP6V1C1**	0.089504	-4.1758	-1.3438157002328400	1674
**H3F3A**	0.089678	-4.506	-1.3427405757732400	1676
**PLXDC1**	0.089678	-4.8058	-1.3427405757732400	1677
**SRI**	0.089678	-4.832	-1.3427405757732400	1678
**USF2**	0.089678	-2.9701	-1.3427405757732400	1675
**CLCN7**	0.089973	-3.4458	-1.3409213454680300	1682
**MRPS23**	0.089973	-3.1959	-1.3409213454680300	1681
**NCOA2**	0.089973	-0.75511	-1.3409213454680300	1679
**ZFAND2B**	0.089973	-2.5249	-1.3409213454680300	1680
**COL17A1**	0.090102	-6.8901	-1.3401272105048600	1683
**SHF**	0.09016	-3.4405	-1.339770432839720	1684
**AIFM3**	0.090174	-4.1804	-1.3396843396391900	1685
**PICK1**	0.090206	-2.9899	-1.3394875924592100	1686
**CDC7**	0.090208	-0.62586	-1.33947529748198	1688
**TMEM255A**	0.090208	-0.16377	-1.33947529748198	1687
**FAM86C1**	0.090434	-3.5432	-1.3380872671841600	1689
**ATP6V1H**	0.09048	-4.8646	-1.3378050633186200	1690
**DEFB4A**	0.090505	-1.5606	-1.3376517363282600	1691
**DGAT2L6**	0.090591	-0.82255	-1.3371245314847500	1692
**FFAR1**	0.090774	-1.7535	-1.3360039237336700	1693

**CRYGN**	0.090864	-2.2675	-1.335453420089360	1694
**CREB3**	0.09087	-4.9177	-1.3354167342341100	1695
**ADGRB2**	0.090894	-1.3188	-1.3352700087827200	1696
**POSTN**	0.090894	-3.1887	-1.3352700087827200	1697
**DEC1**	0.090942	-4.5577	-1.3349766440871400	1699
**RNGTT**	0.090942	-2.5931	-1.3349766440871400	1698
**STX8**	0.091075	-1.3676	-1.3341643790353200	1700
**COL4A5**	0.091104	-1.1092	-1.333987385460340	1701
**JARID2**	0.091104	-2.4997	-1.333987385460340	1702
**SLC38A11**	0.091104	-3.8066	-1.333987385460340	1704
**STOX1**	0.091104	-3.4006	-1.333987385460340	1703
**SETD1B**	0.091119	-2.9531	-1.3338958534549800	1705
**TRIM17**	0.091182	-3.9889	-1.3335115410135600	1706
**OGDHL**	0.091187	-3.7116	-1.3334810484611600	1707
**SSX4B**	0.091351	-4.8549	-1.3324815792383700	1708
**NEFM**	0.091383	-4.0742	-1.3322867159877000	1709
**NLRC4**	0.09152	-3.7376	-1.331453029051060	1710
**KCNE2**	0.091541	-6.8646	-1.331325319394670	1711
**TDRD1**	0.091547	-3.194	-1.331288834909360	1712
**CXCL1**	0.091609	-3.7449	-1.3309119322823300	1713
**ANKHD1-EI**	0.091676	-2.427	-1.3305048467170900	1714
**ZFAND4**	0.091676	-4.3232	-1.3305048467170900	1715
**UNK**	0.091734	-3.4049	-1.3301526222538900	1716
**SCAP**	0.091903	-1.746	-1.329127252832630	1717
**VIPR2**	0.091938	-3.3305	-1.3289150728136700	1718
**UBE2W**	0.091968	-1.4805	-1.328733251832590	1719
**REM2**	0.092138	-3.7978	-1.3277037616615900	1720
**PDLIM4**	0.092157	-4.1441	-1.3275887883912400	1722
**S1PR5**	0.092157	-3.2878	-1.3275887883912400	1721
**TOR1AIP2**	0.092157	-5.0294	-1.3275887883912400	1724
**UPK3A**	0.092157	-4.4151	-1.3275887883912400	1723
**EMILIN3**	0.092163	-4.0004	-1.3275524846884300	1725

**TACR3**	0.092184	-2.6186	-1.3274254355050900	1726
**TBC1D4**	0.092279	-4.2517	-1.3268509567297000	1727
**MYLK2**	0.092284	-2.2427	-1.3268207331329800	1728
**CERS3**	0.092289	-2.951	-1.326790510748230	1731
**KIF16B**	0.092289	-0.57071	-1.326790510748230	1729
**OTUB1**	0.092289	-1.9312	-1.326790510748230	1730
**SEC14L5**	0.092289	-3.7442	-1.326790510748230	1732
**ZNF275**	0.092468	-3.4413	-1.3257093466795900	1733
**SLTM**	0.092743	-1.7344	-1.3240513532749600	1734
**JCHAIN**	0.092862	-4.5382	-1.3233350213226000	1735
**TBC1D21**	0.09299	-1.3154	-1.3225652702611500	1736
**TMX4**	0.09299	-4.3065	-1.3225652702611500	1737
**OXCT2**	0.09311	-3.4191	-1.3218443396620900	1738
**ZNF329**	0.093233	-4.0413	-1.3211060980991400	1739
**CLEC1B**	0.093257	-2.8242	-1.320962134889990	1740
**IL5RA**	0.093364	-2.1794	-1.3203206317811500	1741
**DYNLT3**	0.09337	-3.7339	-1.3202846757298700	1742
**DIP2C**	0.093382	-2.5117	-1.3202127687476000	1744
**MRPS11**	0.093382	-1.7976	-1.3202127687476000	1743
**RBP4**	0.093382	-4.5536	-1.3202127687476000	1745
**OR8J1**	0.093416	-2.9206	-1.3200090693594300	1746
**FAM24B**	0.093564	-3.1448	-1.3191230153595200	1747
**EVC**	0.093686	-4.696	-1.3183933974327200	1749
**ZNF846**	0.093686	-3.1745	-1.3183933974327200	1748
**ZNF730**	0.093847	-4.3144	-1.3174316139066400	1750
**OR2AP1**	0.093869	-4.2032	-1.31730028467976	1751
**PLSCR5**	0.094043	-3.392	-1.316262389382480	1752
**ANXA10**	0.094049	-2.6632	-1.3162266251733400	1753
**ZBTB42**	0.094089	-2.4477	-1.3159882401247400	1754
**SLC35B2**	0.094109	-2.7366	-1.3158690756395000	1755
**ANKRD16**	0.094238	-5.0867	-1.3151009132542000	1756
**ITGAX**	0.094253	-2.9978	-1.3150116423971100	1760

**NRL**	0.094253	-2.6116	-1.3150116423971100	1759
**SLC6A7**	0.094253	-1.4699	-1.3150116423971100	1758
**TRAPPC13**	0.094253	-1.4593	-1.3150116423971100	1757
**NDC80**	0.094368	-3.3815	-1.3143275803769700	1761
**SERPINB6**	0.094421	-3.7441	-1.314012523951200	1762
**CASC4**	0.094431	-2.4505	-1.313953093967050	1763
**PSMC5**	0.094448	-4.4764	-1.3138520736432500	1764
**PYDC1**	0.094587	-4.7435	-1.3130265864673500	1765
**AP1S1**	0.094623	-1.3676	-1.3128129370432600	1766
**FPGT-TNNI3**	0.094647	-2.8952	-1.3126705373787300	1768
**LCLAT1**	0.094647	-2.4342	-1.3126705373787300	1767
**TSSC4**	0.094647	-3.3541	-1.3126705373787300	1769
**DLST**	0.09467	-3.0197	-1.3125340960064300	1770
**ADH6**	0.094923	-1.6063	-1.311034850817690	1771
**KCNRG**	0.094923	-5.0564	-1.311034850817690	1772
**CLEC3A**	0.09497	-1.9279	-1.3107566592915700	1773
**MRPS26**	0.094993	-0.4572	-1.3106205599719100	1774
**DDO**	0.095006	-2.7194	-1.310543645006930	1775
**GUCY2D**	0.095007	-6.7987	-1.3105377287922900	1776
**THAP4**	0.095053	-2.9491	-1.3102656324899400	1777
**IFT172**	0.095107	-3.6325	-1.3099463388214400	1778
**KLHL24**	0.095109	-4.8154	-1.3099345156945200	1779
**ECT2**	0.095178	-3.5493	-1.3095267298964600	1780
**ELAC2**	0.095236	-3.4305	-1.3091841218052200	1781
**OR52I2**	0.095263	-2.9677	-1.3090246842384900	1782
**TSPAN12**	0.095394	-3.5275	-1.3082515887490400	1783
**SCT**	0.095469	-5.0154	-1.3078093285791000	1784
**ARFGEF3**	0.09556	-4.1612	-1.3072730628143700	1785
**RRAS2**	0.095578	-1.587	-1.3071670327940000	1786
**AGPAT2**	0.095629	-2.5611	-1.30686669417995	1790
**LHX8**	0.095629	-0.74111	-1.30686669417995	1787
**MCM10**	0.095629	-0.81556	-1.30686669417995	1788

**STIP1**	0.095629	-3.2195	-1.30686669417995	1791
**WASF1**	0.095629	-2.0543	-1.30686669417995	1789
**C19orf84**	0.095967	-3.9552	-1.3048791874564900	1793
**DGCR6**	0.095967	-4.1246	-1.3048791874564900	1794
**IFNA10**	0.095967	-4.7335	-1.3048791874564900	1797
**MIDN**	0.095967	-4.4783	-1.3048791874564900	1796
**NPTX1**	0.095967	-4.3929	-1.3048791874564900	1795
**TMEM121**	0.095967	-2.6666	-1.3048791874564900	1792
**SUPT4H1**	0.095979	-3.6801	-1.3048087197949600	1798
**SLC7A11**	0.096256	-6.7714	-1.3031838889533300	1799
**GRINA**	0.096267	-0.49337	-1.3031194359851400	1800
**RALGAPA1**	0.096267	-1.0926	-1.3031194359851400	1801
**OR2C3**	0.096508	-0.947	-1.3017086864158800	1802
**RASL10B**	0.096576	-1.881	-1.3013111008842300	1803
**BPIFB4**	0.096621	-4.1734	-1.3010481058763100	1804
**ICE2**	0.096649	-3.0734	-1.3008845099441400	1805
**ZNF562**	0.09668	-1.4023	-1.3007034264786400	1806
**COL9A2**	0.096867	-2.3049	-1.2996119877958100	1807
**ADSL**	0.096915	-6.7576	-1.299332081979360	1808
**MYL10**	0.09692	-2.5158	-1.2993029309773300	1809
**DSCR4**	0.097013	-1.8165	-1.298760923475570	1811
**EOMES**	0.097013	-4.1012	-1.298760923475570	1813
**SPATS2**	0.097013	-1.8514	-1.298760923475570	1812
**TMEM116**	0.097013	-1.0598	-1.298760923475570	1810
**BPIFB1**	0.097082	-3.3864	-1.2983590352943700	1815
**GPR68**	0.097082	-2.4226	-1.2983590352943700	1814
**HTR4**	0.097138	-1.2878	-1.2980330192701700	1816
**ITLN1**	0.097157	-4.6377	-1.297922438030130	1818
**MRPL24**	0.097157	-4.7406	-1.297922438030130	1819
**PHB2**	0.097157	-5.0965	-1.297922438030130	1821
**PRKACB**	0.097157	-4.8491	-1.297922438030130	1820
**TAOK1**	0.097157	-2.7929	-1.297922438030130	1817

**ZNF658**	0.097203	-3.3102	-1.2976547807341900	1822
**ARL14EPL**	0.097272	-2.0907	-1.2972534690120900	1824
**EPS8L3**	0.097272	-2.086	-1.2972534690120900	1823
**CASQ2**	0.097409	-3.2356	-1.2964572803077400	1825
**NIPAL2**	0.097528	-3.185	-1.2957663666981400	1826
**MRPL47**	0.097529	-4.0613	-1.2957605633218800	1827
**HTT**	0.0976	-1.0632	-1.2953486350994400	1828
**FETUB**	0.09767	-1.5248	-1.29494272374872	1829
**NBPF6**	0.097743	-3.9716	-1.2945196433453000	1830
**ANKRD2**	0.097752	-3.2075	-1.2944674987903200	1831
**FMOD**	0.098058	-5.0596	-1.292696673988810	1832
**DEFB119**	0.098079	-1.8739	-1.2925752953728800	1833
**RNPEP**	0.098098	-2.8841	-1.2924654930318800	1834
**BAGE2**	0.09826	-4.8724	-1.2915299157701500	1835
**MOV10L1**	0.098277	-6.7296	-1.2914318034314500	1836
**OR2B11**	0.098296	-4.2969	-1.291322163173770	1838
**OR4L1**	0.098296	-2.9028	-1.291322163173770	1837
**OR5D16**	0.098296	-5.0079	-1.291322163173770	1840
**REEP3**	0.098296	-4.4877	-1.291322163173770	1839
**CERS6**	0.098307	-4.0499	-1.2912586943294600	1843
**TSPAN10**	0.098307	-2.3529	-1.2912586943294600	1842
**ZNF593**	0.098307	-0.64488	-1.2912586943294600	1841
**GOLGA8J**	0.098355	-3.3408	-1.2909818002241900	1844
**AIG1**	0.098385	-1.329	-1.2908087916595500	1845
**ZIK1**	0.098447	-3.2103	-1.2904513630079100	1846
**CASC3**	0.098667	-3.0307	-1.2891843962157400	1847
**ZNF253**	0.098864	-2.8049	-1.2880516386323200	1848
**GNAT1**	0.098983	-3.7209	-1.287368184001730	1851
**IL36A**	0.098983	-3.4736	-1.287368184001730	1850
**PTP4A1**	0.098983	-0.885	-1.287368184001730	1849
**TNFSF15**	0.099251	-2.843	-1.285831173354530	1852
**IARS**	0.099334	-1.4573	-1.2853557744358800	1853

**RAB4B**	0.099358	-1.1508	-1.2852183638194300	1854
**DNAH6**	0.099383	-3.9882	-1.285075253560730	1855
**DDI2**	0.099461	-2.6393	-1.2846289186229500	1856
**LIMCH1**	0.099461	-3.4401	-1.2846289186229500	1857
**TIFA**	0.099461	-4.5637	-1.2846289186229500	1858
**ARHGAP9**	0.099559	-4.6323	-1.284068501334790	1864
**CHST4**	0.099559	-2.3169	-1.284068501334790	1859
**FOXI3**	0.099559	-4.2314	-1.284068501334790	1861
**GLB1L3**	0.099559	-4.3997	-1.284068501334790	1863
**LINGO3**	0.099559	-4.2852	-1.284068501334790	1862
**NUFIP2**	0.099559	-2.6955	-1.284068501334790	1860
**C20orf197**	0.099596	-0.82753	-1.283857020054330	1866
**TIGD1**	0.099596	-0.63903	-1.283857020054330	1865
**ZNF367**	0.099596	-0.89995	-1.283857020054330	1867
**ZNF560**	0.099596	-4.4111	-1.283857020054330	1868
**CFAP99**	0.099803	-4.2604	-1.2826749258146400	1869
**DACT2**	0.099835	-3.4966	-1.2824923464992400	1870
**RASGRF2**	0.099882	-3.0912	-1.2822242606133100	1871
**CWC27**	0.099924	-5.7153	-1.281984772447780	1872
**SYAP1**	0.099938	-0.33868	-1.2819049593966400	1873
**CNR1**	0.10009	-1.8378	-1.2810389428011300	1874
**SPHAR**	0.10026	-4.9009	-1.280071508375330	1875
**CCDC140**	0.1003	-2.3689	-1.2798440507855600	1877
**OR1N1**	0.1003	-4.9538	-1.2798440507855600	1879
**RTL4**	0.1003	-2.0186	-1.2798440507855600	1876
**SNPH**	0.1003	-3.5617	-1.2798440507855600	1878
**PHLPP2**	0.10046	-2.4522	-1.2789348818835100	1880
**ZBTB5**	0.10046	-3.1418	-1.2789348818835100	1881
**CEP131**	0.10052	-1.6375	-1.2785942159478200	1882
**TRIM62**	0.10063	-1.8357	-1.2779700468318400	1883
**KRTAP9-8**	0.10065	-4.5501	-1.2778566150170500	1884
**GPR146**	0.10073	-3.3338	-1.277403052090730	1885

**ABCD2**	0.1009	-3.0685	-1.2764401024058300	1886
**GPT**	0.10096	-1.3981	-1.2761005202509800	1887
**ALPK1**	0.10097	-3.669	-1.2760439375301900	1889
**C7orf72**	0.10097	-4.4201	-1.2760439375301900	1891
**LTA**	0.10097	-4.7869	-1.2760439375301900	1892
**NSL1**	0.10097	-3.1436	-1.2760439375301900	1888
**RERGL**	0.10097	-4.0425	-1.2760439375301900	1890
**STAG2**	0.10102	-2.9227	-1.2757610851872000	1893
**IPO4**	0.10103	-0.041592	-1.2757045269654000	1894
**MRPL9**	0.10107	-0.25708	-1.2754783348752000	1896
**TAF2**	0.10107	-0.22513	-1.2754783348752000	1895
**MCCC2**	0.10119	-4.9342	-1.2748001498376200	1898
**PFKFB1**	0.10119	-1.9314	-1.2748001498376200	1897
**PDGFRL**	0.10121	-2.8263	-1.2746871759883500	1899
**RAB40AL**	0.10121	-3.7836	-1.2746871759883500	1900
**GLIS2**	0.10132	-3.1582	-1.2740661104187400	1901
**EMC2**	0.10136	-3.7907	-1.2738403901941000	1902
**MSR1**	0.10153	-3.5136	-1.2728818025013100	1903
**DHX29**	0.10163	-4.4282	-1.272318473331990	1904
**HTN1**	0.10167	-3.321	-1.2720932546776600	1905
**PNMA8B**	0.10176	-3.7607	-1.2715867484834600	1906
**OPRM1**	0.10178	-2.5451	-1.2714742358442200	1907
**BARD1**	0.10192	-4.0259	-1.2706870976476000	1908
**ANO2**	0.10203	-2.904	-1.2700691837883600	1911
**DHX16**	0.10203	-1.1607	-1.2700691837883600	1909
**WDCP**	0.10203	-2.3994	-1.2700691837883600	1910
**ARIH2**	0.10209	-1.8569	-1.2697323441623400	1912
**LYSMD1**	0.10215	-2.195	-1.2693956485397000	1913
**SMIM17**	0.10215	-3.0189	-1.2693956485397000	1914
**TMEM61**	0.10215	-3.5084	-1.2693956485397000	1915
**FAM43A**	0.1022	-3.1062	-1.2691151787404400	1916
**SDC3**	0.10235	-2.0105	-1.2682743677541600	1917

**ADSS**	0.10237	-4.4858	-1.2681623273499600	1919
**DCDC5**	0.10237	-3.9739	-1.2681623273499600	1918
**UBE2L3**	0.10243	-3.6102	-1.2678263016159200	1920
**PUSL1**	0.10256	-1.5337	-1.267098736535800	1921
**KIAA1324**	0.10263	-2.455	-1.2667072483919100	1923
**PPIL1**	0.10263	-3.6051	-1.2667072483919100	1924
**TNNI3K**	0.10263	-2.1341	-1.2667072483919100	1922
**BCAS1**	0.1029	-1.7975	-1.2651990381152500	1925
**PDCD11**	0.1029	-2.0979	-1.2651990381152500	1926
**STAG3**	0.10292	-3.0885	-1.2650874332801400	1927
**CCDC187**	0.10307	-3.4049	-1.2642508988124100	1928
**MLXIPL**	0.1031	-3.2439	-1.264083698050370	1929
**L3MBTL1**	0.10316	-2.4528	-1.2637494025007700	1930
**BRF2**	0.10322	-2.8477	-1.263415248119930	1932
**DNMT3L**	0.10322	-1.5703	-1.263415248119930	1931
**NDUFB9**	0.10326	-3.9804	-1.2631925568825000	1933
**IFNA8**	0.10334	-1.9223	-1.2627473622389200	1934
**CFAP45**	0.10336	-1.9506	-1.2626361026758200	1936
**KCNA10**	0.10336	-4.7883	-1.2626361026758200	1938
**SAA1**	0.10336	-3.2544	-1.2626361026758200	1937
**ZNF217**	0.10336	-0.50719	-1.2626361026758200	1935
**LECT2**	0.10341	-2.8623	-1.2623580221261500	1939
**COL20A1**	0.10351	-3.0443	-1.2618021536838600	1940
**ROMO1**	0.10351	-3.6427	-1.2618021536838600	1941
**BMP8B**	0.10366	-3.4255	-1.2609690813160400	1943
**PDP2**	0.10366	-3.9177	-1.2609690813160400	1944
**RWDD1**	0.10366	-4.2848	-1.2609690813160400	1945
**SGSM1**	0.10366	-4.6213	-1.2609690813160400	1946
**TMEM109**	0.10366	-2.5514	-1.2609690813160400	1942
**ZNF366**	0.10387	-3.8144	-1.259804248127720	1947
**CWC15**	0.10393	-6.627	-1.2594717524350000	1949
**FAM175B**	0.10393	-3.046	-1.2594717524350000	1948

**ZNF250**	0.10404	-2.6341	-1.2588625383706100	1950
**KLK14**	0.10408	-1.2185	-1.2586411217830700	1951
**PANO1**	0.10412	-0.24892	-1.2584197668835800	1952
**STKLD1**	0.10413	-1.1246	-1.2583644377921600	1953
**POP5**	0.10422	-3.2785	-1.257866649223530	1954
**CHERP**	0.10431	-0.6673	-1.257369172150890	1955
**LOC441155**	0.10431	-1.2811	-1.257369172150890	1956
**RTFDC1**	0.10431	-2.1382	-1.257369172150890	1958
**SUGCT**	0.10431	-1.9145	-1.257369172150890	1957
**LHFPL4**	0.10437	-3.4654	-1.2570376935692900	1959
**ZIC5**	0.10444	-1.8303	-1.2566511430434200	1960
**SLC2A5**	0.1045	-1.2317	-1.2563199634076800	1961
**OR1L4**	0.10459	-3.1483	-1.2558234521614900	1962
**ZNF280D**	0.1046	-3.954	-1.2557683033531100	1963
**OR4C3**	0.10467	-2.6118	-1.2553823685879100	1964
**THOC2**	0.1047	-2.1612	-1.255217025200340	1965
**PWP1**	0.10492	-0.68652	-1.2540055541329700	1966
**SCN3A**	0.10492	-0.83072	-1.2540055541329700	1967
**VPS37B**	0.10492	-3.0383	-1.2540055541329700	1968
**COL24A1**	0.10497	-4.1762	-1.2537304763494500	1972
**FRMPD3**	0.10497	-3.8364	-1.2537304763494500	1971
**HERPUD2**	0.10497	-4.2073	-1.2537304763494500	1973
**ITPKC**	0.10497	-4.4807	-1.2537304763494500	1975
**MOSPD3**	0.10497	-4.8682	-1.2537304763494500	1978
**RIIAD1**	0.10497	-3.4588	-1.2537304763494500	1969
**SLC43A2**	0.10497	-4.508	-1.2537304763494500	1976
**TIGD7**	0.10497	-4.7028	-1.2537304763494500	1977
**WASHC4**	0.10497	-3.7336	-1.2537304763494500	1970
**ZNF286A**	0.10497	-4.3423	-1.2537304763494500	1974
**APOBEC3B**	0.10505	-0.37779	-1.2532905491152500	1979
**LILRB4**	0.10523	-3.4239	-1.252301598661540	1980
**TRIM29**	0.10529	-2.1061	-1.2519722205440400	1981

**PEX3**	0.10553	-6.5963	-1.250656064303340	1982
**RBM48**	0.10562	-4.7445	-1.250163063842500	1983
**ADNP2**	0.10578	-3.7755	-1.2492873680510500	1984
**COX14**	0.10587	-1.9282	-1.2487952098845600	1988
**HNRNPAB**	0.10587	-0.65383	-1.2487952098845600	1986
**PPP2R3A**	0.10587	-0.55482	-1.2487952098845600	1985
**TTI2**	0.10587	-3.0184	-1.2487952098845600	1989
**UPK3B**	0.10587	-3.4391	-1.2487952098845600	1990
**ZNF335**	0.10587	-1.7044	-1.2487952098845600	1987
**FNIP2**	0.10594	-1.9817	-1.2484126292199800	1991
**ALX3**	0.10602	-2.7703	-1.2479756178025100	1996
**DNAJB3**	0.10602	-3.8383	-1.2479756178025100	1998
**DYRK1A**	0.10602	-1.3319	-1.2479756178025100	1993
**GSTA1**	0.10602	-2.994	-1.2479756178025100	1997
**PPP2R2A**	0.10602	-1.835	-1.2479756178025100	1994
**SAMM50**	0.10602	-2.2417	-1.2479756178025100	1995
**UBXN1**	0.10602	-0.903	-1.2479756178025100	1992
**WFDC11**	0.10602	-3.9721	-1.2479756178025100	1999
**CFAP61**	0.10677	-4.1315	-1.2438901740925600	2001
**HEG1**	0.10677	-2.4105	-1.2438901740925600	2000
**SLC35A1**	0.10677	-4.1494	-1.2438901740925600	2002
**CMSS1**	0.10678	-0.69426	-1.2438358416730600	2003
**GLDN**	0.10678	-2.7984	-1.2438358416730600	2004
**NPM1**	0.10678	-2.8345	-1.2438358416730600	2005
**SIRT6**	0.10684	-2.3867	-1.2435099242360100	2006
**GABRR2**	0.10687	-0.5385	-1.2433470150393700	2007
**TMEM86B**	0.10695	-2.9263	-1.242912751756290	2008
**PATZ1**	0.10696	-3.2534	-1.2428584853247200	2009
**DDX53**	0.10715	-2.7329	-1.241828117738570	2010
**PPP5D1**	0.10731	-4.8041	-1.240961461224880	2011
**C9orf152**	0.10735	-3.2478	-1.2407449426807200	2012
**PROSER2**	0.1074	-4.5147	-1.2404743762702700	2014

**RNF113A**	0.1074	-3.4239	-1.2404743762702700	2013
**PRAMEF18**	0.10747	-4.6503	-1.2400957357877700	2015
**OR52N2**	0.10758	-3.3609	-1.2395010882715000	2018
**PBOV1**	0.10758	-0.82129	-1.2395010882715000	2016
**ZNF140**	0.10758	-3.2389	-1.2395010882715000	2017
**KCNQ1**	0.10762	-3.751	-1.2392849614440300	2019
**FAM196B**	0.10765	-3.6963	-1.2391229043044900	2020
**VWA2**	0.10769	-2.0842	-1.2389068787268900	2021
**WDR59**	0.10772	-3.0989	-1.2387448974776000	2022
**BCL7B**	0.10794	-3.8566	-1.2375580267891300	2023
**KRT73**	0.10804	-0.98991	-1.237019115972340	2024
**C19orf35**	0.10812	-4.8533	-1.2365882458449400	2025
**SGCZ**	0.10817	-2.8575	-1.2363190685530200	2026
**EIF2AK2**	0.10827	-3.4603	-1.235780982538360	2027
**HOXA2**	0.10827	-4.5826	-1.235780982538360	2029
**PDZD11**	0.10827	-3.838	-1.235780982538360	2028
**AMACR**	0.10828	-4.6209	-1.2357271936134300	2034
**CCL25**	0.10828	-4.311	-1.2357271936134300	2032
**DPM3**	0.10828	-4.4735	-1.2357271936134300	2033
**GNPNAT1**	0.10828	-3.3327	-1.2357271936134300	2030
**NAPSA**	0.10828	-3.7494	-1.2357271936134300	2031
**ASAH2B**	0.10848	-1.4221	-1.2346521650324300	2038
**ATXN7L1**	0.10848	-1.1268	-1.2346521650324300	2036
**GPER1**	0.10848	-3.7111	-1.2346521650324300	2041
**PANK1**	0.10848	-1.2045	-1.2346521650324300	2037
**SHD**	0.10848	-2.6848	-1.2346521650324300	2040
**SMIM9**	0.10848	-1.575	-1.2346521650324300	2039
**WTAP**	0.10848	-0.67851	-1.2346521650324300	2035
**TRAPPC8**	0.10856	-2.2793	-1.2342225528752200	2042
**LITAF**	0.10862	-2.2724	-1.233900493186860	2043
**PHF14**	0.10877	-2.9332	-1.2330959033745400	2044
**ZNF175**	0.10892	-1.3668	-1.2322921110336500	2045

**TBL3**	0.10905	-0.24582	-1.2315961344491800	2046
**RPS19BP1**	0.10911	-1.2441	-1.2312751156051300	2047
**GPN2**	0.10924	-4.5193	-1.2305800097184000	2048
**GHDC**	0.10931	-0.98123	-1.2302059680853300	2049
**RHOF**	0.10931	-2.5683	-1.2302059680853300	2051
**SLC15A4**	0.10931	-1.7717	-1.2302059680853300	2050
**KIAA0895L**	0.10934	-2.4231	-1.2300457172044700	2052
**SLC25A19**	0.10935	-1.676	-1.2299923072631700	2053
**MGAT4A**	0.10937	-1.4302	-1.2298854979053600	2054
**ATG2A**	0.10939	-2.9253	-1.2297787025765300	2055
**CCDC74B**	0.10947	-4.7554	-1.22935166145298	2056
**SLC3A1**	0.10954	-1.1088	-1.228978184289550	2057
**C3orf33**	0.10974	-2.2904	-1.2279120501155300	2059
**GIPC2**	0.10974	-2.7845	-1.2279120501155300	2061
**LTB4R**	0.10974	-3.3528	-1.2279120501155300	2062
**MTSS1**	0.10974	-3.8287	-1.2279120501155300	2063
**OR52K2**	0.10974	-1.1176	-1.2279120501155300	2058
**PORCN**	0.10974	-4.6071	-1.2279120501155300	2065
**SAMD3**	0.10974	-4.3984	-1.2279120501155300	2064
**SENP6**	0.10974	-2.6892	-1.2279120501155300	2060
**LIN7C**	0.1098	-3.4048	-1.2275924818891800	2066
**OR2T2**	0.1098	-6.5	-1.2275924818891800	2067
**TNNI1**	0.10983	-2.8952	-1.2274327447781600	2068
**BID**	0.10988	-1.6173	-1.2271665858360400	2069
**GPR52**	0.10988	-1.7627	-1.2271665858360400	2070
**SYPL1**	0.10988	-2.7192	-1.2271665858360400	2071
**COL6A2**	0.11022	-1.4795	-1.2253590058762000	2072
**EIF2AK3**	0.11025	-1.1033	-1.225199705663660	2073
**BMF**	0.11027	-4.5225	-1.2250935227925500	2075
**NELFE**	0.11027	-4.5132	-1.2250935227925500	2074
**EED**	0.11032	-2.3492	-1.2248281260269200	2076
**AMELY**	0.1105	-3.2025	-1.2238734112288900	2077

**ENTPD1**	0.1105	-4.2813	-1.2238734112288900	2078
**NCBP2**	0.11053	-3.3885	-1.2237144005179500	2080
**SPATA31A3**	0.11053	-2.4345	-1.2237144005179500	2079
**SPINK6**	0.11053	-5.0229	-1.2237144005179500	2081
**KRT12**	0.11058	-6.4833	-1.223449451402590	2082
**UGT2B10**	0.11067	-1.8858	-1.222972759301860	2083
**MTRNR2L4**	0.11077	-4.6833	-1.2224434271385800	2084
**ATP1B3**	0.1108	-4.6627	-1.22228469426457	2086
**C10orf142**	0.1108	-4.1551	-1.22228469426457	2085
**TEX2**	0.11082	-0.15253	-1.2221788894555700	2087
**CEND1**	0.11085	-4.4589	-1.2220202078907900	2088
**PIEZO2**	0.11087	-2.0048	-1.2219144372731800	2089
**RFX5**	0.11089	-1.6942	-1.2218086803238800	2090
**CGB3**	0.11099	-3.8505	-1.221280100436950	2091
**NVL**	0.11106	-4.2432	-1.2209102974468500	2092
**ALDH1L1**	0.11116	-3.8216	-1.2203822969129100	2096
**COX20**	0.11116	-3.7451	-1.2203822969129100	2095
**DDX50**	0.11116	-4.8827	-1.2203822969129100	2098
**NTNG2**	0.11116	-3.0623	-1.2203822969129100	2094
**POU5F1B**	0.11116	-2.2021	-1.2203822969129100	2093
**QRFPR**	0.11116	-4.452	-1.2203822969129100	2097
**SULT2B1**	0.11125	-4.9198	-1.2199073871528800	2099
**MS4A4A**	0.11135	-3.7953	-1.2193800321278600	2100
**ATRN**	0.11143	-1.4311	-1.218958392140580	2101
**CDK5RAP1**	0.11144	-0.68601	-1.2189057023776200	2102
**DNAH2**	0.11144	-1.2808	-1.2189057023776200	2103
**GPRIN3**	0.11144	-2.6454	-1.2189057023776200	2105
**RUSC1-AS1**	0.11144	-2.3792	-1.2189057023776200	2104
**SIGLEC11**	0.11144	-2.6708	-1.2189057023776200	2106
**OR5AU1**	0.11148	-3.4407	-1.2186949771572200	2107
**PLEKHO1**	0.11154	-4.984	-1.2183789907569600	2110
**RAMP3**	0.11154	-4.0154	-1.2183789907569600	2109

**ZNF571**	0.11154	-3.6077	-1.2183789907569600	2108
**PLCG1**	0.1116	-1.2722	-1.2180631259618000	2111
**OVGP1**	0.11169	-3.366	-1.217589556505130	2112
**GAS7**	0.11179	-1.8592	-1.2170636882800300	2113
**PLEKHM3**	0.11186	-4.0864	-1.2166957806838800	2114
**MRAS**	0.1119	-2.7641	-1.2164856216924500	2115
**SPPL3**	0.11194	-4.5258	-1.2162755164155300	2116
**KCNS1**	0.11224	-3.2813	-1.2147014352885300	2117
**PIGF**	0.11231	-2.9495	-1.2143345824936400	2120
**SLC38A9**	0.11231	-1.4384	-1.2143345824936400	2118
**SPOCK2**	0.11231	-2.8651	-1.2143345824936400	2119
**CUL1**	0.11246	-4.2478	-1.213549019179830	2122
**GLTP**	0.11246	-2.1692	-1.213549019179830	2121
**C6**	0.11269	-3.9535	-1.212345941237450	2125
**CA3**	0.11269	-4.7469	-1.212345941237450	2129
**LMBRD2**	0.11269	-3.282	-1.212345941237450	2124
**MPHOSPH8**	0.11269	-4.5534	-1.212345941237450	2128
**NDUFB1**	0.11269	-4.9592	-1.212345941237450	2130
**PCDHGB7**	0.11269	-4.0578	-1.212345941237450	2126
**SMAD4**	0.11269	-4.1592	-1.212345941237450	2127
**TLL1**	0.11269	-1.9182	-1.212345941237450	2123
**XPO4**	0.11278	-2.8071	-1.2118756489522200	2131
**ORC6**	0.11285	-3.7899	-1.2115100512881200	2132
**EDN2**	0.11295	-1.5018	-1.210988049641920	2133
**EMC3**	0.11295	-4.2937	-1.210988049641920	2135
**RNASE9**	0.11295	-3.5225	-1.210988049641920	2134
**MGAM2**	0.11303	-4.7577	-1.2105706857849800	2136
**MTNR1B**	0.11312	-2.2569	-1.210101403362750	2137
**HDDC3**	0.11318	-1.2195	-1.2097886964087300	2138
**C6orf62**	0.11335	-3.8545	-1.2089033351657000	2139
**OR4F15**	0.1135	-3.5743	-1.2081229202636400	2140
**FAM227B**	0.11367	-0.26254	-1.2072393386087300	2141

**KHDC1**	0.11367	-2.5248	-1.2072393386087300	2143
**SEC22B**	0.11367	-1.95	-1.2072393386087300	2142
**STBD1**	0.11367	-2.802	-1.2072393386087300	2144
**IGSF8**	0.11368	-3.9538	-1.2071873925639000	2145
**AKAP7**	0.11407	-3.7713	-1.2051640320692300	2147
**COG6**	0.11407	-3.7388	-1.2051640320692300	2146
**DCDC2C**	0.11407	-4.9228	-1.2051640320692300	2149
**MALRD1**	0.11407	-4.4416	-1.2051640320692300	2148
**PCGF5**	0.11408	-1.6304	-1.2051122158881800	2150
**MTSS1L**	0.11433	-1.4335	-1.2038178614640300	2151
**IL27RA**	0.11444	-1.8642	-1.2032489839504800	2152
**ANTXR1**	0.11445	-4.2127	-1.2031972871193900	2153
**AMOT**	0.11464	-1.9544	-1.2022156576453700	2157
**CCDC180**	0.11464	-1.5903	-1.2022156576453700	2155
**CFAP97**	0.11464	-1.657	-1.2022156576453700	2156
**DCTN5**	0.11464	-4.7563	-1.2022156576453700	2159
**TMEM8B**	0.11464	-0.4308	-1.2022156576453700	2154
**ZBTB9**	0.11464	-2.3439	-1.2022156576453700	2158
**RCC1**	0.11469	-1.6366	-1.2019575265627800	2160
**WDFY2**	0.11471	-1.0814	-1.20185429655127	2161
**MT1X**	0.11474	-3.9206	-1.2016994755437100	2163
**ST3GAL6**	0.11474	-4.5905	-1.2016994755437100	2164
**TACR2**	0.11474	-3.2406	-1.2016994755437100	2162
**MAPK4**	0.11478	-3.4136	-1.2014930923292900	2165
**RNASET2**	0.11503	-2.5147	-1.2002043550649300	2166
**CLEC3B**	0.11534	-6.3969	-1.1986090840298200	2167
**ZBTB26**	0.11537	-2.7891	-1.1984548647835500	2168
**CACYBP**	0.11541	-3.7376	-1.1982492834500100	2171
**RHOT2**	0.11541	-2.4554	-1.1982492834500100	2169
**ZNF598**	0.11541	-3.0619	-1.1982492834500100	2170
**ZW10**	0.11541	-4.2754	-1.1982492834500100	2172
**TMEM136**	0.11548	-4.4211	-1.1978896379243800	2173

**TMEM204**	0.11558	-3.5669	-1.1973761272166500	2174
**FAM110A**	0.1156	-1.0287	-1.1972734629573400	2177
**NECAB1**	0.1156	-0.79236	-1.1972734629573400	2175
**PBX1**	0.1156	-2.2949	-1.1972734629573400	2178
**STRIP1**	0.1156	-0.84861	-1.1972734629573400	2176
**PPL**	0.11571	-4.0022	-1.1967090349594300	2179
**SCN4B**	0.11577	-1.157	-1.1964013257687500	2180
**PASD1**	0.11581	-3.3053	-1.1961962492260400	2181
**G6PD**	0.11583	-2.0121	-1.196093729817510	2182
**EN2**	0.11587	-0.4356	-1.1958887287053100	2183
**OXSR1**	0.1159	-3.8661	-1.1957350108465300	2184
**NEURL1**	0.11599	-4.3575	-1.195274026708320	2185
**MTERF4**	0.11602	-3.3257	-1.1951204217616400	2186
**OLFM3**	0.11606	-2.068	-1.1949156590195000	2187
**FCHSD1**	0.11612	-0.24295	-1.194608608810720	2188
**KCNH2**	0.11612	-4.2973	-1.194608608810720	2189
**TRIM59**	0.11615	-2.2529	-1.1944551259331000	2190
**CCDC134**	0.11618	-0.1632	-1.194301671188070	2191
**PPIG**	0.11618	-0.61188	-1.194301671188070	2192
**ZNF761**	0.11622	-1.3931	-1.19409710859912	2193
**ICAM5**	0.11624	-6.3789	-1.1939948460401200	2194
**RAB11A**	0.1163	-3.2324	-1.1936881332556100	2195
**SH3GL3**	0.11632	-3.4605	-1.1935859206112200	2196
**NLRP2B**	0.11634	-1.8908	-1.1934837204352000	2197
**PPHLN1**	0.1164	-3.7108	-1.193177194676280	2198
**AVPI1**	0.11641	-3.7929	-1.1931261179482400	2199
**SPX**	0.11653	-2.9159	-1.1925134398382200	2200
**MORC3**	0.11669	-2.7776	-1.19169723135779	2201
**RD3L**	0.11674	-3.7079	-1.1914423289597400	2202
**BET1**	0.11689	-1.0867	-1.1906780858557500	2203
**CGREF1**	0.11689	-3.0959	-1.1906780858557500	2208
**DBF4**	0.11689	-2.5775	-1.1906780858557500	2207

**EOGT**	0.11689	-1.3076	-1.1906780858557500	2204
**GTF3C5**	0.11689	-2.4148	-1.1906780858557500	2206
**SENP2**	0.11689	-2.411	-1.1906780858557500	2205
**EFCAB6**	0.11698	-3.0307	-1.1902198736085500	2209
**NIPA1**	0.11702	-1.1196	-1.190016303912810	2210
**ARPC4-TTL**	0.11711	-3.89	-1.1895584523124100	2215
**C1QTNF5**	0.11711	-2.1479	-1.1895584523124100	2214
**ESYT2**	0.11711	-1.5842	-1.1895584523124100	2212
**GNMT**	0.11711	-4.4505	-1.1895584523124100	2218
**GPRC5D**	0.11711	-4.1819	-1.1895584523124100	2217
**IL2RA**	0.11711	-1.0999	-1.1895584523124100	2211
**PPP3R2**	0.11711	-2.086	-1.1895584523124100	2213
**VPS26A**	0.11711	-4.0856	-1.1895584523124100	2216
**TBX18**	0.11723	-4.3816	-1.1889483711379500	2219
**LOC643802**	0.11727	-3.1762	-1.1887451090569800	2220
**GUCY1A2**	0.11736	-0.10033	-1.188287948853880	2221
**CXorf40B**	0.11741	-1.7299	-1.1880340782386300	2222
**PSG3**	0.11751	-1.9369	-1.1875265665836700	2223
**C10orf53**	0.11753	-1.8158	-1.1874251009507900	2224
**CA8**	0.11772	-3.5132	-1.1864617865166600	2225
**KIF11**	0.11782	-0.51585	-1.1859552208630800	2227
**MUSTN1**	0.11782	-2.4519	-1.1859552208630800	2230
**NOBOX**	0.11782	-2.3861	-1.1859552208630800	2229
**ZBTB47**	0.11782	-2.3034	-1.1859552208630800	2228
**ZNF676**	0.11782	-0.032549	-1.1859552208630800	2226
**RAB2B**	0.1179	-3.2706	-1.1855501873473700	2231
**NFYC**	0.11791	-2.6587	-1.1854995718317400	2232
**STX12**	0.11794	-2.0133	-1.1853477435047900	2233
**SLC4A4**	0.11799	-4.0696	-1.1850947569966500	2234
**TNPO1**	0.11804	-3.5876	-1.1848418463143700	2236
**WDR48**	0.11804	-3.3034	-1.1848418463143700	2235
**KIRREL**	0.11805	-1.8072	-1.1847912732716000	2237

**ZNF468**	0.11807	-3.6817	-1.1846901362757800	2238
**ABCF2**	0.11809	-3.9893	-1.184589011396340	2239
**BCKDK**	0.11812	-3.0956	-1.184437346786760	2241
**RAPGEFL1**	0.11812	-2.6022	-1.184437346786760	2240
**MED16**	0.11817	-3.9808	-1.1841846329635700	2242
**SPATA6L**	0.11844	-0.27771	-1.18282128320972	2243
**CHST8**	0.11854	-3.3558	-1.182316896396920	2244
**COPS7A**	0.11857	-4.8121	-1.1821656389940000	2245
**CCDC155**	0.11867	-3.977	-1.1816616428782300	2246
**CRELD2**	0.11868	-4.0662	-1.1816112597734200	2253
**DOK5**	0.11868	-1.6968	-1.1816112597734200	2250
**FIG4**	0.11868	-3.5392	-1.1816112597734200	2252
**KMT2D**	0.11868	-0.78324	-1.1816112597734200	2249
**MEX3C**	0.11868	-0.25661	-1.1816112597734200	2248
**MPV17L**	0.11868	-0.23585	-1.1816112597734200	2247
**PPFIA1**	0.11868	-2.3869	-1.1816112597734200	2251
**MMP13**	0.11875	-4.7327	-1.1812586619927000	2256
**NPLOC4**	0.11875	-2.8918	-1.1812586619927000	2254
**RIOK1**	0.11875	-4.4363	-1.1812586619927000	2255
**FKBP14**	0.11878	-2.1307	-1.181107593606250	2257
**FAM83D**	0.11883	-3.1848	-1.1808558728446100	2258
**BLVRA**	0.11896	-4.0377	-1.1802017487809300	2260
**SMAD1**	0.11896	-2.4017	-1.1802017487809300	2259
**A1BG**	0.11911	-1.3488	-1.1794476171874500	2261
**POU3F3**	0.11925	-3.0201	-1.1787443657105000	2262
**GALR2**	0.11952	-3.3484	-1.177389738770400	2263
**PF4**	0.11953	-4.148	-1.1773396088854400	2264
**BSPRY**	0.11962	-3.3127	-1.1768885729955800	2271
**CARNS1**	0.11962	-2.7153	-1.1768885729955800	2268
**CCDC12**	0.11962	-4.4063	-1.1768885729955800	2272
**NDST4**	0.11962	-0.23311	-1.1768885729955800	2265
**OR2T8**	0.11962	-2.531	-1.1768885729955800	2267

**PIK3R5**	0.11962	-0.66487	-1.1768885729955800	2266
**RSPH9**	0.11962	-2.8226	-1.1768885729955800	2269
**TMEM101**	0.11962	-2.9997	-1.1768885729955800	2270
**OR1F1**	0.11963	-1.0555	-1.176838472674100	2273
**GPR141**	0.11988	-2.5615	-1.1755869233711100	2274
**KNSTRN**	0.12015	-2.6563	-1.1742373151373600	2275
**NT5C**	0.12015	-4.7681	-1.1742373151373600	2278
**PFDN1**	0.12015	-4.8299	-1.1742373151373600	2279
**STK38**	0.12015	-3.2808	-1.1742373151373600	2276
**STX16**	0.12015	-4.7017	-1.1742373151373600	2277
**SCAMP5**	0.12027	-1.4098	-1.173638175246970	2280
**BMPR1A**	0.12033	-1.6489	-1.173338763228300	2281
**HOXA4**	0.12033	-2.9178	-1.173338763228300	2282
**NDUFAF6**	0.12038	-3.2168	-1.1730893335398700	2283
**ZNF70**	0.12042	-4.2635	-1.172889842324960	2284
**CD84**	0.12045	-4.8777	-1.1727402545401300	2285
**ZNF862**	0.12048	-6.2858	-1.172590692992510	2286
**LCE2A**	0.1205	-0.96471	-1.172490999864120	2287
**PRR15L**	0.12053	-3.0881	-1.1723414820173000	2288
**TTC13**	0.12055	-1.1913	-1.1722418180111800	2289
**TMEM100**	0.12061	-3.1329	-1.1719428958324200	2290
**ARRB2**	0.12069	-0.37431	-1.1715444957481500	2292
**SAMD7**	0.12069	-0.37296	-1.1715444957481500	2291
**JMJD8**	0.12078	-4.3588	-1.1710965178068300	2295
**MTNR1A**	0.12078	-4.0708	-1.1710965178068300	2293
**OR6K6**	0.12078	-4.3468	-1.1710965178068300	2294
**SF3B4**	0.12087	-3.3446	-1.170648774762730	2296
**METRN**	0.12095	-3.6511	-1.1702509778718700	2297
**SKA2**	0.12117	-4.3043	-1.1691579901218600	2298
**AKT1**	0.12139	-1.7122	-1.1680663972910000	2299
**LRRC32**	0.12153	-4.8148	-1.1673724713662300	2300
**C20orf85**	0.12164	-4.5949	-1.1668276379471600	2304

**CTXN1**	0.12164	-1.8965	-1.1668276379471600	2301
**CXorf49B**	0.12164	-2.8934	-1.1668276379471600	2302
**TMEM53**	0.12164	-3.3681	-1.1668276379471600	2303
**ANAPC2**	0.12165	-2.8572	-1.1667781248095800	2306
**ASB8**	0.12165	-4.5938	-1.1667781248095800	2310
**MORN3**	0.12165	-2.4571	-1.1667781248095800	2305
**OVOL2**	0.12165	-3.5762	-1.1667781248095800	2308
**SCTR**	0.12165	-4.1167	-1.1667781248095800	2309
**UBE2D2**	0.12165	-3.3771	-1.1667781248095800	2307
**FGA**	0.12182	-0.4759	-1.1659368387204400	2311
**TECPR2**	0.12201	-2.6419	-1.1649975532788500	2312
**RASL10A**	0.12206	-0.90741	-1.1647505436681400	2313
**RHPN1**	0.12216	-4.6612	-1.1642567375267100	2314
**GMFB**	0.1222	-2.916	-1.1640592945407300	2315
**CBX3**	0.12223	-1.303	-1.1639112420761700	2316
**LASP1**	0.12228	-1.3728	-1.1636645446470000	2317
**NTF4**	0.12232	-3.5349	-1.1634672376822000	2318
**OR10T2**	0.12232	-3.6503	-1.1634672376822000	2319
**BZW1**	0.12246	-1.6963	-1.1627770197254600	2320
**CLDN3**	0.12258	-3.6023	-1.1621858449235500	2321
**CNP**	0.12288	-0.75966	-1.1607096820213600	2323
**F8A1**	0.12288	-0.56229	-1.1607096820213600	2322
**PHPT1**	0.12288	-2.0003	-1.1607096820213600	2324
**PIEZO1**	0.12297	-2.4579	-1.1602673260503700	2325
**KCNB2**	0.12313	-4.4125	-1.159481475534960	2326
**METTL7A**	0.12316	-1.2537	-1.1593342082611700	2327
**RUFY1**	0.12323	-4.1167	-1.1589906823644500	2328
**GRB10**	0.1233	-3.5255	-1.158647293185740	2330
**INTS6**	0.1233	-2.7666	-1.158647293185740	2329
**TCTEX1D4**	0.1233	-4.6961	-1.158647293185740	2332
**ZNF706**	0.1233	-3.73	-1.158647293185740	2331
**SP140**	0.12338	-3.9615	-1.1582550156284400	2333

**IFITM1**	0.12341	-1.654	-1.158107957486490	2334
**SLC9B2**	0.12341	-3.0185	-1.158107957486490	2335
**TUBA1C**	0.12341	-3.7888	-1.158107957486490	2336
**TM9SF3**	0.12345	-3.9667	-1.1579119189146500	2337
**KRTAP10-1**	0.1235	-0.25686	-1.1576669332602700	2338
**SPOCD1**	0.12363	-3.1913	-1.1570302955017500	2339
**FHDC1**	0.12377	-3.3528	-1.1563452096450700	2340
**ZNF302**	0.12387	-1.5442	-1.1558561946889600	2341
**PARK7**	0.12391	-3.279	-1.155660666080550	2342
**NBPF7**	0.12398	-3.7202	-1.1553185972895400	2343
**PITHD1**	0.12402	-3.5355	-1.1551231900896900	2344
**EDEM2**	0.12408	-2.9899	-1.1548301619636100	2345
**PSG11**	0.12419	-1.0534	-1.1542932011193400	2346
**RIT1**	0.12421	-4.0438	-1.1541956076262200	2347
**FAM222A**	0.12426	-1.1375	-1.1539516719754400	2348
**SFPQ**	0.12438	-3.4527	-1.1533665063883400	2349
**EAF1**	0.12454	-0.49537	-1.1525868994065500	2351
**NARS2**	0.12454	-0.24119	-1.1525868994065500	2350
**SMIM29**	0.12455	-2.6681	-1.1525381972269400	2352
**CIDEA**	0.12458	-4.4729	-1.1523921070877500	2353
**CIZ1**	0.12477	-0.3379	-1.1514674401172900	2354
**CALB2**	0.1249	-1.682	-1.1508353400690400	2355
**CBR4**	0.12497	-2.0502	-1.1504951688813200	2357
**CEP192**	0.12497	-3.7084	-1.1504951688813200	2361
**COLEC10**	0.12497	-1.857	-1.1504951688813200	2356
**DNAJB8**	0.12497	-2.5235	-1.1504951688813200	2359
**MID2**	0.12497	-2.1917	-1.1504951688813200	2358
**SLC2A12**	0.12497	-2.8248	-1.1504951688813200	2360
**WDR19**	0.12497	-4.1573	-1.1504951688813200	2362
**COLGALT2**	0.12502	-1.7427	-1.1502522709468400	2363
**SAP30L**	0.12504	-0.99484	-1.1501551307726200	2364
**BIRC3**	0.12517	-0.35491	-1.1495239840057800	2365

**MECR**	0.1253	-3.7167	-1.1488932948152100	2366
**GYPA**	0.12538	-3.2198	-1.1485054054286500	2369
**KRTAP21-3**	0.12538	-2.498	-1.1485054054286500	2368
**LYVE1**	0.12538	-2.272	-1.1485054054286500	2367
**PPP1R9B**	0.12538	-3.3692	-1.1485054054286500	2370
**MDM1**	0.12547	-3.1509	-1.1480692363185000	2371
**KCNK10**	0.12554	-0.96712	-1.147730144618380	2372
**RHBDF1**	0.12555	-2.6963	-1.1476817137190800	2373
**HNRNPC**	0.12558	-2.7124	-1.1475364371702700	2374
**ATP5A1**	0.12575	-3.5642	-1.1467136604615100	2376
**NDUFA9**	0.12575	-1.6078	-1.1467136604615100	2375
**COL6A3**	0.12578	-3.9722	-1.146568545136050	2378
**CXCR4**	0.12578	-4.3236	-1.146568545136050	2380
**MFN2**	0.12578	-3.847	-1.146568545136050	2377
**TRIM45**	0.12578	-4.1177	-1.146568545136050	2379
**HLA-DRA**	0.12579	-3.2563	-1.1465201787260900	2381
**CD3D**	0.12582	-0.3888	-1.1463750955860700	2382
**RMI1**	0.12584	-2.9671	-1.1462783868968300	2383
**SMIM8**	0.12589	-1.3635	-1.1460366620641400	2384
**PDE8B**	0.12613	-4.4032	-1.1448773138448800	2385
**ABCD1**	0.12622	-2.481	-1.1444429547650300	2388
**BANP**	0.12622	-3.6353	-1.1444429547650300	2390
**CLHC1**	0.12622	-2.9824	-1.1444429547650300	2389
**RPS4Y1**	0.12622	-1.6783	-1.1444429547650300	2387
**ZNF792**	0.12622	-1.2591	-1.1444429547650300	2386
**LDHAL6B**	0.12625	-3.3717	-1.1442982163779600	2391
**TWSG1**	0.12629	-2.7564	-1.144105269143750	2392
**ZNF138**	0.12629	-3.9861	-1.144105269143750	2393
**SAP130**	0.12641	-6.1654	-1.1435266828415100	2394
**COX7B**	0.1265	-0.86836	-1.1430929942054500	2396
**RANBP3L**	0.1265	-0.59465	-1.1430929942054500	2395
**PCDHGB1**	0.12654	-2.7292	-1.1429003126960100	2397

**NUDT3**	0.12661	-3.8538	-1.142563222117190	2398
**KPRP**	0.12668	-4.6172	-1.1422262613178100	2400
**TIGD3**	0.12668	-2.0777	-1.1422262613178100	2399
**ICE1**	0.12674	-2.8011	-1.141937540964620	2401
**IGSF1**	0.12681	-2.512	-1.1416008208179100	2402
**TBC1D2**	0.12691	-3.2011	-1.1411200164468400	2403
**FEN1**	0.12698	-0.12461	-1.1407836102833100	2404
**GFER**	0.12698	-1.608	-1.1407836102833100	2405
**MBTPS2**	0.12698	-3.144	-1.1407836102833100	2407
**PROS1**	0.12698	-2.1991	-1.1407836102833100	2406
**TTLL1**	0.12704	-2.8712	-1.1404953648630600	2408
**EEF1B2**	0.12706	-3.4192	-1.1403993041110800	2409
**NOTCH3**	0.12709	-3.3839	-1.140255232710980	2410
**EIF4G1**	0.12717	-2.3294	-1.1398711579715700	2411
**ARHGAP1**	0.1273	-3.4684	-1.1392473948968200	2412
**CIB1**	0.12762	-3.5687	-1.137713863388820	2414
**SERPIND1**	0.12762	-2.8377	-1.137713863388820	2413
**BTBD10**	0.12767	-4.1706	-1.137474490688470	2415
**SLC16A9**	0.12775	-1.8154	-1.1370916298775200	2416
**EID2B**	0.12777	-3.0756	-1.1369959407146600	2420
**LTB4R2**	0.12777	-2.2878	-1.1369959407146600	2418
**RPL38**	0.12777	-2.8392	-1.1369959407146600	2419
**WDR66**	0.12777	-1.2159	-1.1369959407146600	2417
**YJEFN3**	0.12779	-4.5623	-1.1369002619614700	2421
**DRG2**	0.12784	-3.6837	-1.1366611106001700	2423
**STK32C**	0.12784	-3.0723	-1.1366611106001700	2422
**ZMPSTE24**	0.12784	-4.0922	-1.1366611106001700	2424
**GTF2A1L**	0.12789	-4.2099	-1.1364220242306400	2425
**ISLR**	0.12804	-1.2761	-1.1357051545831300	2426
**CHST5**	0.12809	-1.0997	-1.1354663276914400	2427
**PSTPIP1**	0.12823	-4.5497	-1.1347979566956700	2428
**KRTCAP3**	0.12827	-3.3288	-1.1346070866408300	2429

**BRD1**	0.12842	-3.4732	-1.1338916917949600	2430
**SLC10A3**	0.12849	-2.4201	-1.1335580393816200	2431
**ALB**	0.12855	-3.0986	-1.133272152004370	2436
**ANKS1A**	0.12855	-0.96608	-1.133272152004370	2433
**INSIG1**	0.12855	-2.8501	-1.133272152004370	2435
**IPO9**	0.12855	-1.0394	-1.133272152004370	2434
**OR5B21**	0.12855	-3.5711	-1.133272152004370	2437
**ZNF785**	0.12855	-0.55413	-1.133272152004370	2432
**DMD**	0.12866	-3.0596	-1.1327482655733100	2438
**FSCB**	0.12868	-1.1909	-1.1326530468915900	2439
**IMPDH1**	0.12871	-2.6363	-1.132510238120910	2440
**MIOX**	0.12885	-1.8257	-1.1318441023648500	2441
**TMEM106A**	0.12924	-3.903	-1.129991081477810	2442
**KLRC3**	0.12927	-2.9969	-1.1298487020725600	2443
**SYT2**	0.1293	-4.6759	-1.129706345567770	2444
**AGBL2**	0.12939	-0.14287	-1.1292794133537600	2445
**ADAM15**	0.12941	-4.4659	-1.1291845674775500	2448
**OR6B2**	0.12941	-0.60258	-1.1291845674775500	2446
**TYMS**	0.12941	-2.8769	-1.1291845674775500	2447
**ZBTB41**	0.12941	-4.5975	-1.1291845674775500	2449
**BLMH**	0.12948	-1.0501	-1.128852686875410	2450
**ABHD14A-A**	0.1295	-1.1947	-1.1287578866807600	2451
**CNOT6**	0.1295	-3.5867	-1.1287578866807600	2453
**MGLL**	0.1295	-2.3436	-1.1287578866807600	2452
**DNAI2**	0.12956	-3.3198	-1.1284735469435700	2454
**BCAN**	0.12967	-3.9791	-1.1279524942507100	2455
**LRRC38**	0.12978	-2.4058	-1.1274317476122200	2456
**COMMD9**	0.13	-1.0611	-1.1263911704980300	2457
**CFAP126**	0.13006	-4.7596	-1.1261075883350700	2458
**ALMS1**	0.13011	-0.98268	-1.125871339025200	2459
**FAM9B**	0.1302	-3.9487	-1.125446248548390	2460
**ICA1L**	0.1302	-4.2029	-1.125446248548390	2461

**PAXIP1**	0.13027	-1.8716	-1.1251157631701800	2462
**KRTAP8-1**	0.13028	-1.6166	-1.125068561004020	2463
**CEL**	0.13031	-2.8457	-1.1249269695434700	2464
**BSX**	0.13036	-6.0792	-1.1246910338800000	2467
**CHRM2**	0.13036	-1.291	-1.1246910338800000	2465
**ZNF148**	0.13036	-4.3297	-1.1246910338800000	2466
**CYP26B1**	0.13047	-4.4743	-1.1241721956718900	2468
**MUC2**	0.13053	-1.5114	-1.12388932052021	2469
**SCRN1**	0.13054	-1.1809	-1.1238421834043300	2470
**SALL4**	0.1306	-3.2155	-1.1235594131318500	2471
**DCUN1D1**	0.13061	-0.87176	-1.1235122934867900	2472
**HVCN1**	0.13061	-3.3685	-1.1235122934867900	2479
**KIAA0825**	0.13061	-3.2339	-1.1235122934867900	2477
**LRRC4B**	0.13061	-2.5684	-1.1235122934867900	2475
**MAGED1**	0.13061	-2.9214	-1.1235122934867900	2476
**MFRP**	0.13061	-3.2952	-1.1235122934867900	2478
**NRBP1**	0.13061	-2.2757	-1.1235122934867900	2474
**PDGFRB**	0.13061	-2.2547	-1.1235122934867900	2473
**WDR5B**	0.13061	-3.5712	-1.1235122934867900	2480
**CCDC138**	0.1307	-2.5259	-1.123088328883080	2481
**GAA**	0.13077	-0.031861	-1.1227587181478100	2482
**LY6L**	0.13077	-0.44546	-1.1227587181478100	2483
**ANKRD34A**	0.13092	-3.7411	-1.1220528198649700	2484
**ZNF554**	0.13095	-1.6966	-1.1219117072880500	2485
**FBN3**	0.13111	-1.2762	-1.1211594838806700	2486
**FUT6**	0.13111	-1.7798	-1.1211594838806700	2488
**GAPT**	0.13111	-4.4013	-1.1211594838806700	2489
**KIF13A**	0.13111	-1.7592	-1.1211594838806700	2487
**SAA2-SAA4**	0.13111	-4.6257	-1.1211594838806700	2490
**C4orf32**	0.13117	-1.6137	-1.120877563600630	2491
**DAO**	0.1312	-3.7596	-1.1207366368623000	2492
**LYPD4**	0.13124	-2.9746	-1.1205487691612200	2493

**KIAA0408**	0.13138	-4.5826	-1.1198915434369900	2494
**GNB3**	0.1314	-2.8013	-1.1197976935302900	2495
**PIK3R1**	0.13162	-3.5529	-1.1187659948058600	2496
**NUCKS1**	0.13172	-4.4729	-1.1182974342753700	2497
**LOC100134**	0.13178	-1.0373	-1.118016415768380	2498
**TMEM185A**	0.13178	-4.2677	-1.118016415768380	2499
**C22orf46**	0.13185	-0.75809	-1.1176886723933900	2502
**FUT2**	0.13185	-2.4887	-1.1176886723933900	2505
**GRIA4**	0.13185	-0.60905	-1.1176886723933900	2501
**IPO11**	0.13185	-2.4458	-1.1176886723933900	2504
**KRTAP10-4**	0.13185	-0.25588	-1.1176886723933900	2500
**MTRNR2L1**	0.13185	-0.88783	-1.1176886723933900	2503
**KIAA1107**	0.1321	-4.3406	-1.1165191391797800	2508
**KIF19**	0.1321	-3.5623	-1.1165191391797800	2506
**OR56B1**	0.1321	-3.9682	-1.1165191391797800	2507
**BLK**	0.13211	-1.465	-1.1164723896123400	2509
**HCLS1**	0.13218	-3.2097	-1.1161452109387300	2510
**TTC38**	0.13219	-1.4358	-1.1160984808808600	2511
**HLA-DPB1**	0.13225	-2.5861	-1.1158181517001500	2512
**YIF1A**	0.13228	-3.7091	-1.1156780199865100	2513
**CENPP**	0.13231	-2.9895	-1.1155379101778900	2514
**C20orf196**	0.1327	-4.5906	-1.1137184716635000	2517
**CCDC144A**	0.1327	-3.7617	-1.1137184716635000	2515
**TAF3**	0.1327	-4.0027	-1.1137184716635000	2516
**IFNA5**	0.13272	-1.5872	-1.113625266482830	2518
**FAM20A**	0.13284	-1.3027	-1.1130662384391300	2519
**RPL3**	0.13284	-2.7677	-1.1130662384391300	2520
**NCK1**	0.13292	-1.2119	-1.1126937462420900	2521
**SH3BP1**	0.13312	-3.1292	-1.1117631905186900	2522
**SLC30A3**	0.13312	-3.8576	-1.1117631905186900	2523
**EIF2D**	0.13329	-1.7128	-1.1109729744142100	2524
**LXN**	0.13329	-3.3608	-1.1109729744142100	2525

**SHROOM3**	0.13338	-1.0746	-1.1105549054294900	2526
**MAST4**	0.13341	-4.0749	-1.110415592228060	2527
**TFAM**	0.13341	-4.458	-1.110415592228060	2528
**USP19**	0.13353	-1.4385	-1.1098585548064700	2529
**RNF167**	0.13357	-1.1388	-1.1096729521767400	2530
**NOP53**	0.13392	-1.5332	-1.1080505569891700	2531
**CPSF7**	0.13394	-0.96683	-1.1079579367667500	2532
**MAP1LC3C**	0.13396	-4.6505	-1.1078653260479800	2533
**CCT8L2**	0.13399	-3.9632	-1.107726427783130	2539
**KRT6A**	0.13399	-2.7475	-1.107726427783130	2536
**MICU3**	0.13399	-2.2884	-1.107726427783130	2535
**OR2T29**	0.13399	-4.1387	-1.107726427783130	2540
**PDK1**	0.13399	-3.791	-1.107726427783130	2538
**RBBP9**	0.13399	-3.1991	-1.107726427783130	2537
**RTN3**	0.13399	-2.1014	-1.107726427783130	2534
**CC2D1A**	0.13403	-1.9323	-1.107541263333800	2541
**DTL**	0.13413	-1.6475	-1.1070785182603000	2542
**SEPT11**	0.13419	-3.0238	-1.106800984969480	2543
**CHRNA4**	0.13421	-4.4916	-1.1067084928150200	2544
**RGS18**	0.13426	-1.132	-1.1064773038396700	2545
**GOLGA6L2**	0.13442	-1.8602	-1.1057378962234100	2546
**EBF2**	0.13451	-4.2138	-1.1053222449750500	2547
**IVNS1ABP**	0.13467	-2.7694	-1.1045837805586600	2548
**BPIFB6**	0.13472	-0.20798	-1.104353133924110	2549
**CELF5**	0.13472	-3.1923	-1.104353133924110	2552
**EIF4G2**	0.13472	-2.6597	-1.104353133924110	2551
**MAP3K3**	0.13472	-0.51651	-1.104353133924110	2550
**PRDM16**	0.13474	-1.0284	-1.1042608917182300	2553
**MRPS31**	0.13485	-1.1579	-1.1037537274451900	2554
**PEX16**	0.13487	-1.4971	-1.1036615462571500	2555
**TXNL4A**	0.13487	-1.8753	-1.1036615462571500	2556
**LACTBL1**	0.13488	-3.1647	-1.1036154591797700	2557

**EVI5**	0.13489	-3.8646	-1.1035693744463700	2558
**PIP5K1B**	0.13505	-1.4385	-1.102832337218840	2559
**NR2F6**	0.13513	-3.6937	-1.1024640431650000	2560
**SOGA3**	0.1352	-0.99771	-1.1021419084923700	2561
**BFSP2**	0.13559	-2.8175	-1.1003492479115900	2563
**ELMOD2**	0.13559	-2.6611	-1.1003492479115900	2562
**EYS**	0.13559	-3.8136	-1.1003492479115900	2564
**AGBL4**	0.13561	-3.9529	-1.1002574118990400	2570
**GPR174**	0.13561	-3.5183	-1.1002574118990400	2569
**HIST1H2BO**	0.13561	-2.3595	-1.1002574118990400	2565
**IL31**	0.13561	-2.7808	-1.1002574118990400	2566
**JPH1**	0.13561	-3.2931	-1.1002574118990400	2568
**OR5D18**	0.13561	-4.0779	-1.1002574118990400	2571
**PLAC8L1**	0.13561	-4.4842	-1.1002574118990400	2572
**ULK1**	0.13561	-3.132	-1.1002574118990400	2567
**SAMD9L**	0.13573	-3.6278	-1.0997065905787600	2573
**MYF5**	0.13579	-1.4884	-1.099431304999240	2574
**PROCA1**	0.13584	-3.7995	-1.0992019639784900	2575
**ZNF208**	0.13597	-3.8116	-1.0986059477277800	2576
**MLLT1**	0.13605	-1.5055	-1.0982393623926800	2577
**DSG4**	0.13608	-1.4168	-1.0981019309353800	2578
**SMC1A**	0.1362	-0.33278	-1.097552412389170	2579
**RIPK3**	0.13628	-3.4559	-1.0971862507424200	2580
**CENPJ**	0.13629	-5.9394	-1.097140490879130	2581
**HSPA1B**	0.1363	-3.3616	-1.09709473331309	2582
**KLF4**	0.13645	-1.7639	-1.0964086452714800	2583
**CACNA1H**	0.13649	-2.1003	-1.096225775603330	2584
**BIRC7**	0.13656	-1.4955	-1.0959058418650600	2585
**THPO**	0.13678	-0.90464	-1.0949010653341300	2586
**PCDH19**	0.13679	-4.8314	-1.0948554199350900	2587
**CDA**	0.13681	-2.9457	-1.094764135979750	2588
**C10orf55**	0.13688	-3.2667	-1.0944447139508100	2589

**EVPL**	0.1369	-1.9586	-1.0943534710218200	2591
**KLHL28**	0.1369	-0.87171	-1.0943534710218200	2590
**MLIP**	0.1369	-3.8444	-1.0943534710218200	2592
**AHSA2**	0.13719	-4.6565	-1.0930314709712500	2597
**GNB2**	0.13719	-2.563	-1.0930314709712500	2594
**MAGEA10-M**	0.13719	-3.7814	-1.0930314709712500	2595
**MAPKAPK3**	0.13719	-2.4264	-1.0930314709712500	2593
**MPPE1**	0.13719	-4.3283	-1.0930314709712500	2596
**BET1L**	0.1372	-1.2535	-1.0929859188297600	2598
**SF3B3**	0.13733	-5.9147	-1.0923939472461500	2599
**MNAT1**	0.13748	-3.2669	-1.0917113784372700	2600
**OR4X1**	0.13771	-2.585	-1.0906657596928000	2601
**DMBT1**	0.13772	-2.5784	-1.090620325055110	2602
**EDAR**	0.13782	-1.3117	-1.0901661024453000	2603
**MYOD1**	0.13795	-4.6815	-1.0895759490955700	2604
**ABHD12B**	0.13814	-2.5969	-1.0887140992582200	2607
**BCL9**	0.13814	-2.7372	-1.0887140992582200	2608
**MRPL12**	0.13814	-3.2817	-1.0887140992582200	2609
**MYBPC2**	0.13814	-1.696	-1.0887140992582200	2606
**SCAF11**	0.13814	-1.1355	-1.0887140992582200	2605
**TMIE**	0.13826	-0.47095	-1.0881701894514400	2610
**NXT2**	0.13832	-0.49736	-1.0878983552310900	2611
**LRIG3**	0.13834	-2.8413	-1.0878077616865700	2612
**BHMT**	0.13836	-0.10103	-1.0877171770690000	2613
**ZMYND8**	0.13865	-3.3077	-1.08640470173654	2614
**ATP6V0A2**	0.13887	-3.9763	-1.0854102777858100	2616
**IL36RN**	0.13887	-4.3572	-1.0854102777858100	2619
**KCNJ18**	0.13887	-4.0608	-1.0854102777858100	2617
**RBKS**	0.13887	-4.3563	-1.0854102777858100	2618
**TSC22D2**	0.13887	-2.1371	-1.0854102777858100	2615
**TTC23**	0.13887	-4.5314	-1.0854102777858100	2620
**RAI1**	0.13896	-3.4109	-1.0850037772022500	2621

**EIF2B2**	0.13903	-3.1684	-1.0846877339913300	2622
**ADAT2**	0.1392	-2.334	-1.0839206511718300	2626
**AKR1B15**	0.1392	-2.8203	-1.0839206511718300	2628
**ASB5**	0.1392	-3.3386	-1.0839206511718300	2629
**CAT**	0.1392	-2.7605	-1.0839206511718300	2627
**CD226**	0.1392	-1.7967	-1.0839206511718300	2624
**OR52N5**	0.1392	-0.87176	-1.0839206511718300	2623
**ROPN1B**	0.1392	-1.8873	-1.0839206511718300	2625
**TNFRSF1B**	0.1392	-3.516	-1.0839206511718300	2630
**C1orf105**	0.13926	-1.1411	-1.0836500682867900	2631
**CDH11**	0.13937	-2.0377	-1.0831542056182300	2632
**CUZD1**	0.1394	-1.1263	-1.0830190165590900	2633
**FOXD2**	0.1395	-0.72664	-1.0825685292464200	2635
**PPIAL4F**	0.1395	-0.60056	-1.0825685292464200	2634
**TUBB1**	0.13957	-3.4023	-1.0822533188005100	2636
**TNPO3**	0.1396	-0.27025	-1.082118261521620	2637
**KCNE3**	0.13973	-1.5529	-1.0815332412614700	2638
**LGR5**	0.13985	-3.1125	-1.0809935509054	2639
**OR6P1**	0.13991	-1.5153	-1.080723823762270	2640
**ENDOV**	0.14024	-3.3199	-1.0792417277453200	2641
**CMTR2**	0.14028	-2.7278	-1.0790622407936200	2642
**BCL9L**	0.14038	-2.9759	-1.0786136754290700	2644
**C7orf55-LU**	0.14038	-4.342	-1.0786136754290700	2651
**CCR8**	0.14038	-3.9764	-1.0786136754290700	2650
**HLA-DQB2**	0.14038	-3.3581	-1.0786136754290700	2646
**HYI**	0.14038	-3.6535	-1.0786136754290700	2647
**MMP26**	0.14038	-3.7836	-1.0786136754290700	2649
**OR52E8**	0.14038	-1.8201	-1.0786136754290700	2643
**PLXNB2**	0.14038	-3.733	-1.0786136754290700	2648
**PON2**	0.14038	-3.1229	-1.0786136754290700	2645
**SLC12A4**	0.14038	-4.4307	-1.0786136754290700	2652
**ALX1**	0.14048	-3.3655	-1.0781653269881600	2659

**C5orf58**	0.14048	-3.0317	-1.0781653269881600	2658
**CCDC129**	0.14048	-2.0261	-1.0781653269881600	2656
**CD300LG**	0.14048	-0.31636	-1.0781653269881600	2654
**CDR2**	0.14048	-1.5653	-1.0781653269881600	2655
**RAB44**	0.14048	-2.6019	-1.0781653269881600	2657
**SLC2A8**	0.14048	-0.062646	-1.0781653269881600	2653
**DVL2**	0.14053	-1.5273	-1.0779412340203800	2660
**LRIT2**	0.14097	-3.234	-1.075971545313140	2661
**DECR1**	0.141	-1.9929	-1.0758374003326500	2662
**CCDC159**	0.14102	-1.3304	-1.0757479810999000	2663
**ASAH1**	0.14111	-0.54319	-1.0753457009510900	2665
**TM4SF18**	0.14111	-0.12739	-1.0753457009510900	2664
**SLC41A3**	0.14114	-1.859	-1.0752116462336900	2666
**RUNX1T1**	0.14116	-3.5102	-1.0751222871556200	2667
**NME4**	0.14124	-2.8087	-1.0747649366603700	2670
**OR13D1**	0.14124	-1.5554	-1.0747649366603700	2668
**PRG4**	0.14124	-2.6384	-1.0747649366603700	2669
**OS9**	0.14128	-2.3618	-1.0745863128699200	2671
**FOXI1**	0.14139	-3.0914	-1.0740952741438300	2673
**SOX5**	0.14139	-0.099453	-1.0740952741438300	2672
**FZD2**	0.14149	-0.46753	-1.0736490999323900	2674
**LOC101928**	0.14184	-0.99871	-1.072089170609030	2675
**PITX2**	0.14184	-2.5312	-1.072089170609030	2677
**PRPF19**	0.14184	-1.8646	-1.072089170609030	2676
**SETX**	0.14184	-4.0153	-1.072089170609030	2679
**TMED4**	0.14184	-2.5689	-1.072089170609030	2678
**TMEM255B**	0.14188	-1.5894	-1.0719110590407800	2680
**C11orf1**	0.14197	-3.8824	-1.071510432290840	2682
**C19orf12**	0.14197	-1.8899	-1.071510432290840	2681
**NCAPG**	0.14197	-4.3267	-1.071510432290840	2683
**ATXN7**	0.14208	-3.0323	-1.0710210108046700	2684
**LUC7L3**	0.14217	-2.7207	-1.0706207657792900	2685

**FHOD3**	0.14224	-4.0907	-1.0703095826270700	2688
**KIAA1644**	0.14224	-4.1538	-1.0703095826270700	2689
**RFESD**	0.14224	-4.6269	-1.0703095826270700	2690
**TRPS1**	0.14224	-4.0834	-1.0703095826270700	2687
**ZDHHC13**	0.14224	-2.4377	-1.0703095826270700	2686
**KLHL22**	0.14225	-3.8367	-1.070265136351200	2691
**BOLA2**	0.14234	-3.5476	-1.0698652149716700	2692
**CSRNP3**	0.14261	-0.56431	-1.0686664762199300	2694
**DLX1**	0.14261	-0.42665	-1.0686664762199300	2693
**MS4A13**	0.14261	-0.57271	-1.0686664762199300	2695
**SSB**	0.14263	-1.2275	-1.0685777418367800	2696
**DTX3L**	0.14267	-2.8614	-1.0684002983070300	2697
**PCYOX1L**	0.1427	-1.1022	-1.0682672377326800	2698
**ZNF710**	0.14289	-2.0686	-1.0674249596003900	2699
**PARG**	0.14311	-2.6937	-1.0664506352628500	2700
**GTPBP3**	0.14353	-4.3699	-1.0645933673326900	2701
**RGS17**	0.14365	-2.5508	-1.0640633934295300	2702
**POLD3**	0.14372	-1.7336	-1.0637543799571900	2703
**HLA-E**	0.14381	-4.5769	-1.0633572261149200	2706
**HTR2B**	0.14381	-4.4798	-1.0633572261149200	2705
**SGPP1**	0.14381	-0.89926	-1.0633572261149200	2704
**ABCC3**	0.14386	-0.071718	-1.0631366575474200	2707
**CCL19**	0.14386	-2.2286	-1.0631366575474200	2709
**CUL7**	0.14386	-2.3977	-1.0631366575474200	2710
**DCXR**	0.14386	-3.1427	-1.0631366575474200	2712
**HIST1H2AD**	0.14386	-2.446	-1.0631366575474200	2711
**ITM2B**	0.14386	-2.0464	-1.0631366575474200	2708
**SUPT6H**	0.14386	-3.5237	-1.0631366575474200	2713
**SUSD2**	0.14388	-2.109	-1.0630484446011600	2714
**COX6C**	0.14403	-4.4412	-1.0623871109991300	2719
**LPAR6**	0.14403	-4.1276	-1.0623871109991300	2717
**RIMBP3C**	0.14403	-4.2343	-1.0623871109991300	2718

**RPF2**	0.14403	-2.8238	-1.0623871109991300	2716
**SHOC2**	0.14403	-1.816	-1.0623871109991300	2715
**SLC39A3**	0.14405	-2.3069	-1.0622989682881100	2720
**CNTN5**	0.14415	-1.0434	-1.0618583784739200	2721
**MZB1**	0.14421	-4.1845	-1.0615941234979300	2722
**PI4KA**	0.14421	-5.729	-1.0615941234979300	2723
**ZNF646**	0.14439	-2.2656	-1.060801802995960	2724
**RASA2**	0.14443	-2.1906	-1.0606258221906000	2725
**UIMC1**	0.14457	-3.0935	-1.0600101478755400	2726
**DNASE2B**	0.14462	-3.4803	-1.059790361542780	2727
**EFHD2**	0.14485	-1.4887	-1.0587800031080000	2728
**TRAF6**	0.14493	-3.4998	-1.0584288273373600	2729
**KRTAP3-1**	0.14497	-3.4899	-1.0582532883913500	2730
**PMPCB**	0.14503	-1.3732	-1.0579900410972000	2731
**BCORL1**	0.14504	-3.5203	-1.0579461736759000	2732
**BBS2**	0.14506	-3.3849	-1.0578584449403000	2738
**CPNE9**	0.14506	-0.026317	-1.0578584449403000	2733
**EEA1**	0.14506	-2.8717	-1.0578584449403000	2736
**PGR**	0.14506	-3.1235	-1.0578584449403000	2737
**RFC4**	0.14506	-0.86581	-1.0578584449403000	2734
**RSPH1**	0.14506	-1.3893	-1.0578584449403000	2735
**RYR1**	0.14517	-3.1978	-1.0573760823539000	2739
**ACADSB**	0.14523	-5.7004	-1.0571130791546600	2740
**ABR**	0.14545	-3.7507	-1.0561493590620900	2742
**CCL20**	0.14545	-3.2779	-1.0561493590620900	2741
**SPANXN3**	0.14545	-4.3148	-1.0561493590620900	2743
**TBC1D9B**	0.14554	-0.7828	-1.0557553925291100	2744
**MUC19**	0.14564	-0.093974	-1.055317844033710	2746
**PLOD1**	0.14564	-0.085661	-1.055317844033710	2745
**HS6ST2**	0.14578	-3.824	-1.0547056153485000	2747
**CLNS1A**	0.14586	-4.797	-1.0543559478128300	2755
**FOCAD**	0.14586	-4.027	-1.0543559478128300	2752

**HBA2**	0.14586	-2.5457	-1.0543559478128300	2749
**KRTAP7-1**	0.14586	-3.5796	-1.0543559478128300	2751
**LRRC69**	0.14586	-4.5034	-1.0543559478128300	2754
**PRPF3**	0.14586	-2.9668	-1.0543559478128300	2750
**RS1**	0.14586	-1.8645	-1.0543559478128300	2748
**ZNF772**	0.14586	-4.1703	-1.0543559478128300	2753
**CYP4Z1**	0.14591	-1.3681	-1.0541374710513800	2756
**C17orf98**	0.14599	-2.9889	-1.0537880128525600	2757
**C4orf3**	0.14612	-3.5542	-1.0532204175880700	2758
**RSG1**	0.14614	-3.2671	-1.0531331253526600	2760
**SAMD10**	0.14614	-2.14	-1.0531331253526600	2759
**LCK**	0.14619	-1.1977	-1.0529149298647100	2761
**MIF**	0.14619	-2.828	-1.0529149298647100	2762
**PPARA**	0.14619	-3.1867	-1.0529149298647100	2763
**MINDY3**	0.14622	-1.921	-1.0527840366302200	2764
**TST**	0.14622	-5.6713	-1.0527840366302200	2765
**PIWIL1**	0.14634	-3.9343	-1.0522606439689700	2766
**FOXP2**	0.14637	-1.6846	-1.0521298408368300	2767
**KLRF1**	0.1464	-3.739	-1.0519990557035700	2768
**ABCB6**	0.14642	-3.0556	-1.0519118756105500	2769
**PGBD1**	0.1465	-2.2965	-1.0515632351589400	2770
**SCG2**	0.14658	-1.6815	-1.0512147224780300	2771
**ZFP92**	0.14665	-1.0477	-1.050909878587280	2772
**NKD2**	0.14679	-1.0147	-1.0503004836033300	2773
**RAB3GAP2**	0.14682	-3.1867	-1.050169949701350	2774
**OR4C45**	0.14685	-3.5208	-1.050039433690850	2775
**HIST1H4K**	0.14686	-3.0622	-1.0499959323286700	2776
**CAPZB**	0.14688	-1.6408	-1.049908935564700	2777
**BICDL2**	0.14697	-1.525	-1.0495175484211300	2778
**SCRIB**	0.14697	-2.4139	-1.0495175484211300	2779
**BRD4**	0.14727	-3.4829	-1.0482140841655100	2782
**OR6K2**	0.14727	-3.342	-1.0482140841655100	2781

**ZNF546**	0.14727	-1.0083	-1.0482140841655100	2780
**CENPN**	0.14729	-5.6415	-1.0481272498623500	2786
**GFRA2**	0.14729	-5.6415	-1.0481272498623500	2787
**MTHFS**	0.14729	-2.2048	-1.0481272498623500	2784
**SMARCB1**	0.14729	-5.6415	-1.0481272498623500	2785
**TMEM25**	0.14729	-2.0844	-1.0481272498623500	2783
**GRID1**	0.14737	-1.3088	-1.0477799916523600	2788
**FKBP2**	0.14742	-1.898	-1.0475630194182900	2789
**PARL**	0.14742	-3.1534	-1.0475630194182900	2791
**RAB3IL1**	0.14742	-3.4863	-1.0475630194182900	2792
**UGT2B4**	0.14742	-2.2674	-1.0475630194182900	2790
**PELO**	0.14749	-2.7856	-1.0472593411153600	2793
**MAP3K19**	0.14757	-3.3213	-1.0469123984145200	2794
**GSR**	0.1476	-0.14746	-1.0467823273855400	2795
**PPP6R2**	0.14762	-4.3221	-1.0466956232041600	2796
**RAD51D**	0.14787	-0.51298	-1.045612484121850	2797
**SNX22**	0.14787	-3.6035	-1.045612484121850	2799
**ZBTB20**	0.14787	-2.2835	-1.045612484121850	2798
**SLC19A3**	0.14797	-4.0558	-1.0451795718030300	2800
**NPRL3**	0.14801	-4.4662	-1.0450064617115800	2801
**IFNB1**	0.14813	-3.5548	-1.0444873192319000	2803
**OR8K3**	0.14813	-1.0216	-1.0444873192319000	2802
**SEMA4A**	0.14818	-3.4161	-1.0442710929269700	2804
**OR10G8**	0.14823	-2.7268	-1.0440549154346200	2805
**SYNE2**	0.14828	-0.43797	-1.0438387867227100	2806
**ZNF93**	0.14831	-2.0838	-1.0437091328968300	2807
**ATP1B1**	0.14858	-4.4238	-1.0425430370451300	2808
**COL6A1**	0.14873	-3.7008	-1.0418958181491100	2809
**CA4**	0.14874	-1.1471	-1.0418526857396700	2810
**GALNT3**	0.14874	-2.8386	-1.0418526857396700	2814
**PTHLH**	0.14874	-2.7851	-1.0418526857396700	2813
**RNF222**	0.14874	-1.8136	-1.0418526857396700	2811

**SPRED3**	0.14874	-2.242	-1.0418526857396700	2812
**HPF1**	0.14896	-0.36231	-1.0409042626411200	2815
**ATP2A1**	0.14897	-3.8771	-1.040861174745510	2822
**CRYZL1**	0.14897	-3.5994	-1.040861174745510	2817
**FAM162A**	0.14897	-3.7303	-1.040861174745510	2818
**GPATCH11**	0.14897	-3.7912	-1.040861174745510	2819
**GRIN2C**	0.14897	-3.8896	-1.040861174745510	2823
**NIFK**	0.14897	-3.7923	-1.040861174745510	2820
**OR5T2**	0.14897	-3.4656	-1.040861174745510	2816
**TGM6**	0.14897	-3.8264	-1.040861174745510	2821
**USP17L29**	0.14898	-2.9638	-1.0408180887822400	2824
**LDHC**	0.14914	-3.5318	-1.0401289759614800	2826
**RDH5**	0.14914	-2.4275	-1.0401289759614800	2825
**IL13**	0.14924	-1.9811	-1.0396985311594400	2827
**PRKAG3**	0.14926	-3.007	-1.0396124653133100	2828
**MLH1**	0.1494	-5.5801	-1.0390102198791400	2829
**RNF130**	0.14946	-2.8655	-1.0387522300227800	2830
**TMEM178A**	0.14954	-1.842	-1.0384083510574400	2831
**POU3F1**	0.14955	-3.0971	-1.038365374820310	2832
**EMC9**	0.14968	-1.4167	-1.0378068581597300	2834
**ITIH3**	0.14968	-0.3937	-1.0378068581597300	2833
**LRRC29**	0.14968	-2.777	-1.0378068581597300	2837
**NARR**	0.14968	-2.0272	-1.0378068581597300	2836
**SCGN**	0.14968	-1.5433	-1.0378068581597300	2835
**MZF1**	0.14973	-2.9144	-1.0375921302409200	2838
**SYCE2**	0.14976	-3.0273	-1.0374633164503400	2839
**TMEM128**	0.14977	-1.6518	-1.0374203823454400	2840
**KDM8**	0.14982	-3.4552	-1.037205740499360	2841
**PRAMEF8**	0.14984	-4.7251	-1.037119897139550	2843
**SLC15A5**	0.14984	-4.3151	-1.037119897139550	2842
**CPNE6**	0.14992	-2.2777	-1.0367766000996800	2844
**CLIP2**	0.14999	-1.1414	-1.0364763153899600	2845

**PITPNA**	0.15005	-1.7556	-1.036219002862450	2846
**GGN**	0.15013	-2.5745	-1.0358760261778200	2848
**TMEM239**	0.15013	-0.060642	-1.0358760261778200	2847
**ARHGAP27**	0.15025	-2.6001	-1.0353617895046000	2849
**CDX1**	0.15025	-4.4107	-1.0353617895046000	2854
**CIB3**	0.15025	-3.8699	-1.0353617895046000	2851
**DHRS9**	0.15025	-3.0695	-1.0353617895046000	2850
**DIRAS2**	0.15025	-4.2106	-1.0353617895046000	2853
**TMPRSS11E**	0.15025	-4.1355	-1.0353617895046000	2852
**ICAM1**	0.15046	-2.1184	-1.034462533526590	2855
**SLC5A10**	0.15055	-5.5483	-1.0340773940826700	2856
**SLC40A1**	0.15057	-2.7299	-1.0339918283679200	2857
**ASB3**	0.15073	-3.0177	-1.0333075749935500	2860
**EFCAB14**	0.15073	-4.2867	-1.0333075749935500	2861
**FAM19A2**	0.15073	-2.2843	-1.0333075749935500	2859
**ZNF728**	0.15073	-1.99	-1.0333075749935500	2858
**CD19**	0.15076	-2.004	-1.0331793313382500	2864
**EFCAB1**	0.15076	-2.6996	-1.0331793313382500	2865
**FKBP5**	0.15076	-2.9614	-1.0331793313382500	2866
**MBD6**	0.15076	-0.054771	-1.0331793313382500	2862
**PCDH15**	0.15076	-0.76646	-1.0331793313382500	2863
**ZDHHC8**	0.15117	-3.4988	-1.0314283680171400	2867
**GPD1**	0.15127	-3.1754	-1.0310017832630000	2868
**IL10**	0.1513	-2.9427	-1.0308738444167900	2871
**KCNJ14**	0.1513	-0.69186	-1.0308738444167900	2869
**MYO7B**	0.1513	-1.7346	-1.0308738444167900	2870
**GUCA2A**	0.15132	-3.0617	-1.0307885612260900	2872
**NRG2**	0.1514	-2.9723	-1.0304475034087900	2873
**IGFBP4**	0.15143	-1.0951	-1.0303196376250000	2874
**PIK3C2G**	0.15154	-1.2039	-1.0298509404705400	2875
**MFF**	0.15156	-3.1456	-1.0297657471099500	2876
**TM4SF5**	0.15166	-0.76717	-1.029339892367600	2877

**OCLM**	0.15168	-3.1984	-1.0292547438177600	2878
**HS3ST3A1**	0.15173	-3.2698	-1.029041905083980	2879
**SLC1A6**	0.15179	-1.1392	-1.0287865601202800	2880
**HTRA3**	0.15188	-3.0107	-1.0284036683965200	2881
**C10orf128**	0.15202	-2.3801	-1.0278083585449300	2882
**OR4D6**	0.84796	-0.014444	-1.027723344012110	2883
**MRAP2**	0.15215	-1.0046	-1.0272558967912400	2884
**ARMC10**	0.15224	-1.0474	-1.0268736068637200	2885
**IFI6**	0.15224	-4.4175	-1.0268736068637200	2886
**ADPRHL1**	0.15229	-3.5247	-1.0266612884026200	2887
**CCDC142**	0.15232	-3.4378	-1.0265339195378700	2888
**ANKRD22**	0.15237	-2.7123	-1.0263216750958600	2891
**CASP6**	0.15237	-3.49	-1.0263216750958600	2892
**GALNT16**	0.15237	-1.2508	-1.0263216750958600	2889
**TMEM51**	0.15237	-2.0844	-1.0263216750958600	2890
**GCC2**	0.15262	-1.8874	-1.0252611456422600	2893
**DGKE**	0.15267	-3.4604	-1.0250491780954600	2894
**BTNL3**	0.15271	-3.6231	-1.0248796372123000	2900
**CNDP1**	0.15271	-4.5392	-1.0248796372123000	2903
**FRMD6**	0.15271	-3.3846	-1.0248796372123000	2899
**IFNW1**	0.15271	-3.6918	-1.0248796372123000	2901
**OTOP3**	0.15271	-2.0583	-1.0248796372123000	2896
**PSG8**	0.15271	-3.0492	-1.0248796372123000	2898
**SH3PXD2A**	0.15271	-2.2725	-1.0248796372123000	2897
**SLC31A2**	0.15271	-4.2762	-1.0248796372123000	2902
**ZNF586**	0.15271	-1.9332	-1.0248796372123000	2895
**MAP1B**	0.15277	-2.1304	-1.0246253811092700	2904
**FBXL21**	0.15287	-3.6522	-1.024201768069690	2905
**SLC6A18**	0.15291	-0.89924	-1.024032374306120	2906
**TRAPPC2L**	0.15296	-1.0649	-1.0238206734137100	2907
**RPP14**	0.1531	-2.5048	-1.0232281548754300	2908
**ACTR1A**	0.15326	-2.6517	-1.022551430404470	2911

**ADRA1A**	0.15326	-2.8836	-1.022551430404470	2912
**CD209**	0.15326	-3.0504	-1.022551430404470	2914
**DDB1**	0.15326	-2.4179	-1.022551430404470	2909
**RIMBP3B**	0.15326	-3.0449	-1.022551430404470	2913
**SPAG4**	0.15326	-2.5795	-1.022551430404470	2910
**LRP4**	0.1533	-1.9567	-1.022382322442740	2915
**OR5K2**	0.15353	-3.2757	-1.0214105184971600	2916
**KIAA0141**	0.15356	-1.3449	-1.0212838325665000	2917
**MBLAC2**	0.15368	-3.332	-1.0207772526689300	2918
**MGAT5**	0.15381	-2.6463	-1.0202287532232900	2919
**SUDS3**	0.15394	-2.7056	-1.0196805605431200	2920
**URGCP-MR**	0.1541	-0.97368	-1.01900628223149	2921
**NOMO1**	0.15418	-3.4355	-1.0186693167497300	2922
**ACTN1**	0.15422	-3.7406	-1.0185008773758500	2925
**MILR1**	0.15422	-0.54734	-1.0185008773758500	2923
**MOCS1**	0.15422	-4.221	-1.0185008773758500	2926
**SGTB**	0.15422	-1.1981	-1.0185008773758500	2924
**MXRA5**	0.15423	-1.8688	-1.018458772047290	2927
**CLDN23**	0.15424	-1.3818	-1.0184166685242300	2928
**RFX8**	0.15426	-2.7535	-1.0183324668937000	2929
**ACSM3**	0.15429	-1.5103	-1.0182061779834600	2930
**RASSF5**	0.15448	-3.0692	-1.01740672502594	2932
**RIPK4**	0.15448	-0.91259	-1.01740672502594	2931
**MYO5C**	0.15454	-3.8783	-1.0171544012550500	2938
**NKX3-1**	0.15454	-2.6618	-1.0171544012550500	2935
**PCBD1**	0.15454	-3.6483	-1.0171544012550500	2937
**RALGPS2**	0.15454	-3.4839	-1.0171544012550500	2936
**TRABD2A**	0.15454	-2.3604	-1.0171544012550500	2934
**UBA2**	0.15454	-1.9507	-1.0171544012550500	2933
**ATAD3B**	0.1546	-2.0282	-1.0169021422269200	2943
**CKMT1A**	0.1546	-3.054	-1.0169021422269200	2946
**GBP6**	0.1546	-0.93069	-1.0169021422269200	2940

**HIGD1B**	0.1546	-1.9035	-1.0169021422269200	2942
**MOAP1**	0.1546	-1.7743	-1.0169021422269200	2941
**ORC5**	0.1546	-2.2733	-1.0169021422269200	2944
**RAD51AP2**	0.1546	-0.55094	-1.0169021422269200	2939
**SF3A3**	0.1546	-2.6193	-1.0169021422269200	2945
**CLEC4D**	0.15465	-1.3317	-1.0166919757908800	2947
**UBE3D**	0.15477	-1.0751	-1.0161877594722700	2948
**CADM2**	0.15483	-2.1969	-1.015935748168870	2949
**ACTR10**	0.15493	-2.2041	-1.0155158726504900	2950
**NAA35**	0.15495	-3.4329	-1.0154319190285800	2951
**C1orf54**	0.15498	-0.11594	-1.0153060020133200	2953
**OXT**	0.15498	-0.050307	-1.0153060020133200	2952
**MS4A3**	0.15502	-1.1339	-1.0151381376963300	2954
**PNPO**	0.15504	-3.3718	-1.0150542162637800	2955
**CASZ1**	0.1551	-2.4567	-1.0148024948481500	2956
**RBM12**	0.15515	-1.8089	-1.0145927761105500	2957
**KIAA0930**	0.15516	-2.7192	-1.0145508377176300	2958
**TMSB4X**	0.15525	-1.2832	-1.0141734724498000	2959
**WNT9B**	0.15532	-2.5578	-1.0138800659483900	2960
**C16orf78**	0.15534	-2.331	-1.0137962515494400	2961
**C2orf27A**	0.15551	-4.4876	-1.013084116493620	2966
**FJX1**	0.15551	-2.8577	-1.013084116493620	2963
**HSD11B2**	0.15551	-0.84834	-1.013084116493620	2962
**LOC340178**	0.15551	-3.3242	-1.013084116493620	2964
**TMEM222**	0.15551	-3.4063	-1.013084116493620	2965
**SLC17A5**	0.15552	-2.9385	-1.0130422421952400	2967
**APOBEC3D**	0.15566	-1.4855	-1.0124561884220400	2968
**CYYR1**	0.15593	-2.4974	-1.0113269227432600	2971
**FAT4**	0.15593	-2.9885	-1.0113269227432600	2974
**GPRC6A**	0.15593	-2.7424	-1.0113269227432600	2973
**MYO1B**	0.15593	-2.6519	-1.0113269227432600	2972
**NDRG4**	0.15593	-1.3536	-1.0113269227432600	2970

**OR4D5**	0.15593	-0.73747	-1.0113269227432600	2969
**RPEL1**	0.15595	-3.2218	-1.0112433247332600	2975
**LYZL2**	0.15607	-2.8364	-1.0107418850095900	2976
**USP16**	0.15609	-0.71567	-1.0106583364284400	2977
**CBX4**	0.1562	-1.6975	-1.0101989452776700	2978
**PITX3**	0.15626	-1.9658	-1.0099484581214100	2979
**C1orf21**	0.84373	-0.17684	-1.0099067164225300	2980
**MTMR3**	0.1563	-0.87892	-1.0097815018823500	2981
**CGA**	0.15633	-2.2894	-1.0096563031721900	2982
**DNAJC27**	0.15647	-2.8184	-1.0090722516576700	2983
**EARS2**	0.15651	-0.8407	-1.0089054430078900	2984
**SLC26A11**	0.84346	-0.07659	-1.0087803549410600	2985
**GAB1**	0.15659	-0.28109	-1.0085719098987400	2986
**KRTAP1-5**	0.15662	-3.8323	-1.008446863905110	2987
**BICRAL**	0.1567	-4.3653	-1.0081134849870200	2996
**EMCN**	0.1567	-4.2005	-1.0081134849870200	2995
**LHB**	0.1567	-2.5768	-1.0081134849870200	2990
**PANK3**	0.1567	-4.122	-1.0081134849870200	2994
**PGP**	0.1567	-3.4101	-1.0081134849870200	2992
**PRM3**	0.1567	-3.4072	-1.0081134849870200	2991
**RSPH14**	0.1567	-2.2236	-1.0081134849870200	2988
**SUCLG1**	0.1567	-2.5652	-1.0081134849870200	2989
**WT1**	0.1567	-3.9197	-1.0081134849870200	2993
**HSD17B10**	0.15674	-0.92624	-1.0079468375370400	2997
**CCND2**	0.15677	-3.7697	-1.0078218703170100	2998
**CD52**	0.15679	-3.2794	-1.0077385675801000	3002
**CUL5**	0.15679	-1.7265	-1.0077385675801000	3001
**FBXL14**	0.15679	-0.76425	-1.0077385675801000	2999
**PTOV1**	0.15679	-1.1494	-1.0077385675801000	3000
**RCN3**	0.15691	-2.8891	-1.0072388979388600	3003
**KAT8**	0.84305	-0.029443	-1.0070723972660300	3004
**POLR2A**	0.15706	-2.6135	-1.0066146643044000	3005

**CISD1**	0.15736	-1.7776	-1.005367372296120	3006
**SLC39A9**	0.15748	-1.4121	-1.0048688932160400	3007
**C11orf16**	0.1575	-2.6643	-1.0047858376428100	3009
**S100A4**	0.1575	-1.2163	-1.0047858376428100	3008
**SCN9A**	0.15754	-1.7698	-1.0046197472865700	3010
**SAA4**	0.15758	-2.8143	-1.0044536846391200	3011
**FAM153A**	0.15767	-4.2283	-1.004080144921030	3012
**FAM13C**	0.15771	-2.3593	-1.003914172231740	3013
**TRIM49C**	0.15775	-3.3366	-1.0037482271925500	3014
**FHL3**	0.15787	-2.8058	-1.0032505578379500	3015
**OR5AS1**	0.15791	-1.7818	-1.003084723261520	3016
**KRTAP5-3**	0.15792	-2.3111	-1.0030432689275400	3017
**GRIN3A**	0.1581	-3.1089	-1.002297385446610	3018
**CRAMP1**	0.15814	-3.7105	-1.002131709275430	3020
**TMPRSS9**	0.15814	-0.75425	-1.002131709275430	3019
**CRHR1**	0.15815	-0.25527	-1.0020902945304300	3021
**ST6GAL2**	0.15815	-2.5728	-1.0020902945304300	3022
**TMEM205**	0.15831	-4.177	-1.0014278922075200	3024
**TSSK2**	0.15831	-3.8567	-1.0014278922075200	3023
**DDX19B**	0.15855	-1.3276	-1.0004351117849000	3026
**RTN4IP1**	0.15855	-3.0428	-1.0004351117849000	3027
**TNFRSF14**	0.15855	-1.2348	-1.0004351117849000	3025
**PHEX**	0.15875	-0.35075	-0.9996085473686820	3028
**GNPDA2**	0.15876	-3.2605	-0.9995672370743180	3029
**PAFAH1B2**	0.15887	-3.269	-0.9991129363679610	3030
**OPTN**	0.15904	-4.2904	-0.998411240586838	3031
**CSTF3**	0.1591	-2.2548	-0.9981637005935720	3033
**FUNDC1**	0.1591	-1.1329	-0.9981637005935720	3032
**SNAP91**	0.1591	-3.2618	-0.9981637005935720	3034
**TNFSF13**	0.15913	-3.5517	-0.9980399535304010	3035
**MBL2**	0.15928	-2.8215	-0.9974214473224450	3036
**N4BP1**	0.15937	-1.5086	-0.9970505266802300	3037

**PDYN**	0.15937	-4.0042	-0.9970505266802300	3038
**RAP1GAP**	0.15954	-0.45238	-0.9963502728097470	3039
**SMARCC2**	0.15964	-0.68978	-0.9959385868973170	3041
**SMKR1**	0.15964	-0.44932	-0.9959385868973170	3040
**CES1**	0.15965	-2.9696	-0.9958974275895220	3045
**MMP20**	0.15965	-0.42212	-0.9958974275895220	3042
**OSBPL1A**	0.15965	-1.2598	-0.9958974275895220	3044
**SHC3**	0.15965	-0.95619	-0.9958974275895220	3043
**GALNTL6**	0.15975	-4.3159	-0.9954859272659540	3046
**ANKRD31**	0.15978	-3.404	-0.995362510035616	3048
**AXIN1**	0.15978	-3.2378	-0.995362510035616	3047
**CAVIN2**	0.15978	-3.6732	-0.995362510035616	3049
**MACC1**	0.15978	-4.1156	-0.995362510035616	3050
**DAGLB**	0.16018	-2.8053	-0.9937183933216410	3053
**FANCG**	0.16018	-1.2152	-0.9937183933216410	3052
**MRPS18B**	0.16018	-0.63037	-0.9937183933216410	3051
**DIP2B**	0.16038	-3.4304	-0.9928973414472080	3054
**GXYLT1**	0.16045	-1.1649	-0.9926101313773650	3055
**TENM1**	0.16059	-0.2252	-0.992035956737155	3056
**LHX6**	0.16063	-1.18	-0.9918719669008130	3057
**EPHA3**	0.16076	-2.5469	-0.9913391840526420	3060
**NEURL1B**	0.16076	-0.45336	-0.9913391840526420	3058
**NOTCH4**	0.16076	-2.177	-0.9913391840526420	3059
**CACNG3**	0.16078	-1.8494	-0.9912572424348710	3061
**SNX5**	0.1609	-3.0062	-0.9907657324298960	3062
**IL18BP**	0.1612	-0.6099	-0.9895380033000800	3063
**S100P**	0.1612	-1.7837	-0.9895380033000800	3064
**CNFN**	0.16128	-3.4657	-0.9892108606912090	3068
**OR52E4**	0.16128	-2.1027	-0.9892108606912090	3065
**RTF1**	0.16128	-2.3647	-0.9892108606912090	3066
**ZNF865**	0.16128	-2.4139	-0.9892108606912090	3067
**GRPEL1**	0.16149	-0.094956	-0.9883526146111080	3069

**TIMP1**	0.16166	-0.99511	-0.9876583768204950	3070
**CLPSL1**	0.16183	-3.5485	-0.9869646147210210	3074
**INTS4**	0.16183	-2.1384	-0.9869646147210210	3072
**POMT1**	0.16183	-2.1093	-0.9869646147210210	3071
**RABGEF1**	0.16183	-3.2309	-0.9869646147210210	3073
**TRPT1**	0.16214	-3.582	-0.9857007405149490	3075
**PPARG**	0.16218	-0.042375	-0.9855377746453310	3076
**E2F4**	0.16222	-3.727	-0.9853748349452650	3077
**SMG6**	0.16223	-5.1854	-0.9853341041077490	3078
**ADAMTS5**	0.16227	-0.98587	-0.9851711971017230	3080
**CYB5RL**	0.16227	-1.462	-0.9851711971017230	3081
**GOLGA6L22**	0.16227	-0.92965	-0.9851711971017230	3079
**SPATA4**	0.16227	-1.5904	-0.9851711971017230	3082
**C9orf50**	0.16251	-1.2571	-0.9841943035805330	3085
**JRKL**	0.16251	-0.70115	-0.9841943035805330	3084
**LIPA**	0.16251	-0.69156	-0.9841943035805330	3083
**BCL7A**	0.16284	-3.8643	-0.982852606555285	3090
**HYAL2**	0.16284	-3.9592	-0.982852606555285	3091
**KCNV1**	0.16284	-3.7129	-0.982852606555285	3089
**LDLR**	0.16284	-3.5136	-0.982852606555285	3088
**MAT1A**	0.16284	-3.0836	-0.982852606555285	3087
**TULP2**	0.16284	-2.1213	-0.982852606555285	3086
**FAM159B**	0.16293	-2.9211	-0.982486996143029	3092
**C7orf66**	0.16341	-2.1845	-0.9805392876779590	3093
**CEP295NL**	0.16341	-2.9017	-0.9805392876779590	3095
**PDE6C**	0.16341	-2.6555	-0.9805392876779590	3094
**AGPAT3**	0.16343	-1.3045	-0.9804582138775610	3097
**FAM83H**	0.16343	-0.9888	-0.9804582138775610	3096
**PRKAB1**	0.16345	-0.51534	-0.9803771465211550	3098
**RBBP5**	0.16348	-1.8642	-0.9802555575656270	3099
**GNL1**	0.16362	-2.3404	-0.9796883339480380	3100
**FAM122C**	0.83634	-0.041459	-0.979526327943605	3101

**CAMK2A**	0.16386	-3.0983	-0.9787166832407080	3102
**NLRP5**	0.16401	-1.9	-0.9781098704963220	3103
**ATAD2**	0.16417	-3.5644	-0.9774630002948650	3104
**NOL8**	0.16418	-2.4918	-0.9774225844872760	3105
**AMPD2**	0.16421	-0.51244	-0.9773013466426990	3106
**ANKRD30B**	0.16421	-0.59657	-0.9773013466426990	3107
**FTSJ3**	0.16421	-2.7793	-0.9773013466426990	3110
**IFI16**	0.16421	-3.7498	-0.9773013466426990	3111
**PCMTD1**	0.16421	-1.845	-0.9773013466426990	3108
**RXFP3**	0.16421	-2.1191	-0.9773013466426990	3109
**SIAH3**	0.16428	-0.59201	-0.9770185141868290	3112
**UBP1**	0.16443	-0.49516	-0.9764127076588780	3113
**AKR7A2**	0.16455	-3.9022	-0.9759283203230340	3114
**FAM213B**	0.16474	-1.9946	-0.9751618416572890	3115
**ALG14**	0.16476	-2.6178	-0.9750811930166280	3119
**ASIC2**	0.16476	-2.4922	-0.9750811930166280	3118
**DST**	0.16476	-1.3205	-0.9750811930166280	3117
**PPP1R3F**	0.16476	-0.77095	-0.9750811930166280	3116
**FOLR3**	0.16485	-0.70007	-0.9747183525892270	3120
**ABRA**	0.16486	-2.6407	-0.9746780449071860	3121
**BZW2**	0.16488	-1.2774	-0.9745974342934260	3122
**KRTAP1-4**	0.16493	-3.2695	-0.9743959354603810	3123
**POLD2**	0.1652	-1.926	-0.9733085246616010	3124
**TNS4**	0.16524	-0.083715	-0.9731475246334360	3125
**SAP30BP**	0.16536	-3.754	-0.9726646758265490	3126
**FGFR1OP**	0.16545	-2.0351	-0.9723026879868880	3127
**LAIR1**	0.16545	-3.077	-0.9723026879868880	3128
**PNLIPRP2**	0.16545	-3.283	-0.9723026879868880	3129
**IGLON5**	0.16547	-2.5537	-0.9722222635454880	3131
**MAGI1**	0.16547	-2.7308	-0.9722222635454880	3133
**PITPNC1**	0.16547	-2.6849	-0.9722222635454880	3132
**TECPR1**	0.16547	-1.1187	-0.9722222635454880	3130

**APH1B**	0.16588	-1.4186	-0.9705749460477760	3134
**LHCGR**	0.16588	-1.803	-0.9705749460477760	3135
**RPL11**	0.16599	-2.8342	-0.9701334307419230	3136
**USB1**	0.16618	-1.8797	-0.9693712584958240	3137
**AMIGO2**	0.16619	-0.39181	-0.9693311597654150	3138
**EDNRA**	0.16619	-2.097	-0.9693311597654150	3141
**MCPH1**	0.16619	-1.5539	-0.9693311597654150	3139
**RAD54L2**	0.16619	-2.0406	-0.9693311597654150	3140
**TBCA**	0.16632	-4.3889	-0.9688100180290530	3142
**C5orf38**	0.16658	-1.8488	-0.9677685231041180	3143
**CAMTA1**	0.16658	-2.667	-0.9677685231041180	3144
**OR5D13**	0.16662	-1.7465	-0.9676083862759380	3145
**MTMR7**	0.16664	-0.77866	-0.9675283271660660	3146
**PRKAR1A**	0.16664	-3.8047	-0.9675283271660660	3148
**WDR83OS**	0.16664	-3.3365	-0.9675283271660660	3147
**MAD2L1BP**	0.16665	-2.2858	-0.9674882999365390	3149
**C16orf45**	0.16676	-1.539	-0.9670481026730560	3151
**DDX42**	0.16676	-3.1965	-0.9670481026730560	3154
**PRPF4**	0.16676	-2.7426	-0.9670481026730560	3153
**RFPL4A**	0.16676	-0.23903	-0.9670481026730560	3150
**ZMYND12**	0.16676	-1.9811	-0.9670481026730560	3152
**FOXE3**	0.16681	-1.5088	-0.9668480749426860	3155
**OR51T1**	0.16686	-1.5629	-0.966648085889433	3156
**HNRNPL**	0.16693	-2.7319	-0.9663681661487410	3157
**PELP1**	0.16695	-2.9663	-0.9662882029866660	3158
**GPR21**	0.16699	-0.18184	-0.9661282951952460	3159
**WARS2**	0.16711	-0.016252	-0.965648719977347	3160
**BCKDHB**	0.83252	-0.096134	-0.9641714257939780	3161
**TRAF7**	0.16758	-2.6937	-0.9637725181697890	3162
**CALY**	0.16771	-2.2629	-0.9632541674197970	3164
**OR2G3**	0.16771	-1.5726	-0.9632541674197970	3163
**RBMXL3**	0.16771	-3.0104	-0.9632541674197970	3166

**RIN2**	0.16771	-2.5284	-0.9632541674197970	3165
**UBE2I**	0.16771	-4.2798	-0.9632541674197970	3167
**DKK 3.00**	0.16791	-3.5601	-0.9624572097126170	3169
**TCTEX1D1**	0.16791	-1.1191	-0.9624572097126170	3168
**SPN**	0.16827	-3.6227	-0.9610242241569130	3170
**STX10**	0.16828	-4.3007	-0.960984447172363	3171
**DTNBP1**	0.16858	-1.7896	-0.9597918438257040	3173
**PAK4**	0.16858	-0.69417	-0.9597918438257040	3172
**PIGX**	0.16858	-2.5735	-0.9597918438257040	3174
**SFTPD**	0.16858	-3.293	-0.9597918438257040	3175
**WNT3A**	0.83134	-0.079654	-0.9594740467541100	3176
**TIMM22**	0.16873	-2.4029	-0.9591960537852160	3177
**CD151**	0.16887	-1.2869	-0.9586402901071680	3178
**P2RY11**	0.16887	-3.0139	-0.9586402901071680	3179
**HIPK4**	0.16898	-3.8752	-0.9582038262814170	3180
**ACOT4**	0.1693	-0.45999	-0.9569351499516070	3181
**BPIFA2**	0.1693	-2.0416	-0.9569351499516070	3183
**PTDSS2**	0.1693	-1.2408	-0.9569351499516070	3182
**BPIFA3**	0.16932	-1.2973	-0.956855908819102	3185
**GDNF**	0.16932	-4.2035	-0.956855908819102	3188
**KCNJ2**	0.16932	-2.1895	-0.956855908819102	3186
**OR7G1**	0.16932	-3.5966	-0.956855908819102	3187
**TRDMT1**	0.16932	-0.8619	-0.956855908819102	3184
**TRIM72**	0.16933	-4.2197	-0.9568162905058570	3189
**PNPLA1**	0.16949	-0.56592	-0.9561826016150550	3190
**RGS9BP**	0.16966	-0.17627	-0.9555097276098860	3191
**CHMP5**	0.83033	-0.12942	-0.9554701602582540	3192
**CDC27**	0.16979	-1.0562	-0.9549954686611890	3193
**PLA2G4F**	0.1698	-2.6032	-0.9549559207413390	3194
**BEND4**	0.16988	-3.0327	-0.9546395911349110	3196
**PLSCR3**	0.16988	-2.8379	-0.9546395911349110	3195
**TIMM29**	0.16997	-1.8559	-0.9542838344713840	3197

**HPD**	0.17028	-2.6175	-0.9530593737669960	3198
**THEGL**	0.1703	-3.5433	-0.9529804253684370	3199
**RFC5**	0.17033	-2.392	-0.9528620139064080	3200
**PSRC1**	0.17049	-3.9524	-0.9522307116104910	3201
**GAL3ST2**	0.17052	-4.4246	-0.9521123846754170	3207
**KRT6B**	0.17052	-2.7818	-0.9521123846754170	3205
**LOC1001313**	0.17052	-1.2355	-0.9521123846754170	3203
**MOCS3**	0.17052	-2.1465	-0.9521123846754170	3204
**RBM19**	0.17052	-1.1877	-0.9521123846754170	3202
**SPACA9**	0.17052	-3.1366	-0.9521123846754170	3206
**ELOVL5**	0.17093	-3.85	-0.9504965838153780	3210
**NADK2**	0.17093	-3.5284	-0.9504965838153780	3209
**SESN2**	0.17093	-3.9902	-0.9504965838153780	3211
**SGK1**	0.17093	-3.1383	-0.9504965838153780	3208
**ATG4D**	0.17124	-0.41189	-0.9492765263254260	3213
**NBPF19**	0.17124	-0.02812	-0.9492765263254260	3212
**LSG1**	0.17126	-4.8631	-0.9491978614597780	3215
**MYH14**	0.17126	-1.3976	-0.9491978614597780	3214
**ZSWIM4**	0.82871	-0.094152	-0.9490798751733970	3216
**SIK3**	0.17135	-2.9494	-0.9488439422272960	3217
**TMEM50B**	0.17138	-2.2843	-0.9487259955583790	3218
**TIAL1**	0.17156	-1.2775	-0.9480185925134020	3219
**CASC10**	0.1716	-3.2679	-0.9478614562538980	3226
**DYM**	0.1716	-0.73656	-0.9478614562538980	3222
**EXOC2**	0.1716	-3.4772	-0.9478614562538980	3227
**GCA**	0.1716	-3.0208	-0.9478614562538980	3225
**GPAT2**	0.1716	-0.97232	-0.9478614562538980	3223
**PIN4**	0.1716	-0.65775	-0.9478614562538980	3221
**SBSPON**	0.1716	-1.4746	-0.9478614562538980	3224
**ZCCHC7**	0.1716	-0.24446	-0.9478614562538980	3220
**HSPA4**	0.17169	-1.3104	-0.9475079852130030	3228
**PARP16**	0.17176	-4.0205	-0.9472331451163820	3229

**PCDHB13**	0.17201	-1.3821	-0.9462521568761990	3230
**CSPG4**	0.17214	-3.1415	-0.9457424026982380	3232
**OR6Q1**	0.17214	-0.95456	-0.9457424026982380	3231
**C11orf54**	0.17223	-3.1553	-0.9453896398726340	3233
**FCGR2A**	0.17223	-4.1426	-0.9453896398726340	3235
**PMF1-BGLA**	0.17223	-3.6608	-0.9453896398726340	3234
**TBR1**	0.17243	-0.31788	-0.94460614336422	3236
**ACSL6**	0.17253	-1.2293	-0.9442146124809	3237
**FBXW10**	0.17253	-1.6801	-0.9442146124809	3239
**INPPL1**	0.17253	-3.0527	-0.9442146124809	3241
**PACS2**	0.17253	-2.9593	-0.9442146124809	3240
**TEX47**	0.17253	-1.5636	-0.9442146124809	3238
**ENHO**	0.17267	-1.1798	-0.9436667122878440	3242
**FGD1**	0.17267	-2.1904	-0.9436667122878440	3244
**STMND1**	0.17267	-1.616	-0.9436667122878440	3243
**LRRC36**	0.17276	-4.8105	-0.9433146402775170	3245
**KRTAP5-8**	0.17291	-3.2535	-0.9427281132878220	3246
**WDR45B**	0.17291	-3.2831	-0.9427281132878220	3247
**CNTNAP3B**	0.17297	-3.703	-0.9424935932802410	3248
**BAGE5**	0.17314	-2.0381	-0.9418294012467500	3249
**IDS**	0.17318	-2.9818	-0.9416731811430270	3250
**SMAD3**	0.17322	-0.87802	-0.941516984017164	3251
**LIPN**	0.17328	-2.2254	-0.9412827313887410	3252
**SPRED1**	0.1733	-3.444	-0.9412046586567520	3253
**ADNP**	0.17339	-1.0976	-0.9408534023334980	3255
**ARL13A**	0.17339	-3.2539	-0.9408534023334980	3262
**BCL2**	0.17339	-0.64514	-0.9408534023334980	3254
**CWH43**	0.17339	-1.5854	-0.9408534023334980	3257
**HIST1H2BN**	0.17339	-2.4876	-0.9408534023334980	3258
**PLBD2**	0.17339	-2.684	-0.9408534023334980	3259
**PPP2R3B**	0.17339	-2.8489	-0.9408534023334980	3260
**PROCR**	0.17339	-2.8884	-0.9408534023334980	3261

**WDR7**	0.17339	-1.3348	-0.9408534023334980	3256
**SFTA3**	0.17341	-0.77034	-0.940775361136189	3263
**STC1**	0.17342	-3.6172	-0.9407363426861030	3264
**ACOD1**	0.17356	-3.3745	-0.9401902346868340	3265
**CORO2A**	0.17357	-3.234	-0.9401512377018800	3266
**RILP**	0.17361	-1.9148	-0.9399952640573660	3267
**RBM18**	0.17391	-0.98124	-0.9388261898089340	3268
**CRLF3**	0.17411	-0.080081	-0.9380475193141980	3269
**ZFAND1**	0.17411	-0.89544	-0.9380475193141980	3270
**FCRL2**	0.17414	-2.3813	-0.9379307677918450	3274
**KBTBD13**	0.17414	-1.4731	-0.9379307677918450	3271
**MCM9**	0.17414	-3.7047	-0.9379307677918450	3275
**P2RX7**	0.17414	-1.7058	-0.9379307677918450	3272
**SLC25A6**	0.17414	-2.1419	-0.9379307677918450	3273
**KANK4**	0.1742	-3.6481	-0.9376973030930680	3276
**WEE1**	0.17425	-1.9764	-0.9375027882126040	3277
**HYAL3**	0.17431	-2.8048	-0.9372694171679000	3278
**CYLC1**	0.17443	-2.8254	-0.9368028281466140	3279
**OPRL1**	0.17445	-1.1386	-0.9367250831367730	3280
**TSHZ3**	0.17453	-1.965	-0.9364141596986190	3281
**CBL**	0.17462	-0.79781	-0.9360644790021880	3283
**FCHO1**	0.17462	-2.5536	-0.9360644790021880	3284
**POLR2G**	0.17462	-0.70253	-0.9360644790021880	3282
**NSRP1**	0.17464	-2.9538	-0.935986787723577	3285
**OR8D4**	0.17474	-4.2313	-0.935598416041777	3287
**RWDD4**	0.82526	-0.16529	-0.935598416041777	3286
**USP47**	0.17475	-1.3475	-0.9355595866349540	3288
**C11orf70**	0.17478	-3.3901	-0.9354431068769090	3289
**FZD5**	0.17485	-2.7877	-0.9351713701182910	3290
**ATF7**	0.17489	-3.4387	-0.935016122969904	3291
**B3GAT2**	0.17501	-1.9384	-0.9345505166765690	3292
**GCGR**	0.17503	-0.57869	-0.9344729353238380	3293

**NSMF**	0.17512	-0.6697	-0.9341238888156910	3294
**TGFBRAP1**	0.17512	-0.81772	-0.9341238888156910	3295
**ARID3C**	0.17518	-3.2074	-0.9338912543565190	3296
**PKP3**	0.1753	-4.0077	-0.933426136993717	3297
**CBR1**	0.17537	-2.0817	-0.9331549117624790	3298
**NUP37**	0.17545	-3.1502	-0.9328450241011410	3301
**PRM1**	0.17545	-2.2951	-0.9328450241011410	3300
**RUVBL2**	0.17545	-1.4273	-0.9328450241011410	3299
**ACSM1**	0.17563	-4.1964	-0.9321481041761150	3304
**H1FX**	0.17563	-1.9504	-0.9321481041761150	3302
**NPIPB15**	0.17563	-2.8154	-0.9321481041761150	3303
**MBD3L2**	0.82407	-0.047134	-0.9309875759498310	3305
**PRR9**	0.17604	-2.7052	-0.9305623629858110	3309
**PRSS45**	0.17604	-0.58147	-0.9305623629858110	3307
**PRUNE1**	0.17604	-0.05534	-0.9305623629858110	3306
**VSIG10L**	0.17604	-2.6519	-0.9305623629858110	3308
**TIGD2**	0.17605	-0.13955	-0.9305237156046940	3310
**HMMR**	0.82389	-0.30575	-0.9302918604982080	3311
**LAT2**	0.17622	-3.3604	-0.9298669226360610	3313
**SLC35F5**	0.17622	-3.1361	-0.9298669226360610	3312
**UNC13B**	0.17637	-2.8982	-0.9292877323625680	3314
**BRINP3**	0.82354	-0.044039	-0.9289403677869320	3315
**ZSCAN4**	0.17649	-3.982	-0.9288246045010560	3316
**KIF2C**	0.17654	-4.2504	-0.928631693346846	3317
**PAPOLB**	0.17657	-1.0067	-0.9285159632407840	3318
**CNMD**	0.17661	-2.9193	-0.9283616757748190	3319
**ERLIN1**	0.17663	-2.5615	-0.9282845403285000	3321
**IMPAD1**	0.17663	-2.6261	-0.9282845403285000	3322
**MORC1**	0.17663	-0.78229	-0.9282845403285000	3320
**RPS21**	0.17663	-3.1087	-0.9282845403285000	3323
**FSHR**	0.17673	-2.9872	-0.9278989459135610	3324
**ZSCAN31**	0.17676	-2.5479	-0.9277832944892730	3325

**ZNF107**	0.17685	-1.9208	-0.9274364146475050	3326
**OR52H1**	0.17707	-3.7694	-0.9265889555623780	3329
**PSMB1**	0.17707	-1.1301	-0.9265889555623780	3328
**TUBE1**	0.17707	-0.34768	-0.9265889555623780	3327
**ZNF92**	0.17707	-3.9143	-0.9265889555623780	3330
**GOLGA6L4**	0.17711	-1.6717	-0.9264349435807070	3331
**DCAF6**	0.17713	-1.1988	-0.9263579458298370	3332
**EAF2**	0.17723	-2.2005	-0.9259730394255440	3336
**FLT3LG**	0.17723	-1.6863	-0.9259730394255440	3334
**KLHDC7A**	0.17723	-0.35532	-0.9259730394255440	3333
**PFN1**	0.17723	-2.5569	-0.9259730394255440	3337
**TRPM1**	0.17723	-1.9803	-0.9259730394255440	3335
**ULBP2**	0.17723	-2.8979	-0.9259730394255440	3338
**ADH4**	0.1773	-3.5927	-0.9257036865480040	3339
**TMCO2**	0.1773	-4.6403	-0.9257036865480040	3340
**CEBPB**	0.1775	-2.7167	-0.9249344766936440	3342
**CYP4F8**	0.1775	-2.8427	-0.9249344766936440	3343
**IDE**	0.1775	-3.7281	-0.9249344766936440	3344
**MKKS**	0.1775	-1.1401	-0.9249344766936440	3341
**ATIC**	0.82246	-0.14721	-0.924780700387691	3345
**PELI2**	0.17758	-1.4775	-0.9246269459470300	3346
**NDEL1**	0.1778	-2.1786	-0.9237816869255820	3347
**TBC1D31**	0.17781	-2.7659	-0.9237432817430610	3348
**BARX2**	0.17819	-0.79119	-0.9222848929707170	3349
**FAM186B**	0.17819	-2.5553	-0.9222848929707170	3351
**OR8G2P**	0.17819	-2.411	-0.9222848929707170	3350
**TPMT**	0.17819	-3.6242	-0.9222848929707170	3352
**APC2**	0.17821	-0.93379	-0.9222081900051340	3353
**IQCF5**	0.17821	-3.2055	-0.9222081900051340	3354
**RAD51**	0.17822	-1.8751	-0.9221698405568980	3355
**OLFM2**	0.82176	-0.097165	-0.9220931457287760	3356
**FAM184B**	0.17833	-0.98481	-0.9217480860998400	3357

**TFPI**	0.17876	-3.1381	-0.9201009802728950	3358
**MPZ**	0.17883	-3.1881	-0.9198330829798820	3359
**HOXD10**	0.17893	-3.1058	-0.9194504870367350	3360
**LEP**	0.17893	-3.3367	-0.9194504870367350	3361
**EIF4A3**	0.17908	-0.30109	-0.9188768453399970	3362
**HDAC5**	0.17908	-2.9089	-0.9188768453399970	3364
**PYGO2**	0.17908	-3.0466	-0.9188768453399970	3365
**SLC24A3**	0.17908	-1.7548	-0.9188768453399970	3363
**LRRC63**	0.17915	-0.89814	-0.9186092493354640	3366
**FBXO21**	0.17932	-2.572	-0.9179596470119940	3368
**FERD3L**	0.17932	-1.9113	-0.9179596470119940	3367
**LMF2**	0.17932	-3.9004	-0.9179596470119940	3369
**CEP126**	0.17954	-0.69748	-0.9171195597450050	3370
**MYO19**	0.17964	-1.2699	-0.9167379157951200	3371
**NLRP3**	0.17965	-0.31555	-0.9166997587438750	3373
**WDR55**	0.17965	-0.17615	-0.9166997587438750	3372
**SEZ6L**	0.17971	-2.0503	-0.916470844458433	3375
**SOX18**	0.82029	-0.23671	-0.9164708444584330	3374
**LYPD8**	0.17975	-3.1862	-0.9163182616115330	3376
**DNER**	0.17981	-0.86889	-0.9160894273324780	3377
**MYL2**	0.17991	-3.0359	-0.9157081434315990	3378
**SIGLEC12**	0.17994	-2.5354	-0.9155937842180290	3379
**URM1**	0.17997	-3.4112	-0.9154794369773530	3380
**CLP 1**	0.17999	-2.1797	-0.9154032121332000	3381
**BCAT1**	0.18011	-2.287	-0.9149459747115940	3384
**GK5**	0.18011	-2.7166	-0.9149459747115940	3386
**LRRC10B**	0.18011	-2.6638	-0.9149459747115940	3385
**NOL7**	0.18011	-3.1681	-0.9149459747115940	3387
**SGPL1**	0.18011	-1.2765	-0.9149459747115940	3383
**UGT2B11**	0.18011	-0.32978	-0.9149459747115940	3382
**PSMA6**	0.18019	-1.4332	-0.9146412560040240	3388
**LOC1019278**	0.18021	-1.7949	-0.9145650895963120	3389

**CBX5**	0.18032	-4.1967	-0.9141462691546610	3392
**FAM84A**	0.18032	-2.8098	-0.9141462691546610	3391
**PDPN**	0.18032	-1.2283	-0.9141462691546610	3390
**CLCN6**	0.18033	-3.2918	-0.9141082025202360	3393
**NIT1**	0.18038	-2.3425	-0.9139178892135260	3394
**OR2M4**	0.18044	-4.2544	-0.9136895569299610	3395
**MRPL44**	0.18046	-1.2743	-0.9136134567538490	3396
**GALNTL5**	0.18047	-1.029	-0.9135754086498330	3397
**KCNAB1**	0.18051	-3.5528	-0.913423229457272	3398
**FAM155A**	0.18056	-2.0804	-0.9132330352084300	3399
**RAB30**	0.18069	-1.2168	-0.9127386846931450	3400
**LOC285556**	0.1807	-0.5765	-0.9127006669694140	3401
**CCR5**	0.18074	-0.24142	-0.9125486092642470	3402
**CACNA1G**	0.18075	-1.392	-0.912510598134661	3403
**SMAD5**	0.18076	-1.8726	-0.9124725883234660	3404
**CCDC80**	0.18093	-3.1321	-0.9118266231050460	3405
**CERCAM**	0.18105	-1.9737	-0.911370876641182	3407
**HCCS**	0.18105	-1.7261	-0.911370876641182	3406
**ARIH2OS**	0.81892	-0.1255	-0.9112569696002540	3408
**NPHS1**	0.18115	-0.45069	-0.9109912324729800	3409
**C12orf66**	0.18145	-2.3173	-0.9098530869191650	3412
**DCTN3**	0.18145	-1.1533	-0.9098530869191650	3410
**GSTO1**	0.18145	-2.6645	-0.9098530869191650	3413
**NIF3L1**	0.18145	-3.3723	-0.9098530869191650	3415
**SLC7A6**	0.18145	-1.1568	-0.9098530869191650	3411
**TFDP3**	0.18145	-3.0955	-0.9098530869191650	3414
**VAC14**	0.18159	-2.2304	-0.9093223554910130	3416
**HIPK3**	0.18161	-1.0895	-0.9092465576236070	3417
**POGK**	0.18162	-2.2431	-0.9092086606488040	3418
**MUC16**	0.18176	-3.5032	-0.9086782400387330	3419
**OR2A25**	0.18187	-2.7254	-0.9082616602695630	3420
**SLC9C2**	0.18191	-2.7946	-0.9081102157913550	3421

**ARL17A**	0.1821	-3.7926	-0.9073911387544390	3422
**PALB2**	0.18211	-4.4473	-0.9073533055915640	3423
**SLC7A3**	0.18215	-2.8634	-0.9072019859255530	3424
**C1QL4**	0.18219	-0.71121	-0.9070506870294480	3425
**CCRL2**	0.18219	-1.9025	-0.9070506870294480	3428
**DLEC1**	0.18219	-1.1035	-0.9070506870294480	3426
**JAM3**	0.18219	-3.2753	-0.9070506870294480	3432
**MFSD11**	0.18219	-2.6375	-0.9070506870294480	3430
**MKS1**	0.18219	-2.8451	-0.9070506870294480	3431
**TMPRSS11D**	0.18219	-1.9908	-0.9070506870294480	3429
**ZNF574**	0.18219	-1.4836	-0.9070506870294480	3427
**GCLM**	0.18237	-2.4614	-0.9063700987790280	3433
**TMEM179**	0.18252	-3.826	-0.9058032624451150	3434
**FKBP9**	0.18279	-1.8481	-0.9047836897591820	3435
**GTF2I**	0.18279	-2.1596	-0.9047836897591820	3436
**MTCH1**	0.18279	-3.2536	-0.9047836897591820	3437
**ELF3**	0.18285	-0.4917	-0.9045572457604040	3438
**QRICH2**	0.18285	-3.099	-0.9045572457604040	3439
**CERS5**	0.18299	-0.42167	-0.9040290567177420	3440
**CYP1A2**	0.18313	-0.12786	-0.9035011197637460	3441
**MSL1**	0.18345	-3.177	-0.9022953512673290	3442
**ELOVL6**	0.18354	-2.9973	-0.9019564651786120	3448
**FZD1**	0.18354	-2.2938	-0.9019564651786120	3447
**GLUL**	0.18354	-1.7702	-0.9019564651786120	3444
**NDUFA3**	0.18354	-2.0072	-0.9019564651786120	3446
**PRODH2**	0.18354	-1.2492	-0.9019564651786120	3443
**ROCK2**	0.18354	-1.999	-0.9019564651786120	3445
**CCDC81**	0.1836	-4.0396	-0.9017305986548360	3451
**COA6**	0.1836	-4.0352	-0.9017305986548360	3450
**TRUB2**	0.1836	-3.7551	-0.9017305986548360	3449
**CXorf40A**	0.18367	-1.1969	-0.9014671458383230	3453
**PTX4**	0.81633	-0.13633	-0.9014671458383230	3452

**TRPV6**	0.1837	-2.1082	-0.9013542566402320	3454
**TSSK3**	0.18371	-2.7982	-0.9013166294600870	3455
**EHBP1**	0.18392	-0.55819	-0.9005267532279320	3457
**RNF4**	0.18392	-0.5511	-0.9005267532279320	3456
**TOPBP1**	0.18394	-4.3768	-0.9004515562220000	3458
**ITGB1BP1**	0.18401	-1.8399	-0.9001884067881700	3459
**PLA2G4A**	0.18411	-2.3914	-0.899812587121707	3460
**COQ10A**	0.18421	-4.1241	-0.8994368945020340	3462
**OR2T34**	0.18421	-2.4075	-0.8994368945020340	3461
**ACY1**	0.18432	-2.7518	-0.8990237791937140	3466
**GABRR1**	0.18432	-0.86126	-0.8990237791937140	3463
**HIST1H2BA**	0.18432	-2.3658	-0.8990237791937140	3465
**OVCH2**	0.18432	-1.0551	-0.8990237791937140	3464
**SYT5**	0.18432	-3.2807	-0.8990237791937140	3467
**MFAP2**	0.1848	-2.7055	-0.897222886654245	3469
**SCRT1**	0.1848	-2.3028	-0.897222886654245	3468
**CCNE2**	0.18495	-3.6202	-0.8966607042410520	3476
**NUCB1**	0.18495	-1.5445	-0.8966607042410520	3473
**POTEH**	0.18495	-2.5917	-0.8966607042410520	3474
**RB1**	0.18495	-0.37056	-0.8966607042410520	3471
**SMTNL2**	0.18495	-0.71924	-0.8966607042410520	3472
**SPEF2**	0.18495	-2.6687	-0.8966607042410520	3475
**ZFP69B**	0.18495	-0.18168	-0.8966607042410520	3470
**P2RY13**	0.18508	-3.2527	-0.896173708613863	3477
**ARHGAP39**	0.18515	-0.33364	-0.8959115682145320	3480
**ARID1A**	0.18515	-0.52378	-0.8959115682145320	3482
**ECEL1**	0.18515	-0.24129	-0.8959115682145320	3479
**SCGB2B2**	0.18515	-0.45135	-0.8959115682145320	3481
**TIAF1**	0.18515	-0.14021	-0.8959115682145320	3478
**YY1AP1**	0.18523	-3.5567	-0.8956120545517670	3483
**EVX1**	0.1853	-2.175	-0.8953500459886330	3484
**PTPRD**	0.18547	-2.359	-0.8947139952178110	3485

**AANAT**	0.18552	-3.4756	-0.8945269903386340	3486
**BLOC1S5**	0.18561	-3.0439	-0.8941904603629270	3488
**PDGFC**	0.18561	-1.9747	-0.8941904603629270	3487
**PCNA**	0.18563	-2.6146	-0.8941156896758260	3489
**RBBP8**	0.18566	-2.016	-0.8940035430167450	3490
**SEMG2**	0.18572	-1.1415	-0.8937792834228040	3491
**KIAA0895**	0.18583	-3.0591	-0.8933682575351020	3495
**OR51G1**	0.18583	-3.1925	-0.8933682575351020	3496
**PRLH**	0.18583	-2.9059	-0.8933682575351020	3494
**RPS6KA2**	0.18583	-3.5056	-0.8933682575351020	3497
**SLC36A4**	0.18583	-0.012731	-0.8933682575351020	3492
**SND1**	0.18583	-2.7079	-0.8933682575351020	3493
**TRMT112**	0.18604	-1.3356	-0.8925839905485910	3498
**ADIPOR2**	0.18621	-1.152	-0.8919495096326710	3499
**ZSCAN29**	0.18621	-1.2617	-0.8919495096326710	3500
**TSPYL5**	0.18624	-2.9188	-0.8918375796834870	3501
**WISP1**	0.81375	-0.19426	-0.8918002721832920	3502
**CTGF**	0.18626	-3.0773	-0.8917629659243060	3503
**VIPAS39**	0.18643	-0.97527	-0.8911289492962630	3504
**PRDM8**	0.18654	-1.2325	-0.8907188940580170	3505
**ATOH1**	0.81344	-0.005965	-0.890644354650496	3506
**USP17L10**	0.18661	-2.9346	-0.8904580277777030	3507
**TCEANC2**	0.18663	-2.3281	-0.8903835056841810	3508
**PLCH1**	0.18681	-2.0244	-0.8897130292143440	3509
**ARIH1**	0.1869	-2.67	-0.8893779409236090	3514
**GPATCH3**	0.1869	-2.6203	-0.8893779409236090	3512
**ITLN2**	0.1869	-3.069	-0.8893779409236090	3515
**PIK3R2**	0.1869	-0.71755	-0.8893779409236090	3511
**S1PR4**	0.1869	-0.28743	-0.8893779409236090	3510
**SELENOK**	0.1869	-2.6337	-0.8893779409236090	3513
**SIGLEC1**	0.18693	-1.6183	-0.8892662670166450	3516
**KDM4E**	0.18696	-4.3436	-0.8891546041986600	3517

**TFDP2**	0.18698	-0.68028	-0.8890801684787720	3518
**CCL11**	0.18701	-2.9465	-0.8889685241344340	3519
**ESAM**	0.18729	-1.6044	-0.8879270440467450	3520
**MEFV**	0.18729	-2.5541	-0.8879270440467450	3521
**SLC6A2**	0.18743	-3.0748	-0.8874066650195060	3522
**CNOT7**	0.18746	-2.3913	-0.8872951864899750	3523
**IAPP**	0.18752	-2.0541	-0.8870722625045160	3524
**DKC1**	0.18757	-1.4756	-0.8868865261857710	3525
**SOCS2**	0.18763	-1.4448	-0.8866636829815750	3526
**PLK3**	0.18765	-3.2263	-0.8865894116976180	3527
**RPP25**	0.18776	-0.87551	-0.8861810070216810	3528
**PRAMEF2**	0.18787	-3.7379	-0.8857727501021060	3529
**PDLIM3**	0.18788	-1.0349	-0.8857356431574060	3530
**LSS**	0.18794	-2.7617	-0.88551302709517	3531
**TEX35**	0.18798	-0.54553	-0.8853646407640240	3532
**SOS 1**	0.18806	-2.4238	-0.885067926568807	3534
**CD55**	0.18806	-0.64523	-0.885067926568807	3537
**GUK1**	0.18806	-2.8373	-0.885067926568807	3535
**MCTP2**	0.18806	-2.4133	-0.885067926568807	3533
**MICB**	0.18806	-0.5586	-0.885067926568807	3536
**NFIL3**	0.18831	-2.5765	-0.8841411964943360	3538
**CNOT11**	0.18832	-1.39	-0.8841041430859510	3539
**CD70**	0.18836	-0.87412	-0.8839559415890460	3540
**RPL5**	0.18838	-0.36544	-0.8838818481207510	3541
**PTPRCAP**	0.81153	-0.36212	-0.8835484875424740	3542
**ZNF227**	0.18849	-3.4433	-0.8834744207475530	3543
**GFPT2**	0.81148	-0.14375	-0.8833633296416960	3544
**JMJD6**	0.18853	-0.3906	-0.8833263016956310	3545
**LMBR1L**	0.18867	-3.4682	-0.8828080375529080	3546
**SEMA6B**	0.18873	-2.7195	-0.8825859969241810	3547
**IQCF6**	0.18874	-0.20048	-0.8825489943831080	3548
**HPCA**	0.18879	-1.6552	-0.8823639998001690	3549

**NPIPB9**	0.18884	-2.4699	-0.8821790354094050	3551
**NPVF**	0.18884	-1.6891	-0.8821790354094050	3550
**LNX1**	0.18889	-3.1804	-0.8819941011946360	3552
**CRYBG2**	0.18898	-2.2293	-0.8816612955999640	3554
**TMIGD1**	0.18898	-0.86236	-0.8816612955999640	3553
**ELOA3B**	0.18901	-1.4783	-0.8815503821005350	3555
**RRP36**	0.18906	-1.5212	-0.8813655503632930	3556
**CHST3**	0.18908	-2.709	-0.8812916260986840	3559
**PARM1**	0.18908	-1.7788	-0.8812916260986840	3558
**WDR62**	0.18908	-1.4861	-0.8812916260986840	3557
**PPP1R10**	0.18916	-2.9862	-0.8809959771875430	3560
**KAT6A**	0.81083	-0.15068	-0.8809590264880950	3561
**INSC**	0.18934	-2.2136	-0.8803310485178920	3562
**NPAP1**	0.18943	-3.7259	-0.8799987301021550	3563
**CHKB**	0.18948	-0.12933	-0.8798141507464460	3564
**EVI5L**	0.18948	-0.20932	-0.8798141507464460	3566
**OTUD6B**	0.18948	-0.15989	-0.8798141507464460	3565
**CDCA7**	0.18966	-3.2423	-0.879149913102278	3569
**NKX2-4**	0.18966	-2.8713	-0.879149913102278	3568
**WDR36**	0.18966	-2.4545	-0.879149913102278	3567
**PSMA2**	0.18968	-1.1717	-0.8790761328631270	3570
**LRTOMT**	0.1897	-2.8401	-0.8790023574089310	3572
**OR8I2**	0.1897	-1.3369	-0.8790023574089310	3571
**GAB2**	0.81027	-0.4143	-0.878891703197194	3573
**RFC3**	0.18993	-2.7663	-0.8781542832740470	3574
**DNAJC12**	0.19016	-1.4941	-0.8773068402632120	3575
**ATP1B2**	0.19029	-4.2355	-0.876828129324216	3577
**SCN3B**	0.19029	-3.9246	-0.876828129324216	3576
**AMPD1**	0.19047	-1.2697	-0.8761656303202670	3579
**MRO**	0.19047	-2.6063	-0.8761656303202670	3580
**NAA25**	0.19047	-0.42132	-0.8761656303202670	3578
**RGS7**	0.19047	-2.7671	-0.8761656303202670	3581

**STK32A**	0.19047	-2.7745	-0.8761656303202670	3582
**MYOF**	0.19049	-0.61497	-0.8760920430573690	3583
**MBOAT1**	0.1905	-1.9827	-0.876055251204909	3584
**PTPMT1**	0.19052	-0.52552	-0.8759816710573350	3586
**TANC1**	0.19052	-0.054173	-0.8759816710573350	3585
**ARFGAP1**	0.80944	-0.16038	-0.8758345249880370	3587
**GRAMD1C**	0.19064	-1.3953	-0.8755402897245930	3588
**HDAC1**	0.19065	-2.6469	-0.8755035156462490	3589
**FBXO15**	0.19069	-2.8548	-0.8753564311709420	3590
**FAM32A**	0.19086	-2.7348	-0.8747315332956470	3591
**MED30**	0.80914	-0.001637	-0.8747315332956460	3592
**ZNF415**	0.19112	-3.225	-0.873776467265263	3593
**KATNAL2**	0.19123	-2.7146	-0.8733726407249260	3594
**PAOX**	0.80875	-0.2022	-0.873299233019018	3595
**HIBADH**	0.19129	-3.3629	-0.8731524317231030	3596
**MPP2**	0.80868	-0.20234	-0.8730423430986460	3597
**ALOX12B**	0.19141	-0.79561	-0.872712140690686	3598
**OR5H15**	0.19141	-1.8134	-0.872712140690686	3599
**TP53BP2**	0.19141	-2.8899	-0.872712140690686	3600
**TFAP2E**	0.19148	-1.2746	-0.8724553823673840	3601
**PRSS48**	0.19157	-0.85923	-0.8721253490121520	3602
**TEX33**	0.19164	-3.5503	-0.871868722062818	3603
**ANKRD30A**	0.19172	-0.2576	-0.8715755044114120	3605
**CEP68**	0.19172	-0.089996	-0.8715755044114120	3604
**GPATCH1**	0.19194	-4.2154	-0.8707695419641530	3606
**DDX24**	0.19206	-0.054183	-0.870330164406676	3607
**TEAD2**	0.19216	-3.7916	-0.8699641447502110	3608
**PTPN3**	0.19225	-1.8732	-0.86963482668084	3609
**ATMIN**	0.19234	-3.8347	-0.8693056028966000	3611
**NUDT2**	0.19234	-2.9894	-0.8693056028966000	3610
**REV1**	0.19234	-4.0555	-0.8693056028966000	3612
**AKNAD1**	0.19238	-1.9617	-0.8691593114556300	3616

**CSAD**	0.19238	-2.4249	-0.8691593114556300	3620
**ENDOU**	0.19238	-2.4111	-0.8691593114556300	3619
**NINJ2**	0.19238	-0.59495	-0.8691593114556300	3614
**RFPL2**	0.19238	-3.3455	-0.8691593114556300	3621
**SF3B5**	0.19238	-2.2892	-0.8691593114556300	3618
**UGT1A3**	0.19238	-0.19675	-0.8691593114556300	3613
**ZCWPW2**	0.19238	-0.68552	-0.8691593114556300	3615
**ZNF605**	0.19238	-2.1686	-0.8691593114556300	3617
**SOCS6**	0.80732	-0.33608	-0.8680627179382490	3622
**KCTD8**	0.1927	-2.6136	-0.8679896488199860	3623
**MUS81**	0.19277	-1.7931	-0.8677339433922530	3625
**SCG3**	0.19277	-0.26617	-0.8677339433922530	3624
**BOD1**	0.19279	-3.6056	-0.867660895119207	3627
**MUC4**	0.19279	-3.2533	-0.867660895119207	3626
**LSM 11.00**	0.19289	-1.4821	-0.8672957231782070	3628
**C12orf54**	0.19324	-2.0438	-0.86601853107451	3631
**NFAT5**	0.19324	-1.4183	-0.86601853107451	3630
**SPTB**	0.19324	-2.7758	-0.86601853107451	3632
**THOC1**	0.19324	-1.3829	-0.86601853107451	3629
**VTA1**	0.19344	-0.62683	-0.865289340905793	3633
**GNRH2**	0.1935	-0.000314	-0.865070673560113	3634
**SPAG5**	0.1935	-0.94204	-0.865070673560113	3635
**PLET1**	0.19352	-2.0442	-0.8649977936363620	3636
**HMHB1**	0.19355	-1.0691	-0.8648884823644200	3637
**ACSL5**	0.80644	-0.16695	-0.8648520475702720	3638
**FTCD**	0.19357	-3.7832	-0.8648156139241730	3640
**GADD45A**	0.19357	-3.2835	-0.8648156139241730	3639
**GOLGA6L1**	0.19397	-3.3371	-0.8633592080963650	3641
**BIN3**	0.19405	-0.97031	-0.8630681466548290	3643
**CEP295**	0.19405	-2.6265	-0.8630681466548290	3647
**CSN2**	0.19405	-3.0297	-0.8630681466548290	3648
**OR2M3**	0.19405	-0.3938	-0.8630681466548290	3642

**PDK4**	0.19405	-2.1851	-0.8630681466548290	3645
**WDR17**	0.19405	-1.7316	-0.8630681466548290	3644
**ZNF98**	0.19405	-2.6246	-0.8630681466548290	3646
**CASP8**	0.19415	-3.1629	-0.8627044226396390	3649
**PDCD6IP**	0.19425	-2.8899	-0.8623408127201480	3650
**HTN3**	0.19434	-3.1555	-0.8620136612455710	3652
**ZC3H11A**	0.19434	-2.8879	-0.8620136612455710	3651
**FAM69A**	0.19438	-3.4046	-0.8618682902012120	3653
**CHIA**	0.1945	-3.1915	-0.8614322863056330	3654
**ENPP6**	0.19454	-0.36827	-0.8612869880608460	3655
**CHN1**	0.19477	-3.4346	-0.8604518757412890	3657
**JOSD2**	0.19477	-3.7286	-0.8604518757412890	3658
**PRAMEF4**	0.19477	-3.7494	-0.8604518757412890	3659
**RITA1**	0.19477	-2.9025	-0.8604518757412890	3656
**ZNF850**	0.19479	-2.2321	-0.8603792856387280	3660
**CCDC85B**	0.19486	-2.631	-0.8601252559734980	3662
**RPN1**	0.19486	-3.2054	-0.8601252559734980	3664
**TPH1**	0.19486	-2.6686	-0.8601252559734980	3663
**YPEL3**	0.19486	-0.29569	-0.8601252559734980	3661
**NFIC**	0.19488	-1.9096	-0.8600526862634460	3665
**VTCN1**	0.195	-2.2877	-0.8596173630802850	3666
**NCR3LG1**	0.19509	-2.6968	-0.8592909775690030	3667
**CXCL10**	0.19511	-0.061224	-0.8592184598860190	3668
**HIST1H2BE**	0.19511	-0.59647	-0.8592184598860190	3669
**UBE2M**	0.19539	-2.7601	-0.8582036863043290	3670
**CLDN22**	0.19547	-2.6501	-0.8579139132878990	3672
**TRIM34**	0.19547	-0.92958	-0.8579139132878990	3671
**CBWD7**	0.19556	-3.3865	-0.8575880047274450	3673
**ANO7**	0.19599	-3.1184	-0.8560321414789280	3674
**LRFN1**	0.19601	-2.066	-0.8559598261832960	3675
**CCDC136**	0.19604	-2.9837	-0.8558513616319840	3678
**ENDOD1**	0.19604	-2.7793	-0.8558513616319840	3677

**RIPK1**	0.19604	-1.4162	-0.8558513616319840	3676
**PNRC1**	0.19627	-2.7658	-0.8550201343135830	3680
**RBSN**	0.19627	-1.1269	-0.8550201343135830	3679
**FBXO36**	0.19628	-3.7587	-0.8549840073972120	3682
**NAA30**	0.19628	-2.9589	-0.8549840073972120	3681
**ECE1**	0.19645	-0.35111	-0.8543700204493380	3684
**ZFYVE1**	0.19645	-0.28692	-0.8543700204493380	3683
**GTF2B**	0.19649	-0.7407	-0.8542255997362090	3687
**NDUFA10**	0.19649	-0.57786	-0.8542255997362090	3686
**SCARA3**	0.19649	-0.087796	-0.8542255997362090	3685
**CD244**	0.1967	-2.5708	-0.8534676830920220	3688
**ADORA1**	0.19678	-3.1823	-0.8531790818746880	3693
**KHDC3L**	0.19678	-3.582	-0.8531790818746880	3694
**KRT19**	0.19678	-0.85513	-0.8531790818746880	3689
**RGS19**	0.19678	-1.1564	-0.8531790818746880	3690
**TTC6**	0.19678	-3.0247	-0.8531790818746880	3692
**USP26**	0.19678	-1.956	-0.8531790818746880	3691
**ZNF506**	0.19697	-2.3471	-0.8524939386073750	3695
**MAP7D1**	0.19711	-4.0893	-0.8519893521645060	3696
**UBN1**	0.19712	-1.9997	-0.8519533185765030	3697
**CACNG7**	0.19714	-0.43981	-0.851881254718855	3698
**RAPGEF6**	0.80285	-0.25193	-0.8518452244489810	3699
**COL4A6**	0.19716	-0.1237	-0.8518091952849200	3700
**KLHDC1**	0.19726	-2.5968	-0.8514489644388530	3701
**U2SURP**	0.19737	-2.1157	-0.8510528380797180	3702
**S100A6**	0.19744	-1.2295	-0.850800827186572	3703
**TMEM218**	0.80252	-0.31181	-0.8506568452193350	3704
**FNTA**	0.19759	-3.684	-0.8502609857056980	3705
**GPR39**	0.19759	-3.9862	-0.8502609857056980	3706
**BTBD6**	0.19767	-0.37524	-0.8499731715459130	3708
**CDON**	0.19767	-2.3482	-0.8499731715459130	3712
**ERVW-1**	0.19767	-2.6986	-0.8499731715459130	3714

**GNG13**	0.19767	-1.8679	-0.8499731715459130	3711
**LRRN3**	0.19767	-2.4652	-0.8499731715459130	3713
**NUPL2**	0.19767	-0.22284	-0.8499731715459130	3707
**OSBP**	0.19767	-0.72107	-0.8499731715459130	3709
**PSG4**	0.19767	-1.3204	-0.8499731715459130	3710
**CHRNA6**	0.19772	-3.5476	-0.849793323445647	3715
**FAIM**	0.19777	-0.0713	-0.8496135028280070	3716
**FHL5**	0.19779	-0.95602	-0.8495415822729030	3717
**C8orf87**	0.19789	-2.5218	-0.8491820453897670	3718
**GPALPP1**	0.1979	-1.0146	-0.8491460977388840	3719
**ECT2L**	0.19795	-1.3566	-0.8489663759411030	3720
**AKT2**	0.19812	-1.9664	-0.848355526825611	3721
**NHLH2**	0.19814	-1.1752	-0.8482836830351450	3722
**DOK3**	0.19832	-3.6772	-0.8476372858301750	3723
**MSLN**	0.19834	-1.1079	-0.8475654857807540	3724
**NOL4L**	0.80162	-0.080224	-0.8474218987884280	3725
**OR4S2**	0.19838	-1.668	-0.8474218987884280	3726
**ATP6V1B1**	0.19843	-0.95359	-0.8472424396134430	3727
**NMI**	0.19843	-2.8603	-0.8472424396134430	3730
**OR52B6**	0.19843	-1.0903	-0.8472424396134430	3728
**WNT8B**	0.19843	-2.7891	-0.8472424396134430	3729
**C4orf47**	0.1985	-3.861	-0.8469912425986890	3731
**PPP1R17**	0.19864	-1.8869	-0.8464890088316500	3732
**GH2**	0.19865	-3.0958	-0.8464531431616220	3733
**PHACTR2**	0.19868	-0.87385	-0.8463455526838760	3734
**LTC4S**	0.80128	-0.12639	-0.8462021139514810	3735
**AGT**	0.19883	-4.1385	-0.8458077471768430	3737
**PRICKLE4**	0.19883	-2.7897	-0.8458077471768430	3736
**ZNF354B**	0.80089	-0.029357	-0.84480449751366	3738
**PLEKHB2**	0.80087	-0.30373	-0.8447328693619960	3739
**PSMD14**	0.1992	-0.095876	-0.8444822049533140	3740
**UGT3A1**	0.1992	-0.38877	-0.8444822049533140	3741

**C11orf42**	0.19924	-2.2188	-0.8443389919690670	3747
**CBWD5**	0.19924	-1.4634	-0.8443389919690670	3746
**FGF3**	0.19924	-3.248	-0.8443389919690670	3749
**ITPKA**	0.19924	-2.3716	-0.8443389919690670	3748
**MRPL11**	0.19924	-0.71408	-0.8443389919690670	3744
**SHCBP1L**	0.19924	-0.18746	-0.8443389919690670	3742
**SIGLEC9**	0.19924	-0.287	-0.8443389919690670	3743
**STPG4**	0.19924	-1.3642	-0.8443389919690670	3745
**NELL2**	0.19938	-1.3078	-0.8438378828350510	3750
**INO80E**	0.19941	-1.3467	-0.8437305298732020	3751
**TBX15**	0.19952	-3.3852	-0.8433369855077230	3752
**RIPPLY1**	0.80045	-0.12999	-0.8432296778933790	3753
**NEDD4**	0.1997	-2.1204	-0.842693285394415	3755
**SHANK1**	0.1997	-1.8127	-0.842693285394415	3754
**UGT2A1**	0.19977	-2.9952	-0.8424430518542530	3756
**ABCE1**	0.20003	-1.0434	-0.8415140744344470	3757
**FBXO30**	0.20005	-2.5634	-0.8414426447144240	3761
**KCNG3**	0.20005	-3.1265	-0.8414426447144240	3763
**KRTAP10-8**	0.20005	-1.9902	-0.8414426447144240	3758
**NBPF11**	0.20005	-2.649	-0.8414426447144240	3762
**TCEAL3**	0.20005	-2.4903	-0.8414426447144240	3760
**WDR53**	0.20005	-2.1462	-0.8414426447144240	3759
**EIF4E1B**	0.20012	-3.1107	-0.8411926744955550	3764
**LMO7DN**	0.20012	-3.8821	-0.8411926744955550	3767
**MYC**	0.20012	-3.7751	-0.8411926744955550	3766
**PTPN7**	0.20012	-3.5637	-0.8411926744955550	3765
**BANK1**	0.2002	-2.84	-0.8409070585912860	3768
**PSD**	0.79978	-0.1369	-0.8408356653333010	3769
**S1PR2**	0.20036	-3.4516	-0.8403360324715700	3770
**CDKN1B**	0.20048	-4.0747	-0.8399079426383750	3771
**C8orf86**	0.20074	-3.0665	-0.8389809422632030	3775
**CST9L**	0.20074	-0.20663	-0.8389809422632030	3772

**FLYWCH1**	0.20074	-2.0066	-0.8389809422632030	3773
**LRRC30**	0.20074	-2.447	-0.8389809422632030	3774
**RAPGEF3**	0.20074	-3.0807	-0.8389809422632030	3776
**TNS2**	0.20075	-2.5505	-0.8389453028015780	3777
**SLC24A4**	0.79916	-0.37455	-0.838624595584587	3778
**CARD6**	0.20098	-2.3713	-0.8381258890614790	3779
**PPIP5K1**	0.201	-3.0104	-0.8380546622885190	3781
**SLC14A1**	0.201	-0.97373	-0.8380546622885190	3780
**DRGX**	0.20124	-2.217	-0.8372002723743720	3782
**DEFB4B**	0.20149	-3.1497	-0.8363109322615	3783
**CHRNE**	0.20151	-3.4183	-0.8362398136286100	3788
**EIF3G**	0.20151	-2.4596	-0.8362398136286100	3787
**RPP30**	0.20151	-0.53978	-0.8362398136286100	3784
**TRIM51**	0.20151	-0.64097	-0.8362398136286100	3785
**ZNF439**	0.20151	-1.9368	-0.8362398136286100	3786
**RTL6**	0.20166	-0.30687	-0.8357065586322360	3790
**ZCWPW1**	0.20166	-0.28592	-0.8357065586322360	3789
**ZC3H15**	0.20198	-2.5819	-0.8345697414140450	3791
**SLC25A21**	0.20212	-1.4168	-0.8340727229226030	3792
**TEK**	0.2022	-0.5022	-0.8337888048436900	3793
**ANXA7**	0.20223	-3.281	-0.8336823528908260	3795
**APBA1**	0.20223	-0.095433	-0.8336823528908260	3794
**PDZRN3**	0.2024	-1.9994	-0.8330793035063590	3796
**PRKCH**	0.20242	-0.026219	-0.8330083764381510	3797
**SETSIP**	0.2026	-1.6858	-0.8323702212865820	3798
**SHISA8**	0.20261	-4.1689	-0.8323347781621540	3799
**NECAP2**	0.2027	-3.4735	-0.8320158370797550	3800
**ACTL8**	0.20287	-3.0038	-0.8314136236081280	3801
**GLS2**	0.20291	-1.6024	-0.8312719701376950	3803
**OR6C76**	0.20291	-0.5252	-0.8312719701376950	3802
**SDSL**	0.20291	-2.4587	-0.8312719701376950	3804
**ABTB1**	0.79703	-0.24313	-0.8310595212013330	3805

**EEF2K**	0.20299	-3.1313	-0.830988713224305	3806
**CCNH**	0.20304	-2.6914	-0.8308117115067370	3807
**RHCE**	0.20304	-4.0045	-0.8308117115067370	3809
**UFM1**	0.20304	-3.0471	-0.8308117115067370	3808
**ITGB2**	0.20305	-1.5468	-0.8307763142866640	3810
**CTSS**	0.20312	-1.756	-0.8305285628854650	3811
**SRP72**	0.20319	-3.1406	-0.8302808624522080	3812
**GOLGA8B**	0.20321	-0.005982	-0.8302101002562460	3813
**CLK4**	0.20323	-2.2336	-0.8301393422171390	3814
**ESRRG**	0.20336	-1.2495	-0.829679516228081	3815
**PDE12**	0.20343	-1.0175	-0.8294319902677670	3816
**ZNF221**	0.79647	-0.090808	-0.8290784698805730	3817
**RNF126**	0.20356	-1.8913	-0.8289724339682650	3818
**CCDC182**	0.20374	-3.2712	-0.8283364141116290	3819
**BLOC1S3**	0.20377	-2.9219	-0.8282304433786730	3825
**FAM129B**	0.20377	-0.96486	-0.8282304433786730	3821
**MICAL1**	0.20377	-0.38341	-0.8282304433786730	3820
**NDUFA11**	0.20377	-1.7069	-0.8282304433786730	3823
**PAGR1**	0.20377	-1.0165	-0.8282304433786730	3822
**TMEM200A**	0.20377	-1.7217	-0.8282304433786730	3824
**MTRNR2L8**	0.20422	-1.8261	-0.8266419968105280	3826
**ESPN**	0.20434	-3.7317	-0.82621876324809	3827
**METTL11B**	0.2044	-0.80675	-0.8260072019578020	3828
**SAMD13**	0.20441	-2.7875	-0.8259719453370900	3829
**NRROS**	0.20445	-2.3354	-0.8258309291199700	3830
**UGT2B28**	0.20452	-0.77709	-0.8255841902471220	3831
**EIF1AY**	0.20454	-0.43085	-0.8255137026577000	3832
**HERC6**	0.20454	-0.48827	-0.8255137026577000	3833
**SLC15A3**	0.20456	-1.3576	-0.8254432191696010	3834
**C11orf91**	0.20464	-2.3018	-0.825161326213893	3835
**SIK2**	0.20467	-2.6774	-0.8250556332592470	3836
**UBE2K**	0.20477	-1.7797	-0.82470338995395	3837

**KLC3**	0.79508	-0.009938	-0.8241752167679850	3838
**CEP57L1**	0.20509	-0.98283	-0.8235768982956310	3839
**LYG2**	0.20511	-2.8687	-0.823506527270479	3840
**NUP50**	0.20522	-2.4249	-0.8231195595013760	3841
**PIAS1**	0.20526	-2.2556	-0.8229788745060460	3842
**GNG3**	0.20536	-2.6651	-0.8226272332577870	3843
**COG1**	0.20538	-0.17741	-0.8225569172141670	3844
**ZNF76**	0.2054	-2.9091	-0.8224866052373140	3845
**MAP7**	0.20541	-0.30045	-0.8224514507736700	3846
**AKT1S1**	0.20545	-0.91417	-0.8223108430819250	3848
**KLK5**	0.20545	-2.8414	-0.8223108430819250	3851
**PAQR9**	0.20545	-1.5625	-0.8223108430819250	3850
**SMPD1**	0.20545	-1.5434	-0.8223108430819250	3849
**SYT7**	0.20545	-2.9891	-0.8223108430819250	3852
**ZNF625**	0.20545	-0.077587	-0.8223108430819250	3847
**SSBP1**	0.20551	-2.9329	-0.8220999620215580	3853
**CELSR1**	0.20554	-0.83307	-0.8219945352001640	3854
**FER**	0.20558	-3.0664	-0.821853980315302	3855
**HFM1**	0.20562	-2.0044	-0.8217134416648330	3856
**AFG1L**	0.20567	-3.0596	-0.8215377911714220	3860
**CHPT1**	0.20567	-2.4677	-0.8215377911714220	3858
**MTRNR2L7**	0.20567	-2.7987	-0.8215377911714220	3859
**PLA2G10**	0.20567	-1.4878	-0.8215377911714220	3857
**C11orf84**	0.20571	-3.1422	-0.821397289024487	3861
**DGCR6L**	0.79423	-0.40535	-0.8211865662017980	3862
**STXBP2**	0.20579	-1.218	-0.8211163333637030	3863
**ASH1L**	0.20598	-3.1867	-0.8204493232828640	3866
**COL14A1**	0.20598	-2.9231	-0.8204493232828640	3865
**MBD1**	0.20598	-1.9328	-0.8204493232828640	3864
**AGXT2**	0.20628	-1.5998	-0.8193968919755950	3868
**C1orf112**	0.20628	-1.5045	-0.8193968919755950	3867
**FOXF1**	0.7937	-0.15856	-0.8193267621600780	3869

**GPSM1**	0.20638	-0.43182	-0.8190462831836410	3870
**EPB41L2**	0.20643	-1.609	-0.8188710165390460	3872
**IL24**	0.20643	-3.0272	-0.8188710165390460	3873
**LMO2**	0.20643	-0.8672	-0.8188710165390460	3871
**ZNF716**	0.20643	-3.5888	-0.8188710165390460	3874
**PXK**	0.20656	-1.3458	-0.818415440937295	3875
**ZNF384**	0.20666	-2.0091	-0.8180651137212980	3876
**H2AFX**	0.20667	-3.0363	-0.8180300865217950	3877
**MARF1**	0.20675	-1.841	-0.8177499050474900	3878
**CCDC97**	0.20686	-3.6982	-0.8173647603037010	3879
**OR2F2**	0.7931	-0.28664	-0.817224737727876	3880
**SLC4A9**	0.20691	-1.475	-0.8171897345874400	3881
**ILKAP**	0.20705	-2.1284	-0.8166997957025950	3882
**FAM98C**	0.20708	-0.21285	-0.8165948343031260	3883
**F13B**	0.20713	-1.5708	-0.8164199186261490	3890
**GNPTAB**	0.20713	-2.3188	-0.8164199186261490	3892
**KRTAP2-4**	0.20713	-2.8669	-0.8164199186261490	3894
**MAP3K6**	0.20713	-1.1921	-0.8164199186261490	3887
**MMP19**	0.20713	-3.0851	-0.8164199186261490	3895
**MMP23B**	0.20713	-1.518	-0.8164199186261490	3889
**MTRNR2L5**	0.20713	-1.4811	-0.8164199186261490	3888
**MYO1G**	0.20713	-0.77674	-0.8164199186261490	3884
**NEK3**	0.20713	-0.77973	-0.8164199186261490	3885
**OR8G1**	0.20713	-0.99234	-0.8164199186261490	3886
**PLB1**	0.20713	-2.3458	-0.8164199186261490	3893
**WIZ**	0.20713	-1.861	-0.8164199186261490	3891
**ZNF512**	0.79257	-0.33727	-0.8153709486067520	3896
**ZBTB12**	0.20745	-0.2538	-0.815301049174424	3897
**FZR1**	0.7925	-0.17664	-0.8151263180186890	3898
**COL8A1**	0.20756	-2.0661	-0.8149166734763310	3899
**PRSS3**	0.20764	-1.6619	-0.8146372031207870	3900
**RGS22**	0.2078	-2.8595	-0.8140784531938620	3901

**BRIP1**	0.20794	-2.3237	-0.8135897554144940	3902
**MYH3**	0.20794	-4.1717	-0.8135897554144940	3903
**TTC23L**	0.20808	-0.67321	-0.8131012518637810	3904
**KLF7**	0.79186	-0.21793	-0.8128919525898140	3905
**RNH1**	0.20824	-1.0497	-0.8125431995751760	3906
**HEXIM2**	0.20829	-0.69201	-0.812368860124187	3907
**ANOS1**	0.20835	-2.9868	-0.812159685369849	3914
**BRD2**	0.20835	-1.8733	-0.812159685369849	3912
**FAM46D**	0.20835	-2.929	-0.812159685369849	3913
**LYSMD4**	0.20835	-0.17387	-0.812159685369849	3908
**NKAP**	0.20835	-0.58376	-0.812159685369849	3910
**SLC43A1**	0.20835	-3.2511	-0.812159685369849	3915
**SOX7**	0.20835	-0.29002	-0.812159685369849	3909
**YIPF1**	0.20835	-1.223	-0.812159685369849	3911
**MAP3K2**	0.20843	-1.3103	-0.8118808409614370	3916
**TOB2**	0.79151	-0.086755	-0.8116717490756640	3917
**AGAP3**	0.20852	-0.4353	-0.8115672164390160	3918
**HDX**	0.79129	-0.47237	-0.8109053822160280	3919
**NME6**	0.20871	-2.4742	-0.8109053822160280	3920
**WAS**	0.20873	-1.9637	-0.8108357361186510	3921
**FAM118A**	0.20875	-1.5514	-0.8107660939540730	3922
**ZNRF3**	0.20881	-3.123	-0.8105571910493090	3923
**RPRM**	0.79103	-0.47343	-0.8100002894849250	3924
**SIAH1**	0.20898	-2.5353	-0.8099654914785820	3925
**PURA**	0.20902	-3.3339	-0.8098263092598350	3926
**ALG11**	0.20903	-3.7823	-0.8097915161563190	3928
**GRIP1**	0.20903	-1.8673	-0.8097915161563190	3927
**RPS27A**	0.20919	-3.2045	-0.8092349597510090	3929
**KLLN**	0.20929	-1.7436	-0.8088872392561880	3930
**ZNF629**	0.2094	-1.3949	-0.8085048596378470	3931
**BTN1A1**	0.20945	-1.4364	-0.8083310897953640	3933
**C17orf50**	0.20945	-2.5122	-0.8083310897953640	3936

**IL1RAP**	0.20945	-0.12941	-0.8083310897953640	3932
**SLCO1A2**	0.20945	-1.761	-0.8083310897953640	3934
**ZDHHC11B**	0.20945	-2.4792	-0.8083310897953640	3935
**LOC1001297**	0.20954	-3.0067	-0.808018365571086	3937
**STAG1**	0.79041	-0.075944	-0.8078446640318120	3938
**NDUFA7**	0.20964	-2.564	-0.8076709868635750	3940
**PRKAA2**	0.20964	-1.6501	-0.8076709868635750	3939
**ACVR2A**	0.20967	-0.64512	-0.8075667922557070	3941
**TRIM14**	0.20969	-0.11734	-0.8074973340543000	3942
**EMILIN2**	0.20989	-2.2129	-0.8068029661655950	3943
**PRR23B**	0.2099	-2.5412	-0.8067682579830210	3944
**RNF40**	0.21015	-0.95668	-0.8059008690205680	3945
**MAML2**	0.21022	-2.5915	-0.8056581087540090	3948
**MAS1**	0.21022	-1.6878	-0.8056581087540090	3946
**PARP11**	0.21022	-2.9592	-0.8056581087540090	3949
**SRSF12**	0.21022	-1.8689	-0.8056581087540090	3947
**TMEM67**	0.21022	-3.3447	-0.8056581087540090	3950
**FABP12**	0.21027	-1.3867	-0.8054847376288970	3951
**NOC2L**	0.21052	-2.8742	-0.8046182448766440	3952
**DERL3**	0.2107	-2.9845	-0.8039947439885360	3954
**FAM237A**	0.7893	-0.48539	-0.8039947439885360	3953
**TRIM44**	0.21086	-1.2252	-0.8034407832549600	3955
**AKAP10**	0.21096	-2.2774	-0.8030946829685570	3964
**ATP2A2**	0.21096	-0.34758	-0.8030946829685570	3957
**BAIAP2**	0.21096	-0.6462	-0.8030946829685570	3960
**CA6**	0.21096	-0.2713	-0.8030946829685570	3956
**CRCT1**	0.21096	-1.9715	-0.8030946829685570	3963
**FCGRT**	0.21096	-0.83428	-0.8030946829685570	3961
**NSD3**	0.21096	-0.64595	-0.8030946829685570	3959
**PHKG2**	0.21096	-3.3501	-0.8030946829685570	3965
**SLC37A3**	0.21096	-0.44354	-0.8030946829685570	3958
**ZCRB1**	0.21096	-1.6727	-0.8030946829685570	3962

**PRKAG1**	0.21107	-3.176	-0.8027140837288040	3966
**TSPAN7**	0.21111	-1.4184	-0.8025757128322710	3967
**POLDIP2**	0.21113	-1.2514	-0.8025065331461850	3968
**CYB5D2**	0.2113	-2.4582	-0.8019186607959600	3969
**HSF5**	0.21134	-3.2477	-0.80178037816436	3971
**INSM2**	0.21134	-2.6746	-0.80178037816436	3970
**FBXO44**	0.21145	-1.7942	-0.8014001799546330	3972
**PGF**	0.78854	-0.33935	-0.8013656222254330	3973
**RPL35A**	0.2115	-1.0061	-0.8012274008776220	3974
**PDXK**	0.21152	-2.547	-0.80115829594379	3975
**HYKK**	0.78841	-0.15451	-0.8009164587979100	3976
**TRAM1**	0.21161	-1.9533	-0.8008473710738460	3977
**ATP5L2**	0.21167	-0.32846	-0.8006401308325770	3978
**TAS2R1**	0.21167	-1.4175	-0.8006401308325770	3979
**CHAC1**	0.21175	-2.7147	-0.8003638639882860	3980
**SLC52A1**	0.21181	-3.1215	-0.8001567039368080	3981
**DFFA**	0.21183	-1.9136	-0.800087658216927	3982
**HNRNPH3**	0.21194	-0.4909	-0.7997079749136870	3983
**RPGRIP1L**	0.21196	-1.2817	-0.7996389539717520	3984
**AGMAT**	0.21211	-2.9212	-0.7991214182731340	3985
**STAT2**	0.21211	-3.1093	-0.7991214182731340	3986
**TRIM22**	0.21214	-1.6317	-0.7990179368175840	3987
**CCDC106**	0.21224	-1.2692	-0.7986730604079830	3988
**BPI**	0.21226	-0.85914	-0.7986040965251810	3989
**IRF5**	0.21231	-3.7743	-0.7984317034326330	3991
**SMARCA5**	0.21231	-0.61948	-0.7984317034326330	3990
**TRAF1**	0.21236	-0.18182	-0.7982593340657030	3992
**CASP2**	0.21238	-2.4492	-0.7981903929594930	3997
**NUF2**	0.21238	-0.85314	-0.7981903929594930	3993
**PIANP**	0.21238	-1.0908	-0.7981903929594930	3996
**SCML4**	0.21238	-0.86129	-0.7981903929594930	3994
**SPRYD7**	0.21238	-1.0345	-0.7981903929594930	3995

**PRPF39**	0.21245	-1.5072	-0.797949128956602	3998
**OSBPL8**	0.21248	-0.81491	-0.7978457443158920	3999
**MAG**	0.2125	-2.3611	-0.7977768259594480	4000
**DEFB133**	0.21267	-1.3829	-0.7971911728364	4001
**CAPS**	0.21274	-1.3375	-0.7969501010132570	4002
**SMIM25**	0.21286	-1.3735	-0.7965369427462250	4003
**SLC38A8**	0.21291	-1.2438	-0.7963648335938940	4004
**ASPA**	0.21308	-1.0748	-0.7957798388325310	4005
**IMPG1**	0.21328	-2.7044	-0.7950919581639460	4006
**C8orf48**	0.21335	-0.53715	-0.7948512888013370	4008
**KHK**	0.21335	-2.4385	-0.7948512888013370	4011
**KLRK1**	0.21335	-2.4403	-0.7948512888013370	4012
**MLLT3**	0.21335	-2.1971	-0.7948512888013370	4010
**OPRD1**	0.21335	-0.41396	-0.7948512888013370	4007
**RFX3**	0.21335	-0.93443	-0.7948512888013370	4009
**SFTPA2**	0.21335	-3.6189	-0.7948512888013370	4013
**FIZ1**	0.21339	-0.073192	-0.794713784119984	4014
**RECQL4**	0.2135	-1.2271	-0.7943357236983040	4015
**TARP**	0.21353	-2.3965	-0.7942326360140640	4016
**ELFN2**	0.2136	-3.1798	-0.7939921309008350	4017
**STPG2**	0.21372	-3.1437	-0.793579943232933	4018
**MST1R**	0.21375	-2.3229	-0.7934769173821410	4019
**MON1A**	0.21387	-1.1032	-0.7930648981695880	4020
**LMNA**	0.21395	-1.6637	-0.7927902934745930	4021
**RNPEPL1**	0.21397	-1.6271	-0.7927216516416520	4022
**LRRC49**	0.78602	-0.28663	-0.7926873321258010	4023
**RRM2B**	0.21411	-0.50744	-0.7922412633463700	4024
**ZFYVE16**	0.21414	-1.9164	-0.7921383467831100	4025
**EMC6**	0.2142	-1.1943	-0.7919325388226310	4026
**MMD**	0.2143	-2.4541	-0.7915896000751130	4027
**POLL**	0.78564	-0.27861	-0.7913838815065750	4028
**SLFN12**	0.21441	-1.5494	-0.7912124749465450	4029

**C15orf39**	0.21443	-2.6421	-0.7911439188311590	4034
**HPS1**	0.21443	-3.0377	-0.7911439188311590	4035
**L3MBTL2**	0.21443	-1.6723	-0.7911439188311590	4031
**OR6C68**	0.21443	-0.1003	-0.7911439188311590	4030
**PDCD1LG2**	0.21443	-1.7741	-0.7911439188311590	4032
**SASH1**	0.21443	-3.3968	-0.7911439188311590	4036
**TRDN**	0.21443	-2.606	-0.7911439188311590	4033
**GID4**	0.21455	-2.0822	-0.7907326601941230	4037
**TRIM69**	0.21457	-1.7047	-0.7906641300903960	4038
**USP9X**	0.21463	-1.1303	-0.7904585620546390	4039
**CAPN12**	0.78515	-0.14855	-0.7897050981795670	4040
**EIF3CL**	0.21486	-2.8715	-0.7896708604748060	4041
**SOHLH2**	0.21498	-0.019662	-0.7892600801920440	4042
**CTBP1**	0.21499	-1.5533	-0.7892258545136110	4043
**ASS1**	0.21502	-1.4194	-0.7891231830247810	4044
**SEM1**	0.78497	-0.01573	-0.7890889610436940	4045
**SPTSSB**	0.21514	-1.9778	-0.7887125802233070	4046
**ASB11**	0.21543	-1.5143	-0.7877208386136410	4051
**CHRM3**	0.21543	-2.3706	-0.7877208386136410	4054
**FZD9**	0.21543	-1.3419	-0.7877208386136410	4049
**GFRA1**	0.21543	-0.65804	-0.7877208386136410	4047
**MUCL1**	0.21543	-2.2625	-0.7877208386136410	4052
**PTER**	0.21543	-3.0665	-0.7877208386136410	4055
**SLC18B1**	0.21543	-1.3939	-0.7877208386136410	4050
**SLC22A10**	0.21543	-0.74615	-0.7877208386136410	4048
**SUZ12**	0.21543	-2.3226	-0.7877208386136410	4053
**MATN4**	0.78454	-0.43149	-0.7876182888763220	4056
**MPP3**	0.21547	-1.5356	-0.7875841074711740	4057
**ZNF131**	0.21569	-2.4995	-0.7868323492008920	4058
**KRT79**	0.78429	-0.1219	-0.7867640295909870	4059
**NPHP4**	0.21571	-2.6656	-0.7867640295909870	4060
**GPR88**	0.2158	-1.8151	-0.7864566367780660	4061

**FADD**	0.21584	-2.3099	-0.7863200416016020	4062
**SMIM20**	0.21603	-0.94101	-0.7856714147502110	4063
**NOB1**	0.21618	-2.8397	-0.7851595743189620	4064
**BCLAF1**	0.21625	-0.70695	-0.7849207858349000	4065
**TBC1D29**	0.21625	-2.7411	-0.7849207858349000	4068
**TIMM10B**	0.21625	-2.643	-0.7849207858349000	4067
**ZNF441**	0.21625	-1.318	-0.7849207858349000	4066
**UGCG**	0.21627	-3.2548	-0.7848525687740400	4069
**C3orf84**	0.21639	-3.4391	-0.7844433430796160	4070
**AGPS**	0.21646	-3.3027	-0.7842046887476790	4075
**AQP12B**	0.21646	-0.9714	-0.7842046887476790	4072
**AXDND1**	0.21646	-2.3962	-0.7842046887476790	4074
**KCNE4**	0.21646	-0.53274	-0.7842046887476790	4071
**NSA2**	0.21646	-2.0871	-0.7842046887476790	4073
**LRP8**	0.21648	-1.0187	-0.7841365099992750	4076
**PRMT9**	0.21654	-2.8716	-0.7839319956196660	4077
**GMNN**	0.21663	-2.1421	-0.7836252855135070	4078
**ADGRL1**	0.21665	-1.9012	-0.7835571377228730	4079
**RBM4**	0.2168	-1.5089	-0.7830461452292620	4080
**COLQ**	0.21686	-1.4027	-0.7828418054753890	4081
**GNB1L**	0.21695	-1.747	-0.7825353571176170	4082
**CHST9**	0.217	-1.5719	-0.7823651397806720	4083
**C3orf67**	0.78294	-0.098672	-0.7821609088934070	4084
**SREK1**	0.78291	-0.49385	-0.7820588056830510	4085
**SMPX**	0.21741	-2.9437	-0.7809702115566980	4086
**BTBD9**	0.21743	-1.9693	-0.780902205156161	4091
**DDX4**	0.21743	-2.6448	-0.780902205156161	4092
**NR1H2**	0.21743	-0.47957	-0.780902205156161	4087
**RSPO1**	0.21743	-0.53537	-0.780902205156161	4088
**SLC19A1**	0.21743	-2.8125	-0.780902205156161	4094
**SMOX**	0.21743	-1.9354	-0.780902205156161	4090
**WBSCR28**	0.21743	-2.6858	-0.780902205156161	4093

**ZNF804B**	0.21743	-1.6192	-0.780902205156161	4089
**LEMD2**	0.21744	-1.9235	-0.7808682033102040	4095
**SCG5**	0.21752	-3.1382	-0.7805962210344860	4096
**SIRPG**	0.21752	-3.9999	-0.7805962210344860	4098
**SLC36A3**	0.21752	-3.5963	-0.7805962210344860	4097
**MAFK**	0.21755	-1.8977	-0.7804942425674760	4099
**SSTR4**	0.21757	-1.1596	-0.7804262614319410	4100
**KIAA1549**	0.21762	-1.9222	-0.7802563243700030	4101
**PRDM13**	0.21763	-1.7835	-0.7802223396615310	4102
**FAM198A**	0.21768	-0.201	-0.7800524296339540	4103
**FAM71F2**	0.21782	-1.5695	-0.7795768013107340	4104
**WDR27**	0.21784	-0.65682	-0.7795088688041780	4105
**R3HCC1**	0.2179	-1.8721	-0.7793050928643710	4106
**FGD4**	0.78207	-0.18888	-0.7792032170288440	4107
**KRT20**	0.21799	-2.3909	-0.7789994896148340	4108
**NR2F2**	0.2184	-0.97995	-0.7776082165128050	4109
**AKAP5**	0.21857	-0.81106	-0.7770317884737040	4112
**CDX4**	0.21857	-1.0822	-0.7770317884737040	4114
**FUCA2**	0.21857	-0.73095	-0.7770317884737040	4110
**STEAP1**	0.21857	-0.85988	-0.7770317884737040	4113
**TIGD5**	0.21857	-0.7724	-0.7770317884737040	4111
**IQCF3**	0.2186	-3.6765	-0.7769300926796920	4115
**CPLX2**	0.21877	-1.3247	-0.7763539682063830	4116
**CXCL11**	0.78117	-0.042855	-0.7761506916750420	4117
**SLC2A1**	0.21899	-1.7821	-0.7756087776442340	4118
**CSNK1E**	0.219	-3.302	-0.7755749155805640	4119
**TSG101**	0.219	-4.0662	-0.7755749155805640	4120
**SH3BP2**	0.21906	-2.6542	-0.7753717618704540	4121
**TMEM214**	0.2191	-2.5634	-0.7752363438402180	4122
**PODN**	0.21915	-3.0378	-0.775067091291378	4123
**KIT**	0.21918	-0.95055	-0.7749655504187500	4124
**GCN1**	0.21934	-2.0419	-0.7744241339788770	4125

**LPP**	0.21934	-3.5079	-0.7744241339788770	4126
**IL13RA1**	0.21935	-0.51139	-0.7743903029892310	4127
**KIAA0355**	0.21951	-2.1284	-0.7738491276330100	4128
**SMAD2**	0.21961	-0.84565	-0.773511008098779	4129
**ANKRD33**	0.21962	-2.1873	-0.7734772010092170	4131
**CPED1**	0.21962	-2.2382	-0.7734772010092170	4132
**OR2M7**	0.21962	-1.5513	-0.7734772010092170	4130
**PRPS1**	0.21962	-2.4519	-0.7734772010092170	4133
**ZBTB14**	0.21962	-2.7518	-0.7734772010092170	4134
**FBXL22**	0.21963	-2.9905	-0.7734433948036540	4135
**SH3RF2**	0.21969	-2.8682	-0.7732405761294990	4136
**C5orf15**	0.2197	-1.3747	-0.7732067761095510	4137
**CAP2**	0.21979	-1.7495	-0.7729026156692660	4138
**TMEM170A**	0.21999	-1.1213	-0.7722269594314750	4139
**ETFDH**	0.22014	-2.77	-0.77172044851749	4140
**CSK**	0.22025	-1.3588	-0.7713491329794820	4141
**ABCG8**	0.77972	-0.40535	-0.7712478835623360	4142
**HMGA2**	0.22039	-3.2286	-0.7708767033205900	4144
**TMEM184A**	0.22039	-2.4074	-0.7708767033205900	4143
**SLC22A15**	0.77955	-0.35291	-0.7706742861299680	4145
**USP17L25**	0.22047	-3.9732	-0.7706068207499220	4146
**FAM83F**	0.22051	-2.3795	-0.770471900511041	4149
**FBXO31**	0.22051	-2.6957	-0.770471900511041	4150
**LRRD1**	0.22051	-1.2555	-0.770471900511041	4148
**PDIA5**	0.22051	-2.8385	-0.770471900511041	4152
**TGS1**	0.22051	-2.8013	-0.770471900511041	4151
**TMEM177**	0.22051	-0.82778	-0.770471900511041	4147
**CCPG1**	0.22056	-1.4947	-0.7703032699327850	4153
**C6orf136**	0.7793	-0.11995	-0.7698312207972970	4154
**DYNLRB1**	0.22076	-0.99235	-0.7696289665320550	4155
**CISD2**	0.22078	-1.4495	-0.7695615554396770	4156
**AVPR2**	0.22096	-1.1797	-0.7689550128886460	4157

**MFSD6**	0.22104	-0.66482	-0.7686855292135450	4158
**PLA2G7**	0.22104	-2.186	-0.7686855292135450	4159
**TLX3**	0.22116	-2.6266	-0.7682814083341140	4160
**KCNIP1**	0.22122	-0.99304	-0.7680793949403590	4161
**CTH**	0.22128	-0.45682	-0.7678774128866090	4162
**CLPTM1L**	0.22131	-1.0151	-0.767776433606622	4163
**PDCD10**	0.22142	-2.2236	-0.7674062432047910	4164
**SLC9B1**	0.77857	-0.11491	-0.7673725947469210	4165
**AATK**	0.22151	-0.56624	-0.7671034383542040	4166
**COX7B2**	0.22151	-2.9368	-0.7671034383542040	4170
**DCBLD2**	0.22151	-2.4604	-0.7671034383542040	4169
**SRR**	0.22151	-3.3584	-0.7671034383542040	4171
**SV2A**	0.22151	-2.3787	-0.7671034383542040	4168
**USP17L11**	0.22151	-1.9167	-0.7671034383542040	4167
**PHYH**	0.22153	-2.5359	-0.7670361579391610	4172
**ERBB3**	0.22154	-3.2525	-0.7670025190336540	4173
**LOC283713**	0.2216	-2.7746	-0.766800703823596	4174
**LCE1A**	0.22172	-1.8083	-0.7663971670650560	4175
**TMEM170B**	0.22176	-1.5877	-0.7662626825443740	4176
**RMDN3**	0.22179	-2.6544	-0.7661618282479820	4177
**IPMK**	0.22187	-2.9409	-0.7658929215481230	4178
**CNTLN**	0.22202	-1.8829	-0.7653888706946730	4179
**CACNA2D1**	0.22211	-3.1485	-0.7650865335053200	4181
**FNDC3B**	0.22211	-2.9855	-0.7650865335053200	4180
**KLRG2**	0.22224	-1.475	-0.7646499476528300	4183
**WIF1**	0.22224	-0.95865	-0.7646499476528300	4182
**INTS13**	0.22229	-2.6845	-0.764482068822745	4186
**RASIP1**	0.22229	-1.1221	-0.764482068822745	4185
**ZNF587B**	0.22229	-0.84484	-0.764482068822745	4184
**UPK3BL**	0.22235	-2.4209	-0.7642806426623220	4187
**ACOT7**	0.22236	-0.76583	-0.7642470746503420	4188
**UAP1L1**	0.22241	-1.0627	-0.7640792475059030	4189

**PLA2G1B**	0.22243	-3.0804	-0.7640121226738140	4190
**TRPC4**	0.22253	-2.8267	-0.763676550134251	4191
**ITGA8**	0.22258	-0.95669	-0.7635087961101230	4192
**F9**	0.22264	-0.92447	-0.7633075196384450	4193
**NCAM2**	0.22274	-2.4107	-0.7629721275514500	4194
**TSPY1**	0.77705	-0.27887	-0.7622680833606520	4195
**P3H3**	0.22299	-0.27773	-0.7621340225443220	4196
**C11orf65**	0.223	-1.2682	-0.7621005094804280	4197
**CDC42BPA**	0.2231	-3.1614	-0.7617654259032680	4206
**COX17**	0.2231	-2.6795	-0.7617654259032680	4203
**EEF1A2**	0.2231	-2.5005	-0.7617654259032680	4202
**GDAP1L1**	0.2231	-2.7596	-0.7617654259032680	4204
**HMOX2**	0.2231	-0.87769	-0.7617654259032680	4200
**KCNJ3**	0.2231	-0.45439	-0.7617654259032680	4198
**KRTAP2-2**	0.2231	-0.55303	-0.7617654259032680	4199
**LRRC14**	0.2231	-1.4732	-0.7617654259032680	4201
**SCML2**	0.2231	-2.8895	-0.7617654259032680	4205
**BFSP1**	0.22313	-1.6503	-0.7616649175082600	4207
**IQCD**	0.22317	-3.0154	-0.7615309182825200	4208
**FAM222B**	0.2235	-0.85239	-0.7604259458827500	4209
**MYNN**	0.22354	-3.6107	-0.7602920729296370	4210
**FMR1**	0.22358	-1.5805	-0.7601582136010680	4211
**FOXP1**	0.22359	-3.9397	-0.760124750897155	4212
**ATG16L2**	0.22372	-3.2907	-0.7596898131697340	4213
**OR51B2**	0.22375	-0.99458	-0.7595894633333410	4214
**MFSD7**	0.2238	-0.86518	-0.7594222306017270	4215
**GOLGA8M**	0.22404	-0.44864	-0.7586198089163620	4216
**CD33**	0.22414	-0.47134	-0.7582856106872050	4218
**CHIT1**	0.22414	-2.767	-0.7582856106872050	4222
**CMC4**	0.22414	-2.3616	-0.7582856106872050	4221
**PTGDS**	0.22414	-2.1663	-0.7582856106872050	4220
**SFSWAP**	0.22414	-0.18236	-0.7582856106872050	4217

**SNX31**	0.22414	-1.5776	-0.7582856106872050	4219
**C9orf139**	0.22434	-1.2768	-0.7576174681596410	4223
**TRPC5**	0.22437	-1.8419	-0.7575172759521300	4224
**SLC35B4**	0.2244	-0.89135	-0.7574170913483650	4225
**TRIM39**	0.22452	-3.1328	-0.7570164289275730	4226
**COQ4**	0.22457	-3.0001	-0.7568495221157890	4227
**EFHB**	0.22458	-2.1309	-0.7568161432835400	4229
**OR13H1**	0.77542	-0.14443	-0.7568161432835400	4228
**IL2RG**	0.7754	-0.11576	-0.756749388148509	4230
**KLF14**	0.22468	-0.4811	-0.7564824013229400	4231
**LY6H**	0.22477	-2.81	-0.756182105584635	4232
**ZNF687**	0.22487	-1.2132	-0.7558485236091950	4233
**TUBA3C**	0.22496	-0.70265	-0.755548371728225	4234
**CLTB**	0.22502	-0.10053	-0.7553483082849320	4235
**SMAP2**	0.22515	-1.6035	-0.7549149411689640	4236
**ATP10D**	0.22517	-2.612	-0.7548482818890910	4240
**GTF2IRD2B**	0.22517	-0.8399	-0.7548482818890910	4238
**HOXA9**	0.22517	-0.04536	-0.7548482818890910	4237
**SLCO1B1**	0.22517	-3.0434	-0.7548482818890910	4242
**SYNGR2**	0.22517	-2.3359	-0.7548482818890910	4239
**TSHZ2**	0.22517	-2.9911	-0.7548482818890910	4241
**RASL12**	0.22524	-2.0484	-0.754615000817891	4243
**KRT80**	0.22529	-2.5305	-0.7544483966210500	4244
**CYP24A1**	0.22531	-2.7332	-0.7543817608056640	4245
**MYF6**	0.22533	-1.029	-0.7543151283398180	4246
**RBM22**	0.22535	-1.8509	-0.7542484992228760	4247
**IFIT5**	0.22536	-3.8929	-0.7542151859200460	4248
**COQ6**	0.22538	-0.98514	-0.7541485618252770	4249
**AGK**	0.22573	-1.8923	-0.7529831814528330	4250
**MED7**	0.77423	-0.045167	-0.7528500602456280	4251
**MTERF3**	0.2258	-2.9593	-0.7527502280949510	4252
**DNAJC10**	0.22583	-3.2121	-0.7526504034459650	4253

**CCDC3**	0.22593	-1.1455	-0.7523177087676590	4254
**TXN**	0.22594	-1.4539	-0.7522844438798750	4255
**BLOC1S2**	0.22617	-2.9628	-0.7515195810505540	4261
**CDH12**	0.22617	-2.9147	-0.7515195810505540	4260
**HINFP**	0.22617	-2.596	-0.7515195810505540	4257
**NRDE2**	0.22617	-2.7942	-0.7515195810505540	4259
**SLC44A4**	0.22617	-2.7279	-0.7515195810505540	4258
**TRIM49D1**	0.22617	-1.8092	-0.7515195810505540	4256
**LRRC40**	0.77383	-0.30969	-0.7515195810505530	4262
**BBIP1**	0.22623	-2.3191	-0.7513201239193240	4263
**CXorf51A**	0.2263	-1.8339	-0.7510874617035400	4264
**ZMYM5**	0.2263	-2.9376	-0.7510874617035400	4265
**CSNK2A2**	0.22632	-1.1184	-0.7510209942523440	4267
**ING2**	0.22632	-0.812	-0.7510209942523440	4266
**REXO2**	0.22635	-3.1402	-0.7509212992962080	4268
**DAZ2**	0.22638	-1.6863	-0.7508216118029850	4269
**RNFT1**	0.22654	-1.1567	-0.7502900711257950	4270
**METTL1**	0.22656	-0.90835	-0.750223643446848	4271
**ABHD16B**	0.22659	-0.74818	-0.7501240081350450	4272
**MNDA**	0.22663	-0.94841	-0.7499911726350600	4273
**GLYATL3**	0.22674	-1.0327	-0.749625943225112	4274
**SNRNP35**	0.22677	-3.8741	-0.7495263525582410	4275
**CDY2A**	0.77322	-0.12509	-0.7494931573212700	4276
**FBXL7**	0.22696	-1.1642	-0.7488957842033320	4277
**TNR**	0.77303	-0.32604	-0.7488626046445070	4278
**IST1**	0.227	-3.4277	-0.7487630709140600	4279
**PSCA**	0.22705	-2.5049	-0.7485971978454330	4281
**ZNF615**	0.22705	-1.6013	-0.7485971978454330	4280
**CAPZA2**	0.22712	-1.5667	-0.7483650101455440	4285
**CHCHD10**	0.22712	-1.2542	-0.7483650101455440	4284
**CTSC**	0.22712	-2.334	-0.7483650101455440	4286
**LDLRAD4**	0.22712	-0.93861	-0.7483650101455440	4283

**MGAT4D**	0.22712	-2.4885	-0.7483650101455440	4287
**PPP1R1A**	0.22712	-0.77137	-0.7483650101455440	4282
**EMID1**	0.77279	-0.15178	-0.7480665423715840	4288
**OR10H3**	0.22725	-0.93434	-0.747933911416806	4289
**MICU2**	0.2274	-1.9333	-0.7474366624629620	4290
**SLC44A5**	0.77253	-0.24894	-0.747204676189128	4291
**ATP5B**	0.22762	-0.742	-0.7467076980983050	4292
**SH2B2**	0.22769	-3.1326	-0.7464758380967580	4293
**SLC25A18**	0.22777	-0.63516	-0.7462109043664050	4294
**ERP44**	0.22778	-2.2292	-0.7461777913329100	4295
**TPM3**	0.77212	-0.12794	-0.7458467059832120	4296
**IFITM2**	0.22791	-1.534	-0.7457473963206430	4297
**YLPM1**	0.22794	-1.9608	-0.745648094012397	4298
**IQCJ-SCHIF**	0.22801	-1.4018	-0.7454164172150230	4299
**ZNF212**	0.22801	-2.1398	-0.7454164172150230	4300
**ANKRD20A2**	0.22803	-2.0445	-0.7453502311929260	4303
**C8B**	0.22803	-1.5232	-0.7453502311929260	4302
**DOCK5**	0.22803	-2.9602	-0.7453502311929260	4304
**PDIA3**	0.22803	-0.6054	-0.7453502311929260	4301
**TENM2**	0.77194	-0.19071	-0.7452509582813010	4305
**NAMPT**	0.22815	-2.1805	-0.7449531836021130	4306
**TTPA**	0.22829	-2.7591	-0.7444901097901910	4307
**ABCC4**	0.22853	-2.6051	-0.7436966401485280	4308
**FRS3**	0.2288	-2.7865	-0.7428045460971870	4309
**SLC6A6**	0.22894	-3.5345	-0.7423422114804140	4310
**TCEAL1**	0.22894	-3.7884	-0.7423422114804140	4311
**SUV39H2**	0.77102	-0.53514	-0.742210145019672	4312
**ZNF766**	0.22899	-3.4361	-0.7421771304271810	4313
**FOXR2**	0.771	-0.59218	-0.7421441166436160	4315
**IL36B**	0.229	-0.030105	-0.742144116643616	4314
**CAMK2G**	0.22902	-0.11335	-0.7420780915029630	4316
**EPHA7**	0.22902	-2.0867	-0.7420780915029630	4320

**FCMR**	0.22902	-1.9255	-0.7420780915029630	4319
**NOM1**	0.22902	-3.157	-0.7420780915029630	4323
**OR9A4**	0.22902	-2.9308	-0.7420780915029630	4322
**PIR**	0.22902	-0.99914	-0.7420780915029630	4318
**PTPN21**	0.22902	-0.23001	-0.7420780915029630	4317
**RNASE7**	0.22902	-2.0989	-0.7420780915029630	4321
**CFAP47**	0.22916	-0.95849	-0.7416160060588880	4324
**LYG1**	0.22923	-2.3673	-0.7413850227114950	4325
**DCANP1**	0.22938	-3.522	-0.7408901915363070	4326
**ZNF684**	0.22946	-0.89039	-0.7406263557428990	4327
**CDH17**	0.22954	-2.71	-0.7403625714939460	4328
**TMOD1**	0.22967	-3.0195	-0.7399340319248910	4329
**BCAS4**	0.77028	-0.075458	-0.7397692451939410	4330
**KRTAP9-6**	0.22973	-1.4725	-0.7397362902583410	4331
**FNDC1**	0.77023	-0.049364	-0.7396044785487400	4332
**CD53**	0.22982	-2.7188	-0.7394397319799200	4333
**ZDHHC24**	0.77014	-0.2996	-0.7393079491735660	4334
**FAM227A**	0.22987	-1.0645	-0.7392750054781180	4335
**BLOC1S4**	0.23011	-0.53998	-0.7384845973073180	4336
**KRAS**	0.23011	-1.4349	-0.7384845973073180	4340
**MT2A**	0.23011	-0.92246	-0.7384845973073180	4338
**PDXDC1**	0.23011	-0.55185	-0.7384845973073180	4337
**PLD1**	0.23011	-1.8694	-0.7384845973073180	4341
**RPS20**	0.23011	-2.2809	-0.7384845973073180	4342
**UAP1**	0.23011	-1.3198	-0.7384845973073180	4339
**EIF2B5**	0.23013	-0.87181	-0.738418750787329	4343
**ITFG2**	0.23015	-0.54369	-0.7383529074687960	4344
**GPR89B**	0.23017	-1.1964	-0.7382870673511210	4345
**GIGYF1**	0.23019	-3.0961	-0.7382212304337090	4346
**LIN7A**	0.23038	-2.5911	-0.7375959392174040	4347
**URAD**	0.23039	-3.9642	-0.73756303714287	4348
**UQCR11**	0.23043	-1.6662	-0.7374314368282640	4349

**KCNJ15**	0.23045	-0.15167	-0.7373656414600390	4350
**SNIP1**	0.23054	-2.2471	-0.737069601794516	4351
**PLEK**	0.23063	-1.4495	-0.7367736267113380	4352
**MAML1**	0.23077	-1.9542	-0.7363133492663710	4353
**TTC29**	0.76916	-0.39539	-0.7360832690337640	4354
**GCNT2**	0.23087	-1.9905	-0.7359846751476160	4355
**KCMF1**	0.23092	-0.95253	-0.7358203679004090	4356
**MTRR**	0.23104	-0.68349	-0.735426111528358	4358
**TAAR2**	0.23104	-0.077875	-0.735426111528358	4357
**ARMT1**	0.23107	-0.67322	-0.7353275652966020	4360
**CAPN15**	0.23107	-0.59626	-0.7353275652966020	4359
**GFI1B**	0.23107	-1.5451	-0.7353275652966020	4362
**SURF1**	0.23107	-1.4343	-0.7353275652966020	4361
**FOXC2**	0.23109	-3.6246	-0.7352618717758410	4363
**MAP3K13**	0.23121	-2.8869	-0.7348677772630700	4364
**NPFFR2**	0.23125	-2.5236	-0.7347364377872530	4365
**SPRR1A**	0.23133	-0.59868	-0.7344737968500000	4366
**NEIL2**	0.76864	-0.020321	-0.7343753195599030	4367
**NPBWR1**	0.23139	-0.19456	-0.7342768493911040	4368
**PLEKHG1**	0.76854	-0.037513	-0.7340471133466940	4369
**GDPD2**	0.23147	-1.3728	-0.7340142970744470	4370
**BCOR**	0.2315	-2.8053	-0.733915853000098	4371
**STRA6**	0.23161	-1.7296	-0.7335549522265560	4372
**RGS7BP**	0.23181	-1.6755	-0.7328990136540720	4373
**VWC2L**	0.23194	-0.89583	-0.7324728226351480	4374
**OR51A2**	0.23196	-1.067	-0.7324072665951910	4375
**EIF3K**	0.23199	-1.6241	-0.732308938436524	4376
**RPL23**	0.76793	-0.012854	-0.7320467646202710	4377
**EIF5B**	0.23216	-3.2184	-0.7317518792085420	4382
**NFATC2**	0.23216	-2.4471	-0.7317518792085420	4379
**STXBP5L**	0.23216	-3.113	-0.7317518792085420	4381
**SV2B**	0.23216	-2.123	-0.7317518792085420	4378

**ZNF610**	0.23216	-2.6324	-0.7317518792085420	4380
**DSEL**	0.76784	-0.15459	-0.7317518792085410	4383
**FCER2**	0.23227	-1.8222	-0.731391550094198	4384
**STK35**	0.23231	-0.89228	-0.7312605448684820	4385
**FAM220A**	0.23232	-0.96015	-0.7312277955230100	4386
**CPAMD8**	0.23236	-2.924	-0.731096805982779	4387
**LOC653602**	0.76762	-0.10137	-0.7310313159166370	4388
**MTHFSD**	0.23242	-1.7068	-0.730900345189397	4389
**CDH22**	0.23245	-1.1446	-0.7308021253710920	4390
**FAM96B**	0.23246	-1.1043	-0.730769386998341	4391
**BTBD17**	0.23247	-0.48555	-0.7307366494088110	4392
**LRRC3**	0.2325	-3.2761	-0.7306384413388240	4393
**TET2**	0.76749	-0.14725	-0.7306057068814540	4394
**CCDC126**	0.23258	-3.7047	-0.7303765875958390	4395
**TIMM21**	0.2326	-1.1569	-0.7303111319850520	4396
**AMBRA1**	0.23265	-1.4858	-0.7301475066454260	4397
**N4BP2L2**	0.23268	-2.8988	-0.730049340824359	4398
**IL1RL1**	0.23276	-1.9243	-0.7297875996877890	4399
**KIF1B**	0.23295	-1.2487	-0.7291661647593330	4400
**SOX14**	0.23295	-1.7628	-0.7291661647593330	4401
**HBD**	0.76701	-0.39843	-0.7290353722350350	4402
**PRG2**	0.23305	-0.78385	-0.728839206828768	4403
**DENND6A**	0.23333	-2.8475	-0.7279241387973790	4404
**NT5DC3**	0.76665	-0.30268	-0.7278588001220420	4405
**CCL21**	0.23339	-0.03347	-0.727728132092354	4406
**DES**	0.23339	-2.7395	-0.727728132092354	4413
**EEF2KMT**	0.23339	-0.037184	-0.727728132092354	4407
**FAM13B**	0.23339	-2.4837	-0.727728132092354	4411
**FAM193A**	0.23339	-0.49689	-0.727728132092354	4408
**FBLN2**	0.23339	-2.7659	-0.727728132092354	4414
**GABRG1**	0.23339	-2.2282	-0.727728132092354	4410
**MAP2K3**	0.23339	-2.5377	-0.727728132092354	4412

**PDZD9**	0.23339	-0.94546	-0.727728132092354	4409
**MS4A10**	0.23358	-0.38105	-0.727107628540344	4415
**TAS2R3**	0.7664	-0.071575	-0.7270423286636000	4416
**ZFP91**	0.23365	-1.3345	-0.7268790925330630	4417
**C5orf52**	0.23373	-0.99892	-0.7266179550021020	4418
**MCL1**	0.23373	-2.1561	-0.7266179550021020	4419
**RGPD1**	0.23375	-0.86971	-0.7265526783615350	4420
**PHF19**	0.23378	-1.2281	-0.7264547692049910	4421
**ACOXL**	0.76619	-0.3513	-0.7263568670119000	4422
**KLHL26**	0.23385	-0.71201	-0.7262263415831490	4423
**KTN1**	0.23385	-1.4904	-0.7262263415831490	4424
**LRRC57**	0.23432	-1.037	-0.7246935932714040	4425
**ROBO3**	0.23449	-2.5917	-0.724139614142641	4426
**DNPH1**	0.23459	-0.84426	-0.7238138478590740	4427
**JKAMP**	0.23459	-2.7332	-0.7238138478590740	4429
**MSH3**	0.23459	-2.0405	-0.7238138478590740	4428
**CHCHD2**	0.23463	-1.5723	-0.7236835628524180	4430
**TMEM135**	0.23463	-1.7144	-0.7236835628524180	4431
**SLC25A4**	0.23468	-0.18898	-0.7235207238662400	4432
**GALM**	0.23475	-0.87005	-0.7232927815110830	4433
**CNTNAP2**	0.23498	-3.0532	-0.7225440924835450	4434
**LOC1019273**	0.76494	-0.39736	-0.7222837738377040	4435
**TTC1**	0.23509	-1.1586	-0.722186166964014	4436
**LSM 5.00**	0.23513	-1.115	-0.7220560351674400	4437
**ATF4**	0.23515	-0.6921	-0.721990973854321	4438
**ANXA6**	0.23527	-2.7554	-0.721600670132524	4439
**CDC14B**	0.2354	-0.83439	-0.7211779651109400	4440
**CDK7**	0.23551	-2.9594	-0.7208203922545740	4441
**ADGRG3**	0.23559	-2.222	-0.7205603971460280	4446
**ANKRD55**	0.23559	-0.99428	-0.7205603971460280	4443
**C6orf118**	0.23559	-2.9079	-0.7205603971460280	4448
**EAPP**	0.23559	-1.0395	-0.7205603971460280	4444

**FAM163A**	0.23559	-2.7945	-0.7205603971460280	4447
**NUMBL**	0.23559	-3.0147	-0.7205603971460280	4449
**OR5M10**	0.23559	-0.61263	-0.7205603971460280	4442
**TPD52L1**	0.23559	-2.1161	-0.7205603971460280	4445
**GARNL3**	0.23564	-0.77206	-0.7203979249354060	4450
**SRRM5**	0.23566	-1.3461	-0.7203329413756490	4451
**RB1CC1**	0.23579	-1.1464	-0.7199106223543650	4452
**KRTAP10-11**	0.23589	-1.4148	-0.7195858489220580	4453
**POM121L2**	0.76403	-0.12456	-0.7193260848150710	4454
**SLC22A25**	0.23612	-3.0233	-0.7188391578878810	4455
**KCTD6**	0.23616	-0.39236	-0.7187093394915530	4456
**SELENOV**	0.23617	-3.0322	-0.7186768867850130	4458
**SMUG1**	0.23617	-1.8843	-0.7186768867850130	4457
**LOC729159**	0.76381	-0.45747	-0.7186119836424980	4459
**RASAL2**	0.23623	-1.1137	-0.7184821864377780	4460
**DUSP14**	0.76375	-0.31423	-0.7184172923744630	4461
**RELB**	0.2363	-0.99071	-0.7182550704505990	4462
**PEX26**	0.23648	-0.017913	-0.7176712279493780	4463
**CD300A**	0.23655	-2.7468	-0.7174442441487020	4469
**DDX27**	0.23655	-1.0061	-0.7174442441487020	4464
**FEZF1**	0.23655	-1.9108	-0.7174442441487020	4465
**NUDC**	0.23655	-2.7328	-0.7174442441487020	4468
**OR1K1**	0.23655	-2.6573	-0.7174442441487020	4467
**POC1A**	0.23655	-2.6332	-0.7174442441487020	4466
**LCE2C**	0.23659	-1.2183	-0.7173145557146200	4470
**NLRP4**	0.23659	-3.3182	-0.7173145557146200	4471
**KRTAP22-2**	0.23673	-1.6617	-0.7168607411661120	4472
**VWA5B2**	0.23707	-2.4217	-0.715759234192244	4473
**ACTL10**	0.76287	-0.42131	-0.7155649407645470	4474
**ERICH2**	0.76238	-0.073793	-0.7139792207342450	4475
**SIVA1**	0.23766	-2.235	-0.7138498534799750	4476
**GJA5**	0.23768	-0.53412	-0.7137851743327960	4477

**EXOSC7**	0.23774	-2.0254	-0.7135911548045230	4478
**ABCA1**	0.23778	-2.0739	-0.713461823374604	4487
**AEN**	0.23778	-0.53146	-0.713461823374604	4480
**ARSJ**	0.23778	-2.809	-0.713461823374604	4490
**DSC3**	0.23778	-0.76209	-0.713461823374604	4482
**FANCD2**	0.23778	-2.8005	-0.713461823374604	4489
**FNBP4**	0.23778	-0.71576	-0.713461823374604	4481
**GDF7**	0.23778	-1.1811	-0.713461823374604	4484
**GDPD5**	0.23778	-2.1661	-0.713461823374604	4488
**GLUD2**	0.23778	-0.9112	-0.713461823374604	4483
**GTF2H2**	0.23778	-1.7358	-0.713461823374604	4486
**PABPC4**	0.23778	-2.9635	-0.713461823374604	4491
**PET100**	0.23778	-1.5694	-0.713461823374604	4485
**TAB1**	0.23778	-0.37354	-0.713461823374604	4479
**MISP**	0.23779	-2.874	-0.7134294923817810	4492
**BAIAP2L2**	0.23783	-3.2469	-0.7133001758670750	4496
**CLPSL2**	0.23783	-2.0119	-0.7133001758670750	4494
**HHLA2**	0.23783	-3.6945	-0.7133001758670750	4498
**KLF10**	0.23783	-1.0319	-0.7133001758670750	4493
**OR2J3**	0.23783	-2.8156	-0.7133001758670750	4495
**PDLIM7**	0.23783	-3.5511	-0.7133001758670750	4497
**SLC25A44**	0.76216	-0.29438	-0.7132678486022030	4499
**NPIPA5**	0.2379	-1.0211	-0.7130739006636860	4500
**PSMC2**	0.23805	-2.7066	-0.7125891481190840	4501
**C14orf79**	0.23815	-2.9382	-0.7122660727647070	4502
**PML**	0.23833	-1.3954	-0.7116847243869270	4503
**C5orf47**	0.23851	-2.8597	-0.7111036164351600	4504
**ANO10**	0.23858	-0.26934	-0.7108776948543990	4505
**ZNF106**	0.23866	-1.2725	-0.7106195431825590	4506
**MRPL58**	0.76127	-0.29283	-0.7103936993115930	4507
**MTUS2**	0.23885	-2.3488	-0.7100066226631020	4508
**PTBP3**	0.76102	-0.22151	-0.7095874096366170	4509

**TRIM63**	0.23903	-0.73096	-0.7094262070602660	4510
**PPP1R42**	0.23909	-0.58347	-0.7092327882998030	4511
**BTNL9**	0.23925	-2.7221	-0.7087171345983110	4514
**MTRNR2L2**	0.23925	-2.72	-0.7087171345983110	4513
**TGIF2LY**	0.23925	-2.2342	-0.7087171345983110	4512
**SPARC**	0.23931	-0.88044	-0.708523813041306	4515
**H2BFWT**	0.23936	-1.2023	-0.7083627319693100	4516
**DEFB132**	0.23957	-0.35529	-0.7076863920560450	4517
**PPP4C**	0.23988	-1.1705	-0.7066885767091480	4518
**TRAPPC6A**	0.23994	-2.8562	-0.7064955324321550	4519
**NUMA1**	0.76	-0.33209	-0.7063025144799330	4520
**MOB3A**	0.24017	-2.0187	-0.7057557731144340	4521
**CDKN2AIP**	0.24023	-3.8993	-0.7055628559408190	4522
**FOXP3**	0.24031	-0.109	-0.7053056738819500	4523
**EPDR1**	0.24032	-2.5008	-0.7052735294048280	4524
**GHRHR**	0.2405	-1.5873	-0.7046950533636820	4525
**ABT1**	0.24052	-0.013074	-0.7046307928056400	4526
**APOC1**	0.24052	-0.61368	-0.7046307928056400	4528
**ESR1**	0.24052	-0.77858	-0.7046307928056400	4529
**KCNK4**	0.24052	-2.4321	-0.7046307928056400	4531
**N4BP3**	0.24052	-3.0149	-0.7046307928056400	4532
**SYT4**	0.24052	-2.0816	-0.7046307928056400	4530
**TNXB**	0.24052	-0.56995	-0.7046307928056400	4527
**FBXW4**	0.24055	-0.86367	-0.7045344074238890	4533
**LGALS14**	0.24055	-2.3319	-0.7045344074238890	4534
**CACNA1B**	0.7593	-0.32169	-0.704052578651294	4535
**ZNF626**	0.2407	-2.8654	-0.7040525786512940	4536
**PET117**	0.24072	-0.023011	-0.7039883471653520	4537
**PAGE5**	0.24079	-1.5009	-0.7037635598325010	4538
**USP22**	0.24086	-0.33598	-0.7035388080547850	4539
**SYP**	0.24125	-2.2089	-0.7022872694125960	4540
**NR4A3**	0.24128	-0.78631	-0.7021910427822250	4541

**FDXR**	0.24134	-1.3026	-0.7019986090243730	4542
**GBP2**	0.24141	-0.977	-0.701774135823627	4543
**BRINP2**	0.24144	-0.75429	-0.7016779438477670	4544
**FAM189B**	0.24144	-1.2727	-0.7016779438477670	4545
**PDE6G**	0.24144	-2.7326	-0.7016779438477670	4546
**PHF21B**	0.24144	-2.969	-0.7016779438477670	4547
**THRAP3**	0.24164	-2.776	-0.7010368298333220	4548
**MKNK2**	0.2417	-0.59162	-0.7008445518158970	4549
**NCSTN**	0.24172	-0.35457	-0.7007804649013070	4550
**USP17L13**	0.24175	-1.6497	-0.7006843399256480	4551
**CCDC85A**	0.24179	-2.6909	-0.7005561833616470	4555
**DENND2D**	0.24179	-0.88805	-0.7005561833616470	4552
**MFAP5**	0.24179	-2.8747	-0.7005561833616470	4556
**NUTM2G**	0.24179	-1.2509	-0.7005561833616470	4553
**TMEM203**	0.24179	-2.5099	-0.7005561833616470	4554
**KIF5A**	0.24195	-3.4968	-0.7000436721138730	4557
**RRP9**	0.75801	-0.35003	-0.6999155730331550	4558
**ALDH8A1**	0.24204	-3.0003	-0.6997554653313740	4559
**EPGN**	0.24211	-0.66031	-0.6995313446793410	4560
**EEPD1**	0.24237	-0.62604	-0.6986992039516100	4562
**LOC1009966**	0.24237	-0.042475	-0.6986992039516100	4561
**MAST2**	0.24237	-1.7049	-0.6986992039516100	4564
**NFIX**	0.24237	-2.1367	-0.6986992039516100	4565
**NOP9**	0.24237	-2.4515	-0.6986992039516100	4567
**NYX**	0.24237	-2.4464	-0.6986992039516100	4566
**OR56A5**	0.24237	-2.9363	-0.6986992039516100	4568
**PAPSS2**	0.24237	-1.2062	-0.6986992039516100	4563
**ANKRD36B**	0.24239	-0.8915	-0.6986352131691700	4569
**MRPL49**	0.24246	-1.0324	-0.6984112679549430	4570
**NAPB**	0.24251	-1.2382	-0.6982513285303090	4571
**KLHL15**	0.24254	-2.6892	-0.698155373448718	4572
**VSTM4**	0.24257	-3.6609	-0.6980594247948860	4573

**NLGN2**	0.24269	-1.3127	-0.6976756944222690	4574
**NLGN4Y**	0.75723	-0.10171	-0.6974199312389080	4575
**SIRT7**	0.2429	-0.1821	-0.6970044133363780	4576
**FOXD4L3**	0.24293	-1.999	-0.6969085416797670	4577
**RTKN2**	0.2431	-0.99623	-0.6963653898937290	4578
**NEXN**	0.24321	-1.4589	-0.696014048188467	4579
**IQCC**	0.24325	-3.378	-0.6958863088693390	4581
**KCNF1**	0.24325	-0.67945	-0.6958863088693390	4580
**ANKRD27**	0.24326	-2.2364	-0.6958543758137810	4582
**PSMC6**	0.24329	-2.6332	-0.6957585809042270	4583
**PIK3R3**	0.24333	-0.82019	-0.6956308642890300	4584
**TGFBR3**	0.24342	-0.30833	-0.6953435433817340	4585
**CACUL1**	0.24353	-3.1662	-0.6949924513183520	4591
**FIBP**	0.24353	-0.55339	-0.6949924513183520	4586
**KPNA6**	0.24353	-2.0908	-0.6949924513183520	4589
**MCM2**	0.24353	-2.8301	-0.6949924513183520	4590
**TAP1**	0.24353	-1.5341	-0.6949924513183520	4587
**WAC**	0.24353	-2.0269	-0.6949924513183520	4588
**PPP1R32**	0.24362	-2.5497	-0.6947052578844840	4592
**NUP54**	0.24391	-1.1234	-0.6937802462853260	4593
**KRTAP3-2**	0.24397	-2.4676	-0.6935889386945640	4594
**C1orf195**	0.24405	-1.3971	-0.6933339013861460	4595
**CNPY1**	0.2442	-1.4337	-0.6928558279371340	4596
**HEATR5B**	0.24441	-1.8076	-0.6921867910095550	4597
**ELAVL3**	0.24444	-1.1121	-0.6920912395998640	4598
**NR1D2**	0.24447	-3.1867	-0.6919956945086090	4599
**CNPY2**	0.2446	-2.2882	-0.6915817387512180	4600
**AOC2**	0.24468	-0.43747	-0.6913270556426350	4601
**OR6V1**	0.24468	-0.81426	-0.6913270556426350	4602
**PEX13**	0.24468	-1.8901	-0.6913270556426350	4604
**WNK1**	0.24468	-1.4218	-0.6913270556426350	4603
**KDM6B**	0.24472	-2.0773	-0.6911997309031360	4605

**NXF1**	0.24475	-0.9924	-0.6911042447016710	4606
**BMPER**	0.75524	-0.29999	-0.6910724173681150	4607
**HSPB8**	0.24479	-0.55577	-0.6909769395673610	4608
**PTPDC1**	0.75517	-0.11611	-0.6908496456304690	4609
**NCMAP**	0.24486	-0.40073	-0.6907541825236810	4610
**LOC286068**	0.24513	-0.03882	-0.6898952976148230	4611
**MAP7D3**	0.24513	-0.65985	-0.6898952976148230	4614
**MLN**	0.24513	-0.23984	-0.6898952976148230	4613
**TDP1**	0.24513	-0.089562	-0.6898952976148230	4612
**C16orf52**	0.24514	-3.0807	-0.6898634968380680	4615
**DKKL1**	0.24526	-1.0627	-0.6894819419148030	4616
**HS2ST1**	0.24532	-1.7707	-0.6892912020917230	4617
**PPP6R1**	0.75465	-0.16698	-0.6891958415838980	4618
**COPZ1**	0.24539	-3.0592	-0.689068703988331	4619
**VNN 2.00**	0.24562	-0.67488	-0.6883378788206990	4622
**HTR3D**	0.24562	-2.178	-0.6883378788206990	4623
**LHX5**	0.24562	-2.2907	-0.6883378788206990	4620
**WDR81**	0.24562	-0.71623	-0.6883378788206990	4621
**SFXN5**	0.75433	-0.17638	-0.6881790524347820	4624
**G6PC3**	0.75429	-0.33092	-0.6880520038241940	4625
**IP6K1**	0.24576	-2.0333	-0.6878932086770890	4626
**CHEK1**	0.24583	-2.4413	-0.687670924607284	4627
**NT5DC1**	0.24586	-2.8312	-0.68757567040703	4628
**ONECUT1**	0.24587	-3.9133	-0.6875439203933010	4629
**EEF1E1**	0.24608	-1.867	-0.686877330109236	4630
**TMEM210**	0.75361	-0.28814	-0.6858938732787610	4631
**GALK1**	0.24645	-2.7454	-0.6857036034256090	4632
**TMED6**	0.24647	-2.8165	-0.6856401856576790	4633
**HAO2**	0.24654	-1.7011	-0.6854182451812150	4634
**SOCS5**	0.24665	-0.24035	-0.6850695497700380	4635
**IGSF6**	0.24668	-3.4943	-0.6849744654779960	4636
**XKR8**	0.24672	-1.4499	-0.6848476960543940	4637

**CPT2**	0.24678	-0.9703	-0.6846575625519460	4639
**HK3**	0.24678	-1.9545	-0.6846575625519460	4641
**ISG15**	0.24678	-2.0739	-0.6846575625519460	4642
**KCNA3**	0.24678	-0.35384	-0.6846575625519460	4638
**TSTD3**	0.24678	-1.0591	-0.6846575625519460	4640
**CNOT6L**	0.24703	-1.5184	-0.6838656058554750	4644
**NAGS**	0.24703	-0.88262	-0.6838656058554750	4643
**ADAM18**	0.24713	-1.0398	-0.6835489432627580	4645
**MIS12**	0.24729	-3.3604	-0.6830424256277430	4647
**S1PR3**	0.24729	-1.0439	-0.6830424256277430	4646
**SMC6**	0.24737	-3.3272	-0.6827892325188640	4648
**IL3**	0.24739	-0.33194	-0.6827259410809810	4649
**PPIAL4C**	0.24742	-1.0683	-0.682631009051668	4650
**ARL14EP**	0.24746	-0.66737	-0.6825044425813690	4651
**B3GAT1**	0.2475	-0.69056	-0.6823778870432340	4652
**ZNF613**	0.75245	-0.049219	-0.6822197079879560	4653
**KRTAP4-4**	0.24765	-1.6665	-0.6819034010730000	4654
**MMP12**	0.24773	-3.5655	-0.6816504046550920	4657
**RCL1**	0.24773	-0.55206	-0.6816504046550920	4655
**SEMA6C**	0.24773	-1.0047	-0.6816504046550920	4656
**OR5M9**	0.75208	-0.095047	-0.6810497129383640	4658
**CYP3A43**	0.75201	-0.088744	-0.6808284674071600	4659
**GDI2**	0.248	-1.3782	-0.6807968636232960	4660
**CD248**	0.24805	-2.2333	-0.6806388549022520	4665
**DLGAP3**	0.24805	-2.927	-0.6806388549022520	4667
**GDPGP1**	0.24805	-0.083068	-0.6806388549022520	4661
**KLK1**	0.24805	-1.9621	-0.6806388549022520	4664
**LYRM1**	0.24805	-2.6006	-0.6806388549022520	4666
**OR5AR1**	0.24805	-0.48593	-0.6806388549022520	4662
**XXYLT1**	0.24805	-0.54814	-0.6806388549022520	4663
**EHMT2**	0.24807	-1.1236	-0.6805756561718930	4668
**MAPRE3**	0.75189	-0.11048	-0.680449266865175	4669

**TLX2**	0.2483	-1.2736	-0.6798490660184000	4670
**POLR2J**	0.24845	-1.9204	-0.6793753961920750	4671
**VEGFC**	0.24865	-1.6792	-0.6787440734208720	4672
**SLITRK3**	0.24874	-0.047403	-0.6784600664242	4673
**NTN1**	0.24879	-0.53175	-0.6783023084041100	4674
**FAM187A**	0.24882	-0.4185	-0.6782076616947170	4675
**GAB4**	0.75116	-0.25869	-0.6781445672635590	4676
**UVRAG**	0.2489	-2.3279	-0.6779553001652790	4677
**DEFA3**	0.24897	-1.5329	-0.6777345192403040	4678
**MANEAL**	0.24907	-1.4956	-0.6774191752120590	4679
**MRPS12**	0.24917	-1.0033	-0.6771038985333860	4680
**DLG4**	0.24923	-1.7072	-0.6769147648289770	4682
**DNAJC25-G**	0.24923	-0.86664	-0.6769147648289770	4681
**FANK1**	0.24923	-2.7101	-0.6769147648289770	4684
**MAGEB5**	0.24923	-2.8552	-0.6769147648289770	4685
**MYH7B**	0.24923	-2.6373	-0.6769147648289770	4683
**PBXIP1**	0.24923	-3.1202	-0.6769147648289770	4686
**COG3**	0.24935	-3.0364	-0.6765365700409370	4687
**RNASE4**	0.24937	-2.1231	-0.6764735469844700	4688
**ZMAT2**	0.75054	-0.41094	-0.6761899764723510	4689
**TOMM40**	0.75042	-0.48196	-0.6758119670143090	4690
**VAX2**	0.24964	-2.424	-0.6756229984954030	4691
**LAPTM5**	0.24969	-0.89252	-0.6754655431573810	4692
**PARD3**	0.75028	-0.47758	-0.6753710779924140	4693
**ANXA8L1**	0.24987	-2.1802	-0.6748988425315820	4694
**MINOS1-NB**	0.24989	-2.348	-0.6748358891758820	4695
**ACSM2B**	0.24993	-0.91661	-0.6747099904870570	4697
**KNDC1**	0.24993	-0.5252	-0.6747099904870570	4696
**MYO7A**	0.24993	-2.5805	-0.6747099904870570	4698
**NYAP2**	0.25009	-1.4288	-0.6742065026295650	4699
**PLEKHA6**	0.25019	-0.025432	-0.6738919094957520	4700
**TMEM106C**	0.25023	-1.6758	-0.673766090916039	4701

**ARHGEF38**	0.25024	-3.06	-0.673734637937673	4711
**FBXO7**	0.25024	-2.1708	-0.673734637937673	4706
**GLRA1**	0.25024	-2.2091	-0.673734637937673	4707
**IL17RE**	0.25024	-2.4908	-0.673734637937673	4709
**JOSD1**	0.25024	-0.071539	-0.673734637937673	4702
**NOL11**	0.25024	-2.3354	-0.673734637937673	4708
**SPATA18**	0.25024	-2.9856	-0.673734637937673	4710
**TPPP**	0.25024	-1.9629	-0.673734637937673	4705
**ZAN**	0.25024	-1.0783	-0.673734637937673	4704
**ZNF334**	0.25024	-0.61009	-0.673734637937673	4703
**MYPN**	0.25036	-1.5103	-0.6733572541677850	4712
**CATIP**	0.25054	-2.715	-0.6727913582454230	4713
**ATP5D**	0.25056	-1.8862	-0.6727284942215930	4714
**MPP6**	0.25073	-0.98275	-0.6721942573057810	4715
**CRNDE**	0.25077	-1.0421	-0.6720685823842260	4716
**SEC14L1**	0.25079	-1.73	-0.6720057489038970	4717
**CXCR5**	0.25083	-0.85131	-0.671880089901779	4718
**NUP62CL**	0.2509	-3.7024	-0.6716602121718760	4719
**RSPH3**	0.25096	-0.11809	-0.6714717713884950	4720
**MRGPRX3**	0.2512	-2.1029	-0.6707182465373300	4721
**ISOC1**	0.25126	-0.25142	-0.6705299248315950	4722
**MYCBP2**	0.25133	-1.5005	-0.670310246226053	4723
**SLC36A1**	0.2514	-3.1737	-0.670090599964115	4724
**ATAD2B**	0.74858	-0.024277	-0.6700278498271220	4725
**AFF2**	0.25143	-1.0596	-0.6699964757479740	4726
**MINK1**	0.25145	-2.7793	-0.6699337295680800	4727
**PHF20L1**	0.25145	-3.6378	-0.6699337295680800	4728
**GPBP1**	0.25157	-0.006905	-0.6695573078593890	4729
**ADCYAP1**	0.25163	-0.80669	-0.6693691325794000	4730
**GNA12**	0.25164	-2.5062	-0.669337772337183	4732
**GPR25**	0.25164	-1.5693	-0.669337772337183	4731
**HBZ**	0.25164	-2.6574	-0.669337772337183	4733

**IGK**	0.25164	-2.871	-0.669337772337183	4734
**C17orf49**	0.25165	-2.641	-0.6693064127532240	4735
**TRIM77**	0.25184	-1.0503	-0.6687107056492570	4736
**TMEM236**	0.25198	-0.16597	-0.6682719153828380	4737
**MAPK3**	0.74797	-0.043718	-0.66811523575469	4738
**WLS**	0.25223	-0.60271	-0.6674886811648660	4739
**MSH4**	0.25226	-0.91054	-0.6673947205790040	4740
**HOXC13**	0.74773	-0.025626	-0.6673634016930830	4741
**ZMAT5**	0.25237	-0.70806	-0.6670502488265360	4742
**CCDC154**	0.2527	-3.8259	-0.6660173080687790	4743
**BARHL1**	0.25273	-0.18593	-0.6659234396078820	4744
**GINM1**	0.74724	-0.068536	-0.6658295770142750	4745
**AREL1**	0.25286	-2.014	-0.6655167440570970	4746
**HERC1**	0.25289	-1.7717	-0.6654229068703920	4747
**KRT9**	0.25291	-3.203	-0.6653603520010040	4748
**EXOC6**	0.25295	-2.8323	-0.665235250072347	4749
**KRT75**	0.74704	-0.20029	-0.6652039762169550	4750
**ADCY1**	0.253	-0.79246	-0.6650788873007440	4752
**ADCY2**	0.253	-1.2503	-0.6650788873007440	4754
**ARSF**	0.253	-3.1795	-0.6650788873007440	4760
**BSND**	0.253	-2.04	-0.6650788873007440	4756
**COL4A4**	0.253	-2.858	-0.6650788873007440	4759
**DLGAP5**	0.253	-2.8151	-0.6650788873007440	4758
**FCRL1**	0.253	-2.5273	-0.6650788873007440	4757
**TUSC5**	0.253	-1.9088	-0.6650788873007440	4755
**WASHC5**	0.253	-0.68281	-0.6650788873007440	4751
**ZBTB7B**	0.253	-1.1731	-0.6650788873007440	4753
**NFIB**	0.25308	-1.3582	-0.6648287406820530	4761
**MSMO1**	0.25323	-1.8311	-0.6643598278569460	4762
**SNRNP27**	0.25343	-1.538	-0.6637348378869710	4763
**DGKA**	0.25348	-2.2188	-0.6635786309055670	4764
**CENPI**	0.25356	-1.4998	-0.6633287334077310	4765

**LEMD3**	0.25358	-1.2648	-0.663266265505869	4766
**TMX3**	0.25364	-1.0116	-0.6630788773271870	4767
**PARVB**	0.25399	-1.3052	-0.6619862432195430	4768
**PIGS**	0.25422	-3.0654	-0.6612686566791890	4772
**SACM1L**	0.25422	-0.081534	-0.6612686566791890	4769
**ST3GAL4**	0.25422	-2.7645	-0.6612686566791890	4770
**TRIM58**	0.25422	-2.7833	-0.6612686566791890	4771
**SRP14**	0.25428	-2.0367	-0.661081516192296	4773
**ACTL7A**	0.25432	-1.4384	-0.6609567687291820	4774
**PLIN4**	0.25432	-2.7281	-0.6609567687291820	4775
**PLCB1**	0.25435	-2.0893	-0.6608632148815370	4776
**RNF128**	0.25435	-3.5359	-0.6608632148815370	4778
**SIRPB1**	0.25435	-2.1067	-0.6608632148815370	4777
**RGS6**	0.2544	-0.75424	-0.6607073046541380	4779
**NOSIP**	0.25442	-2.5168	-0.660644945060056	4780
**ZNF644**	0.25444	-1.2119	-0.6605825880349370	4781
**ANKRD53**	0.25447	-0.18434	-0.6604890573130720	4782
**LACTB2**	0.25448	-0.9364	-0.6604578816897740	4783
**DHRS7**	0.74547	-0.60636	-0.6603020132006500	4784
**WDR83**	0.74542	-0.036662	-0.6601461607519440	4785
**SPIB**	0.25471	-0.46507	-0.6597410194036270	4786
**TMEM141**	0.25484	-1.6289	-0.659335986316222	4787
**OLIG3**	0.2551	-0.79332	-0.6585262444273100	4788
**DNAJB6**	0.2552	-1.9489	-0.6582149202044100	4789
**PRR22**	0.25534	-1.1204	-0.6577791734353690	4790
**SECTM1**	0.2554	-2.9808	-0.6575924630516700	4793
**SPOPL**	0.2554	-2.063	-0.6575924630516700	4791
**TBPL1**	0.2554	-2.7789	-0.6575924630516700	4792
**SPRR2B**	0.25544	-1.87	-0.6574680021974100	4794
**CFAP46**	0.25558	-3.3474	-0.6570324693805940	4797
**GBA**	0.25558	-2.162	-0.6570324693805940	4796
**NMUR1**	0.25558	-1.8356	-0.6570324693805940	4795

**MAP2K2**	0.74436	-0.19032	-0.6568458506098280	4798
**ENTHD1**	0.2557	-2.7632	-0.6566592547120040	4799
**OR4F21**	0.25574	-1.1494	-0.6565348701480010	4800
**NTF3**	0.256	-0.70249	-0.6557266178937310	4801
**DNAH7**	0.25605	-0.94968	-0.6555712338825160	4802
**CHRNB1**	0.25612	-3.0715	-0.6553537228540540	4803
**UXT**	0.25613	-0.65787	-0.6553226523811390	4804
**GSTZ1**	0.25614	-1.1171	-0.6552915825408440	4805
**IGFBP1**	0.2562	-0.9514	-0.6551051767809850	4806
**RASL11B**	0.25622	-2.0288	-0.6550430465861350	4807
**NCCRP1**	0.25623	-1.6932	-0.6550119824369160	4808
**DCST2**	0.74374	-0.49126	-0.6549187937814170	4809
**MRPL33**	0.2563	-2.8702	-0.6547945510870370	4810
**CACNA1A**	0.25634	-3.0908	-0.6546703184994330	4811
**CD200**	0.25654	-2.7942	-0.6540493070384550	4815
**DENND1C**	0.25654	-1.3193	-0.6540493070384550	4813
**EFNA2**	0.25654	-0.22906	-0.6540493070384550	4812
**ZBTB32**	0.25654	-2.4563	-0.6540493070384550	4814
**STAR**	0.25656	-1.308	-0.653987219767156	4816
**HEMK1**	0.7434	-0.45738	-0.6538630527868550	4817
**GSTM4**	0.25662	-2.4894	-0.6538009730769660	4818
**PELI1**	0.25667	-0.21491	-0.6536457848244410	4819
**TMEM208**	0.25675	-2.429	-0.6533975163576670	4820
**USP28**	0.74322	-0.0125	-0.6533044260655350	4821
**TAF4B**	0.25687	-0.88222	-0.653025189149631	4822
**LMNB2**	0.25693	-3.0698	-0.6528390594911600	4823
**VPS37A**	0.25696	-1.8312	-0.6527460031430850	4824
**RBPJL**	0.25697	-0.36654	-0.652714985616492	4825
**SNAP23**	0.25704	-0.91917	-0.6524978805100630	4826
**ATP5O**	0.25707	-2.0503	-0.6524048448791180	4827
**SLC10A2**	0.25713	-1.3695	-0.6522187905556780	4828
**EPB41L4B**	0.74284	-0.042016	-0.6521257718602040	4829

**ZNF440**	0.25725	-2.3424	-0.6518467496208880	4830
**ADH1B**	0.25731	-3.3459	-0.6516607629857150	4832
**ATP6V1G2**	0.25731	-2.6753	-0.6516607629857150	4831
**DMRTB1**	0.25731	-3.4318	-0.6516607629857150	4833
**PPM1B**	0.25731	-3.4998	-0.6516607629857150	4834
**ULK4**	0.25742	-0.11939	-0.6513198460179740	4835
**CC2D2B**	0.25746	-1.1181	-0.6511958949796670	4836
**KCNJ16**	0.25749	-2.5618	-0.6511029382663230	4837
**CACNA1I**	0.25772	-0.97443	-0.6503904569235390	4838
**YTHDC2**	0.25775	-1.401	-0.6502975489180680	4839
**DSPP**	0.25781	-1.8146	-0.6501117497445820	4845
**HSPB11**	0.25781	-0.1662	-0.6501117497445820	4841
**KIAA0907**	0.25781	-2.4916	-0.6501117497445820	4847
**KRT84**	0.25781	-2.4871	-0.6501117497445820	4846
**PCDHGA5**	0.25781	-0.72315	-0.6501117497445820	4842
**PTPRJ**	0.25781	-0.88449	-0.6501117497445820	4843
**RTL5**	0.25781	-0.07711	-0.6501117497445820	4840
**ZBTB8A**	0.25781	-1.585	-0.6501117497445820	4844
**SRF**	0.74201	-0.28486	-0.6495544868172790	4848
**FAM9A**	0.74187	-0.46937	-0.6491211995188640	4849
**TAS2R8**	0.25818	-2.4016	-0.6489664835882290	4850
**SFTPB**	0.25828	-1.0178	-0.6486570983185630	4851
**LRRIQ1**	0.25836	-1.3139	-0.648409634800665	4852
**MBNL2**	0.25855	-1.5141	-0.6478220680093330	4853
**PSEN2**	0.25856	-2.4764	-0.6477911496376020	4854
**PPFIBP2**	0.74134	-0.46065	-0.6474819999696570	4855
**EEF1A1**	0.25868	-0.21357	-0.6474201774621980	4856
**DGCR2**	0.7413	-0.6311	-0.647358357429101	4857
**RPAP3**	0.74126	-0.3274	-0.6472347247842580	4858
**DCTN6**	0.25875	-1.2354	-0.6472038181688450	4859
**GPRASP1**	0.25875	-2.5076	-0.6472038181688450	4862
**GRK5**	0.25875	-1.8418	-0.6472038181688450	4861

**RPL10**	0.25875	-1.3612	-0.6472038181688450	4860
**SLC16A10**	0.25875	-3.3807	-0.6472038181688450	4863
**ZNF556**	0.25892	-0.6027	-0.6466785002488260	4864
**DPP4**	0.25895	-2.8799	-0.6465858156127330	4865
**VRK3**	0.74097	-0.53018	-0.6463386837327620	4866
**DUSP10**	0.25908	-3.357	-0.64618424635209	4870
**LGALS9**	0.25908	-2.2389	-0.64618424635209	4867
**PIGBOS1**	0.25908	-2.4087	-0.64618424635209	4868
**SKIV2L2**	0.25908	-2.9062	-0.64618424635209	4869
**SLC4A2**	0.25908	-3.6797	-0.64618424635209	4871
**GALNT18**	0.25912	-0.74967	-0.6460607075434990	4873
**ITGB1BP2**	0.25912	-2.4277	-0.6460607075434990	4878
**LSMEM2**	0.25912	-0.54339	-0.6460607075434990	4872
**MMP24**	0.25912	-2.2202	-0.6460607075434990	4877
**MS4A2**	0.25912	-2.7672	-0.6460607075434990	4879
**NRN1**	0.25912	-2.0133	-0.6460607075434990	4876
**PLEKHH2**	0.25912	-1.0673	-0.6460607075434990	4874
**ZNF211**	0.25912	-1.7793	-0.6460607075434990	4875
**NAA20**	0.25913	-0.87738	-0.6460298243820050	4880
**XKR9**	0.74081	-0.22192	-0.6458445383503640	4881
**FPR3**	0.25934	-2.7271	-0.6453814202388300	4882
**VIL1**	0.7406	-0.13411	-0.6451962117506460	4883
**KIAA1024L**	0.74025	-0.0194	-0.6441162695739650	4884
**RTL3**	0.25977	-1.2293	-0.6440545812884100	4885
**PPP3R1**	0.74016	-0.098281	-0.6438386915863890	4886
**THEG**	0.74014	-0.49282	-0.6437770143261610	4887
**PKD2L2**	0.7401	-0.66424	-0.6436536671519540	4888
**C11orf63**	0.26009	-0.60903	-0.6430679017398030	4889
**CCR6**	0.26017	-0.66628	-0.6428213296797540	4890
**CXCL5**	0.26025	-0.32175	-0.642574796695742	4892
**GLRX5**	0.26025	-2.7184	-0.642574796695742	4897
**OR6C6**	0.26025	-1.9406	-0.642574796695742	4893

**RP9**	0.26025	-2.7582	-0.642574796695742	4898
**RPS13**	0.26025	-2.1327	-0.642574796695742	4894
**SAGE1**	0.26025	-2.5921	-0.642574796695742	4896
**TMEM88B**	0.26025	-0.15206	-0.642574796695742	4891
**WDR31**	0.26025	-2.368	-0.642574796695742	4895
**PCDHA6**	0.26027	-0.15946	-0.6425131695521630	4899
**GZMM**	0.2603	-1.6111	-0.6424207334118760	4900
**KREMEN2**	0.2603	-2.1273	-0.6424207334118760	4901
**SPATA24**	0.26032	-0.37288	-0.642359112367783	4902
**USP9Y**	0.26037	-2.7316	-0.6422050704274460	4903
**MAVS**	0.26044	-3.1825	-0.6419894373082670	4904
**ATP5G3**	0.26071	-3.0858	-0.6411579889866210	4909
**BDNF**	0.26071	-2.8912	-0.6411579889866210	4907
**GPCPD1**	0.26071	-1.1128	-0.6411579889866210	4906
**LPAR2**	0.26071	-0.45972	-0.6411579889866210	4905
**TRAPPC6B**	0.26071	-3.5341	-0.6411579889866210	4910
**ZNF665**	0.26071	-3.0808	-0.6411579889866210	4908
**SP7**	0.73927	-0.005590	-0.6410964178577480	4911
**LRRC47**	0.26075	-0.95038	-0.6410348491591860	4912
**FOXL2NB**	0.26078	-2.9521	-0.6409425006672460	4913
**ADHFE1**	0.26119	-1.1005	-0.6396809517997300	4915
**C1orf115**	0.26119	-1.7037	-0.6396809517997300	4916
**GCM2**	0.26119	-0.20312	-0.6396809517997300	4914
**SMG1**	0.26127	-1.8577	-0.6394349146444210	4917
**SPSB1**	0.26132	-0.86519	-0.6392811610773130	4918
**RRS1**	0.26146	-0.69107	-0.6388507314603000	4919
**SLC2A4**	0.26155	-1.0487	-0.6385740891997710	4920
**CORO1C**	0.26165	-3.1268	-0.6382667662161470	4926
**FRYL**	0.26165	-0.64828	-0.6382667662161470	4923
**GABARAP**	0.26165	-0.31175	-0.6382667662161470	4922
**PRKAR2A**	0.26165	-0.26057	-0.6382667662161470	4921
**RUVBL1**	0.26165	-1.5566	-0.6382667662161470	4925

**SLC41A2**	0.26165	-0.85425	-0.6382667662161470	4924
**RASAL3**	0.26166	-2.0294	-0.638236037233559	4927
**SLC24A1**	0.26177	-1.437	-0.6378980581889110	4928
**C3orf62**	0.26193	-2.4026	-0.6374065823358590	4930
**MAPK11**	0.26193	-1.0999	-0.6374065823358590	4929
**LARP7**	0.26217	-1.457	-0.6366696570833080	4931
**ERFE**	0.26227	-2.5477	-0.6363627069327850	4932
**FBXO24**	0.73766	-0.17708	-0.636147877498141	4933
**CCDC153**	0.26242	-3.4844	-0.6359023940885840	4934
**SPEF1**	0.26259	-2.0837	-0.6353808690276280	4935
**ANP32C**	0.26261	-3.7335	-0.6353195245009530	4940
**BRIX1**	0.26261	-3.2697	-0.6353195245009530	4939
**DTWD2**	0.26261	-0.45388	-0.6353195245009530	4937
**PDLIM5**	0.26261	-0.4048	-0.6353195245009530	4936
**SWT1**	0.26261	-2.4091	-0.6353195245009530	4938
**MTRNR2L9**	0.26272	-1.0949	-0.6349821723271460	4941
**B4GALT1**	0.26287	-1.3979	-0.6345222630577750	4942
**BLOC1S1**	0.26288	-1.6605	-0.6344916072123630	4943
**CDK18**	0.2629	-0.20215	-0.6344302973103070	4944
**HMGN5**	0.73693	-0.11262	-0.6339092593822400	4945
**NCDN**	0.73691	-0.29312	-0.63384797211784	4946
**CWC25**	0.2631	-0.76587	-0.63381732937844	4947
**ARPP19**	0.26318	-1.7528	-0.6335722088835870	4949
**BSDC1**	0.26318	-2.4521	-0.6335722088835870	4951
**CBLN4**	0.26318	-0.48285	-0.6335722088835870	4948
**CD48**	0.26318	-2.3293	-0.6335722088835870	4950
**FLJ44635**	0.26318	-2.8883	-0.6335722088835870	4952
**SNX16**	0.26318	-2.9259	-0.6335722088835870	4953
**CR1**	0.26346	-1.194	-0.6327145867142480	4954
**WDTC1**	0.26352	-0.20381	-0.6325308710869430	4955
**TGM5**	0.73634	-0.044107	-0.6321022842844510	4956
**GRB14**	0.26424	-1.6653	-0.6303279453935880	4957

**PDS5A**	0.26429	-3.0968	-0.6301750780940820	4958
**ASXL3**	0.2645	-2.7857	-0.6295331961566230	4967
**CDC16**	0.2645	-1.8419	-0.6295331961566230	4961
**GLYAT**	0.2645	-2.6132	-0.6295331961566230	4966
**HSCB**	0.2645	-1.9232	-0.6295331961566230	4962
**IKZF3**	0.2645	-2.303	-0.6295331961566230	4965
**LCOR**	0.2645	-2.2916	-0.6295331961566230	4964
**NUDT11**	0.2645	-1.6413	-0.6295331961566230	4959
**PCBP4**	0.2645	-2.2072	-0.6295331961566230	4963
**PROX2**	0.2645	-1.7678	-0.6295331961566230	4960
**LHFPL5**	0.26453	-1.9421	-0.6294415199130690	4968
**POLB**	0.26459	-2.5586	-0.6292581832942100	4969
**NSD2**	0.26491	-2.3873	-0.6282807448984940	4970
**ACSF3**	0.73504	-0.15151	-0.6281280743803520	4971
**NEIL3**	0.26496	-0.90191	-0.6281280743803520	4972
**ZNF777**	0.26505	-2.2965	-0.6278533043342980	4973
**CCHCR1**	0.26509	-2.1241	-0.6277311995289320	4974
**ZDHHC11**	0.26519	-1.0349	-0.6274259784519520	4975
**DCAF16**	0.2653	-0.933	-0.6270903027634790	4976
**CFD**	0.26551	-1.5986	-0.6264496634720070	4984
**FPR2**	0.26551	-1.5204	-0.6264496634720070	4982
**GRAPL**	0.26551	-0.2964	-0.6264496634720070	4977
**IPO5**	0.26551	-0.51757	-0.6264496634720070	4978
**IZUMO1R**	0.26551	-1.5423	-0.6264496634720070	4983
**LMTK2**	0.26551	-1.0328	-0.6264496634720070	4979
**MAP1LC3B**	0.26551	-1.3083	-0.6264496634720070	4981
**SPATA6**	0.26551	-1.1558	-0.6264496634720070	4980
**TOMM7**	0.26551	-2.7615	-0.6264496634720070	4985
**PRDX5**	0.26557	-0.23358	-0.6262666708998550	4986
**CTTN**	0.26559	-2.3618	-0.6262056780361250	4987
**MYO18A**	0.26562	-1.0777	-0.626114193108189	4988
**FAM129C**	0.26566	-3.4822	-0.6259922213551140	4990

**FAM160B2**	0.26566	-1.8454	-0.6259922213551140	4989
**ZNF358**	0.26578	-0.56033	-0.625626361956609	4991
**MTIF2**	0.26596	-0.34807	-0.6250777298125390	4992
**CBX6**	0.26598	-0.79784	-0.6250167823008510	4993
**SLC50A1**	0.26607	-2.1311	-0.6247425472223210	4994
**MUC22**	0.26609	-1.5621	-0.6246816124744830	4995
**CPVL**	0.73389	-0.008840	-0.6246206800460350	4996
**BNIP3**	0.73378	-0.0684	-0.624285593130676	4997
**FAM110D**	0.73378	-0.34856	-0.624285593130676	4998
**MTURN**	0.73378	-0.45103	-0.624285593130676	4999
**DAPK2**	0.26657	-0.74686	-0.623219873418086	5000
**RIF1**	0.26663	-2.6735	-0.6230372496945370	5001
**CST11**	0.26686	-2.5613	-0.6223373844678410	5006
**MBOAT2**	0.26686	-2.7825	-0.6223373844678410	5007
**PSMB9**	0.26686	-1.5951	-0.6223373844678410	5003
**TMEM102**	0.26686	-1.8597	-0.6223373844678410	5004
**TRAF3**	0.26686	-2.3044	-0.6223373844678410	5005
**TYW1B**	0.26686	-0.71031	-0.6223373844678410	5002
**STUB1**	0.26701	-1.7799	-0.6218811148182000	5008
**SH2D3C**	0.26705	-1.2211	-0.6217594647775680	5009
**ADGB**	0.26706	-2.9238	-0.6217290537051270	5010
**NUBP1**	0.26706	-3.1228	-0.6217290537051270	5011
**TIAM1**	0.73272	-0.010114	-0.6210601554948850	5012
**NREP**	0.26735	-0.80254	-0.6208473825213930	5013
**KCTD7**	0.26741	-0.92535	-0.6206650280557970	5014
**VAMP3**	0.73247	-0.091218	-0.6203003810243050	5015
**ARRDC2**	0.26759	-2.3352	-0.6201180884369560	5016
**CDHR3**	0.26772	-1.3608	-0.6197231918354330	5017
**CXCL13**	0.26778	-2.6986	-0.6195409644593680	5018
**TRAF2**	0.73216	-0.33934	-0.619358757654042	5019
**CCDC149**	0.26787	-2.0117	-0.6192676619620660	5020
**BEX1**	0.26804	-0.45211	-0.6187515500655230	5021

**ACTR3**	0.26815	-0.89973	-0.6184176831232230	5022
**FAM26F**	0.73183	-0.024923	-0.6183569874492810	5023
**COLEC12**	0.26835	-0.005037	-0.6178108288432640	5024
**CRYBB3**	0.26835	-1.6762	-0.6178108288432640	5027
**LRR1**	0.26835	-1.9505	-0.6178108288432640	5028
**OR4C11**	0.26835	-0.068177	-0.6178108288432640	5025
**PNPLA6**	0.26835	-2.8309	-0.6178108288432640	5032
**SAR1B**	0.26835	-2.101	-0.6178108288432640	5029
**SNRPD3**	0.26835	-2.6305	-0.6178108288432640	5031
**TTC24**	0.26835	-2.2565	-0.6178108288432640	5030
**UBR5**	0.26835	-0.51758	-0.6178108288432640	5026
**LOC1001292**	0.26841	-1.3166	-0.6176288169276330	5033
**IGFL4**	0.26845	-1.7398	-0.6175074870171990	5034
**SVOPL**	0.26846	-1.9963	-0.6174771559599720	5035
**QARS**	0.7315	-0.43576	-0.6173558374111090	5036
**PCSK6**	0.26851	-1.3087	-0.6173255091936590	5037
**CCDC33**	0.26859	-0.81574	-0.6170829038910230	5038
**PQLC2**	0.2686	-0.67524	-0.617052580782077	5039
**DLX2**	0.26862	-0.58627	-0.6169919362662330	5040
**SMARCD1**	0.26873	-1.7488	-0.6166584319782000	5041
**FFAR4**	0.26891	-0.75531	-0.6161128455748080	5042
**MAMDC2**	0.26891	-2.2708	-0.6161128455748080	5043
**MARCH9**	0.26891	-2.9021	-0.6161128455748080	5044
**ZNF34**	0.269	-0.10703	-0.6158401211412260	5045
**ACTR3C**	0.26903	-0.76673	-0.615749223175754	5046
**MYL3**	0.73094	-0.44849	-0.6156583302976090	5047
**OR51A7**	0.26906	-2.8219	-0.6156583302976090	5048
**CD79A**	0.26908	-1.8246	-0.6155977378711530	5049
**DDX56**	0.73088	-0.2499	-0.6154765597980180	5050
**PRR20B**	0.26916	-2.6403	-0.6153553907619800	5051
**HIST1H4A**	0.26923	-2.5802	-0.6151433666850260	5052
**CDH20**	0.73063	-0.032191	-0.6147194014637480	5053

**AHSP**	0.2694	-2.245	-0.6146285661497950	5059
**ENOPH1**	0.2694	-1.2333	-0.6146285661497950	5056
**FBXO34**	0.2694	-2.2838	-0.6146285661497950	5060
**MLEC**	0.2694	-0.74198	-0.6146285661497950	5054
**PPP3CC**	0.2694	-0.84576	-0.6146285661497950	5055
**TSPAN3**	0.2694	-1.9964	-0.6146285661497950	5058
**UMPS**	0.2694	-1.9318	-0.6146285661497950	5057
**SOGA1**	0.7305	-0.13179	-0.6143258183869660	5061
**IL17A**	0.26956	-3.7182	-0.6141441967547190	5063
**PHLDA1**	0.26956	-0.71947	-0.6141441967547190	5062
**CCDC82**	0.26958	-0.75892	-0.6140836607125430	5064
**IQCF2**	0.26988	-0.72364	-0.6131758898986240	5065
**MCC**	0.26992	-0.6311	-0.6130548919747920	5066
**ATP6V0D1**	0.27015	-0.61827	-0.6123593279846760	5067
**NAB2**	0.72955	-0.33988	-0.6114525155666710	5068
**SEMA3A**	0.27047	-1.0809	-0.6113920792874760	5069
**ATR**	0.27049	-0.16312	-0.6113316452413450	5070
**GM2A**	0.27049	-2.3515	-0.6113316452413450	5074
**PRPS2**	0.27049	-1.8239	-0.6113316452413450	5073
**RGPD2**	0.27049	-1.009	-0.6113316452413450	5072
**SCAMP1**	0.27049	-0.26007	-0.6113316452413450	5071
**UBA5**	0.72929	-0.17226	-0.6106670180058050	5075
**VAMP2**	0.27073	-1.1594	-0.6106066107263660	5076
**SUSD6**	0.27076	-1.8574	-0.6105160039846910	5077
**UPK1B**	0.27078	-0.38613	-0.610455602274667	5078
**DGAT1**	0.27084	-2.7418	-0.6102744105056180	5079
**ZNF385B**	0.72911	-0.16536	-0.6101234326685190	5080
**NLRP2**	0.27095	-1.6077	-0.6099422776195470	5082
**ZBTB24**	0.72905	-0.073122	-0.6099422776195470	5081
**HIST1H1T**	0.27099	-2.0554	-0.6098215187066730	5083
**CA5B**	0.27101	-1.133	-0.6097611425850170	5084
**TTC26**	0.27108	-3.025	-0.6095498436602680	5085

**GDPD4**	0.27114	-2.7842	-0.6093687519548130	5087
**ZNF561**	0.72886	-0.080211	-0.6093687519548130	5086
**PLEKHA4**	0.27138	-0.25756	-0.6086445848460180	5088
**SPAG6**	0.2715	-2.0355	-0.6082826209747580	5089
**CD96**	0.27151	-0.10293	-0.6082524609165640	5090
**CPZ**	0.72849	-0.57869	-0.6082524609165640	5091
**RPL32**	0.27151	-0.76849	-0.6082524609165640	5092
**CLDN7**	0.2719	-0.60692	-0.607076649731401	5093
**GALNT4**	0.27195	-2.6473	-0.6069259654230120	5098
**GPC5**	0.27195	-2.3113	-0.6069259654230120	5096
**OSBP2**	0.27195	-0.76369	-0.6069259654230120	5094
**SMPDL3B**	0.27195	-2.526	-0.6069259654230120	5097
**ZKSCAN1**	0.27195	-1.9829	-0.6069259654230120	5095
**ARFGEF1**	0.272	-1.0699	-0.6067752948941390	5099
**CYP11B2**	0.72799	-0.17668	-0.6067451624413830	5100
**C8orf82**	0.7279	-0.28554	-0.6064739951511800	5101
**BAAT**	0.72782	-0.061101	-0.6062329949928010	5102
**G3BP1**	0.27226	-2.726	-0.6059920300400950	5103
**GIMAP7**	0.27243	-3.2003	-0.605480096298649	5106
**MTFR1**	0.27243	-3.6158	-0.605480096298649	5107
**PEMT**	0.27243	-3.1554	-0.605480096298649	5105
**SNAI1**	0.27243	-3.1035	-0.605480096298649	5104
**UBL4B**	0.27264	-1.393	-0.6048479265469890	5108
**ZNF735**	0.27267	-0.96987	-0.6047576363175010	5109
**NR5A1**	0.2728	-1.0703	-0.604366435608151	5110
**DAND5**	0.27286	-1.5071	-0.604185912628836	5111
**TBATA**	0.27291	-0.69947	-0.6040354918522190	5112
**ULBP3**	0.27293	-0.093111	-0.6039753273683750	5113
**GIPC3**	0.27295	-2.8748	-0.6039151650707080	5114
**TAS2R4**	0.2731	-3.3509	-0.6034640174846690	5117
**TMEM231**	0.2731	-0.76916	-0.6034640174846690	5115
**TSPYL6**	0.2731	-3.0841	-0.6034640174846690	5116

**VARS**	0.2731	-3.5629	-0.6034640174846690	5118
**COX6A2**	0.27313	-2.7254	-0.603373802707655	5121
**LIMK2**	0.27313	-2.4499	-0.603373802707655	5119
**MCEE**	0.27313	-2.624	-0.603373802707655	5120
**CYP11B1**	0.27328	-0.76793	-0.6029228024537650	5122
**CCDC43**	0.72671	-0.13866	-0.6028927401311220	5123
**STARD3**	0.72669	-0.087925	-0.6028326171203480	5124
**CCNI2**	0.7266	-0.46011	-0.6025620905320170	5125
**CD164**	0.27357	-0.34931	-0.602051216136034	5126
**AQP1**	0.27362	-0.40657	-0.6019009888657910	5127
**LOXL4**	0.27375	-1.832	-0.6015104615154860	5128
**CUL4B**	0.27386	-2.8951	-0.6011800869364280	5129
**OR4C16**	0.27389	-1.1351	-0.6010899961670190	5130
**IFNK**	0.27393	-3.5209	-0.6009698827295640	5133
**OR2J2**	0.27393	-3.3387	-0.6009698827295640	5132
**TMCO3**	0.27393	-2.7126	-0.6009698827295640	5131
**ZNF624**	0.27393	-3.5443	-0.6009698827295640	5134
**ATL3**	0.72606	-0.38471	-0.6009398557249650	5135
**MRFAP1**	0.27396	-0.45844	-0.6008798033411590	5136
**ALDH3A2**	0.27426	-2.1482	-0.5999792774006910	5143
**CFLAR**	0.27426	-2.1034	-0.5999792774006910	5142
**GNAL**	0.27426	-2.5529	-0.5999792774006910	5145
**LHX4**	0.27426	-2.5751	-0.5999792774006910	5146
**MOCS2**	0.27426	-1.4908	-0.5999792774006910	5139
**OR7E24**	0.27426	-2.3116	-0.5999792774006910	5144
**RP1**	0.27426	-0.4205	-0.5999792774006910	5137
**TMEM206**	0.27426	-1.9801	-0.5999792774006910	5140
**TMEM86A**	0.27426	-0.75165	-0.5999792774006910	5138
**WNT2B**	0.27426	-2.0133	-0.5999792774006910	5141
**C1GALT1C1**	0.27427	-0.077109	-0.5999492682513170	5148
**CA13**	0.27427	-0.01608	-0.5999492682513170	5147
**C9**	0.27431	-0.98833	-0.5998292370561220	5149

**MTMR11**	0.27438	-0.21599	-0.5996192032562290	5150
**ZNF30**	0.27455	-2.0764	-0.5991092312513800	5151
**TMEM119**	0.27457	-2.5704	-0.599049244789892	5152
**MRPL54**	0.27465	-0.45078	-0.5988093204956000	5153
**AKAP12**	0.27479	-2.6862	-0.5983895358974160	5158
**MAP6**	0.27479	-2.3473	-0.5983895358974160	5157
**NFE4**	0.27479	-0.80011	-0.5983895358974160	5154
**PRMT6**	0.27479	-2.2204	-0.5983895358974160	5156
**SPIN4**	0.27479	-1.9309	-0.5983895358974160	5155
**HBG2**	0.72512	-0.10363	-0.5981197300470880	5159
**ZNF461**	0.72501	-0.021631	-0.5977900264572020	5160
**BTNL8**	0.27503	-2.3625	-0.5976701503541770	5161
**EFNA1**	0.27508	-0.34106	-0.5975203173020830	5162
**SH3BGRL2**	0.2751	-2.7152	-0.5974603878372520	5163
**PRND**	0.72482	-0.022171	-0.5972206914316060	5164
**D2HGDH**	0.27523	-0.049988	-0.5970708986017000	5165
**FHAD1**	0.72473	-0.23187	-0.5969510739830820	5166
**PLEKHO2**	0.27541	-2.7538	-0.5965317552906000	5167
**LCE4A**	0.27545	-0.74836	-0.596411969215655	5168
**C19orf54**	0.27546	-0.53755	-0.5963820240340910	5170
**FGF1**	0.27546	-1.225	-0.5963820240340910	5173
**IL7R**	0.27546	-3.5757	-0.5963820240340910	5175
**LOC728485**	0.27546	-2.748	-0.5963820240340910	5174
**RHOU**	0.27546	-1.0056	-0.5963820240340910	5172
**TNFSF9**	0.27546	-0.26161	-0.5963820240340910	5169
**ZSCAN1**	0.27546	-0.77583	-0.5963820240340910	5171
**TRMT2B**	0.72447	-0.3899	-0.5961724227343260	5176
**HIST1H1D**	0.27558	-1.3618	-0.5960227235547900	5177
**CPSF4**	0.27563	-1.3925	-0.5958730377309000	5178
**FAM71A**	0.27568	-1.0462	-0.5957233652569230	5179
**PHF13**	0.27577	-0.14382	-0.5954539884272390	5180
**ZC2HC1C**	0.27577	-1.8529	-0.5954539884272390	5181

**SMIM3**	0.27585	-1.8451	-0.5952145786255620	5182
**OR10A7**	0.27596	-1.1278	-0.5948854458407410	5183
**PADI6**	0.27606	-2.1464	-0.5945862901306900	5184
**PLEKHB1**	0.27616	-1.2974	-0.5942871876234170	5185
**RAB6A**	0.27627	-0.38351	-0.5939582362601190	5186
**SIAE**	0.27627	-3.3884	-0.5939582362601190	5187
**DPH3**	0.27629	-2.5024	-0.5938984338270160	5188
**OVOL1**	0.72363	-0.32751	-0.5936592453300780	5189
**TRIB2**	0.2764	-2.4946	-0.5935695584001000	5190
**LPAR4**	0.72356	-0.51936	-0.5934499832532590	5191
**MFAP3L**	0.27646	-1.1691	-0.5933901988617890	5192
**LARS2**	0.27649	-1.1022	-0.593300526250996	5193
**C9orf40**	0.27655	-0.88424	-0.5931211953399930	5194
**BNIP1**	0.27661	-3.1026	-0.5929418835015780	5200
**CXCL3**	0.27661	-2.8353	-0.5929418835015780	5199
**KRTAP12-4**	0.27661	-1.1183	-0.5929418835015780	5195
**PAIP2B**	0.27661	-2.2542	-0.5929418835015780	5196
**PIH1D3**	0.27661	-2.634	-0.5929418835015780	5198
**TMBIM6**	0.27661	-2.487	-0.5929418835015780	5197
**ACCS**	0.27662	-1.7713	-0.5929120000487660	5201
**TLR6**	0.27675	-3.0379	-0.5925235633279670	5202
**CSNK2A3**	0.72297	-0.15085	-0.5916872338409300	5203
**GET4**	0.27705	-1.3609	-0.5916275118606900	5204
**HLA-B**	0.72291	-0.41333	-0.5915080742301530	5205
**ODF3B**	0.72288	-0.3028	-0.5914185015445270	5206
**ZBTB33**	0.27715	-0.047156	-0.5913289336037800	5207
**ADCY7**	0.27722	-0.90478	-0.5911199601874880	5208
**ATF3**	0.27722	-1.2282	-0.5911199601874880	5209
**CCDC158**	0.27734	-0.63753	-0.5907617800869560	5210
**DNAH12**	0.72247	-0.039229	-0.5901948165336950	5211
**CDC42BPB**	0.27755	-2.8464	-0.5901351471975470	5212
**LETM1**	0.27768	-1.1801	-0.5897473477100870	5213

**SOX11**	0.27783	-1.3026	-0.5892999969400450	5214
**TMEM47**	0.27787	-1.8445	-0.5891807233199340	5215
**POF1B**	0.27801	-0.83116	-0.588763331633054	5216
**SUMO1**	0.27801	-2.0797	-0.588763331633054	5217
**RPIA**	0.27805	-1.4293	-0.5886440957059560	5218
**MTMR6**	0.72188	-0.54669	-0.5884354529680970	5219
**COL5A1**	0.27816	-1.3299	-0.5883162400469990	5221
**PAGE1**	0.27816	-2.4036	-0.5883162400469990	5222
**RPL23A**	0.27816	-2.455	-0.5883162400469990	5223
**TNNI3**	0.27816	-3.3497	-0.5883162400469990	5224
**ZNF570**	0.27816	-0.49357	-0.5883162400469990	5220
**IQCJ**	0.72172	-0.10968	-0.5879586514348850	5225
**ZNF43**	0.72164	-0.20951	-0.5877203007908130	5226
**SLIT2**	0.72152	-0.31063	-0.5873628374134620	5227
**KIAA2026**	0.72149	-0.19182	-0.587273483296808	5228
**TRAK1**	0.27858	-0.64409	-0.5870650085895500	5229
**AFF3**	0.27868	-1.2881	-0.5867672318306390	5230
**FAM3B**	0.2787	-1.0357	-0.586707682722732	5231
**CPA5**	0.27873	-1.2151	-0.5866183629616230	5232
**NAPEPLD**	0.27879	-0.51524	-0.5864397374776930	5233
**GOLGA8H**	0.27886	-0.20262	-0.5862313647260870	5234
**HSDL2**	0.27892	-1.7006	-0.5860527797676960	5235
**KRTAP4-11**	0.27894	-0.40599	-0.5859932556017920	5236
**ENAH**	0.72094	-0.45343	-0.5856361541938230	5237
**LMNB1**	0.2791	-0.21308	-0.5855171369873820	5238
**LILRA4**	0.27915	-0.78726	-0.5853683771415490	5239
**HIST1H3B**	0.27929	-0.50505	-0.5849519184646360	5240
**ABCB1**	0.27932	-1.3676	-0.5848626905197940	5241
**FGGY**	0.27934	-1.5803	-0.5848032078101070	5242
**NTS**	0.27946	-1.6034	-0.5844463549903640	5243
**CD300LF**	0.27952	-0.66395	-0.5842679564892310	5246
**FECH**	0.27952	-2.7496	-0.5842679564892310	5250

**KRTAP19-4**	0.27952	-2.1388	-0.5842679564892310	5248
**MTDH**	0.27952	-2.9115	-0.5842679564892310	5251
**SH2D4B**	0.27952	-0.28173	-0.5842679564892310	5245
**SH3RF1**	0.27952	-0.24272	-0.5842679564892310	5244
**TMEM161B**	0.27952	-2.1497	-0.5842679564892310	5249
**VCX2**	0.27952	-2.0364	-0.5842679564892310	5247
**TMEM50A**	0.27954	-1.2414	-0.5842084944544540	5252
**ING3**	0.27959	-1.2599	-0.5840598484034960	5253
**B4GAT1**	0.7204	-0.41593	-0.5840301207419760	5254
**POGZ**	0.27971	-1.3989	-0.5837031505195160	5255
**XKR4**	0.27971	-3.6636	-0.5837031505195160	5256
**ACTB**	0.27976	-2.7765	-0.5835545483200580	5258
**VPREB3**	0.27976	-2.7606	-0.5835545483200580	5257
**ADAT3**	0.27984	-0.74437	-0.5833168116001760	5259
**SLU7**	0.27989	-0.60546	-0.5831682428911800	5260
**EPC2**	0.7201	-0.16985	-0.5831385306940790	5261
**LAP3**	0.72008	-0.2436	-0.5830791078442270	5262
**VDR**	0.71996	-0.43438	-0.5827226139683180	5263
**GEMIN6**	0.28013	-0.71039	-0.5824552921555970	5264
**PLA1A**	0.28019	-2.6044	-0.5822771007360640	5265
**FRAT2**	0.28021	-0.29215	-0.5822177077048550	5266
**PCDHGA2**	0.28025	-0.63952	-0.5820989278032770	5267
**DPEP1**	0.28044	-2.8908	-0.5815348353694910	5268
**LRRC55**	0.71953	-0.53851	-0.5814457850607910	5269
**TSEN2**	0.71927	-0.066338	-0.5806742087260840	5270
**SLC26A5**	0.28079	-1.1557	-0.5804962017475740	5271
**ATXN1**	0.28081	-2.9618	-0.580436870175665	5279
**C19orf71**	0.28081	-1.7427	-0.580436870175665	5276
**IL6**	0.28081	-2.6185	-0.580436870175665	5278
**KCNN3**	0.28081	-1.4924	-0.580436870175665	5275
**LRP1B**	0.28081	-0.74413	-0.580436870175665	5274
**NOP16**	0.28081	-1.958	-0.580436870175665	5277

**SLC39A13**	0.28081	-0.4379	-0.580436870175665	5273
**SPSB4**	0.28081	-0.11496	-0.580436870175665	5272
**MMP8**	0.28086	-2.15	-0.5802885501841930	5280
**RPS6KL1**	0.719	-0.053435	-0.5798733220984900	5281
**GPR61**	0.28103	-0.85876	-0.5797843576599740	5282
**CRHBP**	0.2811	-2.6474	-0.5795767918125870	5283
**RAMP2**	0.28116	-1.2894	-0.5793988981016680	5284
**NCKAP1L**	0.28121	-3.0194	-0.57925066734808	5285
**PARN**	0.28121	-3.3007	-0.57925066734808	5286
**ST6GAL1**	0.28128	-0.461	-0.5790431656723180	5287
**BAZ1B**	0.28132	-1.407	-0.5789246044797210	5288
**TAX1BP1**	0.71867	-0.14362	-0.578894965453132	5289
**FA2H**	0.28148	-0.084476	-0.5784504410543780	5290
**TEX11**	0.28161	-1.5529	-0.5780652790158200	5291
**FBXL5**	0.28171	-0.90146	-0.5777690588715140	5293
**MOSPD2**	0.28171	-2.2005	-0.5777690588715140	5294
**SH2D6**	0.28171	-0.1108	-0.5777690588715140	5292
**TCAP**	0.71819	-0.11314	-0.5774728894159420	5295
**FAM131A**	0.71818	-0.35021	-0.5774432752565970	5296
**RBM8A**	0.28188	-0.26338	-0.5772656009336420	5297
**TIMM44**	0.28201	-3.0338	-0.5768807023977860	5299
**WDSUB1**	0.28201	-2.9755	-0.5768807023977860	5298
**LBX1**	0.28232	-2.6276	-0.5759632120627520	5300
**KRTAP3-3**	0.71766	-0.17751	-0.5759040357919620	5301
**RPS4Y2**	0.71751	-0.11035	-0.5754602780183000	5302
**PPRC1**	0.28257	-0.71133	-0.5752236535387030	5303
**HSD17B2**	0.28261	-2.2915	-0.575105353376489	5304
**OR2F1**	0.28267	-2.3662	-0.5749279182224100	5305
**GLI2**	0.28273	-1.3032	-0.5747505011671720	5306
**DKK 2.00**	0.28276	-2.3026	-0.5746617994237190	5308
**NR1H3**	0.71724	-0.43681	-0.5746617994237190	5307
**HAND1**	0.28281	-0.64676	-0.5745139732312450	5309

**PAPPA2**	0.28286	-3.3235	-0.5743661595923860	5312
**PDE8A**	0.28286	-2.1956	-0.5743661595923860	5311
**PYROXD1**	0.28286	-3.5741	-0.5743661595923860	5313
**RSF1**	0.28286	-0.92816	-0.5743661595923860	5310
**CNTNAP3**	0.28305	-1.5829	-0.5738045821592080	5318
**CSAG1**	0.28305	-0.51467	-0.5738045821592080	5316
**FPR1**	0.28305	-2.5813	-0.5738045821592080	5323
**KCTD16**	0.28305	-0.51129	-0.5738045821592080	5315
**MPHOSPH1**	0.28305	-2.3878	-0.5738045821592080	5322
**RAB31**	0.28305	-2.2082	-0.5738045821592080	5319
**SLC2A7**	0.28305	-0.62407	-0.5738045821592080	5317
**SPIN3**	0.28305	-2.5986	-0.5738045821592080	5324
**WFDC3**	0.28305	-2.3014	-0.5738045821592080	5321
**WWC1**	0.28305	-2.2652	-0.5738045821592080	5320
**ZNF853**	0.28305	-0.3369	-0.5738045821592080	5314
**AGFG2**	0.28308	-0.95712	-0.5737159285825530	5325
**ALG8**	0.28313	-2.5367	-0.5735681826406480	5326
**DCSTAMP**	0.28315	-0.19566	-0.5735090877696030	5327
**USP38**	0.71682	-0.38267	-0.5734204492180910	5328
**GGTLC2**	0.28324	-2.6423	-0.5732431856290380	5329
**C7orf61**	0.28327	-1.3452	-0.5731545605891880	5330
**GLIPR1L2**	0.28327	-1.5788	-0.5731545605891880	5331
**PRAMEF15**	0.71664	-0.35383	-0.5728887124745050	5332
**NIN**	0.28338	-0.82254	-0.5728296406144850	5333
**CCDC15**	0.28343	-0.055088	-0.5726819697085440	5334
**GBP4**	0.71644	-0.13782	-0.572298083780422	5335
**SNX10**	0.28365	-2.0316	-0.5720323659999440	5336
**GFM1**	0.71633	-0.1205	-0.5719733230894820	5337
**NCAPD2**	0.28381	-1.2075	-0.5715600785258210	5338
**PPIAL4A**	0.28386	-1.223	-0.5714125148367560	5340
**SMIM23**	0.28386	-0.8386	-0.5714125148367560	5339
**WNT10B**	0.71609	-0.28388	-0.5712649635892430	5341

**KCNH6**	0.28416	-2.1022	-0.5705273937891950	5342
**DET1**	0.28431	-0.89012	-0.5700850008683410	5343
**LOC285423**	0.28441	-0.84121	-0.5697901342325210	5344
**CAND2**	0.28463	-1.2003	-0.5691416019307990	5345
**RPSA**	0.28463	-1.7426	-0.5691416019307990	5346
**NUP93**	0.28467	-0.863	-0.5690237126889260	5347
**ZNF260**	0.71531	-0.30947	-0.5689647710335590	5348
**ADGRF2**	0.2847	-0.93261	-0.5689353009471190	5349
**CCDC69**	0.28473	-0.84375	-0.5688468936522740	5350
**ERGIC3**	0.28477	-0.17866	-0.568729024174675	5351
**NSMCE4A**	0.28477	-2.1279	-0.568729024174675	5355
**PRR35**	0.28477	-1.2599	-0.568729024174675	5352
**REEP6**	0.28477	-2.1703	-0.568729024174675	5356
**TBC1D32**	0.28477	-1.7571	-0.568729024174675	5354
**TTLL5**	0.28477	-1.5194	-0.568729024174675	5353
**METTL21A**	0.28479	-2.1292	-0.5686700923989150	5357
**ELL**	0.28482	-0.76064	-0.5685816984381260	5358
**ANKRD63**	0.28496	-2.1456	-0.5681692520079430	5359
**CLPB**	0.28502	-1.1404	-0.5679925188395970	5360
**FAM160A1**	0.28515	-0.6632	-0.5676096578185490	5361
**TRA2A**	0.28527	-1.867	-0.5672563214793910	5362
**BCDIN3D**	0.28528	-0.7971	-0.5672268799815740	5363
**NPY**	0.71471	-0.52238	-0.5671974389754250	5364
**JAG2**	0.28536	-1.1277	-0.5669913656955640	5365
**ATP9B**	0.28538	-1.087	-0.5669324920382250	5366
**CTHRC1**	0.71446	-0.11041	-0.5664615734911630	5367
**MEF2D**	0.28566	-1.6324	-0.5661084670074680	5368
**SQLE**	0.28568	-2.6412	-0.5660496227898790	5369
**MISP3**	0.28578	-2.239	-0.5657554310948450	5370
**BAG1**	0.28583	-3.2631	-0.565608353609023	5374
**LRIG2**	0.28583	-2.9287	-0.565608353609023	5373
**NBPF3**	0.28583	-3.3951	-0.565608353609023	5375

**ODF2L**	0.28583	-2.3472	-0.565608353609023	5371
**WWC2**	0.28583	-2.6048	-0.565608353609023	5372
**NLRP11**	0.28592	-1.1523	-0.5653436449611500	5376
**COPS4**	0.28595	-2.7491	-0.5652554175485390	5377
**FOXA3**	0.71399	-0.40305	-0.5650789759215840	5378
**MSANTD1**	0.71391	-0.19123	-0.5648437477799340	5379
**MIGA1**	0.7138	-0.08597	-0.5645203601064020	5380
**APMAP**	0.28621	-2.4512	-0.5644909641547750	5384
**ARHGEF4**	0.28621	-1.178	-0.5644909641547750	5383
**C11orf58**	0.28621	-1.1574	-0.5644909641547750	5382
**C1orf174**	0.28621	-2.6352	-0.5644909641547750	5385
**COL19A1**	0.28621	-0.57493	-0.5644909641547750	5381
**UNC13C**	0.28621	-2.7324	-0.5644909641547750	5387
**ZNF3**	0.28621	-2.6442	-0.5644909641547750	5386
**RHEB**	0.28628	-2.4154	-0.5642852061489960	5388
**CCNL2**	0.2864	-0.49508	-0.5639325337053680	5389
**CCDC174**	0.71332	-0.37683	-0.5631099039297830	5390
**TFPT**	0.71322	-0.49069	-0.5628161999369180	5391
**CGB1**	0.28679	-3.3764	-0.5627868322081200	5392
**NTN4**	0.28679	-3.4577	-0.5627868322081200	5393
**ABCG5**	0.28683	-1.7855	-0.5626693661462950	5394
**G0S2**	0.28688	-2.4369	-0.5625225444859820	5395
**TBCE**	0.28696	-2.2841	-0.5622876550476300	5396
**ADGRB1**	0.28701	-1.4457	-0.562140864901944	5397
**RPS14**	0.28701	-2.2418	-0.562140864901944	5398
**CYLC2**	0.28708	-1.7684	-0.5619353790445100	5399
**NAT8B**	0.2871	-0.63722	-0.5618766731577650	5400
**RAI14**	0.7128	-0.11264	-0.5615831727632690	5401
**TXNDC12**	0.2872	-0.46962	-0.5615831727632690	5402
**PTGES2**	0.28722	-2.5917	-0.5615244784899060	5403
**PPWD1**	0.71274	-0.017346	-0.5614070957460800	5404
**L1CAM**	0.28728	-1.6313	-0.5613484072749560	5405

**C1orf131**	0.71268	-0.028777	-0.5612310361326410	5406
**MC4R**	0.71256	-0.36227	-0.5608789690814370	5407
**RANBP10**	0.28746	-0.22496	-0.5608202979992410	5408
**MAGEA11**	0.71251	-0.078751	-0.5607322949954520	5409
**ZNF878**	0.2875	-0.65661	-0.5607029616258930	5410
**SERPINA11**	0.28753	-1.209	-0.5606149644117490	5411
**TMEM211**	0.71242	-0.39553	-0.5604683120341580	5412
**BRWD3**	0.28765	-0.86165	-0.5602630189534680	5414
**FAM221B**	0.28765	-0.2396	-0.5602630189534680	5413
**GALNT10**	0.28765	-2.7398	-0.5602630189534680	5420
**GLS**	0.28765	-2.1804	-0.5602630189534680	5419
**HNRNPR**	0.28765	-1.0431	-0.5602630189534680	5415
**NXPE2**	0.28765	-1.1945	-0.5602630189534680	5417
**UBAP1L**	0.28765	-1.1136	-0.5602630189534680	5416
**VPS8**	0.28765	-1.8722	-0.5602630189534680	5418
**IFITM3**	0.28775	-3.2103	-0.5599697840762800	5424
**KDM4A**	0.28775	-2.3043	-0.5599697840762800	5422
**PIF1**	0.71225	-0.097406	-0.5599697840762800	5421
**XRCC6**	0.28775	-2.8826	-0.5599697840762800	5423
**HELQ**	0.28786	-2.9963	-0.559647281314187	5425
**LIX1L**	0.28795	-0.76232	-0.5593834587078980	5426
**TUB**	0.71198	-0.49709	-0.5591782902627860	5427
**C8orf4**	0.28814	-0.29044	-0.5588266276766940	5428
**S100B**	0.28827	-0.93398	-0.5584457378446800	5429
**SCX**	0.28828	-1.6103	-0.5584164419830440	5430
**MAP2K4**	0.71171	-0.057846	-0.5583871466006620	5431
**NOX3**	0.71169	-0.30646	-0.5583285572734990	5432
**PHF6**	0.28834	-0.46749	-0.5582406768761430	5433
**ZNF28**	0.28847	-2.9398	-0.5578599116237990	5434
**DIRC1**	0.28867	-2.7152	-0.5572742767947900	5435
**SNX9**	0.28867	-3.3003	-0.5572742767947900	5436
**TMEM59**	0.28888	-1.6926	-0.5566595658534260	5437

**CARHSP1**	0.28897	-1.9477	-0.5563961826986490	5438
**C1QA**	0.28913	-1.5708	-0.5559280412494100	5441
**LIPT1**	0.28913	-0.45708	-0.5559280412494100	5439
**MARCH3**	0.28913	-2.0106	-0.5559280412494100	5443
**NT5C1B-RD**	0.28913	-2.8187	-0.5559280412494100	5448
**OR9Q2**	0.28913	-2.7198	-0.5559280412494100	5447
**PJA2**	0.28913	-2.2658	-0.5559280412494100	5445
**RELN**	0.28913	-2.6808	-0.5559280412494100	5446
**SRSF1**	0.28913	-2.1738	-0.5559280412494100	5444
**TMEM165**	0.28913	-1.4339	-0.5559280412494100	5440
**TSPAN2**	0.28913	-1.8945	-0.5559280412494100	5442
**SLC25A51**	0.71085	-0.3699	-0.5558695321360190	5449
**DNA2**	0.28927	-0.32331	-0.5555185174045360	5450
**HYLS1**	0.28928	-0.51437	-0.5554892692668790	5451
**GON7**	0.28932	-0.21122	-0.5553722814677460	5452
**RABEP1**	0.28934	-1.6233	-0.555313790418513	5453
**FAM200B**	0.71063	-0.41273	-0.5552260574066710	5454
**RRM2**	0.28946	-1.4864	-0.5549628840074050	5455
**KXD1**	0.28966	-0.85359	-0.5543781917825220	5457
**OCSTAMP**	0.28966	-2.8782	-0.5543781917825220	5458
**TGM7**	0.28966	-0.65476	-0.5543781917825220	5456
**JAKMIP2**	0.28977	-0.29726	-0.5540566918377440	5459
**SORCS3**	0.29	-0.43919	-0.5533846496434850	5460
**C3orf56**	0.29015	-2.4135	-0.5529464958881860	5462
**MAGEB6**	0.29015	-0.23295	-0.5529464958881860	5461
**ADD2**	0.29019	-0.61089	-0.5528296728163590	5463
**WFDC10B**	0.29025	-1.3326	-0.5526544523535170	5464
**YY2**	0.29027	-1.6503	-0.552596049303259	5465
**KANK3**	0.2903	-2.4025	-0.5525084482617890	5466
**SNX4**	0.29037	-2.2349	-0.5523040623176950	5467
**C12orf50**	0.29041	-2.9751	-0.5521872807081570	5468
**MSTN**	0.70954	-0.38691	-0.5520413142853620	5469

**CDH13**	0.29072	-0.32228	-0.5512824783616750	5471
**DCHS2**	0.29072	-3.5237	-0.5512824783616750	5475
**DESI2**	0.29072	-2.0791	-0.5512824783616750	5473
**GULP1**	0.29072	-0.10294	-0.5512824783616750	5470
**KCNA5**	0.29072	-1.2701	-0.5512824783616750	5472
**PI15**	0.29072	-2.2049	-0.5512824783616750	5474
**NFYB**	0.70924	-0.50104	-0.5511657625550740	5476
**KIAA0754**	0.70892	-0.081578	-0.5502323061708030	5477
**TMEM132C**	0.70888	-0.35933	-0.550115657843054	5478
**SAMD4B**	0.29117	-1.6203	-0.5499698579587070	5479
**FAM216B**	0.29128	-0.23865	-0.5496491393555700	5480
**TOX3**	0.29134	-1.6245	-0.5494742257647100	5481
**C12orf60**	0.29145	-1.18	-0.549153594500011	5482
**STK3**	0.29149	-0.008159	-0.5490370153104590	5483
**OR2A2**	0.29152	-0.004999	-0.5489495858149580	5484
**LYL1**	0.29157	-0.92466	-0.5488038793129280	5485
**STXBP5**	0.29159	-1.3023	-0.5487455999745210	5486
**STYK1**	0.29176	-1.2187	-0.5482503008152280	5487
**DDX6**	0.70816	-0.21376	-0.548017265396358	5488
**ANKRD11**	0.70814	-0.39119	-0.5479590111919220	5489
**SLAIN1**	0.29197	-1.396	-0.5476386462972510	5490
**VCP**	0.708	-0.39479	-0.5475512838086010	5491
**EXOSC3**	0.29203	-1.7944	-0.5474639254988000	5492
**PCDHAC1**	0.29206	-3.2729	-0.5473765713667840	5493
**DIRC2**	0.70789	-0.10473	-0.5472309904278240	5494
**MST1L**	0.2922	-0.1749	-0.54696897395897	5495
**TSACC**	0.29224	-0.85012	-0.5468525342484550	5496
**A4GNT**	0.29247	-0.93415	-0.5461831497117910	5500
**ADH7**	0.29247	-0.85986	-0.5461831497117910	5499
**CCP110**	0.29247	-2.3865	-0.5461831497117910	5503
**DHRS11**	0.29247	-2.7374	-0.5461831497117910	5505
**EMC10**	0.29247	-0.44377	-0.5461831497117910	5497

**KCNJ9**	0.29247	-2.5223	-0.5461831497117910	5504
**SNTG1**	0.29247	-2.1643	-0.5461831497117910	5502
**TAF10**	0.29247	-2.7824	-0.5461831497117910	5506
**THSD7B**	0.29247	-0.79575	-0.5461831497117910	5498
**TTC33**	0.29247	-1.0016	-0.5461831497117910	5501
**SIGLEC10**	0.70736	-0.053882	-0.5456885444927060	5507
**TRPC5OS**	0.29271	-2.0378	-0.5454849223219850	5508
**TAF1C**	0.70726	-0.33766	-0.5453976626009880	5509
**LINGO2**	0.29304	-3.178	-0.5445252936108300	5511
**STK10**	0.29304	-3.0821	-0.5445252936108300	5510
**TSHR**	0.70692	-0.59761	-0.5444090090640140	5512
**FRMD5**	0.2931	-1.7275	-0.5443508695511870	5513
**NSDHL**	0.29314	-1.9711	-0.5442345960451300	5514
**ELOF1**	0.29318	-0.28498	-0.5441183298964490	5515
**HMGCS1**	0.70679	-0.33467	-0.5440311351117120	5516
**SYCP3**	0.29325	-1.4847	-0.5439148818324190	5517
**PTPN1**	0.2933	-2.5342	-0.5437695755696470	5518
**HAVCR2**	0.2934	-1.1435	-0.5434789974800270	5519
**NANP**	0.29359	-0.087286	-0.5429270254825300	5520
**IMMT**	0.2937	-0.86765	-0.5426075383474260	5521
**KPNB1**	0.29383	-2.8666	-0.5422300340318660	5523
**ZNF19**	0.29383	-2.6155	-0.5422300340318660	5522
**CENPBD1**	0.29389	-2.2588	-0.5420558273333130	5524
**RANBP9**	0.70604	-0.34504	-0.5418526069739290	5525
**AARD**	0.29397	-0.62941	-0.5418235773208900	5527
**ADORA2B**	0.29397	-0.57564	-0.5418235773208900	5526
**FREM2**	0.29397	-1.1329	-0.5418235773208900	5529
**LOC1019275**	0.29397	-1.2151	-0.5418235773208900	5530
**NEURL3**	0.29397	-1.9943	-0.5418235773208900	5531
**OR4K17**	0.29397	-2.8019	-0.5418235773208900	5533
**OR51I2**	0.29397	-0.80084	-0.5418235773208900	5528
**TRIM23**	0.29397	-2.4501	-0.5418235773208900	5532

**SLC13A1**	0.29412	-0.91812	-0.5413881872957810	5534
**CDK12**	0.29418	-1.2936	-0.5412140600223110	5535
**NUCB2**	0.70581	-0.14398	-0.5411850404055480	5536
**DZIP1**	0.29421	-2.0873	-0.5411270025392260	5537
**PRAMEF26**	0.29437	-1.424	-0.540662765199235	5538
**GPBP1L1**	0.70559	-0.36059	-0.5405467240724850	5539
**TRIM65**	0.29451	-1.5516	-0.5402566530929600	5540
**MVB12A**	0.29461	-0.87492	-0.5399666275644400	5541
**ARHGEF9**	0.70528	-0.022364	-0.5396476519328730	5542
**ARFRP1**	0.29478	-2.5898	-0.5394736883666850	5545
**DDX23**	0.29478	-2.3363	-0.5394736883666850	5544
**TGFBI**	0.29478	-0.66179	-0.5394736883666850	5543
**CHMP1B**	0.70506	-0.089168	-0.5390098653112390	5546
**CFAP52**	0.29498	-0.3439	-0.5388939276675930	5547
**GRAMD2**	0.70495	-0.086363	-0.5386910542185820	5548
**NPM2**	0.70469	-0.30105	-0.5379377182522830	5549
**BMX**	0.2954	-1.912	-0.5376770192444530	5551
**GJB5**	0.2954	-2.4744	-0.5376770192444530	5554
**NCR3**	0.2954	-3.0894	-0.5376770192444530	5555
**TBK1**	0.2954	-2.3147	-0.5376770192444530	5553
**TDRD7**	0.2954	-2.1528	-0.5376770192444530	5552
**TMEM108**	0.2954	-1.5781	-0.5376770192444530	5550
**FAM177B**	0.29555	-1.1674	-0.5372426020793490	5556
**CNTNAP1**	0.70439	-0.54794	-0.5370688636026100	5557
**C1orf50**	0.29569	-0.93672	-0.5368372375144940	5560
**HSD17B4**	0.29569	-2.1015	-0.5368372375144940	5561
**LOC389895**	0.29569	-2.7675	-0.5368372375144940	5562
**SPHKAP**	0.29569	-0.50214	-0.5368372375144940	5558
**TIGIT**	0.29569	-0.77581	-0.5368372375144940	5559
**NCOA3**	0.2957	-1.6268	-0.5368082862787600	5563
**FBXO10**	0.70427	-0.48699	-0.5367214352710140	5564
**PDHB**	0.29577	-1.5976	-0.5366056402246530	5565

**RAE1**	0.70419	-0.53015	-0.5364898523729660	5566
**EXOSC2**	0.29589	-0.56037	-0.53625829824383	5567
**MAPRE2**	0.70406	-0.28969	-0.53611359151364	5568
**PPP1R14D**	0.70398	-0.41657	-0.5358820840922730	5569
**CPE**	0.29633	-0.14159	-0.5349852639044850	5570
**SRGAP2B**	0.29655	-1.1353	-0.5343490718455130	5571
**TDRD3**	0.29661	-2.5489	-0.5341756024597660	5572
**PSMD2**	0.29662	-2.4817	-0.5341466924583330	5573
**CD69**	0.70323	-0.052127	-0.5337130959871390	5574
**TUBA8**	0.29678	-0.97373	-0.533684193124027	5575
**ARSE**	0.29683	-0.42738	-0.5335396854950530	5577
**CD300LD**	0.29683	-0.23571	-0.5335396854950530	5576
**FBXL18**	0.29683	-2.1526	-0.5335396854950530	5581
**HIST2H4B**	0.29683	-1.2616	-0.5335396854950530	5579
**SLC38A7**	0.29683	-0.72784	-0.5335396854950530	5578
**TBX2**	0.29683	-1.3336	-0.5335396854950530	5580
**UBE3C**	0.29683	-3.0496	-0.5335396854950530	5582
**PPIB**	0.29696	-0.84167	-0.5331640177870070	5583
**MAB21L2**	0.29701	-1.2403	-0.5330195502428860	5584
**LCP2**	0.29705	-1.959	-0.5329039842169520	5585
**ADRA2A**	0.70287	-0.5015	-0.532672873513101	5586
**TUBB**	0.29713	-1.0982	-0.532672873513101	5587
**LRIF1**	0.70284	-0.007810	-0.5325862143344200	5588
**TP53TG5**	0.29721	-2.4699	-0.532441791257148	5589
**ZC3H3**	0.70275	-0.015031	-0.5323262607910890	5590
**OR4K14**	0.2973	-2.8473	-0.5321818576992500	5591
**BEX4**	0.29743	-1.067	-0.5318064615799110	5592
**CAD**	0.29752	-2.4306	-0.5315466158570730	5594
**SLMAP**	0.29752	-1.5971	-0.5315466158570730	5593
**IL12A**	0.29765	-2.2144	-0.5311713464920270	5595
**NUDCD2**	0.70217	-0.17546	-0.5306518662064240	5596
**APC**	0.70191	-0.17575	-0.5299017584813750	5597

**ZNF177**	0.7019	-0.4144	-0.5298729141404360	5598
**C10orf91**	0.29818	-1.4023	-0.5296421752803160	5599
**HTATSF1**	0.7018	-0.37292	-0.5295844949715230	5600
**CSTF2**	0.29823	-1.9636	-0.529497977811794	5604
**CUL4A**	0.29823	-2.2975	-0.529497977811794	5605
**MAGEC3**	0.29823	-0.86221	-0.529497977811794	5603
**NLRP6**	0.29823	-0.5632	-0.529497977811794	5601
**SPINT1**	0.29823	-2.3255	-0.529497977811794	5606
**TRIM54**	0.29823	-0.61369	-0.529497977811794	5602
**MSH5**	0.29824	-1.7007	-0.5294691396393230	5607
**PHTF2**	0.29827	-1.27	-0.5293826277637080	5608
**TNNC2**	0.29834	-2.3109	-0.5291807821262720	5609
**NEK7**	0.29842	-1.9132	-0.5289501277940260	5610
**PANX2**	0.29848	-1.2822	-0.5287771555112260	5611
**ATF1**	0.70142	-0.73475	-0.5284889035233810	5613
**HID1**	0.29858	-1.8501	-0.5284889035233810	5614
**OAT**	0.29858	-0.14119	-0.5284889035233810	5612
**HEATR6**	0.70137	-0.27063	-0.5283447939962930	5615
**GNPDA1**	0.29871	-2.5303	-0.5281142415720860	5616
**HNRNPCL2**	0.29871	-2.8353	-0.5281142415720860	5618
**OR2B3**	0.29871	-3.1486	-0.5281142415720860	5619
**PILRA**	0.29871	-2.5627	-0.5281142415720860	5617
**EIF4E**	0.70123	-0.15804	-0.5279413456748500	5620
**SHE**	0.29882	-0.84754	-0.5277972778153110	5621
**PAK1IP1**	0.29884	-0.85817	-0.5277396537388660	5622
**HEXA**	0.29891	-1.1694	-0.5275379832690030	5623
**RNASEH1**	0.70093	-0.58107	-0.5270771027340190	5624
**OR7C1**	0.70089	-0.052462	-0.5269619000953580	5625
**PLCB3**	0.29917	-0.23581	-0.5267891092490170	5626
**FAM9C**	0.29934	-1.5624	-0.5262996205633050	5630
**KCNAB2**	0.29934	-2.1818	-0.5262996205633050	5631
**OR9G4**	0.29934	-0.42598	-0.5262996205633050	5628

**RPL9**	0.29934	-0.3512	-0.5262996205633050	5627
**SSR3**	0.29934	-0.73618	-0.5262996205633050	5629
**TRIM27**	0.29939	-0.82154	-0.5261556773033960	5632
**PAMR1**	0.29948	-0.89123	-0.5258966069030120	5633
**RPS29**	0.70035	-0.031797	-0.5254073479717720	5634
**BCAP29**	0.29974	-1.4362	-0.5251483794532620	5635
**NINL**	0.29976	-1.1109	-0.5250908356767400	5636
**ZFYVE9**	0.29979	-2.5109	-0.5250045232719010	5637
**GPR3**	0.29986	-2.8522	-0.5248031428685050	5639
**LILRB2**	0.29986	-3.4216	-0.5248031428685050	5641
**NUP43**	0.29986	-3.0191	-0.5248031428685050	5640
**PFDN6**	0.29986	-2.0073	-0.5248031428685050	5638
**TMEM39B**	0.29993	-0.95617	-0.5246017837459100	5642
**CHIC2**	0.30001	-1.358	-0.5243716850762570	5643
**ORAI1**	0.30005	-1.2698	-0.5242566461525770	5644
**STUM**	0.69993	-0.54047	-0.5241991292924910	5645
**SPINT3**	0.30011	-2.9332	-0.5240841007743550	5646
**OR14J1**	0.30013	-1.4983	-0.5240265891157130	5647
**IFI44**	0.30018	-1.0477	-0.523882817551328	5648
**BIRC2**	0.69981	-0.20752	-0.5238540645379650	5649
**UBXN10**	0.30021	-2.0679	-0.5237965598104580	5650
**MED28**	0.69976	-0.007276	-0.523710305966694	5651
**LSM 4.00**	0.30025	-3.0509	-0.5236815555512910	5652
**IDH2**	0.3004	-0.42987	-0.5232503512433920	5653
**MGAT3**	0.30047	-0.48013	-0.5230491558620170	5654
**MGAT5B**	0.30055	-1.9607	-0.5228192442059140	5655
**HR**	0.3006	-0.46495	-0.5226755634543060	5657
**IGFBP3**	0.3006	-1.75	-0.5226755634543060	5658
**MCTP1**	0.3006	-2.7045	-0.5226755634543060	5660
**NPSR1**	0.3006	-0.25548	-0.5226755634543060	5656
**S100A12**	0.3006	-2.3649	-0.5226755634543060	5659
**ZNF37A**	0.3006	-2.9019	-0.5226755634543060	5661

**CDO1**	0.30073	-1.2713	-0.5223020439833680	5662
**ALG12**	0.30084	-1.4727	-0.5219860459550860	5663
**FCAR**	0.30101	-2.4228	-0.521497787854822	5665
**HMX1**	0.30101	-0.86729	-0.521497787854822	5664
**WDR70**	0.30101	-3.2914	-0.521497787854822	5667
**ZP2**	0.30101	-3.2721	-0.521497787854822	5666
**ANKRD20A4**	0.30103	-1.1958	-0.521440353900313	5668
**CARTPT**	0.30107	-2.4323	-0.5213254911510460	5669
**SH3D21**	0.30125	-1.9895	-0.5208086938573600	5670
**CHCHD6**	0.30149	-0.95857	-0.5201198470792390	5671
**MINDY4**	0.69838	-0.024899	-0.5197468247854380	5672
**SCGB2A1**	0.30166	-0.16131	-0.5196320632457180	5673
**TVP23A**	0.30166	-0.2761	-0.5196320632457180	5674
**BIRC8**	0.30189	-1.3468	-0.5189723171237210	5675
**LMCD1**	0.30192	-1.1795	-0.5188862799391280	5676
**C10orf99**	0.30195	-0.84231	-0.518800246595393	5678
**DAPP1**	0.30195	-2.4488	-0.518800246595393	5683
**FAM228B**	0.30195	-0.88634	-0.518800246595393	5679
**GREM1**	0.30195	-0.34991	-0.518800246595393	5677
**OR2L8**	0.30195	-2.0624	-0.518800246595393	5681
**OSER1**	0.30195	-0.89122	-0.518800246595393	5680
**SETD5**	0.30195	-2.3254	-0.518800246595393	5682
**IVD**	0.69802	-0.06095	-0.5187142170915360	5684
**ABCC9**	0.30211	-2.8602	-0.5183414669228660	5686
**DDX19A**	0.30211	-0.65109	-0.5183414669228660	5685
**CENPB**	0.69788	-0.45904	-0.5183127968164940	5687
**TMEM189-U**	0.69778	-0.027073	-0.5180261191788320	5688
**KRT78**	0.30234	-1.0717	-0.517682162199521	5689
**RAD18**	0.69753	-0.27624	-0.5173096112382440	5690
**TMEM200B**	0.30251	-2.7464	-0.5171949946191270	5691
**GRK3**	0.30254	-1.2229	-0.5171090366134910	5692
**FBXO18**	0.69742	-0.24085	-0.5169944318823940	5693

**USH2A**	0.30259	-1.9	-0.516965781760644	5694
**NR4A2**	0.30263	-1.9014	-0.5168511855166570	5695
**OXA1L**	0.3027	-0.21055	-0.5166506584197300	5696
**TP53I11**	0.30276	-2.1911	-0.516478794585092	5697
**TBC1D20**	0.30287	-1.0554	-0.5161637505008820	5698
**OTOR**	0.30288	-1.1537	-0.5161351126702800	5699
**IL17REL**	0.3029	-0.59521	-0.5160778382789160	5700
**DEFB127**	0.30297	-0.26768	-0.5158773912386930	5701
**BRSK2**	0.303	-2.4518	-0.5157914917095580	5702
**KIF3B**	0.30303	-1.5479	-0.5157055959861660	5703
**SUSD1**	0.30308	-1.5675	-0.5155624449017530	5704
**GIMAP8**	0.30309	-2.0119	-0.5155338159527340	5707
**OR3A1**	0.30309	-2.2228	-0.5155338159527340	5708
**PEAK1**	0.30309	-1.9192	-0.5155338159527340	5705
**SIGLEC7**	0.30309	-3.1037	-0.5155338159527340	5709
**TECR**	0.30309	-1.9306	-0.5155338159527340	5706
**GRID2**	0.30314	-1.8907	-0.5153906775449680	5711
**TMEM68**	0.30314	-0.12338	-0.5153906775449680	5710
**NEK8**	0.30318	-2.4685	-0.5152761744214030	5712
**RXFP1**	0.30318	-3.1702	-0.5152761744214030	5714
**SLC25A30**	0.30318	-2.8494	-0.5152761744214030	5713
**HS6ST1**	0.30319	-1.6226	-0.515247549696135	5715
**RNF115**	0.69675	-0.5337	-0.515075810209005	5716
**ADAMTSL5**	0.69663	-0.4725	-0.5147323767989270	5717
**ALPI**	0.30347	-2.7716	-0.5144462286616680	5718
**OFCC1**	0.30367	-2.5383	-0.5138740587033910	5719
**LOC100130[illegible]**	0.3037	-0.76399	-0.5137882477224320	5720
**C8orf59**	0.30379	-1.7646	-0.5135308374746090	5721
**OR7D2**	0.30381	-2.4124	-0.5134736398185480	5722
**ZFX**	0.30384	-0.49958	-0.5133878464840450	5723
**PPP1R15B**	0.30395	-0.93462	-0.5130733032453210	5724
**ZNF776**	0.69586	-0.16516	-0.5125301208057660	5725

**KRTAP1-3**	0.30426	-0.43336	-0.5121871361209000	5726
**OR2A7**	0.30426	-3.0747	-0.5121871361209000	5729
**RAB12**	0.30426	-0.84073	-0.5121871361209000	5727
**REXO1**	0.30426	-3.0346	-0.5121871361209000	5728
**CLEC19A**	0.3043	-2.858	-0.5120728212829320	5730
**NPY1R**	0.69569	-0.13018	-0.512044243619055	5731
**MPZL3**	0.69567	-0.13019	-0.5119870895457890	5732
**PPP2R5A**	0.30437	-2.5585	-0.5118727864162150	5733
**CALU**	0.3045	-1.0574	-0.5115013474180340	5734
**CARF**	0.69545	-0.69783	-0.5113585050566180	5735
**CABLES2**	0.30461	-2.46	-0.5111871079941440	5743
**F13A1**	0.30461	-1.5143	-0.5111871079941440	5741
**GTF3A**	0.30461	-0.56943	-0.5111871079941440	5738
**HADHA**	0.30461	-2.7631	-0.5111871079941440	5745
**KY**	0.30461	-0.21813	-0.5111871079941440	5736
**NKPD1**	0.30461	-2.379	-0.5111871079941440	5742
**RFPL4AL1**	0.30461	-1.0871	-0.5111871079941440	5740
**SETD4**	0.30461	-0.37699	-0.5111871079941440	5737
**SLC25A46**	0.30461	-0.67471	-0.5111871079941440	5739
**SWAP70**	0.30461	-2.5533	-0.5111871079941440	5744
**UBE2QL1**	0.30461	-2.8547	-0.5111871079941440	5746
**HIST1H1E**	0.69513	-0.24845	-0.5104445608089400	5747
**CH17-360D5**	0.69509	-0.008336	-0.5103303477672560	5748
**SLC6A17**	0.30492	-1.4164	-0.5103017955470370	5749
**PLAC8**	0.69498	-0.077552	-0.5100162962195510	5750
**KIAA1429**	0.30516	-2.573	-0.509616666983379	5751
**WNT2**	0.30527	-1.7888	-0.5093027296730580	5752
**GYPB**	0.30528	-3.2591	-0.509274192406917	5755
**IL12RB1**	0.30528	-0.3521	-0.509274192406917	5753
**OR6B3**	0.30528	-1.124	-0.509274192406917	5754
**MKLN1**	0.30543	-0.15105	-0.5088461831635370	5756
**CYB5R3**	0.30545	-0.11969	-0.5087891223085600	5757

**PRRC2C**	0.30545	-1.5049	-0.5087891223085600	5758
**KCNJ6**	0.69451	-0.41112	-0.5086750055679770	5759
**FRG2C**	0.30555	-1.1016	-0.508503842876307	5760
**TRAIP**	0.69423	-0.62805	-0.5078763737283150	5761
**ZNF732**	0.30577	-1.6769	-0.5078763737283150	5762
**GPHA2**	0.30581	-2.6815	-0.5077623099162500	5763
**C16orf87**	0.30593	-1.3743	-0.5074201581066700	5764
**ZNF350**	0.30606	-0.63548	-0.5070495606674240	5765
**DEFA5**	0.30607	-2.1631	-0.5070210560570320	5770
**FNBP1L**	0.30607	-1.6528	-0.5070210560570320	5767
**KRTAP10-7**	0.30607	-0.53782	-0.5070210560570320	5766
**LGI1**	0.30607	-2.2734	-0.5070210560570320	5771
**MROH5**	0.30607	-2.7602	-0.5070210560570320	5772
**OTX2**	0.30607	-2.1614	-0.5070210560570320	5769
**SEPSECS**	0.30607	-1.8974	-0.5070210560570320	5768
**GLYATL1**	0.3062	-1.6301	-0.506650533597375	5773
**FOXM1**	0.30622	-1.6489	-0.5065935363165080	5774
**ARHGEF17**	0.30625	-0.11401	-0.5065080434808640	5775
**TMEM134**	0.69368	-0.051612	-0.5063085745925220	5776
**ADGRF3**	0.30633	-2.3253	-0.5062800806816540	5777
**CDHR1**	0.30633	-2.669	-0.5062800806816540	5778
**DEPDC5**	0.30633	-3.2441	-0.5062800806816540	5779
**MSX2**	0.3064	-1.0702	-0.506080634812947	5780
**EIF2AK4**	0.30643	-2.1053	-0.5059951641748100	5781
**PDAP1**	0.3065	-2.2764	-0.505795747058607	5782
**KCP**	0.69306	-0.019872	-0.5045427280875340	5783
**RNF169**	0.30696	-1.8422	-0.5044857915124350	5784
**INHBB**	0.69302	-0.28358	-0.5044288565727300	5785
**GABRB3**	0.30702	-0.53007	-0.5043149915983860	5786
**SQOR**	0.30712	-2.169	-0.5040303577624310	5787
**BEST1**	0.69277	-0.16062	-0.5037173076992470	5788
**NTRK2**	0.30725	-3.0262	-0.5036603948101200	5789

**ANKDD1B**	0.30742	-1.9955	-0.5031767010906630	5792
**CLEC4A**	0.30742	-1.0301	-0.5031767010906630	5790
**DMKN**	0.30742	-2.5563	-0.5031767010906630	5794
**DRC1**	0.30742	-2.5752	-0.5031767010906630	5795
**EPHB6**	0.30742	-1.801	-0.5031767010906630	5791
**OR8H1**	0.30742	-2.3988	-0.5031767010906630	5793
**C18orf8**	0.30744	-2.805	-0.5031198036882430	5798
**DOCK2**	0.30744	-3.0291	-0.5031198036882430	5800
**ELOA2**	0.30744	-2.6	-0.5031198036882430	5796
**N4BP2**	0.30744	-2.7158	-0.5031198036882430	5797
**PCSK1N**	0.30744	-2.98	-0.5031198036882430	5799
**ZDHHC17**	0.30747	-1.426	-0.5030344606383810	5801
**IQGAP1**	0.69225	-0.12217	-0.5022381020522580	5802
**JPH2**	0.30791	-0.38542	-0.5017831830014300	5803
**GOSR2**	0.30815	-0.67487	-0.501100999046833	5804
**ZNF226**	0.30829	-1.7815	-0.5007031660942180	5805
**GDF3**	0.30846	-1.0969	-0.5002201897321490	5806
**FLOT1**	0.30849	-2.3376	-0.5001349707239840	5808
**OR52A5**	0.69151	-0.37773	-0.5001349707239840	5807
**SHOX**	0.30849	-3.2264	-0.5001349707239840	5809
**NRG1**	0.30858	-1.5056	-0.4998793354877300	5810
**ZCCHC24**	0.69132	-0.049181	-0.4995953346435560	5811
**SHANK3**	0.30877	-1.0428	-0.4993397683328870	5812
**CNOT4**	0.30885	-0.86371	-0.49911262565605800	5813
**GPR6**	0.30893	-2.6006	-0.4988855087276190	5819
**INAFM2**	0.30893	-1.9223	-0.4988855087276190	5818
**MRGPRX2**	0.30893	-0.068689	-0.4988855087276190	5814
**NOTUM**	0.30893	-2.7811	-0.4988855087276190	5820
**PCP4**	0.30893	-1.8609	-0.4988855087276190	5817
**PNMA3**	0.30893	-1.6274	-0.4988855087276190	5815
**SART1**	0.30893	-1.8363	-0.4988855087276190	5816
**PRAMEF20**	0.309	-0.83279	-0.4986868025231630	5821

**C19orf24**	0.30902	-2.6618	-0.4986300329386450	5823
**CDH9**	0.30902	-0.091261	-0.4986300329386450	5822
**ADGRA2**	0.30915	-0.28105	-0.49826106979630700	5824
**GLOD5**	0.30919	-0.91309	-0.49814755632297800	5825
**ZNF268**	0.30922	-1.2172	-0.49806242543022300	5826
**ZFP57**	0.30933	-2.5844	-0.49775030969742300	5827
**MYBL2**	0.69066	-0.052576	-0.4977219379446350	5828
**APOL4**	0.30939	-2.2165	-0.49758008518963800	5830
**PRADC1**	0.30939	-1.0288	-0.49758008518963800	5829
**RPL36**	0.30944	-1.3564	-0.4974382424464170	5831
**ZNF497**	0.69048	-0.65016	-0.49721131487111100	5832
**FOXB2**	0.30953	-2.8843	-0.49718295072467900	5833
**MKL1**	0.69038	-0.17573	-0.49692769140240600	5834
**ANP32E**	0.30966	-2.9402	-0.49681425320805900	5835
**LIMK1**	0.6903	-0.13452	-0.49670082140651500	5836
**SLC37A2**	0.3097	-0.96862	-0.49670082140651500	5837
**ZNF569**	0.3097	-1.8176	-0.49670082140651500	5838
**TM4SF1**	0.30979	-2.6904	-0.49644562321544600	5839
**EFNA5**	0.6902	-0.4279	-0.49641726985687500	5840
**MPHOSPH6**	0.30987	-0.87029	-0.49621880751903600	5841
**SYCP1**	0.31	-1.3302	-0.49585028644568600	5842
**CATSPER1**	0.31005	-1.0513	-0.49570856550311400	5843
**FRMPD1**	0.31016	-1.5916	-0.49539681446717600	5844
**MRM3**	0.31017	-0.029596	-0.49536847585169	5845
**GLP2R**	0.68968	-0.19318	-0.49494344433839300	5846
**NOV**	0.31042	-3.3857	-0.49466013966674900	5848
**STRAP**	0.31042	-2.465	-0.49466013966674900	5847
**LONRF2**	0.31046	-0.050522	-0.4945468289151250	5849
**OR1L8**	0.31055	-2.3606	-0.4942919029354470	5850
**UBA7**	0.31067	-1.3138	-0.493952051587637	5851
**APITD1-COI**	0.3107	-2.0225	-0.4938670976657460	5859
**AURKB**	0.3107	-1.182	-0.4938670976657460	5855

**HIST1H3G**	0.3107	-1.69	-0.4938670976657460	5857
**NFE2L2**	0.3107	-0.92615	-0.4938670976657460	5852
**NOX4**	0.3107	-1.0678	-0.4938670976657460	5854
**PCDHA11**	0.3107	-1.3473	-0.4938670976657460	5856
**PRSS58**	0.3107	-1.8838	-0.4938670976657460	5858
**PTPN12**	0.3107	-2.3361	-0.4938670976657460	5860
**SMARCD3**	0.3107	-2.4592	-0.4938670976657460	5861
**ZNF33A**	0.3107	-0.99299	-0.4938670976657460	5853
**TMOD4**	0.3108	-0.2236	-0.49358394366242700	5862
**PLEC**	0.31081	-0.82652	-0.49355563043891800	5863
**TMEM9B**	0.68918	-0.338	-0.4935273176110580	5864
**MRPS7**	0.31084	-1.3572	-0.4934706931421570	5865
**RRAD**	0.6891	-0.36389	-0.49330082922756000	5866
**RIC8B**	0.31132	-1.4319	-0.49211217992755100	5867
**FABP6**	0.31139	-1.0573	-0.49191413933766900	5868
**DDN**	0.31147	-0.62338	-0.4916878308561250	5869
**NDUFAF7**	0.68844	-0.53344	-0.49143326391083300	5870
**ARL6IP1**	0.31163	-2.4964	-0.4912352894141760	5872
**TRIM16**	0.31163	-2.4959	-0.4912352894141760	5871
**HLCS**	0.31174	-0.61099	-0.4909242255255830	5873
**PHACTR1**	0.31186	-1.0548	-0.4905849372683200	5874
**PTTG1IP**	0.31191	-1.2259	-0.4904435838296000	5875
**HMBS**	0.31195	-1.3808	-0.49033050813399600	5876
**NCBP1**	0.68803	-0.13522	-0.490273972637248	5877
**TBC1D3C**	0.31204	-1.2468	-0.49007611073710000	5878
**SARAF**	0.68791	-0.69521	-0.4899347925546660	5879
**USHBP1**	0.31228	-1.0841	-0.4893978726541430	5880
**NARS**	0.3123	-2.1659	-0.4893413629789200	5881
**C2orf66**	0.31233	-1.1965	-0.48925660139588300	5882
**IGSF10**	0.31238	-1.1081	-0.48911533990139600	5883
**UBE4A**	0.31239	-0.52162	-0.48908708877378400	5884
**ARF1**	0.68758	-0.65279	-0.4890023377329200	5885

**ZDHHC3**	0.31249	-1.5751	-0.4888045989614620	5886
**DNAJC14**	0.31252	-0.98545	-0.488719859624624	5889
**GNRHR**	0.31252	-2.7162	-0.488719859624624	5894
**GPR156**	0.31252	-2.4293	-0.488719859624624	5893
**LIPJ**	0.31252	-1.8967	-0.488719859624624	5891
**LMNTD1**	0.31252	-2.3119	-0.488719859624624	5892
**NR2C1**	0.31252	-0.74538	-0.488719859624624	5888
**PLK4**	0.31252	-0.23145	-0.488719859624624	5887
**SMAD6**	0.31252	-1.612	-0.488719859624624	5890
**SNX13**	0.68725	-0.16628	-0.488070307894572	5895
**CTPS2**	0.31281	-2.8421	-0.4879008934898270	5900
**DMRTC1B**	0.31281	-0.57755	-0.4879008934898270	5896
**SH2B3**	0.31281	-2.3272	-0.4879008934898270	5899
**SPTBN4**	0.31281	-2.3193	-0.4879008934898270	5898
**WDR90**	0.31281	-1.2454	-0.4879008934898270	5897
**CASD1**	0.31289	-2.5968	-0.4876750293969640	5901
**OR10A4**	0.31294	-2.0833	-0.48753387697234400	5902
**LILRB1**	0.31301	-1.0451	-0.4873362798948440	5903
**EPM2A**	0.31314	-0.92876	-0.4869693643627320	5904
**SLC13A5**	0.31315	-1.3933	-0.48694114280696600	5905
**SPP1**	0.68683	-0.6299	-0.48688470085887600	5906
**ZC3HC1**	0.31331	-2.1142	-0.48648965063637100	5907
**GDPD3**	0.31341	-1.8421	-0.4862075183867210	5908
**SH3BGRL**	0.31359	-1.1543	-0.4856997778301400	5909
**GPR180**	0.3136	-1.263	-0.48567157369372900	5910
**PGLS**	0.68631	-0.28265	-0.48541775384721000	5911
**B3GNT8**	0.31372	-0.98953	-0.4853331541816010	5912
**PHYHIP**	0.31383	-2.0278	-0.4850229851183370	5913
**CES3**	0.31388	-1.4127	-0.4848820146062350	5914
**HMGB4**	0.31389	-2.7397	-0.48485382166018500	5917
**PKD1L1**	0.31389	-1.9141	-0.48485382166018500	5915
**SLC25A27**	0.31389	-1.9894	-0.48485382166018500	5916

**HHEX**	0.31397	-2.0293	-0.48462829196269800	5919
**MRLN**	0.68603	-0.067469	-0.4846282919626980	5918
**MRS2**	0.31421	-0.37511	-0.48395185068757900	5920
**ARHGAP29**	0.31425	-0.62905	-0.48383913200965000	5923
**GNA14**	0.31425	-1.0316	-0.48383913200965000	5924
**REPS1**	0.31425	-0.21845	-0.48383913200965000	5921
**RFTN2**	0.31425	-2.0056	-0.48383913200965000	5926
**SELPLG**	0.31425	-2.6257	-0.48383913200965000	5928
**SULT1E1**	0.31425	-2.521	-0.48383913200965000	5927
**SURF4**	0.31425	-1.8885	-0.48383913200965000	5925
**VPS28**	0.31425	-0.41058	-0.48383913200965000	5922
**ARNT**	0.31427	-0.78052	-0.4837827749759900	5929
**CNOT1**	0.31432	-0.30579	-0.48364188911354000	5930
**NAT1**	0.31434	-1.7465	-0.4835855374565490	5931
**TAF5**	0.31439	-0.6694	-0.48344466503175100	5932
**USP53**	0.31439	-2.4238	-0.48344466503175100	5933
**SRRM2**	0.31445	-0.17835	-0.4832756307849190	5934
**KIAA1841**	0.68549	-0.3545	-0.48310661034549500	5935
**HES7**	0.31454	-0.64046	-0.48302210530134700	5936
**CCDC148**	0.31461	-0.89252	-0.4828249402770140	5937
**KRTAP20-3**	0.31463	-2.44	-0.482768610860541	5938
**RWDD2A**	0.31467	-2.9249	-0.4826559566227240	5939
**MZT2A**	0.31477	-0.47796	-0.4823743478208650	5940
**FAM181A**	0.31491	-0.66166	-0.4819801597493930	5941
**ZMAT1**	0.31496	-1.3972	-0.4818393964465200	5942
**F7**	0.31497	-2.9893	-0.4818112449316850	5946
**TUBB4B**	0.31497	-0.66838	-0.4818112449316850	5943
**UBTFL1**	0.31497	-2.6869	-0.4818112449316850	5945
**VPS29**	0.31497	-0.87582	-0.4818112449316850	5944
**WDR38**	0.31502	-1.151	-0.48167049308441100	5947
**GOLGB1**	0.31507	-1.9401	-0.48152975077897700	5948
**TPP1**	0.68489	-0.30216	-0.4814171638020130	5949

**MCCD1**	0.31542	-0.17821	-0.4805448214698520	5950
**CRIM1**	0.31549	-0.51809	-0.48034789156702700	5951
**CYTL1**	0.31553	-0.34081	-0.48023536855781600	5952
**PPP1R15A**	0.31553	-1.0584	-0.48023536855781600	5953
**FSD1L**	0.31561	-0.69344	-0.4800103407778610	5954
**C2CD4C**	0.31575	-1.7994	-0.4796166006346420	5958
**IFITM5**	0.31575	-2.1846	-0.4796166006346420	5959
**IFNA13**	0.31575	-2.1998	-0.4796166006346420	5960
**OR4K2**	0.31575	-0.28132	-0.4796166006346420	5955
**PAWR**	0.31575	-0.28342	-0.4796166006346420	5956
**TBL1XR1**	0.31575	-0.70881	-0.4796166006346420	5957
**TSPAN18**	0.31575	-2.9068	-0.4796166006346420	5961
**PSEN1**	0.31579	-0.60852	-0.4795041171094360	5962
**EPHX3**	0.31584	-2.1402	-0.479363521233935	5963
**KDM5D**	0.31589	-0.95097	-0.47922293483354300	5964
**CPNE2**	0.3159	-1.2173	-0.4791948186901210	5965
**ZNF345**	0.31609	-0.87665	-0.4786606839019840	5966
**KRTAP13-3**	0.31643	-0.34974	-0.47770520436178500	5967
**RPP38**	0.31643	-2.3834	-0.47770520436178500	5968
**SLC45A1**	0.31643	-2.936	-0.47770520436178500	5969
**ASIC1**	0.31651	-1.2068	-0.4774804490453420	5971
**ZNF653**	0.68349	-0.44128	-0.47748044904534200	5970
**TRIM66**	0.31665	-0.80077	-0.4770871852643610	5972
**HIST1H4F**	0.68333	-0.12565	-0.47703101074843200	5973
**ZNF582**	0.31676	-1.5819	-0.4767782440508600	5974
**ANKRD60**	0.6832	-0.027682	-0.47666591307455200	5975
**FKBP1B**	0.31705	-0.90146	-0.47596398065498000	5976
**GSTM5**	0.68293	-0.17813	-0.47590783619567400	5977
**AUTS2**	0.31709	-0.481	-0.47585169323649700	5978
**LOC100131**	0.31711	-0.79071	-0.47579555177718900	5979
**PIH1D2**	0.68286	-0.19544	-0.4757113423999260	5980
**USP48**	0.31714	-1.6004	-0.4757113423999260	5981

**ABI3**	0.31722	-0.81915	-0.4754868005497140	5983
**IRX3**	0.31722	-2.6846	-0.4754868005497140	5987
**PEG10**	0.31722	-2.2174	-0.4754868005497140	5986
**SERPINA6**	0.31722	-0.79299	-0.4754868005497140	5982
**UNC5A**	0.31722	-1.7427	-0.4754868005497140	5985
**WNT8A**	0.31722	-1.7033	-0.4754868005497140	5984
**TMEM8A**	0.31726	-0.43538	-0.47537453861484100	5988
**DPPA5**	0.68261	-0.13574	-0.47500972868947900	5989
**STARD5**	0.31739	-1.0499	-0.47500972868947800	5990
**OR10C1**	0.31743	-1.6263	-0.4748974922011090	5991
**TTC31**	0.68246	-0.23087	-0.47458887269673900	5992
**GOLGA1**	0.31762	-0.63265	-0.4743644505383750	5993
**API5**	0.31764	-2.8227	-0.4743083487321040	5994
**LOC650293**	0.31764	-3.2561	-0.4743083487321040	5996
**PCDH20**	0.31764	-3.0657	-0.4743083487321040	5995
**NFAM1**	0.31772	-0.55546	-0.47408395643261900	5997
**CFAP54**	0.31783	-0.69021	-0.47377545599055700	5998
**ATP5G2**	0.3179	-0.54673	-0.47357916100368400	6000
**WDR77**	0.3179	-0.21354	-0.47357916100368400	5999
**OR10K2**	0.68203	-0.1476	-0.47338288426307600	6001
**SNRPF**	0.31798	-0.92838	-0.473354846217555	6002
**CNBP**	0.68185	-0.70718	-0.47287825635162400	6003
**HSPA9**	0.31838	-2.746	-0.47223362919295400	6004
**OXTR**	0.68152	-0.13733	-0.47195341772705200	6005
**CCT7**	0.31855	-0.82568	-0.47175729174901700	6006
**MAN1A1**	0.3186	-1.4408	-0.47161721287440000	6007
**SLC15A1**	0.68132	-0.309	-0.47139310592079800	6008
**DSCAML1**	0.31872	-0.83711	-0.4712810613223480	6009
**EIF3F**	0.68123	-0.11846	-0.4711410138936930	6010
**CLDN12**	0.31879	-0.5529	-0.471084997509676	6011
**DKK 4.00**	0.31881	-0.064085	-0.47102898260381900	6015
**APOE**	0.31881	-1.1965	-0.47102898260381900	6013

**ARMC5**	0.31881	-0.53656	-0.47102898260381900	6012
**FOXB1**	0.31881	-1.3292	-0.47102898260381900	6016
**G6PC2**	0.31881	-2.6299	-0.47102898260381900	6019
**HHIPL2**	0.31881	-2.8185	-0.47102898260381900	6020
**OR4M1**	0.31881	-1.9084	-0.47102898260381900	6018
**RAB28**	0.31881	-1.4816	-0.47102898260381900	6017
**SIX1**	0.31881	-0.88259	-0.47102898260381900	6014
**ZDBF2**	0.31881	-2.8505	-0.47102898260381900	6021
**PRPH**	0.68115	-0.33217	-0.4709169572255690	6022
**SRSF4**	0.681	-0.17335	-0.47049691467288200	6023
**IRF4**	0.68094	-0.082376	-0.47032892089687300	6024
**FKBP7**	0.31916	-1.299	-0.47004896076155700	6025
**IDI2**	0.31922	-1.2204	-0.46988100236404700	6026
**SF3B2**	0.31928	-2.6049	-0.4697130572209470	6027
**LDHA**	0.31943	-0.85596	-0.4692932523064400	6028
**COTL1**	0.3197	-0.84606	-0.4685378117695680	6029
**ADGRG2**	0.31972	-0.74035	-0.4684818638521290	6030
**PTPRQ**	0.31974	-1.6569	-0.4684259174010900	6031
**ADTRP**	0.68021	-0.40817	-0.46828605768734300	6032
**RDX**	0.31988	-0.65813	-0.46803433328187400	6033
**HS3ST6**	0.68005	-0.098895	-0.4678385681357510	6034
**PDF**	0.31995	-2.9908	-0.4678385681357510	6037
**SAG**	0.31995	-2.5187	-0.4678385681357510	6035
**TOP1MT**	0.31995	-2.6761	-0.4678385681357510	6036
**ATPAF1**	0.68002	-0.38386	-0.46775467427618600	6038
**COA3**	0.32	-0.4967	-0.46769874686543900	6039
**KIR3DL2**	0.6799	-0.38073	-0.4674191317498160	6040
**ELMO3**	0.32042	-0.60313	-0.46652460877723100	6042
**KRT24**	0.32042	-1.8053	-0.46652460877723100	6045
**NRG3**	0.32042	-1.5771	-0.46652460877723100	6044
**OAZ2**	0.32042	-2.2463	-0.46652460877723100	6046
**OR10AG1**	0.32042	-0.4604	-0.46652460877723100	6041

**SLC25A37**	0.32042	-2.5229	-0.46652460877723100	6047
**TSN**	0.32042	-0.86456	-0.46652460877723100	6043
**NUDT12**	0.32043	-1.4398	-0.4664966609523000	6049
**ZNF276**	0.67957	-0.58668	-0.4664966609523000	6048
**C14orf1**	0.6793	-0.10499	-0.46574220730832100	6050
**SPA17**	0.6791	-0.21289	-0.46518352364814700	6051
**PFKFB4**	0.67903	-0.28936	-0.4649880186739050	6052
**ZFP41**	0.67898	-0.78707	-0.46484838314479500	6053
**STEAP3**	0.67888	-0.28805	-0.4645691392720760	6054
**GIMAP1**	0.32113	-2.5453	-0.4645412168774920	6056
**IL22RA1**	0.32113	-3.0043	-0.4645412168774920	6057
**PIGV**	0.32113	-0.48341	-0.4645412168774920	6055
**ANKEF1**	0.67885	-0.064807	-0.46448537317484100	6058
**ZNF567**	0.67881	-0.44282	-0.4643736901147310	6059
**NDUFB4**	0.32125	-2.076	-0.46420617638380400	6060
**C17orf53**	0.32128	-2.2662	-0.4641224244031760	6061
**STK31**	0.67871	-0.19013	-0.4640945077997720	6062
**PGGT1B**	0.32139	-0.13966	-0.46381536165321400	6063
**SUSD4**	0.32157	-0.24213	-0.4633129896344730	6064
**TAF12**	0.67837	-0.098613	-0.46314555828240100	6065
**SI**	0.32174	-0.38583	-0.4628386345173880	6066
**SLCO1B3**	0.67826	-0.51229	-0.4628386345173880	6067
**TMEM63C**	0.32174	-2.5548	-0.4628386345173880	6068
**TOMM40L**	0.32174	-2.8243	-0.4628386345173880	6069
**ZHX1-C8orf**	0.67822	-0.095084	-0.4627270366863050	6070
**ZNF550**	0.32179	-1.3175	-0.4626991381290160	6071
**HTR1A**	0.32189	-1.0093	-0.46242017235818300	6073
**RBBP6**	0.67811	-0.54335	-0.46242017235818300	6072
**RAB5B**	0.32194	-0.78528	-0.46228070296797300	6074
**C20orf173**	0.32199	-2.5682	-0.4621412425694020	6075
**SCGB1A1**	0.32203	-1.5563	-0.4620296807219250	6076
**DGKK**	0.32205	-0.36139	-0.4619739019546310	6077

**SZT2**	0.6779	-0.85036	-0.46183446132404300	6078
**ERICH3**	0.32212	-0.33155	-0.4617786875862160	6081
**HEPACAM**	0.32212	-0.2218	-0.4617786875862160	6080
**NOG**	0.67788	-0.15202	-0.4617786875862160	6079
**NUP188**	0.6778	-0.35353	-0.46155560699676800	6082
**SLC32A1**	0.67779	-0.014775	-0.4615277235382870	6083
**HARBI1**	0.32222	-0.8269	-0.4614998404386350	6084
**UPB1**	0.67777	-0.055749	-0.46147195769778200	6085
**SPRR2D**	0.32228	-0.47222	-0.4613325493744240	6086
**ZCCHC3**	0.32228	-3.1347	-0.4613325493744240	6087
**ANXA1**	0.3223	-1.0065	-0.461276788555593	6088
**OR5AN1**	0.32236	-1.2174	-0.4611095147033580	6089
**SPICE1**	0.32257	-2.8119	-0.4605241577737090	6090
**IFNL1**	0.32282	-2.334	-0.4598275099506650	6091
**TSPOAP1**	0.67714	-0.30834	-0.45971606701449400	6092
**GTF3C4**	0.32288	-0.23816	-0.4596603476874850	6093
**DOLK**	0.32301	-1.6417	-0.45929820683541500	6098
**RNF213**	0.32301	-2.8562	-0.45929820683541500	6101
**SPDEF**	0.32301	-1.4933	-0.45929820683541500	6097
**TIRAP**	0.32301	-1.3529	-0.45929820683541500	6095
**TMEM30B**	0.32301	-1.755	-0.45929820683541500	6099
**TMUB2**	0.32301	-1.3988	-0.45929820683541500	6096
**UTS2**	0.32301	-2.202	-0.45929820683541500	6100
**ZCCHC14**	0.32301	-0.94571	-0.45929820683541500	6094
**CREB1**	0.32304	-1.2911	-0.4592146444247550	6102
**TMEM60**	0.32316	-0.25935	-0.4588804268383050	6103
**FOXJ1**	0.32324	-0.82874	-0.45865764358956500	6104
**EIF4A1**	0.3233	-1.9201	-0.4584905710915090	6105
**NEFH**	0.3233	-2.3097	-0.4584905710915090	6106
**ZC3H4**	0.3233	-2.3793	-0.4584905710915090	6107
**ASPHD1**	0.67661	-0.18514	-0.4582399863368520	6108
**ECI1**	0.3235	-0.28717	-0.45793375515071000	6109

**NEK1**	0.3235	-1.4129	-0.45793375515071000	6110
**RIOX1**	0.32367	-0.080399	-0.4574605732145280	6111
**AP4M1**	0.32378	-1.2329	-0.45715445124552500	6112
**SCRN3**	0.32407	-1.3116	-0.4563476076346470	6113
**PRAMEF6**	0.67588	-0.09142	-0.4562085267031640	6114
**TMC6**	0.67588	-0.72523	-0.4562085267031640	6115
**GCSH**	0.6757	-0.31797	-0.45570790838669900	6116
**SNX25**	0.67562	-0.36735	-0.4554854480192820	6117
**RPL36A-HN**	0.3244	-1.7105	-0.45542983644979100	6118
**DMRTA2**	0.67553	-0.21005	-0.4552352070465880	6119
**CADPS**	0.3245	-2.4056	-0.45515179972435	6122
**HS3ST4**	0.3245	-2.486	-0.45515179972435	6123
**IRAK1BP1**	0.3245	-2.8207	-0.45515179972435	6124
**P4HA3**	0.3245	-1.9517	-0.45515179972435	6121
**RNF141**	0.3245	-0.23617	-0.45515179972435	6120
**CINP**	0.32462	-1.2557	-0.45481820209014800	6125
**FLG**	0.32463	-3.0722	-0.45479040457242000	6130
**G3BP2**	0.32463	-3.0614	-0.45479040457242000	6129
**GRASP**	0.32463	-2.5599	-0.45479040457242000	6126
**RBM44**	0.32463	-2.5887	-0.45479040457242000	6127
**TFRC**	0.32463	-2.6817	-0.45479040457242000	6128
**AUH**	0.32481	-0.43623	-0.45429010931599800	6131
**DUSP6**	0.32522	-1.0689	-0.4531509719943140	6132
**ZNF74**	0.32527	-1.7382	-0.45301209305772200	6133
**COPRS**	0.32536	-0.27855	-0.45276213298636600	6134
**SPATA13**	0.3254	-0.56233	-0.4526510487019580	6135
**ORC2**	0.32544	-1.1158	-0.4525399700028970	6136
**ABHD5**	0.32546	-0.56448	-0.4524844327472690	6137
**APOLD1**	0.32552	-1.9156	-0.45231782935309200	6138
**ASRGL1**	0.32582	-2.4633	-0.45148500056188200	6143
**DNAH10**	0.32582	-0.81095	-0.45148500056188200	6139
**PPIL3**	0.32582	-0.91905	-0.45148500056188200	6140

**SKOR1**	0.32582	-2.2503	-0.451485000561882000	6141
**TMCC1**	0.32582	-2.4335	-0.451485000561882000	6142
**ZNF397**	0.32582	-2.7263	-0.451485000561882000	6144
**CSNK2A1**	0.32592	-2.8091	-0.451207460568586	6146
**HOOK3**	0.32592	-3.205	-0.451207460568586	6149
**MGAT4B**	0.32592	-3.0538	-0.451207460568586	6148
**TLR9**	0.32592	-2.8667	-0.451207460568586	6147
**USP2**	0.32592	-2.5192	-0.451207460568586	6145
**NPIPB5**	0.32597	-1.6785	-0.451068703605713000	6151
**PSMC3IP**	0.67403	-0.089388	-0.451068703605713000	6150
**KIF20A**	0.3261	-1.0362	-0.4507079761347810	6152
**PLCD1**	0.32613	-1.3725	-0.450624739663628000	6154
**PPFIBP1**	0.32613	-0.1937	-0.450624739663628000	6153
**SATL1**	0.67379	-0.44326	-0.4504027910013360	6155
**WDR3**	0.32626	-2.6559	-0.450264084354369000	6156
**MARCH2**	0.32629	-0.018624	-0.4501808645243790	6157
**DPY30**	0.32642	-1.788	-0.4498202812768930	6158
**RNF207**	0.32643	-1.0759	-0.4497925465269610	6159
**PREX2**	0.32646	-2.3182	-0.4497093443529610	6160
**ASAP1**	0.6734	-0.17978	-0.449321108689537000	6161
**DNAJB13**	0.3266	-1.8256	-0.449321108689537000	6162
**NXNL1**	0.32673	-0.95528	-0.4489606647778890	6163
**ARGFX**	0.32688	-0.77506	-0.4485448404210680	6164
**B3GNT3**	0.32707	-0.92544	-0.4480182408748990	6165
**CHRNA1**	0.32707	-2.9732	-0.4480182408748990	6166
**AP3B1**	0.32708	-1.5312	-0.447990528551828000	6170
**HOXB6**	0.32708	-0.58405	-0.447990528551828000	6168
**LOC283710**	0.32708	-1.0781	-0.447990528551828000	6169
**OR51S1**	0.32708	-1.7413	-0.447990528551828000	6171
**PPP4R2**	0.32708	-2.195	-0.447990528551828000	6173
**USP41**	0.32708	-1.8627	-0.447990528551828000	6172
**WDR37**	0.32708	-0.40922	-0.447990528551828000	6167

**MYOZ1**	0.32711	-2.656	-0.44790739364675300	6174
**PYGB**	0.32716	-1.06	-0.44776884235029900	6175
**RAB29**	0.32719	-0.61559	-0.44768571569831700	6176
**GBP5**	0.3274	-1.6973	-0.4471039157055570	6177
**IQGAP2**	0.67248	-0.27423	-0.44677152651625700	6178
**NME2**	0.32753	-0.59241	-0.4467438296456200	6179
**C1R**	0.32756	-1.8318	-0.44666074108980700	6180
**CYP1B1**	0.67232	-0.5215	-0.44632841769376900	6181
**HSPB6**	0.32769	-1.1075	-0.44630072630281300	6182
**NT5C2**	0.32777	-0.069775	-0.44607920749285900	6183
**PSG7**	0.32815	-0.94264	-0.44502729178573700	6184
**PSAPL1**	0.32825	-3.1581	-0.44475055373649800	6185
**CPXM1**	0.32849	-0.19586	-0.4440865213138420	6186
**CRYZ**	0.32863	-1.1193	-0.4436992594959760	6187
**TMEM80**	0.32869	-1.2315	-0.44353331051693400	6188
**DHX8**	0.6713	-0.41975	-0.44350565354137300	6189
**AKAP17A**	0.32872	-1.579	-0.4434503406079420	6191
**IL34**	0.32872	-1.8778	-0.4434503406079420	6193
**LY9**	0.32872	-2.7598	-0.4434503406079420	6198
**MRGPRX4**	0.32872	-2.3587	-0.4434503406079420	6197
**PDE3A**	0.32872	-2.2026	-0.4434503406079420	6195
**RPH3A**	0.32872	-0.13374	-0.4434503406079420	6190
**RUNX1**	0.32872	-2.2788	-0.4434503406079420	6196
**WRAP53**	0.32872	-2.1972	-0.4434503406079420	6194
**ZNF692**	0.32872	-1.6929	-0.4434503406079420	6192
**PSMD6**	0.32907	-1.1266	-0.4424825836824920	6200
**UGT1A9**	0.32907	-0.84927	-0.4424825836824920	6199
**TRIM43B**	0.32927	-1.127	-0.44192976578867700	6201
**KYNU**	0.32938	-1.2909	-0.44162577351654600	6202
**DOC2A**	0.67058	-0.027373	-0.4415152409913850	6203
**RBMS2**	0.32947	-0.24541	-0.44137708291997400	6204
**NOP56**	0.32951	-3.2401	-0.44126656252858900	6207

**SOX6**	0.32951	-3.0382	-0.441266562528589OO	6206
**ZNF395**	0.32951	-2.3103	-0.441266562528589OO	6205
**CXCL8**	0.32957	-1.3488	-0.441100792046688OO	6209
**ZIM2**	0.67043	-0.48732	-0.4411007920466880	6208
**L1TD1**	0.32965	-0.80127	-0.440879783590638OO	6210
**ATP11C**	0.67033	-0.31148	-0.440824534841698	6211
**SBF1**	0.3297	-0.9375	-0.4407416642411410	6212
**LGALS16**	0.67028	-0.14247	-0.4406864188556630	6213
**GMEB2**	0.32979	-0.96675	-0.440493070596634OO	6214
**TAS2R20**	0.67008	-0.28046	-0.4401340389359050	6215
**ALKBH7**	0.66975	-0.009991	-0.4392229054914590	6216
**ELF5**	0.33032	-0.18005	-0.439029681653626OO	6217
**MTO1**	0.33032	-0.49753	-0.439029681653626OO	6218
**SCN10A**	0.33032	-2.1641	-0.439029681653626OO	6220
**TGFBR1**	0.33032	-0.69453	-0.439029681653626OO	6219
**GATC**	0.33051	-0.89426	-0.4385052995416390	6222
**SLC8B1**	0.66949	-0.19633	-0.438505299541639OO	6221
**EBF3**	0.33054	-1.2555	-0.4384225133911580	6223
**NPHP3**	0.33072	-0.68764	-0.437925859558686OO	6224
**SFR1**	0.33072	-2.3794	-0.437925859558686OO	6225
**MON1B**	0.33076	-0.69192	-0.437815506712759	6226
**PAGE2B**	0.33077	-1.4292	-0.4377879193343790	6227
**RAMP1**	0.33077	-2.7392	-0.4377879193343790	6228
**DSCC1**	0.66916	-0.36732	-0.4375948170132100	6229
**GMFG**	0.33087	-1.3053	-0.437512063870839OO	6230
**SCFD1**	0.33097	-2.7283	-0.437236241696542OO	6231
**TMEM265**	0.66902	-0.63729	-0.437208661308904OO	6232
**MYDGF**	0.33114	-0.82291	-0.4367674203169390	6233
**ZNF697**	0.66885	-0.056537	-0.4367398455791030	6234
**TDP2**	0.33116	-1.4518	-0.4367122711733490	6235
**PHF5A**	0.33119	-0.22513	-0.436629549948279OO	6236
**TMEM140**	0.33121	-0.72858	-0.436574404124770OO	6237

**CREB3L1**	0.33125	-0.31207	-0.4364641164604030	6238
**ZNF90**	0.33128	-2.2157	-0.43638140419603500	6239
**CD99L2**	0.33134	-0.93946	-0.4362159886225310	6240
**CCDC170**	0.33136	-1.5391	-0.4361608527505830	6241
**ZNF627**	0.3314	-1.05	-0.4360505849841280	6242
**SCGB3A1**	0.33143	-0.91848	-0.43596788763863800	6243
**PRSS57**	0.33152	-2.2277	-0.4357198134876430	6244
**GLDC**	0.66839	-0.44575	-0.4354717661484800	6245
**IL33**	0.33162	-1.1032	-0.4354442069871540	6246
**GCDH**	0.66831	-0.21047	-0.4352513021164570	6247
**IL11**	0.3317	-2.4108	-0.43522374560009400	6251
**MAST3**	0.3317	-0.70788	-0.43522374560009400	6248
**NFKB2**	0.3317	-1.0517	-0.43522374560009400	6249
**RXRB**	0.3317	-2.0642	-0.43522374560009400	6250
**CDH18**	0.66824	-0.4242	-0.43505841344121100	6252
**ARL8A**	0.33178	-1.1512	-0.43500330536443200	6253
**TRIM46**	0.33178	-1.589	-0.43500330536443200	6254
**OR2A5**	0.33189	-0.78528	-0.43470023454953300	6255
**XRRA1**	0.33197	-1.0438	-0.43447984448926500	6256
**DYRK3**	0.33211	-1.1045	-0.43409421265062200	6257
**OLIG2**	0.66785	-0.34433	-0.4339840439838840	6258
**CSNK1D**	0.33219	-1.6205	-0.43387388058425400	6259
**TAF7**	0.33222	-1.1858	-0.43379126148996400	6260
**C1QB**	0.3323	-0.79491	-0.43357095837877400	6261
**KCNG1**	0.33237	-1.6593	-0.43337821041704700	6262
**NUAK2**	0.33247	-0.98784	-0.433102884111339	6263
**ZNF492**	0.66746	-0.4055	-0.43291017523141000	6264
**TTC28**	0.66743	-0.18334	-0.43282759063312700	6265
**RANBP17**	0.3326	-0.59467	-0.4327450089867350	6266
**GUCY2F**	0.33285	-0.35367	-0.43205694329230600	6267
**COX16**	0.66712	-0.49621	-0.4319743891596030	6268
**OMP**	0.33289	-3.0314	-0.43194687176960700	6270

**VCL**	0.33289	-0.41473	-0.43194687176960700	6269
**FAM43B**	0.6671	-0.4626	-0.431919354706682	6271
**ABCG1**	0.33324	-2.6351	-0.43098396897060400	6279
**CCNG1**	0.33324	-0.76644	-0.43098396897060400	6273
**CDC42EP5**	0.33324	-2.8732	-0.43098396897060400	6282
**DEAF1**	0.33324	-2.5975	-0.43098396897060400	6278
**FBXO42**	0.33324	-1.0152	-0.43098396897060400	6275
**FRAT1**	0.33324	-2.3801	-0.43098396897060400	6277
**GFRA3**	0.33324	-2.8282	-0.43098396897060400	6281
**HHLA1**	0.33324	-2.7063	-0.43098396897060400	6280
**LHFPL3**	0.33324	-0.83528	-0.43098396897060400	6274
**RBMX**	0.33324	-2.8937	-0.43098396897060400	6283
**SLC22A23**	0.33324	-0.61709	-0.43098396897060400	6272
**SYNJ1**	0.33324	-2.0159	-0.43098396897060400	6276
**TMEM145**	0.33336	-1.1429	-0.43065392288194400	6284
**HNRNPU**	0.66662	-0.33278	-0.43059891976203700	6285
**CBSL**	0.33341	-0.83374	-0.4305164175246450	6286
**CHST14**	0.33341	-0.99344	-0.4305164175246450	6287
**CIR1**	0.33343	-1.0514	-0.4304614176610290	6288
**GH1**	0.33347	-0.064977	-0.4303514218399220	6289
**NEU3**	0.66642	-0.28812	-0.4300489601731610	6290
**CDH23**	0.66627	-0.10252	-0.42963657583326000	6291
**VASH1**	0.33385	-1.1837	-0.42930672096332800	6292
**ZDHHC21**	0.33393	-2.0943	-0.42908684366645700	6293
**TEX101**	0.33396	-0.59006	-0.4290043950286720	6294
**HNRNPF**	0.33404	-1.3251	-0.4287845462494410	6295
**EML5**	0.33406	-0.044106	-0.42872958729314500	6296
**TCP11**	0.33409	-1.6625	-0.42864715128666100	6297
**LY6E**	0.3341	-2.1717	-0.4286196732651760	6299
**MIA3**	0.6659	-0.024421	-0.4286196732651760	6298
**DOHH**	0.33412	-2.8944	-0.42856471819305000	6302
**DUSP27**	0.33412	-0.97951	-0.42856471819305000	6300

**ELANE**	0.33412	-2.1745	-0.42856471819305000	6301
**FAS**	0.66586	-0.32286	-0.42850976441519300	6303
**KCNK15**	0.33418	-0.96631	-0.42839986074138	6304
**DOCK3**	0.33428	-1.2137	-0.428125124190683	6305
**BLZF1**	0.33451	-2.223	-0.4274933526991790	6306
**WFDC8**	0.66546	-0.24709	-0.4274109603054250	6307
**SMLR1**	0.3347	-2.7264	-0.426971583188194	6308
**BTBD19**	0.33477	-2.4023	-0.4267793816212280	6314
**MRGPRE**	0.33477	-1.1627	-0.4267793816212280	6309
**MRPL38**	0.33477	-1.9314	-0.4267793816212280	6311
**NHLH1**	0.33477	-2.1492	-0.4267793816212280	6312
**NUDT16L1**	0.33477	-1.8534	-0.4267793816212280	6310
**PTRHD1**	0.33477	-2.8482	-0.4267793816212280	6316
**RPL29**	0.33477	-2.8251	-0.4267793816212280	6315
**TTK**	0.33477	-2.3805	-0.4267793816212280	6313
**SGSM3**	0.33479	-0.49928	-0.4267244697837830	6317
**KIF15**	0.33483	-1.1263	-0.4266146499687210	6318
**NEURL4**	0.33499	-0.11273	-0.42617542213974500	6319
**ZNRF1**	0.33502	-0.82492	-0.4260930760768430	6320
**CDT1**	0.3353	-0.47477	-0.425324652044906	6321
**SPCS2**	0.66469	-0.65448	-0.42529721297654400	6322
**GTPBP1**	0.66466	-0.19245	-0.4252148976925790	6323
**CCR2**	0.33539	-1.9552	-0.42507771195468900	6324
**PPDPF**	0.33539	-2.6503	-0.42507771195468900	6325
**SRY**	0.33539	-2.9448	-0.42507771195468900	6326
**EPHX4**	0.33546	-0.29111	-0.42488566536004600	6327
**ILK**	0.33548	-0.72337	-0.4248307977829100	6328
**CCDC6**	0.33554	-0.97047	-0.42466620272407200	6329
**ASZ1**	0.66437	-0.011947	-0.4244193317041620	6330
**STRA8**	0.33563	-0.99795	-0.4244193317041620	6331
**FNDC3A**	0.33572	-0.19063	-0.4241724865480770	6333
**IFNA4**	0.33572	-0.060035	-0.4241724865480770	6332

**MED11**	0.33572	-0.5635	-0.4241724865480770	6334
**MAP3K21**	0.66423	-0.3579	-0.4240353615184490	6335
**MRPL23**	0.66419	-0.12859	-0.4239256672353610(	6336
**OR1B1**	0.66417	-0.26285	-0.4238708220067390	6337
**SKA3**	0.33584	-0.33073	-0.423843399870559	6338
**ITGAM**	0.33586	-0.47111	-0.423788556554325	6339
**TEX29**	0.33592	-0.4731	-0.4236240342525980	6340
**ZNF557**	0.33596	-1.5778	-0.4235143590883510	6341
**PPP2CA**	0.33605	-1.6825	-0.4232676085913670(	6342
**RGR**	0.33606	-1.5831	-0.4232401934604990	6343
**SMCR8**	0.33611	-1.9255	-0.4231031225771190(	6344
**SLC29A3**	0.33616	-1.1995	-0.4229660596427730(	6345
**CRYGD**	0.33619	-2.0013	-0.4228838256962450(	6350
**LOC283357**	0.33619	-0.60691	-0.4228838256962450(	6346
**NENF**	0.33619	-0.95247	-0.4228838256962450(	6347
**NR1D1**	0.33619	-1.4425	-0.4228838256962450(	6348
**RFX1**	0.33619	-2.0178	-0.4228838256962450(	6351
**SMIM18**	0.33619	-2.1975	-0.4228838256962450(	6352
**SNX19**	0.33619	-1.9292	-0.4228838256962450(	6349
**UBXN8**	0.33655	-0.74624	-0.4218972411739020	6353
**ADAMTS9**	0.33656	-2.4107	-0.42186984191385	6354
**LCMT1**	0.33658	-1.9173	-0.421815044343832	6355
**PIAS2**	0.33661	-0.73514	-0.4217328503635990(	6356
**VPS51**	0.33663	-1.0739	-0.4216780559596480(	6357
**ZNF223**	0.66325	-0.58273	-0.4213493161149000	6358
**SPNS2**	0.66323	-0.21669	-0.4212945305685210(	6359
**TMEM200C**	0.33685	-1.8054	-0.4210754010255380(	6360
**INTS14**	0.3369	-2.5204	-0.4209384553287070	6361
**PLA2R1**	0.33693	-1.7168	-0.4208562916999160(	6362
**ZNF132**	0.66303	-0.41735	-0.4207467446141620	6363
**OR11L1**	0.33699	-0.96237	-0.4206919729647710(	6364
**TMEM65**	0.33708	-1.8166	-0.4204455161568820(	6365

**LPAR5**	0.33711	-0.7062	-0.4203633695632050(	6366
**C1QTNF7**	0.33713	-0.3916	-0.4203086067433320(	6367
**ENOX1**	0.33716	-1.6318	-0.4202264648768350(	6369
**SHROOM1**	0.66284	-0.39627	-0.4202264648768350(	6368
**LRRC37B**	0.66279	-0.078465	-0.4200895680663220(	6370
**PCK2**	0.33721	-0.61864	-0.4200895680663220(	6371
**CEP162**	0.33728	-0.44678	-0.4198979257564340(	6373
**REPS2**	0.33728	-0.03646	-0.4198979257564340(	6372
**CYP46A1**	0.33741	-0.18717	-0.4195420595134270	6374
**DAPL1**	0.33749	-0.10179	-0.4193230913108280(	6375
**GABPA**	0.33756	-2.8906	-0.4191315106252700(	6377
**MICALCL**	0.33756	-1.0847	-0.4191315106252700(	6376
**MAP2K7**	0.66232	-0.16421	-0.4188031223750450	6378
**ACMSD**	0.33779	-2.582	-0.4185021394829560	6384
**CYP21A2**	0.33779	-1.6329	-0.4185021394829560	6381
**DNAJB1**	0.33779	-2.7537	-0.4185021394829560	6386
**EMP3**	0.33779	-2.3508	-0.4185021394829560	6382
**KIR2DL4**	0.33779	-1.4966	-0.4185021394829560	6380
**MCMDC2**	0.33779	-0.77632	-0.4185021394829560	6379
**NT5DC4**	0.33779	-2.5533	-0.4185021394829560	6383
**RGS14**	0.33779	-2.6823	-0.4185021394829560	6385
**RNLS**	0.66219	-0.43582	-0.4184474193945820(	6387
**QTRT1**	0.33789	-0.92084	-0.4182285515681880(	6388
**TXNDC9**	0.33798	-0.77474	-0.4179823492081840(	6389
**PNMA2**	0.33805	-1.1098	-0.4177908759994940(	6390
**HRC**	0.33807	-1.2257	-0.4177361721819820(	6391
**ENPP4**	0.66189	-0.44409	-0.4176267682969100	6392
**PPP1R14B**	0.33821	-1.3745	-0.4173532804486520	6393
**CDKL4**	0.33823	-0.21833	-0.417298586625421	6394
**PRDM9**	0.66167	-0.25134	-0.4170251362292400	6395
**SELENOP**	0.33844	-1.6289	-0.4167243768049830	6396
**SPDYC**	0.33857	-2.7799	-0.41636898243931	6397

**CCDC66**	0.6614	-0.18317	-0.4162869758244040	6398
**ARAP3**	0.33877	-0.68307	-0.4158223245306530	6399
**PIGN**	0.33877	-0.9235	-0.4158223245306530	6400
**PMM2**	0.33877	-2.131	-0.4158223245306530	6401
**ADA**	0.33882	-0.87804	-0.4156856794737520	6402
**RAVER1**	0.66116	-0.20418	-0.4156310236243290	6403
**CDK5R1**	0.33885	-0.077455	-0.4156036961652240	6404
**H3F3C**	0.66113	-0.16561	-0.4155490421780860	6405
**VPS13B**	0.66102	-0.63983	-0.4152484674302600	6406
**ZNRF2**	0.33906	-0.019863	-0.4150298911765260	6407
**CSF2**	0.33918	-1.3131	-0.4147020639664410	6410
**EIF2B1**	0.33918	-0.49451	-0.4147020639664410	6409
**FAM189A1**	0.33918	-1.9783	-0.4147020639664410	6415
**GDPD1**	0.33918	-1.936	-0.4147020639664410	6413
**HOXB3**	0.33918	-1.7232	-0.4147020639664410	6411
**MTMR14**	0.33918	-2.3827	-0.4147020639664410	6416
**PFKFB2**	0.33918	-1.9712	-0.4147020639664410	6414
**TMED3**	0.33918	-0.26235	-0.4147020639664410	6408
**TRIM37**	0.33918	-1.7507	-0.4147020639664410	6412
**ZNF628**	0.33918	-2.8388	-0.4147020639664410	6417
**MTERF2**	0.3392	-0.52767	-0.4146474304318390	6418
**ZNF318**	0.33923	-1.6463	-0.4145654824504230	6419
**ELK1**	0.33952	-1.0993	-0.4137734620467780	6420
**SEMA6D**	0.66045	-0.2157	-0.4136915437224630	6421
**GPN1**	0.33956	-2.2768	-0.4136642382312930	6422
**PCDH9**	0.33957	-1.7437	-0.4136369330485470	6423
**WDHD1**	0.33966	-1.2136	-0.4133912002783140	6424
**MBD3L4**	0.66025	-0.23977	-0.4131454924681550	6425
**GPR137**	0.66013	-0.47721	-0.4128179208469950	6426
**SMIM10L1**	0.33994	-3.0573	-0.4126268578581450	6427
**FAAH**	0.66002	-0.1835	-0.4125176857704460	6428
**C20orf78**	0.34007	-2.683	-0.4122720665461190	6430

**NAV1**	0.34007	-0.65101	-0.41227206654611900	6429
**TNFSF12-TN**	0.34008	-0.62344	-0.4122447770567120	6431
**EMC1**	0.65977	-0.13054	-0.4118354715408060	6432
**VGLL4**	0.34027	-0.64282	-0.41172633505765100	6433
**FAM131B**	0.34037	-1.4267	-0.41145351530002200	6434
**PPP6C**	0.65952	-0.29824	-0.4111534489333970	6435
**EBI3**	0.3405	-1.7328	-0.4110988953901590	6436
**TCEA2**	0.34052	-0.97295	-0.41104434307036700	6437
**TUSC1**	0.65937	-0.19307	-0.41074432717091700	6438
**FSD2**	0.65936	-0.7188	-0.41071705483183500	6439
**FRS2**	0.34072	-0.40665	-0.41049888711424200	6440
**C15orf59**	0.34073	-2.1932	-0.4104716175235190	6443
**FOXO1**	0.34073	-1.5527	-0.4104716175235190	6442
**PSMD4**	0.34073	-2.5008	-0.4104716175235190	6444
**RIMBP3**	0.34073	-2.63	-0.4104716175235190	6446
**TMEM173**	0.34073	-2.5755	-0.4104716175235190	6445
**ZIC1**	0.34073	-1.0806	-0.4104716175235190	6441
**LOC730098**	0.65911	-0.34946	-0.41003534556582800	6447
**SPRYD4**	0.65899	-0.000808	-0.40970819281070600	6448
**DNAJC16**	0.34111	-0.46224	-0.4094355990094670	6449
**CDR1**	0.34125	-0.59785	-0.40905401878872700	6450
**SYCE1L**	0.65856	-0.15954	-0.4085362551543890	6451
**PRAMEF12**	0.65841	-0.24764	-0.40812757174550700	6452
**MKI67**	0.34162	-0.93786	-0.40804584324528400	6453
**NEK6**	0.34165	-0.71423	-0.40796411747054700	6454
**SPARCL1**	0.65813	-0.66831	-0.40736487833206800	6455
**WDR63**	0.34187	-0.70167	-0.40736487833206800	6456
**ATP7A**	0.65805	-0.60164	-0.40714700946490400	6457
**EIF4EBP1**	0.65804	-0.18342	-0.40711977721553600	6458
**BLOC1S6**	0.65795	-0.59653	-0.4068747005542390	6459
**ANKRD13A**	0.34236	-2.8379	-0.40603073452586200	6462
**EXOC3L4**	0.34236	-3.0337	-0.40603073452586200	6464

**FAM217B**	0.34236	-0.57242	-0.40603073452586200	6461
**SNX17**	0.34236	-2.9013	-0.40603073452586200	6463
**SRPK2**	0.34236	-0.23911	-0.40603073452586200	6460
**SLC22A9**	0.34266	-0.979	-0.40521426848707800	6465
**MSH2**	0.65727	-0.068873	-0.40502379862627200	6466
**CENPF**	0.34276	-1.6616	-0.40494217318422900	6472
**DDA1**	0.34276	-2.4155	-0.40494217318422900	6480
**ENKUR**	0.34276	-0.39308	-0.40494217318422900	6467
**FGF18**	0.34276	-2.3595	-0.40494217318422900	6478
**IFI35**	0.34276	-2.3696	-0.40494217318422900	6479
**NLRP10**	0.34276	-2.1367	-0.40494217318422900	6476
**NMNAT3**	0.34276	-2.0065	-0.40494217318422900	6475
**PLLP**	0.34276	-0.47546	-0.40494217318422900	6468
**PPAT**	0.34276	-1.7219	-0.40494217318422900	6473
**SELE**	0.34276	-0.99914	-0.40494217318422900	6470
**TAF1B**	0.34276	-1.6559	-0.40494217318422900	6471
**TRMT1L**	0.34276	-1.7797	-0.40494217318422900	6474
**TSTD2**	0.34276	-0.79015	-0.40494217318422900	6469
**ZNF75D**	0.34276	-2.3282	-0.40494217318422900	6477
**ANO3**	0.65721	-0.13085	-0.4048605504401260	6481
**PRRT1**	0.65709	-0.29631	-0.40453408642865600	6482
**BORCS8-MI**	0.34328	-0.043811	-0.4035277602370930	6483
**PGLYRP1**	0.3433	-0.056598	-0.4034733758757900	6484
**SCAF8**	0.65658	-0.6764	-0.4031470947601590	6485
**C1orf194**	0.65628	-0.31195	-0.4023315796164290	6486
**DNAH11**	0.34373	-2.7045	-0.40230440038906400	6488
**ZSCAN30**	0.34373	-2.5453	-0.40230440038906400	6487
**RPL13**	0.65617	-0.31489	-0.40203262445618900	6489
**RSBN1L**	0.65605	-0.33576	-0.40170653252771700	6490
**FBLN7**	0.34396	-1.0876	-0.4016793601288800	6491
**C9orf43**	0.65593	-0.57613	-0.40138048330971200	6492
**PIWIL4**	0.34412	-1.4067	-0.40124464206349600	6493

**C1QBP**	0.3442	-0.88508	-0.40102731146794800	6494
**NCKIPSD**	0.34425	-2.1748	-0.4008914894645620	6495
**TYK2**	0.34438	-0.19318	-0.40053838685668900	6496
**NAALADL1**	0.34442	-1.1617	-0.40042974994879100	6497
**ZNF513**	0.3445	-0.7621	-0.4002124903083440	6498
**FAM122B**	0.65543	-0.47395	-0.4000224036184460	6499
**EDC3**	0.34459	-1.0765	-0.3999680957906410	6500
**SIPA1L3**	0.3446	-0.66029	-0.39994094231911000	6501
**ART5**	0.34464	-1.1399	-0.3998323313815110	6502
**PHF1**	0.34478	-0.83885	-0.39945223023027500	6503
**FCRL4**	0.65517	-0.070768	-0.39931649380705800	6504
**CYB561D1**	0.34488	-1.092	-0.39918076474063500	6505
**C18orf25**	0.34498	-0.051868	-0.3989093286649840	6506
**ARSK**	0.34505	-2.6079	-0.39871934089923000	6507
**HIP1**	0.34507	-0.76536	-0.39866506132406400	6508
**ASAP3**	0.3451	-2.2955	-0.39858364416354200	6509
**BRPF1**	0.34517	-1.1877	-0.3983936810623590	6514
**DCTN4**	0.34517	-2.5515	-0.3983936810623590	6519
**FAM180B**	0.34517	-2.3637	-0.3983936810623590	6517
**KPNA5**	0.34517	-0.89477	-0.3983936810623590	6512
**PAQR8**	0.34517	-0.36033	-0.3983936810623590	6510
**RPL13A**	0.34517	-1.8146	-0.3983936810623590	6516
**SACS**	0.34517	-1.0063	-0.3983936810623590	6513
**SLC5A1**	0.34517	-1.456	-0.3983936810623590	6515
**SRCIN1**	0.65483	-0.64092	-0.3983936810623590	6511
**SUPT3H**	0.34517	-2.4007	-0.3983936810623590	6518
**CHD3**	0.34534	-2.7754	-0.3979324019330800	6520
**CACNB4**	0.34536	-0.59762	-0.39787813936836100	6521
**ASB2**	0.34554	-0.70399	-0.39738982897828700	6522
**GAD2**	0.34556	-0.18249	-0.3973355781191350	6523
**CENPM**	0.34557	-0.54625	-0.39730845312809800	6524
**AK6**	0.34566	-0.94333	-0.3970643413601860	6525

**C1orf35**	0.65396	-0.035214	-0.39603390783689500	6526
**CCDC60**	0.34615	-0.81211	-0.39573570296131100	6527
**APBB1**	0.3462	-0.57518	-0.39560016692483600	6528
**PHC2**	0.34649	-2.5007	-0.39481420111197400	6529
**CCDC121**	0.34665	-1.3785	-0.3943806692116940	6530
**ZFPL1**	0.65327	-0.44794	-0.3941639310600290	6531
**TEX13A**	0.34676	-0.96368	-0.39408265902714500	6532
**ATXN7L2**	0.34683	-2.0317	-0.39389303440492400	6536
**KLHDC10**	0.34683	-1.7268	-0.39389303440492400	6535
**PBX4**	0.34683	-0.83627	-0.39389303440492400	6534
**PMPCA**	0.34683	-0.82825	-0.39389303440492400	6533
**S100G**	0.34683	-2.8388	-0.39389303440492400	6539
**TMEM223**	0.34683	-2.4015	-0.39389303440492400	6537
**ZNF257**	0.34683	-2.6962	-0.39389303440492400	6538
**AP2A2**	0.65309	-0.34145	-0.3936763378921660	6540
**HNRNPCL1**	0.34696	-1.7023	-0.39354091195920400	6541
**TADA2A**	0.34703	-0.8433	-0.39335132777732400	6542
**DEFB108B**	0.34704	-1.6055	-0.39332424547701100	6543
**PPP2R2C**	0.34719	-2.1563	-0.3929180455756160	6544
**ADAMTS3**	0.34722	-0.73506	-0.39283681337663500	6545
**ABHD15**	0.34738	-1.6295	-0.39240361874313700	6546
**USP8**	0.3474	-0.57215	-0.3923494745929100	6547
**PRAMEF10**	0.34741	-0.92178	-0.3923224029491300	6548
**LCA5L**	0.34744	-1.8163	-0.3922411897428110	6550
**TMEM150A**	0.34744	-0.28727	-0.3922411897428110	6549
**CD3G**	0.65252	-0.053262	-0.39213290949180300	6551
**APOL5**	0.65251	-0.25251	-0.3921058401474650	6552
**LINC00452**	0.34756	-2.5246	-0.3919163627804180	6554
**RNF152**	0.34756	-1.8653	-0.3919163627804180	6553
**TMEM176A**	0.34756	-2.8825	-0.3919163627804180	6555
**ARHGAP12**	0.34763	-1.7835	-0.3917268994828390	6556
**DLX3**	0.65231	-0.18504	-0.39156451356203600	6557

**NAT8**	0.65219	-0.095207	-0.3912397726887080	6558
**SLC25A17**	0.34791	-0.90669	-0.3909691868094530	6559
**OR51M1**	0.348	-1.2084	-0.3907256839911560	6560
**LMO1**	0.34814	-0.001005	-0.3903469478672950	6561
**PRDM10**	0.34821	-1.2173	-0.3901576008036900	6562
**CDH2**	0.34822	-1.0115	-0.3901305523651840	6563
**MIPEP**	0.6517	-0.1868	-0.3899141751302510	6564
**TTC21B**	0.65165	-0.038813	-0.3897789486288480	6565
**CHRNG**	0.34841	-1.9053	-0.3896166862348250	6566
**AGGF1**	0.34844	-0.42818	-0.3895355588847780	6568
**C7orf76**	0.34844	-1.4366	-0.3895355588847780	6571
**CXorf56**	0.34844	-2.3367	-0.3895355588847780	6576
**HSD17B8**	0.34844	-0.82588	-0.3895355588847780	6569
**ITK**	0.34844	-1.9737	-0.3895355588847780	6575
**KRTAP2-3**	0.34844	-1.8008	-0.3895355588847780	6574
**OSMR**	0.34844	-2.7242	-0.3895355588847780	6577
**SLC1A4**	0.34844	-1.497	-0.3895355588847780	6572
**TCTN2**	0.34844	-1.327	-0.3895355588847780	6570
**TULP4**	0.34844	-0.10752	-0.3895355588847780	6567
**ZNHIT1**	0.34844	-1.5173	-0.3895355588847780	6573
**PAK2**	0.34861	-1.1081	-0.3890758856397830	6578
**MUC6**	0.34872	-1.1077	-0.3887784938191590	6579
**PLEKHN1**	0.34876	-2.487	-0.3886703598648140	6580
**DBX2**	0.34878	-0.8234	-0.3886162945919440	6581
**ZNF780A**	0.6511	-0.18141	-0.3882919268019820	6582
**OPA3**	0.34891	-1.9004	-0.3882648979973070	6583
**ATXN8**	0.34897	-1.0714	-0.3881027311249520	6584
**TCEAL2**	0.65091	-0.46501	-0.3877784279919670	6585
**SLC5A6**	0.65077	-0.56397	-0.3874001258733980	6586
**RBM47**	0.34928	-0.096997	-0.3872650314117950	6587
**SUV39H1**	0.65055	-0.22717	-0.3868057630876340	6588
**LDLRAD3**	0.34951	-1.6877	-0.3866436878675500	6589

**NDUFS5**	0.6504	-0.30847	-0.3864005940780120	6590
**PAPSS1**	0.34969	-1.536	-0.3861575231205970	6591
**CELF4**	0.34983	-1.9104	-0.3857794580808320	6592
**IL11RA**	0.34994	-2.2743	-0.3854824456590440	6593
**OR11A1**	0.65005	-0.096242	-0.3854554462163550	6594
**NRBF2**	0.65003	-0.34693	-0.3854014481739020	6595
**ASCL2**	0.34999	-0.89609	-0.3853474512551790	6597
**FATE1**	0.34999	-2.6392	-0.3853474512551790	6600
**TMEM79**	0.34999	-2.2585	-0.3853474512551790	6599
**TP53RK**	0.34999	-1.2067	-0.3853474512551790	6598
**UQCRQ**	0.34999	-0.57828	-0.3853474512551790	6596
**CRMP1**	0.35008	-2.8304	-0.3851044790216030	6601
**CDH26**	0.3502	-0.87737	-0.3847805514024040	6602
**NR3C1**	0.64972	-0.1496	-0.384564622086647	6603
**CALCR**	0.3504	-2.5412	-0.3842407617259550	6604
**HIST1H3F**	0.64948	-0.096641	-0.3839169416616780	6605
**CSMD1**	0.35063	-0.97457	-0.3836201419644820	6606
**APH1A**	0.35065	-2.2144	-0.3835661820144300	6607
**AMIGO3**	0.64931	-0.54422	-0.383458265464524	6608
**GOLM1**	0.64929	-0.48373	-0.3834043088642640	6609
**RPS15**	0.35096	-0.71198	-0.3827299454739200	6610
**PSMD9**	0.35103	-0.91148	-0.3825411549239410	6611
**SOX10**	0.35113	-0.83654	-0.3822714777861200	6612
**DMTF1**	0.35117	-0.38127	-0.382163614715907	6613
**FBXL4**	0.64875	-0.65736	-0.3819479019121320	6614
**OTOA**	0.3513	-0.25396	-0.3818130904351320	6615
**LMX1A**	0.64863	-0.087613	-0.3816243660237840	6616
**CLVS1**	0.3514	-0.28586	-0.3815434882943260	6618
**ERI3**	0.3514	-0.29139	-0.3815434882943260	6619
**GRAMD1A**	0.3514	-2.5346	-0.3815434882943260	6622
**VAV2**	0.3514	-0.092227	-0.3815434882943260	6617
**VWA5B1**	0.3514	-1.43	-0.3815434882943260	6621

**WASL**	0.3514	-0.43554	-0.38154348829432600	6620
**GABRD**	0.64856	-0.31184	-0.38143565520377500	6623
**GTF3C6**	0.35145	-1.975	-0.38140869762419300	6624
**TFF3**	0.64852	-0.27449	-0.3813278265483780	6625
**NADK**	0.35151	-1.4207	-0.3812469579664290	6626
**TNKS1BP1**	0.35151	-2.2357	-0.3812469579664290	6627
**FLNB**	0.35154	-2.8097	-0.38116609187766	6628
**MCHR1**	0.35158	-0.80851	-0.3810582743030720	6629
**CLDN16**	0.64839	-0.19589	-0.38097741402907300	6630
**GRHL3**	0.35173	-1.2704	-0.38065399783532800	6631
**METTL9**	0.64823	-0.10561	-0.38054620128642800	6632
**CDNF**	0.35178	-2.3181	-0.3805192528401740	6633
**MAOA**	0.35183	-2.1534	-0.38038451475347500	6634
**LYPD5**	0.35195	-0.70585	-0.38006117151301700	6635
**C17orf67**	0.64795	-0.20419	-0.37979174916451200	6636
**INO80C**	0.35206	-1.2429	-0.3797648084461930	6637
**FRMD8**	0.64793	-0.33484	-0.37973786800350500	6639
**GAGE12J**	0.35207	-0.04347	-0.37973786800350500	6638
**USP14**	0.64791	-0.029369	-0.3796839879449260	6640
**SENP5**	0.64785	-0.008031	-0.37952235438173900	6641
**ZAR 1.00**	0.35218	-0.64615	-0.37944154131845900	6642
**OR10X1**	0.35219	-0.087587	-0.3794146041813920	6643
**NPY5R**	0.35223	-1.5491	-0.37930685838592100	6644
**ZSCAN21**	0.64757	-0.52468	-0.37876819542532500	6645
**LRRC66**	0.35263	-0.75109	-0.3782296423451450	6646
**KIF4A**	0.64732	-0.46847	-0.3780950212203590	6647
**RAB33B**	0.35269	-0.60469	-0.378068097817721	6649
**SMYD2**	0.64731	-0.36514	-0.37806809781772100	6648
**NTRK3**	0.35286	-0.25824	-0.37761044188174800	6650
**CATSPERZ**	0.35291	-2.6322	-0.37747585224940100	6651
**PKHD1L1**	0.35299	-1.5024	-0.3772605230581470	6652
**UMODL1**	0.64697	-0.17675	-0.377152865022421	6653

**NARF**	0.35311	-2.4818	-0.3769375620627560	6654
**UNCX**	0.35321	-0.33049	-0.37666845793125300	6655
**ADAM28**	0.35333	-2.0428	-0.3763455689736050	6667
**ATP6V0B**	0.35333	-0.88264	-0.3763455689736050	6656
**CXorf21**	0.35333	-1.9732	-0.3763455689736050	6666
**DDAH1**	0.35333	-1.5503	-0.3763455689736050	6661
**GHRH**	0.35333	-1.3353	-0.3763455689736050	6658
**IQSEC2**	0.35333	-1.7558	-0.3763455689736050	6663
**IRF3**	0.35333	-1.8797	-0.3763455689736050	6665
**KIF23**	0.35333	-2.3944	-0.3763455689736050	6670
**KRT33A**	0.35333	-1.3427	-0.3763455689736050	6659
**LRRTM1**	0.35333	-1.7102	-0.3763455689736050	6662
**MUC20**	0.35333	-2.5737	-0.3763455689736050	6671
**PEX11B**	0.35333	-2.2999	-0.3763455689736050	6669
**POLRMT**	0.35333	-1.5278	-0.3763455689736050	6660
**PRMT3**	0.35333	-2.7253	-0.3763455689736050	6673
**SMO**	0.35333	-1.8461	-0.3763455689736050	6664
**SSH3**	0.35333	-0.99164	-0.3763455689736050	6657
**ZNF473**	0.35333	-2.6137	-0.3763455689736050	6672
**ZNF641**	0.35333	-2.1814	-0.3763455689736050	6668
**AP4E1**	0.35336	-0.29999	-0.37626485286589400	6674
**PDP1**	0.64663	-0.03392	-0.37623794804143000	6675
**FGFR3**	0.35347	-1.3996	-0.3759689147716500	6676
**GRIFIN**	0.35354	-0.82363	-0.37578060767395600	6677
**CDH19**	0.64641	-0.17351	-0.3756461107622890	6678
**KRT5**	0.35367	-0.12141	-0.3754309298356980	6679
**FAM49B**	0.35373	-1.199	-0.37526955554848700	6680
**DERA**	0.35378	-0.12382	-0.37513508444081800	6681
**RSPRY1**	0.6462	-0.12026	-0.3750812978971770	6682
**TPCN1**	0.64616	-0.21474	-0.37497372806495800	6683
**ZC3H12B**	0.35384	-2.4607	-0.37497372806495800	6684
**CFAP58**	0.35395	-0.97767	-0.37467793339327200	6686

**HOXC10**	0.35395	-0.76941	-0.3746779333932720	6685
**SPATA19**	0.64598	-0.25475	-0.3744897174890620	6687
**C2CD2L**	0.35403	-0.97326	-0.3744628305858420	6688
**ZWINT**	0.3543	-0.30594	-0.3737369864416380	6689
**SIGLEC14**	0.64569	-0.28935	-0.3737101071085890	6690
**ABHD3**	0.3544	-1.0324	-0.373468205258296	6692
**ARMC1**	0.3544	-0.83027	-0.373468205258296	6691
**C12orf56**	0.3544	-2.2349	-0.373468205258296	6693
**GJD2**	0.3544	-2.3644	-0.373468205258296	6694
**EIF4EBP3**	0.35448	-1.9272	-0.3732531997369220	6695
**FSCN2**	0.64536	-0.39009	-0.3728232404409580	6696
**DAZL**	0.64511	-0.63205	-0.3721515669595930	6697
**RIC8A**	0.64492	-0.26113	-0.3716412073943740	6698
**RANBP2**	0.35511	-0.6748	-0.3715606331565920	6699
**COMP**	0.35529	-2.7043	-0.3710772383623330	6702
**SIAH2**	0.35529	-0.32461	-0.3710772383623330	6700
**USP17L7**	0.35529	-2.0032	-0.3710772383623330	6701
**FHL1**	0.64465	-0.29406	-0.3709161260367800	6703
**PSKH1**	0.35536	-0.99185	-0.3708892749186600	6704
**CDC42EP4**	0.3555	-2.7199	-0.3705133873310100	6705
**TBC1D30**	0.35561	-1.428	-0.3702180838072980	6706
**IER3IP1**	0.35586	-2.0729	-0.3695470594370800	6708
**TMEM54**	0.35586	-1.9919	-0.3695470594370800	6707
**TTC39C**	0.35586	-2.3526	-0.3695470594370800	6709
**STK25**	0.64405	-0.39464	-0.3693055314079050	6710
**TTC30B**	0.64402	-0.54168	-0.3692250268528070	6711
**SEPT10**	0.64399	-0.24931	-0.3691445246905810	6712
**RNASEK**	0.64396	-0.23744	-0.3690640249205650	6713
**ZNF683**	0.35606	-1.9124	-0.3690103597358990	6714
**NACAD**	0.35616	-0.53444	-0.3687420497493190	6715
**GJB4**	0.6437	-0.24678	-0.3683664603552450	6716
**TEX46**	0.3563	-0.68873	-0.3683664603552450	6717

**L3HYPDH**	0.64365	-0.4646	-0.36823233388113900	6718
**TRAP1**	0.35648	-0.41644	-0.3678836360418190	6719
**KIAA0922**	0.6432	-0.50113	-0.36702549333299300	6720
**SLC11A2**	0.64318	-0.51374	-0.36697186839336400	6721
**TMPRSS3**	0.64316	-0.39083	-0.3669182445089920	6722
**BRI3BP**	0.35688	-1.0107	-0.3668109999052370	6723
**ARHGAP45**	0.64298	-0.2593	-0.36643567700392400	6724
**AEBP2**	0.35703	-0.52398	-0.3664088702009920	6727
**BTBD8**	0.35703	-2.1801	-0.3664088702009920	6730
**EPHB2**	0.35703	-0.3438	-0.3664088702009920	6726
**HABP4**	0.35703	-1.7315	-0.3664088702009920	6728
**MED12L**	0.35703	-2.3482	-0.3664088702009920	6731
**RAB14**	0.35703	-1.9099	-0.3664088702009920	6729
**S100Z**	0.35703	-0.024085	-0.3664088702009920	6725
**TEX38**	0.35703	-2.9021	-0.3664088702009920	6732
**ZNF695**	0.35711	-0.15644	-0.36619442525431200	6733
**CAPZA1**	0.64274	-0.15967	-0.3657923863551260	6734
**C2orf91**	0.35728	-2.7562	-0.3657387856369310	6735
**SCCPDH**	0.35733	-0.1306	-0.3656047884380960	6736
**CKM**	0.35742	-0.7013	-0.3653636100199480	6737
**MFN1**	0.64245	-0.6084	-0.36501527870497400	6738
**MEOX1**	0.64225	-0.87758	-0.3644794708260740	6739
**SMIM5**	0.64214	-0.18001	-0.36418482109857400	6740
**KCTD3**	0.6421	-0.26784	-0.3640776835831500	6741
**PTPN5**	0.35812	-0.71767	-0.3634885019041890	6742
**MAP2K6**	0.35818	-2.1645	-0.3633278378968790	6743
**ELP3**	0.35826	-1.0504	-0.36311363380757400	6744
**PHTF1**	0.35832	-0.82943	-0.3629529916741330	6745
**EP300**	0.64166	-0.45063	-0.3628994463778640	6746
**HOXA3**	0.64149	-0.62072	-0.3624443533481090	6748
**SLC7A10**	0.64149	-0.26985	-0.3624443533481090	6747
**MYLK3**	0.64145	-0.29325	-0.3623372835470430	6749

**C20orf96**	0.35868	-1.0968	-0.3619893353717200(	6751
**ZNF233**	0.35868	-0.061451	-0.3619893353717200(	6750
**MAGED4B**	0.35906	-1.8982	-0.3609725071454660(	6752
**C16orf74**	0.35916	-2.7558	-0.3607049828331080	6753
**UTP20**	0.35924	-1.2612	-0.3604909819696640	6754
**ADI1**	0.35925	-1.7445	-0.3604642330226980(	6756
**DYRK4**	0.35925	-0.64709	-0.3604642330226980(	6755
**GOLGA7B**	0.35925	-2.701	-0.3604642330226980(	6759
**GRB2**	0.35925	-2.7381	-0.3604642330226980(	6760
**MAJIN**	0.35925	-2.0073	-0.3604642330226980(	6757
**TP73**	0.35925	-2.2804	-0.3604642330226980(	6758
**XRN2**	0.35936	-0.86563	-0.360170011622956	6761
**OAZ1**	0.64062	-0.54489	-0.3601165201733500	6762
**ZNF805**	0.35952	-0.4579	-0.3597421088663450	6763
**SMCO2**	0.35954	-0.91272	-0.3596886256544940(	6764
**NUDCD1**	0.35973	-0.53864	-0.3591805864287380(	6765
**VPS35**	0.35987	-1.3611	-0.3588063010559880	6766
**BCL2L14**	0.35996	-1.0417	-0.3585657155742200(	6767
**TMEM242**	0.36005	-0.15046	-0.3583251508449270(	6768
**PHKG1**	0.36006	-0.47816	-0.3582984227108180(	6769
**KRTAP10-3**	0.63992	-0.8422	-0.3582449672104660	6770
**SWSAP1**	0.36016	-1.2866	-0.3580311554437520(	6771
**UNC93B1**	0.36021	-1.2767	-0.3578975314013460(	6772
**ATRX**	0.36023	-0.82024	-0.3578440835737770	6773
**KLK4**	0.36028	-0.097702	-0.3577104684766580(	6774
**FBLN5**	0.36029	-0.54785	-0.3576837462236370	6775
**OR8K1**	0.63962	-0.10254	-0.3574432574371020(	6776
**IFIH1**	0.36045	-0.8495	-0.3572562248950100	6777
**EPB42**	0.36047	-0.33695	-0.3572027893214570	6779
**MS4A18**	0.36047	-0.010682	-0.3572027893214570	6778
**CCDC47**	0.36066	-1.4533	-0.3566952022142770(	6780
**IFNA2**	0.36071	-0.94199	-0.3565616419380150	6781

**PCDHGC5**	0.36071	-2.4587	-0.3565616419380150	6782
**ATF7IP2**	0.36082	-1.9432	-0.3562678317138030	6785
**BMPR2**	0.63918	-0.27028	-0.3562678317138030(	6783
**EIF1AD**	0.36082	-2.77	-0.3562678317138030	6786
**KRTAP20-2**	0.36082	-1.7055	-0.3562678317138030	6784
**OR13C4**	0.36085	-0.37467	-0.3561877069910080(	6787
**HSPB9**	0.36089	-1.033	-0.3560808775841760	6788
**VPS13C**	0.6391	-0.48838	-0.3560541708674700(	6789
**CYP2C9**	0.36091	-1.0416	-0.3560274644047150(	6790
**FDPS**	0.36098	-1.395	-0.3558405262740980(	6791
**SH3BGR**	0.63884	-0.07420(	-0.3553598853006000	6792
**BBS1**	0.36117	-2.6016	-0.3553331854322330(	6797
**CCL24**	0.36117	-2.3894	-0.3553331854322330(	6796
**FAM217A**	0.36117	-0.86373	-0.3553331854322330(	6795
**SERPINB13**	0.36117	-2.6882	-0.3553331854322330(	6798
**SIRPD**	0.36117	-0.52544	-0.3553331854322330(	6793
**SPATA5**	0.36117	-0.80368	-0.3553331854322330(	6794
**KRTAP12-3**	0.36118	-1.1348	-0.3553064858171760	6799
**SLC16A2**	0.36127	-1.5084	-0.3550662006765940(	6800
**SSRP1**	0.36131	-2.2499	-0.3549594138616310	6801
**HSH2D**	0.36152	-0.09259(	-0.3543988494535	6802
**HHLA3**	0.36155	-1.9338	-0.3543187779179980	6803
**ZNF654**	0.63841	-0.73768	-0.3542120194041960	6804
**HPS5**	0.6384	-0.22986	-0.3541853304065760	6805
**CYP2B6**	0.36174	-0.82757	-0.3538117109189350	6806
**SNUPN**	0.3618	-1.1061	-0.3536516034022750	6807
**MMACHC**	0.36182	-0.77377	-0.3535982362447800	6808
**HAUS8**	0.36198	-0.92096	-0.3531713352225350	6810
**SERPINA3**	0.63802	-0.21707	-0.3531713352225350	6809
**OR4F6**	0.36201	-0.58989	-0.3530912984481740(	6811
**PCDHB3**	0.36203	-0.99404	-0.3530379418551930(	6812
**LNX2**	0.36217	-1.0913	-0.3526644738351660(	6813

**KCNK17**	0.63776	-0.67308	-0.3524777582726570(	6814
**RCC2**	0.36234	-2.3839	-0.3522110430678220(	6816
**SLC1A1**	0.36234	-2.7828	-0.3522110430678220(	6818
**VAX1**	0.36234	-0.6016	-0.3522110430678220(	6815
**WDFY4**	0.36234	-2.4285	-0.3522110430678220(	6817
**LMAN1**	0.36246	-0.90498	-0.3518910178896870(	6820
**OLA1**	0.63754	-0.014903	-0.3518910178896870(	6819
**FOXL2**	0.63752	-0.20312	-0.3518376838645230(	6821
**SGCE**	0.3626	-0.99234	-0.3515177007234690(	6822
**CD74**	0.3627	-0.16457	-0.3512510755981140(	6823
**FBL**	0.3627	-0.4173	-0.3512510755981140(	6824
**HEATR3**	0.3627	-2.8214	-0.3512510755981140(	6829
**MRPS34**	0.3627	-2.2252	-0.3512510755981140(	6826
**TAC4**	0.3627	-2.2429	-0.3512510755981140(	6827
**TESK2**	0.3627	-1.8825	-0.3512510755981140(	6825
**TNFRSF10C**	0.3627	-2.4798	-0.3512510755981140(	6828
**DRD1**	0.36273	-1.4275	-0.3511710929302820(	6831
**RAB24**	0.36273	-0.95492	-0.3511710929302820(	6830
**SOSTDC1**	0.36281	-1.2888	-0.350957816796994	6832
**ABHD14B**	0.36303	-0.79942	-0.3503713897017200(	6833
**ZNF398**	0.36311	-1.171	-0.3501581733662120	6834
**SPATA25**	0.63687	-0.13098	-0.3501048717698990	6835
**MCF2L**	0.36328	-0.044647	-0.3497051414889450	6836
**NABP1**	0.36332	-0.094115	-0.3495985561863690(	6837
**MEOX2**	0.3634	-0.43833	-0.3493853974940070	6838
**BAG4**	0.36349	-1.0385	-0.3491456129385040	6839
**APCDD1L**	0.63645	-0.79807	-0.3489857677208770(	6840
**LOC285500**	0.36362	-0.66046	-0.3487992929024270(	6841
**SPATA31A1**	0.36369	-1.0678	-0.3486128302119070	6842
**C18orf32**	0.36375	-0.62745	-0.3484530146953370	6843
**DAAM2**	0.36375	-1.9718	-0.3484530146953370	6844
**PACSIN3**	0.63624	-0.33715	-0.3484263796412580	6845

**CRIP3**	0.36378	-2.1477	-0.3483731102746150	6846
**PTP4A2**	0.36381	-1.9061	-0.3482932080780880	6847
**STRIP2**	0.36384	-0.4741	-0.3482133081051240	6848
**PPP2R1A**	0.63593	-0.37948	-0.3476008154495760	6849
**LARP1B**	0.36422	-0.37346	-0.3472014339744890	6850
**C11orf57**	0.36429	-0.29398	-0.3470150749073710	6851
**PKIB**	0.36429	-1.541	-0.3470150749073710	6852
**SPANXN5**	0.36429	-1.9869	-0.3470150749073710	6853
**TRIM16L**	0.6357	-0.27852	-0.3469884531674320	6854
**HTR3E**	0.36441	-1.1063	-0.3466956302532630	6855
**SH2D3A**	0.36441	-1.9627	-0.3466956302532630	6856
**MRPL55**	0.36444	-2.3758	-0.3466157746185820	6857
**SLC25A13**	0.63554	-0.005722	-0.3465625387567630	6858
**MYT1**	0.36452	-0.86545	-0.3464028370635010	6859
**SPSB3**	0.36464	-2.4601	-0.3460834601750140	6860
**AIMP1**	0.36481	-2.0992	-0.3456310699950990	6861
**LMO4**	0.63518	-0.19216	-0.345604461011659	6862
**MICAL2**	0.63518	-0.8106	-0.345604461011659	6863
**KLHL30**	0.3649	-1.0379	-0.3453915979513090	6864
**CHSY3**	0.365	-2.1857	-0.3451255411304990	6865
**RAN**	0.63498	-0.17636	-0.3450723326984210	6866
**CDR2L**	0.36509	-0.23938	-0.3448861108782170	6867
**GLUD1**	0.36514	-0.21129	-0.3447531026148740	6868
**CAPRIN2**	0.63479	-0.38731	-0.3445669012915200	6869
**ARRDC5**	0.63475	-0.72493	-0.3444605058991830	6870
**LDAH**	0.36529	-0.65283	-0.3443541144059760	6871
**MLF 1.00**	0.36538	-0.069223	-0.3441147477947380	6872
**CTC1**	0.36543	-0.62833	-0.3439817748638930	6873
**TMSB10**	0.36572	-0.98202	-0.3432106517087150	6874
**LPL**	0.36587	-0.34931	-0.3428118749989990	6875
**C3orf22**	0.36591	-0.67799	-0.3427055437519810	6876
**MBNL3**	0.36593	-0.79882	-0.3426523795815350	6877

**EDRF1**	0.36604	-0.17496	-0.34235999395035600	6878
**PRKD1**	0.36607	-1.2382	-0.34228025749494400	6879
**OR2K2**	0.36608	-0.70432	-0.34225367916008600	6880
**TRMT11**	0.36608	-1.2961	-0.34225367916008600	6881
**C2orf74**	0.36616	-1.0656	-0.342041061182908	6882
**SLC4A5**	0.36622	-1.0753	-0.34188160784712700	6883
**ZNF814**	0.36634	-0.7584	-0.3415627272464370	6884
**CCDC181**	0.36636	-0.1511	-0.3415095838570520	6885
**HPN**	0.36636	-2.0451	-0.3415095838570520	6889
**IDI1**	0.36636	-0.17671	-0.3415095838570520	6886
**MAGEF1**	0.36636	-0.33815	-0.3415095838570520	6887
**NAA11**	0.36636	-2.81	-0.3415095838570520	6890
**XKR3**	0.36636	-0.79579	-0.3415095838570520	6888
**ASTN2**	0.36646	-0.60613	-0.34124388137357900	6891
**B4GALNT1**	0.36654	-0.37849	-0.34103133673183900	6893
**BIRC5**	0.36654	-0.22736	-0.34103133673183900	6892
**H2AFV**	0.36679	-0.41999	-0.3403672339623570	6894
**NFKBIZ**	0.36692	-1.7588	-0.34002195985603800	6895
**SNTN**	0.36694	-1.2493	-0.33996884436141	6896
**GTSF1L**	0.36712	-0.68187	-0.3394908480479170	6897
**GFRA4**	0.36715	-2.3097	-0.33941118953928000	6898
**MYH8**	0.63266	-0.39194	-0.3389067356383070	6899
**WNT3**	0.36734	-1.1116	-0.3389067356383070	6900
**MITD1**	0.63246	-0.59385	-0.3383758246836490	6901
**NCAPG2**	0.36758	-0.6502	-0.338269653941707	6902
**ACAP1**	0.36768	-2.357	-0.3380042437650620	6903
**TBC1D16**	0.36771	-0.6054	-0.33792462535560300	6904
**OR6F1**	0.36784	-1.2035	-0.3375796369927620	6905
**FAM186A**	0.36793	-0.69868	-0.3373408224316380	6906
**ANKRD18A**	0.36825	-2.1588	-0.3364918596784990	6911
**FRRS1L**	0.36825	-0.72797	-0.3364918596784990	6908
**PRPF38A**	0.36825	-1.5503	-0.3364918596784990	6909

**SNAI2**	0.36825	-0.48282	-0.3364918596784990	6907
**TEX37**	0.36825	-1.7147	-0.3364918596784990	6910
**SYT9**	0.36839	-1.2749	-0.3361205147579900	6912
**SERPINA9**	0.36843	-1.4029	-0.3360144247241820(	6913
**PRPF6**	0.63156	-0.36871	-0.3359879028066810(	6914
**THAP5**	0.36852	-0.57367	-0.3357757359727970(	6915
**USP35**	0.36858	-0.32626	-0.3356166207663960(	6916
**AP5B1**	0.6314	-0.69916	-0.3355635842526220	6917
**IQCG**	0.36867	-0.11438	-0.3353779638862360	6918
**CARD16**	0.36882	-0.6713	-0.334980244856699	6919
**DOK4**	0.63117	-0.56762	-0.3349537321393140(	6920
**COX18**	0.36887	-0.75367	-0.3348476836239830	6921
**C20orf194**	0.3689	-2.5309	-0.3347681497087650	6922
**MRPL27**	0.36898	-0.15851	-0.334556069619008	6923
**BAMBI**	0.36899	-1.0382	-0.3345295606659850	6924
**CTDP1**	0.63092	-0.16106	-0.3342909906645840(	6925
**ECHDC2**	0.63091	-0.16037	-0.3342644840613220(	6926
**LMO3**	0.36921	-1.0768	-0.3339464231340320(	6928
**NAE1**	0.36921	-0.58828	-0.3339464231340320(	6927
**ITIH2**	0.63078	-0.067501	-0.3339199195820540	6929
**CTAGE9**	0.36923	-1.5154	-0.333893416264631	6930
**UBE2G1**	0.36939	-0.58727	-0.3334693950667250(	6931
**SMIM6**	0.63043	-0.6253	-0.3329924428697990(	6932
**AP4B1**	0.36962	-2.2985	-0.3328599695952100(	6933
**ZNF572**	0.36968	-0.92942	-0.3327010093757350(	6934
**IER5L**	0.36969	-2.1562	-0.3326745168232360(	6938
**LIMS2**	0.36969	-1.7479	-0.3326745168232360(	6936
**MON2**	0.36969	-1.8505	-0.3326745168232360(	6937
**PPP2R1B**	0.36969	-1.4741	-0.3326745168232360(	6935
**TJP2**	0.36969	-2.3465	-0.3326745168232360(	6939
**MINDY2**	0.36971	-0.22141	-0.3326215324186710	6940
**BBOX1**	0.36981	-1.9772	-0.3323566243986460(	6941

**NKX3-2**	0.36985	-2.5307	-0.3322506677218850	6942
**ABCA10**	0.63014	-0.72887	-0.3322241791355590(	6943
**APOC4**	0.63012	-0.37219	-0.3321712026621830	6944
**BPY2B**	0.63009	-0.36046	-0.3320917396999710	6945
**DUS3L**	0.36994	-0.17792	-0.3320122788346380(	6947
**HAUS4**	0.36994	-0.042497	-0.3320122788346380(	6946
**FBRS**	0.63003	-0.16536	-0.3319328200655740	6948
**MBLAC1**	0.37019	-0.08649	-0.3313501864201690	6949
**GAS8**	0.62973	-0.57575	-0.3311383475349430	6950
**RUSC2**	0.3703	-0.94821	-0.3310589117844240(	6951
**AP2S1**	0.37033	-0.67816	-0.3309794781228320	6954
**CAPN1**	0.37033	-0.43732	-0.3309794781228320	6953
**CEP128**	0.37033	-2.0393	-0.3309794781228320	6957
**CYP8B1**	0.37033	-2.2571	-0.3309794781228320	6960
**DNASE1L3**	0.37033	-2.2797	-0.3309794781228320	6961
**FUT7**	0.37033	-2.1775	-0.3309794781228320	6958
**GPANK1**	0.37033	-1.1959	-0.3309794781228320	6956
**HSPE1**	0.37033	-2.2314	-0.3309794781228320	6959
**KIF5C**	0.37033	-1.0994	-0.3309794781228320	6955
**MEIS1**	0.37033	-0.35185	-0.3309794781228320	6952
**RBM33**	0.37043	-1.103	-0.3307147143296980	6962
**RPL17**	0.37045	-1.8725	-0.3306617643535000	6963
**ADRB1**	0.62954	-0.1926	-0.3306352897130580(	6964
**FAM218A**	0.37052	-1.4899	-0.3304764467361720	6965
**STRN3**	0.62942	-0.6033	-0.3303176120970740	6966
**POM121C**	0.37081	-0.61517	-0.329708823132955	6968
**ZNF879**	0.37081	-0.46126	-0.329708823132955	6967
**GPR33**	0.37085	-0.87954	-0.3296029592703990	6969
**ADGRB3**	0.62913	-0.49281	-0.3295500287243710(	6970
**LRRC1**	0.37091	-0.32107	-0.3294441704019110	6971
**PEX12**	0.37095	-0.8961	-0.3293383157710420(	6972
**SYNPR**	0.37098	-1.1033	-0.3292589272196300(	6973

**THUMPD3**	0.3712	-1.0721	-0.328676807875834	6974
**RPS27**	0.62873	-0.383	-0.3284916114482490	6975
**C2orf69**	0.3713	-0.93689	-0.3284122449998600	6976
**C8orf31**	0.37131	-1.4535	-0.3283857899767860	6977
**CMTM8**	0.37136	-2.3342	-0.3282535183083080	6978
**NUDT22**	0.3714	-0.004982	-0.3281477051084530(	6979
**TRAPPC4**	0.3714	-2.5011	-0.3281477051084530(	6980
**FZD10**	0.37142	-0.3055	-0.3280947998863390(	6981
**ACSL3**	0.37147	-0.91708	-0.3279625408482330(	6982
**ILDR1**	0.62833	-1.034	-0.3274335620334960(	6983
**MED19**	0.37167	-0.96899	-0.3274335620334950(	6984
**SPDYE2B**	0.37173	-0.11606	-0.3272748862603150(	6985
**KIRREL2**	0.62821	-0.31393	-0.3271162187267730	6986
**AARS**	0.37188	-0.88031	-0.3268782328651710	6987
**SETD2**	0.3719	-0.61715	-0.3268253496326880(	6988
**PLEKHG6**	0.37204	-0.83867	-0.3264551925869440	6989
**EHD1**	0.62793	-0.058782	-0.3263758790407680(	6990
**KCTD9**	0.37209	-0.62002	-0.3263230044839340	6991
**RLBP1**	0.37212	-2.1443	-0.3262436943591620	6992
**ROGDI**	0.62778	-0.13037	-0.3259793420934410(	6993
**ARL8B**	0.37224	-0.36232	-0.3259264743743720	6994
**ADPRH**	0.37228	-1.6984	-0.3258207416688760(	7000
**ATOH7**	0.37228	-0.70665	-0.3258207416688760(	6997
**C14orf132**	0.37228	-0.90725	-0.3258207416688760(	6998
**KLRG1**	0.37228	-0.69803	-0.3258207416688760(	6996
**PLCD3**	0.37228	-2.1791	-0.3258207416688760(	7002
**POLR1D**	0.37228	-0.6562	-0.3258207416688760(	6995
**R3HDM4**	0.37228	-2.3846	-0.3258207416688760(	7003
**SLC12A5**	0.37228	-0.93667	-0.3258207416688760(	6999
**USP17L18**	0.37228	-2.066	-0.3258207416688760(	7001
**RNF8**	0.37234	-1.1239	-0.3256621494395490(	7004
**KLHL36**	0.37261	-1.4377	-0.3249485856940290	7005

**TMEM144**	0.62738	-0.58722	-0.3249221605865730	7006
**KRT37**	0.37265	-0.97725	-0.3248428866254230(	7007
**SLC43A3**	0.62728	-0.22335	-0.3246579219869900(	7008
**GOLGA8K**	0.62725	-0.2647	-0.3245786548280300	7009
**MANEA**	0.62723	-0.2712	-0.3245258111883990(	7010
**CA7**	0.37281	-0.56459	-0.3244201266275580(	7011
**XPR1**	0.37299	-0.12102	-0.3239445909223230	7012
**RPS27L**	0.627	-0.53267	-0.3239181744207920(	7013
**NEB**	0.62695	-0.24457	-0.3237860953032380(	7014
**KLHDC9**	0.37312	-1.6279	-0.3236011940268860	7015
**NHSL2**	0.62687	-0.38051	-0.3235747804620910	7016
**GPR107**	0.3732	-2.1663	-0.3233898918281760(	7017
**GFPT1**	0.62677	-0.24428	-0.3233106572265190(	7018
**ZNF675**	0.6267	-0.001299	-0.3231257843816120	7019
**ADAP1**	0.3733	-2.2214	-0.3231257843816110	7020
**HEPHL1**	0.37342	-0.088109	-0.3228088851923420(	7021
**OR5M11**	0.62653	-0.31302	-0.3226768534324710(	7022
**DSG3**	0.37354	-0.2888	-0.3224920184175890	7023
**SPINK4**	0.37367	-1.0738	-0.3221487826125700(	7024
**MAFF**	0.37375	-1.4225	-0.3219375794435900	7025
**SUPV3L1**	0.62612	-0.11736	-0.3215944049150550(	7026
**FAM47E-ST**	0.62598	-0.45715	-0.3212248746952850	7027
**ARMCX6**	0.37406	-1.9764	-0.3211193026907950(	7029
**FARSB**	0.37406	-0.45669	-0.3211193026907950(	7028
**GFOD2**	0.37406	-2.2543	-0.3211193026907950(	7032
**PRR21**	0.37406	-2.0532	-0.3211193026907950(	7030
**VAMP7**	0.37406	-2.1398	-0.3211193026907950(	7031
**LST1**	0.62592	-0.15033	-0.3210665180307120	7033
**APOOL**	0.37409	-2.2368	-0.3210401260361340(	7034
**HERC3**	0.62573	-0.11328	-0.3205651083499860(	7035
**KCNIP2**	0.62559	-0.47775	-0.3201957001561080	7037
**ZNF438**	0.62559	-0.46667	-0.3201957001561080	7036

**KRTAP25-1**	0.62556	-0.28385	-0.3201165469435310	7038
**SPDL1**	0.62549	-0.31954	-0.3199318639124510	7039
**ZP4**	0.62543	-0.62452	-0.3197735728564210	7040
**CS**	0.37461	-1.6248	-0.3196680499370200	7041
**POLD4**	0.62536	-0.1628	-0.319588910083388	7042
**MYEF2**	0.37467	-0.27952	-0.3195097722313090	7043
**SIM1**	0.37468	-1.4657	-0.3194833933919680	7044
**SERPINC1**	0.62524	-0.50156	-0.319272370678418	7045
**ALDH1B1**	0.37479	-1.9056	-0.3191932408265820	7046
**GPR12**	0.37482	-0.76879	-0.3191141129733140	7047
**CMTM2**	0.37492	-2.336	-0.3188503678888770	7048
**ACCSL**	0.37494	-0.95127	-0.3187976215340110	7049
**YTHDF1**	0.37497	-0.59779	-0.318718503664645	7050
**ATF5**	0.62495	-0.54811	-0.3185075324313480	7051
**PATE2**	0.62491	-0.013999	-0.3184020521311480	7052
**CAMK2N2**	0.62483	-0.35035	-0.3181911021565880	7053
**ATF6**	0.62467	-0.2954	-0.3177692446713130	7054
**MBTD1**	0.62467	-0.69944	-0.3177692446713130	7055
**NHEJ1**	0.37537	-0.77109	-0.3176637891384020	7056
**ZBTB7A**	0.37542	-1.0303	-0.3175319746897360	7057
**LEF1**	0.62456	-0.1394	-0.3174792504551410	7058
**GSTA5**	0.37558	-1.8918	-0.3171102055136540	7059
**APTX**	0.62434	-0.000375	-0.3168993420821320	7060
**ATF6B**	0.37569	-1.0924	-0.3168202719283210	7061
**BTBD18**	0.37572	-0.99486	-0.3167412037552280	7064
**C12orf4**	0.37572	-1.8865	-0.3167412037552280	7066
**DACT1**	0.37572	-0.52457	-0.3167412037552280	7063
**EIF5**	0.37572	-2.3234	-0.3167412037552280	7068
**F2RL3**	0.37572	-1.9703	-0.3167412037552280	7067
**MLLT10**	0.37572	-0.22009	-0.3167412037552280	7062
**OR4N2**	0.37572	-1.5996	-0.3167412037552280	7065
**LY96**	0.37575	-1.3362	-0.3166621375622610	7069

**RIMS3**	0.37576	-0.088538	-0.3166357826045300	7070
**TRIM60**	0.62424	-0.11159	-0.3166357826045300	7071
**ATXN1L**	0.62409	-0.54589	-0.31624048461737600	7072
**ITGBL1**	0.37592	-1.5074	-0.31621413317582500	7073
**HBM**	0.62407	-0.58565	-0.31618778195384600	7074
**C10orf62**	0.37595	-0.8148	-0.31613508016852600	7075
**SLC25A22**	0.62399	-0.48243	-0.31597698008006900	7076
**GCK**	0.37605	-1.2497	-0.315871584408927	7077
**ATP2B1**	0.37612	-2.3075	-0.31568715042621500	7078
**KARS**	0.62387	-0.64128	-0.31566080359111600	7079
**HADHB**	0.37615	-1.082	-0.31560811057823700	7080
**AMER1**	0.37619	-1.0582	-0.31550272718114100	7081
**CNPY3**	0.37625	-1.3622	-0.31534465865451800	7082
**RAB17**	0.37639	-0.071956	-0.3149758627232760	7083
**LRRC43**	0.37644	-1.1242	-0.3148441602746350	7084
**ZNF8**	0.37663	-0.82438	-0.3143437407494910	7085
**CDKN3**	0.37665	-2.292	-0.31429106959099000	7086
**DOK6**	0.37668	-0.75588	-0.31421206448799200	7087
**PARP8**	0.37669	-0.89235	-0.31418572988950700	7088
**OGDH**	0.37677	-0.59432	-0.31397506094371900	7089
**OR5C1**	0.37682	-0.13785	-0.31384339992895600	7090
**PARP10**	0.37689	-1.1114	-0.3136590836469930	7091
**BOD1L2**	0.62308	-0.51356	-0.3135800942169080	7093
**TBPL2**	0.62308	-0.29826	-0.3135800942169080	7092
**CBWD1**	0.37696	-1.1502	-0.3134747780200740	7094
**TRAM1L1**	0.62282	-0.31438	-0.31289560104694700	7095
**IGSF21**	0.37722	-1.478	-0.31279030742402100	7096
**PCP4L1**	0.37724	-0.88833	-0.3127376619130230	7097
**SLC25A15**	0.37724	-1.0754	-0.3127376619130230	7098
**NR5A2**	0.37734	-0.58928	-0.31247444735569800	7099
**MMAB**	0.37761	-0.81517	-0.3117638761149590	7100
**TPRG1L**	0.62228	-0.77767	-0.3114744292711430	7101

**SLC7A13**	0.37772	-1.0484	-0.3114744292711420(	7102
**COL25A1**	0.37773	-0.54582	-0.3114481172159820	7103
**BAHD1**	0.3778	-2.4289	-0.311263938865968O(	7106
**DRAM2**	0.3778	-2.4937	-0.311263938865968O(	7107
**GGCT**	0.3778	-1.5625	-0.311263938865968O(	7104
**PDS5B**	0.3778	-2.6239	-0.311263938865968O(	7109
**PKD1L3**	0.3778	-2.5221	-0.311263938865968O(	7108
**POLE3**	0.3778	-1.7034	-0.311263938865968O(	7105
**XAGE1B**	0.37792	-0.56937	-0.3109482291107060	7110
**PARVG**	0.37799	-0.81544	-0.3107640794003860	7111
**USP43**	0.37819	-0.18956	-0.310237995409949O(	7112
**RAD51AP1**	0.37824	-1.4893	-0.310106487832830O(	7113
**DYNC2LI1**	0.37828	-0.095809	-0.310001285632461	7114
**SLC38A10**	0.37839	-2.6675	-0.3097119972672550	7115
**ABL2**	0.37848	-0.91111	-0.309475326064634O(	7116
**OFD1**	0.62145	-0.3527	-0.309291260446871	7117
**GLIPR1**	0.37863	-2.572	-0.309080912570756O(	7120
**OR6C3**	0.37863	-2.4355	-0.309080912570756O(	7119
**RIC1**	0.37863	-1.5054	-0.309080912570756O(	7118
**ZNF727**	0.6213	-0.27583	-0.308896869396979O(	7121
**PCOTH**	0.62126	-0.23145	-0.308791706566893	7122
**TAS2R40**	0.62121	-0.62577	-0.3086602578312420	7123
**H6PD**	0.37893	-0.077119	-0.3082922297342640	7124
**SSTR5**	0.62105	-0.10604	-0.3082396577014880	7125
**CARM1**	0.37898	-0.87009	-0.308160801249570O(	7126
**SLC35G3**	0.62098	-0.15337	-0.3080556622942980	7127
**GFM2**	0.37903	-1.0013	-0.3080293780875900	7128
**WDR82**	0.62096	-0.24037	-0.308003094093682O(	7129
**CRNKL1**	0.3791	-0.86365	-0.307845394597833O(	7130
**TAS2R14**	0.37923	-1.9673	-0.3075037386127910	7131
**ANKFY1**	0.6207	-0.395	-0.307319784874285O(	7132
**LMAN2L**	0.37948	-0.83013	-0.3068468087064210	7133

**NDE1**	0.37949	-1.2776	-0.30682053426619	7134
**MAP4**	0.37951	-0.17105	-0.3067679860211310(	7135
**POLR2F**	0.37985	-0.66546	-0.3058747953144750(	7136
**EEFSEC**	0.3799	-1.7153	-0.3057434643359260(	7138
**MSGN1**	0.3799	-2.7494	-0.3057434643359260(	7142
**PPEF2**	0.3799	-0.26429	-0.3057434643359260(	7137
**RNF170**	0.3799	-1.8248	-0.3057434643359260(	7139
**VMO1**	0.3799	-2.1269	-0.3057434643359260(	7141
**YEATS2**	0.3799	-1.9915	-0.3057434643359260(	7140
**SBDS**	0.37999	-0.96753	-0.3055070818610060(	7143
**EXD2**	0.61993	-0.368	-0.3052969784357470(	7144
**CD81**	0.38014	-0.31302	-0.3051131489933940(	7145
**SPTA1**	0.61976	-0.25703	-0.3048505533872630	7146
**PKD2L1**	0.61966	-0.46901	-0.3045879788005620	7147
**HOXD13**	0.6196	-0.59922	-0.3044304441289370	7148
**CST6**	0.61934	-0.019288	-0.303747881123712	7149
**GSE1**	0.38066	-1.512	-0.303747881123712	7150
**OR4N4**	0.38071	-0.25767	-0.3036166352391900	7151
**APOBEC3C**	0.61928	-0.21202	-0.3035903866899310	7152
**FAM72D**	0.38118	-0.63904	-0.3023831791840950(	7153
**ARHGAP30**	0.38119	-2.4861	-0.3023569404424480	7154
**PRKCB**	0.38134	-0.80424	-0.3019633842848980(	7155
**EBLN1**	0.38148	-0.90505	-0.3015961073972720	7156
**HSD17B3**	0.38148	-1.6069	-0.3015961073972720	7157
**ALS2**	0.38155	-0.87736	-0.3014124842113160(	7159
**GLYR1**	0.38155	-1.7935	-0.3014124842113160(	7162
**HCAR3**	0.38155	-2.4862	-0.3014124842113160(	7164
**KRBA2**	0.38155	-2.1971	-0.3014124842113160(	7163
**RRM1**	0.38155	-1.0722	-0.3014124842113160(	7160
**RTKN**	0.38155	-1.1098	-0.3014124842113160(	7161
**TRIM4**	0.38155	-0.36803	-0.3014124842113160(	7158
**NAA40**	0.38156	-1.0659	-0.3013862531573420(	7165

**NXPH1**	0.38166	-0.72991	-0.3011239540194	7167
**RAB23**	0.61834	-0.2169	-0.3011239540194	7166
**SCAPER**	0.61828	-0.2983	-0.30096658448149800	7168
**SMPD4**	0.38176	-0.43306	-0.3008616755970800	7169
**BIK**	0.38178	-0.84699	-0.30080922239661400	7170
**CRYBB2**	0.38187	-1.0809	-0.3005731932336330	7171
**MSRB2**	0.38209	-0.42189	-0.29999630351368500	7172
**DPY19L2**	0.38216	-1.1063	-0.29981276863791300	7173
**ECHDC3**	0.38231	-0.23116	-0.2994195136141680	7174
**PAK5**	0.38232	-1.1127	-0.29939329825956000	7175
**ZNF91**	0.38234	-1.5336	-0.2993408681675790	7176
**C2orf76**	0.61763	-5.6545E-	-0.2992622245723720	7177
**MNX1**	0.38237	-0.92588	-0.2992622245723720	7178
**UNC5B**	0.38242	-0.89481	-0.29913115602631600	7179
**OR52E2**	0.38253	-1.8868	-0.29884282330869900	7180
**ADGRG1**	0.38254	-1.4867	-0.29881661247571900	7181
**THOP1**	0.38255	-0.81498	-0.29879040184802300	7182
**NUDT13**	0.38261	-0.6992	-0.298633142391633	7183
**ESRRA**	0.61738	-0.4298	-0.2986069332003340	7184
**COPE**	0.38264	-0.61967	-0.2985545154330570	7185
**GAS1**	0.38265	-0.51829	-0.29852830685703800	7186
**ALG1L**	0.61725	-0.22472	-0.29826623237114900	7187
**NXN**	0.38275	-0.28184	-0.2982662323711490	7188
**ATL1**	0.38276	-0.48931	-0.29824002604952400	7189
**ANKIB1**	0.3828	-0.74757	-0.2981352028109880	7190
**VAPA**	0.38283	-0.41676	-0.2980565875318560	7191
**MUC8**	0.61714	-0.25984	-0.29797797409474700	7192
**UQCRB**	0.61704	-0.56288	-0.2977159426005420	7193
**OR1E1**	0.38317	-0.80597	-0.29716574296765100	7194
**CCM2**	0.61667	-0.18565	-0.296746603626803	7195
**CC2D2A**	0.3834	-0.59407	-0.2965632465609700	7197
**MREG**	0.3834	-0.068599	-0.2965632465609700	7196

**CENPL**	0.3835	-0.71239	-0.29630132518805400	7198
**LRP3**	0.38351	-2.0572	-0.29627513416899500	7201
**TAGLN2**	0.38351	-0.9306	-0.29627513416899500	7199
**TMC1**	0.38351	-1.051	-0.29627513416899500	7200
**ZNF431**	0.38351	-2.1696	-0.29627513416899500	7202
**ZNF154**	0.61648	-0.10254	-0.29624894335316700	7203
**CCDC179**	0.61641	-0.58167	-0.29606561333105300	7204
**RPH3AL**	0.38376	-0.96704	-0.2956204246875710	7205
**CEP44**	0.38377	-1.0302	-0.2955942389456470	7206
**CASP14**	0.61608	-0.52323	-0.29520147712677600	7207
**TPI1**	0.384	-1.1897	-0.294992022778862	7208
**HTR3C**	0.38422	-2.4633	-0.29441609001463400	7209
**ZMYND11**	0.38439	-1.7018	-0.2939711179467810	7210
**HPR**	0.38451	-1.5548	-0.2936570550620470	7211
**PKP2**	0.38451	-2.315	-0.2936570550620470	7212
**TMEM130**	0.61544	-0.26548	-0.2935262040765810	7213
**ISG20L2**	0.38458	-2.2667	-0.2934738650896710	7214
**BDKRB2**	0.38464	-1.7292	-0.2933168529517080	7215
**CYP4B1**	0.38465	-0.12783	-0.29329068496514600	7216
**ANGPT2**	0.3847	-0.19224	-0.2931598480443630	7217
**GSTA3**	0.38472	-1.999	-0.2931075146812680	7218
**SLC25A43**	0.38481	-0.19905	-0.29287202447870000	7219
**ARGLU1**	0.61512	-0.61676	-0.2926888766606830	7220
**STK26**	0.38495	-0.81643	-0.29250573865965500	7221
**ARL13B**	0.38496	-1.5865	-0.29247957688904700	7223
**DOCK8**	0.61504	-0.35595	-0.2924795768890470	7222
**SNX18**	0.38497	-1.4073	-0.2924534153186170	7224
**LAX1**	0.38504	-0.78615	-0.2922702899288610	7225
**CAMLG**	0.38506	-2.509	-0.2922179701893460	7226
**HEATR9**	0.61493	-0.42742	-0.2921918106195520	7227
**BRPF3**	0.61488	-0.60769	-0.29206101576939100	7228
**LIPF**	0.61482	-0.54455	-0.2919040685438020	7229

**C14orf80**	0.61473	-0.4418	-0.2916686611849570	7230
**SLC30A1**	0.38528	-1.1383	-0.2916425058096780	7231
**BOLA2B**	0.38534	-0.63812	-0.2914855777469440	7232
**HRASLS**	0.61464	-0.3756	-0.2914332699879850(	7233
**SYTL5**	0.61451	-0.69225	-0.2910932889830080(	7234
**ABHD11**	0.38551	-0.31879	-0.2910409872001950(	7235
**ARMCX4**	0.38568	-1.2217	-0.2905964541722720	7236
**SPANXN2**	0.61426	-0.11991	-0.2904395738760500(	7237
**CCDC130**	0.38584	-2.3777	-0.2901781225971550	7244
**CNIH1**	0.38584	-1.466	-0.2901781225971550	7240
**ILF3**	0.38584	-1.2723	-0.2901781225971550	7239
**KLHL42**	0.38584	-0.38857	-0.2901781225971550	7238
**MAMLD1**	0.38584	-2.2902	-0.2901781225971550	7242
**NCR1**	0.38584	-2.7432	-0.2901781225971550	7245
**PHF23**	0.38584	-2.0651	-0.2901781225971550	7241
**TSSK6**	0.38584	-2.353	-0.2901781225971550	7243
**ABTB2**	0.38598	-1.4145	-0.2898121241227140(	7246
**RUFY2**	0.38599	-0.54648	-0.2897859828605570	7247
**SKAP1**	0.3862	-0.44186	-0.2892370620667560(	7248
**CEP170B**	0.38637	-0.75465	-0.2887927614440190	7249
**RAD1**	0.38645	-1.0263	-0.2885836985274910	7250
**CSNK1A1**	0.61343	-0.29943	-0.2882701277972400	7251
**TOM1**	0.61341	-0.62611	-0.2882178687652580	7252
**VSX1**	0.61339	-0.60731	-0.2881656105203730(	7253
**KCNN2**	0.38665	-1.6995	-0.2880610963912260(	7254
**SP6**	0.38667	-0.68317	-0.2880088405066320	7255
**METTL5**	0.61331	-0.081738	-0.28795658540847	7256
**NPM3**	0.38671	-0.66422	-0.2879043310965730	7257
**EGR1**	0.38674	-1.5161	-0.2878259511026090(	7258
**ZBTB2**	0.38682	-0.46851	-0.2876169464281890	7259
**KCNC3**	0.38705	-0.094583	-0.287016127931709	7260
**TRA2B**	0.38712	-1.655	-0.2868332907009770(	7261

**NCEH1**	0.61268	-0.10357	-0.2863109514205990	7262
**ALPL**	0.38736	-1.7625	-0.2862064929422220	7263
**SLC44A3**	0.38742	-1.1526	-0.2860498110794990	7265
**SLC4A10**	0.38742	-0.77743	-0.2860498110794990	7264
**SAR1A**	0.38746	-0.4691	-0.2859453604056430	7266
**HHIPL1**	0.6125	-0.6071	-0.2858409128512750	7267
**HEXB**	0.38753	-2.0375	-0.2857625792318660	7268
**PCCA**	0.61239	-0.27807	-0.285553698150799	7269
**RACGAP1**	0.61226	-0.12296	-0.2852142929600820	7270
**FAM229A**	0.61223	-0.51769	-0.2851359733522750	7271
**C2orf16**	0.38778	-1.0516	-0.2851098672050190	7272
**ERBB4**	0.61219	-0.092538	-0.2850315499290020	7273
**PHB**	0.38792	-0.056887	-0.2847444015361080	7274
**BBS12**	0.38801	-2.1878	-0.2845094794046040	7275
**OXNAD1**	0.38806	-0.64558	-0.2843789738943380	7276
**SERPINA2**	0.38807	-0.75765	-0.2843528733735480	7277
**ANGPTL2**	0.38811	-2.0194	-0.2842484732272400	7280
**ANKFN1**	0.38811	-2.5781	-0.2842484732272400	7283
**ARL2BP**	0.38811	-0.34781	-0.2842484732272400	7279
**CST1**	0.38811	-2.0245	-0.2842484732272400	7281
**MAP3K8**	0.38811	-2.1086	-0.2842484732272400	7282
**NNT**	0.38811	-0.32129	-0.2842484732272400	7278
**HIST1H2AI**	0.38819	-0.95087	-0.2840396822272260	7284
**RAB15**	0.3882	-0.3198	-0.2840135842230040	7285
**ANKRD50**	0.61174	-0.18148	-0.2838570002591320	7286
**HYDIN**	0.38842	-0.33038	-0.283439477033254	7287
**GPRIN2**	0.61155	-0.53055	-0.2833611969309830	7288
**FUBP3**	0.38848	-1.2143	-0.2832829185650080	7289
**UBXN11**	0.38852	-0.85993	-0.2831785501103170	7290
**GLIS1**	0.61145	-0.44314	-0.2831002757935340	7291
**SLC9A6**	0.38868	-1.0764	-0.2827611071227550	7292
**RPS2**	0.3887	-1.0056	-0.2827089302151000	7293

**LGI2**	0.38878	-1.163	-0.2825002302790970	7294
**CNNM2**	0.61119	-0.19013	-0.2824219709756520	7295
**THOC3**	0.38882	-1.2864	-0.2823958849255580	7296
**ZDHHC14**	0.38884	-0.018464	-0.2823437134018280(	7297
**ST8SIA2**	0.61104	-0.41201	-0.2820307003916270(	7298
**FBP2**	0.61099	-0.29334	-0.2819002864606190	7299
**STAT4**	0.38906	-0.71417	-0.281769877323826	7300
**C3orf14**	0.38908	-2.0854	-0.2817177150109130	7301
**PGAM5**	0.38918	-0.49035	-0.2814569149405390(	7302
**ME1**	0.38936	-0.62536	-0.2809875230376830(	7303
**FGF2**	0.38954	-2.3872	-0.2805181930348430(	7304
**CLIC4**	0.61045	-0.57059	-0.2804921209583610	7305
**ARHGAP25**	0.38962	-0.52367	-0.2803096217603040	7306
**SLA2**	0.61022	-0.43704	-0.2798925157797890	7307
**WWOX**	0.38978	-2.0281	-0.2798925157797890	7308
**SYVN1**	0.38991	-0.28448	-0.2795536530249160(	7309
**CFP**	0.39001	-1.4705	-0.2792930112106700	7310
**AOX1**	0.39006	-1.0661	-0.2791626974192120	7315
**CYP2J2**	0.39006	-1.2616	-0.2791626974192120	7316
**DCAF5**	0.39006	-0.99562	-0.2791626974192120	7314
**HLX**	0.39006	-2.0076	-0.2791626974192120	7318
**MRFAP1L1**	0.39006	-0.44838	-0.2791626974192120	7312
**OR5H1**	0.39006	-1.7464	-0.2791626974192120	7317
**RBM43**	0.39006	-0.56323	-0.2791626974192120	7313
**TMEM185B**	0.39006	-0.44647	-0.2791626974192120	7311
**ST20-MTHF**	0.39028	-0.69762	-0.2785893730179030(	7319
**HGFAC**	0.60971	-0.87118	-0.2785633149948830(	7320
**WASHC2C**	0.3907	-1.4577	-0.2774950986696680(	7321
**GYPE**	0.39072	-0.52436	-0.2774429986648500	7323
**TTBK2**	0.39072	-0.34494	-0.2774429986648500	7322
**TIAM2**	0.39074	-0.96097	-0.2773908994130970	7324
**C1orf106**	0.39083	-0.8451	-0.2771564620950060(	7325

**PKN1**	0.60916	-0.57014	-0.2771304144445880	7326
**MOSPD1**	0.60906	-0.28102	-0.2768699482782170	7327
**POU6F2**	0.39096	-0.35204	-0.2768178572994050	7328
**CXXC4**	0.39105	-0.27326	-0.2765834571873510	7329
**CFHR3**	0.60889	-0.19018	-0.2764271988886030(	7330
**HRASLS5**	0.3912	-1.5243	-0.2761928240934140	7331
**ZWILCH**	0.3913	-2.5343	-0.2759324254460310(	7332
**FBXW2**	0.39138	-0.87085	-0.2757241199991860	7333
**DCAF13**	0.60852	-0.003452	-0.2754637550123690(	7334
**OASL**	0.60847	-0.19366	-0.2753335795221740(	7335
**SRXN1**	0.60832	-0.53271	-0.2749430810339490(	7336
**TPX2**	0.60821	-0.6614	-0.2746567421224170(	7337
**CD163**	0.39189	-1.6835	-0.2743964535610530	7342
**CETN2**	0.39189	-1.5968	-0.2743964535610530	7341
**CLVS2**	0.39189	-2.3679	-0.2743964535610530	7345
**GADD45B**	0.39189	-1.5016	-0.2743964535610530	7340
**LOC728392**	0.39189	-2.7075	-0.2743964535610530	7346
**PADI3**	0.39189	-1.3021	-0.2743964535610530	7339
**SERTAD1**	0.39189	-2.1175	-0.2743964535610530	7344
**SNAPIN**	0.39189	-0.63782	-0.2743964535610530	7338
**USP39**	0.39189	-1.9252	-0.2743964535610530	7343
**KBTBD3**	0.39194	-1.2787	-0.2742663162523550	7347
**TCF7L1**	0.39208	-1.1986	-0.2739019564892290	7348
**LY6G5C**	0.60784	-0.22519	-0.2736937672379090	7349
**GAL3ST4**	0.60776	-0.32063	-0.2734855898482400	7350
**LMTK3**	0.60762	-0.077126	-0.2731213079272210(	7351
**CDRT15**	0.39245	-1.1313	-0.2729391805597990	7352
**FKBPL**	0.39258	-0.90784	-0.2726009680326750	7353
**DPYS**	0.39273	-0.84977	-0.27221076154873	7354
**CYP2W1**	0.39278	-1.0331	-0.2720807019333560(	7355
**SBNO2**	0.39279	-0.70197	-0.2720546905626130(	7356
**DEFB107B**	0.60717	-0.09842	-0.2719506469200740	7357

**SCN11A**	0.39287	-1.2595	-0.2718466062212590(	7358
**ADAM20**	0.60699	-0.58906	-0.2714824869385620	7359
**SLC15A2**	0.39304	-0.71747	-0.2714044660621620	7360
**TMA7**	0.3931	-2.228	-0.2712484292649000	7361
**UROS**	0.39315	-0.67387	-0.2711184036452340	7362
**ASB4**	0.6068	-0.30423	-0.2709883826089860	7363
**PLXNA4**	0.60674	-0.73628	-0.2708323634120500(	7364
**TMCC2**	0.39329	-2.2359	-0.2707543562859050(	7365
**C1orf204**	0.39334	-1.3757	-0.270624348069815	7366
**RBP2**	0.39344	-1.0249	-0.2703643453566380(	7368
**UEVLD**	0.39344	-0.47882	-0.2703643453566380(	7367
**HIPK1**	0.39347	-0.87642	-0.2702863481072670(	7370
**MRPS21**	0.60653	-0.12943	-0.2702863481072670(	7369
**FBXL13**	0.60647	-0.3576	-0.2701303585406570	7371
**OR5AK2**	0.39357	-0.48426	-0.2700263691479360(	7372
**HEATR5A**	0.39359	-2.2389	-0.2699743755465970(	7373
**GSG1L**	0.60639	-0.016321	-0.2699223826750590(	7374
**KLK11**	0.39361	-0.62068	-0.2699223826750590(	7375
**APLP1**	0.39367	-1.8945	-0.2697664084376340	7379
**DHX33**	0.39367	-1.8748	-0.2697664084376340	7378
**FAM216A**	0.39367	-0.92703	-0.2697664084376340	7376
**LBHD1**	0.39367	-0.96054	-0.2697664084376340	7377
**PRAMEF11**	0.39371	-1.0883	-0.2696624292587330(	7380
**ZNF707**	0.60617	-0.32859	-0.2693505092088940(	7381
**RAB5C**	0.3939	-1.3336	-0.2691685679495320	7382
**ZNF503**	0.39392	-0.421	-0.26911658636931	7383
**VIT**	0.60607	-0.78536	-0.2690905958518880(	7384
**ISL1**	0.39399	-2.5685	-0.2689346565637430	7386
**PYCR1**	0.60601	-0.69217	-0.268934656563743	7385
**RCOR3**	0.39405	-2.2464	-0.2687787238148350(	7387
**UHMK1**	0.39415	-1.0598	-0.2685188504198400	7388
**SLC20A2**	0.39417	-0.63887	-0.2684668779173730	7389

**CCT5**	0.60575	-0.91896	-0.26825899515725400	7390
**HEXIM1**	0.60568	-0.42675	-0.26807710725147500	7391
**CLIC2**	0.39437	-1.063	-0.26794719274866200	7393
**PDSS1**	0.39437	-0.23971	-0.26794719274866200	7392
**LILRB5**	0.60556	-0.47851	-0.2677653200412300	7394
**CRYBB1**	0.60546	-0.57479	-0.2675055172491300	7395
**RIPPLY2**	0.39468	-1.3466	-0.2671418236666940	7396
**FXYD6-FXY**	0.3947	-0.50912	-0.2670898703260920	7397
**SNRPE**	0.60523	-0.069981	-0.2669080393098930	7398
**NOP14**	0.39492	-0.77789	-0.26651843112256500	7399
**UBE3B**	0.39499	-1.0101	-0.26633662781569100	7400
**PLRG1**	0.39514	-0.88712	-0.26594707893284600	7401
**IMPA1**	0.39516	-1.0344	-0.26589514213206200	7402
**ERMP1**	0.39531	-0.97481	-0.26550563897596000	7403
**APOB**	0.39543	-2.3852	-0.2651940654524480	7410
**C1orf189**	0.39543	-2.2478	-0.2651940654524480	7408
**KRT15**	0.39543	-2.3401	-0.2651940654524480	7409
**LBP**	0.39543	-1.1858	-0.2651940654524480	7406
**MARCO**	0.39543	-0.13847	-0.2651940654524480	7404
**PAM**	0.39543	-1.4194	-0.2651940654524480	7407
**ZNF783**	0.39543	-0.33307	-0.2651940654524480	7405
**WIPF2**	0.39549	-0.96087	-0.2650382883459520	7411
**ANKRD39**	0.39552	-1.313	-0.2649604022046330	7412
**PAFAH1B1**	0.39555	-1.3682	-0.26488251767054700	7413
**MPP4**	0.60442	-0.32513	-0.2648046347431560	7414
**NEFL**	0.39568	-1.9672	-0.2645450365821760	7415
**DAG1**	0.39569	-0.030118	-0.26451907774685100	7416
**ULK3**	0.39574	-2.3336	-0.26438928624345700	7417
**TOM1L1**	0.39582	-0.2045	-0.2641816291004100	7418
**FDFT1**	0.39595	-1.4915	-0.2638442105331340	7420
**PAXBP1**	0.39595	-0.98561	-0.2638442105331340	7419
**SLC10A4**	0.60389	-0.38136	-0.2634289673700340	7421

**TTLL4**	0.39613	-1.6689	-0.26337706516962	7422
**ADIG**	0.39618	-0.91263	-0.26324731277213700	7423
**NEK4**	0.39632	-1.1747	-0.26288402962766400	7424
**NFATC3**	0.39641	-2.3514	-0.2626505087850300	7425
**KCNMA1**	0.39649	-0.81226	-0.26244294672614100	7426
**TRIM24**	0.39661	-2.2403	-0.262131624832614	7427
**ATP10A**	0.39677	-1.0968	-0.2617165684848210	7428
**ADCK5**	0.60287	-0.34333	-0.2607828564023300	7429
**ARMC4**	0.39718	-1.6481	-0.26065319216608900	7431
**TMPRSS5**	0.39718	-0.54177	-0.26065319216608900	7430
**SLC47A1**	0.60281	-0.40626	-0.2606272598447620	7432
**UBE2C**	0.39722	-1.3045	-0.26054946393227000	7433
**CACNB3**	0.39735	-2.4712	-0.2602123665255740	7438
**GRIA3**	0.39735	-1.9959	-0.2602123665255740	7436
**LDLRAP1**	0.39735	-0.56496	-0.2602123665255740	7434
**MAD1L1**	0.39735	-1.7208	-0.2602123665255740	7435
**THAP9**	0.39735	-2.4894	-0.2602123665255740	7439
**TMEM99**	0.39735	-2.1371	-0.2602123665255740	7437
**COL18A1**	0.39749	-0.6996	-0.25984937161234200	7440
**MND1**	0.39751	-1.2702	-0.2597975179921070	7441
**PALD1**	0.60243	-0.17153	-0.25964196132183100	7442
**REC8**	0.60237	-0.79882	-0.2594864109338760	7443
**VAPB**	0.39772	-1.0339	-0.25925309712195200	7444
**SPTSSA**	0.60224	-0.64008	-0.25914940662480900	7446
**ZNF514**	0.39776	-0.55461	-0.25914940662480900	7445
**RPAP1**	0.39779	-1.2726	-0.2590716405804200	7447
**BTN2A2**	0.39787	-0.57818	-0.25886427211986000	7448
**HSPA5**	0.60201	-0.47599	-0.25855324029578100	7449
**GIT2**	0.60199	-0.05714	-0.2585014040908640	7450
**MED25**	0.39818	-0.53601	-0.2580608243759170	7451
**TMCO4**	0.39822	-0.56167	-0.25795716584300100	7452
**SORCS1**	0.60164	-0.61185	-0.25759438279589800	7453

**PVR**	0.60162	-0.15808	-0.25754255941476200	7454
**TTPAL**	0.3984	-0.48946	-0.25749073672526500	7455
**C1orf74**	0.6013	-0.72611	-0.25671347927237900	7456
**ANKMY2**	0.6012	-0.3107	-0.25645442793693700	7457
**NCK2**	0.39886	-0.45393	-0.2562990053969130	7458
**DNAAF4**	0.60105	-0.50609	-0.25606588319318200	7459
**CCDC173**	0.60101	-0.56364	-0.25596227779219700	7460
**GHRL**	0.60076	-0.93799	-0.2553148062308210	7461
**ADAM2**	0.39936	-1.6804	-0.2550040579107100	7470
**ATP2C1**	0.39936	-0.33021	-0.2550040579107100	7464
**C14orf119**	0.39936	-0.42105	-0.2550040579107100	7465
**C5orf63**	0.39936	-2.4418	-0.2550040579107100	7474
**CYB5A**	0.39936	-1.7919	-0.2550040579107100	7471
**GTSE1**	0.39936	-0.13734	-0.2550040579107100	7463
**HTATIP2**	0.39936	-0.55258	-0.2550040579107100	7466
**MOK**	0.39936	-1.5824	-0.2550040579107100	7469
**OR1A1**	0.39936	-0.000815	-0.2550040579107100	7462
**RNF31**	0.39936	-1.5559	-0.2550040579107100	7468
**TMEM229A**	0.39936	-1.4837	-0.2550040579107100	7467
**ZNF263**	0.39936	-2.0663	-0.2550040579107100	7472
**ZNF609**	0.39936	-2.11	-0.2550040579107100	7473
**MPP1**	0.39939	-0.014455	-0.2549263746791180	7475
**WNT7A**	0.60051	-0.038453	-0.25466744168088800	7476
**FBXO43**	0.3997	-2.72	-0.2541237379330740	7477
**RPL18**	0.39976	-1.1215	-0.253968407808663	7478
**CDK10**	0.39992	-0.54372	-0.25355422408701400	7479
**CTNNBIP1**	0.39993	-1.0273	-0.2535283390492660	7480
**MUC5B**	0.59998	-0.9339	-0.2532953813511460	7481
**TUBA1B**	0.40004	-1.8712	-0.2532436148408730	7482
**C17orf77**	0.40007	-0.86982	-0.25316596634779100	7483
**CLYBL**	0.40013	-0.87237	-0.2530106739400730	7484
**SLC12A3**	0.59978	-0.5969	-0.25277746766668900	7485

**ZNF274**	0.59964	-0.31987	-0.2524154428598710	7486
**GNGT1**	0.59948	-0.70894	-0.2520014218148740	7487
**ZFPM1**	0.40053	-0.61837	-0.2519755469344450	7488
**EFCAB9**	0.40057	-0.81073	-0.2518720490994600	7489
**INSL4**	0.4008	-0.51345	-0.2512769888647270	7490
**AKAP9**	0.40086	-0.3762	-0.2511217704000520	7491
**NPEPL1**	0.59908	-0.42463	-0.2509665579851330	7492
**TRIM31**	0.40095	-1.4649	-0.2508889540450170	7493
**PTMS**	0.599	-0.29472	-0.2507596175020280	7494
**CHL1**	0.59893	-0.65714	-0.2505785533878850	7495
**OR4C13**	0.40111	-0.80547	-0.2504750918683020	7496
**ZNF784**	0.40114	-1.0801	-0.2503974974880800	7497
**EXOC4**	0.40125	-1.0523	-0.2501129976543430	7498
**GJB1**	0.5987	-0.51588	-0.2499836862412170	7499
**MRPL10**	0.40136	-1.0662	-0.2498285180625770	7500
**RTL8A**	0.59859	-0.33845	-0.2496992158417830	7501
**FCRLB**	0.40152	-0.59262	-0.2494147656466500	7502
**NSFL1C**	0.40154	-1.6728	-0.2493630495980400	7503
**ANGPTL3**	0.40162	-2.1118	-0.2491561920710410	7511
**BSG**	0.40162	-1.7298	-0.2491561920710410	7506
**CCDC169-S**	0.40162	-2.1662	-0.2491561920710410	7512
**CREG2**	0.40162	-2.0052	-0.2491561920710410	7510
**LIG3**	0.40162	-0.26635	-0.2491561920710410	7504
**MYH11**	0.40162	-1.9517	-0.2491561920710410	7508
**PDE10A**	0.40162	-1.561	-0.2491561920710410	7505
**RGS5**	0.40162	-1.9925	-0.2491561920710410	7509
**SLC26A4**	0.40162	-2.365	-0.2491561920710410	7513
**TGFA**	0.40162	-1.7665	-0.2491561920710410	7507
**ZNF880**	0.40162	-2.4451	-0.2491561920710410	7514
**GNG11**	0.40165	-1.2967	-0.2490786232473460	7515
**SLURP1**	0.40173	-2.2752	-0.2488717803758870	7516
**RAD23A**	0.40175	-1.0266	-0.2488200713219670	7517

**RAD50**	0.59812	-0.034987	-0.2484839786804350	7518
**GJB7**	0.40197	-0.67051	-0.24825131560230800	7519
**TMEM143**	0.40213	-0.7283	-0.2478377255211880	7520
**ADD3**	0.59782	-0.61856	-0.24770848731748500	7521
**FBXW7**	0.40231	-0.47765	-0.24737248734324100	7522
**ZMYM3**	0.59762	-0.70288	-0.2471915758490250	7523
**SPAG1**	0.4024	-1.6624	-0.2471398883368210	7524
**FES**	0.40247	-1.2741	-0.24695898724235300	7525
**STK11IP**	0.5975	-0.27332	-0.24688146067606000	7526
**REG4**	0.59744	-0.4535	-0.24672641199423500	7527
**RDH13**	0.59739	-0.43624	-0.24659720929007600	7528
**ASB16**	0.59731	-0.43952	-0.24639049352418600	7529
**DENND4A**	0.40284	-0.83739	-0.24600292983067000	7530
**SKA1**	0.59704	-0.73181	-0.24569290548011600	7531
**AKR1C1**	0.40306	-0.96467	-0.24543456989304700	7532
**IRS2**	0.4031	-0.63477	-0.2453312402451220	7533
**RBPJ**	0.40316	-1.8344	-0.24517625068390900	7534
**TEX9**	0.59667	-0.16078	-0.24473714556105900	7535
**PPP1R1C**	0.40335	-1.3647	-0.2446854892385770	7537
**QRSL1**	0.40335	-0.11232	-0.2446854892385770	7536
**LOC388780**	0.59659	-0.58494	-0.24453052418778100	7538
**ZNF813**	0.59657	-1.0582	-0.24447887047588400	7539
**RASSF9**	0.40357	-1.3873	-0.24411731274725700	7540
**C17orf64**	0.59623	-0.70488	-0.2436008570436130	7541
**VSTM2L**	0.40392	-0.52718	-0.24321355790688200	7542
**COMMD1**	0.40393	-1.9169	-0.2431877392621210	7545
**FBXO3**	0.40393	-2.1207	-0.2431877392621210	7547
**KDELC2**	0.40393	-2.1375	-0.2431877392621210	7548
**PRR34**	0.40393	-1.4174	-0.2431877392621210	7543
**SSX1**	0.40393	-1.7408	-0.2431877392621210	7544
**WIPF3**	0.40393	-2.0664	-0.2431877392621210	7546
**CRBN**	0.40407	-1.1633	-0.2428262952473740	7549

**CHMP6**	0.40408	-0.2535	-0.24280047903211600	7551
**PGD**	0.40408	-0.20781	-0.24280047903211600	7550
**KIAA1468**	0.40413	-0.25744	-0.24267140038264300	7552
**PFKL**	0.59577	-0.69138	-0.24241325521006300	7553
**C8orf58**	0.40426	-1.2062	-0.24233581480889500	7554
**PHC1**	0.40428	-1.8999	-0.24228418868215000	7555
**HIGD1C**	0.40435	-1.1788	-0.24210350232253100	7556
**FGFBP2**	0.40444	-1.3897	-0.2418712029009830	7557
**WASF2**	0.40447	-0.98496	-0.24179377266133900	7558
**SLC12A9**	0.40461	-0.58334	-0.24143245070117800	7559
**PPP1R3A**	0.5953	-0.99838	-0.2412001889437840	7560
**CCDC125**	0.40476	-0.53364	-0.24104535500104100	7561
**PRMT5**	0.40482	-0.48598	-0.24089052683654600	7562
**SLC35D2**	0.59515	-1.0435	-0.24081311491984700	7563
**ANXA11**	0.40491	-0.67529	-0.24065829541495800	7564
**TMEM171**	0.40496	-2.1668	-0.2405292835670890	7565
**GALNT8**	0.40498	-1.0869	-0.24047767994893900	7566
**BNIPL**	0.40508	-0.19944	-0.24021967146023000	7568
**CSGALNAC**	0.59492	-0.096895	-0.24021967146023000	7567
**RNF113B**	0.40518	-0.68417	-0.23996167896086900	7569
**GGT6**	0.59474	-0.09492	-0.23975529646084000	7570
**RHOQ**	0.59472	-0.021743	-0.23970370243170300	7571
**RGCC**	0.40529	-0.92598	-0.23967790565641000	7572
**ZNF592**	0.5947	-0.74744	-0.2396521090406080	7573
**C1QTNF3**	0.59456	-0.81958	-0.23929097315525900	7574
**DDX54**	0.4055	-0.61617	-0.23913621018828800	7575
**ARSH**	0.40558	-0.46184	-0.2389298684739620	7576
**MSRB1**	0.59442	-0.6149	-0.23892986847396100	7577
**CLDN10**	0.59433	-0.54617	-0.23869774620350800	7578
**TMEM196**	0.59428	-0.33124	-0.23856879494424100	7579
**OSCAR**	0.59423	-0.64604	-0.23843984765169900	7580
**ENPP1**	0.40578	-0.63419	-0.2384140586689880	7581

**PLA2G4C**	0.40578	-0.74344	-0.2384140586689880	7582
**CENPX**	0.40597	-0.3614	-0.2379240981010350(	7583
**SNRNP25**	0.406	-1.1503	-0.2378467411297780(	7584
**TMEM38B**	0.59396	-0.090633	-0.2377436007152270(	7585
**KRT85**	0.40609	-0.55506	-0.2376146787531990	7586
**RECQL5**	0.59383	-0.16165	-0.237408411827018	7587
**CPLX1**	0.40622	-1.2234	-0.2372795001275990	7588
**P2RX5**	0.59372	-1.0584	-0.2371248112926710	7589
**OR11H6**	0.40638	-0.43329	-0.2368670091737750(	7590
**AKAP11**	0.40639	-0.38403	-0.2368412298279530(	7591
**DCAF1**	0.40639	-1.7538	-0.2368412298279530(	7594
**RNASE12**	0.40639	-1.1406	-0.2368412298279530(	7593
**SEPT4**	0.40639	-0.54366	-0.2368412298279530(	7592
**PIGG**	0.40646	-1.6387	-0.2366607788130670(	7595
**EMB**	0.4065	-0.87266	-0.2365576674074970(	7596
**GNGT2**	0.59343	-0.073506	-0.2363772284984840(	7597
**EML3**	0.40664	-1.897	-0.2361967972848090(	7598
**PRR32**	0.5932	-0.040109	-0.2357844119438740	7599
**LIN7B**	0.4069	-0.35181	-0.2355266914700330	7600
**THEMIS2**	0.5931	-0.64629	-0.2355266914700330(	7601
**PACSIN2**	0.40702	-0.67471	-0.2352174475471570(	7602
**CXCL12**	0.40711	-0.24015	-0.2349855293669050(	7603
**HPDL**	0.59286	-0.38102	-0.2349082261158750(	7604
**EPS8L1**	0.40722	-1.1061	-0.2347020909741340	7606
**FAM135B**	0.40722	-0.8738	-0.2347020909741340	7605
**HFE**	0.40725	-1.4425	-0.2346247928673950(	7607
**RPL3L**	0.59272	-0.31829	-0.2345474961624480	7608
**RBMXL2**	0.40733	-0.033068	-0.2344186714345700(	7609
**FERMT2**	0.59264	-0.62769	-0.2343413784652280	7610
**TSPAN8**	0.40737	-1.4249	-0.2343156144532310(	7611
**RNF10**	0.4076	-1.2844	-0.2337230850642930(	7612
**TXNDC11**	0.59237	-1.3178	-0.2336458046757170(	7613

**SUPT20HL1**	0.40765	-1.9311	-0.23359428519186600	7614
**SLFN13**	0.40766	-0.32249	-0.23356852568244400	7615
**GIPC1**	0.4079	-0.55194	-0.23295034390571900	7616
**KRT86**	0.4079	-0.66232	-0.23295034390571900	7617
**RAD54L**	0.59187	-0.57365	-0.2323580032255470	7618
**FAM78A**	0.40814	-0.90093	-0.23233225113363300	7619
**CDS2**	0.59183	-0.44958	-0.23225499578222400	7620
**MS4A6E**	0.4082	-1.1015	-0.232177741816912	7621
**C9orf142**	0.40824	-0.99927	-0.23207473868509700	7622
**SYT8**	0.40833	-0.78437	-0.2318429906385520	7623
**SLC7A4**	0.40845	-0.20767	-0.23153401260800500	7624
**TMEFF2**	0.40867	-1.1872	-0.23096761026378800	7625
**B4GALNT3**	0.40869	-1.2699	-0.23091612281818500	7630
**EMD**	0.40869	-0.82399	-0.23091612281818500	7629
**GJC2**	0.40869	-2.601	-0.23091612281818500	7632
**GTF2H3**	0.40869	-0.1916	-0.23091612281818500	7626
**HOXD4**	0.40869	-0.55542	-0.23091612281818500	7628
**SLC47A2**	0.40869	-0.22836	-0.23091612281818500	7627
**TBCB**	0.40869	-2.5	-0.23091612281818500	7631
**OR1S2**	0.59129	-1.0529	-0.23086463598469300	7633
**IKZF1**	0.40881	-2.2709	-0.2306072109936260	7634
**TPSG1**	0.40887	-0.11493	-0.2304527633346600	7635
**TRPV2**	0.59092	-0.38112	-0.22991223978863800	7636
**RPS28**	0.40911	-0.035348	-0.2298350276257950	7637
**GALC**	0.40918	-0.74515	-0.2296548712398590	7638
**ENSA**	0.40927	-1.1931	-0.22942325255135400	7639
**CYTH2**	0.40928	-1.2792	-0.22939751790144200	7640
**UNC5D**	0.59067	-0.72543	-0.2292688469302300	7641
**RAB41**	0.40934	-0.62257	-0.229243113191526	7642
**PTK2**	0.59057	-0.45509	-0.22901151637202900	7643
**CMTM1**	0.40962	-0.77935	-0.22852263007070000	7644
**CDSN**	0.59011	-0.014174	-0.22782799081637300	7645

**HIST1H4H**	0.41009	-0.9183	-0.2273135141018960(	7646
**VN1R5**	0.41025	-0.52748	-0.226901976050346	7647
**CD164L2**	0.41027	-0.52579	-0.2268505364971230(	7648
**ILVBL**	0.5897	-0.33688	-0.2267733782926460(	7649
**NFATC1**	0.41038	-1.0629	-0.2265676296792200(	7650
**RNF212B**	0.4104	-0.82113	-0.2265161940247140(	7651
**XDH**	0.41043	-1.1977	-0.2264390416664810(	7652
**CEP85L**	0.58954	-0.21189	-0.2263618906560310	7653
**OR2T12**	0.58944	-0.75083	-0.2261047303486860(	7654
**P2RY12**	0.41057	-2.2024	-0.2260790151405660	7655
**DIRAS1**	0.41068	-0.46238	-0.2257961577135330	7656
**MAPK8IP2**	0.41077	-1.0319	-0.2255647423463740(	7657
**CRISP2**	0.58916	-0.66758	-0.2253847609675820	7658
**C6orf58**	0.5891	-0.41874	-0.2252304970250030(	7659
**RSPO4**	0.41094	-0.32739	-0.2251276573743280	7660
**IL21R**	0.58904	-0.07626(	-0.2250762384418530(	7661
**CRLF1**	0.411	-0.56376	-0.2249734023618840	7662
**C4orf45**	0.41102	-1.1784	-0.2249219852140890	7666
**CKB**	0.41102	-2.3536	-0.2249219852140890	7671
**CLEC4M**	0.41102	-1.8401	-0.2249219852140890	7667
**FGF13**	0.41102	-0.47944	-0.2249219852140890	7663
**MAN1A2**	0.41102	-1.0184	-0.2249219852140890	7665
**MAP3K20**	0.41102	-2.0784	-0.2249219852140890	7668
**RABL2B**	0.41102	-0.66326	-0.2249219852140890	7664
**TMEM247**	0.41102	-2.3055	-0.2249219852140890	7670
**ZNF230**	0.41102	-2.2574	-0.2249219852140890	7669
**DBF4B**	0.58896	-0.097561	-0.2248705686608880	7672
**MBOAT4**	0.41109	-1.2052	-0.2247420298782500(	7673
**PHACTR3**	0.58877	-0.35648	-0.2243821410322240(	7674
**GPR18**	0.41142	-0.05066	-0.2238937669238540	7675
**TTC9**	0.58847	-0.59977	-0.2236110484308200(	7676
**GNG5**	0.58831	-0.69205	-0.2231998534272750(	7677

**SLC28A2**	0.58811	-1.0626	-0.2226859127265820(	7678
**SCGB1D4**	0.58802	-0.42548	-0.2224546586067270(	7679
**DCK**	0.41201	-1.2036	-0.2223775765441780(	7680
**CRISPLD2**	0.58796	-0.47987	-0.2223004958028240(	7681
**KRTAP19-3**	0.58792	-0.66728	-0.2221977235353410	7682
**CASP8AP2**	0.58788	-0.99209	-0.2220949536145620(	7683
**GAPDHS**	0.58787	-0.016211	-0.2220692615009010(	7684
**PTH**	0.41217	-0.38114	-0.2219664945118260(	7685
**PCOLCE**	0.41222	-0.32618	-0.221838039071622	7686
**CREB3L4**	0.58765	-0.33167	-0.2215040720510390(	7687
**NMT1**	0.5876	-0.40718	-0.2213756297766770(	7688
**SLC25A40**	0.41241	-1.2251	-0.2213499417600980(	7689
**COX5A**	0.41256	-0.49513	-0.2209646390275250(	7690
**CCDC7**	0.58723	-0.86539	-0.2204252702909300	7691
**POMGNT2**	0.41283	-2.1283	-0.2202711767213170(	7692
**ABHD13**	0.41289	-0.66072	-0.2201170883815420(	7693
**KCTD19**	0.58701	-0.79686	-0.2198602860928820(	7695
**YRDC**	0.58701	-0.13956	-0.2198602860928820(	7694
**ILF2**	0.413	-1.1135	-0.2198346066616900(	7696
**SYCE3**	0.4131	-0.22425	-0.2195778203193740(	7697
**EPB41**	0.58688	-0.75511	-0.2195264647887670	7698
**CARD8**	0.58686	-0.63495	-0.2194751098370980	7699
**FFAR2**	0.41315	-0.42455	-0.2194494325783200	7700
**SF3A2**	0.41315	-2.6028	-0.2194494325783200	7702
**TADA3**	0.41315	-1.8969	-0.2194494325783200	7701
**CEACAM3**	0.58678	-0.31532	-0.2192696958168410(	7703
**ACAD10**	0.5867	-0.70881	-0.2190642910477250	7704
**PBDC1**	0.41331	-0.49936	-0.2190386161015830	7705
**SMAD9**	0.41362	-0.11131	-0.2182427642927340(	7706
**BMI1**	0.58612	-1.0835	-0.2175753822630580(	7707
**CTNNA2**	0.4139	-0.75791	-0.2175240492011250(	7708
**ELOA**	0.58606	-0.37521	-0.2174213847965760(	7709

**MAGEB2**	0.41403	-0.75481	-0.21719039826260500	7710
**OCM**	0.58593	-0.34856	-0.21708774130089800	7711
**UBE2U**	0.41407	-0.46037	-0.21708774130089800	7712
**CLDN24**	0.4141	-0.57839	-0.21701075008091300	7713
**PCDH11X**	0.58585	-0.4416	-0.2168824342390680	7714
**PTK7**	0.41416	-0.77504	-0.21685677149925200	7715
**HOXC8**	0.41422	-0.32086	-0.21670279805867500	7716
**FGFR2**	0.58577	-0.96251	-0.2166771363181260	7717
**CDKN1A**	0.58574	-0.5761	-0.21660015195247500	7718
**HMGB2**	0.41432	-1.0271	-0.2164461870714950	7720
**TREML1**	0.41432	-0.12562	-0.2164461870714950	7719
**SUPT5H**	0.58562	-0.72039	-0.21629222732095100	7721
**CSNK1G3**	0.41441	-0.77217	-0.21621524936834500	7722
**CAVIN1**	0.41448	-0.86462	-0.2160356391268390	7723
**CRIP1**	0.41455	-0.90379	-0.21585603585394200	7724
**CPNE4**	0.41458	-0.38688	-0.21577906515437700	7725
**FCAMR**	0.5854	-0.069471	-0.2157277520648580	7726
**IRF2**	0.5854	-0.59245	-0.2157277520648580	7727
**WFDC9**	0.58536	-0.47276	-0.21562512758961100	7728
**BFAR**	0.58531	-1.0545	-0.21549685018863800	7729
**IGFL2**	0.41487	-0.35961	-0.2150350808871450	7730
**IL1R1**	0.58507	-0.76846	-0.21488116797972400	7731
**ZFAND3**	0.41495	-1.7387	-0.21482986480853500	7732
**ALAS2**	0.58502	-0.43179	-0.21475291111182500	7733
**CISD3**	0.41505	-1.8273	-0.21457335743059500	7735
**FOXJ3**	0.41505	-1.9928	-0.21457335743059500	7736
**UBXN2B**	0.41505	-1.4678	-0.21457335743059500	7734
**DPYSL3**	0.41519	-0.20893	-0.21421427081304800	7737
**ABLIM2**	0.58479	-1.1338	-0.2141629749805160	7739
**ALDH9A1**	0.58479	-0.75717	-0.2141629749805160	7738
**TMEM63A**	0.41527	-2.329	-0.21400909086321300	7740
**LAMTOR5**	0.41537	-2.5756	-0.21375262859317600	7741

**ZFP62**	0.58461	-0.39605	-0.2137013378266620(	7742
**PRH1**	0.58457	-0.6437	-0.2135987579799520	7743
**WBP1L**	0.41543	-1.2391	-0.2135987579799510(	7744
**ADAM12**	0.41551	-0.45905	-0.2133936050276720(	7746
**CYB5R2**	0.41551	-1.8314	-0.2133936050276720(	7755
**EPN2**	0.41551	-0.55916	-0.2133936050276720(	7749
**EYA4**	0.41551	-1.0557	-0.2133936050276720(	7751
**FUT1**	0.41551	-1.9415	-0.2133936050276720(	7756
**GATAD2A**	0.41551	-0.53224	-0.2133936050276720(	7748
**LRRC74A**	0.41551	-0.59558	-0.2133936050276720(	7750
**PIN1**	0.41551	-0.25548	-0.2133936050276720(	7745
**PLOD3**	0.41551	-1.786	-0.2133936050276720(	7754
**SDCBP**	0.41551	-1.7276	-0.2133936050276720(	7753
**SLC7A14**	0.41551	-1.525	-0.2133936050276720(	7752
**TAS1R2**	0.41551	-2.1974	-0.2133936050276720(	7757
**TRIP4**	0.41551	-0.48417	-0.2133936050276720(	7747
**SCFD2**	0.41554	-0.58276	-0.2133166749863170(	7758
**GSX1**	0.41566	-0.49086	-0.2130089674395590(	7759
**ZNF699**	0.41567	-0.73683	-0.2129833260547060(	7760
**ZNF717**	0.41577	-0.36901	-0.2127269199044350(	7761
**RHOBTB2**	0.58417	-0.28992	-0.2125730829277500(	7762
**MEF2A**	0.41586	-1.9543	-0.2124961663260010(	7763
**CPT1A**	0.58412	-0.35875	-0.2124448892899040(	7764
**APEX1**	0.41593	-0.76259	-0.2123166991429940(	7765
**FAM149B1**	0.58405	-0.2752	-0.2122654240611780(	7766
**ZFHX2**	0.58399	-1.0563	-0.2121116021633540(	7767
**BCS1L**	0.41604	-1.4955	-0.2120346930965710(	7768
**MYBBP1A**	0.41635	-0.17259	-0.2112400394168890	7769
**SSNA1**	0.58361	-0.19796	-0.2111375131840000(	7770
**ARMS2**	0.41643	-1.1834	-0.2110349891703300(	7771
**UBE2J1**	0.5835	-0.18488	-0.2108555774828740	7772
**FOXA1**	0.58346	-0.89354	-0.2107530595660050	7773

**KRTAP4-8**	0.4166	-1.7053	-0.21059928684312000	7774
**YME1L1**	0.58337	-0.93792	-0.21052240234919300	7775
**CISH**	0.58335	-0.051137	-0.21047114671124600	7776
**RBM6**	0.58335	-0.064646	-0.21047114671124600	7777
**PLAG1**	0.41674	-2.3296	-0.21024050318048000	7778
**LOC1027249**	0.41688	-0.42213	-0.20988174657753900	7779
**ACAD11**	0.41693	-1.2828	-0.2097536257676110	7780
**TBL1Y**	0.58303	-0.79123	-0.20965113159859100	7781
**SUPT20HL2**	0.58293	-0.83583	-0.20939490580817600	7782
**DIS3L2**	0.41709	-1.4579	-0.20934366230014500	7783
**YPEL5**	0.41728	-0.47135	-0.2088568763682560	7784
**MXD3**	0.58263	-0.28269	-0.20862631083710000	7785
**C1orf228**	0.41743	-0.39611	-0.20847260664463300	7786
**IKBKG**	0.4175	-0.25147	-0.20829329131075500	7788
**PAPOLG**	0.4175	-1.2104	-0.20829329131075500	7789
**PLEKHA5**	0.4175	-0.006922	-0.20829329131075500	7787
**LOC730159**	0.41764	-1.2306	-0.207934680727141	7790
**KIAA1257**	0.58233	-0.68089	-0.20785783908233900	7791
**R3HCC1L**	0.58229	-1.0008	-0.20775538546487600	7792
**NXF3**	0.58225	-0.26474	-0.20765293402798900	7793
**ACY3**	0.41803	-2.234	-0.20693583492777700	7801
**JSRP1**	0.41803	-0.46202	-0.20693583492777700	7794
**NGB**	0.41803	-2.3009	-0.20693583492777700	7802
**OR6S1**	0.41803	-1.6707	-0.20693583492777700	7797
**PAGE2**	0.41803	-2.3804	-0.20693583492777700	7803
**RBFOX2**	0.41803	-2.2203	-0.20693583492777700	7800
**SMC3**	0.41803	-1.822	-0.20693583492777700	7798
**SPDYA**	0.41803	-2.0568	-0.20693583492777700	7799
**TANGO6**	0.41803	-1.3184	-0.20693583492777700	7796
**TRIM9**	0.41803	-0.56185	-0.20693583492777700	7795
**UBE2E2**	0.41813	-0.020624	-0.2066797539104740	7804
**FAM19A1**	0.58168	-0.34912	-0.20619323729825300	7805

**SPANXA2**	0.58149	-1.1596	-0.2057067694837290	7806
**NDUFA8**	0.41861	-0.058145	-0.20545075334980900	7807
**GPR82**	0.58136	-0.34841	-0.2053739511360520	7808
**JADE3**	0.58134	-0.27413	-0.20532275033320000	7809
**TSPY4**	0.58129	-0.13771	-0.2051947506804590	7810
**UNC93A**	0.4189	-1.3664	-0.20470838263843200	7811
**LLGL1**	0.58105	-0.28475	-0.20458039910061200	7812
**STARD7**	0.58093	-0.27294	-0.20427325227631400	7813
**CBLN2**	0.58085	-0.35435	-0.20406849843346900	7814
**RNF225**	0.5808	-0.40875	-0.2039405316263620	7815
**CUX1**	0.4195	-0.47229	-0.203172800830251	7816
**POT1**	0.41955	-0.50113	-0.20304485735081400	7817
**ADK**	0.41983	-2.1586	-0.2023284352191360	7831
**CREB3L2**	0.41983	-0.33391	-0.2023284352191360	7820
**DYSF**	0.41983	-1.0132	-0.2023284352191360	7826
**HCN2**	0.41983	-1.1515	-0.2023284352191360	7827
**ITGB3**	0.41983	-2.3347	-0.2023284352191360	7833
**NIT2**	0.41983	-0.16089	-0.2023284352191360	7818
**NKX2-6**	0.41983	-0.31467	-0.2023284352191360	7819
**PDE4B**	0.41983	-2.2387	-0.2023284352191360	7832
**POLR1A**	0.41983	-0.53317	-0.2023284352191360	7823
**PQBP1**	0.41983	-1.4051	-0.2023284352191360	7828
**PRDM11**	0.41983	-1.8447	-0.2023284352191360	7830
**SERPINB7**	0.41983	-0.60363	-0.2023284352191360	7824
**TLR7**	0.41983	-1.0093	-0.2023284352191360	7825
**TSGA10**	0.41983	-0.42755	-0.2023284352191360	7822
**ZFYVE28**	0.41983	-1.808	-0.2023284352191360	7829
**ZIM3**	0.41983	-0.42519	-0.2023284352191360	7821
**MICU1**	0.58011	-0.21062	-0.20217492971238800	7834
**ILDR2**	0.41991	-1.367	-0.20212376226890700	7835
**LEMD1**	0.41991	-1.8794	-0.20212376226890700	7837
**MPIG6B**	0.41991	-1.4496	-0.20212376226890700	7836

**MYOG**	0.41991	-2.1371	-0.20212376226890700	7838
**WNK3**	0.41991	-2.2159	-0.20212376226890700	7839
**GRM5**	0.57982	-0.51659	-0.2014330535126310	7840
**SCN2A**	0.42084	-0.66006	-0.1997450580933740	7841
**ZC3H12A**	0.57904	-0.68784	-0.19943821111006100	7842
**SCRT2**	0.42102	-0.37953	-0.19928479466125900	7843
**UTS2B**	0.4211	-0.59353	-0.1990802466911290	7844
**ACP5**	0.57878	-0.78489	-0.19877344034918800	7845
**OR1A2**	0.57869	-0.26922	-0.19854334787169400	7846
**HMGA1**	0.57862	-0.32159	-0.1983643943228630	7848
**SELENOO**	0.57862	-0.1104	-0.1983643943228630	7847
**TWISTNB**	0.4214	-0.68486	-0.19831326590442000	7849
**CDC20**	0.42151	-2.4368	-0.1980320688650580	7858
**CERS4**	0.42151	-0.16589	-0.1980320688650580	7850
**CHP1**	0.42151	-1.0128	-0.1980320688650580	7851
**OPRPN**	0.42151	-2.0549	-0.1980320688650580	7855
**SPAG7**	0.42151	-1.501	-0.1980320688650580	7852
**SSFA2**	0.42151	-1.895	-0.1980320688650580	7853
**SYCP2**	0.42151	-1.9257	-0.1980320688650580	7854
**UNC119**	0.42151	-2.1381	-0.1980320688650580	7856
**XPO6**	0.42151	-2.2213	-0.1980320688650580	7857
**HELT**	0.42153	-0.99439	-0.19798094381347200	7859
**C10orf10**	0.57827	-0.95186	-0.19746972173289200	7860
**TPP2**	0.42178	-2.3197	-0.19734192428082500	7861
**NFS1**	0.42185	-0.71856	-0.19716301326161500	7862
**IQSEC1**	0.42203	-0.47872	-0.19670298531646600	7863
**COLCA2**	0.42204	-0.39246	-0.19667742942973500	7865
**LOXL2**	0.57796	-0.27406	-0.19667742942973500	7864
**TEX45**	0.42224	-1.091	-0.1961663386439250	7866
**C7orf69**	0.42233	-0.5091	-0.1959363645136070	7867
**KCNE1**	0.57759	-0.5292	-0.19573195176342300	7868
**ZFP36L2**	0.57755	-0.67721	-0.19562974845554200	7869

**PTGDR**	0.57749	-0.36197	-0.19547644732450700	7870
**KLKB1**	0.57746	-0.045054	-0.1953997984817900	7871
**UBE2L6**	0.4226	-0.85015	-0.19524650423953800	7872
**CXorf23**	0.57702	-0.086478	-0.1942757470813190	7873
**MROH2A**	0.42298	-0.45451	-0.1942757470813190	7874
**TARS**	0.42309	-0.99569	-0.1939947726339140	7875
**KLC4**	0.42312	-0.85759	-0.19391814589786100	7876
**GPM6B**	0.42316	-1.0764	-0.19381597868741700	7877
**CCDC186**	0.57683	-0.9095	-0.1937904372009290	7879
**HS1BP3**	0.57683	-0.53404	-0.1937904372009290	7878
**CAAP1**	0.42332	-0.34595	-0.19340733006318300	7880
**FAM126A**	0.42332	-2.5874	-0.19340733006318300	7884
**GNB1**	0.42332	-0.54198	-0.19340733006318300	7881
**KLF12**	0.42332	-1.909	-0.19340733006318300	7882
**NACC2**	0.42332	-2.6151	-0.19340733006318300	7885
**SLC17A6**	0.42332	-2.2412	-0.19340733006318300	7883
**MAGEA3**	0.42337	-0.32015	-0.19327963399415600	7886
**LAMB2**	0.42341	-0.79908	-0.19317747940803600	7887
**POTEF**	0.42347	-0.98649	-0.1930242513080070	7888
**PRSS41**	0.57636	-0.47044	-0.19259012962057100	7889
**CALHM3**	0.42369	-0.84333	-0.19246245368172600	7890
**MTHFD2L**	0.42378	-2.2714	-0.19223264489571100	7891
**PSG5**	0.42382	-0.69976	-0.19213051091662300	7892
**FCGR1B**	0.57603	-1.0872	-0.191747526333862	7893
**TAS2R50**	0.42397	-2.2227	-0.191747526333862	7894
**TTC19**	0.57599	-0.55964	-0.19164540186360500	7895
**ACTA2**	0.42404	-1.4816	-0.19156880982253800	7896
**SH3YL1**	0.42409	-1.1602	-0.19144115891766300	7897
**CD80**	0.42412	-0.79313	-0.19136456987209500	7898
**SARS**	0.42414	-0.92926	-0.19131351113198400	7899
**LRRC41**	0.57584	-0.7053	-0.19126245289058700	7900
**GPR85**	0.57582	-0.67322	-0.19121139514776100	7902

**POLR1C**	0.42418	-0.14923	-0.19121139514776100	7901
**EDDM3B**	0.5758	-0.46499	-0.19116033790336500	7903
**ZNF254**	0.4243	-1.9311	-0.19090505915280700	7904
**VSX2**	0.57553	-0.86254	-0.19047111381547900	7905
**CCL14**	0.5753	-0.9162	-0.18988406836508300	7906
**COX7A2**	0.57517	-0.66785	-0.1895522890219430	7907
**PDCL2**	0.42484	-0.21858	-0.1895267683986430	7908
**GCFC2**	0.4249	-0.35922	-0.1893736472502500	7909
**IQCA1L**	0.42494	-1.0437	-0.189271568951354	7910
**BCL2A1**	0.57495	-0.35082	-0.1889908637942460	7911
**RPS23**	0.42509	-0.91175	-0.1888887928845880	7912
**OR1Q1**	0.57489	-0.12868	-0.1888377581677390	7913
**ALOX15**	0.42519	-2.0659	-0.1886336242168890	7918
**KDM3A**	0.42519	-1.3741	-0.1886336242168890	7915
**LAMA3**	0.42519	-0.17955	-0.1886336242168890	7914
**OR4A15**	0.42519	-1.7993	-0.1886336242168890	7916
**TCTEX1D2**	0.42519	-1.9436	-0.1886336242168890	7917
**OR1E2**	0.4252	-1.1665	-0.18860810802585000	7919
**CES2**	0.42522	-0.40973	-0.18855707601212300	7920
**TBXAS1**	0.42561	-1.0287	-0.1875620497104170	7921
**GSDMB**	0.57412	-0.76517	-0.18687329416649800	7923
**ULK2**	0.57412	-0.54249	-0.18687329416649800	7922
**OR10A3**	0.57401	-0.8136	-0.1865927154861000	7924
**ZNF200**	0.57396	-0.39535	-0.18646518457910500	7925
**GPR75-ASB**	0.42612	-0.75994	-0.1862611414344000	7926
**BPIFA1**	0.42631	-1.9997	-0.18577657002513800	7927
**SLC35A3**	0.42642	-0.70404	-0.1854960486256050	7928
**CTNND1**	0.42652	-0.47299	-0.1852410418374540	7929
**ZFAND5**	0.42662	-1.3607	-0.18498604709381900	7930
**CNTNAP4**	0.42677	-0.13962	-0.1846035775230790	7931
**BMP2**	0.57314	-1.1395	-0.1843741087447140	7933
**DPF2**	0.57314	-1.6496	-0.1843741087447140	7934

**RPUSD4**	0.57314	-0.59739	-0.1843741087447140	7932
**SNX6**	0.42702	-0.012786	-0.1839661882101910	7935
**KLHDC8B**	0.42707	-0.61719	-0.1838387193234530(	7936
**HEPN1**	0.5729	-0.60024	-0.1837622394252080(	7937
**HGD**	0.42712	-1.2389	-0.1837112534235010(	7938
**SGMS2**	0.42714	-0.86181	-0.1836602678993250(	7939
**LOC157860**	0.57281	-0.68828	-0.18353280617716	7940
**TPD52L3**	0.42721	-0.98807	-0.1834818223232320	7941
**MN1**	0.42726	-0.48603	-0.1833543647745220(	7942
**FTL**	0.42727	-1.0203	-0.1833288736222570	7943
**POLA1**	0.42741	-1.7096	-0.1829720099898740(	7944
**ADGRL2**	0.42748	-0.68955	-0.1827935869136860(	7945
**JPH4**	0.42752	-0.2822	-0.1826916334829670(	7946
**TRIM68**	0.42755	-0.1267	-0.1826151696560870(	7947
**PLIN5**	0.42758	-1.3326	-0.1825387068968150	7948
**RBM11**	0.42769	-0.28057	-0.1822583525724220(	7949
**FLAD1**	0.42781	-1.3893	-0.1819525278263220(	7950
**MFAP1**	0.57202	-0.48621	-0.1815193052228750	7951
**PLSCR1**	0.57199	-0.76108	-0.1814428577136010(	7952
**NDUFA12**	0.42803	-0.63661	-0.1813918932965040(	7953
**PRKCA**	0.42808	-0.47291	-0.1812644843145320	7954
**CAMKK2**	0.42814	-0.02909	-0.1811115974196700(	7955
**TRPM4**	0.4282	-0.77301	-0.1809587147577590(	7956
**CRK**	0.5717	-0.08991	-0.1807039197174620(	7957
**TRANK1**	0.42833	-2.4448	-0.1806274834935250(	7958
**ART4**	0.57162	-0.28488	-0.1805000921316710(	7959
**SULT1C3**	0.57151	-0.73471	-0.1802198414424920(	7960
**MMP28**	0.57136	-0.013216	-0.1798377042154580(	7961
**CCDC160**	0.5712	-0.40977	-0.1794301201137490(	7962
**INPP5K**	0.42883	-0.036772	-0.1793537014148120(	7963
**TAS1R1**	0.57113	-0.30448	-0.1792518114452860(	7964
**NEUROG2**	0.4289	-0.36216	-0.1791753951893160	7965

**CETN1**	0.42891	-2.2267	-0.1791499233365040	7967
**SRSF7**	0.42891	-0.37386	-0.1791499233365040	7966
**PGAM4**	0.429	-0.81139	-0.1789206818892660(	7968
**ZNF682**	0.42916	-2.5015	-0.1785131647440110(	7969
**DHX30**	0.42944	-0.32147	-0.1778000810211560(	7970
**FBXO28**	0.42944	-1.8071	-0.1778000810211560(	7973
**RSPO2**	0.42944	-1.5752	-0.1778000810211560(	7972
**TMEM175**	0.42944	-1.4782	-0.1778000810211560(	7971
**MAGEA1**	0.42949	-0.53666	-0.1776727541650110	7974
**PLOD2**	0.57043	-0.9602	-0.1774690371850370(	7975
**PLA2G12B**	0.42961	-0.65264	-0.1773671814572130(	7976
**ANKRD46**	0.57035	-0.90887	-0.1772653275693320(	7977
**RIOK3**	0.42972	-0.75672	-0.1770870876895270(	7978
**BANF1**	0.57022	-0.42898	-0.1769343151272590(	7980
**PSMD3**	0.57022	-0.39682	-0.1769343151272590(	7979
**TRIM21**	0.4298	-0.71933	-0.1768833918576100	7981
**MAL**	0.42995	-0.95518	-0.1765014819451010(	7982
**C1orf185**	0.42997	-1.3147	-0.1764505625695280(	7983
**SAMD4A**	0.42998	-0.06436	-0.1764251030532970	7984
**ARHGEF18**	0.43006	-0.89878	-0.1762214310384930	7985
**ACBD5**	0.43023	-1.0962	-0.1757886522649250(	7986
**OR10J5**	0.56968	-0.51967	-0.1755595474235600(	7987
**SLC52A2**	0.43055	-1.6184	-0.1749740991040340(	7988
**ARHGAP10**	0.43056	-1.9638	-0.174948646191003	7989
**UBAC1**	0.56921	-0.039734	-0.1743632604354620	7990
**CALML5**	0.43081	-0.34638	-0.1743123601525810(	7991
**MYH10**	0.56878	-0.61399	-0.1732690036697190(	7992
**ARHGAP17**	0.43129	-0.66443	-0.1730908885152340(	7993
**SRFBP1**	0.43137	-0.2136	-0.1728873350616800	7994
**C5orf34**	0.43159	-1.0328	-0.1723275999698630(	7997
**FAM189A2**	0.43159	-2.0233	-0.1723275999698630(	7998
**HDAC9**	0.43159	-0.40142	-0.1723275999698630(	7996

**ITPRIPL1**	0.43159	-2.2664	-0.1723275999698630(	8000
**KCND3**	0.43159	-0.35019	-0.1723275999698630(	7995
**NLGN3**	0.43159	-2.331	-0.1723275999698630(	8001
**TRIM56**	0.43159	-2.0938	-0.1723275999698630(	7999
**PALMD**	0.43167	-0.77237	-0.1721240733301180(	8002
**IFT20**	0.5683	-0.73937	-0.1720477526786730	8003
**SURF6**	0.43171	-0.68511	-0.1720223126842290	8004
**NAA38**	0.43176	-0.17508	-0.1718951143815040(	8005
**HPCAL4**	0.43182	-0.022077	-0.1717424800888250	8006
**SLC2A6**	0.43185	-0.22722	-0.1716661644429910(	8007
**DUOX1**	0.43188	-1.0202	-0.1715898497968680(	8008
**CCDC85C**	0.43189	-0.049215	-0.1715644118035690(	8009
**FN1**	0.56799	-0.64869	-0.1712591645377250(	8010
**TRAF3IP1**	0.43204	-1.437	-0.1711828552154410(	8011
**GNAO1**	0.43207	-0.8221	-0.1711065468898880(	8012
**LONP1**	0.43213	-0.5318	-0.1709539332270920(	8013
**RCAN3**	0.56784	-0.60086	-0.1708776278889080(	8014
**IFNAR1**	0.56782	-0.85747	-0.1708267582161760(	8015
**RAB40A**	0.43225	-0.70772	-0.1706487178415720(	8016
**ALDH4A1**	0.43231	-1.4673	-0.1704961161113250(	8017
**OR2G2**	0.43237	-1.3596	-0.1703435183510730(	8018
**APOBEC3F**	0.43246	-1.0349	-0.1701146291462130(	8019
**GAGE12G**	0.56751	-0.66717	-0.1700383347255180	8020
**ZNF470**	0.43268	-0.28078	-0.1695551596909530(	8021
**LRRC20**	0.5672	-0.10891	-0.1692500169106890(	8022
**RYBP**	0.56706	-0.95037	-0.1688940369136820(	8023
**PMP2**	0.567	-0.62259	-0.1687414806115240(	8024
**ACE**	0.43305	-0.88682	-0.168614353359524	8025
**SAMD11**	0.43306	-0.37821	-0.1685889282361660(	8026
**NLRP14**	0.43309	-0.83129	-0.1685126535198650(	8027
**BRDT**	0.43313	-1.7784	-0.1684109554229500(	8028
**ITGA11**	0.43315	-0.77967	-0.1683601070276730	8029

**SH3BP4**	0.43323	-0.15737	-0.1681567177978700	8030
**TRIM33**	0.56674	-0.54928	-0.1680804486303650(	8031
**TGFB1I1**	0.4333	-0.20344	-0.1679787579277110(	8032
**FARP2**	0.43332	-0.065603	-0.1679279132277950(	8033
**NAALAD2**	0.56663	-1.1077	-0.1678008033768390(	8034
**POLR1B**	0.43337	-1.9928	-0.1678008033768390(	8035
**SGK494**	0.56658	-0.42736	-0.1676736962367670	8036
**TSPAN4**	0.43363	-1.6286	-0.1671398758023840	8037
**GRM3**	0.43378	-0.49224	-0.1667586046572610	8038
**ASIC4**	0.43381	-1.4503	-0.1666823533386660(	8043
**EXOC3**	0.43381	-0.70917	-0.1666823533386660(	8042
**MPPED1**	0.43381	-2.0861	-0.1666823533386660(	8044
**POU1F1**	0.43381	-0.41481	-0.1666823533386660(	8039
**PUS7**	0.43381	-0.50237	-0.1666823533386660(	8041
**SPTY2D1**	0.43381	-2.3743	-0.1666823533386660(	8045
**VAMP4**	0.43381	-0.43061	-0.1666823533386660(	8040
**OR51I1**	0.56616	-0.082307	-0.1666061029891210	8046
**MED26**	0.43396	-0.32129	-0.1663011112720980(	8047
**TBC1D1**	0.56598	-0.069021	-0.1661486212157290(	8048
**MESDC2**	0.43404	-1.3509	-0.1660977920555930	8049
**KIAA0556**	0.56576	-0.8416	-0.1655895240316190	8050
**C19orf73**	0.43429	-0.3778	-0.1654624637140090(	8051
**FAM50A**	0.56564	-0.51596	-0.1652845837561960(	8052
**DPP6**	0.43441	-0.79587	-0.1651575298454150(	8053
**SLC7A2**	0.43467	-0.94717	-0.1644968924338180	8054
**KYAT1**	0.43469	-0.394	-0.1644460771484050(	8055
**LIG4**	0.4347	-0.94536	-0.1644206696649310(	8056
**SFTA2**	0.43488	-0.30666	-0.1639633530942040	8057
**LRRN1**	0.43507	-0.5198	-0.1634806672413170(	8058
**DNAJC5**	0.43512	-0.66882	-0.1633536509828440(	8059
**NOP2**	0.43525	-0.18155	-0.1630234210379630(	8060
**TNFRSF25**	0.43528	-0.98541	-0.1629472166533520(	8061

**KLHL29**	0.5647	-0.71273	-0.162896414255957	8062
**SMPD2**	0.43531	-0.77573	-0.1628710132149110	8063
**MAN2A1**	0.56464	-1.2203	-0.1627440095855130(	8065
**TEX30**	0.56464	-1.0887	-0.1627440095855130(	8064
**BAIAP3**	0.43547	-0.41952	-0.1624646108370990	8066
**ZNF852**	0.43547	-0.86187	-0.1624646108370990	8067
**BCL2L10**	0.56426	-0.50454	-0.1617788676255540(	8068
**ZBTB16**	0.56424	-0.7578	-0.1617280748620380(	8069
**PLA2G4E**	0.43578	-0.64339	-0.1616772825157300(	8070
**SF3B6**	0.43583	-2.0569	-0.1615503034743450(	8071
**SLC35F6**	0.56413	-0.50855	-0.1614487221166100(	8072
**AVPR1A**	0.43593	-0.65988	-0.1612963532030440	8074
**CYCS**	0.43593	-0.36015	-0.1612963532030440	8073
**SH3D19**	0.43598	-0.91495	-0.1611693819688200(	8075
**LOC283485**	0.43602	-0.056085	-0.1610678068521610(	8076
**VTI1A**	0.43609	-1.603	-0.1608900543957370	8077
**DUSP9**	0.56387	-0.80869	-0.1607884838460160	8078
**TLCD1**	0.43615	-1.6756	-0.1607376991932140(	8079
**MIXL1**	0.43616	-0.98359	-0.1607123070222680(	8080
**PTBP2**	0.56374	-0.53415	-0.1604583910086880(	8081
**N6AMT1**	0.56372	-0.43276	-0.1604076090478160(	8082
**EDNRB**	0.56363	-0.44319	-0.1601790953405360(	8083
**SLC46A1**	0.5636	-1.0912	-0.1601029259639290(	8084
**FAM13A**	0.43652	-1.5378	-0.1597982577408720	8086
**FBXW11**	0.43652	-1.4949	-0.1597982577408720	8085
**GKAP1**	0.43652	-2.3293	-0.1597982577408720	8089
**GLMP**	0.43652	-2.2851	-0.1597982577408720	8088
**PUF60**	0.43652	-1.8775	-0.1597982577408720	8087
**DHCR24**	0.56342	-0.97199	-0.1596459291928660	8090
**NTSR1**	0.4366	-0.1727	-0.1595951538335060(	8091
**OR2T6**	0.4366	-0.86733	-0.1595951538335060(	8092
**MYRF**	0.56327	-0.60067	-0.1592651240200000(	8093

**SBK3**	0.43689	-0.17523	-0.15885895728710700	8094
**USP30**	0.43696	-0.70542	-0.1586812675854170	8095
**TRIP10**	0.43705	-1.7252	-0.1584528167578090	8096
**ZMAT4**	0.43712	-0.93084	-0.15827513849806600	8097
**FOXG1**	0.43716	-1.0678	-0.15817361030748600	8099
**TBC1D23**	0.56284	-0.4544	-0.15817361030748600	8098
**HIST2H2BE**	0.43721	-1.3037	-0.15804670236174800	8100
**ZNF396**	0.43726	-1.4729	-0.1579197969612170	8101
**RPTN**	0.56273	-0.54678	-0.15789441618634500	8102
**SIGLEC5**	0.43733	-1.128	-0.15774213367233300	8103
**HSPA6**	0.43739	-2.0377	-0.15758985481600200	8104
**ZNF420**	0.4375	-1.1917	-0.15731068640024300	8105
**UBL5**	0.5624	-0.67717	-0.15705690756958800	8106
**DPYD**	0.56236	-0.6756	-0.15695539887018300	8107
**ZNF516**	0.56223	-1.146	-0.15662550675972600	8108
**SOX8**	0.43778	-0.2318	-0.1566001311499520	8109
**KLHL7**	0.4378	-2.1617	-0.15654938023287500	8110
**C11orf98**	0.43782	-0.063534	-0.15649862971898500	8111
**PNLIPRP3**	0.43782	-0.38185	-0.15649862971898500	8112
**METTL24**	0.43786	-0.099709	-0.15639712990021100	8113
**FAM122A**	0.43789	-1.0461	-0.1563210060934720	8114
**ZKSCAN8**	0.43792	-0.88413	-0.15624488319251100	8115
**C9orf170**	0.43798	-0.94963	-0.15609264010607500	8116
**EXOSC10**	0.43814	-1.1948	-0.15568667621806300	8117
**CASP4**	0.56184	-0.51005	-0.15563593253734900	8118
**UBXN6**	0.43834	-0.60436	-0.1551792574268230	8119
**UBTD2**	0.56158	-0.32184	-0.15497630110360400	8120
**PIH1D1**	0.43863	-0.022983	-0.15444357110177300	8121
**RCOR1**	0.43863	-0.31817	-0.15444357110177300	8122
**RAB5A**	0.43873	-0.9187	-0.15418990556143200	8123
**SPINK1**	0.43884	-0.35601	-0.15391088492451200	8124
**ACVR2B**	0.43921	-0.23344	-0.15297244875907300	8125

**BMP7**	0.43925	-2.4179	-0.15287100428357300	8134
**CD1B**	0.43925	-1.5608	-0.15287100428357300	8129
**LGALS13**	0.43925	-0.57863	-0.15287100428357300	8126
**NMRK2**	0.43925	-1.8806	-0.15287100428357300	8131
**NRARP**	0.43925	-2.3028	-0.15287100428357300	8133
**RGP1**	0.43925	-1.8331	-0.15287100428357300	8130
**SPINK13**	0.43925	-1.0959	-0.15287100428357300	8128
**UBA6**	0.43925	-1.044	-0.15287100428357300	8127
**UBXN4**	0.43925	-2.1294	-0.15287100428357300	8132
**SELENBP1**	0.43935	-0.5906	-0.15261739997456300	8135
**GK**	0.43939	-0.11113	-0.15251596099998700	8136
**NOMO2**	0.56054	-0.095929	-0.15233844656979300	8137
**ZCCHC11**	0.43948	-0.060231	-0.1522877290430420	8138
**CDRT4**	0.43972	-0.12586	-0.15167914923516500	8139
**P2RX1**	0.43982	-0.010787	-0.15142559090617400	8140
**ZNF462**	0.43989	-1.101	-0.15124810586884900	8141
**NCLN**	0.43995	-0.7904	-0.15109597962883300	8142
**DNTTIP1**	0.44	-1.5685	-0.15096921043317300	8143
**PNMA6A**	0.55999	-0.96531	-0.15094385688521900	8144
**TMEM64**	0.55999	-1.0826	-0.15094385688521900	8145
**IQGAP3**	0.44015	-0.24721	-0.15058891739322900	8146
**FAM207A**	0.44027	-0.36421	-0.1502846986426430	8147
**ICA1**	0.44038	-0.90422	-0.1500058436723420	8148
**C5orf51**	0.44043	-1.0287	-0.14987909527007400	8149
**PHYHD1**	0.44049	-1.3941	-0.14972700036522500	8150
**NUP210L**	0.44061	-0.53018	-0.14942282094174900	8151
**ZNF354A**	0.44064	-0.4789	-0.1493467782469980	8152
**SEMA7A**	0.44068	-2.6409	-0.1492453893305140	8153
**KLF13**	0.55927	-1.311	-0.14911865534207200	8155
**POP1**	0.55927	-0.76236	-0.14911865534207200	8154
**PLEKHJ1**	0.55923	-0.45191	-0.1490172698757230	8156
**ACTN2**	0.44082	-1.4354	-0.14889054019650300	8157

**ASPSCR1**	0.55898	-1.2043	-0.14838364536874600	8158
**SNCAIP**	0.44104	-1.1568	-0.1483329579848480	8159
**MTMR8**	0.44114	-0.78813	-0.148079526778691	8161
**OGG1**	0.55886	-0.44688	-0.148079526778691	8160
**ELAVL1**	0.5587	-0.45784	-0.14767405662208900	8162
**CALM1**	0.55862	-0.21345	-0.14747133065142500	8163
**FILIP1**	0.44169	-0.61384	-0.14668582467661200	8164
**SPAG8**	0.5583	-0.54519	-0.14666048728353800	8165
**SPTBN2**	0.55826	-0.64386	-0.1465591386525430	8166
**XKR7**	0.55814	-0.49928	-0.14625510178682200	8167
**AMELX**	0.44188	-0.33825	-0.1462044302909210	8168
**ARHGEF40**	0.44188	-1.7134	-0.1462044302909210	8170
**NEUROG1**	0.44188	-1.8327	-0.1462044302909210	8171
**NUPR1**	0.44188	-2.2601	-0.1462044302909210	8174
**OR7G2**	0.44188	-1.9164	-0.1462044302909210	8173
**PIK3CD**	0.44188	-1.87	-0.1462044302909210	8172
**TESMIN**	0.44188	-1.6014	-0.1462044302909210	8169
**LOC403312**	0.44191	-0.79577	-0.146128423750839	8175
**HERC2**	0.44204	-1.0103	-0.1457990718255370	8176
**EID2**	0.44208	-0.32822	-0.14569773595472700	8177
**PERM1**	0.44211	-0.68094	-0.1456217350334580	8178
**DYDC2**	0.55778	-1.122	-0.14534307218113000	8182
**ERCC5**	0.55778	-0.080593	-0.14534307218113000	8179
**MRPL14**	0.55778	-1.1107	-0.14534307218113000	8181
**NELFA**	0.44222	-0.38961	-0.14534307218113000	8180
**HLA-DMB**	0.44225	-0.26652	-0.14526707518093000	8183
**ZNF799**	0.44225	-0.68937	-0.14526707518093000	8184
**BEX2**	0.44241	-0.49159	-0.14486177200402600	8186
**CRTC1**	0.55759	-0.025221	-0.14486177200402600	8185
**ADAM10**	0.44244	-0.069022	-0.1447857803093470	8187
**TRMT5**	0.44248	-0.34093	-0.14468445935019800	8188
**SPERT**	0.55741	-0.89932	-0.14440583436736800	8189

**KAAG1**	0.55737	-0.40645	-0.1443045189721510	8190
**PPT1**	0.55733	-0.49687	-0.14420320505807700	8191
**FAM110B**	0.44278	-0.52487	-0.14392459942267700	8192
**MUT**	0.55703	-0.35651	-0.143443397804655000	8193
**B4GALNT4**	0.44307	-0.76744	-0.1431901471419900	8194
**RFX4**	0.55683	-0.59586	-0.142936905662049000	8195
**SERPINB5**	0.44321	-1.8711	-0.142835611637449000	8196
**MAP10**	0.55678	-0.24915	-0.142810288360318000	8197
**C11orf52**	0.55666	-0.76825	-0.1425064161726630	8198
**CHD7**	0.44334	-0.20178	-0.1425064161726620	8199
**LOC283454**	0.44334	-0.27564	-0.1425064161726620	8200
**NCAPH2**	0.44342	-1.1317	-0.1423038420257060	8201
**CDHR5**	0.44346	-1.4762	-0.142202557142429000	8202
**ASCC2**	0.44363	-0.18576	-0.1417721126489830	8203
**HYPK**	0.44367	-0.036767	-0.141670835411788000	8204
**CBLN1**	0.55619	-0.46861	-0.1413163765170490	8205
**POLQ**	0.55616	-0.38713	-0.141240423350908000	8206
**LCORL**	0.44397	-1.2206	-0.140911302373248000	8207
**IRGC**	0.44401	-0.7586	-0.140810037452300000	8208
**PLGLB2**	0.44405	-1.0149	-0.1407087739751940	8209
**NRM**	0.5559	-0.18903	-0.1405821966575700	8210
**GAL3ST3**	0.44414	-0.36524	-0.140480936425162000	8211
**FAM204A**	0.5558	-1.2832	-0.140329048776840000	8213
**TMEM189**	0.5558	-0.86723	-0.140329048776840000	8212
**LOC1001284**	0.44423	-0.23759	-0.1402531061668240	8214
**GRAMD4**	0.44424	-1.2173	-0.140227792143246000	8215
**COL8A2**	0.44442	-0.18453	-0.139772155067264000	8216
**ATPAF2**	0.55553	-1.1114	-0.1396455943660910	8217
**ADCY5**	0.44451	-1.1206	-0.139544347415620000	8218
**HIPK2**	0.44451	-2.1975	-0.139544347415620000	8223
**MGST2**	0.44451	-2.5017	-0.139544347415620000	8224
**NSMCE1**	0.44451	-1.6068	-0.139544347415620000	8222

**SIX2**	0.44451	-1.2983	-0.13954434741562000	8219
**TNFRSF10D**	0.44451	-1.3973	-0.13954434741562000	8221
**UNC13A**	0.44451	-1.3631	-0.13954434741562000	8220
**PATL2**	0.44457	-0.20882	-0.13939247967149800	8225
**MCM4**	0.4447	-2.2807	-0.1390634439158150	8226
**TLL2**	0.44481	-0.99198	-0.1387850408053250	8227
**OR9G1**	0.44487	-0.26557	-0.13863318909817700	8228
**KIZ**	0.4451	-1.4185	-0.13805112046753500	8229
**MZT1**	0.55488	-0.088233	-0.1380005080170530	8230
**HIVEP2**	0.44516	-0.61937	-0.13789928417640200	8231
**ZBTB48**	0.5548	-0.69172	-0.1377980617486050	8232
**MTA3**	0.55476	-0.88401	-0.13769684073258700	8233
**TMEM45B**	0.44524	-0.89633	-0.1376968407325870	8234
**HNRNPA3**	0.44528	-1.2883	-0.13759562112727100	8235
**HDHD2**	0.44531	-1.8336	-0.13751970734841000	8236
**POC5**	0.4454	-0.49978	-0.1372919707647750	8237
**COQ2**	0.44555	-0.62	-0.13691242560798400	8238
**BROX**	0.44557	-0.12083	-0.1368618210783290	8239
**MMP14**	0.44561	-0.62521	-0.13676061307025600	8240
**N4BP2L1**	0.4458	-0.3673	-0.13627989414258400	8241
**GP2**	0.55412	-0.75097	-0.13607749560030300	8242
**ITIH1**	0.44589	-0.23593	-0.1360521961746160	8243
**AP1M2**	0.44594	-1.1864	-0.13592570035199400	8245
**SPINT2**	0.44594	-0.35328	-0.13592570035199400	8244
**PXDC1**	0.44597	-0.48357	-0.13584980390247000	8246
**LIN54**	0.446	-0.67589	-0.13577390823542800	8247
**INPP5E**	0.55375	-0.34584	-0.13514147471259700	8248
**ECH1**	0.44656	-0.41641	-0.13435733217348500	8249
**PHYKPL**	0.44666	-0.3336	-0.13410440061172100	8250
**TIE1**	0.44679	-1.2542	-0.13377560240382400	8251
**PIGU**	0.5531	-0.05862	-0.1334973998319870	8252
**ESCO2**	0.44691	-0.58756	-0.13347210920172700	8253

**HINT3**	0.44702	-0.30993	-0.133193917899371	8255
**HOXA11**	0.44702	-1.7404	-0.133193917899371	8259
**MAL2**	0.44702	-0.97395	-0.133193917899371	8257
**RBM46**	0.44702	-1.8076	-0.133193917899371	8260
**RPA4**	0.44702	-0.89387	-0.133193917899371	8256
**SGMS1**	0.44702	-1.8662	-0.133193917899371	8261
**SLC26A3**	0.44702	-1.6843	-0.133193917899371	8258
**SOX2**	0.44702	-1.9881	-0.133193917899371	8262
**ZNF843**	0.44702	-0.17662	-0.133193917899371	8254
**MAGEB3**	0.55296	-0.98348	-0.1331433387706010	8263
**NDUFC2-KC**	0.44711	-1.863	-0.1329663145012170	8264
**NBPF9**	0.55269	-0.086562	-0.1324605538133520	8265
**RTN4R**	0.44741	-0.83149	-0.1322076861823200	8266
**CHM**	0.55252	-0.5435	-0.1320306838711020	8267
**FAM84B**	0.4475	-0.58349	-0.1319801125421460	8268
**ADAM33**	0.44752	-1.1158	-0.1319295415507070	8269
**RFNG**	0.44757	-0.51933	-0.1318031155478620	8270
**LLGL2**	0.5524	-0.1028	-0.1317272609574330	8271
**LOC643936**	0.4476	-0.59993	-0.1317272609574330	8272
**OR12D3**	0.44766	-1.6352	-0.1315755540498420	8273
**TMCC3**	0.44767	-1.2353	-0.1315502698597310	8274
**MTCH2**	0.44773	-0.83247	-0.1313985664844710	8275
**RARRES2**	0.55225	-1.2033	-0.1313479993648530	8276
**MED1**	0.44777	-0.31566	-0.131297432581078	8277
**IL7**	0.44786	-0.29323	-0.1310698862065770	8278
**ZNF648**	0.55204	-0.42898	-0.1308170648598800	8279
**CGNL1**	0.44802	-0.99525	-0.1306653760662170	8280
**CYP2C8**	0.55194	-0.79161	-0.1305642518741940	8281
**DZIP3**	0.44807	-1.1127	-0.1305389710348380	8282
**ADCY8**	0.44809	-0.73061	-0.1304884096063880	8283
**UGT1A1**	0.5519	-0.034561	-0.1304631290172610	8284
**PHF12**	0.44836	-1.2468	-0.1298058629161680	8285

**GREM2**	0.55161	-1.0935	-0.1297300281334380	8286
**TLR8**	0.44853	-0.94287	-0.129376142337569	8287
**ADAP2**	0.55119	-0.52105	-0.1286684193032070(	8288
**ACTRT3**	0.44908	-1.0109	-0.1279860331227380(	8289
**NR2C2AP**	0.44919	-0.70908	-0.1277080410406110(	8290
**HOXC5**	0.55074	-1.146	-0.1275311421279020(	8291
**MSMP**	0.44927	-0.40666	-0.1275058711805560(	8292
**PRDM12**	0.44937	-0.35564	-0.1272531661827140(	8293
**BPNT1**	0.55061	-0.84715	-0.1272026261587770	8294
**GAK**	0.44942	-0.87904	-0.1271268167320150(	8295
**NLRP7**	0.44943	-1.5796	-0.1271015470854660(	8296
**NXPE1**	0.44943	-1.657	-0.1271015470854660(	8297
**PRB2**	0.44943	-2.1366	-0.1271015470854660(	8299
**TTLL6**	0.44943	-1.8642	-0.1271015470854660(	8298
**FAM83A**	0.44947	-1.9075	-0.1270004693106890(	8300
**TARS2**	0.4496	-0.53373	-0.1266719754977680(	8301
**NAV2**	0.44961	-1.1798	-0.1266467073095210(	8302
**RPS6KC1**	0.55038	-0.94486	-0.1266214392021320(	8303
**GUCY1A3**	0.44963	-0.39848	-0.1265961711755850(	8304
**OSTM1**	0.5502	-1.0699	-0.126166627076977	8305
**TNFRSF11B**	0.44985	-0.71967	-0.1260402950108790(	8306
**NOL4**	0.44997	-0.26581	-0.1257371062551230(	8307
**LACRT**	0.45005	-0.19914	-0.1255349868404940(	8308
**DUSP7**	0.45008	-0.80785	-0.1254591933825540	8309
**TAF9**	0.45032	-0.003756	-0.1248528716261670(	8310
**RALYL**	0.45034	-1.1276	-0.1248023468879230	8311
**ZNF786**	0.45039	-0.76714	-0.1246760364358040(	8312
**DPP7**	0.45042	-0.51712	-0.1246002511194020(	8313
**ANAPC1**	0.45062	-0.23066	-0.1240950339427220(	8314
**MEF2C**	0.54937	-0.097395	-0.1240697739163760(	8315
**CCNA1**	0.45068	-1.0762	-0.1239434749717510(	8316
**EZR**	0.54899	-0.99753	-0.1231099514230130(	8318

**SULT1A2**	0.54899	-1.4243	-0.12310995142301300	8319
**VKORC1L1**	0.54899	-0.087185	-0.12310995142301300	8317
**OLFM1**	0.45102	-0.73876	-0.12308469447170900	8320
**ZNF337**	0.5488	-0.21413	-0.12263008275826300	8321
**S100A2**	0.4513	-0.73841	-0.1223775316517620	8322
**HNRNPK**	0.45139	-1.1575	-0.12215024232972500	8323
**CLLU1OS**	0.54859	-0.54765	-0.12209973444883800	8324
**FOXRED1**	0.54852	-0.7021	-0.12192295931771600	8325
**SLC25A11**	0.54846	-0.69439	-0.12177144080931000	8326
**PALM**	0.45157	-0.83617	-0.1216956826036450	8328
**POTEJ**	0.54843	-0.24757	-0.1216956826036450	8327
**APBA2**	0.45159	-0.50388	-0.12164517752124600	8329
**C11orf40**	0.45176	-1.7793	-0.12121589683502200	8331
**KRT16**	0.45176	-2.091	-0.12121589683502200	8332
**MLX**	0.45176	-1.7359	-0.12121589683502200	8330
**SSX3**	0.45176	-2.104	-0.12121589683502200	8333
**TEX14**	0.54813	-0.63312	-0.12093813888673600	8334
**DPEP3**	0.45193	-0.13882	-0.12078663848512600	8335
**FASN**	0.54781	-0.54123	-0.12013016873369300	8336
**ZNF705B**	0.45221	-1.303	-0.12007967320835000	8337
**KIFC2**	0.45227	-0.81898	-0.11992818846882400	8338
**TMEM192**	0.45231	-0.27594	-0.11982720017157000	8339
**ANKRD20A3**	0.54764	-0.44409	-0.11970096651837700	8340
**SPG7**	0.54755	-0.98393	-0.11947375074782000	8341
**CD38**	0.54746	-0.095754	-0.11924654114508900	8342
**BCCIP**	0.54741	-0.29178	-0.11912031624868800	8343
**HNRNPDL**	0.45262	-1.5169	-0.11904458222195400	8344
**TTC9C**	0.45273	-0.79176	-0.11876689662878900	8345
**RPS6KA5**	0.5472	-0.47912	-0.1185901923843720	8346
**CPSF4L**	0.45282	-0.76006	-0.11853970613774900	8347
**MFGE8**	0.45284	-1.0129	-0.1184892201932620	8348
**GALNT1**	0.45288	-0.51809	-0.11838824921017000	8349

**PPA1**	0.45317	-2.1908	-0.11765624561249000	8350
**CTTNBP2**	0.54681	-0.37196	-0.11760576493399300	8351
**PTPN9**	0.45327	-0.48642	-0.11740384521559300	8352
**SFXN2**	0.45328	-0.28086	-0.11737860558752500	8353
**SLC33A1**	0.5467	-0.45472	-0.11732812655569400	8354
**NUDT7**	0.54669	-0.3472	-0.11730288715189800	8355
**B4GALNT2**	0.45336	-1.2087	-0.11717669125345800	8356
**TMEM168**	0.54652	-0.22279	-0.116873828706758	8357
**KLHL33**	0.4535	-1.1837	-0.11682335265862700	8358
**MNT**	0.54646	-0.73466	-0.11672240145516200	8359
**MRRF**	0.54644	-0.18159	-0.1166719262995650	8360
**ZNF691**	0.45367	-0.72581	-0.11639431825353900	8361
**LRRC73**	0.54631	-0.59929	-0.116343845027691	8362
**ZNF460**	0.45369	-0.7081	-0.11634384502769000	8363
**TRPM2**	0.4537	-0.81696	-0.1163186085259210	8364
**ITIH5**	0.45375	-0.97798	-0.11619242712795300	8365
**HLA-C**	0.54617	-0.92479	-0.11599054073825900	8366
**SNX8**	0.45383	-1.0897	-0.11599054073825800	8367
**KANSL1**	0.54609	-0.22923	-0.11578865907601000	8368
**C7orf57**	0.45398	-0.89109	-0.11561201649288700	8370
**ENTPD8**	0.54602	-0.8478	-0.11561201649288700	8369
**OR51G2**	0.54602	-1.6608	-0.11561201649288700	8372
**SNED1**	0.54602	-1.0063	-0.11561201649288700	8371
**MSL2**	0.45399	-0.15145	-0.11558678213276400	8373
**FAM174A**	0.45405	-1.7	-0.11543537751709300	8374
**ATP2B2**	0.45408	-0.13942	-0.11535967620177800	8375
**AIRE**	0.45412	-1.2555	-0.11525874214297100	8376
**GJD3**	0.45419	-2.0673	-0.11508211036486600	8381
**GTF2A2**	0.45419	-2.0044	-0.11508211036486600	8380
**IPP**	0.45419	-1.8708	-0.11508211036486600	8379
**MTX2**	0.45419	-1.2893	-0.11508211036486600	8377
**PLD2**	0.45419	-1.7888	-0.11508211036486600	8378

**RNF186**	0.45419	-2.3438	-0.1150821103648660(	8382
**PJA1**	0.45429	-0.30144	-0.1148297854800690(	8383
**GGT2**	0.45432	-0.52758	-0.1147540894409830(	8384
**ABCC8**	0.45457	-1.6415	-0.1141233146434380(	8385
**ETNK2**	0.54539	-0.4958	-0.1140223948960200(	8386
**CEACAM1**	0.54532	-0.94397	-0.1138457881317610(	8387
**ZGLP1**	0.4548	-0.74311	-0.1135430419366750(	8388
**CLEC9A**	0.54515	-0.46035	-0.1134169007617380(	8389
**ATP2A3**	0.45486	-0.655	-0.1133916727433730(	8390
**COX7A1**	0.54509	-0.70059	-0.1132655337337540(	8391
**GLB1**	0.45495	-0.089991	-0.1131646238237200(	8392
**TGFB1**	0.45521	-1.2748	-0.1125087374495850(	8394
**ZNF521**	0.45521	-0.78073	-0.1125087374495850(	8393
**GTPBP6**	0.45541	-0.52422	-0.1120042424174500(	8395
**RBFOX3**	0.45541	-1.8666	-0.1120042424174500(	8396
**IZUMO1**	0.45547	-0.30395	-0.1118528994724010(	8397
**PKN3**	0.54449	-1.5664	-0.1117520055992310(	8400
**TPO**	0.54449	-0.7241	-0.1117520055992310(	8399
**ZNF79**	0.54449	-0.46866	-0.1117520055992310(	8398
**RPL7A**	0.45556	-0.24361	-0.1116258898573900(	8401
**RTL8B**	0.45562	-0.82659	-0.1114745533105780(	8402
**ROS1**	0.45565	-0.26155	-0.1113988859948000(	8403
**ATP1A1**	0.45575	-0.73062	-0.1111466662129050(	8404
**BTN2A1**	0.45577	-1.267	-0.1110962231055150(	8405
**CTCF**	0.54409	-0.20167	-0.1107431292617360(	8406
**TRPC4AP**	0.54387	-0.45485	-0.1101882953850740(	8407
**NDUFB3**	0.4562	-0.33128	-0.1100117644549720(	8408
**INTS9**	0.54372	-0.49486	-0.1098100190182100(	8409
**GAS2**	0.4563	-0.7843	-0.1097595833576960(	8410
**DUSP18**	0.45631	-1.3149	-0.1097343656321500(	8411
**LYAR**	0.54364	-1.0784	-0.1096082780508760(	8412
**RASGEF1A**	0.45637	-0.34707	-0.1095830607437980(	8413

**C5orf46**	0.54342	-0.52761	-0.1090535133675890	8414
**ADAMDEC1**	0.45665	-0.58046	-0.1088770043785880	8415
**DMAP1**	0.45665	-2.0268	-0.1088770043785880	8419
**DNMT3A**	0.45665	-1.7066	-0.1088770043785880	8417
**EPX**	0.45665	-2.2853	-0.1088770043785880	8420
**ESRP2**	0.45665	-1.7916	-0.1088770043785880	8418
**F2RL1**	0.45665	-1.656	-0.1088770043785880	8416
**SYT12**	0.45665	-2.3934	-0.1088770043785880	8421
**MARVELD1**	0.54321	-0.49099	-0.1085239965714240	8422
**PHLDB1**	0.45682	-0.32943	-0.1084483538023010	8423
**OLFM4**	0.45683	-0.28561	-0.1084231396838470	8424
**ZSWIM8**	0.54315	-0.26359	-0.1083727116537170	8425
**ADAL**	0.45691	-0.64045	-0.1082214292163980	8426
**RND1**	0.45697	-0.88653	-0.1080701492559240	8427
**TC2N**	0.45702	-0.33422	-0.1079440845116760	8428
**MSANTD3**	0.45711	-0.97462	-0.1077171722937710	8429
**RALBP1**	0.54239	-0.55931	-0.1064566495289600	8430
**LRRN2**	0.45771	-0.70894	-0.1062045653378120	8432
**VGLL1**	0.45771	-0.6135	-0.1062045653378120	8431
**ZFPM2**	0.45778	-0.66509	-0.1060281104206760	8433
**HNRNPH1**	0.45779	-0.87156	-0.1060029028450050	8434
**XKR6**	0.45782	-0.09529	-0.1059272805220870	8435
**DNM1L**	0.45786	-1.0391	-0.1058264517004680	8436
**GLRX3**	0.45788	-1.9392	-0.1057760376931940	8437
**ADM**	0.54205	-0.33321	-0.1055995907840680	8438
**OTUD6A**	0.45795	-0.66095	-0.1055995907840670	8439
**TAB2**	0.458	-1.6047	-0.1054735592908100	8440
**RAB18**	0.45801	-0.86096	-0.1054483531932720	8441
**CEP83**	0.54192	-0.4992	-0.1052719123855790	8442
**ORMDL3**	0.45814	-0.2174	-0.1051206800162900	8443
**NEPRO**	0.54166	-0.25535	-0.1046165894634250	8444
**AP1M1**	0.54163	-1.6306	-0.1045409781765580	8447

**TGFB2**	0.54163	-1.1047	-0.1045409781765580	8446
**ZNF829**	0.45837	-0.32835	-0.1045409781765580(	8445
**MYL7**	0.45839	-0.14563	-0.1044905709840620(	8448
**PPFIA4**	0.45858	-1.8409	-0.1040117158793000(	8449
**TMEM97**	0.4586	-0.27305	-0.1039613114681620(	8450
**C9orf84**	0.4588	-0.41557	-0.1034572818628790(	8452
**DTX2**	0.4588	-0.47915	-0.1034572818628790(	8453
**LRRC75A**	0.4588	-1.8798	-0.1034572818628790(	8455
**SYNJ2**	0.4588	-1.6919	-0.1034572818628790(	8454
**ZNF616**	0.4588	-0.28145	-0.1034572818628790(	8451
**PAF1**	0.45883	-0.38544	-0.1033816796922070(	8456
**ZNF614**	0.54098	-0.55228	-0.1029028796491530(	8457
**TRPM8**	0.54093	-0.94416	-0.1027768835633170(	8458
**TROAP**	0.54091	-0.99883	-0.1027264855859670	8459
**SCAMP2**	0.45911	-0.43388	-0.1026760878695530	8460
**COPG1**	0.54074	-0.29273	-0.1022981133006530(	8461
**ZNF35**	0.45939	-0.067336	-0.1019705471650630(	8462
**CCDC94**	0.54034	-0.87066	-0.1012902524198290(	8463
**LILRA1**	0.54034	-1.3621	-0.1012902524198290(	8464
**SLC6A1**	0.54034	-1.6069	-0.1012902524198290(	8465
**SMOC1**	0.54034	-1.8273	-0.1012902524198290(	8466
**ACBD4**	0.45971	-0.20753	-0.1011642770653520(	8467
**AHCY**	0.45973	-1.163	-0.1011138873732330(	8468
**PI4K2A**	0.5401	-0.91608	-0.1006855853437310(	8469
**SLC38A1**	0.4599	-1.0628	-0.1006855853437310(	8470
**BOLA3**	0.54004	-0.96055	-0.1005344243356560(	8471
**MEP1A**	0.45997	-1.1137	-0.1005092310577580(	8472
**TRIM3**	0.46008	-0.49868	-0.1002321092079940(	8473
**DRC7**	0.53988	-0.45549	-0.1001313395359800(	8474
**APLN**	0.46014	-1.1825	-0.1000809550813630(	8475
**ALKAL2**	0.53976	-0.77697	-0.0998290366169595(	8476
**PCYT2**	0.53968	-0.37804	-0.0996275064080313(	8477

**IFT140**	0.53957	-1.3257	-0.0993504089763950(	8478
**PAQR6**	0.53956	-0.5575	-0.0993252186794117(	8479
**POC1B-GAL**	0.46048	-0.44803	-0.0992244581216407(	8480
**NUB1**	0.46069	-0.26046	-0.0986954816980885(	8481
**CDC25B**	0.46076	-0.65127	-0.0985191623683197(	8482
**C5orf22**	0.53922	-1.9125	-0.0984687859798050(	8486
**FREM3**	0.53922	-0.28152	-0.0984687859798050(	8483
**KRTAP4-6**	0.53922	-1.1667	-0.0984687859798050(	8485
**NCOR2**	0.53922	-0.62732	-0.0984687859798050(	8484
**PHOSPHO2**	0.53916	-0.37969	-0.0983176583132375	8487
**PYROXD2**	0.46085	-0.72801	-0.0982924705872389(	8488
**FRMD4A**	0.46088	-0.19096	-0.0982169077833693(	8489
**EXOSC4**	0.46089	-2.1551	-0.0981917203067332(	8492
**LAMC2**	0.46089	-1.8239	-0.0981917203067332(	8491
**SIGIRR**	0.46089	-1.1019	-0.0981917203067332(	8490
**TNRC6A**	0.46089	-2.3798	-0.0981917203067332(	8493
**GRIA2**	0.46092	-0.84507	-0.0981161582505600(	8494
**S100A14**	0.46098	-0.78618	-0.0979650358185545(	8495
**ADAMTS4**	0.53882	-0.068952	-0.0974613105186928(	8496
**MPL**	0.46119	-0.93834	-0.0974361249039762	8497
**GHITM**	0.46123	-1.1222	-0.0973353830630648	8498
**TIFAB**	0.53871	-0.97421	-0.0971842721537726(	8499
**BMP10**	0.46139	-0.6014	-0.0969324255684799(	8500
**TSPAN17**	0.53859	-0.38627	-0.0968820569897795(	8501
**TRIP11**	0.53845	-1.1422	-0.0965294838144342(	8502
**AADACL2**	0.53842	-0.38424	-0.0964539339825082(	8504
**ATP13A1**	0.46158	-0.18595	-0.0964539339825082(	8503
**SULT2A1**	0.53823	-0.45898	-0.0959754644816302(	8505
**PRODH**	0.46182	-0.52626	-0.0958495551117565(	8506
**PIK3AP1**	0.46191	-0.56257	-0.0956229220742292(	8507
**IL16**	0.46195	-2.1756	-0.0955221978571475(	8508
**ID4**	0.53791	-1.4084	-0.0951696707231446(	8510

**LYPD6**	0.53791	-1.319	-0.0951696707231446(	8509
**SP140L**	0.53791	-1.6762	-0.0951696707231446(	8511
**FGF12**	0.46215	-0.14478	-0.0950185912892412(	8512
**GFI1**	0.46231	-0.44779	-0.0946157233935418(	8513
**CCDC172**	0.46235	-0.89127	-0.0945150088218753(	8514
**TNFRSF11A**	0.46242	-0.43158	-0.0943387606280083(	8515
**PBX3**	0.46244	-0.75378	-0.0942884045397484(	8516
**NEU1**	0.53748	-0.55853	-0.0940869825766302(	8517
**CDC42SE2**	0.46259	-0.91969	-0.0939107414908436(	8518
**IFT57**	0.46263	-0.35475	-0.0938100336091362(	8519
**S100A16**	0.46263	-1.1249	-0.0938100336091362(	8521
**SERPINA10**	0.46263	-0.70292	-0.0938100336091362(	8520
**C17orf97**	0.46268	-0.32332	-0.0936841500950023(	8522
**ANKH**	0.46273	-0.043539	-0.0935582680656424(	8523
**RPL24**	0.5372	-0.20677	-0.0933820357150952(	8524
**AQP7**	0.53705	-0.35163	-0.0930044047241679(	8525
**CENPE**	0.53703	-0.10869	-0.0929540549287719(	8526
**HECW1**	0.46306	-1.079	-0.0927274837641818(	8527
**MGST1**	0.46329	-0.63863	-0.0921484901669648(	8528
**CIB4**	0.4633	-1.2102	-0.0921233172345495(	8530
**CPTP**	0.4633	-0.97625	-0.0921233172345495(	8529
**FAM219B**	0.4633	-2.3722	-0.0921233172345495(	8532
**NUS1**	0.4633	-2.4477	-0.0921233172345495(	8533
**PRAF2**	0.4633	-1.5809	-0.0921233172345495(	8531
**CYSRT1**	0.53657	-1.9113	-0.0917960744203205(	8534
**IGSF3**	0.46366	-0.50106	-0.0912171304263413(	8535
**ITGB5**	0.46378	-1.0805	-0.0909150848412540(	8536
**MOB1B**	0.53617	-0.43824	-0.0907892349640651(	8537
**MFHAS1**	0.46383	-0.51702	-0.0907892349640650(	8538
**ARL17B**	0.53614	-0.99561	-0.0907137257282016(	8539
**COL4A3**	0.46392	-0.97491	-0.0905627088079440(	8540
**FAM92B**	0.46392	-2.2207	-0.0905627088079440(	8541

**SLC35B1**	0.464	-0.55755	-0.0903613561269936	8542
**OR8D1**	0.53598	-0.29586	-0.0903110185295930	8543
**ZNF621**	0.53598	-0.67701	-0.0903110185295930	8544
**CALCOCO1**	0.46403	-0.55754	-0.0902858498167302	8545
**MINDY1**	0.53596	-0.5971	-0.0902606811610693	8546
**AKAP14**	0.53584	-0.25516	-0.0899586617490648	8547
**TESK1**	0.46417	-0.1247	-0.0899334938355671	8548
**CLEC4G**	0.46426	-1.9747	-0.0897069851760713	8549
**ZNF418**	0.46432	-0.55249	-0.0895559819611748	8550
**HOMER3**	0.46438	-0.17231	-0.0894049807886773	8551
**TCP10L2**	0.46443	-1.4504	-0.0892791480359109	8552
**FAM104B**	0.46449	-1.9916	-0.0891281505987389	8553
**ODAM**	0.46452	-1.4701	-0.0890526526425577	8554
**LIPC**	0.46453	-0.74688	-0.0890274867700059	8555
**NUP107**	0.53536	-0.71466	-0.0887506658903462	8556
**CIT**	0.46484	-0.27003	-0.0882473726118629	8557
**EGR3**	0.46495	-0.68343	-0.0879705708465888	8558
**SLC39A7**	0.46496	-0.61363	-0.0879454073843554	8559
**ERN 2.00**	0.46504	-0.24623	-0.0877441016902610	8560
**C1orf123**	0.53494	-1.559	-0.0876937758227498	8562
**LRIT3**	0.53494	-1.3869	-0.0876937758227498	8561
**TBC1D19**	0.46509	-0.64493	-0.0876182874379658	8563
**PRSS27**	0.46512	-0.97242	-0.0875427995525746	8564
**SLC48A1**	0.46515	-0.29818	-0.0874673121661387	8565
**SLC8A2**	0.46554	-0.3873	-0.0864860213489332	8566
**OR10H1**	0.53431	-1.082	-0.0861086240343749	8567
**P4HTM**	0.46575	-0.68696	-0.0859576685462804	8568
**CFAP74**	0.46583	-0.080529	-0.0857563976091724	8569
**SEC11C**	0.46586	-0.39124	-0.0856809219040299	8570
**RAB40B**	0.53404	-0.76107	-0.0854293397436448	8571
**GNA11**	0.46598	-1.8769	-0.0853790239610777	8578
**HIST1H2AB**	0.46598	-1.9498	-0.0853790239610777	8580

**KIAA0319**	0.46598	-1.7468	-0.0853790239610777(	8575
**KLK8**	0.46598	-0.26928	-0.0853790239610777(	8572
**MGAM**	0.46598	-1.1161	-0.0853790239610777(	8574
**MORF4L2**	0.46598	-1.7835	-0.0853790239610777(	8577
**PRL**	0.46598	-1.7594	-0.0853790239610777(	8576
**RIT2**	0.46598	-2.3461	-0.0853790239610777(	8581
**TPRA1**	0.46598	-1.9364	-0.0853790239610777(	8579
**ZNF280B**	0.46598	-1.0598	-0.0853790239610777(	8573
**SPATA31D1**	0.53398	-0.66917	-0.0852783930444193(	8582
**PTPRA**	0.46616	-2.1158	-0.0849261916315652(	8583
**CTF1**	0.46623	-0.22597	-0.0847500948792647	8584
**ZNF454**	0.46625	-0.64962	-0.0846997820045296(	8585
**USP50**	0.46627	-1.5239	-0.0846494693442545(	8586
**PTGDR2**	0.53373	-0.58184	-0.0846494693442543(	8587
**SUCLA2**	0.46645	-0.41414	-0.0841966650311365(	8588
**MED12**	0.4666	-1.3137	-0.0838193412973334(	8591
**NCAN**	0.5334	-1.265	-0.0838193412973334(	8590
**ZNF184**	0.5334	-0.55871	-0.0838193412973334(	8589
**RSRP1**	0.46675	-0.93645	-0.0834420294998308(	8592
**GPX4**	0.533	-0.40305	-0.0828132028813048	8593
**CDHR4**	0.467	-0.35873	-0.0828132028813045	8594
**MRPS17**	0.53297	-0.48791	-0.0827377458930715(	8595
**DBNDD2**	0.46717	-1.3059	-0.0823856195047727(	8596
**RNF5**	0.46733	-0.74906	-0.0819832018556839(	8597
**SLC22A31**	0.46736	-1.1317	-0.0819077500268690(	8598
**ZNF806**	0.46752	-1.2698	-0.0815053481400341(	8599
**CRABP1**	0.46753	-1.1023	-0.0814801984611165(	8600
**HOXA13**	0.46769	-1.3758	-0.0810778105984960(	8601
**LELP1**	0.46775	-0.42244	-0.0809269185389549(	8602
**CLEC6A**	0.53207	-2.001	-0.0804742534050483(	8606
**COL11A1**	0.53207	-1.3646	-0.0804742534050483(	8603
**CSF1R**	0.53207	-1.5928	-0.0804742534050483(	8604

**TMEM159**	0.53207	-1.6512	-0.08047425340504830	8605
**ZNF579**	0.46809	-0.69111	-0.08007189824691920	8607
**ERCC6L**	0.46811	-1.3702	-0.0800216047652069	8608
**RAB40C**	0.46817	-0.55495	-0.07987072553438630	8609
**DTHD1**	0.46825	-0.76369	-0.07966955605522350	8610
**HIST1H2BL**	0.46832	-1.1447	-0.07949353540685570	8611
**PBLD**	0.46844	-0.54422	-0.0791917914528156	8612
**TRIM13**	0.4685	-0.35465	-0.0790409221822364	8613
**SLC3A2**	0.46855	-0.33332	-0.07891519916497600	8614
**KRTAP5-6**	0.46856	-0.87689	-0.07889005471131920	8615
**OTUD3**	0.46862	-0.18585	-0.07873918903659090	8616
**RAB38**	0.46872	-0.19889	-0.07848775022747480	8617
**PPM1K**	0.46895	-2.0791	-0.07790945977727630	8618
**TUBAL3**	0.46902	-1.3628	-0.07773346351796850	8619
**TTC14**	0.46908	-0.18105	-0.07758261149866120	8620
**POU2F1**	0.53078	-1.3787	-0.07723063031577760	8623
**SEMA4B**	0.53078	-1.6197	-0.07723063031577760	8624
**TCF20**	0.53078	-0.18115	-0.07723063031577760	8621
**TMEM35B**	0.53078	-0.764	-0.07723063031577760	8622
**ZNF26**	0.46961	-0.85008	-0.07625016163728330	8625
**CTSG**	0.46971	-0.26044	-0.07599877126078270	8626
**WDPCP**	0.46978	-1.423	-0.07582280085695480	8627
**PIGO**	0.46985	-0.95923	-0.07564683280189720	8628
**C2CD4D**	0.53012	-0.88488	-0.07557141863880650	8629
**PKD1**	0.53006	-0.12958	-0.07542059160207760	8630
**KLF11**	0.47014	-1.0077	-0.07491784718961350	8631
**SNURF**	0.52964	-0.19697	-0.0743648502096148	8632
**FAM209B**	0.47041	-1.2725	-0.07423917225666670	8633
**PROK1**	0.47052	-0.41911	-0.07396268488660570	8634
**NPIPB4**	0.52944	-0.55253	-0.07386214542829380	8635
**TMEM221**	0.47061	-0.71656	-0.07373647215564520	8636
**MTA2**	0.52938	-2.1262	-0.07371133764098690	8640

**PLA2G12A**	0.52938	-1.6988	-0.0737113376409869(	8639
**ZBTB4**	0.52938	-1.6475	-0.0737113376409869(	8638
**ZCCHC6**	0.52938	-1.1698	-0.0737113376409869(	8637
**ASCC1**	0.47072	-0.42318	-0.0734599505402050(	8642
**MIER3**	0.47072	-0.34447	-0.0734599505402050(	8641
**ARL 15.00**	0.47074	-0.71874	-0.0734097270942551(	8643
**SLC25A47**	0.52919	-0.87754	-0.0732337906956469(	8644
**TMC2**	0.47088	-1.872	-0.0730578565648531	8645
**CTSH**	0.47092	-1.7396	-0.0729573237919139(	8649
**KAZALD1**	0.47092	-1.6156	-0.0729573237919139(	8648
**PRR19**	0.47092	-1.5173	-0.0729573237919139(	8647
**RPL14**	0.47092	-1.9715	-0.0729573237919139(	8652
**SLC30A7**	0.47092	-1.8398	-0.0729573237919139(	8651
**SLC8A3**	0.47092	-1.8263	-0.0729573237919139(	8650
**SPATA7**	0.47092	-0.95675	-0.0729573237919139(	8646
**PROK2**	0.47103	-0.67861	-0.0726808624669583(	8653
**MPDU1**	0.47108	-0.78382	-0.0725552000667965(	8654
**CEP72**	0.52883	-0.04177	-0.0723290106336589(	8655
**ZBED5**	0.47123	-0.20723	-0.0721782197356787(	8656
**KRT71**	0.52866	-0.56551	-0.0719017740179796(	8657
**AFMID**	0.47139	-0.19653	-0.0717761186921580(	8658
**C1orf141**	0.5286	-0.19901	-0.0717509877631153(	8659
**FGR**	0.52857	-0.35414	-0.0716755952479421(	8660
**WFDC12**	0.4715	-0.54357	-0.0714996809635551(	8661
**SYT14**	0.52844	-0.54835	-0.0713488990533099(	8662
**CCDC13**	0.47166	-1.4846	-0.0710975994742818(	8663
**CDIPT**	0.5283	-0.25909	-0.0709970809013317(	8664
**XRCC5**	0.47172	-0.65259	-0.0709468218840574(	8665
**IGSF22**	0.47176	-0.94582	-0.0708463043872695(	8666
**TLE3**	0.47177	-0.65315	-0.0708211751250121(	8667
**INPP5B**	0.5282	-1.2706	-0.0707457876066420(	8670
**PTCH1**	0.5282	-1.2636	-0.0707457876066420(	8669

**VSTM2B**	0.5282	-0.37326	-0.0707457876066420(	8668
**ANKRD7**	0.47181	-1.1793	-0.0707206585232645(	8671
**MYT1L**	0.52815	-0.70264	-0.0706201426363979	8672
**GINS4**	0.47188	-0.82298	-0.0705447561897706(	8673
**TOR1B**	0.52808	-0.81148	-0.0704442415514785(	8674
**SLC24A2**	0.52801	-0.58105	-0.0702683426472503(	8675
**NFASC**	0.47207	-0.45762	-0.0700673179913258(	8676
**OR2A42**	0.52791	-0.018336	-0.070017062270303	8677
**CPA1**	0.47211	-0.4365	-0.0699668067262078(	8678
**KCNC4**	0.52774	-0.37597	-0.0695898957721290(	8679
**IL22RA2**	0.52752	-0.8371	-0.0690371109159333(	8680
**HNRNPH2**	0.47265	-0.46393	-0.0686099734464498(	8681
**MRPL42**	0.52733	-0.20036	-0.0685597228043544(	8682
**CHMP2A**	0.47273	-0.64066	-0.0684089719168536	8683
**SERPINB1**	0.47275	-0.58712	-0.0683587219668554(	8684
**GCSAML**	0.47277	-0.01976	-0.0683084721895607(	8685
**ZFP42**	0.52709	-0.79853	-0.0679567285734779(	8686
**DAD1**	0.52696	-1.8943	-0.0676301170348444(	8690
**POPDC3**	0.52696	-1.7275	-0.0676301170348444(	8689
**SFXN1**	0.52696	-1.6082	-0.0676301170348444(	8688
**SRRM1**	0.52696	-1.4114	-0.0676301170348444(	8687
**SLC38A5**	0.47318	-0.54038	-0.0672783896033505	8691
**CCL4L2**	0.47319	-1.5163	-0.0672532665345720(	8693
**OR6N1**	0.47319	-1.2858	-0.0672532665345720(	8692
**SIGLEC6**	0.47319	-1.5791	-0.0672532665345720(	8694
**TMEM120A**	0.47319	-1.853	-0.0672532665345720(	8696
**TMEM217**	0.47319	-1.6898	-0.0672532665345720(	8695
**ZNF555**	0.47321	-2.4169	-0.0672030205244160(	8697
**ESM1**	0.47334	-0.60516	-0.0668764255906647(	8698
**MTMR9**	0.47344	-0.14198	-0.0666252035771849(	8699
**PSMC3**	0.47345	-0.022267	-0.0666000816075038(	8700
**TMEM107**	0.52651	-0.53319	-0.0664995941491916	8701

**UBL4A**	0.4736	-1.5885	-0.0662232571002126(	8702
**RGS11**	0.47373	-0.9043	-0.0658966834733173(	8703
**TMEM115**	0.52617	-0.63267	-0.065645477780829(	8704
**B3GALT2**	0.47386	-0.96384	-0.0655701168785216(	8705
**RPRD1B**	0.47392	-0.32733	-0.0654193961968325(	8706
**DEFB130**	0.47394	-0.17675	-0.0653691563002266(	8707
**ABI1**	0.47397	-0.69472	-0.0652937967648380(	8708
**PAICS**	0.47402	-0.77396	-0.0651681983635135(	8709
**HDC**	0.47404	-0.34166	-0.0651179592911247	8710
**GATA4**	0.47418	-0.10227	-0.0647662903821370(	8711
**GRPEL2**	0.52578	-0.22542	-0.0646658150250101(	8712
**RNF183**	0.52578	-1.4825	-0.0646658150250101(	8714
**SSBP4**	0.52578	-0.7022	-0.0646658150250101(	8713
**CD82**	0.52577	-0.45942	-0.0646406962878368(	8715
**KLRC1**	0.47425	-0.6713	-0.0645904589359116(	8716
**PRAME**	0.47441	-0.593	-0.0641885659820798(	8717
**NKRF**	0.47447	-0.60713	-0.0640378588025216(	8718
**ATP5G1**	0.47448	-0.90074	-0.0640127410808551	8719
**RFWD2**	0.4745	-0.89919	-0.0639625057587457	8720
**AAK1**	0.47453	-0.57772	-0.0638871530783986	8721
**KDELR1**	0.47459	-0.025423	-0.0637364488062657(	8722
**FXN**	0.5254	-0.4768	-0.0637113315685003(	8723
**ORC4**	0.47465	-1.1136	-0.0635857459826747(	8724
**ZNF134**	0.52533	-0.23443	-0.0635355120294476(	8725
**FHOD1**	0.47471	-1.5687	-0.0634350446041826(	8726
**ZBTB11**	0.47474	-0.26996	-0.0633596944557706(	8727
**BHMG1**	0.5252	-1.4021	-0.0632089952384621(	8729
**GNA13**	0.4748	-0.82897	-0.0632089952384621(	8728
**SLCO4C1**	0.52519	-0.81799	-0.0631838788419954(	8730
**RPL22**	0.47486	-0.23174	-0.0630582974576377(	8731
**ZSWIM3**	0.52514	-0.79329	-0.0630582974576377(	8732
**ZNF669**	0.52511	-0.64493	-0.0629829491048304(	8733

**HAVCR1**	0.52508	-0.31291	-0.0629076011098523	8734
**MCF2L2**	0.47497	-0.46362	-0.0627820219122268	8735
**ZNF891**	0.47508	-0.14832	-0.06250575116219010	8736
**TMEM40**	0.47512	-0.21491	-0.06240529025605760	8737
**CFAP53**	0.47518	-0.35817	-0.06225460007829960	8738
**LPGAT1**	0.47519	-1.532	-0.06222948518630400	8739
**NUDT1**	0.52477	-1.6825	-0.06212902601096450	8741
**SNX20**	0.52477	-1.4506	-0.06212902601096450	8740
**NARFL**	0.4753	-0.14639	-0.06195322396334720	8742
**DRAM1**	0.47539	-0.46073	-0.06172719557406580	8743
**CLEC7A**	0.47543	-0.21812	-0.06162673952585430	8744
**KRTAP26-1**	0.47568	-1.1067	-0.06099890328455850	8745
**PDE4A**	0.52419	-0.37111	-0.06067243796418690	8746
**ZC3HAV1**	0.52413	-0.48913	-0.06052176384725660	8747
**DNAJA3**	0.52398	-0.11025	-0.06014508456338720	8748
**CNOT2**	0.52392	-0.37123	-0.05999441524418990	8749
**HS3ST2**	0.52376	-0.65099	-0.05959263704740180	8750
**ITGA7**	0.52376	-1.8044	-0.05959263704740180	8753
**OR4Q3**	0.52376	-1.5786	-0.05959263704740180	8752
**RPL27**	0.52376	-1.2191	-0.05959263704740180	8751
**BAP1**	0.47637	-1.1767	-0.0592661993545089	8757
**NCOA5**	0.47637	-2.1791	-0.0592661993545089	8760
**PCBP2**	0.47637	-0.83052	-0.0592661993545089	8756
**PRLR**	0.47637	-1.8061	-0.0592661993545089	8758
**RBM34**	0.47637	-2.0697	-0.0592661993545089	8759
**SRP19**	0.47637	-0.51779	-0.0592661993545089	8755
**UNC5C**	0.47637	-0.18045	-0.0592661993545089	8754
**OGN**	0.52342	-0.29099	-0.05873889026431770	8761
**UNC50**	0.52342	-0.88546	-0.05873889026431770	8762
**TROVE2**	0.47662	-0.64504	-0.05863845229542920	8763
**TCP10L**	0.52324	-0.59405	-0.05828692405994010	8764
**CENPW**	0.52312	-0.49588	-0.05798561988307840	8765

**SERPINA7**	0.4769	-0.22404	-0.0579354030332508(	8766
**NAAA**	0.52309	-0.52517	-0.0579102946631808	8767
**PCED1B**	0.47701	-0.8977	-0.0576592129696012(	8768
**WNT4**	0.47703	-0.014997	-0.0576089970679759(	8769
**TUBB4A**	0.47716	-0.65349	-0.0572825972458428(	8770
**SLC25A20**	0.47722	-0.903	-0.0571319532363630(	8771
**AIFM2**	0.5226	-1.7079	-0.0566800289804817(	8777
**CDADC1**	0.5226	-0.74977	-0.0566800289804817(	8772
**LETMD1**	0.5226	-1.1794	-0.0566800289804817(	8774
**LIPI**	0.5226	-1.2314	-0.0566800289804817(	8775
**MYL5**	0.5226	-1.109	-0.0566800289804817(	8773
**PLA2G2F**	0.5226	-1.3673	-0.0566800289804817(	8776
**LZTS2**	0.47752	-0.70363	-0.0563787525860042(	8778
**SLC7A8**	0.47754	-0.11083	-0.0563285403523209(	8779
**UTS2R**	0.47762	-0.20989	-0.0561276928378662(	8780
**DDX3X**	0.52236	-0.69278	-0.0560774813136858(	8782
**NUDT5**	0.52236	-0.68689	-0.0560774813136858(	8781
**MAGEA5**	0.47768	-0.24792	-0.0559770586897552	8783
**RCAN1**	0.47774	-0.46824	-0.0558264258130248(	8784
**RRNAD1**	0.47781	-1.1965	-0.0556506890591421(	8785
**LIAS**	0.52204	-1.0004	-0.0552741160868901(	8787
**NAP1L2**	0.52204	-0.98574	-0.0552741160868901(	8786
**MYBPC3**	0.52202	-0.70076	-0.0552239069512351(	8788
**WFDC6**	0.52199	-1.3835	-0.0551485935090064(	8789
**RAC2**	0.47802	-0.21966	-0.0551234890978674(	8790
**POP7**	0.47804	-0.92889	-0.0550732803799020(	8791
**RPRML**	0.47809	-0.43204	-0.0549477591927268(	8792
**DLGAP1**	0.47825	-0.39285	-0.0545460972075974(	8793
**CHTF18**	0.47855	-0.06089	-0.0537930046773209(	8794
**G2E3**	0.52141	-1.7453	-0.0536925946567917(	8798
**HTRA1**	0.52141	-0.89736	-0.0536925946567917(	8796
**USP45**	0.52141	-1.5676	-0.0536925946567917(	8797

**VWCE**	0.52141	-0.86034	-0.05369259465679170	8795
**ZDHHC15**	0.52141	-1.8178	-0.05369259465679170	8799
**PHF20**	0.47865	-1.0245	-0.05354198064178120	8800
**OR8K5**	0.47881	-0.1038	-0.05314034920344370	8801
**DDTL**	0.47889	-0.81147	-0.05293953670631470	8802
**NAT9**	0.47896	-0.95493	-0.05276382752476440	8803
**EFNB2**	0.479	-0.8455	-0.05266342301072520	8804
**ANHX**	0.47904	-0.40079	-0.05256301902818790	8805
**ACSS1**	0.47906	-2.35	-0.05251281723591490	8812
**C2orf42**	0.47906	-1.773	-0.05251281723591490	8810
**LRCH4**	0.47906	-2.3841	-0.05251281723591490	8813
**MUC15**	0.47906	-2.1422	-0.05251281723591490	8811
**SFRP5**	0.47906	-0.45958	-0.05251281723591490	8808
**TERB1**	0.47906	-0.058477	-0.05251281723591490	8806
**UPP2**	0.47906	-0.18128	-0.05251281723591490	8807
**XCL2**	0.47906	-1.7135	-0.05251281723591490	8809
**FCRL5**	0.47912	-0.5684	-0.05236221265355360	8814
**CNTN4**	0.47918	-0.72344	-0.05221160926021190	8815
**ZP1**	0.47946	-0.17184	-0.05150880904534990	8816
**STAB2**	0.47962	-0.55256	-0.05110722038091790	8817
**KIR3DL1**	0.47963	-0.26244	-0.05108212136406070	8818
**HSBP1**	0.52022	-1.3699	-0.05070563996857400	8820
**LY6G6D**	0.52022	-1.8032	-0.05070563996857400	8821
**MTCP1**	0.52022	-1.9284	-0.05070563996857400	8823
**NDOR1**	0.52022	-0.13786	-0.05070563996857400	8819
**RPS6KA4**	0.52022	-1.8356	-0.05070563996857400	8822
**IFFO1**	0.4798	-0.36237	-0.05065544299401870	8824
**MTMR12**	0.5201	-0.25508	-0.05040446003565180	8825
**MIB2**	0.48006	-0.68106	-0.05000289390808160	8826
**MAP3K7CL**	0.48009	-0.60792	-0.04992760115991540	8827
**GLRX**	0.51977	-0.21459	-0.04957623874208750	8828
**CFTR**	0.51974	-0.77131	-0.0495009475942019	8829

**CHST12**	0.48043	-0.49183	-0.04907430305091120	8830
**SALL2**	0.48052	-0.8486	-0.048884843603642930	8831
**TAS2R39**	0.51948	-0.70647	-0.048884843603642890	8832
**PPFIA2**	0.51942	-0.58384	-0.04869785941381030	8833
**C12orf71**	0.48061	-0.32692	-0.04862257151732800	8834
**ADRA1B**	0.4807	-0.66972	-0.04839670948205030	8835
**IGFBP7**	0.48072	-0.62142	-0.04834651825480260	8836
**MOB2**	0.48073	-2.1864	-0.04832142268692150	8837
**EPHA1**	0.48077	-0.36096	-0.04822104071998120	8838
**ASB9**	0.51921	-0.41767	-0.04817084991903980	8839
**NR4A1**	0.51919	-1.4284	-0.04812065923961510	8841
**TGOLN2**	0.51919	-1.5703	-0.04812065923961510	8842
**TRAT1**	0.51919	-1.8159	-0.04812065923961510	8843
**UNC45B**	0.51919	-1.3305	-0.04812065923961510	8840
**PPP1R27**	0.48084	-0.49676	-0.04804537344804430	8844
**USP13**	0.48088	-0.70094	-0.04794499281674020	8845
**ZSWIM5**	0.481	-1.5504	-0.04764385382147660	8846
**PFN3**	0.51893	-0.63938	-0.04746819140740890	8847
**GADL1**	0.51887	-0.63697	-0.04731762479160810	8848
**TADA1**	0.48117	-1.109	-0.04721724764475840	8849
**CD99**	0.51871	-0.12952	-0.04691611905873780	8850
**FAM209A**	0.48136	-0.25293	-0.04674046268616970	8851
**S100A7A**	0.51842	-0.61375	-0.04618840917849360	8852
**COMMD10**	0.48164	-0.62287	-0.04603785158474220	8853
**KRTAP5-4**	0.48171	-0.37925	-0.04586220237942950	8854
**CD274**	0.48174	-0.8292	-0.04578692458272080	8855
**NDP**	0.48175	-1.8864	-0.04576183204158640	8857
**ZNF552**	0.48175	-1.2509	-0.04576183204158640	8856
**ETV2**	0.51809	-0.48788	-0.04536035529737850	8858
**ASB1**	0.51803	-1.1101	-0.04520980340969740	8860
**CDK8**	0.51803	-1.2713	-0.04520980340969740	8861
**IL17RD**	0.51803	-1.1019	-0.04520980340969740	8859

**S100A3**	0.51803	-1.5142	-0.0452098034096974(	8862
**SLC38A3**	0.51803	-1.6896	-0.0452098034096974(	8864
**SPACA5B**	0.51803	-1.5911	-0.0452098034096974(	8863
**C7orf73**	0.482	-0.7457	-0.0451345278509531(	8865
**PRR3**	0.48214	-0.42302	-0.0447832452902396(	8866
**TMEM14A**	0.48218	-1.0386	-0.0446828798628816(	8867
**CA9**	0.51762	-0.83082	-0.0441810594683716	8868
**SNN**	0.48245	-1.2143	-0.0440054249696071(	8869
**ARMCX1**	0.51748	-0.87568	-0.0438297918305855(	8870
**ZNF536**	0.51719	-0.77364	-0.0431021831930778(	8871
**OR4F5**	0.48283	-0.64039	-0.0430520041340427(	8872
**SERTM1**	0.48283	-2.129	-0.0430520041340427(	8873
**MRPL22**	0.48302	-1.2001	-0.0425753084714430(	8874
**ANAPC11**	0.51697	-0.61964	-0.0425502194954711(	8875
**C22orf34**	0.51697	-1.9615	-0.0425502194954711(	8879
**FRMPD4**	0.51697	-1.3992	-0.0425502194954711(	8877
**GPATCH4**	0.51697	-0.70516	-0.0425502194954711(	8876
**MPP7**	0.51697	-1.8572	-0.0425502194954711(	8878
**EGR2**	0.5169	-0.22207	-0.0423745974140814(	8880
**TMEM199**	0.48313	-0.24636	-0.0422993312089072(	8881
**RNF168**	0.48322	-0.003552	-0.0420735340320679(	8882
**TM4SF4**	0.48325	-0.11822	-0.0419982687845906(	8883
**PLA2G4D**	0.48337	-0.80882	-0.0416972101739803(	8884
**RPL39L**	0.48347	-0.58774	-0.0414463308920008(	8885
**ACR**	0.48357	-0.90975	-0.0411954542236335(	8886
**PASK**	0.48366	-1.9456	-0.0409696674436713(	8888
**SLC39A4**	0.51634	-0.72012	-0.0409696674436713(	8887
**GALE**	0.51632	-0.36581	-0.0409194928882780(	8889
**RNFT2**	0.48378	-0.26174	-0.0406686216570241(	8890
**RNF103**	0.48382	-1.3978	-0.0405682738834941(	8891
**ZNF607**	0.48388	-0.54613	-0.0404177529903641(	8892
**OGFOD2**	0.48398	-0.042805	-0.0401668868724878(	8893

**NTNG1**	0.51589	-1.4697	-0.0398407647036842(	8894
**ATP5SL**	0.48421	-0.52983	-0.0395899043880206(	8895
**C18orf63**	0.48421	-1.9543	-0.0395899043880206(	8901
**COL10A1**	0.48421	-1.7297	-0.0395899043880206(	8899
**FAAP24**	0.48421	-1.5131	-0.0395899043880206(	8896
**FAM86B2**	0.48421	-1.5902	-0.0395899043880206(	8897
**HEATR1**	0.48421	-1.8936	-0.0395899043880206(	8900
**KAT5**	0.48421	-1.6737	-0.0395899043880206(	8898
**NBPF20**	0.48421	-1.9851	-0.0395899043880206(	8902
**BCAM**	0.48437	-0.11956	-0.0391885330693547(	8903
**FUT11**	0.51561	-0.57151	-0.0391383621006888(	8904
**PLS3**	0.48442	-0.57588	-0.0390631058327640(	8905
**RNF144A**	0.51545	-1.3281	-0.0387369978949141(	8906
**FSCN3**	0.48457	-0.19183	-0.0386868278102402(	8907
**GXYLT2**	0.4846	-0.9135	-0.0386115728661693(	8908
**SMIM22**	0.48493	-0.5768	-0.0377837828513808(	8909
**CNTN3**	0.515	-1.8321	-0.0376081943852274(	8910
**NPB**	0.515	-2.1337	-0.0376081943852274(	8911
**DVL3**	0.48515	-0.11263	-0.0372319372928450(	8912
**GPIHBP1**	0.51484	-0.3139	-0.0372068536751243(	8913
**MOCOS**	0.4852	-0.49227	-0.0371065194387366(	8914
**HTR1D**	0.51471	-0.93115	-0.0368807687743465(	8915
**LMX1B**	0.48537	-0.50993	-0.0366801030991456	8916
**LPO**	0.51457	-1.2905	-0.0365296048148008(	8917
**ZFAT**	0.51449	-1.4725	-0.0363289417252610(	8918
**ECE2**	0.51445	-0.9196	-0.0362286107308979(	8919
**CHD8**	0.48558	-0.68814	-0.0361533627250997(	8920
**FSD1**	0.51441	-0.63088	-0.0361282801021254(	8921
**TEAD1**	0.51438	-0.55828	-0.0360530323698562(	8922
**COL27A1**	0.48564	-0.12509	-0.0360028673287316(	8923
**MPEG1**	0.48566	-0.072278	-0.0359527023784360(	8924
**PFAS**	0.48581	-0.28073	-0.0355764681336500(	8925

**SREK1IP1**	0.48596	-0.58124	-0.0352002389376228(	8926
**ATXN2**	0.51393	-1.9718	-0.0349243407051187(	8928
**TAL2**	0.51393	-1.3031	-0.0349243407051187(	8927
**CASQ1**	0.48608	-0.99645	-0.0348992591801045(	8929
**EPHB1**	0.48614	-0.49086	-0.0347487704917381(	8930
**ESF1**	0.48616	-0.85916	-0.0346986077711286(	8931
**HTR3A**	0.48621	-0.18212	-0.0345732013523419(	8932
**ZNF530**	0.48625	-1.042	-0.0344728766099430(	8933
**PIP**	0.51374	-0.016419	-0.0344477954787435(	8934
**PPP1R12A**	0.48634	-0.32613	-0.0342471472100562	8935
**ASPG**	0.48643	-0.26096	-0.0340214195599650(	8936
**ADAM21**	0.48665	-0.11307	-0.0334696481544172(	8937
**ALKBH8**	0.48668	-0.59167	-0.0333944073946100(	8938
**SLC34A1**	0.48677	-0.78245	-0.0331686862510447(	8939
**EIF5A2**	0.48679	-1.4669	-0.0331185262274879(	8942
**IGH**	0.48679	-2.1007	-0.0331185262274879(	8946
**REXO4**	0.48679	-0.23576	-0.0331185262274879(	8940
**SEMA5A**	0.48679	-1.7892	-0.0331185262274879(	8944
**SNRPG**	0.48679	-2.0142	-0.0331185262274879(	8945
**STX2**	0.48679	-1.7062	-0.0331185262274879(	8943
**ZBTB21**	0.48679	-0.92447	-0.0331185262274879(	8941
**MRPS14**	0.48687	-1.0833	-0.0329178869676925(	8947
**NCR2**	0.48691	-0.70344	-0.0328175678366884(	8948
**PKNOX1**	0.48696	-1.0488	-0.0326921693886342	8949
**PSG2**	0.51302	-0.83242	-0.0326420101538932(	8950
**PSMB6**	0.48705	-0.81718	-0.0324664534801390(	8951
**DLG3**	0.48709	-0.63651	-0.0323661358318081(	8952
**SBNO1**	0.5129	-0.87183	-0.0323410564708126(	8953
**SLFNL1**	0.48712	-0.051131	-0.0322908978100163(	8954
**LY6G6C**	0.51282	-1.6989	-0.0321404223160475(	8957
**RDM1**	0.51282	-0.26172	-0.0321404223160475(	8955
**SNRPD2**	0.51282	-1.6382	-0.0321404223160475(	8956

**RNF112**	0.4872	-0.93358	-0.0320902639804436(	8958
**EMX1**	0.48755	-0.76294	-0.0312125061041362(	8960
**FAM160B1**	0.48755	-0.60401	-0.0312125061041362(	8959
**CDC73**	0.51232	-0.047616	-0.0308864879306098(	8961
**ARL4C**	0.51228	-0.033016	-0.0307861753106742(	8962
**QRICH1**	0.51221	-1.1841	-0.0306106289730704(	8963
**OR1N2**	0.51218	-1.4686	-0.0305353951184281(	8964
**NAGA**	0.48783	-0.080825	-0.0305103172054385(	8965
**STXBP4**	0.48784	-0.6209	-0.0304852393117006(	8966
**PECR**	0.48788	-0.2864	-0.0303849279289572(	8967
**SYT16**	0.48799	-0.56495	-0.0301090732051889(	8968
**MPPED2**	0.48811	-0.27457	-0.0298081434005444(	8969
**FBXL20**	0.48825	-0.2914	-0.0294570620501345(	8970
**CNTROB**	0.51173	-1.7093	-0.0294069078699571(	8974
**DCAF15**	0.51173	-0.52816	-0.0294069078699571(	8971
**LIPK**	0.51173	-1.5593	-0.0294069078699571(	8973
**MMRN1**	0.51173	-1.8203	-0.0294069078699571(	8975
**ZC2HC1A**	0.51173	-1.0172	-0.0294069078699571(	8972
**KIFC3**	0.48832	-0.16816	-0.0292815227440156(	8976
**AREG**	0.48838	-0.23761	-0.0291310612025502(	8977
**KLF15**	0.48852	-0.20638	-0.0287799868405339(	8978
**DNAJC18**	0.48856	-0.10491	-0.0286796805357797	8979
**DENND5A**	0.48886	-0.24224	-0.0279273924123499(	8980
**NAT2**	0.5111	-0.79374	-0.0278270885372847(	8981
**ADAMTS6**	0.48895	-0.45982	-0.0277017090885706(	8982
**GOLGA3**	0.51096	-0.69014	-0.0274760271812559(	8983
**SLC22A13**	0.48905	-0.73027	-0.0274509515007144(	8984
**SLC36A2**	0.48906	-0.08592	-0.0274258758375020(	8985
**PIK3R4**	0.48912	-0.45814	-0.0272754222212687(	8986
**ZC3H8**	0.48923	-0.032994	-0.0269995921990688(	8987
**ABCB5**	0.51075	-0.19886	-0.0269494415081560(	8988
**CCL13**	0.48928	-1.2526	-0.0268742155993569(	8991

**KRT2**	0.48928	-1.9429	-0.0268742155993569(	8993
**MYO1E**	0.48928	-1.7297	-0.0268742155993569(	8992
**PCDHGB3**	0.48928	-0.12686	-0.0268742155993569(	8989
**SHOX2**	0.48928	-0.84204	-0.0268742155993569(	8990
**PAPPA**	0.5107	-1.5414	-0.0268240650783535(	8994
**ETFB**	0.48935	-0.16334	-0.0266986890719490(	8995
**MOXD1**	0.51063	-0.38628	-0.0266485387874972(	8996
**ADIPOR1**	0.48944	-0.34743	-0.0264730133210965(	8997
**KIF21A**	0.5105	-1.6277	-0.0263225635734287(	8998
**ZRANB1**	0.48962	-0.7123	-0.0260216658697472(	8999
**PAG1**	0.51036	-0.26059	-0.0259715164832478(	9000
**COPB1**	0.48975	-1.1649	-0.0256956960267548(	9001
**OR6N2**	0.48978	-0.74004	-0.0256204726075457(	9002
**C9orf135**	0.51016	-0.48229	-0.0254700262055604(	9003
**NR2E1**	0.51012	-1.9846	-0.0253697289261559(	9004
**ZNF746**	0.51	-1.1581	-0.0250688386220688(	9005
**TAGLN**	0.49007	-0.41011	-0.0248933203326920(	9006
**PSORS1C2**	0.50992	-0.06758	-0.0248682463543717(	9007
**ATP2B4**	0.49013	-0.9179	-0.0247428766980691(	9008
**PPP1R21**	0.49014	-1.0998	-0.0247178028137581(	9009
**CLK1**	0.49017	-0.61922	-0.0246425812544400(	9010
**SMG5**	0.49026	-0.57856	-0.0244169174153447(	9011
**THRA**	0.50964	-0.57187	-0.0241661812811476(	9012
**RAD9B**	0.49038	-0.094717	-0.0241160342380135(	9013
**ENY2**	0.5096	-1.6965	-0.0240658872558204(	9015
**KLRC2**	0.5096	-1.5251	-0.0240658872558204(	9014
**TPRX1**	0.5096	-2.1683	-0.0240658872558204(	9016
**CABP2**	0.49046	-0.55225	-0.0239154466736275(	9017
**SNX11**	0.49048	-0.1542	-0.0238652999339399(	9018
**DMWD**	0.49049	-0.04076	-0.0238402265867195(	9019
**SLC38A4**	0.49052	-0.084232	-0.0237650066353715(	9020
**ZNF831**	0.49065	-0.68767	-0.0234390550689540(	9021

LOC1019299	0.50933	-0.78118	-0.02338890889713950	9023
MYPOP	0.50933	-0.40845	-0.02338890889713950	9022
ZCCHC10	0.50925	-0.1824	-0.02318832479977760	9024
ARID5B	0.50916	-0.067559	-0.02296266881079040	9025
CTNS	0.49085	-2.1517	-0.02293759599589080	9026
SOX1	0.49089	-0.7617	-0.02283730488109180	9027
CENPH	0.50905	-0.61898	-0.02268686864155360	9028
ANKUB1	0.49097	-0.070878	-0.02263672334324760	9029
HHATL	0.50898	-0.66824	-0.02251136034757280	9030
KDM3B	0.49104	-0.66852	-0.02246121524900870	9031
TMEM110-M	0.49111	-0.27786	-0.02228570785039830	9032
FAM89A	0.50877	-0.74932	-0.02198483962706230	9033
DAP	0.50872	-0.2868	-0.02185947845954380	9034
MSC	0.49128	-0.94904	-0.02185947845954350	9035
CTDSP2	0.49138	-0.16488	-0.02160875715895270	9036
RIMKLA	0.50849	-0.67138	-0.02128282150822620	9037
CAMKK1	0.49152	-1.7383	-0.02125774962973180	9041
ERI2	0.49152	-1.3577	-0.02125774962973180	9038
SCYL1	0.49152	-1.6973	-0.02125774962973180	9040
TRIM7	0.49152	-1.6439	-0.02125774962973180	9039
PCOLCE2	0.49154	-0.81844	-0.02120760591305320	9042
FAM3C	0.50836	-0.6304	-0.02095688813172610	9043
PRKX	0.50831	-0.51727	-0.02083152973901260	9044
RTL1	0.49169	-1.072	-0.02083152973901230	9045
SHISA2	0.50827	-0.18066	-0.02073124326206390	9046
ACTR5	0.49178	-0.53638	-0.02060588546075500	9048
XRCC3	0.49178	-0.38765	-0.02060588546075500	9047
DOCK11	0.50818	-0.025517	-0.02050559945438150	9049
ANK3	0.49186	-0.62034	-0.02040531365551690	9050
UBLCP1	0.50808	-0.084208	-0.02025488534409550	9051
EFTUD2	0.49204	-1.2221	-0.01995403010154910	9052
CELA3A	0.50793	-1.0167	-0.01987881657599690	9053

**NBPF4**	0.49211	-0.64462	-0.0197785320512525(	9054
**CDC25A**	0.49223	-0.47322	-0.0194776796742500(	9055
**EBP**	0.50768	-0.63696	-0.0192520415576389(	9056
**PDCD1**	0.50753	-0.26647	-0.0188759802172052(	9057
**FABP7**	0.5075	-0.99527	-0.0188007682733833(	9058
**FAM135A**	0.50749	-1.1875	-0.0187756976492640(	9061
**GRB7**	0.50749	-1.6728	-0.0187756976492640(	9063
**SIRT3**	0.50749	-0.51219	-0.0187756976492640(	9059
**SLC29A1**	0.50749	-1.3676	-0.0187756976492640(	9062
**TMC7**	0.50749	-2.0139	-0.0187756976492640(	9064
**TRIM48**	0.50749	-0.94076	-0.0187756976492640(	9060
**HRH1**	0.50747	-0.83177	-0.0187255564366607(	9065
**HOMEZ**	0.49256	-0.86114	-0.0186503447066109(	9066
**SNAPC1**	0.49279	-0.76018	-0.0180737249413695(	9067
**C4B**	0.5072	-1.654	-0.0180486546555027(	9068
**BTG2**	0.50706	-0.8546	-0.0176976718461529(	9069
**SNAP47**	0.507	-0.56562	-0.0175472513171359(	9070
**NAGPA**	0.50696	-0.24403	-0.0174469711869386(	9071
**CDK13**	0.50694	-0.53317	-0.0173968311882073	9072
**GTF2IRD2**	0.49311	-0.15938	-0.0172714813839397(	9073
**TCP11L1**	0.50689	-0.60611	-0.0172714813839394(	9074
**TMEM151A**	0.49318	-1.4879	-0.0170959921170305(	9075
**SIDT1**	0.4932	-0.42013	-0.0170458524241958(	9076
**SCARB2**	0.50676	-1.5814	-0.0169455731679901(	9077
**CYP26A1**	0.49333	-0.032543	-0.0167199454680671	9078
**PTGES3L-A**	0.49344	-1.0117	-0.0164441794435415(	9079
**TBX3**	0.50655	-0.48497	-0.0164191098677944(	9080
**PATE4**	0.50654	-0.70712	-0.0163940403024529(	9081
**TMEM57**	0.50644	-2.1149	-0.0161433452179152(	9082
**OTOP2**	0.50641	-0.62699	-0.0160681368928171(	9084
**PPP1R13L**	0.50641	-0.48594	-0.0160681368928171(	9083
**CHMP3**	0.50639	-0.64763	-0.0160179980603747(	9085

FRG2	0.49367	-1.8478	-0.0158675818062546(	9086
ENO3	0.50625	-0.19897	-0.0156670273635526(	9087
CCER2	0.49379	-0.61454	-0.0155667503811343(	9088
FAM171B	0.50612	-0.81932	-0.0153411277466545(	9089
SLC38A6	0.49391	-0.76642	-0.0152659203775374(	9090
APRT	0.49393	-0.069141	-0.0152157821799024(	9091
DHRS12	0.50607	-1.0127	-0.0152157821799024(	9092
UBE3A	0.49395	-0.24181	-0.0151656440208732(	9093
PCDHGB2	0.494	-1.1668	-0.0150402987913779(	9094
TMEM249	0.50597	-0.76118	-0.0149650917683071(	9095
HOXC9	0.49408	-0.21119	-0.0148397469195352(	9096
ELP2	0.49415	-0.63107	-0.0146642645262839(	9097
DMBX1	0.49424	-0.40452	-0.0144386449756607(	9098
SLC9A3R2	0.49429	-0.59318	-0.0143133011023592(	9099
CIRBP	0.4943	-1.5791	-0.0142882323550196(	9101
DLL1	0.4943	-0.44111	-0.0142882323550196(	9100
HCAR1	0.4943	-1.7707	-0.0142882323550196(	9102
PCDH11Y	0.4943	-1.9231	-0.0142882323550196(	9103
SRGAP1	0.4943	-1.9865	-0.0142882323550196(	9104
LRRC45	0.50568	-0.81757	-0.0142380948875346(	9105
CSGALNAC'	0.49458	-0.29088	-0.0135863110545858(	9107
WDYHV1	0.50542	-0.14338	-0.0135863110545858(	9106
OXER1	0.5053	-1.0276	-0.0132854897314774(	9108
SRPK1	0.49477	-0.23129	-0.0131100111894425(	9109
C11orf21	0.49485	-0.77451	-0.0129094647838457(	9111
ZNF347	0.50515	-0.49591	-0.0129094647838457(	9110
HEBP1	0.50508	-1.6506	-0.0127339871100381(	9112
PCIF1	0.50503	-1.0468	-0.0126086461576550(	9113
MRPL1	0.50498	-0.51885	-0.0124833054057091(	9114
EPHA6	0.50495	-1.097	-0.0124081010499336(	9115
CYP2R1	0.49507	-0.66406	-0.0123579648522355(	9116
TDG	0.5049	-0.78757	-0.0122827606146003(	9117

**CXCL17**	0.50483	-0.31337	-0.0121072843328495(	9118
**PGM5**	0.4952	-0.85117	-0.0120320803279619(	9119
**LENG1**	0.49522	-0.37335	-0.0119819443630094(	9120
**CATSPERB**	0.50476	-2.0379	-0.0119318084285567(	9124
**CTNNA1**	0.50476	-1.1691	-0.0119318084285567(	9121
**EGFLAM**	0.50476	-2.1802	-0.0119318084285567(	9125
**PBK**	0.50476	-1.5245	-0.0119318084285567(	9123
**TRIM49D2**	0.50476	-1.5113	-0.0119318084285567(	9122
**COX4I1**	0.49529	-0.92567	-0.0118064687250327(	9126
**APCDD1**	0.5047	-0.54807	-0.0117814008069350(	9127
**CBX1**	0.5047	-1.1425	-0.0117814008069350(	9128
**SDHD**	0.50465	-0.1936	-0.0116560613286168(	9129
**THAP7**	0.49539	-0.25651	-0.0115557898796253(	9130
**NKX2-3**	0.49553	-0.78383	-0.0112048407286210(	9131
**SUSD5**	0.49562	-1.7804	-0.0109792313015845(	9132
**SPACA6**	0.49575	-0.24622	-0.0106533520155715(	9133
**C17orf102**	0.50424	-0.61893	-0.0106282844261713(	9134
**MTPAP**	0.49579	-0.64042	-0.0105530816985653(	9135
**ZNF343**	0.49585	-2.0577	-0.0104026764246223(	9136
**SOX4**	0.50412	-1.1892	-0.0103274738774368(	9137
**HS3ST5**	0.49592	-0.50036	-0.0102272039066809(	9138
**ACSF2**	0.50403	-0.88911	-0.0101018665899356(	9139
**CLCN3**	0.4961	-0.45209	-0.00977599031575357	9140
**MVD**	0.50386	-0.65882	-0.0096757209067738	9141
**SLC6A8**	0.49618	-0.057796	-0.00957545159666921	9142
**MTMR1**	0.49624	-0.057843	-0.0094250478147031	9143
**TNN**	0.49626	-0.48072	-0.0093749132690628	9144
**METTL18**	0.49629	-0.75522	-0.0092997114954967	9145
**STXBP6**	0.49629	-0.94669	-0.0092997114954967	9146
**TNS3**	0.4964	-0.27419	-0.0090239721135580	9147
**CEMIP**	0.50355	-0.58601	-0.0088986362629685(	9148
**POMK**	0.49646	-0.25601	-0.0088735691099925(	9149

**EYA1**	0.50348	-0.14732	-0.00872316631080177	9150
**SLC51A**	0.50332	-0.53812	-0.00832209315300726	9151
**MED17**	0.49675	-0.40791	-0.00814662407946168	9153
**RNASE11**	0.49675	-0.086399	-0.00814662407946168	9152
**GALNT11**	0.50317	-0.38696	-0.00794608830840091	9154
**SLC12A7**	0.49686	-0.10721	-0.00787088747877041	9155
**SEMA3D**	0.50312	-0.25149	-0.00782075361762165	9156
**CLDN1**	0.50305	-0.086239	-0.00764528526083096	9157
**CCR1**	0.50293	-0.43834	-0.00734448291997062	9158
**ITGA5**	0.4971	-1.567	-0.00726928244199923	9159
**CEP19**	0.49711	-0.58992	-0.00724421562537399	9160
**DNAJC11**	0.49716	-1.5293	-0.00711888161178188	9161
**SERP1**	0.49716	-1.5299	-0.00711888161178188	9162
**TMBIM4**	0.49716	-2.24	-0.00711888161178188	9163
**HPCAL1**	0.49718	-0.004089	-0.00706874803850096	9164
**LOXHD1**	0.50278	-0.9103	-0.00696848094637187	9165
**ZNF181**	0.50275	-1.0047	-0.00689328067440957	9166
**HILPDA**	0.49727	-1.021	-0.00684314718197810	9167
**PLK5**	0.49732	-0.31628	-0.00671781352771629	9168
**DOK7**	0.49735	-0.21108	-0.00664261338720589	9169
**PPARD**	0.49742	-0.68619	-0.00646714654173867	9170
**WEE2**	0.49747	-1.2464	-0.00634181320609776	9171
**SNX27**	0.49759	-0.089922	-0.00604101361413434	9172
**DCUN1D5**	0.5023	-0.07931	-0.00576528114921332	9173
**ADO**	0.49771	-0.050685	-0.00574021458418799	9174
**BORCS8**	0.49776	-0.51414	-0.00561488181446841	9176
**FAM53B**	0.50224	-0.25157	-0.00561488181446841	9175
**OCRL**	0.5021	-1.102	-0.00526395053657723	9177
**CALHM1**	0.50206	-1.5294	-0.00516368458204799	9180
**FAM156A**	0.50206	-1.5105	-0.00516368458204799	9179
**FSBP**	0.50206	-0.68382	-0.00516368458204799	9178
**IER5**	0.50206	-1.5881	-0.00516368458204799	9181

**PRDM4**	0.49803	-0.93108	-0.00493808637900605	9182
**SLC9A5**	0.49804	-0.36626	-0.00491301992827131	9183
**TDRD12**	0.50195	-0.12862	-0.00488795348073333	9184
**GSTM2**	0.50174	-0.95827	-0.00436155879302253	9186
**TXNL1**	0.50174	-0.74853	-0.00436155879302253	9185
**SLC13A3**	0.49836	-0.55586	-0.00411089510686677	9187
**TVP23B**	0.49852	-0.49586	-0.00370983376287917	9188
**ARHGEF33**	0.50145	-0.11011	-0.00363463483244645	9189
**MDFI**	0.49858	-1.0094	-0.00355943592357803	9190
**PCDHA1**	0.50139	-0.27426	-0.00348423703584948	9191
**DHX40**	0.50126	-0.25633	-0.00315837542297059	9192
**ATP9A**	0.50118	-0.19901	-0.00295784537806824	9193
**DSTYK**	0.50107	-0.55596	-0.00268211677078300	9194
**MEGF10**	0.50098	-1.5577	-0.00245652080045105	9195
**DUSP12**	0.49903	-1.2329	-0.00243145459002636	9196
**KIAA1524**	0.50093	-0.53021	-0.00233118976459532	9198
**PPM1G**	0.49907	-0.015042	-0.00233118976459532	9197
**TFG**	0.49927	-0.31633	-0.00182986599651172	9199
**ZFP36**	0.49931	-0.36018	-0.00172960130767870	9200
**MRI1**	0.49932	-0.45289	-0.00170453513851758	9201
**SNTB1**	0.50068	-1.1249	-0.00170453513851758	9202
**MB**	0.50053	-1.0063	-0.00132854273481080	9203
**HECTD4**	0.49951	-0.010918	-0.00122827813218832	9204
**ACSS3**	0.49955	-1.1986	-0.00112801354379510	9206
**GOLGA8O**	0.49955	-0.41948	-0.00112801354379510	9205
**GTF2H4**	0.49955	-2.1488	-0.00112801354379510	9209
**HYAL1**	0.49955	-1.9816	-0.00112801354379510	9208
**ZNF589**	0.49955	-1.3445	-0.00112801354379510	9207
**CNR2**	0.49965	-2.1485	-0.00087735212847227	9210
**C22orf31**	0.49967	-0.23301	-0.00082721985394572	9211
**P3H4**	0.49971	-0.89891	-0.00072695531242555	9212
**SNX30**	0.49973	-0.49565	-0.00067682304518094	9213

**CKMT1B**	0.50007	-0.44084	-0.00017550047171686	9215
**MS4A4E**	0.49993	-0.42707	-0.00017550047171686	9214
**NTMT1**	0.49995	-0.000925	-0.00012536822150633	9216
**PDE3B**	0.49997	-0.46915	-0.00007523597209194	9217
**OR10W1**	0.50001	-0.21059	-0.00002510372334710	9218
**PDE6D**	0.50001	-0.60609	-0.00002510372334710	9219
**DEFB118**	0.49999	1.6023	0.00002510372334732	9220
**CADM1**	0.49989	0.39549	0.00027576497502482	9221
**DOPEY1**	0.49975	0.10913	0.00062669078011181	9222
**PGM3**	0.49969	0.6886	0.00077708758197173	9223
**MRAP**	0.50032	0.99122	0.00080215371764710	9224
**TAF1A**	0.50032	0.63902	0.00080215371764710	9225
**SNRPB2**	0.50036	0.31821	0.00090241826673306	9226
**CHTF8**	0.50046	0.061002	0.00115307968961414	9228
**LPCAT1**	0.50046	0.70553	0.00115307968961414	9227
**OXR1**	0.49947	0.35653	0.00132854273481052	9229
**ZSCAN5A**	0.50054	1.4935	0.00135360888780729	9230
**GNAQ**	0.50056	0.99964	0.00140374119671951	9231
**SLC6A9**	0.49941	0.5421	0.00147893966762109	9232
**FAM166B**	0.5006	0.86363	0.00150400582666002	9233
**ATP5C1**	0.49917	0.54574	0.00208052780940855	9234
**SPI1**	0.50084	0.19461	0.00210559399826514	9235
**HP**	0.50088	0.36494	0.00220585876823428	9236
**TAF1**	0.50089	0.58903	0.00223092496444090	9237
**RAD51B**	0.50096	0.3138	0.00240638838124335	9238
**MADD**	0.50099	0.91083	0.00248158701253460	9239
**ARMC8**	0.49901	1.5082	0.00248158701253493	9240
**LAMC3**	0.50101	0.32646	0.00253171944173858	9241
**MYL1**	0.49893	0.24908	0.00268211677078300	9242
**KBTBD4**	0.49885	0.34186	0.00288264664420387	9243
**REV3L**	0.50117	0.68243	0.00293277913149591	9244
**ZNF436**	0.50122	0.76108	0.00305811038424008	9245

**MIER1**	0.49873	1.3113	0.00318344168785834(	9246
**WTIP**	0.50129	0.56795	0.00323357422399010(	9247
**SLC6A14**	0.50135	0.67652	0.00338397188436298(	9248
**NME7**	0.50139	0.20172	0.00348423703584948(	9249
**COMMD7**	0.5014	1.5595	0.00350930332943618(	9250
**POU2AF1**	0.49859	0.048177	0.00353436962534098(	9251
**IDH1**	0.50145	0.98117	0.00363463483244645(	9257
**NBEAL1**	0.50145	1.2063	0.00363463483244645(	9256
**OR2C1**	0.50145	1.4212	0.00363463483244645(	9255
**PDIK1L**	0.50145	1.4394	0.00363463483244645(	9254
**SDCCAG3**	0.50145	1.953	0.00363463483244645(	9253
**SEC61G**	0.50145	0.20613	0.00363463483244645(	9258
**TRIM71**	0.50145	1.9977	0.00363463483244645(	9252
**HMGN1**	0.50148	0.8998	0.00370983376287889(	9259
**COBLL1**	0.5015	0.45419	0.00375996639535637(	9260
**GLMN**	0.49844	0.53872	0.00391036435267709(	9261
**IGLL5**	0.49836	0.8149	0.00411089510686677(	9262
**EHD2**	0.49835	0.82295	0.00413596146309974(	9268
**GPA33**	0.49835	1.3536	0.00413596146309974(	9266
**KCTD20**	0.49835	1.5349	0.00413596146309974(	9264
**LYPLA1**	0.49835	2.1022	0.00413596146309974(	9263
**SUMO2**	0.49835	1.4613	0.00413596146309974(	9265
**YEATS4**	0.49835	1.3352	0.00413596146309974(	9267
**MUC1**	0.50166	0.15714	0.00416102782204178(	9269
**FAM174B**	0.49826	1.1394	0.00436155879302280(	9270
**SSTR3**	0.50191	1.1314	0.00478768772223183(	9271
**PNMA1**	0.49807	0.94281	0.00483782059518396(	9272
**DIP2A**	0.49805	0.34928	0.00488795348073333	9273
**AGTRAP**	0.502	2.0732	0.00501328575054554(	9274
**SLBP**	0.49792	1.0663	0.00521381755254328(	9275
**SLITRK1**	0.50229	0.058691	0.00574021458418799(	9276
**C1orf116**	0.50232	0.66564	0.00581541429046901(	9277

**WDR54**	0.50239	0.5185	0.005990880404179840	9278
**PGPEP1**	0.50242	1.7368	0.006066080224958040	9279
**CREBBP**	0.50272	0.33421	0.006818080442377400	9280
**DLGAP2**	0.49716	0.048025	0.007118881611781880	9281
**KCNH1**	0.5029	0.42289	0.007269282441999230	9282
**MAGEA2B**	0.50299	0.70584	0.007494884003758180	9283
**TAF8**	0.497	0.48698	0.007519950867811720	9284
**HSPE1-MOB**	0.49693	0.95633	0.007695419052283360	9285
**DLL3**	0.50317	0.43051	0.007946088308400910	9286
**SERHL2**	0.50317	0.019441	0.007946088308400910	9287
**PLIN3**	0.49681	0.078725	0.007996222220275270	9288
**RNF6**	0.49678	0.56429	0.008071423126582680	9289
**NSUN6**	0.49665	0.32682	0.00839729426418686	9290
**DCP1A**	0.50348	0.13582	0.00872316631080177	9291
**HSPD1**	0.50349	1.9336	0.00874823342995158	9292
**SPATC1L**	0.49651	0.018256	0.00874823342995158	9293
**SYNE3**	0.50352	1.3087	0.00882343482105401	9294
**C16orf91**	0.49642	0.92767	0.008973837756117630	9295
**TYRO3**	0.50363	0.32666	0.009099173693042440	9296
**SORCS2**	0.49624	0.61488	0.009425047814703190	9297
**B2M**	0.50391	0.49378	0.0098010576835652	9298
**JPT1**	0.49604	0.52878	0.009926394616810910	9299
**GNPAT**	0.504	0.92167	0.010026664277373400	9300
**SPANXC**	0.504	0.21825	0.010026664277373400	9301
**RERE**	0.50403	0.086191	0.010101866589935600	9303
**TCTE3**	0.50403	1.9838	0.010101866589935600	9302
**NABP2**	0.5041	1.2284	0.010277338878945900	9304
**MEN1**	0.50414	0.44803	0.010377608902279300	9305
**FOXL1**	0.50423	0.56301	0.010603216843546200	9306
**KRTAP9-3**	0.49577	0.63338	0.010603216843546400	9307
**TREM2**	0.50427	1.6807	0.010703487214765000	9308
**LRBA**	0.49561	0.7609	0.011004298987503800	9309

**ARHGEF25**	0.49554	0.56989	0.011179772986164300	9310
**ACOT13**	0.49551	1.3071	0.01125497623496390	9316
**CNNM3**	0.49551	1.5463	0.01125497623496390	9315
**FBXL17**	0.49551	1.6223	0.01125497623496390	9314
**FYCO1**	0.49551	1.3031	0.01125497623496390	9317
**P2RY1**	0.49551	1.7962	0.01125497623496390	9313
**T**	0.49551	2.0148	0.01125497623496390	9311
**TXNRD3NB**	0.49551	1.8283	0.01125497623496390	9312
**APELA**	0.50468	1.6355	0.011731264993221000	9321
**CARD17**	0.50468	0.95616	0.011731264993221000	9326
**CSPP1**	0.50468	0.69481	0.011731264993221000	9328
**EPHA8**	0.50468	1.8422	0.011731264993221000	9318
**GPR132**	0.50468	1.5796	0.011731264993221000	9322
**GZF1**	0.50468	1.3343	0.011731264993221000	9325
**LCE1D**	0.50468	1.4633	0.011731264993221000	9323
**LIMD1**	0.50468	1.678	0.011731264993221000	9319
**P3H2**	0.50468	1.392	0.011731264993221000	9324
**SRSF8**	0.50468	0.13782	0.011731264993221000	9330
**TNFAIP6**	0.50468	0.92511	0.011731264993221000	9327
**UQCRHL**	0.50468	1.6592	0.011731264993221000	9320
**VPS11**	0.50468	0.62488	0.011731264993221000	9329
**LDOC1**	0.50481	0.58373	0.012057148321913700	9331
**ARMC12**	0.495	0.022152	0.012533441682561300	9332
**ABHD1**	0.49498	0.64106	0.012583577991293700	9334
**TMEM154**	0.50502	0.96	0.012583577991293700	9333
**SSR1**	0.50505	0.59877	0.012658782514446100	9335
**TRIP12**	0.49488	0.18746	0.012834260017578400	9336
**AQP5**	0.49487	0.64441	0.012859328264821000	9337
**FUT3**	0.49481	0.68918	0.013009737920493900	9338
**HIST4H4**	0.50533	1.0339	0.013360694947231600	9339
**GPR139**	0.50536	0.54365	0.013435900239385300	9340
**NDUFS2**	0.49461	0.02694	0.013511105608361900	9341

**PRSS50**	0.50542	0.64712	0.01358631105458580	9342
**FOXRED2**	0.49452	0.55863	0.01373672218047480	9343
**CANT1**	0.50548	0.57075	0.01373672218047510	9344
**FMR1NB**	0.49448	0.0763	0.01383699643868050	9345
**GTDC1**	0.49445	0.29278	0.01391220222454710	9346
**PROP1**	0.50558	0.99188	0.01398740808992320	9348
**SPG20**	0.50558	1.1419	0.01398740808992320	9347
**GOLPH3**	0.49441	0.37523	0.01401247672945810	9349
**LYPLA2**	0.50564	0.51598	0.01413782006090520	9350
**COQ10B**	0.49435	1.1892	0.01416288875405960	9351
**PRKD3**	0.49428	1.7242	0.01433836985878430	9352
**GPR152**	0.50577	0.74914	0.01446371377775080	9353
**ZNF541**	0.49418	0.4813	0.01458905792622220	9354
**BEND2**	0.50598	0.56707	0.01499016076648220	9355
**PRDM15**	0.506	1.1671	0.01504029879137790	9356
**TBC1D17**	0.49394	0.11936	0.01519071309556930	9357
**EFEMP2**	0.50613	0.46703	0.01536619688913240	9359
**RHBDF2**	0.50613	1.3009	0.01536619688913240	9358
**CASP1**	0.50621	0.49814	0.01556675038113450	9360
**ARID1B**	0.49373	0.45913	0.01571716591430690	9361
**ALDH3A1**	0.49365	0.78088	0.01591772051721920	9362
**ALKBH3**	0.49365	0.65219	0.01591772051721920	9363
**PPP1R36**	0.49361	0.45815	0.01601799806037470	9364
**GRHL1**	0.5064	0.97373	0.01604306747151020	9365
**TMEM71**	0.50647	0.53458	0.01621855363511230	9366
**LEO1**	0.4935	0.37553	0.01629376214483310	9367
**ALKBH6**	0.50656	0.51717	0.01644417944354150	9368
**MAP3K12**	0.49341	0.2736	0.01651938823337920	9369
**RNF44**	0.49341	0.032494	0.01651938823337920	9370
**PARD6G**	0.50665	0.64194	0.01666980609614040	9371
**CDKN2C**	0.49334	0.29924	0.01669487577681380	9372
**ANXA8**	0.49319	0.86306	0.01707092226520610	9373

**ARHGAP44**	0.50683	1.4833	0.0171210619796853	9374
**NOTO**	0.49313	0.67133	0.0172213415389270	9375
**IGF2BP1**	0.50693	0.65354	0.0173717612053775	9376
**WDR74**	0.49305	1.5446	0.0174219011820558	9377
**C9orf64**	0.507	0.80744	0.0175472513171359	9378
**OLIG1**	0.50705	1.4354	0.01767260173005570	9379
**DICER1**	0.49294	1.2386	0.0176976718461534	9380
**MLST8**	0.49288	1.3243	0.01784809277865470	9384
**MOS**	0.49288	1.8175	0.01784809277865470	9381
**NXF5**	0.49288	1.3998	0.01784809277865470	9383
**RPGRIP1**	0.49288	1.0864	0.01784809277865470	9385
**THAP2**	0.49288	1.0081	0.01784809277865470	9386
**TMEM216**	0.49288	1.706	0.01784809277865470	9382
**RTP4**	0.49286	1.5843	0.0178982331797389	9387
**COLCA1**	0.50714	1.735	0.01789823317973920	9388
**KCNN4**	0.49281	1.556	0.0180235843810641	9389
**NKX2-2**	0.49272	1.93	0.0182492172637440	9390
**TTLL9**	0.50731	1.2245	0.0183244284320403	9391
**IYD**	0.50737	0.37985	0.0184748510822834	9392
**GJC3**	0.50741	0.60072	0.01857513308281010	9393
**GJA10**	0.49251	0.92928	0.0187756976492640	9394
**CHCHD1**	0.5075	0.52714	0.0188007682733833	9395
**EFCAB13**	0.50756	0.042734	0.0189511922685481	9396
**RBMS3**	0.50757	1.1066	0.0189762629762964	9397
**ALDOB**	0.4924	0.7023	0.01905147517166430	9398
**MRPL20**	0.49236	1.8855	0.0191517582676922	9399
**DAPK3**	0.50767	1.0442	0.019226970716917	9404
**G6PC**	0.50767	1.7005	0.019226970716917	9401
**GPR84**	0.50767	1.6188	0.019226970716917	9402
**PLCL2**	0.50767	1.7672	0.019226970716917	9400
**TIMP2**	0.50767	1.3478	0.019226970716917	9403
**USP17L1**	0.50767	0.83332	0.019226970716917	9405

**TRPV3**	0.4922	0.79745	0.01955289260105990	9406
**FAAH2**	0.50786	0.58649	0.01970331878913640	9408
**ZNFX1**	0.50786	1.6893	0.01970331878913640	9407
**NPFFR1**	0.49212	0.55041	0.01975346095138790	9409
**PRAMEF17**	0.49203	1.839	0.01997910130195880	9410
**SH3TC2**	0.50811	0.004699	0.02033009944193950	9412
**TOMM20L**	0.50811	1.9731	0.02033009944193950	9411
**CCDC24**	0.49183	0.41958	0.02048052798525020	9413
**C2CD2**	0.5083	0.99414	0.02080645810005340	9415
**IFNGR1**	0.4917	1.7287	0.02080645810005340	9414
**TTC7A**	0.49166	0.11835	0.02090674473499360	9416
**MMP7**	0.50835	0.91606	0.02093181642674110	9417
**TBXA2R**	0.50839	1.0455	0.02103210332625750	9418
**MGARP**	0.49158	0.48697	0.02110731864048650	9419
**KBTBD8**	0.50849	0.43839	0.02128282150822620	9420
**SEC24C**	0.49148	0.66604	0.02135803722451080	9421
**STXBP1**	0.50868	0.86259	0.02175918977436220	9422
**XAGE2**	0.49125	1.7226	0.02193469511847250	9423
**ICOSLG**	0.50881	1.8712	0.02208512881113800	9424
**SMCO3**	0.50883	0.81753	0.02213527348687540	9425
**TMEM98**	0.50887	0.72168	0.02223556300637990	9426
**C22orf42**	0.49112	0.4503	0.02226063542135380	9427
**IMMP2L**	0.50896	0.2417	0.02246121524900840	9428
**CSH2**	0.50905	2.0403	0.02268686864155360	9429
**NOMO3**	0.49091	0.14263	0.02278715941034980	9430
**MRPS24**	0.50909	0.92969	0.02278715941035010	9431
**SESN1**	0.50919	0.85382	0.02303788734261880	9432
**EXOC3L1**	0.50927	0.66337	0.02323847073588660	9433
**LGALS9C**	0.49073	0.25125	0.02323847073588660	9434
**PRSS55**	0.49071	0.41284	0.02328861673073220	9435
**HSPA1L**	0.49065	0.50731	0.02343905506895400	9436
**DNAJC6**	0.50944	0.027949	0.02366471357704150	9437

**BRD8**	0.50947	0.73243	0.023739933348305900	9438
**DDX21**	0.49052	0.99354	0.02376500663537150	9439
**NISCH**	0.49049	1.5534	0.023840226586719500	9441
**PLIN1**	0.49049	1.8092	0.023840226586719500	9440
**WDR18**	0.49049	1.1476	0.023840226586719500	9442
**DARS**	0.49047	1.0173	0.023890373296236300	9443
**TET3**	0.49039	1.0459	0.02409096073930740	9444
**TMOD2**	0.50969	1.2172	0.024291549156427600	9445
**AHRR**	0.50985	0.22996	0.024692728945059700	9446
**MYL9**	0.50995	0.65759	0.024943468336518200	9447
**SMDT1**	0.48999	0.97207	0.025093912726616200	9448
**POU4F1**	0.51006	1.4022	0.025219283487408300	9449
**TMEM92**	0.5102	0.46127	0.025570323742331900	9450
**ZAP70**	0.51031	0.25344	0.025846143303732400	9451
**GRK1**	0.48968	0.34974	0.025871217906929100	9452
**PLEKHS1**	0.51037	0.016156	0.025996591168289200	9453
**DHX15**	0.51038	1.7701	0.0260216658697469	9455
**MKL2**	0.51038	1.3795	0.0260216658697469	9458
**OOEP**	0.51038	0.93985	0.0260216658697469	9460
**OR5B12**	0.51038	1.5071	0.0260216658697469	9456
**RPS6KA3**	0.51038	1.4699	0.0260216658697469	9457
**SFRP1**	0.51038	1.2709	0.0260216658697469	9459
**UBE2V1**	0.51038	1.8587	0.0260216658697469	9454
**CAMK4**	0.4896	0.54597	0.026071815321973400	9461
**RIOX2**	0.48948	1.1692	0.026372713422666700	9462
**FUT9**	0.48944	0.63312	0.026473013321096500	9463
**NEK2**	0.51057	0.091219	0.026498088337452900	9464
**MRGPRX1**	0.48941	1.1536	0.02654823842037500	9465
**PITPNB**	0.51075	0.57625	0.02694944150815600	9466
**RABGAP1**	0.51083	0.1334	0.027150044681419400	9467
**PRAMEF5**	0.48916	0.047058	0.027175120155044900	9468
**KLK6**	0.48911	0.69678	0.027300497780834000	9469

**INAFM1**	0.48909	0.30484	0.027350648951685300	9470
**LALBA**	0.48902	0.4082	0.027526178594392800	9471
**PHF24**	0.51106	0.58983	0.02772678494327660	9472
**TMEM33**	0.48893	0.86469	0.027751860815484700	9473
**HMGN2**	0.51108	1.124	0.0277769367052119	9474
**NPR 1.00**	0.48891	1.0794	0.02780201261247330	9475
**CCZ1B**	0.48884	0.31324	0.02797754445559410	9476
**BCKDHA**	0.51116	0.56804	0.02797754445559420	9477
**OTOGL**	0.48883	2.0927	0.028002620503698600	9478
**CUL9**	0.4887	0.89317	0.028328610743337400	9479
**CHODL**	0.48869	0.66342	0.02835368704034370	9480
**HNRNPA1**	0.48853	0.50164	0.028754910237137500	9481
**ABCA12**	0.51153	1.6685	0.028905370130301500	9483
**CHMP2B**	0.51153	1.3009	0.028905370130301500	9485
**CNTD2**	0.51153	1.8726	0.028905370130301500	9482
**DDX25**	0.51153	1.3966	0.028905370130301500	9484
**SLC22A6**	0.51153	1.0282	0.028905370130301500	9488
**SLC52A3**	0.51153	1.1097	0.028905370130301500	9487
**ZNF670**	0.51153	1.1186	0.028905370130301500	9486
**TSPAN33**	0.48834	0.070197	0.02923136882310890	9489
**PGAM1**	0.48829	0.89768	0.029356753764014100	9490
**DPH2**	0.51175	0.10015	0.029457062050134700	9491
**SASH3**	0.48819	1.5876	0.02960752503733630	9492
**TSPAN1**	0.48813	0.32756	0.029757988697192400	9493
**SOX12**	0.4881	0.094016	0.029833220780429100	9494
**ATXN3L**	0.48808	1.2305	0.029883375596694200	9501
**CORO1A**	0.48808	1.856	0.029883375596694200	9496
**FAM155B**	0.48808	1.0505	0.029883375596694200	9503
**HSPBP1**	0.48808	1.1617	0.029883375596694200	9502
**IL19**	0.48808	1.4098	0.029883375596694200	9499
**KCND1**	0.48808	1.9575	0.029883375596694200	9495
**MEAF6**	0.48808	1.6003	0.029883375596694200	9498

**NRGN**	0.48808	1.2554	0.029883375596694200	9500
**SHISA3**	0.48808	1.8282	0.029883375596694200	9497
**SLC16A13**	0.51194	0.66028	0.02993353048839270	9504
**ZBTB8OS**	0.48794	2.0289	0.03023446142973040	9505
**TMEM74B**	0.48793	0.13761	0.030259539131812500	9506
**TRHDE**	0.48791	0.68134	0.030309694593276100	9507
**RGS2**	0.48777	0.79965	0.030660784972715200	9508
**CCL1**	0.48773	2.0617	0.030761097204293700	9509
**EVPLL**	0.51227	0.47219	0.030761097204293700	9510
**SLC37A4**	0.5124	0.74976	0.031087114106950700	9511
**KRT14**	0.51242	0.19212	0.03113727084686980	9513
**PTPN13**	0.48758	0.54314	0.03113727084686980	9512
**C10orf12**	0.51261	1.8917	0.03161376381339430	9514
**CERS2**	0.51261	1.5692	0.03161376381339430	9515
**DCC**	0.51261	1.301	0.03161376381339430	9520
**EPB41L3**	0.51261	0.99567	0.03161376381339430	9522
**FOLH1**	0.51261	0.86885	0.03161376381339430	9523
**IL32**	0.51261	1.242	0.03161376381339430	9521
**KLF2**	0.51261	1.3706	0.03161376381339430	9518
**LTN1**	0.51261	1.3916	0.03161376381339430	9517
**PDZD3**	0.51261	1.511	0.03161376381339430	9516
**ZNF213**	0.51261	1.3444	0.03161376381339430	9519
**VN1R4**	0.48735	1.3558	0.03171407903602750	9524
**ACACB**	0.48733	1.1436	0.0317642367673358	9525
**MAX**	0.48733	0.98623	0.0317642367673358	9526
**CYP4A22**	0.48727	0.45239	0.031914710442742100	9527
**RLIM**	0.48709	1.2841	0.03236613583180810	9528
**SMIM12**	0.48707	0.91813	0.03241629461507260	9529
**EXO1**	0.48701	0.88283	0.032566771456186600	9530
**ANKRD36C**	0.51307	1.0996	0.03276740839546780	9531
**THAP1**	0.4869	1.6804	0.032842647588345200	9532
**RFWD3**	0.51327	0.34031	0.03326900654937310	9533

**C21orf58**	0.48669	0.90108	0.0333693271834941	9534
**RFPL1**	0.48665	0.1038	0.03346964815441720	9535
**STC2**	0.48659	0.27057	0.03362013024451600	9536
**ERICH4**	0.48643	0.26577	0.03402141955996500	9537
**CDKAL1**	0.48629	0.30367	0.03437255221545480	9538
**ALDOA**	0.51374	1.6123	0.03444779547874350(	9540
**DNAJC1**	0.51374	1.5549	0.03444779547874350(	9541
**FMNL1**	0.51374	1.1855	0.03444779547874350(	9545
**FRRS1**	0.51374	1.2957	0.03444779547874350(	9542
**ITPKB**	0.51374	1.2342	0.03444779547874350(	9543
**MAB21L1**	0.51374	0.1632	0.03444779547874350(	9548
**PRICKLE2**	0.51374	1.1857	0.03444779547874350(	9544
**SYF2**	0.51374	1.0843	0.03444779547874350(	9547
**TBCK**	0.51374	1.7448	0.03444779547874350(	9539
**TYW5**	0.51374	1.103	0.03444779547874350(	9546
**DEFB1**	0.48612	0.056404	0.03479893330002010(	9549
**DTNB**	0.48605	0.6667	0.03497450382124690	9550
**DCAF8L1**	0.51398	0.75376	0.03504974866092920(	9551
**TMEM176B**	0.4859	0.39184	0.03535073001359200	9552
**POLH**	0.48584	0.12474	0.0355012218922483	9553
**S100A7**	0.48583	0.82624	0.03552630395030350(	9554
**ADAMTS20**	0.48579	0.80086	0.03562663240675250	9555
**CT47A10**	0.48576	0.5927	0.0357018789849744	9556
**TTC8**	0.48572	0.61088	0.03580220807126040(	9557
**PSIP1**	0.48562	0.43728	0.03605303236985640	9558
**LARGE2**	0.51455	0.88698	0.0364794389044979	9559
**KAT6B**	0.48545	1.723	0.03647943890449820	9560
**AGPAT1**	0.48541	0.36227	0.03657977081725820	9561
**CRELD1**	0.48536	1.5402	0.03670518622741250(	9562
**TP53TG3F**	0.48533	0.72773	0.0367804357511718	9563
**CTDSP1**	0.51469	0.47721	0.03683060221630260	9564
**AIM2**	0.51484	1.95	0.03720685367512430	9565

**AP3B2**	0.51484	0.19292	0.03720685367512430	9572
**APOC2**	0.51484	1.8106	0.03720685367512430	9567
**FASTKD3**	0.51484	1.1589	0.03720685367512430	9571
**PLA2G5**	0.51484	1.5868	0.03720685367512430	9569
**POTEB3**	0.51484	1.6259	0.03720685367512430	9568
**SMIM2**	0.51484	1.8667	0.03720685367512430	9566
**SRD5A1**	0.51484	0.17245	0.03720685367512430	9573
**TMEM261**	0.51484	1.3187	0.03720685367512430	9570
**GPR1**	0.48512	1.1069	0.03730718828695720(	9574
**A2M**	0.48509	1.3972	0.03738243949282450	9582
**ARID5A**	0.48509	0.027428	0.03738243949282450	9579
**CGB8**	0.48509	1.3263	0.03738243949282450	9580
**KRTAP10-12**	0.48509	0.12931	0.03738243949282450	9575
**NBPF1**	0.48509	1.8511	0.03738243949282450	9577
**STMN2**	0.48509	1.4602	0.03738243949282450	9578
**WBP1**	0.48509	1.8267	0.03738243949282450	9576
**WDR26**	0.48509	0.068584	0.03738243949282450	9581
**RGS16**	0.48507	1.0152	0.0374326070812553	9583
**COA1**	0.48497	1.2067	0.03768344644238390	9584
**BUB1**	0.48485	0.72414	0.03798445681415690	9585
**NECAB2**	0.48483	0.54511	0.03803462554408460	9586
**RINT1**	0.48478	0.1072	0.03816004778894370	9587
**SPATA31D3**	0.48467	0.72326	0.03843597884817920	9588
**RPS15A**	0.48455	0.86062	0.0387369978949142	9589
**RPS25**	0.48452	0.83054	0.0388122532051819(	9590
**ZFP2**	0.48442	0.019993	0.0390631058327640(	9591
**ANKLE1**	0.48435	0.31307	0.03923870413687350	9592
**ZNF532**	0.51566	0.96768	0.0392637897077429	9593
**C1QL1**	0.48424	0.63302	0.0395146467808813	9594
**CACNA1C**	0.51594	1.6564	0.0399661958026883(	9597
**CR1L**	0.51594	1.5541	0.0399661958026883(	9598
**HOXA7**	0.51594	1.0495	0.0399661958026883(	9600

**KIF18A**	0.51594	1.1049	0.03996619580268830	9599
**LRCOL1**	0.51594	1.7762	0.03996619580268830	9596
**TOMM20**	0.51594	1.9453	0.03996619580268830	9595
**KDM7A**	0.51596	0.031098	0.04001636841860290	9601
**IL25**	0.48399	0.89722	0.04014180040027490	9602
**TBX4**	0.48399	0.46308	0.04014180040027490	9603
**TWIST2**	0.48375	0.24075	0.04074388275639840	9604
**FEZ2**	0.51633	0.64883	0.04094458015306460	9605
**GNAS**	0.48359	0.19378	0.04114527920220170	9606
**OGFOD1**	0.48355	0.48803	0.04124562934897700	9607
**PRDM5**	0.51652	1.023	0.04142124310781230	9608
**C1orf198**	0.48341	1.0598	0.04159685814654220	9609
**MSI1**	0.48335	2.2385	0.04174738634540070	9610
**MARCH5**	0.51682	0.51733	0.04217388806688430	9611
**DOCK7**	0.51688	0.34384	0.04232441991727030	9612
**RNF145**	0.4831	1.3095	0.04237459741408140	9613
**OARD1**	0.48304	1.8625	0.04252513054633170	9614
**ERVMER34-**	0.517	0.88524	0.04262548650394640	9615
**SLC5A12**	0.483	0.16903	0.04262548650394640	9616
**MAP3K11**	0.48297	0.29336	0.04270075375433650	9617
**FGL1**	0.48295	0.81341	0.04275093205587260	9618
**KCTD11**	0.5171	1.4682	0.04287637828176470	9622
**MAPK1IP1L**	0.5171	1.8151	0.04287637828176470	9620
**PLEKHG3**	0.5171	1.5286	0.04287637828176470	9621
**ZNF770**	0.5171	1.9009	0.04287637828176470	9619
**FAM24A**	0.48272	1.4306	0.04332799030598320	9623
**COL12A1**	0.4827	1.1243	0.04337816996454840	9624
**AK1**	0.48267	0.83227	0.04345343965759280	9625
**TMEM235**	0.48251	1.0735	0.04385488219597350	9626
**CEP89**	0.51756	0.4217	0.04403051552891160	9627
**CTIF**	0.48242	0.50831	0.04408069673083380	9628
**DEFB110**	0.5177	0.51046	0.04438178628177300	9629

**C17orf51**	0.48222	1.6849	0.04458251488636050	9631
**EP400**	0.48222	0.91057	0.04458251488636050	9634
**GABRQ**	0.48222	1.9564	0.04458251488636050	9630
**PTCRA**	0.48222	0.91879	0.04458251488636050	9633
**RNASEH2C**	0.48222	1.2613	0.04458251488636050	9632
**BCAP31**	0.48214	1.1866	0.04478324529023960	9635
**PACRGL**	0.482	0.84726	0.04513452785095310(	9636
**SCYL2**	0.51817	0.2631	0.04556109275007670(	9637
**TMEM219**	0.51821	1.0386	0.04566146216547350	9638
**MYO5A**	0.48179	0.49274	0.04566146216547370	9639
**HESX1**	0.51822	1.3431	0.04568655459127010(	9641
**NUP153**	0.51822	1.4474	0.04568655459127010(	9640
**CXCL6**	0.51824	0.561	0.04573673952931090	9642
**CDC37L1**	0.48169	2.0254	0.04591238772181710	9643
**PGA4**	0.48161	1.6678	0.04611313025075870(	9644
**SMC2**	0.48161	0.61848	0.04611313025075870(	9645
**RSRC2**	0.48156	0.43118	0.04623859527592760	9646
**PCGF1**	0.48152	1.2984	0.04633896782083080(	9647
**KRCC1**	0.48146	0.47793	0.04648952751517530	9648
**MRPS5**	0.48144	0.74635	0.04653971431432760	9649
**ZNF543**	0.48141	0.18935	0.04661499473321410	9650
**GSTCD**	0.48118	2.1416	0.04719215343252800	9651
**TRIM50**	0.51886	1.3119	0.04729253046017450(	9652
**ALPPL2**	0.48109	0.54856	0.0474180024159026	9653
**RIOK2**	0.48101	0.2102	0.0476187591008835	9654
**IKZF5**	0.48063	0.62856	0.04857237973968820	9655
**FLRT1**	0.48061	1.3046	0.04862257151732800	9656
**C9orf16**	0.51951	1.1393	0.04892372476342160(	9664
**ELK3**	0.48049	0.94107	0.04892372476342160(	9668
**ELL2**	0.51951	1.5198	0.04892372476342160(	9661
**GPR179**	0.51951	1.5987	0.04892372476342160(	9659
**H2AFY2**	0.51951	1.4695	0.04892372476342160(	9662

**HPGDS**	0.51951	0.96828	0.0489237247634216OO	9667
**KCNC1**	0.51951	1.5983	0.0489237247634216OO	9660
**MCUB**	0.51951	1.2356	0.0489237247634216OO	9663
**MTBP**	0.51951	0.98093	0.0489237247634216OO	9666
**NDUFA4L2**	0.51951	1.7157	0.0489237247634216OO	9657
**ROBO4**	0.51951	1.1387	0.0489237247634216OO	9665
**TCF12**	0.51951	1.6069	0.0489237247634216OO	9658
**USP10**	0.51951	0.72507	0.0489237247634216OO	9669
**HKDC1**	0.48033	0.91062	0.04932526934107720	9670
**C6orf132**	0.48029	0.062188	0.0494256567272923OO	9671
**GGACT**	0.48003	0.34748	0.0500781869400767OO	9672
**PCDHB7**	0.48	0.6789	0.05015348025633060	9673
**CCDC8**	0.47997	0.33867	0.05022877385727070	9674
**CLSTN2**	0.52021	0.84621	0.0506805414653135	9675
**MATR3**	0.47975	1.8208	0.05078093567030240	9676
**DPF3**	0.47961	1.7795	0.05113231943001060	9677
**RANGAP1**	0.5204	0.062785	0.05115741851135300	9678
**PALM3**	0.47946	1.5392	0.05150880904534990	9681
**SMAP1**	0.47946	1.6851	0.05150880904534990	9680
**SMR3B**	0.47946	2.0729	0.05150880904534990	9679
**SYNGR3**	0.47946	1.3655	0.05150880904534990	9682
**ATG16L1**	0.47941	0.63496	0.05163430720558350	9683
**AKR7A3**	0.47937	0.016452	0.0517347063198985	9684
**CAPN13**	0.52093	1.1739	0.0524877163894719OO	9691
**MANSC4**	0.52093	1.8724	0.0524877163894719OO	9687
**MGAT4C**	0.52093	1.3272	0.0524877163894719OO	9690
**NSF**	0.52093	2.0262	0.0524877163894719OO	9686
**PRICKLE1**	0.52093	1.7948	0.0524877163894719OO	9688
**TBC1D22B**	0.52093	2.0319	0.0524877163894719OO	9685
**WRAP73**	0.52093	1.5807	0.0524877163894719OO	9689
**CACNA2D3**	0.479	1.4549	0.05266342301072520	9692
**THTPA**	0.479	0.8171	0.05266342301072520	9693

**DAZ4**	0.47885	0.052627	0.05303994268722320	9694
**IL1A**	0.52118	0.5681	0.0531152475241470[illegible]	9695
**MED4**	0.47882	0.11803	0.0531152475241470[illegible]	9696
**CYP2C19**	0.47875	0.24389	0.05329095998370970	9697
**CDC42SE1**	0.47861	0.99214	0.05364238984981190	9698
**FABP9**	0.52145	1.1139	0.05379300467732060	9699
**HMGN4**	0.47855	1.3446	0.05379300467732090	9700
**UBE4B**	0.47852	1.7942	0.0538683125489634[illegible]	9701
**THBD**	0.52163	0.22852	0.05424485650321430	9702
**DNAH8**	0.52167	0.56348	0.05434526952247400	9703
**KATNAL1**	0.47828	0.42137	0.05447078656803750	9704
**PROSER1**	0.4782	0.27657	0.05467161562861170	9705
**MYBPC1**	0.5218	0.55404	0.0546716156286120[illegible]	9706
**NFE2L3**	0.47805	0.37193	0.05504817607304300	9707
**CAPG**	0.47802	0.57468	0.0551234890978674[illegible]	9708
**AXIN2**	0.47793	2.0619	0.0553494300519455	9709
**ZNF583**	0.52208	0.64531	0.05537453477678000	9710
**DMRTA1**	0.47776	1.0505	0.05577621513605010	9711
**OR4D11**	0.52226	1.6334	0.05582642581302460	9712
**PPP1R3G**	0.52226	1.2458	0.05582642581302460	9714
**SLITRK6**	0.52226	1.2517	0.05582642581302460	9713
**TP63**	0.52226	0.88784	0.05582642581302460	9715
**CDV3**	0.52236	0.58478	0.05607748131368580	9716
**ADAMTS19**	0.47757	0.31978	0.05625322226829690	9717
**NLRP12**	0.5226	0.14314	0.0566800289804817[illegible]	9718
**LRRIQ3**	0.52261	0.6128	0.05670513557875860	9719
**TBC1D8B**	0.4773	0.18699	0.0569310965751340[illegible]	9720
**ARHGAP36**	0.47727	0.19912	0.05700641755325430	9721
**REEP5**	0.47711	0.16258	0.05740813491419870	9722
**EVX2**	0.47708	0.19843	0.05748345794975800	9723
**C19orf43**	0.4769	1.5063	0.05793540303325080	9729
**DUSP11**	0.4769	1.7386	0.05793540303325080	9726

**F10**	0.4769	1.5342	0.05793540303325080	9728
**LGMN**	0.4769	1.6172	0.05793540303325080	9727
**NUP214**	0.4769	2.0504	0.05793540303325080	9724
**PRRC1**	0.4769	1.0714	0.05793540303325080	9731
**RBM15B**	0.4769	1.1027	0.05793540303325080	9730
**TFEC**	0.4769	2.0276	0.05793540303325080	9725
**TUFM**	0.4769	0.10442	0.05793540303325080	9732
**RAB37**	0.47688	0.98346	0.0579856198830780000	9733
**SCARF2**	0.52314	0.25708	0.0580358368792599000	9734
**ASCL4**	0.5232	1.9731	0.0581864887472041000	9735
**SEC61A2**	0.5232	1.9438	0.0581864887472041000	9736
**ZNF671**	0.5232	1.3834	0.0581864887472041000	9737
**PFDN5**	0.47673	1.2993	0.05836225093062210	9738
**HMGB1**	0.47668	0.6602	0.05848779645185090	9739
**ID3**	0.52361	0.83931	0.0592159787328685000	9740
**RHOV**	0.47624	0.67717	0.05959263704740180	9742
**TGFB3**	0.47624	0.78241	0.05959263704740180	9741
**GLP1R**	0.52381	0.79447	0.05971819169662850	9743
**BRD7**	0.52398	1.9206	0.06014508456338720	9744
**FER1L6**	0.52398	1.2417	0.06014508456338720	9747
**HKR1**	0.52398	1.6342	0.06014508456338720	9746
**KRT31**	0.52398	1.161	0.06014508456338720	9748
**RPS6KA6**	0.52398	1.7885	0.06014508456338720	9745
**TFE3**	0.52398	0.42453	0.06014508456338720	9749
**GCNA**	0.47593	0.20325	0.0603710911054082000	9750
**ERAS**	0.47586	1.2341	0.0605468761044939	9751
**AP5M1**	0.4756	0.47885	0.06119980825189280	9752
**CFAP36**	0.47552	0.46302	0.0614007156913496000	9753
**MRPS28**	0.47544	0.19966	0.06160162561108650	9754
**TJP3**	0.47542	0.44551	0.06165185347952100	9755
**ZC3H7A**	0.52472	1.0113	0.06200345292404450	9756
**COL6A6**	0.47523	2.191	0.06212902601096420	9757

**PTH1R**	0.52498	0.29798	0.06265644370538110	9758
**BAX**	0.52499	1.8932	0.06268155926758350	9759
**CSRP2**	0.52499	1.6741	0.06268155926758350	9760
**LAGE3**	0.52499	1.45	0.06268155926758350	9761
**C9orf62**	0.52506	0.59909	0.0628573693117804	9762
**NKX2-8**	0.4748	1.3231	0.06320899523846210	9763
**SRCAP**	0.47477	0.70485	0.06328434466734090	9764
**UGT1A6**	0.47474	0.50777	0.06335969445577060	9765
**MKRN3**	0.52533	0.60468	0.06353551202944760	9766
**C1orf27**	0.52534	0.46632	0.06356062898599870	9767
**MTMR2**	0.47462	0.75839	0.06366109721361790	9768
**C9orf106**	0.47453	1.6193	0.0638871530783986	9773
**CT62**	0.47453	1.8826	0.0638871530783986	9771
**FDCSP**	0.47453	1.8069	0.0638871530783986	9772
**IFNL2**	0.47453	1.4251	0.0638871530783986	9774
**LRRC72**	0.47453	0.92149	0.0638871530783986	9776
**MET**	0.47453	1.1578	0.0638871530783986	9775
**SAYSD1**	0.47453	1.9686	0.0638871530783986	9770
**SEMA3G**	0.47453	2.0076	0.0638871530783986	9769
**ATP6V1G3**	0.47438	0.5603	0.06426392011872410	9777
**SNCB**	0.47432	1.7289	0.06441462948789930	9778
**SH3GL2**	0.47424	1.5367	0.06461557759147660	9779
**PHKA2**	0.47414	1.0311	0.06486676639352950	9780
**CNN2**	0.47403	0.65286	0.06514307880675390	9781
**GAL3ST1**	0.47399	0.69396	0.06524355728073950	9782
**FBXL19**	0.47394	0.052067	0.06536915630022660	9783
**B9D2**	0.52608	1.1961	0.06541939619683250	9786
**CBLB**	0.52608	1.2065	0.06541939619683250	9785
**GOT1**	0.52608	1.2452	0.06541939619683250	9784
**LOC400927-**	0.52608	0.9393	0.06541939619683250	9787
**NOTCH2**	0.47391	0.17486	0.06544451620708720	9788
**UACA**	0.52616	0.77238	0.06562035743679990	9789

**CETN3**	0.52623	0.67321	0.06579620069679770	9790
**AK7**	0.47373	1.0026	0.06589668347331730	9791
**EMC8**	0.4737	0.96516	0.06597204599255690	9792
**FLVCR2**	0.47365	0.62693	0.0660976510246626	9793
**TIMM50**	0.47362	1.2448	0.06617301454465140	9794
**SCNN1G**	0.47357	0.24044	0.06629862124722770	9795
**OAZ3**	0.47354	0.33595	0.06637398577103280	9796
**UCHL3**	0.52647	0.50928	0.06639910736277420	9797
**GLB1L**	0.47336	1.8817	0.06682618085061590	9798
**ME2**	0.52697	0.21749	0.06765524074263270	9799
**ATP13A4**	0.47302	0.58187	0.06768036449314960	9800
**ADIPOQ**	0.52705	1.1704	0.06785623194450240	9804
**DENND4C**	0.52705	1.5335	0.06785623194450240	9802
**FAM83E**	0.52705	1.2506	0.06785623194450240	9803
**OSCP1**	0.52705	1.151	0.06785623194450240	9805
**ZNF479**	0.52705	1.5754	0.06785623194450240	9801
**KRT77**	0.47293	1.1489	0.06790648017321420	9806
**HIST1H3C**	0.47289	0.072702	0.0680069771454238	9807
**OXSM**	0.4728	1.5458	0.06823309784716050	9808
**FANCA**	0.47269	0.10888	0.06850947233547560	9809
**DHX36**	0.47267	0.37801	0.06855972280435460	9810
**MPV17**	0.52735	0.26991	0.06860997344644980	9811
**IMP3**	0.47255	1.8748	0.06886122925964280	9812
**PQLC2L**	0.52753	0.51607	0.06906223704113590	9813
**MTIF3**	0.47246	0.44926	0.06908736320996120	9814
**GAD1**	0.47242	0.13813	0.06918786832182430	9815
**ARRDC4**	0.47238	1.1767	0.06928837413294860	9816
**ERICH6B**	0.52773	0.077848	0.06956476872746680	9817
**NBPF26**	0.47217	0.12068	0.06981604115420530	9818
**FBLN1**	0.4721	1.1464	0.06999193447614710	9819
**SEMA3F**	0.47207	1.2051	0.07006731799132580	9820
**TEX36**	0.47204	1.0865	0.0701427019048789	9821

**GNG12**	0.52808	1.1125	0.07044424155147850	9822
**APPL1**	0.4719	2.2846	0.0704944987815529	9824
**CCNYL1**	0.4719	1.6285	0.0704944987815529	9827
**CLRN3**	0.4719	1.4849	0.0704944987815529	9829
**CPN2**	0.4719	2.2929	0.0704944987815529	9823
**LOC1001308**	0.4719	1.6053	0.0704944987815529	9828
**MLXIP**	0.4719	0.24608	0.0704944987815529	9835
**MRPS27**	0.4719	1.3688	0.0704944987815529	9830
**NFKBIL1**	0.4719	1.069	0.0704944987815529	9834
**OR4K15**	0.4719	1.2323	0.0704944987815529	9831
**PSAT1**	0.4719	1.1485	0.0704944987815529	9832
**S100A10**	0.4719	1.1168	0.0704944987815529	9833
**SLC22A7**	0.4719	2.2423	0.0704944987815529	9825
**ZHX2**	0.4719	2.0292	0.0704944987815529	9826
**ACYP1**	0.47188	1.2927	0.07054475618977060	9836
**CACNG8**	0.52821	0.8006	0.0707709167347143	9838
**GNB4**	0.52821	1.0417	0.0707709167347143	9837
**NDUFS1**	0.47168	1.888	0.07104734009803060	9839
**TIMM17A**	0.52843	0.65818	0.07132376889280940	9840
**PAQR4**	0.47147	0.51357	0.07157507252834220	9841
**NR1I2**	0.47144	0.25413	0.07165046450014950	9842
**SEMA4C**	0.47143	0.0028854	0.07167559524794210	9843
**LIN37**	0.47116	1.2281	0.07235414260986290	9844
**AP2B1**	0.47105	1.2455	0.07263059736921790	9845
**KIF24**	0.47095	0.79632	0.07288192469637460	9846
**ITGB8**	0.47069	1.0285	0.07353539734190650	9847
**KIAA1210**	0.47067	0.22436	0.07358566576617490	9848
**ERH**	0.47061	0.97359	0.07373647215564520	9849
**ARHGEF11**	0.47053	0.60575	0.0739375499519478	9850
**CXCR3**	0.52952	1.1879	0.07406322509286770	9854
**DPH5**	0.52952	1.3098	0.07406322509286770	9853
**MDH1B**	0.52952	1.4918	0.07406322509286770	9852

**SH2D5**	0.52952	2.074	0.07406322509286770	9851
**PPP6R3**	0.47047	0.92885	0.07408836026142040	9855
**BBC3**	0.52962	0.06957	0.07431457888753760	9856
**RRAS**	0.47017	0.15741	0.07484243716467570	9857
**EFCAB7**	0.52986	0.24328	0.07491784718961350	9858
**ATG4A**	0.47006	0.36756	0.07511894267430350	9860
**PROM1**	0.47006	0.67794	0.07511894267430350	9859
**HACD1**	0.47	0.88305	0.0752697662817328	9861
**C10orf67**	0.53039	0.80148	0.07625016163728330	9862
**TRIM28**	0.46953	0.91911	0.07645127740861880	9863
**ALDH18A1**	0.46944	1.5298	0.07667753634944730	9872
**CD63**	0.46944	1.8485	0.07667753634944730	9868
**CLPX**	0.46944	2.1649	0.07667753634944730	9865
**DLX4**	0.46944	2.1687	0.07667753634944730	9864
**ENOX2**	0.46944	1.8667	0.07667753634944730	9867
**FFAR3**	0.46944	1.4772	0.07667753634944730	9873
**PCDHGB4**	0.46944	1.5685	0.07667753634944730	9871
**POP4**	0.46944	1.7065	0.07667753634944730	9869
**SPINT4**	0.46944	1.6861	0.07667753634944730	9870
**ZFP90**	0.46944	1.9737	0.07667753634944730	9866
**CHD1L**	0.53074	1.4437	0.07713006602345480	9878
**DCAF4L2**	0.53074	1.7818	0.07713006602345480	9875
**FBXO41**	0.53074	1.3486	0.07713006602345480	9879
**ISL2**	0.53074	1.6652	0.07713006602345480	9877
**PTX3**	0.53074	0.3385	0.07713006602345480	9881
**SCN5A**	0.53074	1.7426	0.07713006602345480	9876
**SLC26A9**	0.53074	1.063	0.07713006602345480	9880
**SORT1**	0.53074	2.1041	0.07713006602345480	9874
**RBBP4**	0.46911	0.15635	0.07750718615151170	9882
**PRAC1**	0.46907	1.1824	0.07760775337913970	9883
**TOMM5**	0.46905	0.55175	0.07765803728733580	9884
**DUSP19**	0.46904	1.1096	0.07768317931508540	9885

**FAM173A**	0.46899	0.16456	0.07780889019099330	9886
**THBS1**	0.46892	0.69611	0.07798488748423750	9887
**TUBA3D**	0.53131	0.67202	0.07856318134829110	9888
**DEFB128**	0.46865	0.20337	0.07866375687171180	9889
**TM6SF1**	0.46862	0.90126	0.07873918903659090	9890
**RORB**	0.46858	0.15969	0.07883976595367140	9891
**PLA2G2C**	0.46839	0.54512	0.079317517222213	9892
**KRTAP21-2**	0.46833	0.68792	0.07946838980119520	9894
**OR13F1**	0.46833	0.709	0.07946838980119520	9893
**OR1D2**	0.4683	0.93327	0.07954382676903320	9895
**SDF2**	0.46828	2.0906	0.07959411833246040	9896
**ANAPC10**	0.46825	0.20146	0.07966955605522350	9897
**DSCR3**	0.46817	0.12679	0.07987072553438630	9898
**BEST3**	0.53186	0.92088	0.07994616492223270	9902
**C2orf15**	0.53186	1.1256	0.07994616492223270	9901
**MAP3K1**	0.53186	1.6196	0.07994616492223270	9899
**ZNF649**	0.53186	1.4809	0.07994616492223270	9900
**C2orf70**	0.46791	0.77281	0.08052454871479970	9903
**GABRA6**	0.46776	0.16407	0.08090177004164870	9904
**ZMYND19**	0.46774	1.0268	0.08095206708745790	9905
**CCR7**	0.4676	0.58772	0.08130415215123450	9906
**LIM2**	0.4676	0.53669	0.08130415215123450	9907
**FMNL3**	0.53241	1.0654	0.08132930146963	9908
**ENKD1**	0.46743	0.085274	0.08173169757277000	9909
**TPBGL**	0.53263	0.32454	0.0818825995209362	9910
**OR52E6**	0.46728	0.47115	0.08210895594139730	9911
**FCGR2B**	0.53281	0.32662	0.08233531657014950	9912
**SLC25A42**	0.46715	0.64593	0.08243592264792110	9913
**FAT1**	0.46712	0.60764	0.08251137775391140	9914
**HNMT**	0.46706	0.27275	0.08266228937605180	9915
**ZNF566**	0.46703	0.983	0.08273774589307120	9916
**CAMSAP2**	0.53297	1.4335	0.08273774589307150	9917

**CNGA3**	0.53297	1.3326	0.08273774589307150	9918
**TMEM56-RV**	0.53297	1.3073	0.08273774589307150	9919
**TTLL12**	0.53297	1.2733	0.08273774589307150	9920
**R3HDML**	0.46696	0.2852	0.08291381293269900	9921
**C4BPB**	0.4669	1.1012	0.08306472958420870	9928
**HOXA1**	0.4669	1.4068	0.08306472958420870	9926
**KCTD10**	0.4669	1.5565	0.08306472958420870	9923
**KIF14**	0.4669	1.5409	0.08306472958420870	9924
**POU2F2**	0.4669	1.8862	0.08306472958420870	9922
**RGPD3**	0.4669	1.3772	0.08306472958420870	9927
**TAL1**	0.4669	0.82963	0.08306472958420870	9929
**TMEM164**	0.4669	1.4429	0.08306472958420870	9925
**CYP2D6**	0.53315	0.5079	0.08319049490589930	9930
**C1orf146**	0.53319	0.76882	0.08329110811080340	9931
**JAG1**	0.53322	0.70894	0.08336656856789680	9932
**NRXN2**	0.46658	0.9156	0.08386965043766660	9933
**SNX7**	0.46655	0.70298	0.08394511454632490	9934
**FZD3**	0.46624	1.9664	0.08472493841508230	9935
**SYNDIG1**	0.46612	0.91182	0.08502681952953020	9936
**TMEM55A**	0.46601	0.90307	0.08530355069256500	9937
**C22orf39**	0.4659	1.5551	0.08558028838984930	9938
**DNAJB11**	0.5341	0.81868	0.08558028838984930	9939
**ATG10**	0.5342	0.90986	0.08583187380294920	9942
**GUCA1A**	0.5342	0.16588	0.08583187380294920	9943
**RAB26**	0.5342	1.4235	0.08583187380294920	9941
**UQCC3**	0.5342	1.7338	0.08583187380294920	9940
**PPEF1**	0.53424	0.75956	0.08593250948883550	9944
**ZBTB38**	0.46575	0.66755	0.0859576685462804	9945
**MRPL43**	0.46553	0.027715	0.08651118160738830	9946
**CLTC**	0.46547	1.1257	0.08666214430900260	9947
**PGS1**	0.46544	0.40606	0.08673762640039060	9949
**TAF7L**	0.46544	0.88618	0.08673762640039060	9948

**ABLIM1**	0.46537	0.18361	0.08691375320333730	9950
**CDK4**	0.46517	0.38922	0.08741698751883050	9951
**ARHGEF28**	0.46511	0.4065	0.087567962125577	9952
**PDHA2**	0.46502	0.84678	0.0877944277800482	9953
**FNBP1**	0.46475	0.49052	0.08847385181277890	9954
**ACTR6**	0.53542	0.81697	0.08890165825282370	9959
**CTSD**	0.53542	1.3692	0.08890165825282370	9956
**PCDHA10**	0.53542	0.97626	0.08890165825282370	9957
**SENP3**	0.53542	0.97164	0.08890165825282370	9958
**ZASP**	0.53542	1.5362	0.08890165825282370	9955
**HK2**	0.46456	1.3593	0.08895198949064520	9960
**TNFRSF21**	0.53547	0.53704	0.08902748677000590	9961
**LGALS4**	0.46448	0.64095	0.08915331669702700	9962
**PPP1R13B**	0.46439	0.6064	0.08937981412491700	9963
**GMPR2**	0.46438	1.6166	0.08940498078867730	9964
**PUS1**	0.46427	1.3461	0.08968181783160680	9965
**RYR2**	0.46427	0.94579	0.08968181783160680	9966
**ZNF280A**	0.46426	1.1142	0.08970698517607130	9967
**BAIAP2L1**	0.46407	2.2466	0.09018517553714180	9968
**CTAGE15**	0.46407	1.7637	0.09018517553714180	9971
**IMPG2**	0.46407	1.1197	0.09018517553714180	9973
**LYSMD2**	0.46407	1.8422	0.09018517553714180	9970
**NEUROD4**	0.46407	0.057967	0.09018517553714180	9974
**TM2D3**	0.46407	1.3439	0.09018517553714180	9972
**ZNF737**	0.46407	1.8832	0.09018517553714180	9969
**ZNF674**	0.46392	0.85375	0.09056270880794400	9975
**UBIAD1**	0.46384	1.2737	0.0907640651612724	9976
**SRA1**	0.4636	1.1291	0.09136815633844520	9977
**TCN1**	0.46356	1.0033	0.0914688414376163	9978
**ACADS**	0.53647	0.35428	0.09154435587056050	9979
**CNBD2**	0.46351	1.1801	0.09159469911589230	9980
**ANGPT4**	0.4635	0.44489	0.09161987082561790	9981

**FAM19A3**	0.46347	0.8013	0.0916953863032276	9982
**CSTF1**	0.46346	0.39139	0.09172055824529510	9983
**GPR108**	0.53661	1.6937	0.09189676346820830	9987
**HIST1H4I**	0.53661	0.53667	0.09189676346820830	9990
**MAGED2**	0.53661	1.2892	0.09189676346820830	9988
**MCM3**	0.53661	0.69562	0.09189676346820830	9989
**PIK3R6**	0.53661	1.7956	0.09189676346820830	9984
**TMEM2**	0.53661	1.7383	0.09189676346820830	9985
**VARS2**	0.53661	1.7291	0.09189676346820830	9986
**FUCA1**	0.46334	1.2869	0.09202262608858030	9991
**PSMC4**	0.46328	0.19976	0.09217366315778230	9992
**MAPK12**	0.46325	1.4154	0.09224918248080580	9993
**C14orf39**	0.4632	1.112	0.09237504918872260	9994
**CARD11**	0.53686	0.27793	0.09252609117047040	9995
**USP21**	0.53696	0.56139	0.09277783250003080	9996
**LRRC6**	0.46289	1.6086	0.09315545552574420	9997
**SCLY**	0.46288	0.85486	0.0931806308659218	9998
**TSPO2**	0.53731	1.0413	0.09365897357041300	9999
**NOVA2**	0.46258	0.35375	0.093935918610073	10000
**BEST4**	0.53745	0.13837	0.0940114503251427	10001
**ZGPAT**	0.46249	0.58422	0.09416251536497350	10002
**SLC4A3**	0.46246	2.1944	0.09423804869061060	10003
**KIAA0100**	0.46237	1.396	0.09446465189566670	10004
**ATP8A2**	0.46225	1.3131	0.09476679705111140	10005
**MROH1**	0.46218	0.77977	0.0949430523859021	10006
**ATRAID**	0.46207	1.4478	0.09522003101706310	10007
**CSTA**	0.53801	1.0362	0.09542147460928620	10012
**DCPS**	0.53801	1.5289	0.09542147460928620	10009
**DRD4**	0.53801	1.577	0.09542147460928620	10008
**SAPCD1**	0.53801	1.1825	0.09542147460928620	10011
**TUBA3E**	0.53801	1.387	0.09542147460928620	10010
**DNAI1**	0.46188	0.20559	0.09569846587371620	10014

**SOWAHC**	0.46188	0.29568	0.09569846587371620	10013
**TEX28**	0.46184	1.2547	0.09579919178943390	10015
**CEP41**	0.46156	1.5351	0.09650430047592000	10016
**CCDC157**	0.46154	0.57561	0.09655466721417350	10017
**CHRDL2**	0.53852	0.13689	0.09670576889927380	10018
**GALNT9**	0.46131	1.4411	0.0971339023440417	10019
**SCHIP1**	0.46126	0.4951	0.09725982733077760	10020
**TRPV4**	0.46122	0.37781	0.09736056843063150	10021
**ACAP2**	0.46112	2.2905	0.09761242550595140	10022
**ACKR2**	0.46112	1.3657	0.09761242550595140	10030
**ATPIF1**	0.46112	1.5299	0.09761242550595140	10028
**FAM167A**	0.46112	1.3587	0.09761242550595140	10031
**FRK**	0.46112	1.887	0.09761242550595140	10024
**HOXB7**	0.46112	1.2595	0.09761242550595140	10032
**KLHL14**	0.46112	1.4523	0.09761242550595140	10029
**LRRTM4**	0.46112	0.70643	0.09761242550595140	10034
**NAA60**	0.46112	1.6265	0.09761242550595140	10027
**PCYT1B**	0.46112	1.7712	0.09761242550595140	10025
**RHPN2**	0.46112	2.2616	0.09761242550595140	10023
**TRAPPC9**	0.46112	1.2229	0.09761242550595140	10033
**ZFP69**	0.46112	1.7524	0.09761242550595140	10026
**SLFN12L**	0.46109	0.026269	0.09768798383535780	10035
**OTUD7A**	0.46103	0.28132	0.09783910216791970	10036
**FYB**	0.53901	0.18813	0.09793984896418580	10037
**TRAM2**	0.46083	0.21396	0.09834284610161710	10038
**COX5B**	0.53927	1.1851	0.09859472741996820	10044
**DEFB125**	0.53927	1.4099	0.09859472741996820	10041
**DIAPH1**	0.53927	1.3044	0.09859472741996820	10043
**GABRE**	0.53927	0.85382	0.09859472741996820	10045
**PCLO**	0.53927	0.67287	0.09859472741996820	10046
**SETD3**	0.53927	1.5232	0.09859472741996820	10040
**SOAT1**	0.53927	1.6482	0.09859472741996820	10039

**SORBS1**	0.53927	0.22014	0.09859472741996820	10047
**VSTM1**	0.53927	1.3664	0.09859472741996820	10042
**OR8H3**	0.46071	1.5755	0.0986451044338426	10048
**RAB3C**	0.46071	0.88227	0.0986451044338426	10049
**APOBR**	0.46067	1.3556	0.09874585921283640	10050
**TMEM232**	0.46038	0.52067	0.0994763614073732	10051
**FAM210B**	0.46036	0.07676	0.09952674282156380	10052
**ERAP2**	0.53979	0.49623	0.0999046114902742	10053
**RELL2**	0.45994	0.57699	0.10058481108291400	10054
**GUCA1C**	0.45971	0.51033	0.10116427706535200	10055
**RP2**	0.45967	0.20666	0.10126505722039000	10056
**TAS2R30**	0.54034	0.1495	0.10129025241982900	10057
**EGFR**	0.45964	1.9396	0.10134064301163400	10058
**TIPRL**	0.45964	0.44536	0.10134064301163400	10059
**AGO2**	0.45959	0.4605	0.10146662061732600	10060
**MAOB**	0.54044	0.7781	0.10154220795363000	10062
**PTGFR**	0.45956	1.004	0.10154220795363000	10061
**AMTN**	0.5405	0.94354	0.1016933843672940	10067
**ANGPT1**	0.5405	1.6315	0.1016933843672940	10063
**CDK9**	0.5405	0.96321	0.1016933843672940	10066
**KCNAB3**	0.5405	1.5275	0.1016933843672940	10065
**SMURF1**	0.5405	1.5744	0.1016933843672940	10064
**DDRGK1**	0.45942	1.7908	0.10189495653510500	10068
**NBEA**	0.45939	1.5848	0.10197054716506300	10069
**NKX1-1**	0.45938	0.37476	0.1019957441711840	10070
**INSL3**	0.45929	0.85662	0.10222252014247000	10071
**CHKA**	0.45925	1.123	0.10232331114993900	10072
**FAM124B**	0.4592	1.2576	0.10244930137129400	10073
**DPPA2**	0.45914	0.90115	0.1026004917839060	10074
**LRP10**	0.54087	0.16582	0.1026256904139480	10075
**SGTA**	0.45901	1.9781	0.10292807906230100	10076
**LRAT**	0.45897	0.77914	0.10302887736870600	10077

**CRYGC**	0.45889	0.17367	0.1032304771232180	10078
**ETAA1**	0.54119	0.34849	0.10343208107363500	10080
**TOGARAM2**	0.45881	1.7783	0.10343208107363500	10079
**ITGA6**	0.4588	0.13424	0.10345728186287900	10081
**LOC339862**	0.45868	1.363	0.10375969646369700	10082
**LUC7L2**	0.45857	0.68214	0.10403691818396200	10083
**CDK11B**	0.45845	0.35021	0.10433935099912400	10084
**AGR2**	0.54189	2.0532	0.10519629590017300	10085
**RABEP2**	0.54189	1.4269	0.10519629590017300	10086
**SART3**	0.54189	0.78093	0.10519629590017300	10088
**TATDN3**	0.54189	0.61827	0.10519629590017300	10089
**TPM2**	0.54189	0.97721	0.10519629590017300	10087
**COQ3**	0.45809	1.2681	0.10524670682358000	10090
**SBK2**	0.45801	1.0291	0.10544835319327200	10091
**ANKS1B**	0.45789	2.1065	0.10575083079038400	10092
**CDC26**	0.45789	1.596	0.10575083079038400	10094
**CGB5**	0.45789	1.4444	0.10575083079038400	10095
**OR2AK2**	0.45789	1.0692	0.10575083079038400	10096
**PRKCZ**	0.45789	1.9455	0.10575083079038400	10093
**SUPT16H**	0.45789	0.048256	0.10575083079038400	10097
**RBMY1E**	0.45782	0.27843	0.10592728052208700	10098
**TXK**	0.45777	1.1565	0.10605331806372200	10099
**PCGF6**	0.45773	0.92543	0.10615414930999400	10100
**MDH2**	0.45767	0.38126	0.1063053982034670	10101
**FAM46A**	0.45766	0.069518	0.10633060658873000	10102
**PRKAR2B**	0.45764	0.39575	0.10638102356199000	10103
**TERB2**	0.45758	0.75132	0.10653227610481900	10104
**RETN**	0.45752	0.02618	0.10668353108497300	10105
**LURAP1L**	0.54256	0.99605	0.10688520818935200	10106
**SLC35F1**	0.5426	0.81171	0.10698604837140400	10107
**ART3**	0.54272	0.47175	0.10728857544958500	10108
**USO1**	0.4572	0.04029	0.10749026562221300	10109

**FAM25G**	0.45709	0.71486	0.10776759675134900	10110
**FAM229B**	0.45703	0.23121	0.10791887176875400	10111
**KLHL35**	0.54302	0.38297	0.10804493616973400	10113
**PIP5K1A**	0.45698	0.71066	0.1080449361697340	10112
**LOXL1**	0.45693	1.6605	0.10817100228787700	10114
**CNTFR**	0.54315	1.1705	0.1083727116537170	10118
**MADCAM1**	0.54315	1.5519	0.1083727116537170	10115
**MARCH8**	0.54315	1.1908	0.1083727116537170	10117
**OR52K1**	0.54315	1.3411	0.1083727116537170	10116
**PSMA7**	0.54315	1.0557	0.1083727116537170	10119
**RFX2**	0.54315	0.91993	0.1083727116537170	10120
**GID8**	0.45683	0.82561	0.10842313968384700	10121
**IQCE**	0.45678	0.68236	0.10854921096577300	10122
**COL2A1**	0.54328	0.28172	0.10870049878174100	10123
**OGFR**	0.45647	1.24	0.10933089150309300	10124
**CWF19L1**	0.45643	1.0435	0.10943175836418000	10125
**SCARB1**	0.45643	0.46176	0.10943175836418000	10126
**HERC5**	0.45627	0.40731	0.10983523695321100	10127
**BST2**	0.45616	0.84842	0.11011263885201500	10128
**GRXCR1**	0.45611	2.0606	0.11023873342415700	10129
**SFXN4**	0.45608	0.54711	0.11031439100867900	10130
**SLC25A29**	0.45592	1.8613	0.11071790880174700	10131
**CD93**	0.45588	0.40294	0.11081879106442500	10132
**EPOP**	0.45576	1.594	0.11112144462386600	10133
**IRX4**	0.45574	1.3359	0.1111718878726500	10136
**KIAA1462**	0.54426	1.5588	0.11117188787265	10135
**MX1**	0.54426	1.7682	0.11117188787265	10134
**OR5B3**	0.54426	1.2317	0.11117188787265	10138
**SFT2D2**	0.54426	1.2495	0.11117188787265	10137
**CHAF1B**	0.45558	1.8624	0.11157544405789100	10139
**PPP1R16A**	0.45556	0.8784	0.11162588985739000	10140
**LOR**	0.45555	0.017321	0.111651112863657	10141

**RIMKLB**	0.45553	0.7068	0.11170155908931000	10142
**RNF157**	0.45526	2.371	0.11238261101195000	10143
**STX19**	0.45526	0.34043	0.11238261101195000	10144
**RUBCNL**	0.45521	1.158	0.11250873744958500	10145
**KRT38**	0.45506	0.81631	0.11288712750968200	10146
**ZNF444**	0.54494	0.33337	0.11288712750968200	10147
**CLINT1**	0.54502	0.11908	0.11308894214747100	10148
**KRTAP16-1**	0.45496	1.2875	0.11313939652630500	10149
**ADAR**	0.45495	2.1678	0.11316462382372000	10151
**C16orf46**	0.45495	1.5776	0.11316462382372000	10155
**CCDC39**	0.45495	0.71144	0.11316462382372000	10165
**DDX58**	0.45495	1.3299	0.11316462382372000	10159
**EIF3B**	0.45495	2.1884	0.11316462382372000	10150
**HAS3**	0.45495	2.1484	0.11316462382372000	10152
**MARVELD2**	0.45495	1.1448	0.11316462382372000	10163
**MME**	0.45495	1.332	0.11316462382372000	10158
**MT3**	0.45495	0.95316	0.11316462382372000	10164
**NLGN1**	0.45495	1.7144	0.11316462382372000	10154
**OR4C6**	0.45495	1.2183	0.11316462382372000	10162
**RBM26**	0.45495	1.3218	0.11316462382372000	10160
**RNF165**	0.45495	1.4709	0.11316462382372000	10157
**RNF208**	0.45495	1.5741	0.11316462382372000	10156
**SLC22A8**	0.45495	1.2513	0.11316462382372000	10161
**TMLHE**	0.45495	1.7561	0.11316462382372000	10153
**COL7A1**	0.45492	0.46787	0.11324030614815700	10166
**HSF1**	0.4548	0.39949	0.11354304193667500	10168
**MPRIP**	0.4548	0.44228	0.11354304193667500	10167
**FAM210A**	0.45479	0.77067	0.11356827038840000	10169
**MMEL1**	0.45475	1.107	0.11366918491830800	10170
**APEH**	0.54528	1.4105	0.11374487157535800	10172
**BDH1**	0.54528	1.2252	0.11374487157535800	10173
**CACNA2D2**	0.54528	0.78553	0.11374487157535800	10175

**FASTK**	0.54528	0.67596	0.11374487157535800	10176
**KRT35**	0.54528	0.57389	0.11374487157535800	10177
**LACTB**	0.54528	1.0936	0.11374487157535800	10174
**PLEKHH1**	0.54528	1.7399	0.11374487157535800	10171
**CR2**	0.54535	0.21937	0.11392147630989400	10178
**RGL1**	0.45447	0.85182	0.11437561909954900	10179
**BCR**	0.45426	0.32266	0.11490548217712500	10180
**DCAKD**	0.45413	1.1186	0.11523350881178100	10181
**NCOA6**	0.45411	1.647	0.11528397554754900	10182
**ETF1**	0.45402	0.81107	0.11551107949394	10183
**OLFML2B**	0.45398	1.1285	0.11561201649288700	10184
**GPN3**	0.45393	0.37029	0.115738189398122	10185
**MTX3**	0.54619	0.47102	0.11604101189215400	10186
**CST3**	0.54633	1.7312	0.11639431825353900	10187
**HARS2**	0.54633	0.86503	0.11639431825353900	10190
**TPM1**	0.45367	0.93744	0.11639431825353900	10189
**TSSK1B**	0.54633	1.1961	0.11639431825353900	10188
**SEC31A**	0.45363	0.80485	0.11649526559493300	10191
**DPH7**	0.45326	0.2553	0.117429084918453	10192
**NATD1**	0.45318	0.29085	0.11763100523577100	10194
**OR10J3**	0.45318	0.45225	0.11763100523577100	10193
**OR5K4**	0.45313	0.58632	0.11775720786908600	10195
**KCNB1**	0.54692	1.2482	0.11788341237794700	10196
**LRRC37A2**	0.45306	0.07358	0.11793389470710800	10197
**COL9A1**	0.54702	0.70707	0.11813582703057300	10199
**DCTD**	0.54702	0.042914	0.11813582703057300	10200
**TM4SF20**	0.54702	1.1223	0.11813582703057300	10198
**FAM206A**	0.4529	1.6746	0.11833776417129900	10201
**KIF22**	0.45286	0.053148	0.11843873455078000	10202
**ARID4B**	0.45279	1.95	0.11861543562102700	10203
**MRNIP**	0.45279	0.89919	0.11861543562102700	10204
**LGALS12**	0.4527	0.19468	0.11884262815416800	10205

**KRTAP1-1**	0.45264	0.50744	0.11899409325014900	10206
**OIP5**	0.4526	1.8658	0.11909507149721800	10207
**ST3GAL2**	0.5476	1.5674	0.11959998096923000	10208
**TNFRSF13C**	0.5476	1.5578	0.11959998096923000	10209
**SCAND1**	0.45235	0.34334	0.11972621309636000	10210
**CCSER2**	0.45232	1.71	0.11980195328824200	10212
**DNAAF5**	0.45232	1.8409	0.11980195328824200	10211
**GADD45G**	0.45232	1.1326	0.11980195328824200	10214
**MAGEA9B**	0.45232	1.5731	0.11980195328824200	10213
**DEFB131**	0.45229	1.1731	0.11987769416737600	10215
**WNT9A**	0.45221	0.51537	0.12007967320835000	10216
**PIGB**	0.45216	0.37105	0.12020591259607200	10217
**EIF1**	0.45207	0.59395	0.12043314832279700	10218
**WWP1**	0.45206	0.87484	0.12045839712053100	10219
**MCM7**	0.45196	1.0934	0.12071088932413600	10220
**TIMM8A**	0.45195	0.62279	0.12073613896748700	10221
**RNF187**	0.45193	1.4344	0.12078663848512600	10222
**PSME4**	0.45191	1.4269	0.12083713831079300	10223
**LOC730183**	0.45185	0.35229	0.12098863963727900	10224
**EIF4B**	0.5482	0.3782	0.12111489286386	10225
**TP53TG3C**	0.45174	0.6521	0.12126639928426800	10226
**C18orf65**	0.45168	0.63124	0.1214179084882470	10227
**PF4V1**	0.45143	1.5982	0.12204922687942400	10228
**ATP6V1C2**	0.54872	0.97508	0.12242804124812500	10231
**CDH15**	0.54872	0.85196	0.12242804124812500	10232
**DYNC1LI2**	0.54872	1.2406	0.12242804124812500	10230
**MRC1**	0.54872	1.3382	0.12242804124812500	10229
**PLCXD3**	0.54872	0.17462	0.12242804124812500	10233
**SFRP2**	0.45128	0.14917	0.12242804124812500	10234
**BIVM**	0.45125	1.0514	0.12250380622834100	10235
**JMY**	0.45116	0.85834	0.1227311053900010	10236
**ADAM19**	0.54917	0.34172	0.12356458999152300	10237

**PCSK7**	0.54922	1.0819	0.12369088301140000	10238
**ZSCAN22**	0.45069	0.058271	0.1239182154201310	10239
**LARP4B**	0.54935	0.56978	0.124019254101154	10241
**PPP1R14A**	0.45065	1.9855	0.124019254101154	10240
**CBFA2T3**	0.45056	0.86678	0.12424659576415000	10242
**C11orf86**	0.45055	0.79152	0.12427185634510100	10243
**ALDH3B2**	0.45053	2.153	0.12432237774490600	10244
**ARC**	0.45051	0.026563	0.1243728994620250	10245
**NYAP1**	0.54956	0.33813	0.12454972797272000	10246
**SPEG**	0.45032	1.7746	0.12485287162616700	10247
**KRT23**	0.45028	0.64241	0.12495392205891800	10249
**LRP5**	0.54972	0.83459	0.12495392205891800	10248
**THOC6**	0.45016	1.1397	0.12525708101686800	10250
**ACER1**	0.54988	0.9775	0.12535813655963000	10259
**C5orf64**	0.54988	0.7071	0.12535813655963000	10261
**CYP20A1**	0.54988	0.54031	0.12535813655963000	10262
**EZH1**	0.54988	1.7046	0.12535813655963000	10252
**FOXP4**	0.54988	1.2338	0.12535813655963000	10257
**KIAA1551**	0.54988	0.84695	0.12535813655963000	10260
**MIF4GD**	0.54988	1.0509	0.12535813655963000	10258
**P2RX2**	0.54988	1.9666	0.12535813655963000	10251
**RPL26**	0.54988	1.4087	0.12535813655963000	10255
**STARD13**	0.54988	1.6216	0.12535813655963000	10254
**TIMM10**	0.54988	1.3133	0.12535813655963000	10256
**USP42**	0.54988	1.6262	0.12535813655963000	10253
**C11orf97**	0.54989	0.44555	0.12538340064530400	10263
**CELF6**	0.44995	1.0564	0.12578763691104300	10264
**ZCCHC9**	0.55009	0.63223	0.12588869918653200	10265
**TTC36**	0.44982	0.58416	0.12611609400902700	10266
**AGAP1**	0.44974	1.6339	0.12631822821437700	10271
**ATAD3A**	0.44974	2.1838	0.12631822821437700	10267
**COASY**	0.44974	1.0043	0.12631822821437700	10273

**HSFY2**	0.44974	1.6729	0.12631822821437700	10270
**IL10RA**	0.44974	1.8392	0.12631822821437700	10268
**IRX6**	0.44974	1.4843	0.12631822821437700	10272
**KNCN**	0.44974	0.027855	0.12631822821437700	10275
**KPNA1**	0.44974	0.54033	0.12631822821437700	10274
**OR4P4**	0.44974	1.7233	0.12631822821437700	10269
**CKS2**	0.44965	0.72276	0.12654563536495000	10276
**MAP2K5**	0.55036	0.2398	0.1265709032298630	10277
**C12orf42**	0.44958	0.78326	0.12672251211690500	10278
**PDZK1IP1**	0.44943	0.54465	0.12710154708546600	10279
**TSPAN31**	0.44934	0.62907	0.12732897682809800	10280
**TMEM167B**	0.44932	0.55252	0.1273795176648670	10281
**TTLL7**	0.44929	0.17123	0.12745532953012300	10282
**NCAM1**	0.44917	2.0537	0.1277585843208720	10283
**OPN3**	0.44917	0.44687	0.1277585843208720	10284
**STRADB**	0.44915	0.3494	0.12780912792749700	10285
**ACD**	0.4491	0.52954	0.12793548837278400	10287
**CABP4**	0.4491	1.2014	0.12793548837278400	10286
**C2CD4B**	0.44903	0.39938	0.12811239642838700	10288
**OVOL3**	0.44897	1.4921	0.1282640350955610	10289
**CATSPERD**	0.44894	0.8143	0.1283398555349060	10290
**DTYMK**	0.44891	1.433	0.12841567671202200	10291
**ADAMTSL3**	0.55114	1.4283	0.12854204698126400	10295
**CTR9**	0.55114	1.1811	0.12854204698126400	10297
**EGLN3**	0.55114	1.0884	0.12854204698126400	10301
**GLRX2**	0.55114	1.0007	0.12854204698126400	10303
**KRTAP29-1**	0.55114	1.1067	0.12854204698126400	10300
**METRNL**	0.55114	1.067	0.12854204698126400	10302
**NAP1L3**	0.55114	1.7561	0.12854204698126400	10293
**NLRP9**	0.55114	1.1663	0.12854204698126400	10299
**PSD3**	0.55114	1.8273	0.12854204698126400	10292
**PSD4**	0.55114	1.4122	0.12854204698126400	10296

**SERPINH1**	0.55114	1.6721	0.12854204698126400	10294
**ZC3H10**	0.55114	1.1799	0.12854204698126400	10298
**RRP15**	0.44879	1.0542	0.12871896880720600	10304
**MAGEB1**	0.44875	0.44996	0.12882006880203600	10305
**SRP9**	0.55146	0.60031	0.12935086540120100	10306
**OSGIN2**	0.5515	0.14757	0.1294519736426890	10307
**BTBD3**	0.55157	0.82865	0.12962891625020800	10308
**CXCL14**	0.44842	1.4585	0.1296541940967360	10309
**AR**	0.44835	0.77065	0.12983114134293900	10310
**ZSCAN23**	0.44832	0.34914	0.12990697712107500	10311
**SMCP**	0.44815	1.169	0.13033672732193500	10312
**YIPF5**	0.44808	0.78552	0.13051369027890800	10313
**ARRB1**	0.55196	0.059734	0.13061481380325200	10315
**PPP1R3C**	0.44804	2.2217	0.13061481380325200	10314
**CYR61**	0.44796	0.47281	0.13081706485988000	10316
**OR10H5**	0.44793	1.0789	0.13089291038522300	10317
**SLC25A39**	0.44782	0.36578	0.13117101709008100	10318
**PQLC1**	0.44778	0.070854	0.13127214931509000	10319
**RBPMS2**	0.44769	0.80979	0.13149970173177700	10320
**PPP4R1**	0.44768	1.6711	0.13152498575371500	10321
**PRRG1**	0.55237	0.47253	0.13165140712491000	10322
**MRPS6**	0.44761	0.96273	0.13170197626240200	10323
**PTGS2**	0.55242	0.45828	0.1317778306001450	10324
**MAGT1**	0.44757	0.83715	0.13180311554786200	10325
**PI3**	0.44755	1.8477	0.13185368569609700	10326
**GABRB2**	0.55252	1.2286	0.13203068387110200	10330
**NAT10**	0.55252	1.2426	0.13203068387110200	10329
**PIM1**	0.55252	1.0911	0.13203068387110200	10331
**RBM41**	0.55252	1.397	0.13203068387110200	10328
**VAMP1**	0.55252	1.4582	0.13203068387110200	10327
**SST**	0.44739	1.6964	0.13225825903175400	10332
**C14orf180**	0.4473	0.11863	0.13248584104201300	10333

**TNP2**	0.44713	2.2069	0.13291573690407100	10334
**TRMT1**	0.44706	0.78789	0.13309275998242800	10335
**RTN4**	0.447	2.1869	0.13324449736886900	10336
**MATN3**	0.44694	0.35681	0.13339623782307900	10337
**CLTA**	0.44687	1.0003	0.13357327223513300	10338
**OR5H6**	0.55313	0.80953	0.13357327223513300	10339
**MCFD2**	0.55318	0.39663	0.13369972794927000	10340
**ABCA6**	0.44676	0.86562	0.1338514776284860	10341
**NMS**	0.44676	0.47986	0.1338514776284860	10342
**IRS4**	0.44644	0.4605	0.13466086139485900	10344
**MRPL30**	0.44644	1.1324	0.13466086139485900	10343
**SNAPC4**	0.55359	0.65779	0.13473674563753800	10345
**SOS 2**	0.5538	0.84891	0.13526795708514000	10347
**DNAAF3**	0.5538	1.4656	0.13526795708514000	10350
**LRCH3**	0.5538	1.0166	0.13526795708514000	10349
**MMAA**	0.5538	1.2882	0.13526795708514000	10348
**PAK6**	0.5538	1.3223	0.13526795708514000	10346
**PRR27**	0.5538	1.5297	0.13526795708514000	10351
**ZNF330**	0.4461	1.505	0.13552092832406700	10352
**MYOZ3**	0.44606	0.68068	0.13562211924698000	10353
**FGF20**	0.44602	0.89909	0.1357233115585550	10354
**DDX31**	0.55403	0.37287	0.13584980390247000	10355
**BCL7C**	0.44596	0.40003	0.13587510263201300	10356
**VPS41**	0.44584	0.66062	0.1361786941741580	10357
**EVI2A**	0.4458	1.6174	0.13627989414258400	10358
**TOX**	0.4458	0.82804	0.13627989414258400	10359
**DISP1**	0.44573	1.2439	0.1364569974462520	10360
**USP7**	0.44563	0.91258	0.13671000959156900	10361
**NRBP2**	0.4454	0.077742	0.1372919707647750	10362
**ABLIM3**	0.44526	0.49487	0.13764623075365900	10363
**TRIM43**	0.44523	1.876	0.13772214585429600	10364
**GRM1**	0.4452	1.4753	0.13779806174860500	10365

**AQP6**	0.55521	1.1619	0.13883565875200100	10366
**DGKB**	0.55521	1.1132	0.13883565875200100	10367
**EFHD1**	0.55521	1.0765	0.13883565875200100	10368
**LRRC24**	0.55521	0.95978	0.13883565875200100	10369
**LRRC8A**	0.55521	0.92005	0.13883565875200100	10370
**PRKCD**	0.55521	0.86765	0.13883565875200100	10371
**SGCA**	0.55521	0.3157	0.13883565875200100	10372
**FST**	0.44478	1.7678	0.13886096785871800	10373
**ARHGAP40**	0.44474	0.30192	0.13896220517518600	10374
**C10orf105**	0.44468	1.0027	0.13911406382052600	10375
**E4F1**	0.55542	0.32662	0.13936716869344700	10376
**CACHD1**	0.44455	0.76912	0.13944310189550800	10377
**VSTM2A**	0.44449	0.44786	0.1395949707119930	10378
**STH**	0.44446	2.2579	0.13967090632732800	10379
**MED27**	0.44437	2.0746	0.1398987180071620	10380
**HBS1L**	0.44436	0.3873	0.13992403086397400	10381
**FUK**	0.44423	1.8268	0.1402531061668240	10382
**HIVEP3**	0.44423	1.305	0.1402531061668240	10386
**OSBPL3**	0.44423	1.7214	0.1402531061668240	10385
**RHOBTB1**	0.44423	1.7248	0.1402531061668240	10384
**STAM**	0.44423	1.73	0.1402531061668240	10383
**CKAP5**	0.44406	1.4991	0.1406834583313910	10387
**KIAA1586**	0.44398	1.1347	0.14088598600759200	10388
**MCAM**	0.44394	0.7281	0.1409872520121300	10389
**IRX1**	0.44388	0.19377	0.14113915373001900	10390
**CLEC14A**	0.44385	0.93745	0.1412151058099380	10391
**ARL6IP4**	0.55653	1.0331	0.14217723614957300	10395
**BTF3**	0.55653	1.5298	0.14217723614957300	10393
**DEFB136**	0.55653	1.6186	0.14217723614957300	10392
**PTS**	0.55653	1.1138	0.14217723614957300	10394
**ZBED9**	0.55653	1.0027	0.14217723614957300	10396
**FAIM2**	0.44344	1.787	0.14225319940166300	10397

**RNASE10**	0.44341	0.51441	0.14232916347457300	10398
**RAB3IP**	0.44329	0.95657	0.14263302798351800	10399
**GABRR3**	0.44327	1.3707	0.14268367334793000	10400
**SDR42E1**	0.44318	1.2996	0.1429115820184540	10401
**ZNF549**	0.44315	0.72796	0.1429875532242310	10402
**C11orf87**	0.44313	0.66583	0.14303820115318000	10403
**KMT2E**	0.55691	0.81696	0.14313949811192400	10404
**PCTP**	0.44304	1.6294	0.14326612137583400	10405
**MAGEC1**	0.44301	0.21342	0.14334209643658000	10406
**ZNF117**	0.44293	0.13351	0.1435447006446710	10407
**TCEAL4**	0.44287	1.8812	0.14369665766695	10408
**POLR3GL**	0.55718	0.92425	0.14382329105324400	10409
**SAAL1**	0.44282	0.39124	0.14382329105324400	10410
**RASSF8**	0.44278	1.1157	0.14392459942267700	10411
**GOLGA2**	0.44273	1.584	0.14405123696174100	10412
**DUSP3**	0.44269	0.58967	0.14415254865613100	10413
**CCDC175**	0.4426	1.625	0.1443805053796470	10414
**ZNF623**	0.4425	1.3915	0.14463379942764200	10415
**RMI2**	0.55755	0.52908	0.14476044993026200	10416
**KRTAP19-2**	0.44235	0.027562	0.14501375790332500	10418
**OR2T33**	0.44235	0.33966	0.14501375790332500	10417
**GGT7**	0.44234	1.828	0.14503908921210100	10419
**DLK1**	0.55773	1.0706	0.14521641098023300	10423
**MAPK6**	0.55773	1.5295	0.14521641098023300	10421
**NRCAM**	0.55773	1.5544	0.14521641098023300	10420
**OR7A5**	0.55773	1.3588	0.14521641098023300	10422
**FAM136A**	0.44215	0.44418	0.14552040178001500	10424
**ADH1C**	0.44214	2.0416	0.14554573495325800	10425
**SLC35A5**	0.55802	0.1429	0.1459510784392080	10426
**C10orf71**	0.4418	1.7072	0.1464071185280870	10431
**HNF4A**	0.4418	1.0059	0.1464071185280870	10435
**HSPA12B**	0.4418	1.3982	0.1464071185280870	10434

**KIAA1671**	0.4418	2.1617	0.1464071185280870	10427
**LRRC3C**	0.4418	1.806	0.1464071185280870	10430
**NPPB**	0.4418	1.5564	0.1464071185280870	10432
**PKD2**	0.4418	1.8421	0.1464071185280870	10429
**RFX6**	0.4418	1.874	0.1464071185280870	10428
**RSL24D1**	0.4418	1.4251	0.1464071185280870	10433
**FAHD1**	0.55823	0.16794	0.14648312816718700	10436
**BGLAP**	0.55828	0.65791	0.14660981277981400	10437
**ANAPC15**	0.55832	1.0077	0.1467111621638500	10438
**LZTR1**	0.55836	1.0191	0.1468125130547810	10439
**DECR2**	0.55842	1.2388	0.14696454221898500	10440
**SPRR1B**	0.44132	0.17968	0.14762337456080700	10441
**CD302**	0.44127	0.54301	0.1477500804252490	10442
**TAMM41**	0.44123	0.77664	0.14785144682448400	10443
**ARPIN**	0.44119	0.098996	0.1479528147428360	10444
**ARL5B**	0.55885	1.3482	0.14805418418139200	10446
**COL16A1**	0.55885	1.3516	0.14805418418139200	10445
**EXTL3**	0.55885	1.065	0.14805418418139200	10449
**IL5**	0.55885	1.3237	0.14805418418139200	10447
**NFKBIB**	0.55885	0.70044	0.14805418418139200	10452
**NUMB**	0.55885	0.8851	0.14805418418139200	10451
**SPDYE1**	0.55885	1.2763	0.14805418418139200	10448
**TAS2R7**	0.55885	1.0623	0.14805418418139200	10450
**CLCN4**	0.55888	0.32299	0.14813021225859700	10454
**FOXS1**	0.44112	0.84043	0.1481302122585970	10453
**C11orf94**	0.44108	2.0579	0.1482315843601260	10455
**DEPDC7**	0.44097	0.82431	0.14851036549656700	10456
**CHD5**	0.44078	0.21833	0.14899192374849300	10457
**CST4**	0.5593	0.99779	0.14919469544761800	10458
**INO80B**	0.44056	0.66124	0.14954956068653500	10459
**TEC**	0.44053	0.32402	0.1496256056862740	10460
**UGT8**	0.44046	0.87439	0.14980304738450700	10461

**OR8S1**	0.44038	0.54474	0.1500058436723420	10462
**OPN1LW**	0.44032	0.54625	0.15015794493611900	10463
**TYR**	0.55982	0.49999	0.15051286140018100	10464
**MMP1**	0.44013	0.90447	0.15063962187247300	10465
**C6orf163**	0.5599	0.58732	0.15071567931749900	10467
**CCDC28B**	0.5599	1.4306	0.15071567931749900	10466
**PRDM2**	0.44008	1.0122	0.15076638476515500	10468
**SLC22A20**	0.44003	0.88496	0.15089315008035700	10469
**PRKRIP1**	0.56003	0.85321	0.15104527165932200	10470
**CDK17**	0.5601	0.074841	0.1512227512525510	10471
**TTI1**	0.43988	0.96	0.151273460582371	10472
**LINC02210-**	0.43984	1.4994	0.15137488040904600	10473
**PRR30**	0.43983	0.78119	0.151400235608946	10474
**GPBAR1**	0.4398	1.2694	0.15147630179267500	10475
**DZIP1L**	0.43975	1.2168	0.15160308071334300	10476
**TINAGL1**	0.56041	1.2123	0.15200878964379000	10477
**PRR18**	0.56048	1.2445	0.15218629516448600	10478
**RPS16**	0.43934	1.5219	0.15264275996352800	10479
**ABCA9**	0.43921	1.4081	0.15297244875907300	10487
**CCDC71L**	0.43921	1.8538	0.15297244875907300	10483
**PGRMC1**	0.43921	1.5459	0.15297244875907300	10486
**RILPL2**	0.43921	2.0456	0.15297244875907300	10482
**SIGLEC15**	0.43921	2.0806	0.15297244875907300	10481
**ST20**	0.43921	1.7174	0.15297244875907300	10484
**STX11**	0.43921	2.1167	0.15297244875907300	10480
**ZDHHC23**	0.43921	1.5515	0.15297244875907300	10485
**ZNF296**	0.43921	0.31301	0.15297244875907300	10488
**DNM2**	0.56081	0.35866	0.1530231715870610	10489
**OTOL1**	0.56086	0.37987	0.15314998037965500	10490
**C12orf40**	0.43911	1.0916	0.1532260668371220	10491
**NFKBID**	0.56105	0.39171	0.1536318762685820	10492
**AHNAK2**	0.43888	0.90063	0.15380942584641300	10493

**ADAMTS2**	0.56115	1.5393	0.15388552000650600	10494
**CDC42**	0.43885	0.007773[illegible]	0.15388552000650600	10501
**CLCN5**	0.56115	1.4763	0.15388552000650600	10496
**CNEP1R1**	0.56115	0.84703	0.15388552000650600	10500
**NT5DC2**	0.56115	1.2536	0.15388552000650600	10498
**PPP3CA**	0.56115	1.1842	0.15388552000650600	10499
**SPIRE2**	0.56115	1.447	0.15388552000650600	10497
**TMEM125**	0.56115	1.5264	0.15388552000650600	10495
**PEX2**	0.43879	1.8765	0.15403771100013400	10502
**WDR43**	0.43857	0.71646	0.1545957751949050	10503
**DOK2**	0.43847	0.41777	0.1548494566435230	10504
**RAB3A**	0.5616	0.35786	0.1550270395856590	10505
**SH3PXD2B**	0.43837	1.2167	0.1551031480570500	10506
**CLEC4F**	0.4383	2.081	0.15528073798480200	10507
**LTBP3**	0.56173	1.444	0.15535684945259500	10508
**SLC10A1**	0.56177	0.62529	0.15545833280961000	10509
**APEX2**	0.43821	0.51676	0.15550907508840900	10510
**HTR5A**	0.43815	1.0498	0.15566130432760700	10511
**PTF1A**	0.43804	2.0855	0.1559404006372020	10512
**ARF3**	0.56209	0.53116	0.15627025739221800	10514
**HIST1H2AH**	0.43791	1.0946	0.15627025739221800	10513
**PROKR2**	0.43775	0.61252	0.15667625828183700	10515
**BBS5**	0.43767	0.23264	0.15687926840692900	10516
**ITIH6**	0.43766	0.2297	0.15690464512697200	10517
**CNBD1**	0.56237	1.2008	0.15698077589338600	10519
**KIF1A**	0.56237	1.0981	0.15698077589338600	10521
**LPIN2**	0.56237	1.1937	0.15698077589338600	10520
**SLC16A4**	0.56237	1.3564	0.15698077589338600	10518
**WNT5A**	0.56237	0.47397	0.15698077589338600	10522
**COL21A1**	0.43744	0.47798	0.15746295856044900	10523
**GUCD1**	0.43734	2.3252	0.15771675360903900	10524
**ATG3**	0.43722	1.1877	0.1580213210780940	10525

**ADORA3**	0.43715	1.3353	0.15819899220224700	10526
**TXLNA**	0.43711	1.9833	0.158300520800575	10527
**ZFC3H1**	0.43702	1.2608	0.15852896611431400	10528
**KRT4**	0.563	0.97761	0.15857973286262700	10529
**H2AFZ**	0.43696	1.4581	0.1586812675854170	10530
**PABPC1L2B**	0.56308	1.0502	0.15878280394400900	10531
**AMDHD2**	0.4369	0.35719	0.15883357273707300	10532
**APOO**	0.56311	0.99172	0.15885895728710700	10533
**ENGASE**	0.56316	0.82213	0.15898588157298900	10534
**PLSCR2**	0.4368	2.0634	0.15908742284557800	10535
**RFC2**	0.5632	1.2952	0.15908742284557800	10536
**TAF1L**	0.56323	0.55284	0.15916357987634100	10537
**C2orf68**	0.43674	1.8309	0.15923973783017800	10538
**C3AR1**	0.43674	1.5812	0.15923973783017800	10542
**FARSA**	0.43674	1.4142	0.15923973783017800	10543
**FCN3**	0.43674	1.3963	0.15923973783017800	10544
**PARP1**	0.43674	1.8134	0.15923973783017800	10539
**PROX1**	0.43674	1.658	0.15923973783017800	10540
**UHRF1**	0.43674	1.3733	0.15923973783017800	10545
**ZNF214**	0.43674	1.6307	0.15923973783017800	10541
**OR1D5**	0.43668	0.84669	0.15939205650893800	10546
**UBL3**	0.43662	1.1355	0.15954437888557100	10547
**SLC22A14**	0.43654	1.3506	0.1597474811464120	10548
**CLUAP1**	0.43652	0.75489	0.1597982577408720	10549
**RRAGB**	0.56356	0.55417	0.16000136823984100	10550
**RFLNA**	0.56357	1.7066	0.1600267575161230	10551
**HEY2**	0.43639	0.97488	0.16012831565290800	10552
**MED13**	0.43631	0.81756	0.16033143688201500	10554
**OTP**	0.43631	1.0172	0.16033143688201500	10553
**OSM**	0.4363	0.61494	0.1603568275005710	10555
**ADRA2C**	0.56374	1.3519	0.16045839100868800	10556
**AJUBA**	0.56374	0.40241	0.16045839100868800	10559

**DNAH9**	0.56374	1.2036	0.16045839100868800	10557
**NPTX2**	0.56374	0.90568	0.16045839100868800	10558
**ATP6V1E2**	0.4362	1.1693	0.16061073937444100	10560
**SDHAF4**	0.4362	0.25742	0.16061073937444100	10561
**ZC3H13**	0.4361	1.1083	0.16086466160274900	10562
**AK5**	0.56424	0.69173	0.16172807486203800	10563
**CYP2F1**	0.43572	1.1435	0.16182966080641800	10564
**TAPBP**	0.43568	0.76649	0.16193124842073700	10565
**COPS6**	0.43561	0.037826	0.16210903076740600	10566
**KLC2**	0.43551	0.90213	0.162363014437497	10567
**METTL14**	0.43536	1.0217	0.16274400958551300	10568
**FMNL2**	0.4353	0.56059	0.162896414255957	10569
**PRR29**	0.43525	0.74074	0.16302342103796300	10570
**GBGT1**	0.56488	1.343	0.16335365098284400	10572
**GPR45**	0.56488	1.1797	0.16335365098284400	10573
**GPR65**	0.56488	0.96713	0.16335365098284400	10575
**KHDRBS1**	0.56488	1.1591	0.16335365098284400	10574
**ZNF100**	0.56488	1.6397	0.16335365098284400	10571
**RHOG**	0.435	0.90881	0.16365849443435100	10576
**AURKA**	0.43491	0.2432	0.1638871370018260	10577
**PTAFR**	0.43485	1.5097	0.1640395701389680	10578
**GIMD1**	0.43475	1.7404	0.16429363383919400	10579
**SDE2**	0.43458	1.4781	0.16472556647626600	10580
**BCAS2**	0.43452	1.6328	0.16487802062239100	10581
**ZNF721**	0.43448	0.02234	0.16497965884844000	10582
**HRCT1**	0.43436	0.0057603	0.16528458375619500	10584
**WDR13**	0.43436	2.452	0.16528458375619500	10583
**RPGR**	0.56566	0.034295	0.16533540606742600	10585
**ARHGDIA**	0.56567	0.35283	0.16536081738316400	10586
**NECAP1**	0.43431	1.4167	0.16541164033497400	10587
**MYL4**	0.43429	1.6767	0.16546246371400900	10588
**OR13C5**	0.43429	1.401	0.16546246371400900	10589

**RGS4**	0.43429	1.3873	0.16546246371400900	10590
**PRKD2**	0.43427	0.53878	0.16551328752040900	10591
**ARL 9.00**	0.43422	0.90251	0.16564034890703400	10592
**BCAR1**	0.43409	0.25297	0.1659707210322410	10593
**GPR153**	0.43403	1.7969	0.16612320658201500	10594
**MAGI2**	0.43398	0.5305	0.16625028082384300	10595
**PRSS36**	0.43386	0.56463	0.1665552699608890	10596
**RTP2**	0.43379	0.28466	0.1667331874433610	10597
**MMRN2**	0.56622	1.1383	0.16675860465726100	10599
**TCF4**	0.56622	1.0452	0.16675860465726100	10600
**ZFYVE26**	0.56622	1.2327	0.16675860465726100	10598
**OSBPL9**	0.4337	1.3585	0.1669619462479910	10601
**LINC02312**	0.43354	0.85415	0.1673686501480270	10603
**PPP1R18**	0.43354	0.97453	0.1673686501480270	10602
**MYH4**	0.43347	0.80512	0.16754659180541100	10604
**TWF2**	0.43347	0.19859	0.16754659180541100	10605
**PLA2G2D**	0.56662	0.10252	0.16777538173202300	10606
**CYP4F3**	0.43337	0.6832	0.16780080337683900	10607
**ATP13A2**	0.43322	0.17898	0.16818214107103900	10608
**TSPEAR**	0.56685	0.10326	0.16836010702767300	10609
**ZNF160**	0.56688	1.1991	0.16843637978385700	10610
**GCNT1**	0.56689	0.85137	0.168461804253634	10611
**CHMP4B**	0.43306	1.2754	0.16858892823616600	10612
**FAM98A**	0.56695	0.049169	0.168614353359524	10613
**LRP11**	0.56701	1.3729	0.16876690638908800	10614
**LOC1001289**	0.56705	0.50086	0.16886861059046900	10615
**OR2V2**	0.43293	0.56334	0.16891946334607800	10616
**KCNA4**	0.43288	0.12061	0.1690465971463930	10617
**SLC27A6**	0.43276	0.80492	0.16935172941848900	10618
**ARMC6**	0.56756	1.0177	0.17016549264339600	10622
**HRH3**	0.56756	0.89732	0.17016549264339600	10623
**NOTCH1**	0.56756	1.1697	0.17016549264339600	10620

**SNX32**	0.56756	1.2238	0.17016549264339600	10619
**TNFSF10**	0.56756	0.79047	0.17016549264339600	10624
**TPCN2**	0.56756	1.1505	0.17016549264339600	10621
**RECK**	0.56765	0.75984	0.17039438383027800	10625
**CTAGE6**	0.43218	0.42382	0.17082675821617600	10626
**C9orf47**	0.56791	0.28242	0.1710556752263600	10627
**FAM102B**	0.43202	1.4964	0.1712337279861870	10628
**CNPPD1**	0.432	0.46687	0.17128460120006200	10629
**ADAT1**	0.43176	2.2579	0.17189511438150400	10630
**EPHB4**	0.43176	0.42866	0.17189511438150400	10635
**GCLC**	0.43176	1.8719	0.17189511438150400	10631
**PEX14**	0.43176	1.5741	0.17189511438150400	10634
**SLC1A5**	0.43176	1.8617	0.17189511438150400	10632
**UTP18**	0.43176	1.7633	0.17189511438150400	10633
**OR6C4**	0.56826	1.1318	0.1719459933687650	10636
**THOC7**	0.56833	0.68376	0.17212407333011800	10637
**SOX30**	0.43161	0.17433	0.17227671764108200	10638
**TMEM269**	0.43158	0.74123	0.17235304130154400	10639
**MBD3**	0.43157	2.0417	0.17237848274477300	10640
**MT1B**	0.43157	2.0046	0.17237848274477300	10641
**MTM1**	0.43157	1.257	0.17237848274477300	10642
**AES**	0.43154	1.9439	0.17245480774393600	10643
**FLI1**	0.43143	1.9446	0.1727346746724820	10644
**B3GLCT**	0.43142	0.67405	0.17276011779105100	10645
**ATG14**	0.43129	1.1068	0.17309088851523400	10646
**ASB17**	0.56878	0.76496	0.17326900366971900	10651
**CALD1**	0.56878	1.587	0.17326900366971900	10647
**COL23A1**	0.56878	1.3819	0.17326900366971900	10649
**ENTPD2**	0.56878	0.67588	0.17326900366971900	10652
**HIST1H4L**	0.56878	0.81377	0.17326900366971900	10650
**KRT76**	0.56878	0.67427	0.17326900366971900	10653
**RSL1D1**	0.56878	1.4219	0.17326900366971900	10648

**HEBP2**	0.43117	0.043353	0.17339623214505800	10654
**CYP19A1**	0.56903	0.76399	0.17390517413459500	10655
**CYSLTR1**	0.43097	0.45365	0.17390517413459500	10656
**CNRIP1**	0.43089	1.3228	0.1741087635354400	10657
**RPL19**	0.56911	0.29779	0.1741087635354400	10658
**CCDC116**	0.43081	1.1162	0.17431236015258100	10660
**CD46**	0.43081	1.7808	0.17431236015258100	10659
**HRH2**	0.43075	0.29557	0.17446506235651600	10661
**ZNF385A**	0.5693	0.58934	0.1745923173004510	10662
**SPAG11A**	0.56933	0.75609	0.17466867162371500	10663
**EPO**	0.56936	1.2585	0.1747450269652330	10664
**TGM2**	0.56941	0.6767	0.17487228813183200	10665
**ATP2B3**	0.43053	1.4623	0.1750250052701600	10667
**CERS1**	0.43053	2.1723	0.1750250052701600	10666
**TMEM117**	0.43038	2.1032	0.17540681598227300	10668
**ATP5J**	0.43036	1.1146	0.175457726007928	10669
**COPS2**	0.4303	1.3014	0.175610458813817	10670
**TAS1R3**	0.43026	0.99901	0.17571228295991500	10671
**COPS8**	0.43012	0.38628	0.17606868182458900	10672
**CALCRL**	0.4301	0.83704	0.1761195977726280	10673
**ZNF845**	0.43001	2.1836	0.17634872519062100	10674
**ANKDD1A**	0.57006	0.85353	0.17652694180447900	10678
**CENPU**	0.57006	1.0381	0.17652694180447900	10677
**GIMAP2**	0.57006	1.5546	0.17652694180447900	10675
**SLC39A14**	0.57006	1.1437	0.17652694180447900	10676
**KRT32**	0.57012	0.075048	0.17667970336413500	10679
**GUCA2B**	0.42983	1.9586	0.17680700781306500	10680
**MYO9B**	0.57026	0.2082	0.17703616304307800	10681
**PRR12**	0.42973	0.91502	0.17706162530890900	10682
**INTS11**	0.42971	1.0381	0.1771125501849490	10683
**SUB1**	0.4296	1.2089	0.17739264521663000	10684
**SUPT7L**	0.57047	0.15617	0.17757089475392900	10685

**AGO4**	0.42932	0.90091	0.17810567724016100	10686
**MARVELD3**	0.57069	1.1422	0.17813114434211300	10687
**OXGR1**	0.42928	0.73096	0.178207546341178	10688
**SLC6A4**	0.42926	1.1643	0.17825848158507100	10689
**AADACL4**	0.42925	1.6378	0.17828394938042600	10696
**ARID3B**	0.42925	1.0661	0.17828394938042600	10701
**CLEC12B**	0.42925	1.688	0.17828394938042600	10695
**CRTC3**	0.42925	1.2415	0.17828394938042600	10700
**EDARADD**	0.42925	1.2442	0.17828394938042600	10699
**KRTAP5-10**	0.42925	1.2893	0.17828394938042600	10697
**MAP4K5**	0.42925	2.2617	0.17828394938042600	10692
**MDH1**	0.42925	1.7184	0.17828394938042600	10694
**NT5C3B**	0.42925	1.258	0.17828394938042600	10698
**PIGK**	0.42925	2.3613	0.17828394938042600	10691
**RASD1**	0.42925	2.499	0.17828394938042600	10690
**UCP3**	0.42925	0.5941	0.17828394938042600	10702
**ZNF768**	0.42925	1.8931	0.17828394938042600	10693
**PYURF**	0.42897	1.686	0.17899709465976800	10703
**CRYBG1**	0.42867	2.2527	0.17976127992288700	10704
**FGB**	0.42867	1.4754	0.17976127992288700	10707
**HLTF**	0.42867	1.6767	0.17976127992288700	10706
**VANGL2**	0.42867	2.1284	0.17976127992288700	10705
**ADH5**	0.42857	0.9254	0.1800160316501580	10708
**CXCR2**	0.42855	0.72591	0.18006698339694200	10709
**ZP3**	0.42854	0.3227	0.18009245944561500	10710
**AGRN**	0.57149	0.32092	0.180168888292969	10711
**TCHHL1**	0.42848	0.27447	0.18024531819269300	10712
**DBH**	0.57159	1.3418	0.1804236587206090	10714
**PLTP**	0.57159	0.88088	0.1804236587206090	10715
**TESC**	0.57159	1.391	0.1804236587206090	10713
**TMEM132A**	0.57159	0.80344	0.1804236587206090	10716
**ADSSL1**	0.57189	0.32665	0.18118804033774400	10717

**FMO5**	0.4281	0.90821	0.18121352154568200	10718
**RBM24**	0.57192	0.71685	0.1812644843145320	10719
**LRMP**	0.42806	0.48957	0.18131544755413400	10720
**RPS11**	0.57196	0.37291	0.18136641126462900	10721
**TMEM56**	0.42802	0.23486	0.1814173754461550	10722
**SEC16A**	0.42793	0.38063	0.18164672009584900	10723
**REEP1**	0.42776	1.3226	0.18207995273424300	10724
**KCNJ4**	0.42775	0.76982	0.18210543807051300	10725
**LOC100144!**	0.42771	2.1295	0.18220738059847000	10726
**DNAJA4**	0.42753	1.2904	0.18266614542202100	10727
**KIAA1024**	0.42744	2.0157	0.1828955422443530	10728
**OR10Q1**	0.42742	0.39605	0.18294652062252100	10729
**NUTF2**	0.42739	2.2915	0.18302298908121100	10730
**MBD4**	0.42738	1.3387	0.18304847880522900	10731
**RNF151**	0.42731	0.8461	0.18322691020419800	10732
**DCDC2**	0.5728	1.011	0.1835073141905760	10733
**ALG1L2**	0.42696	0.97541	0.18411915481994700	10737
**KBTBD6**	0.42696	1.7457	0.18411915481994700	10735
**LINC01835**	0.42696	0.17187	0.18411915481994700	10738
**RDH12**	0.42696	2.0586	0.18411915481994700	10734
**SGSH**	0.42696	1.6547	0.18411915481994700	10736
**ZNF224**	0.57324	1.1836	0.18462907465378500	10739
**PTCHD1**	0.57331	0.65577	0.18480755793027300	10740
**GATM**	0.42658	1.4374	0.18508804354706500	10741
**BCL2L2-PAI**	0.42642	1.7293	0.1854960486256050	10742
**ARF4**	0.57361	0.082144	0.18557255301338000	10743
**MIER2**	0.4263	0.3444	0.1858020726951000	10744
**MEI1**	0.42621	1.6749	0.18603160216446400	10745
**NAIF1**	0.57395	0.61482	0.1864396787616660	10749
**PCSK2**	0.57395	1.4765	0.1864396787616660	10746
**PFN4**	0.57395	1.1881	0.1864396787616660	10748
**SNX15**	0.57395	1.2823	0.1864396787616660	10747

**ZBTB39**	0.42593	0.97042	0.18674575658039600	10750
**CEACAM4**	0.42583	1.2543	0.18700083479210800	10751
**KRT18**	0.42582	1.8189	0.18702634328216600	10752
**STK39**	0.42582	1.6807	0.18702634328216600	10753
**MEGF9**	0.42581	1.492	0.18705185189391200	10754
**PER2**	0.42577	1.0785	0.18715388755811700	10755
**AMOTL1**	0.42571	0.89837	0.18730694470805100	10756
**PER3**	0.42571	0.44115	0.18730694470805100	10757
**MOB3B**	0.57444	1.2161	0.18768960678773700	10758
**KIAA0586**	0.57451	0.45341	0.18786819182673400	10759
**WBP2NL**	0.42537	0.88053	0.18817435155039800	10760
**DPYSL2**	0.57468	0.67964	0.18830192330584300	10761
**CPA3**	0.5748	0.6616	0.18860810802585000	10762
**CALML6**	0.42514	0.38016	0.18876120701454100	10763
**GGCX**	0.42511	0.35962	0.1888377581677390	10764
**NAGK**	0.42509	0.29315	0.1888887928845880	10765
**SYNRG**	0.425	1.1329	0.1891184552002120	10766
**MDM2**	0.57501	1.1209	0.18914397385076900	10767
**NACA**	0.42493	0.58286	0.18929708834115600	10768
**CYP1A1**	0.42478	1.408	0.1896798939905180	10769
**IL17F**	0.42475	2.0739	0.1897564584539630	10770
**OR2L13**	0.4247	1.5068	0.18988406836508300	10771
**ELMOD3**	0.42469	0.40558	0.18990959071827500	10772
**TMEM202**	0.42452	1.0945	0.19034348966276400	10773
**CDC42BPG**	0.5755	0.80283	0.1903945389517240	10778
**LIN9**	0.5755	0.98997	0.1903945389517240	10776
**LRFN3**	0.5755	1.0477	0.1903945389517240	10775
**PARP4**	0.5755	0.86024	0.1903945389517240	10777
**VMAC**	0.5755	1.0708	0.1903945389517240	10774
**C1orf53**	0.42444	1.9563	0.19054768979604000	10781
**CCNB1**	0.42444	1.5451	0.19054768979604000	10783
**CHTOP**	0.42444	2.4313	0.19054768979604000	10779

**EBF1**	0.42444	1.5895	0.19054768979604000	10782
**HSBP1L1**	0.42444	1.1819	0.19054768979604000	10784
**NDUFS3**	0.42444	2.0779	0.19054768979604000	10780
**RHO**	0.5756	0.59227	0.19064979284154200	10785
**OXCT1**	0.42431	1.3473	0.19087953196220700	10786
**DUOXA1**	0.57572	1.0564	0.19095611390720400	10787
**PRIMA1**	0.42426	1.5507	0.1910071691593160	10788
**ZNF14**	0.5758	0.64763	0.19116033790336500	10789
**ZC4H2**	0.42418	2.4902	0.19121139514776100	10790
**CLLU1**	0.42412	0.39694	0.19136456987209500	10791
**FSCN1**	0.42406	1.781	0.19151774908614200	10792
**LGSN**	0.57595	0.99874	0.1915432793919200	10793
**CTSO**	0.42393	0.89036	0.191849652803834	10794
**PMAIP1**	0.42385	1.6107	0.19205391174749700	10796
**RNF125**	0.42385	2.0099	0.19205391174749700	10795
**GGA3**	0.57628	0.77827	0.19238584962435400	10797
**PIBF1**	0.42357	0.3965	0.19276888120948400	10798
**INHBE**	0.42335	0.47157	0.1933307120434750	10799
**ANKRD34C**	0.57695	1.2887	0.19409694338719400	10802
**C20orf202**	0.57695	1.5115	0.19409694338719400	10800
**ZBED4**	0.57695	1.3254	0.19409694338719400	10801
**GAGE10**	0.57714	0.069044	0.1945822821531640	10804
**SLC4A7**	0.42286	0.84629	0.1945822821531640	10803
**RIMBP2**	0.42268	0.68919	0.19504211905225100	10805
**ADIRF**	0.57734	0.35064	0.19509321458496300	10806
**FXYD2**	0.57745	0.7889	0.19537424912266200	10807
**TSPAN5**	0.42251	0.81573	0.19547644732450600	10808
**IRF7**	0.57759	0.64553	0.19573195176342300	10810
**UBA52**	0.57759	0.77374	0.19573195176342300	10809
**KCNH4**	0.57761	0.018526	0.1957830541839500	10811
**AMHR2**	0.57768	0.32018	0.19596191668283300	10813
**SERGEF**	0.42232	0.6835	0.19596191668283300	10812

**COX19**	0.57772	0.76268	0.19606412663929800	10814
**TRIO**	0.42227	1.6306	0.19608967944839400	10815
**FAM163B**	0.5778	1.0824	0.19626855269786200	10816
**OR2T27**	0.42218	1.3611	0.1963196604936820	10817
**AMOTL2**	0.42217	0.077466	0.19634521458386800	10826
**ANKRD44**	0.42217	2.47	0.19634521458386800	10818
**C4orf50**	0.42217	1.7227	0.19634521458386800	10822
**FOXO3**	0.42217	1.7269	0.19634521458386800	10821
**HCFC1**	0.42217	1.9669	0.19634521458386800	10820
**P2RX4**	0.42217	1.7083	0.19634521458386800	10823
**PLAT**	0.42217	2.1693	0.19634521458386800	10819
**RABL2A**	0.42217	1.702	0.19634521458386800	10824
**STMN4**	0.42217	0.74452	0.19634521458386800	10825
**PDE1A**	0.57792	0.61521	0.19657520716704800	10827
**FKTN**	0.42207	2.0077	0.19660076254012000	10828
**MRPL15**	0.57801	0.0031465	0.19680521014816300	10829
**LIG1**	0.57802	0.5845	0.19683076667737100	10830
**PRCC**	0.42196	0.61573	0.19688188012145200	10831
**IKBKB**	0.42192	0.5386	0.19698410855278400	10832
**OR6A2**	0.42185	1.2133	0.19716301326161500	10833
**PDE11A**	0.57815	0.071382	0.19716301326161500	10834
**CDCA5**	0.42181	0.48057	0.19726524735663900	10835
**MANSC1**	0.57848	1.1472	0.19800650627457600	10836
**PPP1CA**	0.57848	1.0716	0.19800650627457600	10837
**LINGO1**	0.5789	0.044125	0.19908024669112900	10838
**CYP27B1**	0.579	0.94022	0.19933593295621800	10839
**TMEM59L**	0.42095	0.33574	0.19946378097444400	10840
**UBE2E3**	0.5791	0.89234	0.19959163225280000	10841
**FABP5**	0.57927	0.20356	0.2000263510153540	10842
**NLE1**	0.57929	0.23143	0.20007749688271300	10844
**SLC35A2**	0.57929	0.51383	0.20007749688271300	10843
**ERICH5**	0.57931	0.90242	0.20012864327342800	10845

**AKIRIN2**	0.57935	0.013759	0.2002309376254940	10847
**JAK2**	0.42065	1.8954	0.20023093762549400	10846
**NTHL1**	0.4205	1.2649	0.20061456011511900	10848
**AQP4**	0.57953	0.21728	0.2006912881539780	10849
**HIST1H1B**	0.57961	0.27933	0.20089590203490300	10850
**CAVIN3**	0.42032	0.97223	0.20107494607978100	10851
**PRDM14**	0.42019	0.71585	0.2014074735541940	10852
**RBAK-RBAI**	0.42017	1.126	0.2014586336028650	10853
**ANKRD42**	0.42007	1.1325	0.20171444175800800	10859
**CYBB**	0.42007	1.9224	0.20171444175800800	10855
**EIF3E**	0.42007	2.0067	0.20171444175800800	10854
**NAT14**	0.42007	1.6103	0.20171444175800800	10857
**TRIML1**	0.42007	1.8118	0.20171444175800800	10856
**ZNF524**	0.42007	1.4669	0.20171444175800800	10858
**FRMPD2**	0.58003	1.0783	0.20197026311273700	10861
**KCTD17**	0.58003	1.3207	0.20197026311273700	10860
**CARMIL2**	0.41994	0.81694	0.20204701209578100	10862
**RICTOR**	0.58014	0.10721	0.2022516818700690	10863
**GNAZ**	0.41983	0.01249	0.2023284352191360	10867
**MACROD2**	0.41983	1.1495	0.2023284352191360	10864
**NOVA1**	0.41983	0.18877	0.2023284352191360	10866
**SHANK2**	0.41983	0.44545	0.2023284352191360	10865
**ARHGAP8**	0.41982	0.96691	0.2023540199336620	10868
**PMM1**	0.41965	1.7047	0.20278898036002800	10869
**NPC1L1**	0.58042	0.94337	0.20296809285855300	10870
**RPS7**	0.41951	1.5913	0.20314721186837800	10871
**GALNT2**	0.41947	1.1059	0.2032495685141140	10872
**LPAR3**	0.58062	0.40594	0.20347987875415300	10873
**IQCB1**	0.58065	0.87793	0.20355665123195000	10874
**STK24**	0.41932	0.45137	0.20363342490945500	10875
**MYO1F**	0.41926	1.5432	0.203786975865551	10876
**MTFP1**	0.58081	0.6518	0.20396612472056400	10877

**FAM120B**	0.58085	0.20886	0.20406849843346900	10878
**KIF2A**	0.4191	0.38769	0.20419646858217700	10879
**MSRB3**	0.5809	0.0047211	0.2041964685821770	10880
**SRC**	0.41907	1.4924	0.20427325227631400	10881
**AFF1**	0.419	0.60047	0.20445241891347700	10882
**RC3H2**	0.41898	1.3554	0.2045036105863940	10883
**TMEM251**	0.58105	0.53446	0.20458039910061200	10884
**YPEL4**	0.41895	0.31735	0.20458039910061200	10885
**AMDHD1**	0.4189	0.81148	0.20470838263843200	10886
**ACO1**	0.41873	0.41892	0.20514355176073600	10891
**CHUK**	0.58127	1.2417	0.205143551760736	10888
**COLGALT1**	0.58127	0.79721	0.205143551760736	10890
**HAUS7**	0.58127	1.1481	0.205143551760736	10889
**PLAU**	0.58127	1.2878	0.205143551760736	10887
**SAMD9**	0.5814	0.19222	0.20547635435699500	10892
**M1AP**	0.41831	0.033442	0.20621884221808300	10893
**ZMIZ2**	0.58179	0.30013	0.20647489885493500	10894
**PPP1R2**	0.41814	1.0021	0.20665414655440300	10895
**PADI2**	0.41803	1.5311	0.20693583492777700	10896
**CASS4**	0.58213	0.64899	0.20734559278909700	10897
**PIMREG**	0.58227	0.76672	0.20770415947393400	10898
**NIPSNAP1**	0.41763	2.2038	0.2079602948815450	10899
**HSD17B13**	0.4176	0.79481	0.20803713816342200	10900
**FCHO2**	0.41755	1.7255	0.20816521302986200	10901
**CPLX3**	0.4175	1.6041	0.20829329131075500	10906
**FAM46B**	0.4175	2.2472	0.20829329131075500	10902
**GOLGA4**	0.4175	0.15221	0.20829329131075500	10908
**PDIA4**	0.4175	1.5773	0.20829329131075500	10907
**PXMP4**	0.4175	2.0483	0.20829329131075500	10905
**TEX261**	0.4175	2.1525	0.20829329131075500	10903
**ZNF2**	0.4175	2.1297	0.20829329131075500	10904
**MEI4**	0.41746	1.3319	0.2083957563954090	10909

**SMURF2**	0.41737	0.37933	0.20862631083710000	10910
**HES2**	0.58265	0.25863	0.20867754666298300	10911
**GOLT1B**	0.58268	0.2467	0.2087544014289300	10912
**WDR61**	0.41729	1.341	0.2088312574278630	10913
**TBC1D15**	0.41728	0.40688	0.2088568763682560	10914
**KISS1**	0.41704	0.30516	0.20947177210118500	10915
**RGN**	0.58303	1.3069	0.20965113159859100	10916
**ZNHIT6**	0.58303	0.97504	0.20965113159859100	10917
**NEK11**	0.41695	1.9673	0.20970237840775100	10918
**PRKACA**	0.41695	0.90843	0.20970237840775100	10919
**ANAPC16**	0.41684	1.4024	0.20998424570583100	10920
**ZNF442**	0.41681	1.8812	0.21006112149968900	10921
**STARD4**	0.58324	1.1116	0.210189250581659	10922
**WWC3**	0.58327	0.82154	0.21026612968697100	10923
**ZNF836**	0.58334	0.82966	0.2104455190996180	10924
**POU2F3**	0.58335	0.19558	0.21047114671124600	10925
**OR51L1**	0.41656	1.2539	0.21070180143820100	10926
**TYMSOS**	0.58348	0.15477	0.21080431824751400	10927
**GSS**	0.41648	0.30758	0.21090683727223100	10928
**SYN3**	0.41626	1.0197	0.21147073156099100	10929
**DDX60L**	0.41613	0.93916	0.2118039734187900	10930
**SPP2**	0.584	0.57505	0.2121372387977210	10931
**SZRD1**	0.41592	1.0769	0.21234233689321200	10932
**MAD2L1**	0.41585	2.17	0.212521805053552	10933
**SLC25A36**	0.41585	1.6395	0.212521805053552	10934
**OPN1MW2**	0.58422	0.24197	0.21270128005882900	10935
**GNLY**	0.41577	2.0671	0.21272691990443500	10936
**LSM 2.00**	0.41571	0.44951	0.21288076191534600	10937
**AP1G2**	0.41567	1.3728	0.21298332605470600	10938
**TEX26**	0.41562	1.9819	0.2131115343795730	10939
**DGKG**	0.58447	1.5449	0.2133423181931480	10941
**FAM117A**	0.58447	0.75634	0.2133423181931480	10942

**GPR137C**	0.58447	1.6139	0.2133423181931480	10940
**PRRX2**	0.41551	0.034129	0.21339360502767200	10943
**ADCK1**	0.41527	1.873	0.21400909086321300	10944
**ATP11A**	0.41526	0.58447	0.214034737864091	10945
**CENPK**	0.41519	2.0192	0.21421427081304800	10946
**POMGNT1**	0.41519	1.5027	0.21421427081304800	10948
**PTCD3**	0.41519	1.9859	0.21421427081304800	10947
**ITGA2**	0.41518	0.87623	0.21423991894066500	10949
**CEPT1**	0.41515	0.70032	0.2143168641691410	10950
**PLCZ1**	0.58489	0.0069495	0.2144194597809240	10951
**ABCC5**	0.41495	0.50619	0.21482986480853500	10952
**FAM161A**	0.4149	1.8117	0.21495812379694800	10954
**KBTBD12**	0.4149	1.9212	0.21495812379694800	10953
**SYBU**	0.41477	2.2236	0.21529161372121900	10955
**CREB5**	0.41476	1.1872	0.21531726778356800	10956
**RBCK1**	0.41458	0.66488	0.21577906515437700	10957
**CHRNB4**	0.41444	0.005498	0.21613827269685200	10958
**MAFG**	0.58561	0.80087	0.21626656786103900	10959
**TSKU**	0.41435	1.5793	0.21636920655516800	10960
**WRN**	0.41427	1.3471	0.2165744907825280	10961
**PRTN3**	0.41415	1.6724	0.2168824342390680	10962
**ARL 16.00**	0.58591	0.49688	0.21703641367796500	10963
**ATP6V1D**	0.414	0.14131	0.21726739248582800	10964
**BRICD5**	0.41398	0.29628	0.217318722683465	10965
**GPS1**	0.41397	1.59	0.21734438799698500	10966
**ZFP82**	0.41383	0.87077	0.21770371742641900	10967
**SRSF10**	0.41379	1.218	0.21780638813852900	10968
**ANKRD36**	0.58625	0.52783	0.21790906114651900	10969
**CFAP20**	0.58628	1.1628	0.21798606740989600	10971
**FAM81B**	0.58628	0.80256	0.21798606740989600	10973
**IGFBP6**	0.58628	1.1806	0.21798606740989600	10970
**PARS2**	0.58628	1.0359	0.21798606740989600	10972

**TUBB6**	0.58628	0.67737	0.21798606740989600	10974
**RING1**	0.58633	0.35897	0.21811441405488000	10975
**TFAP2B**	0.41357	0.45169	0.2183711181257740	10976
**ERICH6**	0.58649	0.087496	0.21852514747432500	10977
**THNSL2**	0.58656	0.0552	0.2187048549337610	10978
**GSG2**	0.58658	0.11279	0.21875620121916300	10979
**FKBP11**	0.58672	0.10447	0.21911564137322900	10980
**HELZ2**	0.41327	1.1941	0.21914131675262900	10983
**LRRC10**	0.41327	1.5604	0.21914131675262900	10982
**SUN3**	0.41327	2.0182	0.21914131675262900	10981
**RAD23B**	0.41318	0.99012	0.2193724016699830	10984
**AEBP1**	0.41316	1.4948	0.21942375546422000	10992
**C21orf59**	0.41316	1.3879	0.21942375546422000	10994
**COPS3**	0.41316	1.3934	0.21942375546422000	10993
**FAM213A**	0.41316	1.7718	0.21942375546422000	10990
**GANAB**	0.41316	2.2259	0.21942375546422000	10986
**KCNMB3**	0.41316	1.8448	0.21942375546422000	10988
**LMAN2**	0.41316	1.8351	0.21942375546422000	10989
**METTL16**	0.41316	0.90283	0.21942375546422000	10997
**OST4**	0.41316	1.2404	0.21942375546422000	10995
**RMDN1**	0.41316	2.2197	0.21942375546422000	10987
**SLAMF9**	0.41316	1.1812	0.21942375546422000	10996
**TRAPPC2**	0.41316	2.3809	0.21942375546422000	10985
**ZNF749**	0.41316	1.6185	0.21942375546422000	10991
**EXO5**	0.58697	0.60764	0.21975756923778700	10998
**EPG5**	0.58698	1.04	0.21978324823416100	10999
**TRAF3IP2**	0.41302	0.58901	0.21978324823416100	11000
**PXT1**	0.41292	2.2072	0.22004004617159000	11001
**DENND1B**	0.41288	0.98813	0.2201427694084920	11002
**CASTOR2**	0.58714	0.63881	0.22019413189795100	11003
**PNO1**	0.58716	0.71875	0.22024549496827900	11004
**PDE7A**	0.58717	1.0195	0.22027117672131700	11005

**DNAAF1**	0.58719	1.1919	0.22032254066323000	11006
**DHDDS**	0.41276	1.1477	0.2204509530612710	11007
**CHPF**	0.41267	1.3354	0.22068210453972100	11008
**RAD17**	0.4126	1.6386	0.22086189717422600	11009
**CCL15**	0.58748	0.73279	0.22106738321322900	11011
**HSF2**	0.58748	0.85046	0.22106738321322900	11010
**TKTL1**	0.5875	1.1106	0.22111875618110500	11012
**AAR2**	0.58754	0.85017	0.22122150386764900	11013
**POTEB2**	0.58755	0.11749	0.2212471911541420	11015
**SLCO5A1**	0.41245	0.58785	0.2212471911541420	11014
**PFKFB3**	0.58761	0.20427	0.22140131793932900	11016
**HSP90AA1**	0.41238	1.3206	0.2214270062480720	11017
**KAZN**	0.58762	1.0268	0.22142700624807300	11018
**MYO1C**	0.58763	0.39309	0.2214526947029260	11019
**CCDC83**	0.58768	1.0732	0.22158113916950000	11020
**FBXL12**	0.41213	0.013625	0.22206926150090100	11021
**VSIG4**	0.41211	1.6834	0.22212064587481900	11022
**CDKN2B**	0.41203	1.0139	0.22232618923650700	11023
**TGM4**	0.58803	0.084805	0.22248035292126300	11024
**FAM81A**	0.58813	0.94528	0.22273730414777100	11026
**KCNA7**	0.58813	1.4153	0.22273730414777100	11025
**PLA2G16**	0.58813	0.90508	0.22273730414777100	11027
**JADE2**	0.41181	2.6286	0.22289148194138700	11028
**RALY**	0.41181	1.7794	0.22289148194138700	11029
**PNLIP**	0.41176	0.61332	0.22301996748313900	11030
**ZNF480**	0.41152	1.0377	0.22363674937315600	11031
**KCNJ13**	0.41149	0.082055	0.22371385308652100	11032
**KNG1**	0.41146	0.63845	0.2237909581298140	11033
**PRMT2**	0.58856	0.70566	0.22384236223110700	11034
**LRG1**	0.41142	0.34264	0.2238937669238540	11035
**COX15**	0.41139	0.83663	0.22397087507227400	11036
**DIO2**	0.58862	0.39927	0.22399657808430200	11037

**SV2C**	0.41124	0.9744	0.2243564357986040	11038
**CNKSR2**	0.41118	2.3308	0.22451066942453100	11040
**FAP**	0.41118	1.4084	0.22451066942453100	11045
**FMO2**	0.41118	1.5589	0.22451066942453100	11044
**HLA-DOA**	0.41118	1.5938	0.22451066942453100	11043
**IMPA2**	0.41118	1.1416	0.22451066942453100	11049
**NUDT19**	0.41118	1.3785	0.22451066942453100	11046
**PDGFD**	0.41118	0.23547	0.22451066942453100	11050
**SATB1**	0.41118	2.357	0.22451066942453100	11039
**TRIM11**	0.41118	1.3108	0.22451066942453100	11047
**UQCRC1**	0.41118	2.0443	0.22451066942453100	11042
**ZNF235**	0.41118	2.0686	0.22451066942453100	11041
**ZNF764**	0.41118	1.2825	0.22451066942453100	11048
**ITPR2**	0.58885	0.60366	0.22458778823995500	11051
**OCM2**	0.58891	0.6612	0.2247420298782500	11052
**HUS1B**	0.58893	0.17438	0.22479344494562100	11053
**DNAH14**	0.41103	0.61588	0.22489627686317400	11054
**ZNF580**	0.41098	1.0231	0.22502482010442200	11055
**DMRT2**	0.41089	1.1385	0.225256207309808	11056
**LIME1**	0.41085	0.31358	0.22535904993813100	11057
**FLRT2**	0.58916	1.3139	0.2253847609675820	11058
**CYP2U1**	0.41083	1.7175	0.2254104721460180	11059
**COCH**	0.58944	0.76433	0.22610473034868600	11060
**CRNN**	0.41051	1.7401	0.2262333086323130	11061
**GRPR**	0.41044	1.8942	0.22641332451326900	11062
**MAEA**	0.41042	1.7928	0.22646475896944700	11063
**STX17**	0.41039	0.743	0.22654191177705300	11064
**AZI2**	0.58963	0.41424	0.22659334773123600	11065
**DEFB105B**	0.41032	0.19141	0.22672194023967900	11066
**TNRC18**	0.4103	1.6766	0.22677337829264600	11067
**BCL2L11**	0.41028	1.5047	0.22682481694560200	11068
**BAZ2A**	0.41022	1.0531	0.22697913650591200	11069

**DCUN1D3**	0.41015	1.8784	0.22715918282494400	11070
**SLC5A7**	0.41014	1.3171	0.22718490432862900	11071
**C10orf113**	0.40991	1.9066	0.22777654043359200	11072
**CEP152**	0.40991	1.2242	0.22777654043359200	11073
**FTSJ1**	0.59014	0.82386	0.22790516752134800	11075
**SALL3**	0.59014	1.0988	0.22790516752134800	11074
**CEP63**	0.59026	0.55107	0.2282138879204650	11076
**KIF3A**	0.40946	0.11218	0.22893432016226300	11077
**RER1**	0.40945	0.73944	0.2289600520805840	11078
**FBXL2**	0.40938	1.762	0.22914017975454300	11079
**OR51Q1**	0.59064	0.37559	0.22919164616950500	11080
**SGIP1**	0.40932	0.59228	0.22929458082075500	11081
**SEPT1**	0.40931	1.4697	0.2293203148631200	11082
**NEDD8-MDI**	0.40925	0.25495	0.2294747223070000	11083
**GLE1**	0.40923	0.30054	0.22952619267053300	11084
**ZNF311**	0.40921	1.0846	0.22957766364210300	11085
**PDE6B**	0.40916	1.0856	0.22970634373218300	11086
**ARHGDIB**	0.40906	1.433	0.22996371532529600	11087
**MMS22L**	0.40905	1.7396	0.22998945332211000	11088
**C15orf54**	0.409	0.72819	0.23011814559178800	11091
**GCNT3**	0.409	2.5371	0.23011814559178800	11089
**ONECUT3**	0.591	0.92527	0.2301181455917880	11090
**ACTG2**	0.40887	0.35218	0.2304527633346600	11099
**CGB7**	0.40887	0.32935	0.2304527633346600	11100
**CHURC1-FN**	0.40887	1.5489	0.2304527633346600	11096
**GABRG3**	0.40887	1.4139	0.2304527633346600	11097
**HOXB13**	0.40887	2.3525	0.2304527633346600	11092
**LRRC53**	0.40887	1.8224	0.2304527633346600	11095
**MMP2**	0.40887	1.8501	0.2304527633346600	11094
**SHB**	0.40887	1.8971	0.2304527633346600	11093
**UBALD1**	0.40887	1.031	0.2304527633346600	11098
**RBMS1**	0.40878	0.38864	0.23068443688567200	11101

**SYTL4**	0.40867	0.16208	0.23096761026378800	11102
**ANAPC4**	0.40865	0.15234	0.23101909832165400	11103
**ACSS2**	0.40859	0.25779	0.23117356617033900	11104
**GABARAPL2**	0.40854	0.73764	0.23130229359086400	11105
**PIGC**	0.40847	0.83187	0.2314825184192010	11106
**ELAVL2**	0.40841	1.0532	0.23163700282754100	11107
**SLC10A7**	0.40835	2.1732	0.2317914927639290	11108
**ANGPTL1**	0.40829	0.46834	0.23194598823244900	11109
**B4GALT2**	0.40823	0.54941	0.23210048923718600	11110
**C7orf49**	0.40817	2.0854	0.23225499578222400	11111
**CYP4A11**	0.40817	1.5339	0.23225499578222400	11114
**GGNBP2**	0.40817	1.7354	0.23225499578222400	11112
**MCRIP2**	0.40817	1.2937	0.23225499578222400	11115
**MT1HL1**	0.40817	1.5751	0.23225499578222400	11113
**CCND3**	0.40814	0.82818	0.23233225113363300	11116
**NUP35**	0.59191	0.41878	0.23246101313425300	11117
**STAC3**	0.59202	0.63832	0.2327443031052160	11118
**SPRN**	0.40792	0.27688	0.2328988327788410	11119
**MATK**	0.59213	1.0716	0.23302761175499900	11120
**OAF**	0.59213	0.52072	0.23302761175499900	11122
**STEAP4**	0.59213	0.60512	0.23302761175499900	11121
**SP1**	0.40786	0.52438	0.23305336801389100	11123
**FYTTD1**	0.59234	0.75089	0.23356852568244400	11125
**KRTAP10-2**	0.40766	2.1622	0.23356852568244400	11124
**DOCK9**	0.40764	1.9004	0.23362004485628500	11126
**RMND1**	0.40758	0.82684	0.2337746060987640	11127
**C2CD4A**	0.40755	0.54464	0.23385188881398100	11128
**MTUS1**	0.40748	1.7962	0.23403222058185800	11129
**MSANTD4**	0.40747	0.75631	0.23405798288410400	11130
**JRK**	0.40742	2.0568	0.2341867967257680	11131
**PDX1**	0.59267	0.3618	0.2344186714345700	11132
**UMAD1**	0.59269	0.52541	0.23447020085878000	11133

**KIAA1755**	0.59271	0.064058	0.2345217309055530	11134
**XPNPEP3**	0.59272	0.39009	0.2345474961624480	11135
**STK32B**	0.40704	1.1301	0.2351659090809150	11136
**C19orf47**	0.40702	1.546	0.23521744754715700	11137
**LAPTM4A**	0.407	0.91539	0.23526898663816700	11138
**NDUFAF3**	0.40696	1.7105	0.23537206669509300	11139
**CHAF1A**	0.59315	0.44432	0.23565554975051800	11140
**HMOX1**	0.40684	0.80093	0.23568132187608300	11141
**IL31RA**	0.59318	0.90525	0.2357328665968360	11142
**TRIM61**	0.5933	0.23114	0.23604214807871200	11143
**OIT3**	0.40666	0.52416	0.23614524692198900	11144
**PRKCSH**	0.40664	0.19759	0.23619679728480900	11145
**PRR23D2**	0.40658	0.47325	0.23635145213982500	11146
**ENPP7**	0.59348	0.47814	0.23650611264787300	11147
**GRXCR2**	0.4064	1.3809	0.23681545063952100	11148
**IQSEC3**	0.4064	0.11069	0.23681545063952100	11149
**LOC100996**	0.40638	0.62574	0.23686700917377500	11153
**RRAGD**	0.40638	2.0385	0.23686700917377500	11150
**SCAMP3**	0.40638	1.2869	0.23686700917377500	11152
**TRIL**	0.40638	1.971	0.23686700917377500	11151
**UGT1A8**	0.40636	1.0499	0.2369185683376670	11154
**TLN1**	0.59367	1.0112	0.236995908264411	11155
**STX18**	0.40629	0.31637	0.23709903037189400	11156
**GUF1**	0.40596	2.3592	0.23794988407447900	11157
**CDK11A**	0.40592	1.4658	0.23805302955055100	11158
**C17orf47**	0.59427	1.157	0.23854300516847000	11159
**TSPO**	0.59427	1.0882	0.23854300516847000	11160
**LAMB1**	0.59428	0.15982	0.23856879494424100	11161
**MRPS2**	0.40556	1.2376	0.23898145294854100	11162
**PHF10**	0.40554	1.5236	0.23903303805902000	11163
**CCDC27**	0.40548	1.6704	0.23918779720738300	11164
**PCDH8**	0.59458	1.1015	0.23934256208434700	11165

**STARD10**	0.4054	1.5854	0.23939415165040500	11166
**LILRA6**	0.59471	1.097	0.23967790565641000	11167
**PPIL6**	0.59471	0.83497	0.23967790565641000	11168
**PCNT**	0.40526	0.79857	0.23975529646084000	11169
**SDF4**	0.59476	0.38024	0.2398068911281710	11170
**DEFB129**	0.40519	0.90982	0.23993588058960900	11171
**PTAR1**	0.40512	0.1576	0.24011647254283700	11172
**BEST2**	0.5949	0.35002	0.24016807168182300	11173
**KLHL17**	0.40504	1.0968	0.24032287293591900	11174
**PIP4K2A**	0.59496	0.24042	0.24032287293591900	11175
**LMNTD2**	0.40499	1.18	0.2404518783800010	11176
**NCAPD3**	0.59504	1.1408	0.2405292835670890	11177
**FANCC**	0.40481	2.1531	0.24091633112953700	11178
**OR6T1**	0.40481	1.3542	0.24091633112953700	11179
**ZNF135**	0.40481	1.1493	0.24091633112953700	11180
**KANSL3**	0.5952	0.64085	0.24094213558293900	11181
**CAPSL**	0.40478	1.6664	0.24099374497105300	11182
**KCNG4**	0.59522	1.2427	0.24099374497105300	11183
**DNASE2**	0.40475	1.2439	0.24107116025678600	11184
**AIP**	0.59528	0.11722	0.24114857698725300	11185
**TRIOBP**	0.59539	1.1503	0.24143245070117800	11186
**PIPOX**	0.40457	0.27267	0.2415356823287410	11187
**RHOJ**	0.4045	0.5527	0.24171634387126600	11188
**URB1**	0.59559	1.0182	0.24194863459071700	11189
**RAG1**	0.40421	0.88446	0.24246488295176700	11190
**DYNLT1**	0.59581	0.25465	0.24251651133972300	11191
**BRAP**	0.40417	2.0877	0.24256814037408500	11192
**VHLL**	0.40413	1.1245	0.24267140038264300	11193
**NTM**	0.59589	1.1522	0.24272303135714700	11194
**CEBPE**	0.40408	0.8201	0.24280047903211600	11203
**EDA**	0.40408	0.94884	0.24280047903211600	11202
**GDA**	0.40408	1.4657	0.24280047903211600	11198

**GPR176**	0.40408	2.4149	0.24280047903211600	11195
**LTA4H**	0.40408	1.1301	0.24280047903211600	11201
**REG1B**	0.40408	1.4058	0.24280047903211600	11199
**SLC9A8**	0.40408	1.6384	0.24280047903211600	11197
**TBC1D25**	0.40408	1.2509	0.24280047903211600	11200
**XAGE5**	0.40408	2.0739	0.24280047903211600	11196
**C7**	0.40406	1.3965	0.2428521116244630	11204
**HOXD1**	0.404	1.247	0.24300701328655500	11205
**CNOT10**	0.40396	2.2144	0.24311028430036700	11206
**GORASP1**	0.40379	0.49145	0.24354921504880700	11207
**TEX44**	0.40378	0.29686	0.24357503596501500	11208
**MEGF8**	0.4036	1.6646	0.24403984024420200	11209
**GGT1**	0.59642	0.59094	0.2440914884168020	11210
**DPY19L1**	0.40356	0.74677	0.2441431372405060	11211
**BEND5**	0.59644	0.31144	0.24414313724050700	11212
**USP24**	0.40342	0.070346	0.2445046972502900	11213
**FASLG**	0.4034	1.1303	0.2445563512883750	11214
**CEACAM16**	0.40322	2.0061	0.2450212670117970	11215
**KLF1**	0.40318	1.102	0.24512458880573200	11216
**ACOT6**	0.59684	0.75506	0.24517625068390900	11218
**MBTPS1**	0.59684	0.84964	0.24517625068390900	11217
**EIF3M**	0.40311	1.9967	0.2453054082424440	11220
**GABRB1**	0.40311	1.4637	0.2453054082424440	11221
**MAGI3**	0.40311	2.3836	0.2453054082424440	11219
**ZBED8**	0.40306	1.9545	0.24543456989304700	11222
**CHRNA7**	0.40302	1.2377	0.2455379021614410	11223
**SPEN**	0.40301	0.95525	0.24556373563813300	11224
**MERTK**	0.40299	1.9945	0.24561540308316300	11225
**GPHN**	0.40291	1.727	0.24582207942141200	11226
**COQ8A**	0.40287	0.43768	0.2459254215275770	11227
**IGSF11**	0.40278	0.07295	0.24615795087090100	11228
**GOLGA6A**	0.59728	1.0475	0.24631297782666900	11229

**NGEF**	0.40261	0.30794	0.24659720929007600	11230
**ARAP1**	0.40255	1.0331	0.2467522530292270	11231
**METAP1D**	0.40253	1.4048	0.24680393559352800	11232
**UGGT1**	0.40252	1.2167	0.24682977712287500	11233
**NPAS4**	0.4025	0.84092	0.24688146067606000	11234
**ETFRF1**	0.40245	1.1643	0.2470106724444510	11235
**SYNPO**	0.40243	0.51702	0.24706235830638600	11236
**PGBD3**	0.4024	1.7172	0.2471398883368210	11237
**FCF1**	0.59764	0.13293	0.24724326402160900	11238
**CPSF3**	0.59774	0.3027	0.24750171479563300	11239
**LRRC71**	0.4022	1.7601	0.2476567931945160	11240
**RAP1A**	0.59782	0.49384	0.24770848731748500	11241
**DEFA4**	0.59789	0.4449	0.24788942196158900	11242
**UMOD**	0.40209	0.5281	0.2479411190644620	11243
**GPC1**	0.59794	0.67054	0.24801866596124600	11244
**ACSL4**	0.40203	1.5583	0.24809621434946700	11247
**IFRD1**	0.40203	2.2287	0.24809621434946700	11245
**ISG20**	0.40203	1.9016	0.24809621434946700	11246
**OR2Z1**	0.40203	0.91633	0.24809621434946700	11248
**OR5K1**	0.40203	0.76673	0.24809621434946700	11249
**DNAJA1**	0.402	1.2741	0.24817376422964600	11250
**ANGPTL7**	0.40194	2.0069	0.24832886846797800	11251
**TTC39B**	0.40181	1.1597	0.2486649481511900	11252
**HAUS1**	0.5982	0.054945	0.24869080159737200	11255
**MIGA2**	0.4018	2.3228	0.24869080159737200	11253
**WDR4**	0.4018	1.2621	0.24869080159737200	11254
**EPPIN-WFD**	0.59821	0.17679	0.24871665520977500	11256
**EXOSC6**	0.40175	2.0349	0.24882007132196700	11257
**TTC7B**	0.59826	0.86655	0.24884592576575900	11258
**KCNA6**	0.40172	1.9984	0.24889763515237000	11259
**TNC**	0.40162	0.16501	0.24915619207104100	11260
**GC**	0.40157	0.76565	0.2492854767755140	11261

**ENTPD5**	0.40156	0.68298	0.2493113342163300	11262
**HPS4**	0.59866	0.32289	0.24988024012028200	11263
**TMEM237**	0.40132	1.8877	0.2499319628464440	11264
**OAS2**	0.40119	2.0984	0.2502681768708460	11265
**AVEN**	0.40114	0.79321	0.2503974974880800	11266
**MDN1**	0.40106	1.047	0.2506044191868310	11267
**NKAIN4**	0.59897	0.015821	0.250682017589701	11268
**AMD 1.00**	0.401	1.0113	0.2507596175020280	11269
**HCN1**	0.4006	2.2136	0.2517944274937420	11270
**ZNF511**	0.5994	0.21995	0.25179442749374200	11271
**ALAS1**	0.59942	0.87712	0.2518461750622990	11273
**CDS1**	0.59942	1.1922	0.2518461750622990	11272
**KIAA0753**	0.59942	0.64284	0.2518461750622990	11274
**SRRM3**	0.59942	0.46638	0.2518461750622990	11275
**HOXB8**	0.59945	0.79934	0.25192379767964600	11276
**NGF**	0.59945	0.73764	0.25192379767964600	11277
**VCAM1**	0.40054	1.6184	0.25194967222270900	11278
**EWSR1**	0.40047	0.78659	0.25213079874809300	11279
**ARHGAP20**	0.40044	1.7853	0.2522084269335840	11280
**TUT1**	0.40044	1.4264	0.2522084269335840	11281
**ZNF618**	0.40044	1.3114	0.2522084269335840	11282
**KLHL13**	0.40038	0.85358	0.2523636878645420	11283
**BIN2**	0.40023	1.7229	0.25275186681639800	11286
**C10orf82**	0.40023	1.9019	0.25275186681639800	11285
**C1orf127**	0.40023	0.63375	0.25275186681639800	11291
**EGF**	0.40023	1.0466	0.25275186681639800	11289
**LPAR1**	0.40023	0.46897	0.25275186681639800	11292
**RASGEF1C**	0.40023	1.2531	0.25275186681639800	11288
**SLC39A2**	0.40023	0.8518	0.25275186681639800	11290
**TLR10**	0.40023	2.3601	0.25275186681639800	11284
**TYSND1**	0.40023	1.3803	0.25275186681639800	11287
**ADGRF1**	0.40003	0.9399	0.2532694980111750	11293

**GRM7**	0.59999	0.19366	0.25332126486080800	11294
**BNIP3L**	0.60001	0.61962	0.25337303238927700	11295
**BGN**	0.39992	0.42723	0.25355422408701400	11296
**EPB41L4A**	0.60011	0.51542	0.25363188021964400	11297
**GPX5**	0.39986	1.3827	0.2537095378817700	11298
**SFMBT2**	0.39979	1.1147	0.25389074504440300	11299
**SEC23B**	0.39973	0.30354	0.2540460721047110	11300
**DOPEY2**	0.60028	0.68866	0.2540719605438810	11301
**C6orf15**	0.39968	0.91736	0.2541755160035300	11302
**PCF11**	0.39967	2.1554	0.2542014052942800	11303
**RHOBTB3**	0.39957	0.83828	0.25446030757564100	11304
**CD109**	0.39955	0.71131	0.2545120900781360	11305
**ASL**	0.60046	0.40293	0.25453798158528800	11306
**MAGEA4**	0.39943	0.13305	0.2548227994298380	11307
**ITGA3**	0.39942	0.74497	0.2548486929858410	11308
**FXYD1**	0.39939	1.5262	0.2549263746791180	11309
**CLECL1**	0.60068	0.23242	0.25510763794668200	11310
**ZNF883**	0.39928	1.7405	0.25521122071963400	11311
**CHST15**	0.60074	0.12683	0.25526301313287000	11312
**IL22**	0.60102	0.93015	0.25598817888483000	11313
**FARP1**	0.39883	0.96378	0.2563767158928180	11314
**C9orf131**	0.39871	0.92823	0.25668757336390100	11315
**FAHD2A**	0.39867	1.8161	0.2567911980315700	11316
**NR2E3**	0.39867	1.6182	0.2567911980315700	11318
**TRIM38**	0.39867	1.7222	0.2567911980315700	11317
**NF2**	0.39866	2.1996	0.2568171046292860	11319
**SKIDA1**	0.3986	1.7003	0.25697254783579100	11320
**CHD1**	0.60152	1.1284	0.2572834528805190	11321
**ZNF142**	0.6016	0.706	0.25749073672526500	11322
**GPNMB**	0.6017	0.4755	0.25774985709072000	11324
**RAP2C**	0.3983	1.7508	0.25774985709072000	11323
**DOLPP1**	0.39823	0.086289	0.2579312516428850	11325

CCDC112	0.39821	1.2247	0.2579830802163430	11330
CD4	0.39821	1.7319	0.2579830802163430	11328
GK2	0.39821	1.0257	0.2579830802163430	11331
MSI2	0.39821	2.2069	0.2579830802163430	11326
PPIL4	0.39821	1.7873	0.2579830802163430	11327
SATB2	0.39821	0.81589	0.2579830802163430	11332
SLC17A7	0.39821	1.4424	0.2579830802163430	11329
RABGAP1L	0.39813	2.02	0.25819040144155400	11333
ENAM	0.60188	0.060684	0.2582163173748110	11334
EFR3B	0.39809	0.29966	0.2582940662151960	11335
ZNF304	0.60191	0.78702	0.2582940662151960	11336
AVP	0.39803	0.91061	0.25844956858050300	11337
FAM71C	0.60211	1.3227	0.2588124317442750	11338
IL1F10	0.60211	1.2325	0.2588124317442750	11339
MFSD5	0.39774	0.22977	0.25920125152503500	11340
GSDMD	0.39758	0.9651	0.2596160358210150	11342
XPOT	0.39758	1.2691	0.2596160358210150	11341
RDH8	0.6025	0.097716	0.2598234447149010	11343
ST8SIA3	0.39742	0.77836	0.2600308647862190	11344
TRNT1	0.39728	0.18978	0.2603938768371940	11346
UST	0.39728	0.48296	0.2603938768371940	11345
NCOA1	0.39719	1.3299	0.2606272598447620	11347
ZNF808	0.39719	1.2025	0.2606272598447620	11348
SERPINF2	0.39696	0.72004	0.2612237476164210	11349
LOC1005065	0.39693	1.7436	0.26130155721705100	11350
TIPARP	0.60307	0.50114	0.26130155721705100	11351
RNF14	0.3969	0.2863	0.26137936839966800	11352
SCMH1	0.60311	0.53749	0.26140530581218600	11353
ERV3-1	0.39685	0.54266	0.261509057221013	11354
RAB39A	0.39682	1.0629	0.26158687262488200	11355
KDM2B	0.60322	0.31233	0.26169062896072500	11356
SCN4A	0.60326	0.53555	0.2617943881137510	11357

**RAD9A**	0.39667	0.71004	0.26197597341421500	11358
**TMEM233**	0.39657	0.41731	0.262235395972844	11359
**TTC25**	0.39655	0.62423	0.2622872826018260	11360
**TONSL**	0.39654	1.9291	0.2623132261811030	11361
**ACAP3**	0.39648	1.5996	0.26246889136501900	11362
**RNF150**	0.39637	0.19189	0.2627542940572510	11363
**RNMT**	0.39622	0.75474	0.26314351404506400	11364
**RAD51C**	0.60383	0.37528	0.26327326289702100	11365
**CD83**	0.60397	0.29856	0.2636365832695080	11366
**FAM71E2**	0.39599	1.8317	0.26374039548016500	11367
**CPB1**	0.60405	0.39442	0.2638442105331340	11368
**CABP5**	0.39592	2.2697	0.2639220736888950	11369
**WSCD1**	0.39584	2.0064	0.2641297165948790	11370
**AAMDC**	0.39582	1.5054	0.2641816291004100	11377
**ARHGAP24**	0.39582	1.4471	0.2641816291004100	11378
**B3GNT4**	0.39582	1.2319	0.2641816291004100	11379
**C1orf216**	0.39582	2.023	0.2641816291004100	11373
**GPD2**	0.39582	0.88101	0.2641816291004100	11381
**INTS1**	0.39582	1.9653	0.2641816291004100	11374
**LHPP**	0.39582	2.1471	0.2641816291004100	11372
**MAK16**	0.39582	0.39755	0.2641816291004100	11382
**PDGFB**	0.39582	2.1486	0.2641816291004100	11371
**PIKFYVE**	0.39582	1.7016	0.2641816291004100	11376
**RDH14**	0.39582	1.9577	0.2641816291004100	11375
**SLC22A4**	0.39582	0.98475	0.2641816291004100	11380
**CTNND2**	0.39572	0.44243	0.2644412023102470	11383
**FANCB**	0.39564	0.41884	0.2646488737063030	11384
**GMPR**	0.3956	1.9376	0.2647527136839040	11385
**C9orf66**	0.39558	0.67832	0.26480463474315600	11386
**PHLDB3**	0.39547	1.5533	0.26509021333333900	11387
**CCL26**	0.60455	0.43633	0.2651421390354510	11388
**ANO8**	0.60456	0.1532	0.26516810215457900	11390

**CLK3**	0.39544	1.0193	0.26516810215457900	11389
**FCGBP**	0.3953	2.1902	0.26553160459928200	11391
**USP46**	0.39524	0.69804	0.26568740209934900	11392
**TCEANC**	0.3951	1.1706	0.2660509546866610	11393
**PLXNC1**	0.39504	0.1613	0.2662067737003980	11394
**EFNB3**	0.60502	0.70377	0.26636259917759200	11398
**HIGD2A**	0.60502	1.1137	0.26636259917759200	11396
**SRMS**	0.60502	1.1937	0.26636259917759200	11395
**ZFP28**	0.39498	1.0175	0.26636259917759200	11397
**SLC18A3**	0.39487	1.8454	0.2666482960204920	11399
**NSUN7**	0.39478	0.41167	0.26688206417091900	11400
**C2orf49**	0.60526	0.58728	0.26698596580737800	11401
**KCNH5**	0.39466	1.8074	0.2671937777283410	11403
**SLC4A1AP**	0.39466	2.3603	0.2671937777283410	11402
**ATP6V0C**	0.39454	2.0485	0.2675055172491300	11404
**CFDP1**	0.60548	0.67832	0.26755747636257500	11405
**PPME1**	0.39449	0.84834	0.2676354163871560	11406
**NTRK1**	0.60552	0.51298	0.2676613967566060	11407
**SLX4**	0.39433	1.3412	0.26805112398902700	11408
**NUP160**	0.39432	1.1539	0.26807710725147500	11410
**OR5I1**	0.39432	1.2732	0.26807710725147500	11409
**MVB12B**	0.39424	0.34317	0.26828497986812600	11411
**LCE6A**	0.6058	0.80715	0.2683889205232500	11412
**SDAD1**	0.60582	0.89753	0.2684408919380740	11413
**TNIP1**	0.39409	1.6109	0.2686747722796630	11414
**ELP6**	0.60596	0.20321	0.2688047121523900	11415
**SLITRK4**	0.60604	0.8058	0.2690126253901390	11416
**RUFY3**	0.39388	0.015142	0.26922055025705900	11417
**TMEM129**	0.39385	2.4288	0.26929852508240000	11418
**MRPL32**	0.39374	1.9384	0.2695844467878160	11419
**INO80**	0.6063	0.51725	0.2696884237800950	11420
**TGM3**	0.39363	0.66033	0.26987039053316000	11421

**COPZ2**	0.60653	0.47888	0.27028634810726700	11422
**KMT2B**	0.60659	0.69802	0.2704423442507770	11423
**KRTAP24-1**	0.60663	0.91353	0.27054634533576600	11424
**LSM 12.00**	0.39336	2.1091	0.2705723460641960	11425
**C11orf74**	0.39336	2.4345	0.2705723460641960	11427
**C15orf38-AI**	0.39336	2.0895	0.2705723460641960	11429
**DNAJC15**	0.39336	1.5198	0.2705723460641960	11431
**HMX2**	0.39336	0.39657	0.2705723460641960	11432
**KLHL25**	0.39336	0.19025	0.2705723460641960	11426
**PXDNL**	0.39336	1.3233	0.2705723460641960	11430
**RASGRP3**	0.39336	1.9864	0.2705723460641960	11428
**PLEKHG2**	0.39332	1.1435	0.2706763508072490	11433
**TOR3A**	0.39317	1.2389	0.2710663946808660	11434
**USMG5**	0.39315	1.5782	0.2711184036452340	11435
**MMP16**	0.39305	0.83388	0.27137845947050600	11437
**VRK2**	0.39305	0.8656	0.27137845947050600	11436
**PEBP1**	0.39299	1.4615	0.27153450177422500	11438
**SPACA1**	0.39289	1.3326	0.27179458697534300	11439
**MS4A14**	0.39286	1.5925	0.27187261612006000	11440
**CPQ**	0.39277	1.7779	0.2721067134881820	11441
**PKDREJ**	0.60739	0.42411	0.27252292341728500	11442
**PDHA1**	0.60748	0.29952	0.27275706224517800	11443
**CIPC**	0.39248	0.56766	0.27286112874543100	11444
**ZNF7**	0.60764	0.23506	0.2731733459816430	11445
**POLR3F**	0.39225	0.61709	0.273459568508037	11446
**TDRD10**	0.39221	1.168	0.2735636549799870	11447
**UCN2**	0.39213	0.23792	0.2737718368165590	11448
**LRIG1**	0.60794	1.1683	0.2739540056566590	11449
**MIS18A**	0.60794	0.85863	0.2739540056566590	11451
**PKM**	0.60794	0.99102	0.2739540056566590	11450
**ZNF787**	0.60794	0.53688	0.2739540056566590	11452
**CTRL**	0.39204	0.62802	0.27400605556625200	11453

**AKAP6**	0.392	0.10306	0.274110157612579	11454
**FAT2**	0.39195	0.70261	0.2742402893480500	11455
**NPAS3**	0.39186	1.9506	0.27447453817673800	11456
**SOX3**	0.60819	0.56953	0.27460468292208400	11457
**HMP19**	0.39177	1.3323	0.2747088020671060	11458
**HSPA12A**	0.39161	1.7382	0.27512530844087800	11459
**MYOT**	0.39159	1.22	0.27517737509207000	11460
**CLDN34**	0.60842	0.66249	0.275203408697398	11461
**CCDC137**	0.60843	0.33885	0.27522944248924200	11462
**GRHPR**	0.39156	0.85039	0.27525547646762100	11463
**FCHSD2**	0.60846	0.11544	0.275307544984066	11464
**MICALL2**	0.39152	1.0351	0.2753596142468980	11465
**SLC27A4**	0.60851	0.68055	0.27543771954097500	11466
**CELF3**	0.39137	0.77505	0.27575015752560200	11467
**HTR6**	0.39136	2.0151	0.27577619523896000	11468
**MARCH6**	0.60865	0.58405	0.27580223313928000	11469
**ZNF793**	0.60872	0.021086	0.2759845036781810	11470
**LENG8**	0.60873	0.27618	0.27601054307493300	11471
**OR1S1**	0.39125	1.09	0.27606262242989400	11472
**OR1G1**	0.60878	0.18385	0.27614074286628400	11473
**TPR**	0.60879	0.60771	0.2761667833862160	11474
**SHC4**	0.3909	1.483	0.2769741324898130	11475
**AMY2A**	0.60912	0.035867	0.27702622572292400	11476
**TMEM246**	0.60919	0.392	0.2772085579599880	11477
**AGR3**	0.39072	0.95949	0.2774429986648500	11484
**CEACAM6**	0.39072	1.5945	0.2774429986648500	11482
**DAGLA**	0.39072	0.027339	0.2774429986648500	11485
**ITIH4**	0.39072	1.6698	0.2774429986648500	11481
**NHLRC1**	0.39072	2.1135	0.2774429986648500	11478
**PNKD**	0.39072	1.1262	0.2774429986648500	11483
**TMEM87B**	0.39072	1.9505	0.2774429986648500	11479
**TTC27**	0.39072	1.9067	0.2774429986648500	11480

**OR13C2**	0.60929	0.58264	0.27746904857311600	11486
**PPP2R5E**	0.60951	0.065249	0.27804219422758400	11487
**SYNC**	0.60961	0.72056	0.2783027451642410	11488
**ANKAR**	0.60978	1.1078	0.2787457251291960	11489
**C17orf82**	0.3902	1.7879	0.2787978440137080	11490
**FNDC5**	0.39014	0.63507	0.2789542052117740	11491
**TFAP4**	0.39009	1.2659	0.27908451141987600	11492
**CD200R1**	0.39005	2.1719	0.2791887597981920	11494
**KCNG2**	0.39005	2.5134	0.2791887597981920	11493
**LRRC8E**	0.39005	1.9846	0.2791887597981920	11495
**CDK1**	0.60999	0.84495	0.27929301121067000	11496
**CD86**	0.61003	0.87027	0.27939726565861900	11497
**PTPN18**	0.38996	2.1501	0.27942332974505200	11498
**C1orf52**	0.38989	1.091	0.2796057836661700	11499
**JAZF1**	0.61012	0.709	0.27963184927173100	11500
**SVOP**	0.61022	0.69205	0.2798925157797890	11501
**GPR87**	0.38974	0.12924	0.27999678770712800	11502
**AMMECR1**	0.38971	2.0282	0.280074993650394	11503
**CYC1**	0.61035	0.018788	0.28023141067642900	11505
**TEPP**	0.38965	0.22527	0.28023141067642900	11504
**CHSY1**	0.38962	1.8181	0.2803096217603040	11506
**CNDP2**	0.38957	1.0236	0.2804399773773550	11507
**GEM**	0.38943	1.6748	0.28080499846553900	11508
**SREBF1**	0.3894	0.36552	0.2808832221363270	11509
**FSTL3**	0.38938	0.42445	0.28093537220498800	11510
**GALR1**	0.38927	1.5757	0.2812222112441240	11511
**IFT81**	0.38913	0.62059	0.28158731258170200	11512
**QSOX2**	0.38901	0.72195	0.2819002864606190	11513
**TRAK2**	0.38898	0.29264	0.28197853424375300	11514
**ENO1**	0.61108	0.13729	0.28213503498999500	11515
**SEC14L2**	0.38889	1.8676	0.2822132879542140	11516
**DCLK2**	0.61119	0.76031	0.2824219709756520	11517

**TK2**	0.38874	0.983	0.28260457870850500	11518
**ADRB2**	0.61135	0.68869	0.2828393739276470	11519
**CYB561**	0.38861	1.5034	0.28294373236273300	11523
**FOXD4L4**	0.38861	1.6003	0.28294373236273300	11522
**IL12RB2**	0.61139	0.11796	0.28294373236273300	11525
**SLC1A3**	0.38861	1.7051	0.28294373236273300	11521
**UBE2Q2L**	0.38861	1.3606	0.28294373236273300	11524
**USP3**	0.38861	2.5392	0.28294373236273300	11520
**MTAP**	0.61149	0.27774	0.2832046419347710	11526
**MPZL1**	0.38843	0.96068	0.2834133834728740	11527
**STPG3**	0.61163	0.1371	0.28356994773032000	11528
**SERTAD2**	0.38829	0.83055	0.28377871088721100	11529
**DEK**	0.3882	1.1243	0.28401358422300400	11538
**ERLIN2**	0.3882	1.1817	0.28401358422300400	11536
**FAM3D**	0.3882	1.5237	0.28401358422300400	11534
**KRTAP5-7**	0.3882	1.1552	0.28401358422300400	11537
**MDP1**	0.3882	1.635	0.28401358422300400	11533
**MSRA**	0.3882	1.7108	0.28401358422300400	11531
**PHLPP1**	0.3882	1.653	0.28401358422300400	11532
**PILRB**	0.3882	1.3434	0.28401358422300400	11535
**RNPS1**	0.3882	1.8559	0.28401358422300400	11530
**SEL1L**	0.3882	0.90771	0.28401358422300400	11539
**HIF1A**	0.38815	0.50937	0.2841440761789070	11540
**ADCYAP1R1**	0.38812	0.52994	0.2842223736747730	11541
**CLIC5**	0.61197	0.39147	0.2844572766191530	11544
**PAPD7**	0.61197	0.74219	0.2844572766191530	11543
**ZNF324**	0.61197	1.2526	0.2844572766191530	11542
**FAM161B**	0.38799	1.7279	0.2845616829653750	11545
**YES1**	0.38794	0.59087	0.28469219526041500	11546
**RPS26**	0.38792	1.3655	0.2847444015361080	11548
**WFDC13**	0.38792	1.935	0.2847444015361080	11547
**HIST2H2BF**	0.3879	0.73133	0.28479660858786600	11549

**HDAC7**	0.38783	0.89875	0.28497933938291000	11550
**ISCA1**	0.61221	0.21178	0.28508376125206800	11551
**TRAF4**	0.38775	0.94912	0.28518818622978500	11552
**FITM1**	0.38767	2.2715	0.2853970455161860	11553
**ERMN**	0.6124	0.80164	0.2855798076044140	11554
**C7orf34**	0.38759	0.89875	0.285605917252708	11555
**RPN2**	0.61243	0.0053159	0.28565813713341200	11556
**ACVR1B**	0.61246	0.65514	0.28573646841507000	11557
**ZNF714**	0.38753	0.34224	0.2857625792318660	11558
**PTGES**	0.38749	0.49075	0.285867024447488	11559
**LSM 8.00**	0.61253	0.14244	0.2859192482246510	11560
**ZDHHC22**	0.38745	1.1661	0.28597147278160300	11561
**OR6Y1**	0.38739	0.59523	0.2861281511328520	11562
**EDA2R**	0.38728	1.0293	0.28641541302310600	11563
**GNRH1**	0.3872	1.9036	0.2866243456058180	11565
**REP15**	0.3872	2.4208	0.2866243456058180	11564
**RNF149**	0.3872	1.532	0.2866243456058180	11566
**AMIGO1**	0.61282	0.0010397	0.28667658070615100	11567
**KSR1**	0.61284	0.71512	0.2867288165886780	11568
**HDGFL1**	0.38713	2.1929	0.2868071718794450	11569
**TM9SF2**	0.61288	0.31768	0.28683329070097700	11570
**CYP7B1**	0.38702	0.39074	0.28709448968173800	11571
**MYO1H**	0.38701	0.73829	0.2871206106568150	11572
**MAML3**	0.61306	0.55275	0.28730346296874900	11573
**CLIC1**	0.38693	1.7339	0.28732958551161700	11574
**NAP1L4**	0.61307	0.032176	0.28732958551161700	11576
**PODXL**	0.61307	0.50827	0.28732958551161700	11575
**CDX2**	0.61311	0.55082	0.2874340776439720	11577
**C20orf203**	0.38686	1.9112	0.28751244880274700	11578
**LIMS4**	0.38684	0.86264	0.2875646972229590	11579
**PPP1R26**	0.3868	0.16593	0.2876691964186020	11580
**SYNPO2**	0.38655	0.66433	0.28832238761648600	11581

**PTPRR**	0.38653	2.2937	0.2883746482231620	11582
**PRNP**	0.3865	0.92841	0.28845304060997000	11583
**NFYA**	0.61357	0.15438	0.28863596307381300	11584
**ZNF20**	0.38639	1.7123	0.2887404945319100	11585
**RGS9**	0.38624	2.6732	0.28913251561030600	11586
**AMPH**	0.61378	0.25035	0.2891847884434420	11587
**AFM**	0.3861	2.1982	0.2894984420418470	11590
**CXXC5**	0.3861	1.0444	0.2894984420418470	11596
**DCLRE1C**	0.3861	1.5657	0.2894984420418470	11594
**FAM169A**	0.3861	2.0762	0.2894984420418470	11591
**STOML3**	0.3861	0.11188	0.2894984420418470	11597
**TEAD4**	0.3861	1.8844	0.2894984420418470	11592
**TERF2**	0.3861	1.1661	0.2894984420418470	11595
**TMEM44**	0.3861	2.4419	0.2894984420418470	11588
**TRRAP**	0.3861	1.7122	0.2894984420418470	11593
**VEGFA**	0.3861	2.349	0.2894984420418470	11589
**FXR2**	0.61395	0.74224	0.289629139445396	11598
**C16orf47**	0.38603	2.617	0.28968141979196400	11599
**NBL1**	0.38603	2.1071	0.28968141979196400	11601
**RBM39**	0.38603	2.2943	0.28968141979196400	11600
**HTR3B**	0.38599	0.44271	0.2897859828605570	11602
**SESN3**	0.6141	0.65782	0.29002126135135000	11603
**HIST1H4G**	0.61424	0.30529	0.2903872820325690	11604
**RTEL1**	0.38573	0.59447	0.290465720095599	11605
**MORN1**	0.61441	0.23509	0.29083178802835400	11606
**FAM168B**	0.38554	0.65355	0.2909625360186270	11607
**CBY3**	0.61474	0.44705	0.29169481675976500	11608
**NMU**	0.38523	2.0273	0.2917732846815770	11609
**SARNP**	0.3852	1.9078	0.29185175439990500	11610
**RASGEF1B**	0.38514	0.37787	0.2920086992283770	11611
**AAGAB**	0.38512	0.98532	0.29206101576939100	11612
**FAM53C**	0.61488	0.049452	0.29206101576939100	11613

**SNRK**	0.61489	0.17316	0.2920871743396520	11614
**BICDL1**	0.38508	0.56864	0.29216565124970800	11615
**GAPVD1**	0.61498	0.33437	0.29232261046842100	11616
**ACSM5**	0.61502	0.67821	0.2924272539483460	11617
**NAV3**	0.38478	1.3609	0.2929505194076910	11618
**BHMT2**	0.61523	0.53074	0.2929766847851070	11619
**CLOCK**	0.38472	1.4125	0.2931075146812680	11625
**IFNA1**	0.38472	1.7043	0.2931075146812680	11623
**IRAK1**	0.38472	2.1633	0.2931075146812680	11622
**PENK**	0.38472	2.3174	0.2931075146812680	11620
**SLC23A2**	0.38472	1.6395	0.2931075146812680	11624
**ZNF121**	0.38472	2.2632	0.2931075146812680	11621
**TIMELESS**	0.38455	1.5594	0.29355237387155300	11626
**CEP97**	0.3845	0.36315	0.2936832258624270	11627
**INTS8**	0.61564	0.23881	0.29404963819750400	11628
**ARL5A**	0.61569	0.45792	0.2941805093111030	11629
**NME9**	0.38429	0.66364	0.2942328591672050	11630
**PRB4**	0.38422	0.42106	0.29441609001463400	11631
**OR52L1**	0.6158	0.2689	0.29446844350061400	11632
**CAP1**	0.38407	0.769	0.29480876084014200	11633
**C15orf48**	0.61595	0.78738	0.2948611203835000	11635
**ETHE1**	0.61595	1.0033	0.2948611203835000	11634
**PRKAR1B**	0.61595	0.60269	0.2948611203835000	11636
**GLI4**	0.61598	0.041034	0.2949396612142740	11637
**CST5**	0.61602	0.14424	0.29504438515223800	11639
**RPL8**	0.38398	0.9647	0.29504438515223800	11638
**DHX34**	0.38396	1.8153	0.29509674833456900	11640
**CLC**	0.38378	0.24792	0.2955680534064040	11641
**ATAD1**	0.61625	0.29288	0.2956466106322000	11642
**BTG4**	0.38371	1.5089	0.2957513564381610	11643
**ZNF18**	0.38368	2.0062	0.29582991792204700	11644
**CA2**	0.38367	0.81544	0.2958561054890570	11650

**CFL2**	0.38367	1.9193	0.2958561054890570	11646
**CLEC2B**	0.38367	0.39958	0.2958561054890570	11651
**LARS**	0.38367	0.23767	0.2958561054890570	11652
**OC90**	0.38367	1.6645	0.2958561054890570	11647
**PRUNE2**	0.38367	2.0086	0.2958561054890570	11645
**TNK1**	0.38367	1.4588	0.2958561054890570	11648
**ZNF408**	0.38367	1.0806	0.2958561054890570	11649
**SLC25A16**	0.38356	0.70932	0.29614418212173900	11653
**SRPX**	0.38352	1.0165	0.2962489433531670	11654
**PRPF31**	0.38338	1.5678	0.2966156332764330	11655
**SERP2**	0.38338	1.0673	0.2966156332764330	11656
**PNPLA2**	0.38337	1.0315	0.2966418269394130	11657
**LRRC74B**	0.38334	1.53	0.29672040914959200	11658
**HDLBP**	0.61667	0.03945	0.296746603626803	11659
**EFCAB12**	0.38326	0.56447	0.29692997066957200	11660
**NDUFV3**	0.61676	0.59209	0.2969823630867340	11661
**NOC4L**	0.61685	0.17649	0.2972181390545680	11662
**OR7A17**	0.38312	1.6738	0.29729673471493400	11663
**B3GALT5**	0.6169	0.37407	0.29734913284208500	11664
**UNKL**	0.38309	1.7571	0.29737533221179600	11665
**VSIG8**	0.38303	1.7669	0.2975325327172960	11666
**TMEM260**	0.38301	0.91481	0.29758493451953400	11667
**STX6**	0.61702	0.72895	0.297663538755106	11668
**XCL1**	0.38284	1.5352	0.2980303828481780	11669
**LSM 10.00**	0.61725	0.35239	0.29826623237114900	11670
**RHOD**	0.38268	2.2401	0.29844968235920200	11671
**C5**	0.38262	0.5008	0.29860693320033400	11672
**RFX7**	0.38258	0.052594	0.29871177119643100	11673
**PAQR3**	0.3825	0.23657	0.2989214570395570	11674
**CNGA4**	0.61751	0.68512	0.2989476686938870	11675
**MPC2**	0.61759	0.8421	0.29915736932436400	11676
**ARHGAP11B**	0.61767	0.42332	0.29936708311070400	11677

**ZNF763**	0.61768	0.069387	0.29939329825956000	11678
**TLK2**	0.3823	0.98277	0.2994457291745470	11679
**DHX35**	0.38228	1.551	0.2994981609127110	11680
**NAPA**	0.38219	0.60655	0.29973411392581100	11681
**ELF2**	0.61793	0.35544	0.3000487439054660	11682
**CBLL1**	0.38206	1.4247	0.3000749644107700	11683
**ACACA**	0.38196	2.4	0.30033718081406100	11684
**LDHAL6A**	0.38196	1.629	0.30033718081406100	11686
**NCL**	0.38196	2.0311	0.30033718081406100	11685
**ZNF664**	0.61811	0.41364	0.30052074458286900	11687
**CAPN10**	0.38184	1.2181	0.3006518677601470	11688
**DEPDC1**	0.38151	0.48058	0.30151741050133400	11689
**C1orf210**	0.38145	1.0281	0.30167480616107000	11697
**CLUH**	0.38145	1.0656	0.30167480616107000	11696
**DMP1**	0.38145	1.519	0.30167480616107000	11691
**FKBP10**	0.38145	1.4334	0.30167480616107000	11694
**IFT74**	0.38145	2.426	0.30167480616107000	11690
**NPTXR**	0.38145	1.4742	0.30167480616107000	11693
**SAC3D1**	0.38145	1.4801	0.30167480616107000	11692
**USP31**	0.38145	1.276	0.30167480616107000	11695
**SSR4**	0.38137	2.2205	0.30188467866734600	11698
**PSMD5**	0.38132	0.53521	0.302015855735759	11699
**SNRNP48**	0.38119	1.049	0.3023569404424480	11700
**LRRC23**	0.61882	0.08181	0.30238317918409500	11701
**DPP9-AS1**	0.38113	0.49821	0.3025143760154680	11702
**MTHFD2**	0.61903	0.38936	0.30293424088134800	11703
**DUS4L**	0.38078	0.88869	0.3034328997860850	11704
**CACNA1F**	0.38077	0.88627	0.30345914708067600	11705
**COG2**	0.61926	0.1031	0.30353789021888400	11707
**TMEM158**	0.61926	0.3191	0.30353789021888400	11706
**NT5M**	0.61929	0.49055	0.3036166352391900	11708
**AP1S3**	0.38069	1.8996	0.3036691329652900	11709

**KDR**	0.61938	0.40929	0.3038528815984830	11710
**ACER2**	0.6194	0.16545	0.30390538309205600	11711
**GLYCTK**	0.3806	0.13212	0.30390538309205700	11712
**TTC16**	0.38059	0.31135	0.30393163415296600	11713
**YWHAH**	0.61943	0.38056	0.30398413690313700	11714
**ZNF713**	0.38044	0.20757	0.3043254252118240	11715
**PTH2**	0.38037	1.4903	0.30450921052013100	11716
**ZNF197**	0.38037	1.4169	0.30450921052013100	11717
**S100A9**	0.3803	0.6113	0.30469300611428500	11718
**MOB3C**	0.61972	0.68997	0.30474552103145400	11719
**FAM53A**	0.61974	0.13516	0.3047980367890550	11720
**UBE2H**	0.61983	0.27674	0.3050343681032360	11721
**SPRR4**	0.38011	2.0431	0.30519193177723200	11722
**TSR2**	0.38005	0.9132	0.3053495030282750	11723
**PKHD1**	0.38003	1.3494	0.3054020284632130	11724
**PPP4R4**	0.37999	2.2592	0.30550708186100600	11725
**PAX7**	0.62028	0.62102	0.30621628055622200	11726
**C10orf107**	0.3797	1.8676	0.30626881991576100	11727
**ARHGEF39**	0.6204	0.68649	0.30653152939822600	11729
**VPS18**	0.3796	2.1289	0.3065315293982260	11728
**C15orf52**	0.62046	0.30763	0.3066891652417220	11730
**BAG3**	0.37951	0.17521	0.30676798602113100	11735
**BTG3**	0.37951	1.8988	0.30676798602113100	11732
**CD180**	0.37951	1.9548	0.30676798602113100	11731
**OR52M1**	0.37951	1.5881	0.30676798602113100	11733
**ZNF264**	0.37951	0.60742	0.30676798602113100	11734
**GP5**	0.37948	0.98871	0.3068468087064210	11736
**PSMF1**	0.37934	0.75555	0.3072146731215420	11737
**NELFB**	0.62069	1.0004	0.30729350661782700	11738
**CNNM1**	0.37924	0.47265	0.3074774588703570	11739
**MBP**	0.37918	0.21161	0.3076351405109220	11740
**CSNK1G1**	0.37916	0.82093	0.3076877027573590	11741

**IL36G**	0.37906	0.11946	0.3079505267441800	11742
**FOXN4**	0.37897	1.0289	0.3081870865206060	11743
**FBXO25**	0.62109	0.1239	0.3083448026191070	11744
**CDCP2**	0.3789	1.4428	0.30837108938110700	11748
**PTPRM**	0.3789	1.8912	0.30837108938110700	11747
**RERG**	0.3789	2.3725	0.30837108938110700	11745
**VPS13D**	0.3789	2.2031	0.30837108938110700	11746
**UROD**	0.37884	1.6242	0.3085288144285750	11749
**FAM76A**	0.37876	0.93119	0.30873912643250100	11750
**COG8**	0.62135	0.70632	0.3090283277388160	11751
**ZNF316**	0.62138	0.54352	0.3091072053072140	11752
**XAGE3**	0.37846	1.802	0.30952791816672600	11753
**HABP2**	0.37834	1.7269	0.30984348876419400	11754
**HPS3**	0.62169	0.006736	0.3099223862337120	11755
**NKAIN1**	0.37821	0.92872	0.3101853917353960	11756
**LCN12**	0.62191	0.48008	0.31050102666334800	11758
**WWTR1**	0.37809	1.734	0.31050102666334800	11757
**FBXL16**	0.62195	0.69352	0.3106062451789110	11759
**SLC7A5**	0.62197	0.68013	0.3106588557261440	11760
**IL12B**	0.37801	1.1278	0.31071146713324500	11761
**CD7**	0.62203	0.44156	0.3108166925277410	11763
**CTSL**	0.37797	0.75233	0.3108166925277410	11762
**SLC35D1**	0.37784	1.5971	0.31115869883527200	11764
**MSS51**	0.3778	0.8828	0.31126393886596800	11765
**KRTAP5-2**	0.37775	0.42359	0.311395493752497	11766
**AZIN2**	0.37771	1.3656	0.3115007415419430	11767
**ANGEL1**	0.62237	0.46744	0.31171124747401400	11768
**SELENOF**	0.37759	0.82016	0.31181650561942300	11769
**GPR4**	0.37756	0.49754	0.31189545149560000	11770
**MAGEA8**	0.37753	2.0035	0.31197439931567400	11771
**TTN**	0.37746	1.0625	0.31215861845875100	11772
**C1QTNF1**	0.62256	0.01954	0.3122112544451620	11773

**TCF21**	0.3774	0.5041	0.31231652901315600	11774
**ATP8B4**	0.37739	2.4306	0.31234284819594300	11776
**C19orf60**	0.37739	1.9812	0.31234284819594300	11777
**POLR2D**	0.37739	1.606	0.31234284819594300	11778
**ZNF528**	0.37739	2.5369	0.31234284819594300	11775
**ARHGEF19**	0.37738	2.2278	0.31236916759508900	11780
**ATP6V1A**	0.37738	0.052135	0.31236916759508900	11789
**CEP112**	0.37738	2.3096	0.31236916759508900	11779
**DLK2**	0.37738	1.1171	0.31236916759508900	11786
**HDHD5**	0.37738	2.0778	0.31236916759508900	11781
**IL15**	0.37738	1.5675	0.31236916759508900	11784
**LIPG**	0.37738	1.0322	0.31236916759508900	11787
**OR51F2**	0.37738	1.2993	0.31236916759508900	11785
**RPAP2**	0.37738	1.6601	0.31236916759508900	11782
**SLC18A2**	0.37738	0.42018	0.31236916759508900	11788
**SLC6A5**	0.37738	1.6126	0.31236916759508900	11783
**TMEM123**	0.62263	0.54321	0.31239548721061600	11790
**ID2**	0.37731	1.0282	0.3125534094489910	11791
**RWDD3**	0.37728	0.8562	0.31263237349108300	11792
**SSSCA1**	0.62278	0.50151	0.31279030742402100	11793
**ACHE**	0.37718	1.7356	0.3128956010469470	11794
**SETD6**	0.62282	0.083332	0.31289560104694700	11795
**PCYOX1**	0.62286	0.73502	0.3130008981389400	11796
**JUND**	0.62294	0.68843	0.3132115027357170	11797
**NEURL2**	0.37705	0.74749	0.31323782928693800	11798
**CD1A**	0.37696	0.82277	0.3134747780200740	11799
**RNF166**	0.3769	1.8784	0.31363275361954400	11800
**TBC1D10B**	0.62314	0.61572	0.31373807503412600	11801
**IRF2BP2**	0.37681	0.65886	0.3138697316966150	11802
**UCP2**	0.37678	2.1145	0.3139487283054300	11803
**MFSD1**	0.37674	2.5443	0.3140540601649470	11804
**SMC1B**	0.37674	0.70248	0.3140540601649470	11805

**ALKBH2**	0.62331	1.1201	0.3141857298895070	11806
**CYS1**	0.37668	1.6469	0.31421206448799200	11807
**KRTAP10-6**	0.37666	1.1285	0.31426473433871200	11808
**SSR2**	0.37665	1.1412	0.31429106959099000	11809
**FLII**	0.62343	0.232	0.3145017594581330	11810
**TMEM11**	0.37656	0.81838	0.3145280966729690	11811
**ATP5E**	0.37649	0.084424	0.31471246328684800	11812
**ZSWIM6**	0.37636	1.0629	0.3150548868148090	11813
**TMEM156**	0.37629	1.2862	0.31523928401407000	11814
**RPS19**	0.37622	1.8952	0.3154236919327070	11815
**ZFP64**	0.37619	1.9668	0.31550272718114100	11816
**ZNF155**	0.37616	1.526	0.3155817644004140	11817
**ASTL**	0.37596	2.0766	0.3161087296051400	11820
**COA7**	0.37596	2.3777	0.3161087296051400	11819
**MARC2**	0.37596	2.5076	0.3161087296051400	11818
**TSTD1**	0.37596	1.1547	0.3161087296051400	11821
**MT1F**	0.62409	0.28021	0.31624048461737600	11822
**C15orf61**	0.62421	0.51904	0.31655671905080500	11823
**GABBR2**	0.37566	2.1119	0.3168993420821320	11824
**RSU1**	0.37559	0.76076	0.31708384681381900	11825
**SSMEM1**	0.37552	1.0828	0.3172683623401920	11827
**TET1**	0.37552	2.1222	0.3172683623401920	11826
**ZBTB25**	0.62458	0.9467	0.3175319746897360	11828
**SOD1**	0.37538	0.40842	0.3176374258071890	11829
**MROH6**	0.62464	0.23923	0.31769015269039800	11830
**TXNL4B**	0.37535	1.1529	0.31771651646320200	11831
**MYO1D**	0.37532	1.3407	0.31779560910666600	11832
**CATSPERG**	0.37516	0.036915	0.3182174701288900	11833
**GLT6D1**	0.37511	1.1017	0.31834931330954300	11834
**TRMT10B**	0.62499	0.38474	0.3186130162753770	11835
**C5AR1**	0.62503	0.070807	0.318718503664645	11836
**LRRC15**	0.62508	0.52256	0.31885036788887700	11837

**CDCA2**	0.37491	1.4279	0.3188767413989640	11838
**AMN**	0.3749	0.90299	0.3189031151308490	11846
**CCDC141**	0.3749	1.2317	0.3189031151308490	11844
**CTCFL**	0.3749	1.531	0.3189031151308490	11842
**FBXO17**	0.3749	0.051039	0.3189031151308490	11848
**LILRA2**	0.3749	1.3497	0.3189031151308490	11843
**NFKBIE**	0.3749	1.0148	0.3189031151308490	11845
**PAPOLA**	0.3749	1.6893	0.3189031151308490	11840
**PGAP2**	0.3749	1.5847	0.3189031151308490	11841
**UNC5CL**	0.3749	0.3466	0.3189031151308490	11847
**ZNF143**	0.3749	1.9937	0.3189031151308490	11839
**GSG1**	0.37466	1.1831	0.31953615129297700	11852
**IDH3G**	0.37466	2.5556	0.31953615129297700	11849
**MASP1**	0.37466	2.0912	0.31953615129297700	11850
**TBP**	0.37466	1.5858	0.31953615129297700	11851
**ARCN1**	0.62542	0.31689	0.31974719179280100	11853
**PRKAB2**	0.3745	0.53713	0.3199582465343820	11854
**RPL6**	0.62552	0.31157	0.3200110124463770	11855
**DUSP28**	0.37441	0.017537	0.3201957001561080	11857
**PTPRC**	0.37441	1.4669	0.3201957001561080	11856
**RNF32**	0.62565	0.49952	0.320354012600256	11858
**BICC1**	0.37419	0.76303	0.32077621838538300	11859
**TMCO1**	0.37417	1.2695	0.3208289981276970	11860
**SS18L1**	0.37411	1.5597	0.3209873427177940	11861
**HK1**	0.37401	1.0769	0.3212512682557710	11862
**ING5**	0.37397	0.79028	0.32135684473583800	11863
**GEMIN2**	0.6262	0.30208	0.3218055847556310	11864
**ZNF517**	0.37371	2.2497	0.322043179232433	11865
**GTF2H1**	0.37366	2.057	0.32217518401891100	11866
**MINPP1**	0.37359	1.2181	0.3223600001524190	11867
**WFIKKN1**	0.37354	0.3794	0.3224920184175890	11868
**LRRC58**	0.62655	0.22497	0.32272966546129500	11869

**MYD88**	0.37343	1.129	0.32278247839025500	11870
**PRSS38**	0.3734	1.9713	0.32286169947184	11871
**SCUBE2**	0.62668	0.44963	0.3230729655974270	11872
**PRPF38B**	0.62675	0.61666	0.32325783528640300	11873
**TNFAIP8**	0.6268	0.30655	0.32338989182817600	11874
**C8orf34**	0.62684	0.38734	0.3234955411220930	11875
**LIFR**	0.37311	0.65921	0.3236276078174510	11876
**RDH16**	0.62705	0.11226	0.3240502591892590	11878
**ZDHHC5**	0.62705	0.6574	0.3240502591892590	11877
**CGN**	0.62707	0.10856	0.32410309467952600	11880
**SCGB2A2**	0.37293	0.62486	0.32410309467952600	11879
**RPS17**	0.62713	0.23662	0.32426160657966400	11881
**PDXP**	0.37281	0.68309	0.32442012662755800	11882
**FAM111B**	0.6272	0.29044	0.324446547428022	11883
**RNF114**	0.37268	2.4408	0.32476361470563400	11884
**CARS**	0.37265	0.82197	0.32484288662542300	11885
**PPIC**	0.62737	0.12619	0.3248957357060010	11886
**CLDN25**	0.37238	1.6599	0.325556425837186	11887
**RPUSD2**	0.62763	0.28213	0.3255828563965880	11888
**ANKRD40**	0.62771	0.35958	0.32579430906167200	11889
**AGO3**	0.37228	0.34454	0.32582074166887600	11898
**ARMCX2**	0.37228	1.7445	0.32582074166887600	11892
**CBLC**	0.37228	0.88872	0.32582074166887600	11896
**CMTM3**	0.37228	1.7687	0.32582074166887600	11891
**GDAP1**	0.37228	1.7157	0.32582074166887600	11893
**KCNMB2**	0.37228	1.4085	0.32582074166887600	11894
**KDM5C**	0.37228	0.80316	0.32582074166887600	11897
**L3MBTL4**	0.37228	0.17904	0.32582074166887600	11899
**TLK1**	0.37228	1.2056	0.32582074166887600	11895
**TVP23C-CD**	0.37228	1.9134	0.32582074166887600	11890
**PLP2**	0.37221	1.0957	0.32600577629463400	11900
**OR5L1**	0.37216	2.1037	0.32613795071808100	11901

**TMEM41B**	0.62792	0.20483	0.3263494416483050	11902
**SLC17A2**	0.37207	1.1562	0.32637587904076800	11903
**KLF16**	0.37205	2.4521	0.32642875451006500	11904
**LYRM9**	0.37205	1.7275	0.32642875451006500	11906
**OR56A1**	0.37205	2.2125	0.32642875451006500	11905
**DLG2**	0.62803	0.35423	0.326640265515481	11907
**TAZ**	0.37175	0.010348	0.32722199616719100	11908
**MTG1**	0.37162	1.2737	0.32756579814214700	11909
**KHDRBS3**	0.37153	0.37979	0.32780383757454300	11910
**CNTN1**	0.37147	1.0317	0.32796254084823300	11911
**CIDEB**	0.62865	0.20978	0.3282799721824930	11913
**IFNG**	0.62865	0.40661	0.3282799721824930	11912
**ACTR1B**	0.37134	1.2645	0.3283064262864100	11914
**TLN2**	0.37131	1.4217	0.3283857899767860	11915
**BBS7**	0.62873	0.01354	0.3284916114482490	11916
**L2HGDH**	0.37126	1.1718	0.32851806739084100	11917
**PPP1R35**	0.37107	0.61088	0.3290207740135560	11918
**HOXC11**	0.62897	0.60989	0.3291266175783090	11919
**PSPN**	0.37102	2.1245	0.32915307904557500	11920
**HINT2**	0.62899	0.27786	0.3291795407433180	11921
**ZC3H6**	0.37094	0.36679	0.3293647790827520	11922
**KCNE5**	0.37091	0.3246	0.3294441704019110	11923
**MAGEA10**	0.37085	2.4053	0.3296029592703990	11924
**VWA5A**	0.37085	1.8684	0.3296029592703990	11925
**ACTBL2**	0.37076	0.81615	0.3298411581576970	11926
**NR1I3**	0.62929	0.4251	0.32997349895901500	11927
**ZNF595**	0.37068	2.1559	0.3300529062137370	11928
**VWF**	0.62939	0.24533	0.3302381979026700	11929
**OCA2**	0.3705	0.6879	0.3305293934684720	11930
**SELP**	0.62963	0.436	0.33087356982242600	11931
**KIAA0513**	0.62967	0.58967	0.3309794781228310	11932
**CILP**	0.6298	0.7836	0.33132370574652200	11933

**DDC**	0.62997	0.99897	0.3317739088137940	11934
**PRX**	0.36996	1.0422	0.33195930608904800	11935
**DTNA**	0.36994	1.331	0.33201227883463800	11938
**HSPA8**	0.36994	1.4382	0.33201227883463800	11937
**LOC286254**	0.36994	1.8752	0.33201227883463800	11936
**RBM23**	0.36993	0.4428	0.33203876555680000	11939
**ROR1**	0.36978	1.6751	0.33243609435488000	11940
**CTSW**	0.63024	0.65421	0.3324890754920720	11941
**CD320**	0.6303	0.80606	0.33264802450422200	11942
**RAB3GAP1**	0.36969	2.4283	0.33267451682323600	11943
**SRSF3**	0.36969	1.9086	0.33267451682323600	11945
**USP49**	0.36969	1.9671	0.33267451682323600	11944
**CCDC185**	0.63033	0.54011	0.33272750216174400	11946
**CYB5D1**	0.36955	0.69752	0.3330454338157850	11947
**TRMT2A**	0.36946	0.53918	0.33328390464864600	11948
**SUSD3**	0.36944	1.1015	0.3333369007405700	11950
**TTC12**	0.36944	1.9244	0.3333369007405700	11949
**LOC100133**	0.3694	0.88256	0.3334428957332510	11951
**RYR3**	0.3694	0.73103	0.3334428957332510	11952
**CD36**	0.36937	1.7172	0.3335223944362030	11953
**DCAF11**	0.36933	0.037385	0.33362839598590000	11954
**PRSS54**	0.36932	0.69048	0.33365489695902800	11955
**RASEF**	0.36923	1.4199	0.333893416264631	11956
**E2F8**	0.36891	0.96552	0.3347416388742910	11957
**HTR2C**	0.63121	1.3049	0.33505978442174400	11958
**PPP1R14C**	0.63121	0.79759	0.33505978442174400	11959
**GAGE2B**	0.36871	0.004536	0.33527190029374400	11960
**TPT1**	0.36863	1.3811	0.33548403125167200	11961
**COMMD8**	0.36855	2.055	0.33569617730722200	11962
**GDF15**	0.36855	1.3441	0.33569617730722200	11963
**SPDYE6**	0.36855	0.9682	0.33569617730722200	11964
**ATP5I**	0.36847	1.5508	0.3359083384720930	11966

**NCOA7**	0.36847	1.2919	0.3359083384720930	11967
**RHBDL2**	0.36847	2.2125	0.3359083384720930	11965
**TMEFF1**	0.63158	0.52299	0.3360409468780410	11968
**DNAJB4**	0.36835	2.3792	0.33622660857498200	11969
**FGL2**	0.36835	1.0302	0.33622660857498200	11973
**GEN1**	0.36835	1.3126	0.33622660857498200	11972
**MIR205HG**	0.36835	2.155	0.33622660857498200	11970
**NXPH2**	0.36835	0.065845	0.33622660857498200	11976
**PSMG2**	0.36835	0.83717	0.33622660857498200	11974
**RAD21L1**	0.36835	0.20118	0.33622660857498200	11975
**TMEM106B**	0.36835	2.1137	0.33622660857498200	11971
**CLEC17A**	0.6319	0.36902	0.3368897807351870	11977
**SERPINE1**	0.36807	0.88477	0.3369693713460990	11978
**ST7**	0.36798	0.53804	0.33720815598911800	11979
**AMER2**	0.63212	0.47471	0.33747349481172100	11980
**ZNF486**	0.63214	0.27721	0.3375265654269150	11981
**LAMTOR3**	0.63216	0.64601	0.337579636992762	11982
**SNX12**	0.36778	0.77836	0.3377388573960540	11983
**RILPL1**	0.63242	0.7579	0.338269653941707	11984
**SEMA4F**	0.63248	0.35898	0.33842891148491500	11985
**PNPLA3**	0.3674	0.18383	0.33874745232824900	11986
**MKRN2**	0.63263	0.20964	0.33882709290872800	11987
**ARID2**	0.63265	0.56233	0.33888018782295400	11988
**NME8**	0.36733	0.33864	0.33893328369251900	11989
**SLCO6A1**	0.36732	0.23435	0.33895983198561200	11990
**CDH1**	0.36721	0.43663	0.3392518789823580	11991
**ITSN2**	0.63281	0.46565	0.3393049815444570	11992
**SYT3**	0.63282	0.11972	0.33933153318430000	11993
**ADAMTS14**	0.36716	0.76179	0.33938463718168800	11994
**C19orf70**	0.36707	0.44983	0.3396236170165770	12000
**EXOSC1**	0.36707	2.3204	0.3396236170165770	11996
**KLF3**	0.36707	2.3769	0.3396236170165770	11995

**MYBPH**	0.36707	0.92189	0.3396236170165770	11999
**TMED9**	0.36707	2.0063	0.3396236170165770	11997
**ZNF417**	0.36707	1.1674	0.3396236170165770	11998
**TCF25**	0.36703	0.36855	0.33972983650200100	12001
**C6orf47**	0.36686	0.99264	0.3401813120964730	12002
**PPIH**	0.36674	1.4225	0.340500042498434	12003
**OR5H14**	0.63333	0.27619	0.34068598453970600	12004
**LGALS8**	0.36654	1.2948	0.34103133673183900	12011
**LGALSL**	0.36654	1.973	0.34103133673183900	12008
**MXRA8**	0.36654	2.1246	0.34103133673183900	12006
**RHNO1**	0.36654	2.1505	0.34103133673183900	12005
**TMTC4**	0.36654	2.0288	0.34103133673183900	12007
**VPS25**	0.36654	1.308	0.34103133673183900	12010
**ZNF239**	0.36654	1.6331	0.34103133673183900	12009
**GPR31**	0.36643	1.796	0.3413235895882460	12012
**HAX1**	0.36634	1.7557	0.3415627272464370	12013
**CCDC51**	0.36631	0.27851	0.3416424441393130	12014
**TXNDC16**	0.3662	0.28853	0.34193475799303400	12015
**RC3H1**	0.63395	0.15826	0.34233341489005600	12016
**PPAN-P2RY**	0.36602	1.3808	0.3424131527965600	12017
**CCL22**	0.63399	0.18882	0.34243973258251000	12018
**KIAA1211**	0.36598	1.0242	0.34251947339203300	12019
**ZSCAN20**	0.63406	0.31364	0.34262579785949600	12020
**AACS**	0.36592	0.22813	0.34267896154568800	12021
**CTU2**	0.63412	0.29189	0.3427852918239200	12022
**GPRASP2**	0.36584	0.99668	0.3428916259778540	12023
**CELSR3**	0.63425	0.23143	0.34313089200243100	12024
**SMS**	0.63427	0.43642	0.3431840648973820	12025
**SLC16A11**	0.3657	2.4142	0.3432638260592130	12026
**TTC30A**	0.63436	0.6306	0.34342335493503200	12027
**CXorf51B**	0.63438	0.75747	0.3434765331690320	12028
**KLHDC2**	0.63442	0.58201	0.3435828925512380	12029

**RHOXF2B**	0.63453	0.42353	0.3438754008983370	12030
**RARS**	0.36545	2.4709	0.3439285873946540	12031
**ANGPTL4**	0.36541	1.6908	0.3440349633062370	12032
**CRACR2B**	0.6346	0.1623	0.34406155789238300	12034
**RBP3**	0.6346	0.6568	0.34406155789238300	12033
**ASCL3**	0.63462	0.60164	0.34411474779473800	12035
**TPSB2**	0.63466	0.16017	0.34422113052034900	12036
**NEMP2**	0.63469	0.51781	0.3443009201210810	12037
**PYCR2**	0.36529	0.79018	0.34435411440597600	12038
**PXYLP1**	0.36504	2.2169	0.34501912524326900	12039
**WDR5**	0.36499	0.91836	0.34515214571294300	12040
**AARS2**	0.36496	1.7358	0.3452319609261790	12041
**FLT4**	0.63504	1.1454	0.3452319609261790	12042
**FRZB**	0.36492	0.80584	0.3453383846318020	12043
**KIAA1211L**	0.36484	0.8859	0.3455512437788490	12044
**PCDHB11**	0.36481	0.61006	0.3456310699950990	12045
**AICDA**	0.36473	1.6571	0.3458439506745530	12046
**UGP2**	0.63532	0.51233	0.34597700905797000	12047
**OR11H2**	0.63536	0.18865	0.34608346017501400	12048
**ARHGAP18**	0.36451	1.7738	0.34642945339874500	12050
**ELMSAN1**	0.36451	0.27989	0.34642945339874500	12053
**FAM196A**	0.36451	1.5286	0.34642945339874500	12051
**SF3A1**	0.36451	1.323	0.34642945339874500	12052
**SIPA1L1**	0.36451	2.0065	0.34642945339874500	12049
**CALR3**	0.3643	1.8055	0.34698845316743200	12054
**GMPS**	0.36427	1.931	0.34706831912508200	12057
**KRT83**	0.36427	2.3602	0.34706831912508200	12055
**MARS**	0.36427	2.2802	0.34706831912508200	12056
**POLR2M**	0.36427	1.3394	0.34706831912508200	12059
**ST6GALNAC**	0.36427	1.5017	0.34706831912508200	12058
**CSMD2**	0.63578	0.56784	0.3472014339744890	12060
**PCDHA2**	0.63582	0.049992	0.3473079302836920	12062

**TMEM250**	0.36418	1.9705	0.34730793028369200	12061
**HIST1H4D**	0.63585	0.095493	0.3473878051005580	12063
**ZMYND10**	0.36409	0.33113	0.3475475613840750	12064
**BRI3**	0.36407	0.57123	0.34760081544957600	12065
**MDGA2**	0.36405	2.1096	0.3476540705008870	12066
**C4orf19**	0.36403	1.3446	0.3477073265381960	12067
**ADAM22**	0.36401	0.11421	0.347760583561689	12068
**SKP1**	0.36398	0.2621	0.3478404709464370	12069
**KDM5A**	0.36392	1.6367	0.3480002523764810	12070
**NIPAL4**	0.63616	0.29777	0.3482133081051240	12071
**PSMC1**	0.3638	1.4707	0.3483198418965030	12072
**ZCCHC13**	0.63626	0.27819	0.34847964999662000	12073
**NAF1**	0.6363	0.46212	0.3485861936742550	12074
**GOSR1**	0.63638	0.32724	0.3487992929024260	12075
**CROT**	0.63644	0.14177	0.34895912771826700	12076
**FCRL6**	0.36354	0.70436	0.3490124079711610	12077
**FEZ1**	0.63647	0.22142	0.34903904846914200	12078
**LOC388436**	0.36351	1.8136	0.34909233020828800	12079
**TRIM5**	0.63649	0.43974	0.34909233020828800	12080
**SLIT3**	0.3634	1.4776	0.3493853974940070	12081
**ARSD**	0.36337	0.93568	0.3494653301427990	12082
**OR9K2**	0.63674	0.052919	0.3497584356299900	12083
**ART1**	0.3632	1.9055	0.34991832401442	12084
**RIMS1**	0.63682	0.48978	0.34997162213028900	12085
**PRCP**	0.36317	1.4292	0.34999827156102800	12086
**SEPT3**	0.63693	0.60723	0.3502647795435380	12087
**TSPAN6**	0.36281	1.9709	0.350957816796994	12088
**RHOH**	0.63721	0.034636	0.3510111343333420	12089
**HNRNPUL2**	0.36276	1.5305	0.351091112508904	12090
**CMA1**	0.36272	2.3861	0.3511977535699230	12092
**MMP17**	0.36272	1.3632	0.3511977535699230	12094
**RNF135**	0.36272	1.9089	0.3511977535699230	12093

**SAT2**	0.36272	2.5816	0.3511977535699230	12091
**THEM4**	0.36264	1.4343	0.3514110476756930	12095
**MYO3A**	0.63741	0.57985	0.35154436461013800	12096
**TM2D2**	0.36252	2.4449	0.35173101881637100	12097
**ATP5L**	0.36248	2.3105	0.35183768386452300	12098
**ACSM2A**	0.36223	0.23357	0.35250443117195900	12106
**ARHGAP21**	0.36223	2.3387	0.35250443117195900	12101
**ATP6AP1**	0.36223	1.5763	0.35250443117195900	12104
**CEP76**	0.36223	1.6325	0.35250443117195900	12103
**DHRS3**	0.36223	2.3327	0.35250443117195900	12102
**EML6**	0.36223	1.3366	0.35250443117195900	12105
**PA2G4**	0.36223	2.423	0.35250443117195900	12100
**TAS2R5**	0.36223	2.522	0.35250443117195900	12099
**ANKRD10**	0.63781	0.76898	0.3526111252772920	12107
**CXorf66**	0.63781	0.68728	0.3526111252772920	12108
**CYP26C1**	0.63787	0.11814	0.35277117396227800	12109
**SERPINE2**	0.36212	0.53097	0.3527978496215610	12110
**TMCO5A**	0.36206	1.9108	0.35295790885013500	12111
**POLR3E**	0.36205	0.5244	0.3529845862672640	12112
**PDRG1**	0.63807	0.36361	0.35330473487442000	12113
**FAM91A1**	0.36182	0.7361	0.3535982362447800	12114
**CNTF**	0.36179	2.0952	0.35367828735872400	12115
**DNM3**	0.63822	0.45305	0.35370497156700500	12116
**EDEM3**	0.36158	0.96683	0.3542387086541240	12117
**MED20**	0.63849	0.0059792	0.3544255404702520	12118
**SYT13**	0.36144	1.1094	0.3546123846588060	12119
**FANCF**	0.36117	2.0817	0.35533318543223300	12120
**TOP 1.00**	0.36116	2.4567	0.35535988530060000	12123
**ARPP21**	0.36116	1.7995	0.35535988530060000	12121
**VASP**	0.36116	2.4092	0.35535988530060000	12122
**REG3A**	0.63889	0.5222	0.35549338844289000	12124
**LNPK**	0.3611	1.1274	0.35552008983160700	12125

**FAM109A**	0.63893	0.17386	0.35560019551870400	12126
**TMOD3**	0.63901	0.62092	0.3558138218421040	12127
**NUP210**	0.36096	0.8484	0.3558939358993940	12128
**TGFBR2**	0.36073	1.7308	0.35650821960852300	12129
**MTMR4**	0.63931	0.41473	0.35661506528513400	12130
**ADAMTSL4**	0.63937	0.3236	0.356775341434155	12131
**OLFML2A**	0.63943	0.3219	0.356935626748697	12132
**KPTN**	0.6395	0.1945	0.35712263787342100	12133
**SNAPC3**	0.36049	0.81834	0.35714935476782600	12134
**MTFR1L**	0.36047	2.4179	0.3572027893214570	12135
**PRPF40A**	0.36047	1.1307	0.3572027893214570	12140
**RTP1**	0.36047	0.87375	0.3572027893214570	12141
**SMARCD2**	0.36047	1.1588	0.3572027893214570	12139
**TMPRSS11E**	0.36047	1.5532	0.3572027893214570	12137
**TRNAU1AP**	0.36047	1.4861	0.3572027893214570	12138
**USP37**	0.36047	1.5696	0.3572027893214570	12136
**YBEY**	0.63954	0.79877	0.35722950698073100	12142
**NKX2-5**	0.36022	2.0585	0.3578708073597720	12143
**CCT2**	0.35985	1.9869	0.35885976731638000	12145
**FIGNL1**	0.35985	2.5384	0.35885976731638000	12144
**RIN1**	0.35985	1.3812	0.35885976731638000	12146
**ABHD17A**	0.35984	0.2323	0.35888650083126500	12148
**ZBTB10**	0.64016	0.39131	0.3588865008312650	12147
**WDR93**	0.64024	0.57107	0.3591003781860640	12149
**CD2BP2**	0.35973	0.95724	0.35918058642873800	12150
**LDLRAD1**	0.3597	1.0741	0.35926079698221600	12151
**SERTAD4**	0.35964	1.4847	0.35942122502418200	12152
**NAA50**	0.35957	1.1246	0.35960840276575800	12153
**PKP1**	0.64049	0.31319	0.3597688508581500	12154
**CDH24**	0.35931	1.7733	0.3603037447562110	12155
**LHX2**	0.35928	2.581	0.36038398772917700	12156
**TBC1D13**	0.6409	0.65346	0.3608654943214310	12157

**EPCAM**	0.641	0.48883	0.36113303413310900	12158
**TRIM52**	0.6412	0.066652	0.3616681913351640	12159
**PRKAA1**	0.35877	1.5358	0.36174847384619200	12160
**LONRF1**	0.3587	0.32363	0.3619358087738700	12161
**C6orf120**	0.35868	2.0075	0.36198933537172000	12164
**CCL2**	0.35868	2.4494	0.36198933537172000	12162
**CHID1**	0.35868	1.0771	0.36198933537172000	12168
**DCT**	0.35868	2.3424	0.36198933537172000	12163
**HIST1H4C**	0.35868	1.5857	0.36198933537172000	12166
**MID1**	0.35868	1.5604	0.36198933537172000	12167
**NEUROD2**	0.35868	1.6003	0.36198933537172000	12165
**RNF17**	0.35868	0.22785	0.36198933537172000	12169
**WDR76**	0.35867	0.073461	0.3620160990595660	12170
**OR1L1**	0.64133	0.57998	0.3620160990595670	12171
**THRB**	0.35864	0.99019	0.3620963916790780	12172
**F11**	0.35852	0.67277	0.3624175855083530	12174
**KIR2DL3**	0.35852	1.5761	0.3624175855083530	12173
**TBC1D10C**	0.35839	2.2789	0.3627655876887270	12175
**PREPL**	0.64165	0.30899	0.36287267411990900	12176
**SLC2A11**	0.35834	1.4686	0.3628994463778640	12177
**BATF2**	0.3582	2.0846	0.36327428531203600	12178
**RNASEH2B**	0.3582	0.54893	0.36327428531203600	12179
**LCE3B**	0.64183	0.42891	0.3633546145800380	12180
**MTA1**	0.64184	0.24067	0.36338139152372200	12182
**TNNC1**	0.64184	0.79695	0.36338139152372200	12181
**B3GNT6**	0.64185	0.35473	0.3634081687279540	12183
**SDHA**	0.35815	0.57277	0.36340816872795500	12184
**ST6GALNAC**	0.35809	1.9149	0.36356883742623500	12185
**AGMO**	0.64194	0.0095575	0.3636491752947500	12186
**SRBD1**	0.35797	2.1795	0.36389020298566300	12187
**GPATCH8**	0.35793	1.6587	0.36399733318906000	12188
**FEZF2**	0.64213	0.12707	0.3641580363278620	12189

**ISM2**	0.35781	1.0399	0.36431874887178300	12190
**CSTB**	0.35778	0.42514	0.36439910867230400	12191
**IL13RA2**	0.64243	0.37702	0.36496169320372200	12192
**MCUR1**	0.64248	0.55483	0.36509565892215400	12193
**POLE**	0.35748	1.0739	0.36520283621464500	12194
**ORAI2**	0.64256	0.47443	0.36531001770237800	12195
**TDRD6**	0.64266	0.23025	0.36557798978613100	12196
**CTAG2**	0.64278	0.73107	0.36589959094458000	12197
**STK4**	0.35718	1.3959	0.36600679973942300	12198
**KCNK5**	0.35712	1.0722	0.366167620820207	12201
**LRRK2**	0.35712	1.9193	0.366167620820207	12200
**RRBP1**	0.35712	2.0286	0.366167620820207	12199
**PTP4A3**	0.35695	0.74276	0.3666233319989200	12202
**PYGO1**	0.35684	0.54256	0.3669182445089920	12203
**CIDEC**	0.35681	0.29108	0.3669986807312590	12204
**PEX1**	0.35664	0.40887	0.36745453086277200	12205
**OR5D14**	0.64339	0.6462	0.3675349829278470	12206
**TMEM52**	0.64342	0.85221	0.36761543737187700	12207
**ATAD3C**	0.35653	1.9501	0.367749533400292	12211
**FAR2**	0.35653	1.7515	0.367749533400292	12212
**HSD17B1**	0.35653	2.3222	0.367749533400292	12208
**KAT2B**	0.35653	0.93823	0.367749533400292	12219
**KLHDC3**	0.35653	1.0333	0.367749533400292	12218
**LRRC70**	0.35653	2.2213	0.367749533400292	12209
**METTL22**	0.35653	0.10475	0.367749533400292	12220
**MSTO1**	0.35653	1.7135	0.367749533400292	12214
**MTR**	0.35653	1.3618	0.367749533400292	12216
**PDE5A**	0.35653	1.6447	0.367749533400292	12215
**TAAR9**	0.35653	1.2377	0.367749533400292	12217
**TAS2R10**	0.35653	1.7488	0.367749533400292	12213
**ZNF696**	0.35653	2.1596	0.367749533400292	12210
**S100PBP**	0.35652	1.0008	0.36777635339945000	12221

**PABPC5**	0.35649	1.3429	0.3678568149843170	12222
**AQP3**	0.64363	0.23285	0.3681786851464300	12223
**TACC1**	0.35632	1.3567	0.36831280897050900	12224
**SVEP1**	0.35631	0.27601	0.36833963453034500	12225
**SERTAD3**	0.35623	1.0887	0.3685542485534010	12227
**VPS39**	0.35623	1.7046	0.3685542485534010	12226
**CX3CL1**	0.6438	0.4306	0.3686347331881700	12228
**FAM102A**	0.64385	0.61877	0.36876887955312600	12229
**PLBD1**	0.35592	1.9725	0.3693860383565400	12230
**DYNLRB2**	0.35591	2.7726	0.36941287453809800	12231
**GPR89A**	0.35591	1.4594	0.36941287453809800	12234
**PROSER3**	0.35591	1.8984	0.36941287453809800	12232
**SCNM1**	0.35591	1.8685	0.36941287453809800	12233
**SPACA3**	0.64409	0.548	0.36941287453809800	12235
**KCNK9**	0.64414	0.40434	0.3695470594370800	12236
**SYT10**	0.35576	1.1757	0.36981544920087300	12237
**OR2T35**	0.64426	0.5517	0.36986913034983800	12238
**ARFGAP3**	0.64431	0.59091	0.3700033378858270	12239
**TMEM133**	0.35567	2.1953	0.3700570227661690	12240
**SHQ1**	0.64444	0.40867	0.37035230867795400	12241
**TRIM64B**	0.35549	0.19478	0.37054023470774100	12242
**HIKESHI**	0.3554	0.34717	0.3707818731199590	12243
**TBC1D3I**	0.35533	0.90051	0.3709698290753260	12244
**CCR4**	0.35529	0.021839	0.3710772383623330	12247
**OR8A1**	0.35529	0.13359	0.3710772383623330	12246
**PAQR5**	0.35529	0.2773	0.3710772383623330	12245
**OBSL1**	0.35525	0.2859	0.37118465193054300	12248
**DYTN**	0.64483	0.26315	0.3713994919169020	12249
**PTRH2**	0.3551	0.83763	0.37158749096779900	12250
**EQTN**	0.64501	0.48105	0.3718829445874790	12251
**SGCB**	0.35491	1.833	0.37209784033744700	12252
**SULT1A4**	0.3549	0.37217	0.3721247035142500	12253

**MRPL51**	0.3548	0.30724	0.3723933500560410	12254
**LRTM1**	0.35466	1.7135	0.37276950037635400	12259
**LYZL4**	0.35466	2.6552	0.37276950037635400	12256
**PIWIL3**	0.35466	2.3818	0.37276950037635400	12258
**SLC25A45**	0.35466	2.8116	0.37276950037635400	12255
**TGIF2LX**	0.35466	2.6408	0.37276950037635400	12257
**DCAF7**	0.35461	0.21606	0.3729038525568060	12260
**RNF122**	0.64549	0.62929	0.3731725771151700	12261
**HSF4**	0.35446	1.9537	0.37330694949928500	12262
**APOBEC3A**	0.3544	1.7843	0.373468205258296	12264
**ERC2**	0.3544	2.0727	0.373468205258296	12263
**KLHL10**	0.3544	0.43752	0.373468205258296	12266
**ZNF600**	0.3544	0.83819	0.373468205258296	12265
**C5orf66**	0.3542	0.78716	0.3740057946277470	12267
**FCER1A**	0.6458	0.25848	0.3740057946277470	12268
**PRPH2**	0.64583	0.2247	0.3740864423528350	12270
**UBE2J2**	0.35417	2.0131	0.3740864423528350	12269
**OR52W1**	0.35407	1.2408	0.3743552856797010	12271
**FNIP1**	0.64596	0.48185	0.37443594395332000	12272
**RBM27**	0.646	0.50005	0.37454349210770100	12273
**FAM25C**	0.35397	1.0143	0.3746241560665230	12274
**ARHGEF26**	0.64607	0.27652	0.37473171180361400	12275
**ARL 10.00**	0.64617	0.028115	0.37500062011619200	12276
**GSN**	0.64621	0.72651	0.3751081910333490	12277
**TBC1D22A**	0.35376	1.0054	0.3751888720697460	12278
**SULT1B1**	0.64631	0.053976	0.3753771373205090	12279
**HSD3B7**	0.35362	1.6965	0.3755654158770360	12280
**CATSPER2**	0.35356	2.3036	0.3757268080937030	12281
**CCDC107**	0.35345	0.97507	0.3760227192480440	12282
**C22orf23**	0.64657	0.30336	0.37607652481302000	12284
**ST8SIA6**	0.35343	0.931	0.37607652481302000	12283
**RAX2**	0.35338	1.6663	0.3762110434893120	12285

**OR4X2**	0.64663	0.1307	0.37623794804143000	12286
**ATP6V1F**	0.35328	2.7422	0.3764801012690240	12288
**NPNT**	0.35328	2.484	0.3764801012690240	12290
**PCDHA3**	0.35328	2.7792	0.3764801012690240	12287
**PNKP**	0.35328	0.4343	0.3764801012690240	12291
**RHD**	0.35328	2.5314	0.3764801012690240	12289
**MYL6B**	0.64686	0.48728	0.37685682795799300	12292
**TCF23**	0.64687	0.52301	0.37688373905329300	12293
**WDR88**	0.35307	2.5854	0.37704521135782400	12294
**GAMT**	0.35297	0.39253	0.3773143537156850	12295
**DNAJC30**	0.35288	1.0373	0.3775566052081200	12296
**ARMC3**	0.35272	1.8519	0.3779873292539960	12297
**PTPRN2**	0.64744	0.83114	0.37841812343688900	12299
**SNAP25**	0.35256	0.9772	0.37841812343688900	12298
**FARS2**	0.35245	1.316	0.3787143351760060	12300
**ZNF500**	0.3524	1.4936	0.3788489878596230	12301
**CCKBR**	0.64765	0.35566	0.3789836474126500	12302
**AFF4**	0.3523	1.8557	0.3791183138382320	12310
**BTC**	0.3523	1.4444	0.3791183138382320	12311
**CLN6**	0.3523	2.4117	0.3791183138382320	12303
**CYP27A1**	0.3523	2.2002	0.3791183138382320	12306
**GPR55**	0.3523	2.1813	0.3791183138382320	12307
**IFNGR2**	0.3523	1.3559	0.3791183138382320	12312
**IRF2BPL**	0.3523	0.70291	0.3791183138382320	12318
**LOC100287**	0.3523	1.0273	0.3791183138382320	12317
**NEBL**	0.3523	1.1555	0.3791183138382320	12314
**NUP98**	0.3523	1.2843	0.3791183138382320	12313
**PLD4**	0.3523	1.9284	0.3791183138382320	12309
**PTK6**	0.3523	1.1077	0.3791183138382320	12315
**RUFY4**	0.3523	1.0435	0.3791183138382320	12316
**SLC25A5**	0.3523	1.9356	0.3791183138382320	12308
**TNFAIP3**	0.3523	2.2527	0.3791183138382320	12304

**ZNF860**	0.3523	2.24	0.3791183138382320	12305
**ISLR2**	0.64779	0.50008	0.3793607307331480	12319
**TMEM248**	0.64786	0.54341	0.3795492926202770	12320
**PRICKLE3**	0.64799	0.25038	0.37989951479461500	12321
**EFCAB8**	0.35196	1.4437	0.3800342280369900	12322
**RPL4**	0.64814	0.20187	0.380303675216201	12323
**ACP1**	0.35186	0.30682	0.38030367521620200	12324
**AIF1**	0.35184	1.5213	0.3803575679648720	12329
**GP9**	0.35184	2.4743	0.3803575679648720	12327
**GPLD1**	0.35184	2.2598	0.3803575679648720	12328
**NCKAP5**	0.35184	2.8635	0.3803575679648720	12325
**TACSTD2**	0.35184	2.777	0.3803575679648720	12326
**TSPYL2**	0.64819	0.84512	0.38043840915934200	12330
**HORMAD2**	0.64831	0.64808	0.38076179880765800	12331
**YBX2**	0.35164	1.6183	0.38089655624598100	12332
**CLASP2**	0.64839	0.1838	0.38097741402907300	12333
**GJA8**	0.35145	0.72054	0.38140869762419300	12334
**PCDHA8**	0.64859	0.86357	0.38151652960580400	12335
**PCDHGA4**	0.64864	0.44913	0.38165132582165100	12336
**SLAMF8**	0.35133	1.078	0.3817322068797520	12337
**TLR3**	0.35123	0.41147	0.3820018284465010	12338
**FXR1**	0.3512	0.16815	0.38208272033108300	12339
**RAB11FIP3**	0.64883	0.6299	0.382163614715907	12340
**COPG2**	0.35113	1.5546	0.3822714777861200	12341
**PLEKHD1**	0.64889	0.56656	0.382325410989017	12342
**EMX2**	0.3511	1.6955	0.3823523780075010	12343
**ASIP**	0.64891	0.52606	0.3823793453040450	12344
**HLA-G**	0.64893	0.10977	0.3824332807314060	12346
**RAB11FIP2**	0.64893	0.18226	0.3824332807314060	12345
**GRIK4**	0.64897	0.71444	0.3825411549239410	12347
**VSTM5**	0.64907	0.068549	0.38281085988531400	12348
**ING4**	0.35088	0.46501	0.3829457228077590	12349

**SERBP1**	0.64945	0.29264	0.3838359929367700	12350
**MRPL21**	0.35051	0.84323	0.3839439251290090	12351
**OR8B3**	0.3505	0.52719	0.38397090887589600	12352
**ZC3HAV1L**	0.6495	0.49345	0.3839709088758960	12353
**OR51V1**	0.35041	2.0465	0.3842137751821060	12354
**SREBF2**	0.35035	0.98666	0.3843756986432510	12355
**PLP1**	0.35032	0.71683	0.3844566641529870	12356
**NELL1**	0.64977	0.015298	0.38469957580710300	12357
**MGAT1**	0.6498	0.2139	0.38478055140240400	12358
**C7orf31**	0.35016	2.2024	0.384888522787764	12361
**EHF**	0.35016	0.6363	0.384888522787764	12370
**FAM8A1**	0.35016	1.8242	0.384888522787764	12363
**GABPB1**	0.35016	0.92827	0.384888522787764	12369
**IDH3B**	0.35016	1.4046	0.384888522787764	12367
**IFNA16**	0.35016	1.0461	0.384888522787764	12368
**MEGF6**	0.35016	0.29247	0.384888522787764	12371
**OR2G6**	0.35016	1.8833	0.384888522787764	12362
**RGPD8**	0.35016	1.582	0.384888522787764	12365
**SH3RF3**	0.35016	1.7824	0.384888522787764	12364
**UBE2B**	0.35016	1.5488	0.384888522787764	12366
**VILL**	0.35016	2.442	0.384888522787764	12359
**WDR87**	0.35016	2.3994	0.384888522787764	12360
**OR56B4**	0.64985	0.010314	0.3849155163351590	12372
**CAND1**	0.35009	1.8442	0.3850774835103600	12373
**TSGA10IP**	0.64992	0.47032	0.38510447902160400	12374
**RPLP0**	0.35007	0.9139	0.3851314748134980	12375
**PGLYRP3**	0.34996	0.17007	0.38542844705464900	12376
**GNAI3**	0.34994	0.96412	0.38548244565904400	12377
**CLDN11**	0.34991	0.78749	0.38556344567326600	12378
**SMC4**	0.34986	1.2062	0.38569845131902300	12379
**CXCL9**	0.34977	1.5173	0.385941479199969	12380
**UVSSA**	0.34965	2.111	0.3862655518396390	12381

**KATNBL1**	0.65057	0.21303	0.3868597904188180	12382
**MARS2**	0.34942	2.5471	0.38688680450786700	12384
**SPON1**	0.34942	2.6909	0.38688680450786700	12383
**KLHDC8A**	0.34939	0.31255	0.38696784846915000	12385
**GGA2**	0.34922	1.1499	0.38742714561409500	12386
**DALRD3**	0.34915	0.61171	0.38761629172024500	12387
**ANKRD20A1**	0.34892	2.0102	0.388237869476279	12388
**CD5L**	0.34888	0.82486	0.38834598526237800	12389
**AKR1B10**	0.34885	1.0524	0.3884270750809700	12390
**GRM6**	0.34881	1.8884	0.3885351988124370	12391
**EYA2**	0.65125	0.13244	0.3886973929272860	12392
**FGFR1OP2**	0.34868	0.35399	0.38888663231967900	12393
**ZNF32**	0.65137	0.73969	0.3890218118409010	12394
**SUCLG2**	0.34855	1.296	0.38923811386331400	12395
**GEMIN4**	0.34839	1.8918	0.3896707725594870	12396
**DCP2**	0.65162	0.24759	0.3896978161492840	12397
**DPYSL5**	0.34833	2.2431	0.38983303837396100	12398
**C22orf24**	0.34826	2.4555	0.39002236146514600	12399
**KIF4B**	0.34816	2.5574	0.39029284727849700	12400
**MYL12B**	0.34816	2.2896	0.39029284727849700	12401
**CCR10**	0.34814	1.931	0.3903469478672950	12403
**CDH3**	0.34814	2.2759	0.3903469478672950	12402
**ERF**	0.34814	0.12241	0.3903469478672950	12410
**LNPEP**	0.34814	0.44561	0.3903469478672950	12409
**PCDHB5**	0.34814	1.6337	0.3903469478672950	12405
**POTEA**	0.34814	1.5838	0.3903469478672950	12408
**PRAMEF33**	0.34814	1.6002	0.3903469478672950	12406
**PRSS53**	0.34814	1.5912	0.3903469478672950	12407
**PTPRT**	0.34814	1.8047	0.3903469478672950	12404
**DSG1**	0.34798	1.4831	0.39077979372594100	12411
**SAE1**	0.34798	0.30582	0.39077979372594100	12412
**NRN1L**	0.65203	0.19348	0.3908068490223880	12413

**TCAF1**	0.34784	1.9034	0.39115859391802300	12414
**MRPS33**	0.34779	0.77752	0.3912938933015440	12415
**ZBTB40**	0.65229	0.10116	0.39151038721640500	12416
**CPPED1**	0.34751	0.85522	0.3920517023207010	12417
**ANKRD26**	0.34745	1.394	0.392214119248959	12418
**METTL13**	0.65265	0.3099	0.3924848371255340	12419
**GPC2**	0.65279	0.15852	0.39286389048824600	12420
**EPS15L1**	0.65287	0.12329	0.3930805177527700	12421
**DEXI**	0.34709	0.54627	0.3931888383021570	12422
**C14orf177**	0.34699	2.01	0.39345965986391900	12424
**DNMT1**	0.34699	2.3552	0.39345965986391900	12423
**KDM4D**	0.34699	0.66919	0.39345965986391900	12425
**ZFR2**	0.3469	0.78065	0.39370342394511600	12426
**TRUB1**	0.65314	0.48796	0.3938117710456400	12427
**ABCB10**	0.65316	0.66645	0.39386594632950200	12428
**TRERF1**	0.34678	0.65216	0.3940284791179880	12429
**MYBPHL**	0.34671	0.10103	0.3942181138616870	12430
**CCDC183**	0.65331	0.017754	0.39427229782071200	12431
**EIF2AK1**	0.34659	1.1688	0.3945432349836240	12432
**AGTR1**	0.34647	0.91582	0.3948683978157930	12433
**ZNF568**	0.65358	0.14994	0.395003894650228	12434
**CD207**	0.65366	0.19639	0.39522070467178200	12435
**ABCB11**	0.34629	2.477	0.3953562203693830	12437
**ACAA2**	0.34629	1.4003	0.3953562203693830	12443
**AOAH**	0.34629	1.4168	0.3953562203693830	12442
**CARMIL3**	0.34629	1.7806	0.3953562203693830	12439
**DCAF12L2**	0.34629	1.74	0.3953562203693830	12440
**NCAPH**	0.34629	1.6737	0.3953562203693830	12441
**OR11H4**	0.34629	2.5608	0.3953562203693830	12436
**WDR35**	0.34629	2.4709	0.3953562203693830	12438
**HIST3H3**	0.34624	0.51003	0.39549174332792600	12444
**GOLGA7**	0.34615	0.97829	0.39573570296131100	12445

**SPINK14**	0.65392	0.03774	0.3959254656268680	12446
**ERAP1**	0.34604	2.1771	0.39603390783689500	12447
**UBE2G2**	0.34602	0.4882	0.3960881306883530	12448
**RNF144B**	0.65401	0.51168	0.3961694671491870	12449
**C19orf66**	0.65403	0.085405	0.396223692912478	12450
**SLC9A3**	0.34595	2.0941	0.39627791984086900	12451
**IGF2**	0.65407	0.092759	0.3963321479345700	12452
**PDZD2**	0.6541	0.3255	0.39641349226053300	12453
**ITGA10**	0.65423	0.0057333	0.3967660146604590	12454
**MYO9A**	0.34569	0.7765	0.396982976029405	12455
**TMEM253**	0.65438	0.41227	0.3971728325572440	12456
**PRDM6**	0.65484	0.63121	0.39842081776790400	12457
**CEBPD**	0.34515	2.3721	0.3984479547668520	12459
**NCF4**	0.34515	2.1375	0.3984479547668520	12460
**PIK3CB**	0.34515	1.7706	0.3984479547668520	12461
**RHBDL3**	0.34515	0.47259	0.3984479547668520	12462
**ZSCAN2**	0.34515	2.8742	0.3984479547668520	12458
**UBE2E1**	0.34512	0.029572	0.39852936752437000	12463
**C14orf105**	0.34498	2.3457	0.3989093286649840	12465
**HIST1H2BG**	0.34498	1.7179	0.3989093286649840	12467
**RTP3**	0.34498	1.6572	0.3989093286649840	12468
**TNFAIP8L2-**	0.34498	2.7246	0.3989093286649840	12464
**VCPKMT**	0.34498	2.1231	0.3989093286649840	12466
**CLMN**	0.34484	0.22373	0.3992893474053360	12472
**DYNC1I2**	0.34484	1.7354	0.3992893474053360	12469
**FIP1L1**	0.34484	0.93126	0.3992893474053360	12471
**STARD6**	0.34484	1.5237	0.3992893474053360	12470
**C4orf33**	0.65524	0.11153	0.3995065268602030	12473
**NRP1**	0.65532	0.55583	0.39972372516028800	12474
**CFC1**	0.34463	1.346	0.3998594836736850	12475
**MFAP4**	0.65541	0.21444	0.39996809579064100	12476
**ANXA2**	0.65545	0.27345	0.4000767126260860	12477

**PRM2**	0.65549	0.44886	0.4001853341817110	12478
**GRHL2**	0.34439	1.1952	0.4005112271865900	12479
**MAGIX**	0.34436	1.9902	0.40059270708329800	12480
**MST1**	0.34432	1.0712	0.40070135108289300	12481
**NINJ1**	0.34423	0.70356	0.400945817378316	12482
**LEFTY2**	0.34415	0.63342	0.4011631408697730	12483
**FOXF2**	0.65587	0.27711	0.40121747470280700	12484
**CCNF**	0.65591	0.011085	0.4013261459225650	12485
**ZNF689**	0.65593	0.16173	0.40138048330971200	12486
**RCHY1**	0.34396	1.1282	0.4016793601288800	12487
**SNTA1**	0.34377	2.3538	0.4021956864511850	12488
**APOA4**	0.65626	0.077941	0.4022772214588840	12489
**TNFRSF17**	0.65628	0.10128	0.4023315796164290	12490
**ARL4D**	0.34348	1.9488	0.4029839702977300	12492
**COL1A2**	0.65652	0.41083	0.4029839702977300	12494
**PIGH**	0.34348	2.4618	0.4029839702977300	12491
**SIRT2**	0.34348	1.0762	0.4029839702977300	12493
**DOCK4**	0.65653	0.0076088	0.40301115696335500	12495
**AMY1C**	0.34328	2.2868	0.4035277602370930	12496
**COL9A3**	0.65672	0.17726	0.40352776023709300	12504
**CRKL**	0.34328	1.4675	0.4035277602370930	12501
**KRTAP12-1**	0.34328	2.2033	0.4035277602370930	12497
**LIN28A**	0.34328	1.7779	0.4035277602370930	12500
**MPST**	0.34328	1.4603	0.4035277602370930	12502
**PLEKHA7**	0.34328	1.7929	0.4035277602370930	12499
**RPS10**	0.34328	1.8822	0.4035277602370930	12498
**TALDO1**	0.34328	1.3205	0.4035277602370930	12503
**NNAT**	0.65687	0.30914	0.4039356810053600	12506
**RPL27A**	0.34313	2.2799	0.40393568100536000	12505
**ZIC3**	0.34307	2.2278	0.4040988681309230	12507
**MOB1A**	0.34298	0.16222	0.4043436689996960	12508
**MACF1**	0.65705	0.39384	0.4044252746736180	12509

**CHST1**	0.65707	0.15126	0.40447967995250400	12510
**SLC2A3**	0.65714	0.43339	0.404670107858477	12511
**PRTG**	0.65725	0.51842	0.40496938136510300	12512
**ZRSR2**	0.34267	1.7079	0.40518705760706100	12513
**HES6**	0.3426	1.9385	0.4053775400689240	12514
**ACOT2**	0.65767	0.65194	0.4061123960094920	12515
**SH3GLB1**	0.34231	1.6281	0.4061668385031480	12516
**KRT25**	0.34229	2.2722	0.40622128220070900	12517
**GIN1**	0.34205	1.5303	0.4068747005542390	12518
**NANS**	0.34203	1.1379	0.40692915992211600	12519
**CACNA1S**	0.34185	2.5952	0.4074193485699070	12520
**FTHL17**	0.34185	2.3513	0.4074193485699070	12521
**GPAM**	0.65815	0.026201	0.4074193485699070	12523
**SLC45A3**	0.34185	1.8784	0.4074193485699070	12522
**NDST3**	0.34181	0.26174	0.4075282926723470	12524
**FAH**	0.65822	0.35011	0.4076100039234440	12525
**MEF2B**	0.34173	0.48226	0.4077461953903140	12526
**TSSK4**	0.34168	2.0963	0.40788239442056900	12527
**TACC2**	0.6584	0.080551	0.40810032860922300	12529
**USP12**	0.3416	1.901	0.40810032860922300	12528
**CRISPLD1**	0.65853	0.056989	0.40845451301581600	12530
**DUSP22**	0.34145	1.0094	0.40850900747158400	12531
**ISY1-RAB43**	0.65858	0.43979	0.40859075142997800	12532
**CALM3**	0.34116	1.2411	0.4092993135183610	12538
**CENPQ**	0.34116	0.93722	0.4092993135183610	12541
**DARS2**	0.34116	2.316	0.4092993135183610	12535
**FAF2**	0.34116	1.1859	0.4092993135183610	12540
**FGF11**	0.34116	0.48424	0.4092993135183610	12542
**SLC35E2**	0.34116	1.9602	0.4092993135183610	12537
**SYNPO2L**	0.34116	2.5362	0.4092993135183610	12534
**VCX3A**	0.34116	2.5815	0.4092993135183610	12533
**WFDC1**	0.34116	1.2124	0.4092993135183610	12539

**ZNF69**	0.34116	2.2361	0.4092993135183610	12536
**CACNA1E**	0.34115	1.6438	0.40932657000832700	12543
**PSMA8**	0.34115	0.8822	0.40932657000832700	12544
**ADGRV1**	0.34079	1.9054	0.4103080063882200	12545
**GALNS**	0.34079	1.4175	0.4103080063882200	12546
**LAMA1**	0.65925	0.024881	0.4104170792577590	12547
**RAB36**	0.34073	0.052443	0.4104716175235190	12548
**PARP2**	0.34072	2.5026	0.41049888711424200	12549
**NR0B1**	0.6593	0.45671	0.41055342721151000	12550
**CPM**	0.34064	1.5029	0.4107170548318350	12551
**IPO13**	0.65937	0.73456	0.41074432717091700	12552
**FAM193B**	0.65952	0.32867	0.4111534489333970	12553
**BRCC3**	0.34046	2.2104	0.41120800370029800	12555
**ELOB**	0.34046	2.585	0.41120800370029800	12554
**FAM208A**	0.34046	1.5352	0.41120800370029800	12558
**KLK9**	0.34046	1.4915	0.41120800370029800	12559
**SHROOM4**	0.34046	2.1254	0.41120800370029800	12556
**SPIC**	0.34046	1.9192	0.41120800370029800	12557
**UCMA**	0.65958	0.55589	0.41131711690596300	12560
**CMKLR1**	0.3404	2.0368	0.41137167534516000	12561
**EDDM3A**	0.34036	2.3531	0.4114807958973710	12562
**DCAF4L1**	0.34027	1.8755	0.41172633505765100	12563
**LRRC28**	0.34027	1.2518	0.41172633505765100	12564
**ANKRD13B**	0.65981	0.038616	0.4119446129294900	12565
**GOT1L1**	0.34003	1.4256	0.41238122757433000	12566
**TMIGD2**	0.34	0.1358	0.41246310157036100	12567
**TMEM241**	0.33997	2.0306	0.4125449783313880	12568
**TBCC**	0.66011	0.29441	0.4127633298841380	12569
**C11orf80**	0.66028	0.53722	0.4132273922994290	12571
**CSNK2B**	0.33972	2.2029	0.4132273922994290	12570
**ODF3**	0.33962	1.7095	0.4135004117601880	12572
**GPRIN1**	0.66049	0.72401	0.41380076877190900	12573

**SSC5D**	0.66054	0.32447	0.41393730702649600	12574
**SLC6A15**	0.33934	2.1243	0.4142650303309630	12575
**FRY**	0.33931	0.76586	0.41434696810826900	12576
**C20orf24**	0.33919	2.5646	0.4146747470444220	12577
**CEP250**	0.33919	1.8735	0.4146747470444220	12580
**EFEMP1**	0.33919	2.0138	0.4146747470444220	12579
**PMEL**	0.33919	2.07	0.4146747470444220	12578
**POLR3C**	0.33917	1.1316	0.41472938119792000	12581
**GSTT2**	0.66094	0.51447	0.41502989117652500	12582
**MDC 1.00**	0.33906	1.8786	0.41502989117652600	12589
**ARHGEF15**	0.33906	1.8786	0.41502989117652600	12593
**C9orf92**	0.33906	1.4326	0.41502989117652600	12584
**CSNK1A1L**	0.33906	2.2801	0.41502989117652600	12594
**EFCAB11**	0.33906	0.91534	0.41502989117652600	12588
**EFCAB3**	0.33906	1.8822	0.41502989117652600	12590
**ORM1**	0.33906	2.0644	0.41502989117652600	12586
**PLGLB1**	0.33906	2.0944	0.41502989117652600	12585
**SPOUT1**	0.33906	1.8021	0.41502989117652600	12591
**TMEM132B**	0.33906	2.6971	0.41502989117652600	12583
**TMF1**	0.33906	1.5435	0.41502989117652600	12592
**WIPI1**	0.33906	1.9651	0.41502989117652600	12587
**ZNF428**	0.66096	0.63633	0.4150845333806660	12595
**OR4C12**	0.33899	2.3211	0.4152211443137960	12596
**PIDD1**	0.33892	1.2354	0.41541241264022400	12597
**SLX4IP**	0.33889	2.4856	0.4154943894321970	12598
**TMEM256**	0.66124	0.26131	0.4158496544736840	12599
**IFIT1**	0.33872	0.30895	0.41595897735222500	12600
**CTSK**	0.33868	0.43803	0.41606830520235200	12601
**PRAM1**	0.33849	1.1042	0.4165876804377550	12602
**SPANXN1**	0.66151	0.12397	0.41658768043775500	12603
**SGK3**	0.33848	0.65564	0.4166150190883760	12604
**GPX1**	0.33841	1.3249	0.4168063983630320	12606

**TEAD3**	0.33841	2.5879	0.4168063983630320	12605
**TRIM42**	0.66165	0.22225	0.4169704498924540	12607
**OR13C8**	0.33828	0.42947	0.4171618575281400	12608
**MAP9**	0.66176	0.033861	0.4172712401819290	12609
**EIF2S1**	0.66177	0.31857	0.41729858662542100	12610
**ZNF219**	0.33818	1.8826	0.4174353235245280	12611
**SRSF5**	0.66186	0.034535	0.4175447186634450	12612
**MLF 2.00**	0.33808	1.0781	0.4177088207420110	12613
**AQP2**	0.66198	0.045202	0.4178729340701050	12614
**COL22A1**	0.66205	0.67102	0.41806441384565700	12615
**ALAD**	0.33794	1.3287	0.41809176935047000	12619
**KRTAP4-1**	0.33794	2.2119	0.41809176935047000	12616
**RABL6**	0.33794	1.7529	0.41809176935047000	12617
**STAMBPL1**	0.33794	0.53469	0.41809176935047000	12620
**ZFAND2A**	0.33794	1.4515	0.41809176935047000	12618
**CMTM7**	0.33788	0.90074	0.4182559089506570	12621
**KRTAP12-2**	0.33787	1.0106	0.41828326664616400	12622
**PIGT**	0.33779	0.78453	0.4185021394829560	12623
**CTAG1B**	0.33773	1.2366	0.4186663072678770	12624
**BTD**	0.33764	1.2696	0.41891258010527800	12625
**SLC34A2**	0.33753	2.2194	0.41921361474926400	12626
**GOLGA8A**	0.66261	0.43137	0.41959680470692700	12627
**FAM98B**	0.66264	0.28005	0.41967892485521500	12628
**IGF1**	0.66266	0.79481	0.4197336731930900	12629
**PRRC2B**	0.33732	1.2022	0.41978842278910300	12630
**TTC17**	0.33729	1.3052	0.4198705495426190	12631
**GRAMD1B**	0.33728	2.1469	0.41989792575643400	12633
**NPS**	0.33728	1.6565	0.41989792575643400	12634
**SLC31A1**	0.33728	2.1945	0.41989792575643400	12632
**MAST1**	0.66287	0.25142	0.4203086067433310	12635
**MAN2B2**	0.33713	2.2521	0.42030860674333200	12636
**SLC27A3**	0.33711	2.5162	0.42036336956320500	12637

**SPAM1**	0.33691	2.231	0.4209110671367230	12638
**ALKBH5**	0.33672	0.52349	0.4214314968058480	12639
**NWD1**	0.3367	1.8947	0.4214862855143910	12640
**GAPDH**	0.33668	2.7738	0.42154107548819100	12641
**MMP10**	0.33668	2.4121	0.42154107548819100	12642
**SLC39A6**	0.33668	2.0596	0.42154107548819100	12643
**TH**	0.66349	0.15374	0.4220068413817080	12644
**KCTD1**	0.33649	1.1586	0.42206164338656300	12645
**FTH1**	0.66352	0.15288	0.42208904486432500	12646
**POLK**	0.33644	2.4959	0.42219865394492900	12647
**P4HA2**	0.33638	0.30415	0.422363077077567	12648
**NIPA2**	0.6637	0.55943	0.42258232568569800	12649
**CAPN7**	0.33624	0.88324	0.4227467754730790	12650
**ENOSF1**	0.66376	0.16726	0.4227467754730790	12651
**GDF10**	0.33604	0.092837	0.42329502404036400	12652
**ZNF292**	0.33596	1.2704	0.4235143590883510	12653
**C22orf29**	0.33593	2.1654	0.423596614983866	12654
**COX6B2**	0.66414	0.014144	0.4237885565543250	12655
**CCDC38**	0.33583	1.8689	0.4238708220067380	12656
**ARHGAP42**	0.33572	2.1332	0.4241724865480770	12658
**CAMSAP3**	0.33572	1.6415	0.4241724865480770	12660
**CLIC3**	0.33572	0.57173	0.4241724865480770	12664
**KBTBD11**	0.33572	1.3816	0.4241724865480770	12661
**METTL25**	0.33572	2.0945	0.4241724865480770	12659
**TADA2B**	0.33572	2.236	0.4241724865480770	12657
**USP34**	0.33572	1.2175	0.4241724865480770	12663
**ZHX1**	0.33572	1.3455	0.4241724865480770	12662
**PIK3C2B**	0.33568	1.0461	0.42428219231458300	12665
**RPL21**	0.33557	0.51774	0.42458390950908100	12666
**STARD3NL**	0.33555	0.71655	0.4246387713328860	12667
**CBX7**	0.33545	2.3636	0.42491309962827500	12671
**FBXO38**	0.33545	1.8608	0.42491309962827500	12673

**GABRA4**	0.33545	2.9298	0.42491309962827500	12668
**KLHL12**	0.33545	2.6873	0.42491309962827500	12669
**TNFAIP8L3**	0.33545	2.1232	0.42491309962827500	12672
**WDR86**	0.33545	2.3974	0.42491309962827500	12670
**TKT**	0.33517	0.53302	0.42568138908513800	12674
**PPM1M**	0.66484	0.5247	0.42570883263933500	12675
**ARHGEF12**	0.66496	0.45956	0.42603818030673400	12676
**MEA1**	0.33487	0.95477	0.42650483529859200	12678
**NID1**	0.33487	2.2135	0.42650483529859200	12677
**CMPK2**	0.66516	0.1014	0.4265871958189210	12679
**GNE**	0.66518	0.31839	0.4266421044400780	12680
**OR51A4**	0.66525	0.70319	0.4268342947455850	12681
**PEAR1**	0.66544	0.096093	0.4273560336548580	12682
**PIGL**	0.33446	0.1762	0.42763067980506400	12683
**METTL12**	0.33444	0.79735	0.4276856129054490	12684
**PQLC3**	0.66569	0.18124	0.428042709527482	12685
**LRRN4CL**	0.6657	0.42763	0.4280701807588160	12686
**RSBN1**	0.66572	0.42487	0.428125124190683	12687
**ADAMTS15**	0.33421	2.2142	0.4283174363817900	12688
**CLDN5**	0.33421	1.4968	0.4283174363817900	12690
**OR51B4**	0.33421	1.9519	0.4283174363817900	12689
**AMH**	0.33406	1.0271	0.42872958729314500	12695
**HIST1H2BI**	0.33406	1.5138	0.42872958729314500	12693
**MB21D1**	0.33406	1.3625	0.42872958729314500	12694
**PDE4D**	0.33406	1.9988	0.42872958729314500	12692
**TBC1D26**	0.33406	2.0181	0.42872958729314500	12691
**CD3EAP**	0.66612	0.03143	0.4292242645452780	12696
**LCN1**	0.66614	0.13236	0.4292792351663480	12697
**TIMMDC1**	0.66615	0.37723	0.42930672096332800	12698
**CNGA1**	0.33373	0.93151	0.42963657583326000	12699
**SIL1**	0.66644	0.005286	0.43010395027645300	12700
**VPS36**	0.33355	2.4526	0.4301314458158220	12701

**LDHB**	0.33354	1.2802	0.4301589416803790	12702
**CYB561A3**	0.33343	1.8401	0.4304614176610290	12703
**SNRNP200**	0.33339	1.9991	0.430571418690606	12704
**ZNF565**	0.66684	0.42542	0.4312040257790380	12705
**KRTAP19-7**	0.66688	0.0046828	0.4313140620135330	12706
**PROZ**	0.33305	1.6567	0.4315066379919370	12707
**NUDT18**	0.33302	0.97684	0.43158917545274700	12708
**CHST10**	0.33299	2.3046	0.43167171585385500	12710
**DRAXIN**	0.33299	2.7546	0.43167171585385500	12709
**FAM26D**	0.33299	0.11205	0.43167171585385500	12711
**WFDC2**	0.33297	1.6916	0.43172674442180700	12712
**ZBTB17**	0.33283	0.18276	0.43211198101665700	12713
**ADGRA3**	0.33274	1.5542	0.43235966697808100	12714
**PCMT1**	0.33268	1.0654	0.43252480568861400	12715
**MLPH**	0.33258	2.2962	0.4328000630897130	12716
**DPH6**	0.33253	0.095747	0.43293770408694800	12717
**PRY2**	0.6676	0.44454	0.43329560907674000	12718
**DIABLO**	0.33231	0.74479	0.4335434219696110	12719
**DUSP2**	0.33226	0.85192	0.4336811073033450	12726
**FBXO9**	0.33226	1.6385	0.4336811073033450	12722
**MEX3D**	0.33226	1.5929	0.4336811073033450	12723
**PEX11A**	0.33226	1.3168	0.4336811073033450	12725
**S1PR1**	0.33226	2.5089	0.4336811073033450	12720
**SETD7**	0.33226	2.131	0.4336811073033450	12721
**SNX33**	0.33226	1.3744	0.4336811073033450	12724
**CFHR2**	0.33223	1.4962	0.43376372244989100	12727
**LCE5A**	0.66784	0.11426	0.4339565026402570	12728
**C8orf22**	0.33203	1.1548	0.43431456579278700	12729
**OR4S1**	0.66814	0.58655	0.43478288626539700	12730
**PNMA5**	0.33179	0.70146	0.4349757518214450	12731
**THBS3**	0.66824	0.23273	0.43505841344121100	12732
**PRB1**	0.33176	0.19461	0.4350584134412120	12733

**C16orf97**	0.33174	1.5357	0.43511352283926100	12735
**DPH3P1**	0.33174	2.0862	0.43511352283926100	12734
**EXD1**	0.33173	0.043553	0.43514107803383400	12736
**ASH2L**	0.66829	0.3439	0.4351961894142230	12737
**RGL3**	0.3317	2.6046	0.43522374560009400	12738
**HIST1H3H**	0.66831	0.41236	0.4352513021164570	12739
**TM7SF3**	0.66833	0.069654	0.4353064161407680	12740
**ATP8B2**	0.33162	1.1824	0.4354442069871540	12741
**SORD**	0.33159	1.8039	0.43552688546341200	12742
**POLE2**	0.33156	2.1157	0.4356095669169420	12743
**CCZ1**	0.33151	1.4273	0.4357473759577720	12744
**TNFRSF12A**	0.66851	0.42579	0.43580250189117600	12745
**PABPN1L**	0.66855	0.057165	0.43591275773137700	12746
**POLR2J3**	0.66874	0.79362	0.43643654537386200	12747
**SLC9A3R1**	0.3311	1.5805	0.43687772258966500	12748
**PIGR**	0.33104	1.1809	0.43704318596609700	12749
**SYCE1**	0.33091	0.70381	0.43740173100803700	12750
**PATL1**	0.33078	1.3538	0.4377603322891820	12751
**TRHR**	0.33078	1.0355	0.4377603322891820	12752
**COX10**	0.33074	0.10785	0.43787068246918300	12753
**CDC14A**	0.33063	2.4825	0.4381741729645450	12754
**ESPNL**	0.66937	0.019467	0.4381741729645450	12755
**GPR83**	0.66943	0.2185	0.43833973024533900	12756
**NLRX1**	0.33052	1.319	0.43847770382424600	12757
**TEF**	0.33042	1.0575	0.438753676028908	12758
**SCN1B**	0.66981	0.25768	0.43938853897664500	12759
**BMP1**	0.33015	0.53976	0.4394989679971290	12761
**NBR1**	0.66985	0.65206	0.43949896799712900	12760
**ADARB2**	0.33009	0.017517	0.4396646215780620	12776
**ADORA2A**	0.33009	2.2286	0.4396646215780620	12763
**AGA**	0.33009	2.6318	0.4396646215780620	12762
**CA5A**	0.33009	0.9397	0.4396646215780620	12768

**CHST13**	0.33009	1.4073	0.4396646215780620	12767
**CTBS**	0.66991	0.18362	0.4396646215780620	12775
**GLRA2**	0.33009	1.8919	0.4396646215780620	12764
**GOLT1A**	0.33009	0.78339	0.4396646215780620	12772
**ME3**	0.33009	0.87367	0.4396646215780620	12771
**NKG7**	0.33009	0.92818	0.4396646215780620	12770
**NPEPPS**	0.33009	1.8654	0.4396646215780620	12765
**NUBP2**	0.33009	0.93878	0.4396646215780620	12769
**PAX9**	0.33009	1.7416	0.4396646215780620	12766
**PPP1R3B**	0.33009	0.68786	0.4396646215780620	12774
**VMP1**	0.33009	0.74349	0.4396646215780620	12773
**GRIN2D**	0.33005	0.51799	0.43977506400154500	12777
**ERICH1**	0.33002	2.4782	0.4398578993394880	12778
**HDAC10**	0.67006	0.41312	0.440078808332285	12779
**GPC4**	0.32993	1.2051	0.44010642346628000	12780
**ATXN10**	0.32989	0.76797	0.4402168873588500	12781
**FAM162B**	0.32975	2.1686	0.4406035532992010	12782
**COX7A2L**	0.67028	0.36765	0.4406864188556630	12783
**DDX5**	0.67037	0.17192	0.4409350336853730	12784
**CD47**	0.32951	1.2969	0.44126656252858900	12785
**NHSL1**	0.67056	0.2187	0.44145997675165900	12786
**EXD3**	0.32939	2.0154	0.4415981398794690	12787
**CLIP4**	0.32935	1.0757	0.44170867645133200	12788
**OPRK1**	0.32917	1.774	0.44220615784276400	12789
**CCDC120**	0.32914	1.149	0.4422890820458540	12793
**HMCES**	0.32914	2.0477	0.4422890820458540	12791
**PDE1C**	0.32914	1.0108	0.4422890820458540	12794
**POLN**	0.32914	2.1099	0.4422890820458540	12790
**UGDH**	0.32914	1.8612	0.4422890820458540	12792
**KIAA1522**	0.67098	0.35474	0.4426208092811540	12795
**FIS1**	0.6711	0.52526	0.44295258523126800	12796
**COMMD2**	0.32865	1.5449	0.44364394181216000	12797

**ENPP3**	0.67136	0.2146	0.44367160048435900	12798
**ALX4**	0.32858	1.7101	0.4438375596463350	12799
**MED13L**	0.67144	0.17974	0.4438928820834260	12800
**DOT1L**	0.32847	1.7835	0.4441418498665350	12801
**C11orf71**	0.32845	1.1829	0.44419717977890000	12802
**ZNF185**	0.67182	0.59914	0.4449442667932400	12803
**HAGH**	0.32807	1.7411	0.4452487067659120	12804
**ALKBH4**	0.32803	1.5371	0.4453594224414700	12809
**CCDC84**	0.32803	1.6543	0.4453594224414700	12808
**DENND3**	0.32803	2.7828	0.4453594224414700	12805
**FBXW12**	0.32803	1.2387	0.4453594224414700	12811
**LTB**	0.32803	2.0464	0.4453594224414700	12807
**NPW**	0.32803	2.3974	0.4453594224414700	12806
**PCDHGA3**	0.32803	1.2565	0.4453594224414700	12810
**HNRNPCL3**	0.32794	1.0023	0.4456085526756910	12812
**ATP10B**	0.67207	0.11975	0.4456362355198610	12813
**LBH**	0.6722	0.083041	0.44599614358277900	12814
**ENO2**	0.32762	1.4707	0.44649457322794900	12818
**PGAM2**	0.32762	1.8785	0.44649457322794900	12817
**PIRT**	0.32762	2.5308	0.44649457322794900	12815
**TMBIM1**	0.32762	2.2076	0.44649457322794900	12816
**EYA3**	0.32759	0.58434	0.4465776556175180	12819
**TCIRG1**	0.32751	1.9791	0.4467992237296280	12820
**KPNA2**	0.32742	0.97718	0.44704851407783300	12821
**ITPRIPL2**	0.32738	1.2849	0.4471593187056390	12822
**ARPC2**	0.32734	2.5855	0.44727012882383000	12823
**NAT6**	0.32729	0.80885	0.4474086491953260	12824
**MAN2B1**	0.32726	2.0142	0.4474917655389890	12825
**USE1**	0.67275	0.60871	0.44751947167385000	12826
**SGCD**	0.67283	0.10686	0.4477411331225410	12827
**CCDC113**	0.32713	0.95655	0.44785197209652000	12828
**NHLRC4**	0.67297	0.002546	0.4481290936082070	12830

**THBS2**	0.32703	0.82158	0.44812909360820700	12829
**RHBDD2**	0.32691	2.0709	0.44846168485820500	12831
**ACVR1C**	0.3268	2.2807	0.4487666037550450	12832
**NLN**	0.67323	0.55871	0.44884977069496400	12833
**CCDC150**	0.32675	0.62218	0.4489052170463700	12834
**FANCL**	0.32667	1.838	0.4491270162555150	12835
**CLDND1**	0.67335	0.20092	0.44918246950987800	12836
**DHX32**	0.32664	0.99639	0.44921019665503600	12837
**PLAGL2**	0.32661	1.7447	0.4492933801627710	12838
**RREB1**	0.32656	0.89317	0.4494320262516700	12839
**GDF2**	0.32633	1.8157	0.45006990960059900	12843
**GNB5**	0.32633	0.42517	0.45006990960059900	12845
**INO80D**	0.32633	1.9054	0.45006990960059900	12842
**PCGF2**	0.32633	1.9572	0.45006990960059900	12841
**PLEKHG7**	0.32633	2.6082	0.45006990960059900	12840
**RBM12B**	0.32633	1.3326	0.45006990960059900	12844
**ADGRL3**	0.67369	0.68815	0.45012538636979800	12846
**GIGYF2**	0.67372	0.2667	0.45020860412126300	12847
**GNAI1**	0.32613	1.4323	0.45062473966362800	12849
**HUS1**	0.67387	0.46342	0.45062473966362800	12850
**SGCG**	0.32613	2.8165	0.45062473966362800	12848
**CYP3A4**	0.32595	0.47792	0.45112420534855100	12851
**THEG5**	0.32592	0.36026	0.451207460568586	12852
**PLEKHA8**	0.32584	0.3019	0.451429489781641	12853
**CCDC54**	0.67422	0.37562	0.45159602629642200	12854
**ANLN**	0.67431	0.1532	0.45184585455647300	12855
**BORA**	0.32562	1.4924	0.4520401849240130	12856
**AP1B1**	0.6744	0.33505	0.4520957110215000	12857
**ITGB6**	0.67452	0.38057	0.45242889688725300	12859
**NTN5**	0.32548	0.73378	0.45242889688725300	12858
**KPNA4**	0.32546	2.1074	0.4524844327472690	12860
**CT47A7**	0.32543	2.0894	0.4525677391541420	12861

**PHF7**	0.67465	0.20321	0.4527899049304050	12862
**TTC21A**	0.3253	1.5965	0.4529287698897320	12863
**INVS**	0.67477	0.1479	0.45312319550786000	12864
**FGF9**	0.32517	1.4191	0.45328985967160700	12865
**NEDD9**	0.32509	0.23851	0.45351209814537000	12867
**PACRG**	0.32509	1.8597	0.45351209814537000	12866
**MAPK1**	0.32502	0.36959	0.45370657518550000	12868
**B4GALT3**	0.325	2.2438	0.4537621432057530	12869
**POM121L12**	0.675	0.76919	0.45376214320575300	12870
**ARV1**	0.32498	1.1261	0.45381771262718100	12871
**SERPINA5**	0.67514	0.16278	0.45415115859399000	12872
**SIT 1.00**	0.32485	2.2166	0.4541789480368340	12875
**HLF**	0.32485	1.4386	0.4541789480368340	12873
**TRO**	0.32485	2.0069	0.4541789480368340	12874
**PRR13**	0.32481	2.0147	0.45429010931599800	12876
**FGF8**	0.32478	2.1778	0.45437348395939500	12877
**NME5**	0.32473	1.5283	0.45451244871800200	12878
**RAB11FIP4**	0.32468	0.99034	0.45465142225441300	12879
**SRRT**	0.67538	0.59153	0.45481820209014800	12880
**SOWAHB**	0.32457	1.9602	0.4549571949510810	12881
**GALNT6**	0.3245	0.24281	0.45515179972435	12882
**YWHAQ**	0.67554	0.79966	0.4552630101910980	12883
**FAM45A**	0.32444	1.1484	0.45531861753594200	12884
**DEDD**	0.32438	0.80183	0.4554854480192820	12892
**GORAB**	0.32438	1.4419	0.4554854480192820	12887
**GRK7**	0.32438	0.84582	0.4554854480192820	12891
**HUNK**	0.32438	1.2145	0.4554854480192820	12890
**KHNYN**	0.32438	1.3365	0.4554854480192820	12889
**MCEMP1**	0.32438	2.499	0.4554854480192820	12885
**MRPL19**	0.32438	0.7573	0.4554854480192820	12893
**PTGIS**	0.32438	0.52869	0.4554854480192820	12894
**RASL11A**	0.32438	2.4684	0.4554854480192820	12886

**TMEM41A**	0.32438	1.4114	0.4554854480192820	12888
**NMNAT2**	0.67568	0.14527	0.4556522911809400	12895
**TNFRSF19**	0.32413	0.92417	0.4561807115758910	12896
**SLC16A7**	0.32406	2.2601	0.45637542488018000	12897
**KLK12**	0.32404	2.2046	0.45643106043072800	12898
**DCAF12**	0.32386	0.34441	0.4569318439929350	12899
**AK4**	0.67616	0.43834	0.45698749368291000	12900
**MOB4**	0.3238	1.388	0.4570987973089600	12901
**C6orf141**	0.32375	1.151	0.45723793480548500	12902
**FAM180A**	0.32373	0.59457	0.4572935922825560	12903
**MFSD3**	0.67638	0.51158	0.4575997337352690	12904
**TSC1**	0.3236	2.8557	0.4576554004248080	12905
**ARL 1.00**	0.67647	0.030723	0.45785024500829000	12906
**TMEM5**	0.32351	2.1156	0.4579059180817500	12907
**RETREG2**	0.3234	1.9256	0.4582121453627330	12908
**MECOM**	0.32332	1.5567	0.45843488310294200	12909
**HMX3**	0.3233	1.5762	0.4584905710915090	12910
**HSFY1**	0.32329	0.96292	0.4585184156189880	12911
**INTS7**	0.32326	1.2578	0.4586019513345750	12912
**PIAS4**	0.67681	0.25623	0.4587968804515950	12913
**INTS10**	0.32316	1.5926	0.4588804268383050	12914
**FAM126B**	0.32308	1.8867	0.45910323286487300	12915
**PROC**	0.32296	2.0688	0.459437484647545	12916
**OR2H1**	0.32293	0.0030552	0.45952105561279200	12917
**FAM208B**	0.3229	0.63685	0.45960462978753100	12918
**ASB7**	0.67715	0.26573	0.4597439272132220	12919
**CFH**	0.67719	0.0066609	0.45985537157700200	12921
**ITGB1**	0.67719	0.49001	0.45985537157700200	12920
**PTGER2**	0.32278	1.0187	0.4599389585980130	12922
**SCP2D1**	0.32274	2.4554	0.4600504129585060	12923
**NEUROD1**	0.67734	0.49198	0.4602733388268180	12924
**TOR1AIP1**	0.67746	0.15684	0.4606077705271650	12925

**C12orf29**	0.32252	1.7535	0.46066351415180700	12927
**INCENP**	0.32252	2.4772	0.46066351415180700	12926
**KIAA1191**	0.32238	0.37357	0.46105375961987400	12929
**SLC12A6**	0.32238	1.5136	0.46105375961987400	12928
**BHLHE41**	0.32234	0.85343	0.4611652712203070	12931
**S100A8**	0.32234	1.9747	0.4611652712203070	12930
**COQ7**	0.6777	0.15791	0.46127678855559300	12932
**LDB2**	0.67774	0.19678	0.4613883116277100	12933
**RPS4X**	0.32224	0.3194	0.4614440753156970	12934
**C9orf172**	0.67777	0.039411	0.46147195769778200	12935
**GLIPR2**	0.67785	0.55375	0.461695029672711	12936
**GSTA2**	0.32213	1.1944	0.46175080125598000	12937
**ANKRD1**	0.32212	2.1706	0.4617786875862160	12943
**CPNE3**	0.32212	0.19212	0.4617786875862160	12948
**DAPK1**	0.32212	2.5873	0.4617786875862160	12938
**DUSP23**	0.32212	2.2618	0.4617786875862160	12940
**HCRTR1**	0.32212	0.11356	0.4617786875862160	12949
**KLHL3**	0.32212	2.1142	0.4617786875862160	12944
**LRRC46**	0.32212	2.1857	0.4617786875862160	12942
**MEGF11**	0.32212	2.4351	0.4617786875862160	12939
**PYGM**	0.32212	1.7359	0.4617786875862160	12945
**RAB11B**	0.32212	1.6925	0.4617786875862160	12946
**STK19**	0.32212	2.2476	0.4617786875862160	12941
**ZNF548**	0.32212	1.0648	0.4617786875862160	12947
**CTSA**	0.32208	0.42365	0.46189023649855100	12950
**TARDBP**	0.67801	0.56243	0.4621412425694020	12951
**ESPL1**	0.32182	1.4592	0.4626154446178070	12952
**SYNCRIP**	0.3218	1.4783	0.46267123993185700	12953
**DAW1**	0.32169	0.24483	0.46297813991290000	12954
**PTPN11**	0.67832	0.040194	0.4630060420732010	12956
**ZCCHC17**	0.32168	0.61434	0.4630060420732010	12955
**ATF7IP**	0.32152	2.0731	0.4634525256832810	12957

**ATP6V0A1**	0.67852	0.35817	0.46356416101957700	12958
**ANKS6**	0.32141	2.5827	0.46375953676137300	12960
**CEP70**	0.32141	2.7617	0.46375953676137300	12959
**FMN2**	0.32141	1.4806	0.46375953676137300	12965
**KCTD5**	0.32141	2.2369	0.46375953676137300	12962
**MXRA7**	0.32141	2.1711	0.46375953676137300	12963
**ZNF655**	0.32141	2.4686	0.46375953676137300	12961
**ZNF709**	0.32141	1.8669	0.46375953676137300	12964
**FGD5**	0.32133	1.2116	0.46398284500268400	12966
**THAP11**	0.3212	1.9374	0.46434577025477	12967
**CFI**	0.67882	0.6994	0.4644016103366880	12968
**NUDT9**	0.32103	1.8212	0.46482045712667400	12969
**LY6K**	0.679	0.25173	0.46490423626864500	12970
**MID1IP1**	0.32086	2.1075	0.4652952487593950	12971
**KRBA1**	0.32077	2.5345	0.465546651499688	12972
**SLCO3A1**	0.32066	0.96583	0.46585396148153700	12973
**C21orf140**	0.32051	0.4874	0.4662730914675290	12974
**PLA2G2A**	0.3201	1.2872	0.467419131749816	12975
**NR0B2**	0.32007	1.6877	0.46750301244632200	12976
**ATP1A2**	0.32004	2.4343	0.46758689643232000	12977
**C18orf21**	0.32004	1.8387	0.46758689643232000	12979
**H2BFM**	0.32004	1.8355	0.46758689643232000	12980
**KRT34**	0.32004	1.845	0.46758689643232000	12978
**NOD1**	0.32004	0.25953	0.46758689643232000	12982
**ZNF83**	0.32004	1.6769	0.46758689643232000	12981
**UBE2Q1**	0.67998	0.42667	0.4676428209175670	12983
**C12orf10**	0.31999	1.1768	0.4677267103879370	12988
**CADM4**	0.31999	2.495	0.4677267103879370	12985
**CAVIN4**	0.31999	1.5512	0.4677267103879370	12987
**FAM86B1**	0.31999	2.019	0.4677267103879370	12986
**VTI1B**	0.31999	3.1177	0.4677267103879370	12984
**CHST6**	0.68008	0.12638	0.4679224652882030	12989

**EDC4**	0.68012	0.067627	0.46803433328187400	12990
**PEX10**	0.6802	0.20344	0.4682580868438580	12991
**PGK 2.00**	0.31979	2.1532	0.46828605768734300	12992
**CXADR**	0.31965	1.0023	0.4686776879803220	12993
**DIO3**	0.31949	1.241	0.46912535350002600	12994
**RUNDC1**	0.31922	2.4082	0.46988100236404700	12995
**PPP1R9A**	0.31881	2.5725	0.47102898260381900	12996
**RBBP7**	0.31873	0.57699	0.47125305109722000	12997
**CLDN14**	0.31871	1.8425	0.4713090719172380	13001
**EEF2**	0.31871	2.256	0.4713090719172380	13000
**PCDHGA9**	0.31871	2.9912	0.4713090719172380	12998
**TCF15**	0.31871	2.8468	0.4713090719172380	12999
**SEMA3B**	0.31868	1.0215	0.47139310592079800	13002
**IL23R**	0.68134	0.5651	0.4714491304392290	13003
**DEFB124**	0.31865	2.3504	0.47147714325335800	13004
**HIP1R**	0.68138	0.61046	0.4715611839157760	13005
**PCDH12**	0.31841	2.5792	0.47214956186078500	13006
**HDAC8**	0.31835	0.70399	0.4723176998627060	13007
**AIMP2**	0.31813	2.3084	0.4729343201709610	13008
**SERPINF1**	0.31809	2.0407	0.4730464522695550	13009
**MKRN2OS**	0.31803	1.2663	0.4732146615710760	13010
**ELMOD1**	0.3179	1.9746	0.47357916100368400	13014
**KRT74**	0.3179	1.0347	0.47357916100368400	13017
**NDUFA4**	0.3179	1.5885	0.47357916100368400	13015
**OSBPL10**	0.3179	2.0548	0.47357916100368400	13013
**PRMT7**	0.3179	1.4063	0.47357916100368400	13016
**PYY**	0.3179	2.6377	0.47357916100368400	13011
**VPS52**	0.3179	2.1762	0.47357916100368400	13012
**ZNF821**	0.3179	0.004878[illegible]	0.47357916100368400	13018
**CCL18**	0.68213	0.1974	0.473663285191754	13019
**ZNF362**	0.31781	0.19622	0.47383154362540900	13020
**RTBDN**	0.31775	0.668	0.47399981547483400	13021

**GCNT4**	0.31772	1.4827	0.47408395643261900	13022
**PRPF4B**	0.31757	1.642	0.47450471158680200	13023
**ACOT12**	0.68247	0.32711	0.4746169271470570	13024
**ELAC1**	0.31747	1.521	0.4747852616947650	13028
**PBX2**	0.31747	1.6206	0.4747852616947650	13027
**RPP25L**	0.31747	1.731	0.4747852616947650	13026
**UTF1**	0.31747	1.7857	0.4747852616947650	13025
**ELOVL1**	0.31742	1.6144	0.4749255507623070	13029
**STRADA**	0.6828	0.14815	0.4755429337643230	13030
**HLA-DQB1**	0.31716	2.2766	0.4756552046891720	13031
**PI4K2B**	0.31708	1.8352	0.4758797645285850	13032
**CD27**	0.68299	0.55046	0.476076274075006	13033
**TBC1D28**	0.68311	0.35173	0.4764131903649160	13034
**PDCD6**	0.31674	0.54703	0.47683441179506400	13035
**CLIC6**	0.68331	0.5234	0.4769748377377830	13036
**MALSU1**	0.68336	0.08744	0.47711527308688400	13037
**TEDDM1**	0.68363	0.5489	0.4778737866861720	13038
**TOE1**	0.31635	0.69542	0.4779299838122610	13039
**LEUTX**	0.31633	2.6768	0.477986182447761	13040
**ICOS**	0.68372	0.47999	0.47812668564188100	13041
**MTF2**	0.68375	0.14677	0.4782109920890430	13042
**LRP5L**	0.68377	0.076281	0.4782671982754780	13043
**WNT7B**	0.68395	0.065871	0.47877312197779500	13044
**HSPG2**	0.316	0.83419	0.4789136780850820	13045
**ATP13A5**	0.68421	0.011471	0.4795041171094360	13046
**LSM 1.00**	0.31553	1.593	0.48023536855781600	13050
**ADRA2B**	0.31553	2.2083	0.48023536855781600	13049
**APOH**	0.31553	2.2527	0.48023536855781600	13055
**ARHGAP32**	0.31553	1.4941	0.48023536855781600	13047
**BDP1**	0.31553	3.0025	0.48023536855781600	13057
**CD5**	0.31553	0.80571	0.48023536855781600	13058
**DUPD1**	0.31553	0.25636	0.48023536855781600	13056

**EEF1G**	0.31553	1.3564	0.48023536855781600	13053
**KIAA1456**	0.31553	1.6944	0.48023536855781600	13054
**SLC30A4**	0.31553	2.3155	0.48023536855781600	13048
**SNX21**	0.31553	1.8558	0.48023536855781600	13051
**SOX9**	0.31553	1.8282	0.48023536855781600	13052
**GBX2**	0.31542	1.8681	0.4805448214698520	13059
**IL3RA**	0.31542	0.84745	0.4805448214698520	13060
**ABCB8**	0.31523	3.0395	0.4810794394752280	13061
**BIN1**	0.31523	2.4441	0.4810794394752280	13062
**OSTN**	0.3149	2.3856	0.4820083135559350	13063
**BCL3**	0.31484	1.9064	0.4821772444197140	13064
**NRSN1**	0.68518	0.088789	0.4822335577653860	13065
**C9orf78**	0.31479	1.7758	0.4823180306514310	13066
**TFAP2C**	0.31476	1.9705	0.4824025069793030	13067
**CDKN2AIPN**	0.68535	0.31196	0.48271228297586500	13068
**SELENOI**	0.31463	1.5844	0.482768610860541	13069
**PSMA1**	0.31455	2.6327	0.4829939377198300	13070
**SPEM1**	0.3144	2.3572	0.4834164916981310	13071
**FAM192A**	0.31431	1.4697	0.48367006551796700	13072
**KIF5B**	0.68571	0.4201	0.4837264194788500	13073
**ASNS**	0.68574	0.098788	0.483810953300738	13074
**SYT6**	0.6858	0.32022	0.4839800313177970	13075
**C16orf96**	0.31415	0.12998	0.4841209402349430	13076
**KATNB1**	0.68588	0.25842	0.4842054901992600	13077
**C6orf226**	0.31407	1.3362	0.48434641449935	13078
**CACNG6**	0.31402	3.0471	0.484487348419125	13079
**CRADD**	0.31402	2.3445	0.484487348419125	13082
**CYSLTR2**	0.31402	2.6013	0.484487348419125	13081
**YY1**	0.31402	2.7736	0.484487348419125	13080
**TSEN34**	0.31391	2.2404	0.48479743692419200	13083
**PSG6**	0.68617	0.42618	0.4850229851183370	13084
**ATG5**	0.31382	2.4091	0.48505118037735900	13085

**NR2F1**	0.31374	1.04	0.48527675633428400	13086
**PIWIL2**	0.31368	2.3453	0.4854459545077810	13087
**DENND5B**	0.31362	2.1245	0.4856151665798840	13088
**ALDH1L2**	0.3135	2.5674	0.48595363244842600	13089
**MCOLN1**	0.3135	0.37298	0.48595363244842600	13094
**NDRG1**	0.3135	1.3481	0.48595363244842600	13091
**PGLYRP4**	0.3135	0.20519	0.48595363244842600	13096
**PNLDC1**	0.3135	0.3567	0.48595363244842600	13095
**PRSS46**	0.3135	0.85504	0.48595363244842600	13093
**TBC1D9**	0.3135	0.080608	0.48595363244842600	13097
**ZNF527**	0.3135	1.6053	0.48595363244842600	13090
**ZNF75A**	0.3135	0.95489	0.48595363244842600	13092
**LMOD2**	0.68664	0.28522	0.4863485796724260	13098
**GKN1**	0.31328	0.63107	0.4865742978620150	13099
**TOR2A**	0.3132	0.248	0.48680004084484600	13100
**RELT**	0.31314	1.9845	0.4869693643627320	13101
**SMIM21**	0.3131	1.9089	0.4870822544647090	13102
**GRID2IP**	0.31308	0.38446	0.4871387018435090	13103
**UFD1**	0.68696	0.43585	0.4872516012580580	13104
**RHBG**	0.313	0.44022	0.4873645068836400	13105
**KIDINS220**	0.31285	2.1294	0.487787958332834	13108
**KRT72**	0.31285	2.7324	0.487787958332834	13107
**STXBP3**	0.31285	2.8541	0.487787958332834	13106
**STT3B**	0.31284	2.2611	0.4878161915387690	13109
**ARHGAP33**	0.68719	0.052952	0.4879008934898270	13110
**PRIM2**	0.68722	0.22693	0.487985598941462	13111
**ABHD16A**	0.68727	0.021697	0.4881267824756860	13112
**NGLY1**	0.31262	2.1743	0.48843742050809800	13113
**KIAA1143**	0.68753	0.19256	0.4888610938027200	13114
**PRIMPOL**	0.31244	0.069959	0.48894583899032000	13115
**KIF12**	0.31239	2.331	0.48908708877378400	13116
**ANPEP**	0.68765	0.53997	0.489200095626669	13117

**RALGDS**	0.68766	0.15364	0.4892283483160180	13118
**MFSD2A**	0.31221	2.6617	0.48959566882678200	13119
**TFB1M**	0.31218	1.9667	0.489680444478657	13120
**STAB1**	0.31206	0.2697	0.49001958228980400	13121
**ABCD3**	0.3118	1.3679	0.49075457433515300	13130
**APOF**	0.3118	1.7702	0.49075457433515300	13127
**BIRC6**	0.3118	1.7937	0.49075457433515300	13126
**HAS1**	0.3118	1.3938	0.49075457433515300	13129
**NOS3**	0.3118	0.25169	0.49075457433515300	13131
**OGT**	0.3118	1.9093	0.49075457433515300	13125
**TCN2**	0.3118	1.9729	0.49075457433515300	13124
**TMEM243**	0.3118	2.1377	0.49075457433515300	13123
**TTYH2**	0.3118	1.6291	0.49075457433515300	13128
**WTH3DI**	0.3118	2.316	0.49075457433515300	13122
**ALG3**	0.31174	2.1571	0.4909242255255830	13135
**C7orf43**	0.31174	3.1202	0.4909242255255830	13132
**HMBOX1**	0.31174	2.3502	0.4909242255255830	13134
**RNF11**	0.31174	2.993	0.4909242255255830	13133
**RBP5**	0.31165	2.5876	0.4911787288088030	13136
**STAT5B**	0.68836	0.4125	0.49120700891506000	13137
**ENO4**	0.68838	0.63118	0.4912635703061830	13138
**ELMO2**	0.68841	0.33024	0.4913484153398910	13139
**HBQ1**	0.31154	1.295	0.491489831590372	13140
**P2RY6**	0.31154	0.51622	0.491489831590372	13141
**DDX47**	0.3115	1.4905	0.49160297166804400	13142
**C1orf186**	0.31136	0.90459	0.491999011513333	13143
**ADAD1**	0.31094	0.67473	0.49318759452521900	13144
**TSFM**	0.31074	0.81664	0.4937538313140430	13145
**TANK**	0.31062	0.22352	0.4940936493803770	13146
**DDIAS**	0.3106	1.2062	0.49415029127128500	13147
**TCEAL6**	0.31056	0.44835	0.49426357980969100	13148
**CBY1**	0.31054	1.2459	0.4943202264577330	13149

**CAV2**	0.31046	2.686	0.4945468289151250	13151
**CST8**	0.31046	2.4256	0.4945468289151250	13154
**IFNLR1**	0.31046	2.6872	0.4945468289151250	13150
**NTPCR**	0.31046	2.6811	0.4945468289151250	13152
**PCDH18**	0.31046	2.4292	0.4945468289151250	13153
**TFPI2**	0.68959	0.1784	0.4946884683469930	13155
**SARS2**	0.31025	1.4653	0.49514178124635000	13156
**ACSM6**	0.31017	1.5295	0.49536847585169	13159
**FBXO39**	0.31017	2.6301	0.49536847585169	13157
**KIAA0368**	0.31017	0.95066	0.49536847585169	13161
**LGALS3**	0.31017	1.6387	0.49536847585169	13158
**MORC4**	0.31017	0.46562	0.49536847585169	13162
**NDUFAF4**	0.31017	0.97089	0.49536847585169	13160
**TMEM37**	0.31017	0.41212	0.49536847585169	13163
**TCEA3**	0.31012	1.9368	0.4955101729079620	13164
**DUSP8**	0.30985	2.0296	0.4962755090494450	13165
**CLU**	0.69025	0.55131	0.4965590406411480	13166
**BBX**	0.30973	1.7222	0.4966157517493250	13167
**CCL7**	0.69052	0.1181	0.49732477545745200	13168
**RANBP3**	0.69062	0.30478	0.4976084549395580	13169
**MAMSTR**	0.30924	1.6512	0.49800567350703400	13170
**BTN3A3**	0.30915	1.6959	0.49826106979630700	13175
**CLIP1**	0.30915	2.5084	0.49826106979630700	13173
**KANSL1L**	0.30915	3.0345	0.49826106979630700	13171
**MSX1**	0.30915	2.7708	0.49826106979630700	13172
**NEMF**	0.30915	2.397	0.49826106979630700	13174
**PAEP**	0.6909	0.35226	0.498402970667277	13176
**CKAP2L**	0.30906	2.7245	0.49851649859016800	13177
**FOXQ1**	0.69102	0.3817	0.49874357371490000	13178
**BTAF1**	0.30895	2.0862	0.49882873351663800	13179
**WDR45**	0.69107	0.15562	0.4988855087276180	13180
**ERI1**	0.30893	0.10664	0.4988855087276190	13181

**CYP4F22**	0.69108	0.28256	0.4989138969361580	13182
**SFT2D1**	0.30883	1.5088	0.4991694089106680	13183
**PLSCR4**	0.30848	1.4702	0.5001633766564390	13184
**TMEM120B**	0.69157	0.30955	0.500305412373235	13185
**ITPK1**	0.3084	1.8753	0.5003906386481710	13186
**CPOX**	0.69165	0.73566	0.5005326905174740	13187
**KRTAP17-1**	0.6918	0.35698	0.5009589067480600	13188
**CCER1**	0.30819	2.3228	0.5009873243985700	13189
**CORT**	0.69183	0.18173	0.5010441609133890	13190
**COA5**	0.30815	1.5582	0.501100999046833	13198
**CYP4F11**	0.30815	0.53001	0.501100999046833	13203
**DDOST**	0.30815	1.3592	0.501100999046833	13199
**GBA3**	0.30815	1.9392	0.501100999046833	13197
**GJA4**	0.30815	1.0361	0.501100999046833	13200
**LY75-CD302**	0.30815	0.15232	0.501100999046833	13205
**MLC1**	0.30815	0.58655	0.501100999046833	13202
**RPL34**	0.30815	2.2337	0.501100999046833	13195
**RYK**	0.30815	0.9187	0.501100999046833	13201
**SEC13**	0.30815	2.4262	0.501100999046833	13193
**SMCO1**	0.30815	0.26885	0.501100999046833	13204
**SPAG17**	0.30815	2.6171	0.501100999046833	13192
**TMTC2**	0.30815	2.2857	0.501100999046833	13194
**TRIM74**	0.30815	2.6848	0.501100999046833	13191
**USP29**	0.30815	2.105	0.501100999046833	13196
**MAPK7**	0.30802	2.2548	0.5014704863872800	13206
**GSTK1**	0.30794	0.85574	0.501697897241951	13207
**HSD11B1**	0.30794	0.37123	0.501697897241951	13208
**WFDC5**	0.69211	0.52305	0.501840042202128	13209
**BTBD16**	0.30781	2.2079	0.5020674952325990	13210
**CYB5R4**	0.69226	0.18762	0.5022665379433740	13211
**DCTN2**	0.30773	1.7088	0.5022949742406310	13212
**CSRNP2**	0.30771	2.6568	0.5023518480537090	13214

**FAM133A**	0.30771	2.6157	0.5023518480537090	13215
**FAM47C**	0.30771	2.8929	0.5023518480537090	13213
**HSPA4L**	0.30771	2.3196	0.5023518480537090	13216
**SUCO**	0.30771	0.19754	0.5023518480537090	13217
**CNOT3**	0.30759	0.38198	0.5026931250664690	13218
**TOPAZ1**	0.69241	0.17522	0.5026931250664690	13219
**ALG9**	0.30755	0.49605	0.5028068970809120	13220
**FXYD3**	0.30753	2.3005	0.50286378552879	13221
**PAX1**	0.69248	0.44824	0.502892230363015	13222
**PTPRK**	0.69254	0.27228	0.503062907914546	13223
**EXTL2**	0.30735	0.28734	0.5033758548293270	13224
**CFAP69**	0.69269	0.48694	0.503489665929786	13225
**MRPL40**	0.69282	0.21384	0.5038595970610910	13226
**QPCT**	0.30712	2.0341	0.5040303577624310	13227
**OR2AG2**	0.30709	2.5418	0.5041157436245720	13228
**TMC4**	0.69305	0.11797	0.5045142595955440	13229
**SLC22A16**	0.69316	0.19429	0.5048274355036900	13230
**PNPLA7**	0.69321	0.009941	0.5049698045570820	13231
**CD2AP**	0.69336	0.59791	0.5053969731508010	13232
**AGPAT5**	0.30656	2.7388	0.5056248341099810	13233
**CPEB2**	0.30644	1.2076	0.5059666747835390	13234
**TINF2**	0.69359	0.24205	0.5060521441894780	13235
**MAPK8IP3**	0.30637	0.97933	0.5061661091483070	13236
**BMT2**	0.30632	2.1835	0.5063085745925220	13239
**SLC25A14**	0.30632	2.4615	0.5063085745925220	13237
**ZNF573**	0.30632	2.1991	0.5063085745925220	13238
**ALG10**	0.30625	2.1513	0.5065080434808640	13241
**NECAB3**	0.30625	1.4596	0.5065080434808640	13243
**NKD1**	0.30625	2.9449	0.5065080434808640	13240
**TIPIN**	0.30625	2.0531	0.5065080434808640	13242
**XCR1**	0.30618	2.3996	0.5067075325242510	13244
**REL**	0.30614	1.3189	0.5068215353171570	13245

**PRRT3**	0.30604	2.5388	0.5071065711242210	13246
**MORN4**	0.30592	1.7135	0.5074486684881590	13247
**STON1-GTF**	0.69419	0.40961	0.5077623099162500	13249
**TCAIM**	0.30581	0.86364	0.5077623099162500	13248
**TMEM72**	0.69426	0.45666	0.5079619259238600	13250
**EDIL3**	0.30571	1.4874	0.5080474818374590	13251
**MIB1**	0.30542	1.7072	0.5088747142123200	13252
**SMIM1**	0.30532	2.1155	0.5091600474893740	13253
**SESTD1**	0.69469	0.2007	0.5091885830967770	13254
**CHCHD4**	0.30517	2.8076	0.5095881251527960	13255
**DEGS1**	0.30517	1.2873	0.5095881251527960	13259
**GEMIN8**	0.30517	2.3346	0.5095881251527960	13257
**KBTBD2**	0.30517	2.4563	0.5095881251527960	13256
**MFSD2B**	0.30517	2.1932	0.5095881251527960	13258
**HEXDC**	0.69488	0.14856	0.5097308384576780	13260
**CRACR2A**	0.30511	1.842	0.5097593823644200	13261
**INMT**	0.69489	0.10335	0.5097593823644200	13262
**SRSF9**	0.305	1.3671	0.5100733927586670	13263
**MFSD8**	0.30476	1.9366	0.5107586810158160	13264
**APOA5**	0.3047	0.32876	0.5109300405528750	13272
**DNAJC5B**	0.3047	2.304	0.5109300405528750	13266
**HOGA1**	0.3047	1.2392	0.5109300405528750	13271
**KIAA1328**	0.3047	1.4874	0.5109300405528750	13267
**LOC1005063**	0.3047	1.3224	0.5109300405528750	13270
**RBM42**	0.3047	1.4843	0.5109300405528750	13268
**SEMG1**	0.3047	1.3672	0.5109300405528750	13269
**SLC29A2**	0.3047	2.4382	0.5109300405528750	13265
**SLC7A9**	0.69531	0.029944	0.5109586019342740	13273
**TYW1**	0.30465	1.3654	0.5110728516284750	13274
**ZYG11B**	0.30462	1.8143	0.5111585432771530	13275
**SEL1L2**	0.30461	0.17082	0.5111871079941440	13276
**CUTA**	0.30432	1.027	0.5120156663733520	13277

**IGFALS**	0.30432	0.028051	0.5120156663733520	13278
**TMEM52B**	0.30425	0.10081	0.512215715876184	13279
**FAM20B**	0.69576	0.022788	0.5122442960498570	13280
**NFIA**	0.69578	0.45407	0.5123014576525090	13281
**CDK14**	0.30405	2.9616	0.5127873988907530	13282
**DUXA**	0.30405	2.2102	0.5127873988907530	13285
**IRF8**	0.30405	2.6573	0.5127873988907530	13284
**OR5M1**	0.30405	2.7922	0.5127873988907530	13283
**TMEM132D**	0.69598	0.56322	0.512873165794944	13286
**REC114**	0.30385	2.7439	0.513359249545625	13287
**CMTM4**	0.30382	0.53796	0.5134450416204710	13288
**WSB2**	0.69639	0.4322	0.514045692018631	13289
**OR7G3**	0.3035	2.0803	0.514360392435003	13291
**SLC44A1**	0.3035	2.8994	0.514360392435003	13290
**SUN2**	0.69665	0.1269	0.5147896114836150	13292
**TAOK2**	0.30328	2.5685	0.5149899461624160	13293
**ALPP**	0.30314	2.453	0.5153906775449680	13294
**COG5**	0.30314	2.4428	0.5153906775449680	13295
**FBXO4**	0.30314	0.12792	0.5153906775449680	13302
**GMNC**	0.30314	1.1036	0.5153906775449680	13301
**STK16**	0.30314	2.1486	0.5153906775449680	13298
**TBRG1**	0.30314	2.3494	0.5153906775449680	13296
**TMEM259**	0.30314	1.6872	0.5153906775449680	13300
**TRMT6**	0.30314	0.018123	0.5153906775449680	13303
**UTY**	0.30314	1.8538	0.5153906775449680	13299
**ZMYM6**	0.30314	2.1858	0.5153906775449680	13297
**RPL7L1**	0.69687	0.062064	0.5154193043816630	13304
**GATA3**	0.30308	1.8373	0.5155624449017530	13305
**LAIR2**	0.30308	0.44491	0.5155624449017530	13306
**APPL2**	0.30301	0.91557	0.5157628593789480	13307
**ATP6V1E1**	0.30275	1.3645	0.5165074374980110	13308
**CEP120**	0.30274	2.8511	0.5165360808346890	13311

**DQX1**	0.30274	2.6451	0.5165360808346890	13313
**LOC1005058**	0.30274	2.0584	0.5165360808346890	13317
**PGM2**	0.30274	2.862	0.5165360808346890	13310
**ROPN1**	0.30274	2.6773	0.5165360808346890	13312
**STPG1**	0.30274	2.4815	0.5165360808346890	13315
**TNF**	0.30274	2.5501	0.5165360808346890	13314
**UGT1A4**	0.30274	2.1686	0.5165360808346890	13316
**ZNF704**	0.30274	2.8722	0.5165360808346890	13309
**NAXD**	0.30263	0.77397	0.5168511855166570	13318
**MFSD6L**	0.69743	0.26212	0.5170230824285180	13319
**KANK2**	0.69747	0.5637	0.5171376888574710	13320
**SEPT12**	0.30239	1.869	0.517538864867787	13321
**KDM6A**	0.30225	1.8722	0.5179401241898380	13322
**AKIRIN1**	0.3021	2.3075	0.5183701374553110	13323
**TMEM262**	0.302	1.96	0.5186568662217470	13324
**HBE1**	0.30195	0.74059	0.518800246595393	13325
**SLC12A8**	0.69805	0.10588	0.5188002465953930	13326
**ARTN**	0.30194	2.5981	0.5188289239499360	13327
**APOC3**	0.69807	0.39642	0.518857601731169	13328
**RAB39B**	0.30189	2.0779	0.5189723171237210	13329
**PCLAF**	0.69813	0.4857	0.519029677381077	13330
**FIGNL2**	0.69828	0.032864	0.5194599337666060	13331
**TFDP1**	0.30167	1.3273	0.519603373930145	13332
**CAPNS2**	0.30166	0.53181	0.5196320632457180	13338
**CCDC88A**	0.30166	2.2343	0.5196320632457180	13333
**CCNY**	0.30166	2.1873	0.5196320632457180	13335
**GPR119**	0.30166	2.1184	0.5196320632457180	13336
**IL17B**	0.30166	2.2047	0.5196320632457180	13334
**SGSM2**	0.30166	0.29694	0.5196320632457180	13340
**UBQLN4**	0.69834	0.33851	0.5196320632457180	13339
**YTHDF3**	0.30166	0.99522	0.5196320632457180	13337
**GNG4**	0.30165	2.4738	0.5196607529889980	13345

**IL1RAPL2**	0.30165	2.7589	0.5196607529889980	13344
**STAT5A**	0.30165	2.9676	0.5196607529889980	13342
**UPF3A**	0.30165	3.075	0.5196607529889980	13341
**ZRANB3**	0.30165	2.8268	0.5196607529889980	13343
**RPS6KB1**	0.30155	1.1351	0.5199476739536520	13346
**NOS1AP**	0.69852	0.56252	0.5201485440990960	13347
**SDHAF3**	0.69871	0.22329	0.5206938689066190	13348
**ADGRA1**	0.30128	0.38664	0.520722574500632	13349
**HNRNPD**	0.30123	1.4382	0.5208661089077860	13351
**SOCS3**	0.30123	1.6308	0.5208661089077860	13350
**MRGBP**	0.30113	0.91927	0.5211532099221370	13352
**SCYL3**	0.69887	0.10703	0.5211532099221370	13353
**CNPY4**	0.30107	2.2577	0.5213254911510460	13354
**DNAJC4**	0.69901	0.32407	0.5215552235296390	13355
**PLEKHA2**	0.30068	0.37417	0.5224456966919090	13356
**ADAM30**	0.30064	1.9988	0.5225606266216540	13362
**BCAS3**	0.30064	0.95444	0.5225606266216540	13363
**DNAJC19**	0.30064	2.1422	0.5225606266216540	13361
**FAM187B**	0.30064	2.8604	0.5225606266216540	13359
**GRIN1**	0.30064	3.0615	0.5225606266216540	13357
**KAT7**	0.30064	2.3253	0.5225606266216540	13360
**SEMA3C**	0.30064	2.9809	0.5225606266216540	13358
**OR1I1**	0.30042	0.0037976	0.5231928646877000	13364
**INSR**	0.69965	0.45189	0.5233940751987410	13365
**MPC1L**	0.30028	2.3944	0.5235953069021220	13366
**BTN3A2**	0.30025	0.56734	0.5236815555512910	13367
**CAMK1**	0.30018	2.567	0.523882817551328	13370
**CSF3R**	0.30018	1.863	0.523882817551328	13373
**HTR2A**	0.30018	0.63423	0.523882817551328	13376
**MYO15B**	0.30018	2.5881	0.523882817551328	13369
**NFE2**	0.30018	0.75959	0.523882817551328	13375
**RAD52**	0.30018	2.1179	0.523882817551328	13372

**RRP1**	0.30018	2.7019	0.523882817551328	13368
**SPCS3**	0.30018	0.94874	0.523882817551328	13374
**TEX13C**	0.30018	2.1906	0.523882817551328	13371
**IFIT3**	0.30014	2.5767	0.5239978339363710	13377
**TJAP1**	0.69993	0.47751	0.5241991292924910	13378
**GPS2**	0.29994	2.414	0.5245730198934370	13379
**PANX1**	0.29991	2.0116	0.524659312753022	13380
**GLT1D1**	0.70043	0.089443	0.5256375717949170	13381
**FLRT3**	0.29954	0.19817	0.5257239129112500	13383
**RNF20**	0.29954	0.64017	0.5257239129112500	13382
**STMN3**	0.2995	3.1441	0.5258390404966580	13384
**ZNF256**	0.70055	0.11869	0.5259829597804420	13385
**PKD1L2**	0.29942	1.9963	0.5260693165802360	13386
**RAP1GDS1**	0.29935	2.9009	0.5262708310390190	13387
**CIITA**	0.29926	1.0151	0.5265299524648430	13388
**ADGRD1**	0.29923	1.1439	0.526616334129526	13389
**SLC2A14**	0.29914	2.2896	0.52687550270583	13390
**NEK5**	0.70093	0.22441	0.5270771027340190	13391
**POM121**	0.29904	1.1058	0.5271635093037820	13392
**SPATA31A5**	0.29901	0.14368	0.5272499198096050	13393
**CPXM2**	0.29899	1.1066	0.5273075290006830	13394
**WDR1**	0.29896	0.76622	0.5273939460688360	13395
**RPL35**	0.29882	2.4398	0.5277972778153110	13396
**DLGAP4**	0.70136	0.072682	0.5283159734076250	13398
**ZBTB49**	0.29864	1.0652	0.5283159734076250	13397
**ARAF**	0.29862	2.0763	0.5283736150238280	13400
**HRG**	0.29862	1.9386	0.5283736150238280	13402
**OPALIN**	0.29862	0.8294	0.5283736150238280	13405
**PAM16**	0.29862	1.9979	0.5283736150238280	13401
**PDE2A**	0.29862	1.4352	0.5283736150238280	13404
**SMIM10**	0.29862	2.35	0.5283736150238280	13399
**STK17A**	0.29862	0.20168	0.5283736150238280	13406

**TMPRSS11F**	0.29862	1.8417	0.5283736150238280	13403
**PRKCQ**	0.29847	2.7284	0.528805983126241	13407
**DNAJB7**	0.70154	0.15038	0.5288348111807210	13408
**SLN**	0.70155	0.0013672	0.5288636396747010	13409
**SSTR1**	0.7018	0.347	0.5295844949715230	13410
**OPCML**	0.29803	1.0718	0.530074833786926	13411
**CYSTM1**	0.298	1.1226	0.5301613773941920	13412
**TTC32**	0.2979	0.45615	0.5304498847702810	13413
**PPP1CC**	0.29779	1.2538	0.5307672938900580	13414
**ITGAD**	0.29776	2.1181	0.530853869293774	13415
**PPM1J**	0.29769	2.466	0.531055894045218	13416
**SCGB3A2**	0.29755	0.91097	0.5314600085919730	13417
**MUL1**	0.29748	2.8308	0.5316620984130960	13418
**FAM149A**	0.29715	1.611	0.5326151002828780	13419
**SLC25A33**	0.29708	1.8889	0.5328173143680420	13420
**MED15**	0.29706	1.2721	0.5328750938225650	13421
**C1QTNF2**	0.29698	2.2593	0.5331062294342000	13422
**CLEC10A**	0.29683	1.6411	0.5335396854950530	13430
**DEFB134**	0.29683	1.8864	0.5335396854950530	13429
**ERMAP**	0.29683	2.1559	0.5335396854950530	13426
**FXYD4**	0.29683	2.4744	0.5335396854950530	13424
**NEDD4L**	0.29683	1.8894	0.5335396854950530	13428
**NEU2**	0.29683	1.5372	0.5335396854950530	13432
**NFXL1**	0.29683	2.3651	0.5335396854950530	13425
**OLFML3**	0.29683	0.27543	0.5335396854950530	13434
**PRELID2**	0.29683	1.4269	0.5335396854950530	13433
**THEM6**	0.29683	1.598	0.5335396854950530	13431
**TTC22**	0.29683	2.5767	0.5335396854950530	13423
**UTP11**	0.29683	0.19755	0.5335396854950530	13435
**ZNF432**	0.29683	1.9145	0.5335396854950530	13427
**MUC5AC**	0.29671	1.2313	0.5338865225302120	13436
**CD1D**	0.29647	1.4643	0.5345803893761990	13437

**WFS1**	0.29637	2.0179	0.5348695765217660	13438
**FAM50B**	0.29634	1.5589	0.5349563413876030	13439
**ZBTB34**	0.29633	2.2131	0.5349852639044850	13440
**QRFP**	0.2961	2.2902	0.5356506053885040	13441
**HACD2**	0.70403	0.21355	0.5360267728640440	13442
**VPS4A**	0.29582	1.7858	0.5364609065339260	13443
**DHDH**	0.29578	2.59	0.5365766925873260	13444
**MPV17L2**	0.29572	0.35246	0.5367503851570580	13445
**SIN3A**	0.70456	0.39341	0.5375611647485900	13446
**LDB3**	0.70465	0.37961	0.5378218475139530	13447
**XRCC2**	0.70471	0.26943	0.5379956563298680	13448
**AGFG1**	0.29526	2.6352	0.5380825668326370	13449
**AP1S2**	0.29526	0.99195	0.5380825668326370	13450
**CRIPAK**	0.29508	2.1578	0.538604115241909	13451
**C1QTNF9B**	0.295	0.52337	0.5388359615621090	13452
**ARHGEF10**	0.29498	2.4798	0.5388939276675930	13454
**ARHGEF5**	0.29498	1.5882	0.5388939276675930	13459
**CDKL1**	0.29498	2.179	0.5388939276675930	13455
**FAM105A**	0.29498	2.5506	0.5388939276675930	13453
**FBXO27**	0.29498	1.8856	0.5388939276675930	13456
**MAP7D2**	0.29498	1.7915	0.5388939276675930	13458
**OSGEP**	0.29498	1.4099	0.5388939276675930	13461
**PNPLA8**	0.29498	1.5807	0.5388939276675930	13460
**SPC24**	0.29498	1.7962	0.5388939276675930	13457
**TMEM183B**	0.29498	0.43422	0.5388939276675930	13463
**WBP4**	0.29498	0.47533	0.5388939276675930	13462
**COMT**	0.70506	0.29507	0.5390098653112390	13464
**MLLT6**	0.7051	0.37561	0.5391258102005050	13466
**USP4**	0.2949	1.6456	0.5391258102005050	13465
**OTUD7B**	0.70536	0.18787	0.5398796287630530	13467
**DUSP5**	0.29458	1.4191	0.5400536304529390	13468
**ZFP36L1**	0.29449	0.18894	0.5403146636515480	13469

**CCT6A**	0.29447	0.70086	0.540372676028485	13470
**SLC35E2B**	0.70553	0.041759	0.540372676028485	13471
**ZNF182**	0.29446	1.8746	0.5404016828989310	13472
**SNRPB**	0.70562	0.42308	0.5406337542350670	13473
**UBA1**	0.29434	0.18806	0.5407498008222010	13474
**CASTOR1**	0.70577	0.41734	0.5410689664955880	13475
**LOC1001299**	0.29417	2.347	0.5412430800948620	13476
**RAP1B**	0.70588	0.63068	0.5413881872957800	13477
**SRSF6**	0.29408	2.6967	0.5415042812646370	13478
**PSMD11**	0.29402	0.92221	0.5416784359039450	13479
**CMYA5**	0.7061	0.20838	0.5420267944829730	13480
**DIEXF**	0.2939	0.14657	0.5420267944829740	13481
**ACSL1**	0.2938	1.9361	0.5423171435520840	13482
**ALCAM**	0.29369	1.2093	0.5426365803434010	13486
**DDX11**	0.29369	2.017	0.5426365803434010	13484
**NSUN4**	0.29369	2.5725	0.5426365803434010	13483
**RPL18A**	0.29369	1.1925	0.5426365803434010	13487
**ZNF394**	0.29369	1.2748	0.5426365803434010	13485
**ST6GALNAC**	0.70632	0.14462	0.542665622797065	13488
**HLA-DOB**	0.29363	1.2305	0.5428108419321040	13489
**ZNF414**	0.2935	2.3026	0.5431884652724460	13490
**MAFB**	0.29348	0.28707	0.543246568045271	13491
**ZNF195**	0.29339	2.613	0.5435080532236530	13492
**TXNRD3**	0.29334	2.1512	0.543653338825456	13493
**ANKRD12**	0.2933	1.095	0.5437695755696470	13494
**CCDC168**	0.29318	2.4101	0.5441183298964490	13498
**CXCR6**	0.29318	2.785	0.5441183298964490	13496
**LAMC1**	0.29318	3.0493	0.5441183298964490	13495
**TMCO6**	0.29318	2.3801	0.5441183298964490	13499
**TPSD1**	0.29318	2.6318	0.5441183298964490	13497
**CTNNAL1**	0.29301	0.21919	0.5446125118531920	13500
**RMND5A**	0.29297	1.8252	0.5447288092880150	13501

**PNPLA4**	0.29295	1.8148	0.5447869607682790	13502
**MXD1**	0.29284	1.8823	0.5451068268495830	13503
**SLC35F3**	0.29273	1.5642	0.5454267487131990	13504
**TMED10**	0.7073	0.099722	0.5455140098186400	13505
**C3orf18**	0.29259	0.37149	0.54583400275345	13506
**PLCB4**	0.29258	1.1774	0.5458630957914180	13507
**AQR**	0.29255	1.4452	0.5459503776776680	13508
**NDUFA2**	0.2924	1.4414	0.5463868495203190	13509
**CXXC1**	0.29235	2.0147	0.5465323632649470	13510
**AQP10**	0.29227	2.8368	0.5467652093310870	13511
**BOK**	0.29227	2.4118	0.5467652093310870	13513
**C2orf83**	0.29227	1.6208	0.5467652093310870	13516
**FBXO6**	0.29227	0.024516	0.5467652093310870	13519
**ODF2**	0.29227	1.7638	0.5467652093310870	13514
**RBM25**	0.29227	0.41387	0.5467652093310870	13518
**SHTN1**	0.29227	2.767	0.5467652093310870	13512
**TCEAL5**	0.29227	1.6555	0.5467652093310870	13515
**ZBP1**	0.29227	1.5138	0.5467652093310870	13517
**SPSB2**	0.29225	2.6807	0.5468234254793490	13520
**BCL2L15**	0.29218	3.0957	0.5470271965952340	13521
**CIAPIN1**	0.29188	1.2688	0.547900758846967	13526
**CTPS1**	0.29188	2.8319	0.547900758846967	13522
**FOXI2**	0.29188	0.28515	0.547900758846967	13527
**HMGXB4**	0.29188	2.5082	0.547900758846967	13523
**PCYT1A**	0.29188	2.2586	0.547900758846967	13525
**PLCE1**	0.29188	2.3796	0.547900758846967	13524
**ACVR1**	0.2914	1.736	0.5492993289833730	13528
**SLC39A1**	0.29136	2.2771	0.5494159249702960	13529
**ASNSD1**	0.29128	2.4198	0.5496491393555700	13532
**C12orf75**	0.29128	1.1178	0.5496491393555700	13533
**SMARCE1**	0.29128	3.2149	0.5496491393555700	13530
**TMEM89**	0.29128	2.7731	0.5496491393555700	13531

**GUSB**	0.70874	0.23417	0.5497074476227900	13534
**CCL17**	0.2912	1.2968	0.5498823836397030	13535
**XRCC1**	0.70894	0.70706	0.5502906331423100	13536
**OR51D1**	0.29101	2.1152	0.5504364587625640	13537
**ABAT**	0.29093	1.2938	0.5506698041057390	13543
**ARFIP2**	0.29093	1.7582	0.5506698041057390	13541
**AURKC**	0.29093	1.4154	0.5506698041057390	13542
**CHST2**	0.29093	2.4909	0.5506698041057390	13538
**GATAD2B**	0.29093	2.2028	0.5506698041057390	13539
**OOSP2**	0.29093	2.0249	0.5506698041057390	13540
**UQCRH**	0.29093	0.66386	0.5506698041057390	13544
**IKZF2**	0.29092	1.2718	0.5506989743817520	13545
**NLRC5**	0.29069	2.7897	0.5513700201451990	13546
**TNFSF8**	0.29065	2.0156	0.5514867490967100	13547
**PALLD**	0.70958	0.87357	0.5521580864824760	13548
**BMP8A**	0.29027	0.94301	0.552596049303259	13549
**ZBTB44**	0.29022	2.293	0.5527420604636250	13550
**RORA**	0.7098	0.43885	0.5528004682273110	13551
**HCRT**	0.29017	2.5366	0.5528880834090650	13552
**CC2D1B**	0.29015	3.0717	0.5529464958881860	13553
**IZUMO3**	0.29015	0.43751	0.5529464958881860	13555
**PCDHB12**	0.29015	2.797	0.5529464958881860	13554
**STARD8**	0.29008	0.81332	0.5531509544252620	13556
**DEFB116**	0.29005	1.5145	0.5532385865912530	13557
**A2ML1**	0.28977	1.3454	0.5540566918377440	13561
**ASPN**	0.28977	2.1954	0.5540566918377440	13559
**ENTPD6**	0.28977	2.4001	0.5540566918377440	13564
**LRRFIP1**	0.28977	1.3876	0.5540566918377440	13565
**PLCXD2**	0.28977	0.12188	0.5540566918377440	13568
**PLPBP**	0.28977	1.4148	0.5540566918377440	13563
**PPP2R2B**	0.28977	2.2737	0.5540566918377440	13560
**PRKCE**	0.28977	0.81989	0.5540566918377440	13566

**PSMD10**	0.28977	2.4036	0.5540566918377440	13558
**SMIM11B**	0.28977	1.578	0.5540566918377440	13562
**VIPR1**	0.28977	0.40969	0.5540566918377440	13567
**MED31**	0.28954	1.4187	0.5547289843613300	13569
**CASK**	0.28947	2.2472	0.5549336448914510	13570
**PIGM**	0.28935	1.74	0.5552845456063390	13571
**CBX8**	0.28921	2.462	0.55569401621175	13572
**APOA1**	0.28917	0.44317	0.555811024925494	13574
**TICRR**	0.28917	2.0977	0.555811024925494	13573
**NDUFAF2**	0.28916	2.1884	0.5558402782929180	13575
**TRAF3IP3**	0.28906	1.6607	0.5561328381360350	13576
**CD9**	0.28905	1.9163	0.5561620967381260	13577
**ADAM11**	0.28899	2.3025	0.5563376583506510	13578
**AGBL5**	0.28896	1.7844	0.5564254455872970	13579
**LUC7L**	0.28891	1.4232	0.5565717671786560	13580
**EPN1**	0.28882	2.4838	0.5568351760779770	13581
**SDCBP2**	0.28877	2.3906	0.5569815310504230	13582
**ESCO1**	0.28855	1.8617	0.5576256347434250	13583
**KLF5**	0.71146	0.44893	0.5576549176795060	13584
**IKBKAP**	0.28846	1.4349	0.5578891983865750	13585
**CCDC42**	0.28832	2.721	0.558299263328636	13586
**HIST2H3A**	0.28832	0.76564	0.558299263328636	13590
**NFKB1**	0.28832	2.5099	0.558299263328636	13587
**SLK**	0.28832	2.0167	0.558299263328636	13589
**UPRT**	0.28832	2.4118	0.558299263328636	13588
**FOXJ2**	0.28819	0.968	0.5586801219958330	13591
**CDKN2A**	0.71182	0.052976	0.5587094221725980	13592
**RPLP1**	0.28816	1.1771	0.5587680239651570	13593
**AP1AR**	0.28814	1.5541	0.5588266276766940	13600
**C1orf100**	0.28814	2.1909	0.5588266276766940	13597
**C6orf201**	0.28814	0.90999	0.5588266276766940	13602
**CRAT**	0.28814	0.70666	0.5588266276766940	13603

**DNTT**	0.28814	2.202	0.5588266276766940	13596
**KLHL20**	0.28814	1.9464	0.5588266276766940	13598
**LCN15**	0.28814	2.5979	0.5588266276766940	13594
**LHX3**	0.28814	2.4799	0.5588266276766940	13595
**MS4A15**	0.28814	1.3677	0.5588266276766940	13601
**PIP5KL1**	0.28814	1.7035	0.5588266276766940	13599
**NMNAT1**	0.28807	1.8295	0.5590317557837540	13604
**SLC35C1**	0.288	0.32789	0.5592369074163320	13605
**MARK2**	0.28789	2.7706	0.5595593361181480	13606
**PRKG2**	0.7125	0.13299	0.5607029616258930	13607
**OR10H2**	0.28746	2.8148	0.5608202979992410	13608
**SMIM4**	0.28746	2.5585	0.5608202979992410	13609
**ZNF837**	0.28731	0.89298	0.5612603781933310	13610
**CEACAM19**	0.71311	0.28868	0.5624931816091020	13611
**PUM1**	0.28688	0.68083	0.5625225444859820	13612
**TRIM25**	0.28657	2.712	0.5634330344372710	13613
**TRIB1**	0.28649	0.42216	0.5636680753975300	13614
**CCDC88B**	0.2864	2.2255	0.5639325337053680	13615
**HES5**	0.2864	1.539	0.5639325337053680	13617
**SYNDIG1L**	0.2864	1.169	0.5639325337053680	13618
**TBX21**	0.2864	1.8221	0.5639325337053680	13616
**ZNF41**	0.2864	0.091647	0.5639325337053680	13619
**CA11**	0.28627	0.74675	0.5643145986870110	13620
**IFT22**	0.71396	0.19538	0.5649907617049880	13621
**HOXD9**	0.28598	0.72615	0.5651671945357260	13622
**RAB1A**	0.28591	1.1178	0.5653730550766140	13623
**LRRC37A3**	0.28582	1.4703	0.5656377681272860	13624
**DDX43**	0.2857	1.1267	0.5659907805322630	13625
**ARHGEF10L**	0.28554	2.3394	0.5664615734911630	13626
**FAAP20**	0.71453	0.04066	0.566667584892165	13629
**IBSP**	0.28547	2.9401	0.566667584892165	13628
**RANBP1**	0.28547	3.0421	0.566667584892165	13627

**ABCD4**	0.28545	2.6796	0.5667264497096680	13630
**TNFRSF8**	0.28534	2.3087	0.567050241318234	13631
**GSK3A**	0.28532	2.0851	0.567109118906568	13632
**GNAT2**	0.71472	0.12494	0.5672268799815740	13633
**SUMF2**	0.28527	2.8217	0.5672563214793910	13634
**TBC1D24**	0.28522	0.85371	0.5674035363449580	13635
**OLFML1**	0.71481	0.14381	0.5674918711669970	13636
**POLR2J2**	0.71485	0.018597	0.5676096578185490	13637
**PHLDB2**	0.71487	0.21832	0.567668554097437	13638
**C1QTNF8**	0.28512	0.74953	0.5676980029753060	13639
**CNIH4**	0.28508	2.0382	0.5678158034105980	13640
**BMP3**	0.28505	2.3043	0.5679041589082470	13641
**MRPS9**	0.28491	2.0695	0.5683165432056730	13642
**ABHD4**	0.28482	2.0087	0.5685816984381260	13644
**ACRBP**	0.28482	1.8184	0.5685816984381260	13645
**GFAP**	0.28482	2.7502	0.5685816984381260	13643
**GNL3**	0.28482	0.075324	0.5685816984381260	13647
**PLXNB1**	0.28482	0.57977	0.5685816984381260	13646
**AP4S1**	0.71523	0.40952	0.5687290241746750	13648
**REEP2**	0.28467	1.873	0.5690237126889260	13649
**EVA1C**	0.28463	2.3262	0.5691416019307990	13650
**SLC25A2**	0.71542	0.46164	0.5692889746075030	13651
**CXorf36**	0.28449	2.3386	0.5695542765897380	13653
**RPS6KA1**	0.28449	2.9583	0.5695542765897380	13652
**SLC9A1**	0.28449	1.3826	0.5695542765897380	13654
**RABGGTB**	0.28427	1.8492	0.5702029614014140	13655
**SEPT7**	0.71577	0.25862	0.5703209298692620	13656
**CFAP70**	0.28411	1.2515	0.570674882908547	13657
**ZNF280C**	0.7159	0.2319	0.570704382222051	13658
**ATG7**	0.28402	1.5379	0.5709403946128120	13659
**TPTE**	0.284	0.030641	0.5709994026794810	13660
**BLNK**	0.71603	0.059926	0.5710879185075980	13661

**SPG11**	0.71617	0.27309	0.571501051556869	13662
**ETS1**	0.2838	2.7745	0.5715895927570870	13663
**ARMC9**	0.71621	0.21035	0.5716191074862720	13665
**DIMT1**	0.71621	0.24366	0.5716191074862720	13664
**NKX2-1**	0.28378	2.102	0.571648622713414	13666
**MAPK13**	0.28375	0.84139	0.571737171383026	13667
**NPR 2.00**	0.28365	1.6567	0.5720323659999440	13668
**IPCEF1**	0.28365	2.2634	0.5720323659999440	13669
**UHRF2**	0.71642	0.35574	0.5722390318932850	13670
**GVQW1**	0.28347	2.6447	0.5725638419749130	13672
**TOR4A**	0.28347	2.6447	0.5725638419749130	13671
**PRRG4**	0.28329	1.9276	0.5730954797302370	13673
**AGO1**	0.28327	2.6017	0.5731545605891880	13674
**SEMA6A**	0.28324	1.1864	0.5732431856290380	13675
**MRPL50**	0.2832	2.1566	0.5733613593532890	13676
**PDZRN4**	0.2832	0.15861	0.5733613593532890	13677
**AK2**	0.28315	0.88949	0.5735090877696030	13688
**C16orf86**	0.28315	1.6291	0.5735090877696030	13685
**DPM1**	0.28315	1.7184	0.5735090877696030	13684
**GJA9**	0.28315	1.7711	0.5735090877696030	13682
**GSPT2**	0.28315	1.6222	0.5735090877696030	13686
**IPO7**	0.28315	1.3177	0.5735090877696030	13687
**MAGED4**	0.28315	2.065	0.5735090877696030	13680
**PIGA**	0.28315	2.0798	0.5735090877696030	13679
**RHBDL1**	0.28315	0.2542	0.5735090877696030	13690
**SLC39A12**	0.28315	0.52257	0.5735090877696030	13689
**SNCG**	0.28315	1.8224	0.5735090877696030	13681
**SOWAHD**	0.28315	2.6022	0.5735090877696030	13678
**SPACA5**	0.28315	1.7525	0.5735090877696030	13683
**PPP1R1B**	0.28308	1.501	0.5737159285825530	13691
**CCDC17**	0.28296	2.5028	0.5740705699540750	13692
**CWC22**	0.28287	1.9443	0.5743365983705760	13693

**AMBP**	0.71714	0.1235	0.5743661595923860	13697
**ATP8B1**	0.28286	1.721	0.5743661595923860	13695
**PDZD8**	0.28286	3.1606	0.5743661595923860	13694
**PPP1R8**	0.28286	1.6799	0.5743661595923860	13696
**ZSWIM1**	0.28279	1.9277	0.5745731022015	13698
**GGPS1**	0.28267	1.4058	0.5749279182224100	13699
**TRPM7**	0.28262	1.4214	0.5750757795935510	13700
**SLC25A34**	0.28259	0.26889	0.5751645024514180	13701
**CSDC2**	0.28243	2.7259	0.5756377675246070	13702
**CMBL**	0.28208	0.59144	0.5766734847373050	13705
**EPRS**	0.28208	1.305	0.5766734847373050	13704
**EPS8L2**	0.28208	2.1686	0.5766734847373050	13703
**S100A7L2**	0.28191	0.19326	0.5771767706057910	13706
**MYH6**	0.71815	0.61297	0.5773544358168550	13707
**LRRC14B**	0.28183	2.5814	0.5774136616036630	13708
**ACO2**	0.28172	2.3296	0.5777394396456790	13709
**PCDH1**	0.28171	0.093119	0.5777690588715140	13710
**WDFY3**	0.28169	2.8279	0.57782829884389	13711
**UBE2D4**	0.28159	2.2787	0.5781245291311440	13712
**ACOT11**	0.28151	2.2653	0.5783615498922160	13713
**BST1**	0.28148	1.3858	0.5784504410543780	13715
**GABRA1**	0.28148	0.77954	0.5784504410543780	13719
**LRP2BP**	0.28148	2.5807	0.5784504410543780	13714
**SLC25A52**	0.28148	0.87717	0.5784504410543780	13718
**SLC35C2**	0.28148	0.92605	0.5784504410543780	13717
**TRIM35**	0.28148	0.99304	0.5784504410543780	13716
**MCM6**	0.71861	0.55152	0.5787171419713500	13721
**PHRF1**	0.28139	2.6148	0.5787171419713500	13720
**LOC157562**	0.28128	1.4329	0.5790431656723180	13722
**SET**	0.28127	1.3528	0.5790728072422430	13723
**LOC100289[illegible]**	0.28125	1.3967	0.5791320919085330	13724
**BCL10**	0.28105	3.1262	0.5797250505835870	13725

**KCNQ3**	0.71895	0.3527	0.5797250505835870	13728
**TP53BP1**	0.28105	2.9351	0.5797250505835870	13727
**USP1**	0.28105	2.9546	0.5797250505835870	13726
**COL5A3**	0.28096	2.0328	0.5799919484896860	13729
**LANCL3**	0.28094	1.8125	0.5800512647460010	13730
**EDN1**	0.28077	2.0934	0.5805555353630470	13731
**HSPB2**	0.71935	0.4875	0.5809115799903210	13732
**UNC119B**	0.28052	2.4597	0.5812973781248750	13733
**KIF26B**	0.28044	2.1046	0.5815348353694910	13734
**VHL**	0.7197	0.073069	0.5819504644742610	13735
**APP**	0.71976	0.73257	0.582128622008676	13736
**LSM 7.00**	0.28021	2.5389	0.5822177077048550	13739
**DNASE1**	0.28021	2.0693	0.5822177077048550	13737
**LSMEM1**	0.28021	1.6121	0.5822177077048550	13742
**MAD2L2**	0.28021	0.069357	0.5822177077048550	13744
**PCDHGB5**	0.28021	1.8818	0.5822177077048550	13741
**SUMO4**	0.28021	0.11303	0.5822177077048550	13743
**TNNT2**	0.28021	2.0933	0.5822177077048550	13738
**WWP2**	0.28021	1.9064	0.5822177077048550	13740
**KCNMB1**	0.2801	1.955	0.5825443948008610	13745
**KCNJ12**	0.28	0.50237	0.5828414370296450	13746
**MYOM3**	0.27998	2.6016	0.5829008516462710	13747
**CASP7**	0.27996	3.2179	0.5829602683206750	13748
**CD34**	0.27996	3.0392	0.5829602683206750	13749
**PPP2R2D**	0.27996	2.7215	0.5829602683206750	13750
**RAPGEF1**	0.27996	2.4679	0.5829602683206750	13751
**PC**	0.27983	1.9238	0.5833465268869830	13752
**SERPINB9**	0.72027	0.15818	0.5836437080931370	13753
**AOC3**	0.2797	2.497	0.583732872506136	13754
**PCDHGC4**	0.7204	0.018253	0.5840301207419760	13755
**MED9**	0.27931	2.8243	0.5848924326506560	13756
**OR5AP2**	0.27921	0.63713	0.5851898824238130	13757

**LIX1**	0.27917	1.1382	0.585308876830322	13758
**SLC6A13**	0.27915	0.96124	0.5853683771415490	13759
**MBD5**	0.27909	2.0702	0.5855468905113770	13760
**ASIC5**	0.27903	2.1638	0.5857254225428870	13761
**ZNF501**	0.72105	0.030474	0.5859634942974310	13762
**VASN**	0.72106	0.043947	0.5859932556017920	13763
**SAXO1**	0.72112	0.41736	0.5861718343294880	13764
**AP5S1**	0.27886	2.3512	0.5862313647260870	13767
**CDK6**	0.27886	2.5784	0.5862313647260870	13766
**F2RL2**	0.27886	2.1384	0.5862313647260870	13768
**MYO6**	0.27886	3.0621	0.5862313647260870	13765
**RABL3**	0.27886	0.75331	0.5862313647260870	13769
**ACER3**	0.27879	3.0576	0.5864397374776930	13770
**BECN1**	0.27879	0.3475	0.5864397374776930	13776
**C14orf159**	0.27879	0.35971	0.5864397374776930	13775
**DND1**	0.27879	1.9565	0.5864397374776930	13774
**KIRREL3**	0.27879	2.7444	0.5864397374776930	13772
**KRT39**	0.27879	2.7532	0.5864397374776930	13771
**NPIPA3**	0.27879	2.6144	0.5864397374776930	13773
**ELOVL7**	0.27862	2.2457	0.5869458916406830	13777
**STN 1.00**	0.2784	2.7428	0.5876011379893900	13778
**RPS6KB2**	0.27834	2.2524	0.5877798853211410	13779
**BMP2K**	0.72182	0.4203	0.588256636721794	13780
**TAX1BP3**	0.27806	1.7868	0.5886142880318440	13781
**STAU2**	0.27803	0.73715	0.5887037126232840	13782
**HDAC4**	0.27784	2.5711	0.5892701777491180	13784
**LRRC8D**	0.27784	2.6974	0.5892701777491180	13783
**PDE9A**	0.27784	2.3143	0.5892701777491180	13785
**BCAT2**	0.72223	0.28648	0.5894789230913510	13786
**MPO**	0.72227	0.090351	0.5895982176771750	13788
**TAPT1**	0.27773	0.98082	0.5895982176771750	13787
**DNAJB5**	0.27769	1.494	0.5897175206543190	13789

**AFP**	0.27754	2.64	0.5901649816029640	13790
**NAIP**	0.27748	1.604	0.5903439990686160	13791
**ZMYM2**	0.27742	0.11607	0.5905230354552640	13792
**RP1L1**	0.72266	0.31176	0.5907617800869550	13793
**AHI1**	0.27722	1.9929	0.5911199601874880	13794
**POTEM**	0.27719	1.7612	0.5912095170615970	13795
**ABCC10**	0.27715	1.6418	0.5913289336037800	13799
**CD40LG**	0.27715	1.2715	0.5913289336037800	13801
**GPR34**	0.27715	1.1271	0.5913289336037800	13802
**LRRC19**	0.27715	1.3963	0.5913289336037800	13800
**NDUFA13**	0.27715	3.1481	0.5913289336037800	13796
**P2RY8**	0.27715	2.0261	0.5913289336037800	13798
**USP33**	0.27715	2.3289	0.5913289336037800	13797
**THADA**	0.72288	0.53762	0.5914185015445270	13803
**IFT80**	0.7229	0.26939	0.5914782161409930	13804
**ADGRD2**	0.277	0.68316	0.5917768207685260	13805
**DUSP15**	0.27697	2.8113	0.5918664124458880	13808
**OR52N1**	0.27697	2.8843	0.5918664124458880	13807
**SMARCAL1**	0.27697	2.9215	0.5918664124458880	13806
**IL20**	0.27683	1.7537	0.5922845697786700	13809
**NDRG2**	0.72321	0.31931	0.592404062323687	13810
**MSANTD2**	0.27664	0.59121	0.5928522347315220	13811
**TMEM87A**	0.27663	2.5945	0.5928821171254280	13812
**MCRIP1**	0.27655	2.871	0.5931211953399930	13813
**CACNA2D4**	0.27648	0.3197	0.5933304165911320	13814
**CDH6**	0.27631	0.72415	0.5938386335178250	13815
**OPN5**	0.72374	0.22276	0.5939881382732520	13816
**PGAP3**	0.72377	0.07375	0.5940778474995200	13817
**RAB32**	0.72384	0.059357	0.5942871876234170	13818
**MPC1**	0.27612	1.8636	0.5944068222449170	13819
**AQP11**	0.27609	0.070002	0.5944965537942950	13820
**ISPD**	0.27597	0.69614	0.5948555278743080	13826

**MIS18BP1**	0.27597	2.3545	0.5948555278743080	13822
**MSANTD3-T**	0.27597	1.227	0.5948555278743080	13825
**NDUFAB1**	0.27597	2.1124	0.5948555278743080	13824
**SLC13A4**	0.27597	2.7129	0.5948555278743080	13821
**TSPYL1**	0.27597	2.1958	0.5948555278743080	13823
**ZSCAN5B**	0.27597	0.21037	0.5948555278743080	13827
**FAM96A**	0.27593	2.8568	0.5949752029351300	13828
**HOXB5**	0.27593	1.4554	0.5949752029351300	13829
**HAGHL**	0.72409	0.42788	0.5950350436610900	13830
**ASPDH**	0.27577	1.2841	0.5954539884272390	13838
**DIS3**	0.27577	1.2835	0.5954539884272390	13839
**DSG2**	0.27577	2.5124	0.5954539884272390	13832
**FAM170A**	0.27577	1.994	0.5954539884272390	13835
**FANCE**	0.27577	1.3409	0.5954539884272390	13837
**LYPLAL1**	0.27577	2.2261	0.5954539884272390	13833
**MECP2**	0.27577	2.0156	0.5954539884272390	13834
**RPS9**	0.27577	1.2165	0.5954539884272390	13840
**STOML1**	0.27577	2.7279	0.5954539884272390	13831
**TIGD4**	0.27577	1.7866	0.5954539884272390	13836
**CLDN4**	0.27567	0.9269	0.5957532986840010	13841
**CYB5R1**	0.72436	0.21182	0.5958431021682950	13842
**LGI4**	0.72439	0.59498	0.5959329104581450	13843
**GPATCH2**	0.27557	0.16736	0.5960526623220610	13844
**RHOC**	0.27548	2.3799	0.596322135275248	13845
**GABRA2**	0.7246	0.32359	0.5965617031467390	13846
**LOC646588**	0.27539	2.7515	0.5965916515379290	13847
**PTPN2**	0.27509	2.3015	0.5974903523014280	13848
**C1GALT1**	0.27506	2.7005	0.5975802489130190	13850
**FAM89B**	0.27506	3.1395	0.5975802489130190	13849
**SDHB**	0.27506	0.20085	0.5975802489130190	13851
**STATH**	0.275	0.49287	0.5977600566261490	13852
**ZNF133**	0.27485	0.29713	0.5982096604918200	13853

**RAB25**	0.27479	1.3835	0.5983895358974160	13854
**PRSS23**	0.27476	1.3386	0.5984794808607750	13855
**CEP135**	0.72548	0.33824	0.5991992149860540	13856
**LTBP4**	0.72551	0.51644	0.5992892035727700	13857
**TCL1B**	0.27443	1.8112	0.5994691953073430	13858
**DDX20**	0.27438	1.4864	0.5996192032562290	13864
**FAM157A**	0.27438	1.7689	0.5996192032562290	13863
**IGFBP5**	0.27438	2.4384	0.5996192032562290	13859
**SLC5A4**	0.27438	0.48325	0.5996192032562290	13866
**SMN2**	0.27438	1.9256	0.5996192032562290	13862
**TAF9B**	0.27438	2.0332	0.5996192032562290	13861
**TAP2**	0.27438	2.1037	0.5996192032562290	13860
**WFIKKN2**	0.27438	0.53677	0.5996192032562290	13865
**KRTAP5-1**	0.27431	0.29936	0.5998292370561220	13867
**TUSC2**	0.7258	0.37282	0.6001593436453420	13868
**PLAC1**	0.72581	0.12812	0.6001893565779520	13869
**BHLHE23**	0.27418	0.6341	0.6002193700512120	13870
**IGF2BP3**	0.27414	2.1132	0.600339429351663	13871
**AGAP4**	0.27402	2.3983	0.6006996591895140	13872
**TRAF5**	0.27385	1.7352	0.6012101182772080	13873
**IFRD2**	0.27364	1.757	0.6018409017611700	13874
**TNFSF14**	0.72636	0.08317	0.6018409017611700	13875
**MTRF1**	0.72654	0.21996	0.6023817639704030	13876
**INPP1**	0.27341	0.54483	0.6025320347445130	13877
**LYRM2**	0.2734	2.8823	0.6025620905320170	13878
**OPTC**	0.2734	2.0725	0.6025620905320170	13879
**ANKRD18B**	0.27339	2.0111	0.6025921468638590	13881
**CT83**	0.27339	0.021478	0.6025921468638590	13882
**GCC1**	0.27339	2.6466	0.6025921468638590	13880
**PCGF3**	0.27322	0.039252	0.6031031878341570	13883
**COL4A1**	0.27306	1.642	0.6035843114946710	13884
**C19orf44**	0.27293	1.3412	0.6039753273683750	13885

**CMC2**	0.27291	2.514	0.6040354918522190	13887
**ERCC6-PGE**	0.27291	1.6023	0.6040354918522190	13891
**GPRC5C**	0.27291	2.5319	0.6040354918522190	13886
**HADH**	0.27291	1.4064	0.6040354918522190	13892
**KLHL18**	0.27291	0.60504	0.6040354918522190	13893
**L3MBTL3**	0.27291	2.394	0.6040354918522190	13888
**MORN5**	0.27291	2.0441	0.6040354918522190	13889
**OTOF**	0.27291	1.7468	0.6040354918522190	13890
**EFCC1**	0.27289	2.1676	0.6040956585226190	13894
**ARHGAP28**	0.72725	0.30724	0.6045168864706370	13895
**C21orf62**	0.27265	0.46393	0.6048178292559780	13896
**VMA21**	0.72742	0.55053	0.6050285218008630	13897
**TMEM151B**	0.2725	1.8447	0.6052693461734000	13898
**ZC3H18**	0.27247	1.9703	0.6053596643632640	13899
**KCTD21**	0.27243	0.76625	0.605480096298649	13902
**RNF24**	0.27243	3.4147	0.605480096298649	13900
**WDR24**	0.27243	2.9765	0.605480096298649	13901
**TLE1**	0.27216	2.6808	0.606293241730915	13903
**C19orf68**	0.72799	0.18039	0.6067451624413830	13904
**CALN1**	0.27196	1.2667	0.6068958302150670	13905
**PKNOX2**	0.72805	0.16782	0.6069259654230120	13906
**KCNK2**	0.27194	0.86391	0.6069561011821390	13907
**CDC40**	0.2718	1.4313	0.6073780597104960	13908
**ARL4A**	0.27175	2.2346	0.607528785393095	13909
**GRSF1**	0.27164	2.3259	0.607860430490823	13910
**SPATA2L**	0.27156	1.7724	0.6081016689237860	13911
**PSORS1C1**	0.72848	0.43908	0.6082223014116470	13912
**C3orf52**	0.27151	1.7883	0.6082524609165640	13917
**CTU1**	0.27151	1.6988	0.6082524609165640	13918
**KIAA0232**	0.27151	1.0122	0.6082524609165640	13919
**KLHL8**	0.27151	2.3778	0.6082524609165640	13913
**OR10G7**	0.27151	1.8413	0.6082524609165640	13916

**SLCO1B7**	0.27151	2.3235	0.6082524609165640	13914
**TSPAN13**	0.27151	2.3235	0.6082524609165640	13915
**PHF2**	0.27146	0.66278	0.6084032667412510	13920
**C1QL3**	0.27138	1.7937	0.6086445848460180	13923
**CCDC18**	0.27138	0.1136	0.6086445848460180	13924
**GGA1**	0.27138	3.028	0.6086445848460180	13921
**USP17L17**	0.27138	2.6146	0.6086445848460180	13922
**FSTL1**	0.27124	0.40463	0.6090669768442670	13925
**IRAK3**	0.2712	2.5201	0.6091876802310030	13926
**MRPL34**	0.27114	0.30425	0.6093687519548130	13927
**ACAT2**	0.27102	2.6138	0.6097309553577160	13928
**FTH1P18**	0.27084	1.8774	0.6102744105056180	13929
**CELA2A**	0.72924	0.80065	0.6105160039846910	13930
**LYZ**	0.27072	2.6181	0.6106368140875540	13931
**ANO5**	0.27069	2.3046	0.6107274275136820	13932
**LMF1**	0.27061	1.6336	0.6109690878372870	13933
**ZNF391**	0.27052	0.40552	0.611240998358298	13934
**CDK16**	0.27049	1.8227	0.6113316452413450	13937
**SCEL**	0.27049	2.264	0.6113316452413450	13936
**SH2D1B**	0.27049	2.318	0.6113316452413450	13935
**KMT2C**	0.27024	2.3263	0.6120872314164920	13938
**CRB3**	0.27007	0.50844	0.6126012296669060	13939
**LOC1019284**	0.26985	2.6888	0.6132666442330780	13941
**RETNLB**	0.26985	2.9692	0.6132666442330780	13940
**BVES**	0.26983	1.9812	0.6133271499289550	13948
**CDH8**	0.26983	2.5349	0.6133271499289550	13943
**DBX1**	0.26983	2.1618	0.6133271499289550	13947
**FAN1**	0.26983	0.64926	0.6133271499289550	13952
**FCGR3B**	0.26983	1.8606	0.6133271499289550	13949
**GIMAP6**	0.26983	2.3573	0.6133271499289550	13945
**GPR171**	0.26983	0.051508	0.6133271499289550	13953
**HELZ**	0.26983	2.7114	0.6133271499289550	13942

**INSRR**	0.26983	1.632	0.6133271499289550	13951
**ITGA1**	0.26983	2.2326	0.6133271499289550	13946
**POLDIP3**	0.26983	2.5061	0.6133271499289550	13944
**PRF1**	0.26983	1.6367	0.6133271499289550	13950
**CRX**	0.26978	2.4496	0.6134784239933230	13954
**IRAK2**	0.26976	2.6589	0.6135389375499590	13955
**FERMT1**	0.73025	0.21461	0.6135691951708040	13956
**OR2Y1**	0.26969	2.611	0.6137507526943250	13957
**CD22**	0.73039	0.41897	0.6139928608684760	13958
**LOC284898**	0.26947	2.5554	0.6144166368024640	13959
**TSR3**	0.26935	2.9008	0.6147799611576280	13960
**KLK2**	0.73076	0.23795	0.6151130797886920	13961
**TREML4**	0.73085	0.33083	0.6153856821739330	13962
**ATM**	0.2691	3.0088	0.6155371477047520	13964
**CDC25C**	0.2691	3.1865	0.6155371477047520	13963
**DCLRE1B**	0.2691	2.8448	0.6155371477047520	13965
**GIMAP5**	0.2691	2.7023	0.6155371477047520	13968
**MYO18B**	0.2691	2.8	0.6155371477047520	13967
**RALB**	0.2691	2.096	0.6155371477047520	13969
**SSX5**	0.2691	2.8128	0.6155371477047520	13966
**TEX15**	0.26894	0.93464	0.6160219323394310	13970
**CDCA3**	0.26891	2.6819	0.6161128455748080	13971
**DNAJB14**	0.26891	0.88788	0.6161128455748080	13972
**DPCD**	0.26891	0.66654	0.6161128455748080	13973
**S100A11**	0.26874	0.68049	0.616628116807909	13974
**IFNA14**	0.26864	2.271	0.6169312940194600	13975
**RARG**	0.26857	2.8315	0.6171435518112100	13976
**FOXO6**	0.26855	1.4133	0.6172042020014490	13977
**SOCS7**	0.2685	2.1341	0.6173558374111080	13978
**SPOCK1**	0.26848	1.8952	0.617416495549629	13979
**PLG**	0.73165	0.43876	0.6178108288432640	13980
**CSH1**	0.26819	2.6466	0.6182962940532670	13981

**CENPO**	0.26818	1.2015	0.6183266404665570	13982
**RALGAPA2**	0.26817	0.3816	0.6183569874492810	13983
**ZNF782**	0.26815	3.2662	0.6184176831232230	13984
**PGRMC2**	0.26813	2.4768	0.6184783810754900	13985
**PPP1R12C**	0.73188	0.14585	0.6185087309061170	13986
**SMG8**	0.26809	2.4948	0.6185997838165700	13987
**TMEM95**	0.26806	2.0944	0.618690841855916	13988
**ALK**	0.26804	1.4994	0.6187515500655230	13989
**AKR1E2**	0.26802	1.6973	0.618812260555625	13995
**CCDC184**	0.26802	2.3858	0.618812260555625	13991
**PAN3**	0.26802	1.067	0.618812260555625	13999
**PITPNM1**	0.26802	1.691	0.618812260555625	13996
**PPM1L**	0.26802	0.5301	0.618812260555625	14001
**PRR16**	0.26802	0.93696	0.618812260555625	14000
**RAB43**	0.26802	1.538	0.618812260555625	13997
**ST8SIA1**	0.26802	2.0261	0.618812260555625	13993
**TAS2R42**	0.26802	1.4007	0.618812260555625	13998
**TMSB15B**	0.26802	2.5323	0.618812260555625	13990
**VASH2**	0.26802	2.1238	0.618812260555625	13992
**ZNF835**	0.26802	1.877	0.618812260555625	13994
**BNIP2**	0.26798	0.010639	0.6189336883788940	14002
**GGTLC1**	0.26792	2.7339	0.6191158472273920	14003
**DENR**	0.26783	0.72543	0.6193891240268600	14004
**ECHS1**	0.26777	1.3162	0.6195713342598990	14005
**GSC2**	0.26763	2.9682	0.619996571493073	14006
**SH2D1A**	0.26739	2.0128	0.6207258105841530	14007
**ZNF430**	0.26735	1.7385	0.6208473825213930	14008
**OR9Q1**	0.2673	2.8894	0.6209993603480350	14009
**SSBP3**	0.26727	0.82121	0.6210905539291220	14010
**ANAPC5**	0.26711	2.7482	0.6215770069667310	14013
**CCNA2**	0.26711	2.9863	0.6215770069667310	14012
**FAM151A**	0.26711	2.7063	0.6215770069667310	14014

**TRPM5**	0.26711	3.0843	0.6215770069667310	14011
**SLC23A3**	0.26661	0.56899	0.623098121959975	14015
**MARCKSL1**	0.26653	0.60908	0.6233416341151810	14016
**FAM72B**	0.26645	2.9861	0.6235851832391540	14017
**NUGGC**	0.73357	0.36196	0.6236460762995250	14018
**CHD2**	0.73362	0.42171	0.6237983190687580	14019
**PSTPIP2**	0.26636	0.89662	0.6238592202248260	14020
**SMYD3**	0.26633	1.4515	0.6239505762977280	14021
**CRYBA1**	0.26632	0.55884	0.6239810294792430	14024
**HECTD1**	0.26632	1.9561	0.6239810294792430	14023
**HNRNPA0**	0.26632	2.3261	0.6239810294792430	14022
**DHCR7**	0.26629	0.8835	0.6240723924961320	14025
**ASPHD2**	0.73378	0.25992	0.624285593130676	14026
**CLEC11A**	0.73378	0.20503	0.624285593130676	14027
**KLC1**	0.73378	0.0030545	0.624285593130676	14028
**HRK**	0.73396	0.1555	0.6248339536938160	14029
**ATXN7L3**	0.26596	1.1639	0.6250777298125390	14042
**C3orf36**	0.26596	1.5861	0.6250777298125390	14038
**CABYR**	0.26596	2.421	0.6250777298125390	14033
**DSCAM**	0.26596	2.5632	0.6250777298125390	14032
**HEATR4**	0.26596	1.2605	0.6250777298125390	14041
**HJURP**	0.26596	1.7242	0.6250777298125390	14037
**KIAA2013**	0.26596	1.7331	0.6250777298125390	14036
**NPAS1**	0.26596	1.3366	0.6250777298125390	14040
**RIPOR3**	0.26596	2.3614	0.6250777298125390	14034
**TCF24**	0.26596	2.612	0.6250777298125390	14031
**TPK1**	0.26596	1.9246	0.6250777298125390	14035
**TSEN54**	0.26596	1.5649	0.6250777298125390	14039
**WHAMM**	0.26596	2.7121	0.6250777298125390	14030
**KANK1**	0.26582	0.033285	0.6255044274332330	14043
**ZNF551**	0.2658	1.3794	0.6255653935323020	14044
**B9D1**	0.26563	2.0709	0.6260836992967180	14045

**DCLK1**	0.26563	2.0608	0.6260836992967180	14046
**CCKAR**	0.26554	2.4507	0.6263581645641200	14047
**C1orf64**	0.26547	2.7261	0.6265716701749610	14048
**PRKCI**	0.73453	0.23975	0.6265716701749610	14049
**ABL1**	0.26541	0.56264	0.6267546977197770	14050
**FGF23**	0.26533	3.2501	0.6269987671119450	14051
**MYH1**	0.2653	2.4047	0.6270903027634790	14052
**BUB3**	0.2652	1.5805	0.6273954595592910	14054
**LMOD1**	0.2652	1.792	0.6273954595592910	14053
**HUWE1**	0.73489	0.16218	0.6276701506359000	14055
**GTPBP8**	0.26507	2.7894	0.6277922507615960	14056
**TMEM257**	0.26505	1.2043	0.6278533043342980	14057
**JAM2**	0.26486	0.13151	0.6284334300622420	14058
**RBM20**	0.73532	0.074601	0.6289832180158330	14059
**SLC22A18**	0.26461	1.0388	0.6291970757881260	14060
**FAXC**	0.26459	1.3851	0.6292581832942100	14061
**ZNF385C**	0.26451	2.1047	0.6295026368209220	14062
**PDCD5**	0.73554	0.27633	0.6296554393791090	14063
**CCM2L**	0.26418	1.5901	0.6305114055987880	14066
**DTX3**	0.26418	1.621	0.6305114055987880	14065
**HIC2**	0.26418	0.52266	0.6305114055987880	14069
**ITPR1**	0.26418	2.165	0.6305114055987880	14064
**KRTAP19-6**	0.26418	1.2986	0.6305114055987880	14068
**SNRPC**	0.26418	1.5672	0.6305114055987880	14067
**MTMR10**	0.26405	1.9397	0.6309089755351640	14070
**RASSF6**	0.26397	0.1823	0.6311536835292470	14071
**CDC42EP1**	0.26384	0.70178	0.6315514146531760	14072
**F2R**	0.26373	1.9656	0.631888034421192	14073
**RAPGEF4**	0.26366	0.0062549	0.6321022842844510	14074
**TXNDC2**	0.26365	1.3666	0.6321328937762940	14075
**GTPBP4**	0.26362	0.7714	0.6322247258057360	14076
**AAMP**	0.26358	2.7097	0.6323471768060530	14077

**MRPL57**	0.26354	3.1443	0.6324696372887070	14078
**SPESP1**	0.73648	0.11325	0.6325308710869430	14079
**UBE2Q2**	0.26348	2.2608	0.6326533457993000	14080
**DLD**	0.26344	3.3981	0.6327758300022600	14081
**SLC22A5**	0.26339	1.7937	0.632928948607162	14082
**F2**	0.26331	2.3642	0.6331739692463560	14083
**DUSP13**	0.26321	2.1039	0.6334802985122520	14084
**PDIA2**	0.26315	2.2191	0.6336641246073630	14085
**SNCA**	0.26311	2.8715	0.6337866872341710	14086
**RETSAT**	0.26305	1.3168	0.633970549027784	14087
**COL5A2**	0.26298	2.4758	0.6341850815447060	14088
**RELL1**	0.26298	1.5203	0.6341850815447060	14089
**CGRRF1**	0.26295	0.86914	0.6342770329872250	14090
**AHCYL2**	0.2629	0.007468	0.6344302973103070	14096
**CCDC61**	0.2629	1.8156	0.6344302973103070	14091
**PCNP**	0.2629	1.1915	0.6344302973103070	14092
**PDPK1**	0.2629	1.1513	0.6344302973103070	14093
**SLC9C1**	0.2629	0.94	0.6344302973103070	14094
**ZDHHC20**	0.2629	0.76676	0.6344302973103070	14095
**ABCC11**	0.26288	1.1364	0.6344916072123630	14097
**RPL36AL**	0.26286	2.4675	0.6345529194995120	14098
**C10orf120**	0.26274	0.44781	0.6349208433326850	14099
**KRTAP27-1**	0.26267	2.4155	0.6351355052635700	14100
**PMCH**	0.26265	2.6291	0.6351968426193420	14101
**SPZ1**	0.73738	0.4003	0.635288853134162	14102
**CCDC114**	0.26229	2.8777	0.6363013240978220	14103
**NUDT14**	0.26226	1.8466	0.6363933992494240	14104
**FOXA2**	0.26223	2.1793	0.636485479796596	14105
**POLR3A**	0.26221	2.4239	0.6365468698262820	14106
**POLR3D**	0.73783	0.25499	0.6366696570833080	14107
**LYNX1**	0.26209	2.7829	0.6369152603997130	14108
**SOCS1**	0.73797	0.14383	0.6370994881022790	14109

**MANBAL**	0.26196	1.3693	0.6373144477551760	14110
**METTL15**	0.26193	0.27711	0.6374065823358590	14111
**REG1A**	0.26171	0.46682	0.6380824013594310	14112
**FAT3**	0.73833	0.32113	0.6382053088536290	14113
**POLR3B**	0.26155	1.0619	0.6385740891997710	14114
**TEX43**	0.26146	0.95633	0.6388507314603000	14116
**UBL7**	0.26146	0.43151	0.6388507314603000	14117
**VAMP8**	0.26146	2.0824	0.6388507314603000	14115
**ZNF285**	0.26131	1.9579	0.6393119105816210	14118
**SH2D4A**	0.26109	2.7095	0.6399885527094810	14119
**PLXNA2**	0.26096	2.1098	0.640388524447382	14120
**IRGM**	0.26094	1.4766	0.6404500676533370	14121
**NUTM2B**	0.26092	0.021818	0.6405116132851420	14122
**C10orf35**	0.26044	2.7347	0.6419894373082670	14123
**DHRSX**	0.26041	2.9439	0.6420818478464050	14124
**GLG1**	0.73964	0.29332	0.64223587759631	14126
**HLA-DRB5**	0.73964	0.49181	0.64223587759631	14125
**DCUN1D4**	0.26032	2.9468	0.642359112367783	14127
**GREB1L**	0.26032	1.6764	0.642359112367783	14130
**PRKACG**	0.26032	2.5061	0.642359112367783	14128
**SCAF4**	0.26032	2.2786	0.642359112367783	14129
**DEFB107A**	0.26022	2.5423	0.6426672419872610	14134
**IGFN1**	0.26022	2.5746	0.6426672419872610	14132
**PTCD1**	0.26022	0.52395	0.6426672419872610	14133
**SLC29A4**	0.26022	1.7511	0.6426672419872610	14138
**SP110**	0.26022	1.7194	0.6426672419872610	14136
**TULP1**	0.26022	2.9467	0.6426672419872610	14137
**ZNF320**	0.26022	2.6712	0.6426672419872610	14131
**ZNF830**	0.26022	2.1976	0.6426672419872610	14135
**CMAS**	0.26015	0.14085	0.6428829690303190	14139
**PGLYRP2**	0.26014	2.5746	0.6429137896215780	14140
**DDX41**	0.26011	1.9371	0.6430062550599150	14141

**OR13J1**	0.25998	0.20741	0.6434070021765210	14142
**PDPR**	0.25968	2.7963	0.644332197879849	14143
**ULBP1**	0.25968	2.4968	0.644332197879849	14144
**GLO1**	0.74065	0.64075	0.6453505506202320	14145
**PLXNA1**	0.25926	1.7139	0.6456283993328530	14146
**TEX19**	0.25923	2.1478	0.6457210266465710	14147
**UGT1A10**	0.2592	2.4895	0.6458136595008070	14148
**SPHK2**	0.74082	0.23742	0.6458754178157490	14149
**ZNF584**	0.25894	1.4531	0.6466167098742310	14150
**FAM117B**	0.25888	0.35115	0.646802088405919	14151
**OR8D2**	0.25882	1.9662	0.6469874891678210	14152
**PRPSAP1**	0.2588	1.9197	0.6470492943638590	14153
**DDX51**	0.25868	1.9289	0.6474201774621980	14158
**DNAJB2**	0.25868	1.4091	0.6474201774621980	14160
**FBN2**	0.25868	2.4984	0.6474201774621980	14156
**FGF22**	0.25868	2.5468	0.6474201774621980	14155
**GTF3C2**	0.25868	2.6898	0.6474201774621980	14154
**MFSD10**	0.25868	1.6653	0.6474201774621980	14159
**NUDT10**	0.25868	0.012982	0.6474201774621980	14161
**UGT2A3**	0.25868	2.4162	0.6474201774621980	14157
**DLG5**	0.2584	0.21328	0.6482859179314010	14162
**ABCC6**	0.25834	0.12589	0.6484714969570640	14163
**GLCCI1**	0.74167	0.15347	0.6485024289658970	14164
**CCL27**	0.25831	2.295	0.6485642948450990	14168
**NOA1**	0.25831	2.5233	0.6485642948450990	14167
**TBC1D5**	0.25831	3.0957	0.6485642948450990	14166
**ZNF99**	0.25831	3.2786	0.6485642948450990	14165
**OR6C65**	0.74182	0.3794	0.6489664835882290	14169
**BTBD7**	0.25813	2.2983	0.649121199518864	14170
**USPL1**	0.74213	0.68879	0.6499259730111750	14171
**AGRP**	0.25783	1.9231	0.6500498216740230	14172
**CHMP4A**	0.74223	0.39282	0.6502356133661670	14173

**GPC3**	0.25768	0.88382	0.6505143429980260	14174
**HPS6**	0.25721	2.5407	0.6519707532385160	14175
**LOC284513**	0.25716	2.3605	0.6521257718602040	14176
**NOXO1**	0.25709	2.5579	0.6523428242622730	14177
**KRT13**	0.25707	2.598	0.6524048448791180	14178
**PGAP1**	0.25704	2.167	0.6524978805100630	14180
**WDR12**	0.25704	2.2749	0.6524978805100630	14179
**FPGS**	0.25698	1.274	0.6526839687178570	14182
**VENTX**	0.25698	3.3917	0.6526839687178570	14181
**ARF5**	0.25697	1.7765	0.652714985616492	14186
**LINC01750**	0.25697	1.6543	0.652714985616492	14187
**MAGEB17**	0.25697	2.588	0.652714985616492	14183
**MCM5**	0.25697	2.0271	0.652714985616492	14185
**RAB34**	0.25697	1.5445	0.652714985616492	14188
**SERINC3**	0.25697	2.5683	0.652714985616492	14184
**KCNK6**	0.25693	2.8314	0.6528390594911600	14189
**C6orf222**	0.25692	3.2193	0.6528700795301370	14190
**NAPG**	0.25688	2.0384	0.652994165968932	14191
**RASSF10**	0.2567	2.8084	0.6535526794287880	14192
**PSPC1**	0.74335	0.40779	0.653707858236208	14193
**NR2C2**	0.25653	2.0535	0.654080351619586	14194
**ORAI3**	0.25649	2.7794	0.6542045362486130	14195
**SKOR2**	0.25648	2.2039	0.6542355839822740	14196
**SORBS2**	0.25648	1.2179	0.6542355839822740	14197
**RPL36A**	0.2563	1.9251	0.6547945510870370	14198
**ELK4**	0.25614	1.0774	0.6552915825408440	14199
**ANKRD24**	0.25607	0.92593	0.6555090847098870	14200
**MORF4L1**	0.25604	2.3323	0.6556023094184020	14201
**MAIP1**	0.25595	2.1694	0.6558820177385900	14203
**TERT**	0.25595	3.1648	0.6558820177385900	14202
**SHCBP1**	0.25583	0.36251	0.6562550420093020	14204
**ZNF45**	0.2558	0.69212	0.6563483123448590	14205

**U2AF1**	0.25566	0.46174	0.6567836494363800	14206
**GGT5**	0.25554	0.97359	0.6571568946076070	14207
**LZTS3**	0.25539	2.2783	0.6576235798568600	14208
**HDGFRP2**	0.25531	1.2305	0.6578725372265460	14212
**KBTBD7**	0.25531	1.5376	0.6578725372265460	14211
**LYZL1**	0.25531	2.5606	0.6578725372265460	14209
**MRGPRG**	0.25531	0.057329	0.6578725372265460	14214
**RGL4**	0.25531	0.95847	0.6578725372265460	14213
**TEKT4**	0.25531	2.0149	0.6578725372265460	14210
**OR3A2**	0.25524	0.3644	0.6580904083776730	14215
**ERVV-1**	0.25516	1.8448	0.6583394422364430	14216
**C4orf22**	0.25504	1.7872	0.6587130696003760	14217
**POLM**	0.25502	3.3847	0.6587753497673230	14218
**ANXA5**	0.25487	2.7428	0.6592425325034120	14219
**CEP350**	0.25485	0.69586	0.659304834405499	14220
**ZNF678**	0.74519	0.16119	0.6594294458877880	14221
**TGDS**	0.2548	2.9516	0.6594606003582980	14222
**LRRC25**	0.25476	2.8875	0.6595852246417810	14223
**B3GALNT1**	0.25468	0.5149	0.6598345039474460	14224
**C7orf25**	0.2546	1.9529	0.6600838242621910	14228
**SLITRK2**	0.2546	2.6241	0.6600838242621910	14225
**SURF2**	0.2546	2.0934	0.6600838242621910	14227
**WNT1**	0.2546	2.1881	0.6600838242621910	14226
**NUSAP1**	0.25459	1.3408	0.6601149921864290	14229
**HIST1H2BB**	0.25443	1.159	0.6606137662264050	14230
**ACTRT2**	0.74567	0.3437	0.6609255834705670	14231
**TRIM26**	0.25428	1.2808	0.661081516192296	14232
**TXNDC5**	0.25424	1.3271	0.6612062739439890	14233
**EMG1**	0.25418	3.0312	0.6613934298705730	14234
**ADAMTS7**	0.25417	2.8373	0.6614246247772900	14235
**CRYAB**	0.2541	0.32698	0.6616430071500240	14236
**SPATA22**	0.25408	2.1344	0.6617054079093520	14237

**GLA**	0.25406	0.11145	0.6617678112453780	14238
**SPINK9**	0.25401	3.276	0.6619238308614850	14239
**FAM120AOS**	0.25391	0.50593	0.662235918443831	14240
**PABPC1**	0.74632	0.41704	0.6629539648092360	14241
**C1S**	0.25366	3.2324	0.663016419775066	14242
**HIST1H1C**	0.25366	2.2768	0.663016419775066	14243
**XKRX**	0.25366	2.2506	0.663016419775066	14244
**ZHX3**	0.25366	1.2355	0.663016419775066	14245
**FAM78B**	0.25364	0.9267	0.6630788773271870	14246
**C19orf38**	0.25351	0.3446	0.6634849144884270	14256
**OR1L3**	0.25351	1.3239	0.6634849144884270	14251
**PCDHA4**	0.25351	1.2223	0.6634849144884270	14252
**RBMY1J**	0.25351	0.85935	0.6634849144884270	14254
**RHOA**	0.25351	1.6205	0.6634849144884270	14249
**RNF38**	0.25351	0.59814	0.6634849144884270	14255
**RTL8C**	0.25351	1.2056	0.6634849144884270	14253
**STARD9**	0.25351	1.4748	0.6634849144884270	14250
**SYMPK**	0.25351	1.6336	0.6634849144884270	14248
**TENM3**	0.25351	2.255	0.6634849144884270	14247
**ALS2CL**	0.25335	0.48179	0.663984802750505	14257
**ZBTB46**	0.25332	0.47848	0.6640785502705190	14258
**GAS2L2**	0.25324	2.7412	0.6643285721963970	14259
**TRIM39-RPI**	0.25304	2.9774	0.6649538087903400	14260
**CDH5**	0.25301	2.7781	0.6650476166977430	14261
**SLC25A32**	0.25289	1.8478	0.6654229068703920	14262
**CCNE1**	0.25273	2.9226	0.6659234396078820	14263
**DRICH1**	0.25273	1.9706	0.6659234396078820	14265
**TSPAN14**	0.25273	2.5214	0.6659234396078820	14264
**DEPTOR**	0.25267	1.1623	0.6661111823985260	14266
**CHADL**	0.25265	1.626	0.6661737685462690	14267
**LMO7**	0.25257	0.1892	0.6664241392372150	14268
**TMEM62**	0.25241	2.6607	0.6669250059953310	14269

**SRGAP2C**	0.25239	1.0281	0.6669876261031540	14270
**PSME2**	0.25237	2.5981	0.6670502488265360	14271
**FEV**	0.25229	0.44665	0.6673007658849410	14272
**PAX4**	0.25204	2.2643	0.6680839017972400	14273
**DEFB121**	0.25198	0.30405	0.6682719153828380	14277
**HORMAD1**	0.25198	0.44738	0.6682719153828380	14276
**TMEM147**	0.25198	1.8047	0.6682719153828380	14275
**TOP2A**	0.25198	2.5709	0.6682719153828380	14274
**SLC22A3**	0.25197	2.5247	0.6683032532770540	14278
**RPS18**	0.25193	2.15	0.6684286114178190	14279
**LOC401052**	0.25192	1.8311	0.6684599525942760	14280
**RNF215**	0.74811	0.84944	0.6685539800636180	14281
**SDK2**	0.74811	0.42431	0.6685539800636180	14282
**UFSP1**	0.74831	0.34741	0.6691809809987970	14283
**WHRN**	0.25162	0.41781	0.6694004934799330	14284
**C4orf26**	0.25161	2.6429	0.6694318550388400	14285
**LPIN3**	0.25121	2.5698	0.6706868579346250	14286
**DHH**	0.25106	3.4004	0.6711577563898030	14287
**TMEM63B**	0.25106	1.105	0.6711577563898030	14288
**DLC1**	0.74909	0.0092509	0.6716288037184840	14289
**ZNF471**	0.74911	0.034832	0.6716916212878700	14290
**F5**	0.25083	0.68472	0.671880089901779	14292
**INSL6**	0.25083	2.7913	0.671880089901779	14291
**OR10AD1**	0.74923	0.51303	0.6720685823842250	14293
**LIMA1**	0.25075	2.3439	0.6721314185180290	14294
**GPR37L1**	0.25072	0.89559	0.6722256776950350	14295
**POLR1E**	0.74929	0.09485	0.6722570987479530	14296
**SLC16A6**	0.25066	1.1967	0.6724142139695730	14297
**SETMAR**	0.25063	1.9179	0.672508491070294	14298
**LAMA5**	0.25043	3.0501	0.6731371579172430	14299
**BUD13**	0.25032	1.4287	0.6734830380999790	14300
**CAV3**	0.25019	2.2264	0.6738919094957520	14305

**CDC37**	0.25019	2.2304	0.6738919094957520	14304
**DEFB113**	0.25019	2.3778	0.6738919094957520	14301
**FOLR2**	0.25019	2.242	0.6738919094957520	14302
**KLHL38**	0.25019	2.1065	0.6738919094957520	14306
**LIN52**	0.25019	0.81308	0.6738919094957520	14314
**NES**	0.25019	1.806	0.6738919094957520	14308
**OR2A1**	0.25019	2.238	0.6738919094957520	14303
**PKIG**	0.25019	1.6634	0.6738919094957520	14310
**SIGMAR1**	0.25019	0.85533	0.6738919094957520	14313
**TCF19**	0.25019	1.9171	0.6738919094957520	14307
**WASF3**	0.25019	1.0688	0.6738919094957520	14311
**ZFYVE19**	0.25019	0.92103	0.6738919094957520	14312
**ZNF630**	0.25019	1.7158	0.6738919094957520	14309
**FAM25A**	0.74998	0.012529	0.6744267575298370	14315
**CLIP3**	0.24979	2.262	0.6751506827074320	14316
**CDIP1**	0.75022	0.26577	0.6751821657401140	14317
**NWD2**	0.24971	3.1674	0.675402565711055	14319
**ODF1**	0.24971	3.37	0.675402565711055	14318
**UBE2R2**	0.24968	2.4127	0.6754970328851850	14320
**FBXO16**	0.75036	0.21021	0.6756229984954030	14321
**MDM4**	0.24945	1.2989	0.6762214816216090	14322
**ELOVL3**	0.24944	2.7154	0.6762529874420820	14323
**ATP4B**	0.2494	2.061	0.6763790174373320	14324
**NPAS2**	0.24929	1.7919	0.6767256553358140	14325
**SCD5**	0.7508	0.38376	0.6770093286539650	14326
**IFI27**	0.24917	0.047379	0.6771038985333860	14327
**CD101**	0.24911	3.3736	0.6772930564620080	14328
**DENND6B**	0.2491	1.827	0.6773245851394880	14329
**CCNJL**	0.249	3.1136	0.6776399089565480	14330
**TSPY2**	0.75104	0.44529	0.67776605734967	14331
**ANP32B**	0.24893	0.77635	0.6778606757229540	14332
**SLAMF6**	0.24869	0.90041	0.6786178413313700	14333

**ACAA1**	0.2486	1.9802	0.6789018787437970	14335
**DFNA5**	0.2486	2.9284	0.6789018787437970	14334
**DNAJC21**	0.2486	1.8254	0.6789018787437970	14336
**KIF6**	0.2486	1.7025	0.6789018787437970	14337
**KMO**	0.2486	0.76492	0.6789018787437970	14340
**ZNF180**	0.2486	0.98605	0.6789018787437970	14339
**ZXDA**	0.2486	1.4282	0.6789018787437970	14338
**TMEM9**	0.24832	0.95673	0.6797859012301460	14341
**AGBL1**	0.24829	1.3842	0.6798806494297140	14343
**TPST1**	0.24829	3.0465	0.6798806494297140	14342
**CEBPZ**	0.24823	2.7448	0.6800701641420200	14346
**CTLA4**	0.24823	2.2298	0.6800701641420200	14348
**FLJ45513**	0.24823	1.6493	0.6800701641420200	14351
**GJB2**	0.24823	2.3279	0.6800701641420200	14347
**LOC100506**	0.24823	1.9595	0.6800701641420200	14350
**OR2T10**	0.24823	2.7657	0.6800701641420200	14345
**PERP**	0.24823	2.788	0.6800701641420200	14344
**PIM2**	0.24823	0.23104	0.6800701641420200	14354
**RPL28**	0.24823	0.75838	0.6800701641420200	14352
**TSPAN19**	0.24823	0.24949	0.6800701641420200	14353
**UQCC1**	0.24823	2.0769	0.6800701641420200	14349
**PRR36**	0.24818	0.20601	0.6802281117290280	14355
**DPAGT1**	0.24814	1.8258	0.6803544820179710	14356
**ZNF774**	0.75189	0.29508	0.680449266865175	14357
**C1orf158**	0.75195	0.31366	0.6806388549022520	14358
**CUTC**	0.24791	0.12498	0.6810813221641470	14359
**LRRFIP2**	0.2479	2.2076	0.6811129320704430	14360
**ORAOV1**	0.24787	1.42	0.6812077658730210	14361
**WISP3**	0.24782	2.1368	0.6813658358266100	14362
**TRAPPC11**	0.24772	2.8375	0.6816820268210520	14363
**PHC3**	0.75234	0.39844	0.6818717741340560	14364
**HSD3B1**	0.24763	0.65502	0.6819666569972460	14365

**TFR2**	0.24754	2.9254	0.6822513424333480	14366
**PID1**	0.24746	2.879	0.6825044425813690	14367
**ZNF800**	0.24746	1.8149	0.6825044425813690	14368
**BLM**	0.75255	0.015394	0.6825360831739010	14369
**GPR35**	0.75257	0.54615	0.6825993664089890	14370
**EXOC6B**	0.2473	1.3815	0.6830107740947220	14371
**ABHD2**	0.24728	2.514	0.683074077845065	14372
**SLC22A2**	0.24725	1.6278	0.6831690386034530	14373
**OR5P3**	0.24711	2.4922	0.6836122702970720	14374
**SMAGP**	0.24709	0.32318	0.68367560007301	14375
**SIMC1**	0.75319	0.37607	0.6845625050819450	14376
**OR4M2**	0.24676	1.2161	0.6847209376356930	14377
**NPY4R**	0.75333	0.14296	0.6850061595538740	14378
**ASGR1**	0.24665	2.2766	0.6850695497700380	14381
**CDC123**	0.24665	1.5431	0.6850695497700380	14385
**CDKN2D**	0.24665	2.5088	0.6850695497700380	14380
**ETV6**	0.24665	1.9568	0.6850695497700380	14382
**LRRC39**	0.24665	1.7297	0.6850695497700380	14383
**MAP3K15**	0.24665	2.55	0.6850695497700380	14379
**RETREG3**	0.24665	1.3343	0.6850695497700380	14386
**SH2D7**	0.24665	1.1217	0.6850695497700380	14387
**TBC1D8**	0.24665	1.7024	0.6850695497700380	14384
**DCTN1**	0.2466	3.1656	0.6852280373556960	14388
**CRYM**	0.75348	0.068223	0.6854816533004030	14389
**NOC3L**	0.24649	2.145	0.68557677064715	14390
**EIF3D**	0.24636	2.0107	0.6859890175174160	14391
**ATL2**	0.24632	2.9113	0.6861158861630150	14393
**C11orf49**	0.24632	0.45637	0.6861158861630150	14396
**MRPS35**	0.24632	2.9691	0.6861158861630150	14392
**NT5E**	0.24632	2.5239	0.6861158861630150	14394
**SSU72**	0.24632	1.9021	0.6861158861630150	14395
**HSPA1A**	0.24607	2.2882	0.6869090655814780	14397

**GOLGA6C**	0.24596	2.1733	0.687258201450596	14398
**SPRR2G**	0.75408	0.30383	0.6873851807195200	14399
**CDCA4**	0.75415	0.07489	0.6876074211138960	14400
**BAZ1A**	0.24575	1.8758	0.6879249663187410	14401
**ICAM2**	0.75432	0.023656	0.6881472892408100	14402
**ANKRD65**	0.24566	0.57946	0.6882108163230750	14403
**PLD6**	0.24564	1.6737	0.688274346182871	14404
**LIF**	0.24556	2.9171	0.6885284934073630	14405
**RIN3**	0.2455	2.8145	0.6887191330142930	14406
**HECW2**	0.24543	1.9712	0.6889415775298930	14407
**FICD**	0.24529	0.3771	0.6893865688681280	14408
**LKAAEAR1**	0.75485	0.31417	0.6898316967589520	14409
**ARR3**	0.24513	2.4876	0.6898952976148230	14411
**CCIN**	0.24513	1.852	0.6898952976148230	14414
**ERCC1**	0.24513	1.2079	0.6898952976148230	14417
**GALNT7**	0.24513	1.5572	0.6898952976148230	14415
**GRIK5**	0.24513	2.0248	0.6898952976148230	14413
**MED23**	0.24513	2.2199	0.6898952976148230	14412
**PDK3**	0.24513	1.2053	0.6898952976148230	14418
**RNF220**	0.24513	1.4671	0.6898952976148230	14416
**SPRR3**	0.24513	2.9611	0.6898952976148230	14410
**IQCF1**	0.2451	2.176	0.689990704131536	14419
**LPIN1**	0.24499	0.80554	0.6903405817764770	14420
**CYFIP1**	0.24491	2.3784	0.6905950913328160	14422
**KCNK3**	0.24491	2.3784	0.6905950913328160	14421
**RBM38**	0.2449	2.361	0.6906269081725710	14423
**FNTB**	0.24477	1.5278	0.6910405907345870	14424
**LSM14A**	0.24468	2.7233	0.6913270556426350	14425
**TRMT13**	0.24464	2.2546	0.6914543915906500	14426
**NGFR**	0.75538	0.43877	0.6915180637691120	14427
**FBXO45**	0.24443	3.2428	0.6921230893676280	14430
**FMO1**	0.24443	3.2002	0.6921230893676280	14431

**MALT1**	0.24443	1.8922	0.6921230893676280	14434
**MVP**	0.24443	2.2449	0.6921230893676280	14432
**NDUFA1**	0.24443	3.2527	0.6921230893676280	14429
**TEKT5**	0.24443	3.4343	0.6921230893676280	14428
**ZSCAN18**	0.24443	1.9016	0.6921230893676280	14433
**HSPB3**	0.75564	0.070533	0.6923460574046460	14435
**PRRT2**	0.24435	0.05127	0.6923779127910740	14436
**PKIA**	0.75573	0.46007	0.692632781182071	14437
**PCDHB16**	0.75582	0.49759	0.6929195619126040	14438
**FAM178B**	0.75584	0.070418	0.6929832987028550	14439
**CHAD**	0.24413	0.31227	0.6930789091670580	14440
**SCNN1B**	0.24409	2.7134	0.6932063996424690	14441
**CSPG5**	0.24404	0.12186	0.6933657785832090	14442
**PLIN2**	0.7561	0.11326	0.6938121333523530	14443
**BEAN1**	0.24355	1.7692	0.6949286256020720	14454
**BECN2**	0.24355	2.5239	0.6949286256020720	14446
**HIST1H1A**	0.24355	2.5079	0.6949286256020720	14447
**KCND2**	0.24355	0.82003	0.6949286256020720	14457
**PDC**	0.24355	2.7806	0.6949286256020720	14444
**PHLDA2**	0.75645	0.16598	0.6949286256020720	14459
**PRIM1**	0.24355	2.3319	0.6949286256020720	14448
**RAET1E**	0.24355	0.19969	0.6949286256020720	14458
**RPE65**	0.24355	2.023	0.6949286256020720	14451
**SCIMP**	0.24355	1.7906	0.6949286256020720	14453
**SIRT1**	0.24355	2.1981	0.6949286256020720	14450
**STK40**	0.24355	1.8102	0.6949286256020720	14452
**TEKT2**	0.24355	1.6119	0.6949286256020720	14456
**TPGS1**	0.24355	2.5699	0.6949286256020720	14445
**ZNF16**	0.24355	1.7127	0.6949286256020720	14455
**ZNF534**	0.24355	2.2581	0.6949286256020720	14449
**ZNF789**	0.24355	0.016431	0.6949286256020720	14460
**COX11**	0.24351	1.1198	0.6950562798659680	14461

**SIX5**	0.24323	0.50094	0.695950177109394	14462
**CRLF2**	0.24318	2.8336	0.696109860131356	14463
**C3orf20**	0.24307	2.2659	0.6964612252775620	14464
**RBM45**	0.24304	0.24047	0.6965570670584250	14465
**KIAA2022**	0.24299	0.63991	0.6967168175801950	14466
**MRGPRF**	0.24291	1.7986	0.696972455405719	14467
**GJA1**	0.24289	2.7393	0.69703637197891	14468
**CMTM6**	0.24287	2.6677	0.6971002913998550	14469
**GABARAPL1**	0.24287	1.9619	0.6971002913998550	14470
**OR1J4**	0.24285	2.1749	0.6971642136690680	14471
**SUFU**	0.24279	2.6167	0.6973559975714690	14472
**TSKS**	0.75723	0.25465	0.6974199312389080	14473
**GAS2L3**	0.24274	0.40586	0.6975158370855620	14474
**ATP2C2**	0.24261	0.69935	0.6979315032521650	14475
**CCDC152**	0.2425	1.3215	0.6982833149861990	14476
**CCDC102A**	0.24235	2.5397	0.6987631975952300	14477
**NEK10**	0.24229	0.038181	0.6989551956983490	14478
**OAS1**	0.24227	1.6076	0.6990192007918690	14479
**BPIFB2**	0.75776	0.29269	0.6991152138018800	14481
**WDR25**	0.24224	2.2593	0.6991152138018800	14480
**CHRM4**	0.2422	2.4198	0.6992432411747840	14482
**OR8H2**	0.24217	0.98024	0.6993392692263990	14484
**SLC6A3**	0.24217	2.0313	0.6993392692263990	14483
**DMGDH**	0.24215	1.9762	0.6994032915103230	14485
**AZGP1**	0.24206	2.5454	0.6996914272731630	14486
**CA1**	0.24203	2.4223	0.6997874854366000	14487
**ABHD8**	0.242	1.7443	0.6998835500575380	14489
**ADGRE3**	0.242	0.90548	0.6998835500575380	14493
**ARF6**	0.242	2.0163	0.6998835500575380	14488
**C7orf65**	0.242	0.41669	0.6998835500575380	14495
**GMCL1**	0.242	0.66892	0.6998835500575380	14494
**MICALL1**	0.242	1.5949	0.6998835500575380	14490

**NIM1K**	0.242	0.3755	0.6998835500575380	14496
**PTTG2**	0.242	1.5336	0.6998835500575380	14491
**RBM3**	0.242	1.3586	0.6998835500575380	14492
**RGS21**	0.242	0.30286	0.6998835500575380	14497
**KLHDC4**	0.24199	2.618	0.6999155730331550	14498
**LRRC4C**	0.24195	3.3877	0.7000436721138730	14499
**SLC35G6**	0.24186	2.293	0.7003319370560230	14500
**CCL3L1**	0.24183	2.0557	0.7004280383026380	14501
**LYRM4**	0.24174	3.4932	0.700716380864791	14502
**COL4A3BP**	0.2415	3.0289	0.701485579370654	14504
**KDELR3**	0.2415	3.1951	0.701485579370654	14503
**RFK**	0.24145	1.0481	0.7016458812985960	14505
**NANOS3**	0.24141	2.1813	0.701774135823627	14506
**KCTD13**	0.24135	1.908	0.7019665392587850	14507
**MIPOL1**	0.24105	1.4872	0.7029289465632460	14508
**BTLA**	0.24101	1.7854	0.7030573167239590	14509
**STIM1**	0.24095	1.8612	0.7032498936913520	14510
**XRN1**	0.24083	2.8628	0.7036351258929940	14511
**E2F5**	0.24082	0.37124	0.7036672332896790	14512
**ODC1**	0.24081	0.23605	0.7036993414117810	14513
**LRRC3B**	0.75922	0.33825	0.7037956701312500	14514
**PDE4C**	0.24063	2.4949	0.7042774117311190	14515
**FAM19A4**	0.24056	2.3776	0.704502280417791	14516
**NRIP3**	0.75947	0.41447	0.7045986636177480	14518
**PLEKHM1**	0.24053	2.7703	0.7045986636177480	14517
**NPIPB6**	0.24051	2.0938	0.7046629227209300	14519
**ZNF705E**	0.2405	2.47	0.7046950533636820	14520
**TANC2**	0.24047	2.9061	0.7047914496573800	14521
**UBTD1**	0.24042	0.98764	0.7049521247023110	14522
**GAS6**	0.24036	3.337	0.7051449587828840	14523
**C3orf70**	0.24031	0.3355	0.7053056738819500	14535
**CGGBP1**	0.24031	0.34259	0.7053056738819500	14534

**GPX8**	0.24031	1.7259	0.7053056738819500	14532
**HSD17B12**	0.24031	2.8036	0.7053056738819500	14524
**KIAA0319L**	0.24031	2.0194	0.7053056738819500	14530
**MARK3**	0.24031	2.1898	0.7053056738819500	14526
**OR52I1**	0.24031	2.1878	0.7053056738819500	14527
**SERINC1**	0.24031	1.7781	0.7053056738819500	14531
**SIRT4**	0.24031	2.0986	0.7053056738819500	14528
**SPATS2L**	0.24031	2.7484	0.7053056738819500	14525
**TMEM184B**	0.24031	1.2126	0.7053056738819500	14533
**TOP3A**	0.24031	2.0688	0.7053056738819500	14529
**TNMD**	0.24001	2.9393	0.7062703473795730	14536
**WDR73**	0.75999	0.37309	0.706270347379573	14537
**GALNT14**	0.23991	1.8104	0.7065920512791570	14539
**NAA16**	0.23991	3.3172	0.7065920512791570	14538
**MTRF1L**	0.7602	0.16274	0.706946010052967	14540
**KIAA1324L**	0.76024	0.12111	0.7070747442949660	14541
**UBAP2**	0.76042	0.22946	0.7076541934692290	14542
**NKIRAS2**	0.23957	2.3917	0.7076863920560450	14543
**UBE2A**	0.23955	1.8907	0.7077507914308740	14544
**CCNK**	0.23951	1.711	0.7078795989872000	14545
**FOS**	0.23949	0.87258	0.7079440071697660	14546
**MRPS25**	0.23943	2.6647	0.7081372493415000	14547
**ZNF510**	0.23917	2.6793	0.7089749378845860	14548
**CEP57**	0.23908	2.0708	0.7092650229171710	14549
**FIGN**	0.23901	1.2037	0.7094906858783450	14550
**USP17L8**	0.23894	2.5188	0.7097163849752900	14551
**C11orf53**	0.23886	3.4328	0.7099743710778540	14552
**SDC1**	0.23881	1.6381	0.7101356363901820	14553
**CAB39**	0.23872	0.2281	0.7104259605031390	14554
**WNT10A**	0.23867	1.6653	0.7105872775534920	14555
**NTN3**	0.2386	0.2503	0.7108131524956090	14556
**CREBRF**	0.23858	2.4758	0.7108776948543990	14557

**ECSCR**	0.23858	2.3906	0.7108776948543990	14560
**GSTT2B**	0.23858	1.7941	0.7108776948543990	14561
**HPGD**	0.23858	1.7025	0.7108776948543990	14562
**OLAH**	0.23858	1.3253	0.7108776948543990	14563
**PEPD**	0.23858	0.45026	0.7108776948543990	14564
**ZDHHC4**	0.23858	2.4226	0.7108776948543990	14559
**ZNF467**	0.23858	2.4722	0.7108776948543990	14558
**AMT**	0.23849	0.409	0.7111681721247510	14565
**DCLRE1A**	0.23843	1.2741	0.7113618569790170	14567
**LMBRD1**	0.23843	0.34653	0.7113618569790170	14568
**OR9I1**	0.23843	3.1735	0.7113618569790170	14566
**DHRS4L1**	0.23793	2.4943	0.7129769367525350	14569
**KRTAP10-1(**	0.2379	2.978	0.7130739006636860	14570
**PRTFDC1**	0.7621	0.022225	0.7130739006636860	14571
**DHFR**	0.23777	1.9109	0.7134941551132210	14572
**COMMD6**	0.23775	1.8569	0.7135588208281130	14573
**C9orf129**	0.76248	0.54139	0.7143026911615790	14574
**FGD6**	0.23749	0.92798	0.7143997468642950	14575
**CCDC167**	0.23741	0.87302	0.7146585949786980	14576
**ARPC5L**	0.23731	1.9702	0.7149822224749400	14578
**CYP17A1**	0.23731	1.7529	0.7149822224749400	14580
**FIGLA**	0.23731	1.5973	0.7149822224749400	14581
**GAR1**	0.23731	1.8985	0.7149822224749400	14579
**OR6C1**	0.23731	2.38	0.7149822224749400	14577
**RNASE2**	0.23731	0.46337	0.7149822224749400	14583
**TOP2B**	0.23731	1.0938	0.7149822224749400	14582
**TDRD9**	0.23717	1.9711	0.7154354268185560	14584
**NPL**	0.23704	2.2074	0.7158563910390330	14585
**U2AF1L4**	0.23696	0.80559	0.7161155090068340	14586
**FAM109B**	0.23684	1.2463	0.7165042761392200	14587
**WIPF1**	0.2367	2.2168	0.716957974709844	14588
**ZKSCAN5**	0.23656	1.9361	0.7174118209089890	14589

**ANKRD62**	0.23648	3.4039	0.7176712279493780	14590
**MAATS1**	0.23648	1.9517	0.7176712279493780	14591
**SPDYE5**	0.23648	1.6506	0.7176712279493780	14592
**CFL1**	0.23616	3.038	0.7187093394915530	14594
**CHDH**	0.23616	2.1084	0.7187093394915530	14599
**COL28A1**	0.23616	0.065273	0.7187093394915530	14604
**E2F1**	0.23616	3.0752	0.7187093394915530	14593
**ESRP1**	0.23616	2.2716	0.7187093394915530	14598
**HMGCLL1**	0.23616	2.0988	0.7187093394915530	14600
**LRRTM2**	0.23616	2.5966	0.7187093394915530	14595
**NOTCH2NL**	0.23616	0.48929	0.7187093394915530	14602
**SLAMF7**	0.23616	2.3606	0.7187093394915530	14596
**SMG7**	0.23616	0.1828	0.7187093394915530	14603
**ST5**	0.23616	2.2967	0.7187093394915530	14597
**ZNF266**	0.23616	1.9303	0.7187093394915530	14601
**HIST1H2BF**	0.23606	2.1944	0.7190339082005260	14605
**GDF6**	0.23584	1.0962	0.7197482261485210	14606
**MUSK**	0.2357	1.7921	0.7202029833807590	14607
**ZNF354C**	0.23564	0.94674	0.7203979249354060	14608
**XPA**	0.23559	2.4727	0.7205603971460280	14609
**GLCE**	0.76442	0.132	0.7205928938706360	14610
**TRIM40**	0.23552	1.707	0.7207878902015560	14611
**LOC389602**	0.2355	0.86617	0.7208528950690730	14612
**ARHGAP6**	0.23544	1.3548	0.7210479279510940	14613
**NDC1**	0.23538	1.7498	0.7212429882640930	14614
**CHAT**	0.76464	0.033638	0.7213080144668150	14615
**U2AF1L5**	0.23533	2.8985	0.7214055594900670	14616
**ZDHHC2**	0.23532	3.2684	0.7214380760232110	14617
**TLX1**	0.23526	0.72507	0.7216331912435980	14618
**USP40**	0.23517	1.6117	0.7219259155972300	14619
**SYNE1**	0.23514	0.04835	0.7220235041288420	14620
**PIM3**	0.23512	2.4469	0.7220885669701860	14621

**SS18L2**	0.7649	0.24442	0.7221536328684030	14622
**EPS15**	0.23508	1.9292	0.7222187018240550	14623
**TNFRSF1A**	0.23505	2.2309	0.7223163109914540	14624
**UQCRFS1**	0.23497	3.1404	0.7225766357569510	14625
**PAGE3**	0.23495	1.7766	0.722641724599657	14626
**ABCF1**	0.23475	2.0146	0.7232927815110830	14631
**BMP6**	0.23475	0.22468	0.7232927815110830	14636
**C14orf2**	0.23475	2.1059	0.7232927815110830	14630
**C2orf80**	0.23475	1.2942	0.7232927815110830	14635
**CAMK2D**	0.23475	2.5823	0.7232927815110830	14627
**EGR4**	0.23475	2.3456	0.7232927815110830	14628
**GPATCH2L**	0.23475	1.6849	0.7232927815110830	14633
**IRF9**	0.23475	1.4586	0.7232927815110830	14634
**KIFAP3**	0.23475	1.8153	0.7232927815110830	14632
**MANF**	0.23475	0.0093183	0.7232927815110830	14637
**TEP1**	0.23475	2.179	0.7232927815110830	14629
**TMPPE**	0.23468	3.5801	0.7235207238662400	14638
**LY6G6F**	0.23463	2.8922	0.7236835628524180	14640
**VPREB1**	0.23463	3.3002	0.7236835628524180	14639
**RABEPK**	0.7654	0.30756	0.7237812754556510	14641
**IL6R**	0.23441	2.4155	0.7244002825100100	14642
**POLR2L**	0.2344	0.39184	0.7244328695167340	14643
**MIP**	0.23438	2.9539	0.7244980458381610	14644
**SAMD5**	0.234	0.91614	0.7257369813714890	14645
**RPS3A**	0.76604	0.0081378	0.7258674604307590	14646
**YPEL1**	0.23368	2.1435	0.7267811601510720	14647
**CLDN19**	0.23367	2.8083	0.7268138035038740	14648
**CADPS2**	0.23312	3.346	0.7286103826294770	14650
**EID3**	0.23312	2.9031	0.7286103826294770	14653
**LPCAT3**	0.23312	2.9724	0.7286103826294770	14652
**MC3R**	0.23312	3.2038	0.7286103826294770	14651
**PAK3**	0.23312	3.5383	0.7286103826294770	14649

**PTPN20**	0.23312	1.6077	0.7286103826294770	14654
**ATRIP**	0.23293	1.7769	0.7292315656992480	14666
**CORIN**	0.23293	1.4662	0.7292315656992480	14670
**CPT1B**	0.23293	3.0192	0.7292315656992480	14657
**DCAF17**	0.23293	2.9553	0.7292315656992480	14659
**DISP2**	0.23293	1.515	0.7292315656992480	14669
**ELSPBP1**	0.23293	1.9412	0.7292315656992480	14663
**FAM160A2**	0.23293	0.33889	0.7292315656992480	14671
**FBXO2**	0.23293	2.9922	0.7292315656992480	14658
**HEPACAM2**	0.23293	2.9054	0.7292315656992480	14660
**HRAS**	0.23293	1.7364	0.7292315656992480	14667
**HSDL1**	0.23293	1.8634	0.7292315656992480	14665
**HSPH1**	0.23293	1.9286	0.7292315656992480	14664
**LETM2**	0.23293	1.6879	0.7292315656992480	14668
**PRDX2**	0.23293	2.6682	0.7292315656992480	14661
**ROPN1L**	0.23293	2.1035	0.7292315656992480	14662
**SNX1**	0.23293	3.2412	0.7292315656992480	14655
**UFSP2**	0.23293	3.2213	0.7292315656992480	14656
**VAT1**	0.23293	0.0097734	0.7292315656992480	14672
**OR11G2**	0.23252	2.7787	0.7305729732069420	14673
**UBE2D3**	0.7676	0.16536	0.7309658289857040	14674
**ANAPC13**	0.23235	2.0751	0.7311295521917350	14675
**CLEC2L**	0.76765	0.029104	0.7311295521917350	14676
**C1orf168**	0.76767	0.31234	0.7311950469617770	14677
**ANKRD6**	0.23228	2.868	0.7313587976110490	14678
**UBXN7**	0.23225	2.6854	0.7314570574143740	14679
**PRDM1**	0.23218	2.6287	0.731686357758337	14680
**ADPGK**	0.2321	2.0692	0.7319484624111320	14681
**GML**	0.2319	0.0013466	0.7326039441597190	14682
**TAS2R16**	0.2317	1.1337	0.7332597408276910	14683
**RBMXL1**	0.76845	0.26679	0.7337517953464080	14684
**CCDC62**	0.23139	3.2549	0.7342768493911040	14686

**SRL**	0.23139	3.2334	0.7342768493911040	14687
**TCP11L2**	0.23139	3.5884	0.7342768493911040	14685
**RNF182**	0.23128	2.4517	0.7346379414973230	14688
**MESP2**	0.23126	2.2842	0.7347036048986430	14689
**ARRDC3**	0.23104	1.6605	0.735426111528358	14696
**FAM107A**	0.23104	0.0040549	0.735426111528358	14701
**HOXD3**	0.23104	2.3527	0.735426111528358	14691
**IGDCC3**	0.23104	0.14522	0.735426111528358	14700
**P2RY2**	0.23104	1.6984	0.735426111528358	14695
**RNF26**	0.23104	0.63657	0.735426111528358	14699
**SLC17A4**	0.23104	1.5702	0.735426111528358	14697
**SNRPA**	0.23104	1.7485	0.735426111528358	14694
**SPNS1**	0.23104	2.733	0.735426111528358	14690
**THYN1**	0.23104	2.0955	0.735426111528358	14693
**YTHDF2**	0.23104	0.9879	0.735426111528358	14698
**ZBTB43**	0.23104	2.2252	0.735426111528358	14692
**FLNC**	0.23101	2.6441	0.7355246649026240	14702
**PRR14**	0.23065	2.5582	0.7367078632356310	14703
**KLK10**	0.23056	1.3045	0.7370038239745250	14704
**SAP25**	0.23035	2.9124	0.7376946502323130	14705
**MYLPF**	0.2303	1.5156	0.737859184565985	14706
**STX7**	0.76982	0.087553	0.73825414849242	14707
**LOC100130[illegible]**	0.7699	0.12875	0.7385175217680450	14708
**SYN2**	0.23008	1.4103	0.7385833730913360	14709
**ACADL**	0.77005	0.33536	0.739011484791282	14710
**PM20D1**	0.77007	0.082811	0.7390773601512470	14711
**BOC**	0.77025	0.17849	0.7396703827971310	14712
**ASB12**	0.22973	3.2307	0.7397362902583410	14713
**CDK15**	0.22971	3.3992	0.7398022009329720	14714
**DEF6**	0.22971	0.19063	0.7398022009329720	14718
**PHF8**	0.22971	0.57515	0.7398022009329720	14717
**TPBG**	0.22971	3.1767	0.7398022009329720	14715

**ZBED3**	0.22971	2.204	0.7398022009329720	14716
**NBN**	0.22964	1.1667	0.7400329136085200	14719
**CCAR2**	0.22959	2.4539	0.740197732496167	14720
**KIF2B**	0.22946	3.653	0.7406263557428990	14721
**CCNDBP1**	0.22938	2.1909	0.7408901915363070	14723
**DCDC2B**	0.22938	2.8921	0.7408901915363070	14722
**ZNF507**	0.22938	2.0298	0.7408901915363070	14724
**C2orf73**	0.22936	3.0934	0.740956158542993	14725
**MT4**	0.22931	2.419	0.7411210901683800	14726
**RGS13**	0.2293	3.5983	0.7411540789126900	14727
**KIF1C**	0.77079	0.31151	0.7414510139169050	14728
**DPEP2**	0.22915	2.6225	0.7416490069099080	14729
**PAH**	0.22907	0.80392	0.7419130428022490	14730
**ANK1**	0.229	2.0989	0.742144116643616	14737
**B3GAT3**	0.229	0.64505	0.742144116643616	14741
**EN1**	0.229	2.4893	0.742144116643616	14734
**FH**	0.229	2.4806	0.742144116643616	14735
**FXYD7**	0.229	2.5593	0.742144116643616	14732
**GLTPD2**	0.229	2.0804	0.742144116643616	14738
**HYAL4**	0.229	2.5761	0.742144116643616	14731
**KCNN1**	0.229	0.29001	0.742144116643616	14742
**KRTAP5-9**	0.229	2.5336	0.742144116643616	14733
**NLRP1**	0.229	2.2007	0.742144116643616	14736
**OR2A14**	0.229	0.89164	0.742144116643616	14740
**STAP1**	0.229	2.0131	0.742144116643616	14739
**EI24**	0.22898	2.4514	0.7422101450196720	14743
**FGD2**	0.22895	3.3671	0.7423091936514600	14744
**OR10S1**	0.22868	2.3602	0.7432009593206240	14745
**METTL2B**	0.22857	2.4425	0.7435644407318870	14746
**OSTF1**	0.22855	2.2894	0.7436305388156820	14747
**SLIT1**	0.77154	0.24652	0.7439280204085470	14748
**CTAGE8**	0.22841	1.6927	0.7440933164097420	14749

**ABCA4**	0.22838	0.96789	0.744192503769785	14750
**SETD1A**	0.22826	0.96231	0.7445893264506120	14751
**HIST2H2AA4**	0.22816	2.1727	0.7449201016042220	14752
**HIST2H2AB**	0.22779	3.6217	0.7461446791175580	14753
**AHCYL1**	0.2277	3.0558	0.7464427185153480	14754
**CLSTN1**	0.22767	1.8251	0.7465420797161590	14755
**GAP43**	0.22764	3.2935	0.7466414482878660	14756
**NRAP**	0.22754	0.3382	0.7469727301211250	14757
**MPI**	0.22752	3.3744	0.7470389963251730	14758
**KRT40**	0.77249	0.12114	0.7470721306573710	14759
**LSAMP**	0.22748	1.6112	0.7471715385755910	14760
**TEX13B**	0.22739	0.53992	0.747469806642873	14761
**HCAR2**	0.22738	3.1951	0.7475029516439280	14762
**MUC3B**	0.22736	0.44821	0.7475692441097770	14763
**ASF1B**	0.22734	2.9065	0.7476355398611270	14764
**GRIN2A**	0.2273	0.86162	0.7477681412227920	14765
**MRPL52**	0.77272	0.11525	0.7478344468343440	14766
**FUNDC2**	0.22724	2.4608	0.7479670679219720	14769
**LRRC8B**	0.22724	1.6542	0.7479670679219720	14770
**SLC17A8**	0.22724	2.5701	0.7479670679219720	14768
**TMEM74**	0.22724	0.9582	0.7479670679219720	14771
**ZCCHC12**	0.22724	2.8928	0.7479670679219720	14767
**KRTAP19-5**	0.22709	2.2091	0.7484645142188630	14772
**ARNT2**	0.77324	0.10319	0.7495595486211530	14773
**TMEM14C**	0.22674	1.0569	0.749625943225112	14774
**MSMB**	0.22664	3.3451	0.7499579658278330	14775
**CUEDC2**	0.22663	2.6264	0.7499911726350600	14778
**CYP7A1**	0.22663	2.963	0.7499911726350600	14777
**RNF19A**	0.22663	3.0154	0.7499911726350600	14776
**SLC35G4**	0.22663	2.321	0.7499911726350600	14779
**ZNF451**	0.22663	1.9738	0.7499911726350600	14780
**DYNLL1**	0.22617	0.07723	0.7515195810505540	14781

**GIP**	0.77385	0.29831	0.7515860734050850	14782
**ALOX5AP**	0.22613	3.8393	0.7516525690827210	14783
**MAP1LC3B2**	0.22608	3.5087	0.7518188228195020	14784
**ZNF585B**	0.22605	2.8168	0.751918585036834	14785
**MAP4K1**	0.22597	2.9139	0.7521846542107400	14786
**MYEOV**	0.22595	0.15135	0.7522511798245130	14787
**C11orf95**	0.22593	1.2352	0.7523177087676590	14792
**CORO6**	0.22593	2.1182	0.7523177087676590	14790
**HHIP**	0.22593	2.1725	0.7523177087676590	14789
**KRBOX1**	0.22593	2.4322	0.7523177087676590	14788
**NME3**	0.22593	1.7089	0.7523177087676590	14791
**PARVA**	0.22593	0.39887	0.7523177087676590	14794
**RFTN1**	0.22593	0.83364	0.7523177087676590	14793
**ZNF446**	0.22593	0.0009557	0.7523177087676590	14795
**ARID4A**	0.22588	3.3734	0.7524840456956520	14796
**CD160**	0.22586	1.7839	0.7525505862965490	14797
**RUNDC3B**	0.22578	1.5958	0.7528167820284170	14798
**GFY**	0.22572	1.7161	0.753016463839617	14799
**ASTE1**	0.2256	2.3083	0.7534159175672680	14802
**CLEC2D**	0.2256	3.4315	0.7534159175672680	14800
**HOXD8**	0.2256	0.58368	0.7534159175672680	14804
**KRTAP4-7**	0.2256	1.9688	0.7534159175672680	14803
**ZNF251**	0.2256	2.8612	0.7534159175672680	14801
**TMC5**	0.22553	1.3206	0.753648987766841	14805
**ST3GAL3**	0.22549	2.1805	0.7537821891205270	14806
**ASMTL**	0.77454	0.1012	0.753882098913124	14807
**MDGA1**	0.22527	2.0312	0.7545150357866050	14808
**ANKRD52**	0.22522	2.7609	0.7546816483616410	14809
**NRIP2**	0.22513	0.50846	0.7549816038034390	14810
**TMEM191B**	0.22499	3.3041	0.7554483362269390	14811
**VIM**	0.22494	3.4431	0.7556150662630730	14812
**ECHDC1**	0.22492	0.63077	0.7556817641591890	14813

**CD68**	0.22484	1.3891	0.7559485893690400	14814
**TCFL5**	0.22472	0.26128	0.756348928129957	14815
**C12orf43**	0.22468	1.4567	0.7564824013229400	14821
**C1orf159**	0.22468	1.6238	0.7564824013229400	14820
**CCL23**	0.22468	0.23068	0.7564824013229400	14822
**DNMBP**	0.22468	2.5665	0.7564824013229400	14816
**PSME1**	0.22468	2.361	0.7564824013229400	14817
**SPATA9**	0.22468	1.7426	0.7564824013229400	14819
**ZNF169**	0.22468	2.1899	0.7564824013229400	14818
**CLPP**	0.22459	3.2347	0.756782765294473	14823
**TMPRSS2**	0.22452	2.7262	0.7570164289275730	14824
**TPGS2**	0.22441	2.8611	0.7573836981697910	14825
**MAP6D1**	0.22436	2.68	0.757550672509667	14826
**DCLK3**	0.22414	0.088154	0.7582856106872050	14827
**CLUL1**	0.22411	0.43513	0.7583858612619200	14828
**TRIM8**	0.22404	2.8062	0.7586198089163620	14830
**ZNF790**	0.22404	3.4965	0.7586198089163620	14829
**GRTP1**	0.22365	0.87426	0.7599239925445820	14831
**PGBD2**	0.22354	2.4831	0.7602920729296370	14832
**FBXL3**	0.22352	3.6137	0.7603590077028020	14833
**TUBD1**	0.22352	3.1065	0.7603590077028020	14834
**C16orf82**	0.77658	0.10058	0.7606937326833070	14835
**DMC1**	0.22339	3.2122	0.7607941667976150	14836
**AMN1**	0.22333	2.4197	0.7609950580525980	14838
**AP2A1**	0.22333	2.4665	0.7609950580525980	14837
**GPR162**	0.22333	1.3207	0.7609950580525980	14840
**LGALS7B**	0.22333	1.1317	0.7609950580525980	14842
**NXPE4**	0.22333	0.4611	0.7609950580525980	14844
**PRRC2A**	0.22333	1.6479	0.7609950580525980	14839
**SP9**	0.22333	0.11181	0.7609950580525980	14845
**SPATA46**	0.22333	1.3166	0.7609950580525980	14841
**ZNF322**	0.22333	0.85338	0.7609950580525980	14843

**PPP2R5D**	0.22324	2.8376	0.7612964525337970	14846
**TPRKB**	0.22319	0.57331	0.7614639237971620	14847
**KCNJ8**	0.22317	1.7828	0.7615309182825200	14848
**MTRNR2L3**	0.22314	1.0327	0.7616314164197440	14851
**SKIV2L**	0.22314	2.3085	0.7616314164197440	14849
**TSPAN11**	0.22314	1.8564	0.7616314164197440	14850
**MAP3K14**	0.22306	3.2323	0.7618994490683850	14852
**ASAH2**	0.22305	0.67335	0.7619329569980230	14853
**ATP8A1**	0.22303	2.8901	0.7619999754238980	14854
**EFNA4**	0.22248	2.6222	0.7638443256520700	14855
**CABS1**	0.22244	2.623	0.7639785615486600	14856
**RGL2**	0.22239	2.4326	0.7641463757809130	14857
**VN1R1**	0.22229	0.05187	0.764482068822745	14858
**TARBP2**	0.22227	2.3128	0.7645492177690600	14859
**NUTM1**	0.22204	1.5637	0.7653216786071670	14860
**FCN1**	0.22202	2.0683	0.7653888706946730	14862
**GSK3B**	0.22202	1.7914	0.7653888706946730	14864
**KRT1**	0.22202	0.079135	0.7653888706946730	14868
**OR52J3**	0.22202	1.3684	0.7653888706946730	14866
**RPL10A**	0.22202	0.22594	0.7653888706946730	14867
**TMPRSS7**	0.22202	1.8747	0.7653888706946730	14863
**TOP3B**	0.22202	2.2832	0.7653888706946730	14861
**XAF1**	0.22202	1.7696	0.7653888706946730	14865
**ALS2CR12**	0.22201	1.3502	0.7654224680342860	14869
**FAM205C**	0.2217	3.1976	0.7664644145235240	14870
**LOC286238**	0.2217	2.4293	0.7664644145235240	14873
**PEX6**	0.2217	2.6737	0.7664644145235240	14872
**SPIRE1**	0.2217	2.8226	0.7664644145235240	14871
**C15orf65**	0.22166	3.3788	0.7665989198400220	14874
**DNASE1L1**	0.22166	2.8229	0.7665989198400220	14875
**EFS**	0.22166	2.7915	0.7665989198400220	14876
**RARRES3**	0.22166	1.7234	0.7665989198400220	14877

**USP20**	0.22165	3.1588	0.7666325483362030	14878
**ALDH1A2**	0.22146	1.7583	0.7672716545858500	14879
**CCNI**	0.22135	2.4519	0.7676418067435570	14880
**CPEB3**	0.22132	0.29341	0.7677427755863520	14881
**ACTA1**	0.22104	1.3961	0.7686855292135450	14884
**ARSG**	0.22104	1.6753	0.7686855292135450	14883
**CELF1**	0.22104	1.9901	0.7686855292135450	14882
**FLVCR1**	0.22104	0.40803	0.7686855292135450	14886
**PIP5K1C**	0.22104	1.3954	0.7686855292135450	14885
**SQSTM1**	0.22104	0.10953	0.7686855292135450	14887
**C16orf95**	0.22102	0.22456	0.768752894898325	14888
**ANKRD9**	0.221	2.3622	0.7688202640719900	14889
**SGPP2**	0.77911	0.35446	0.7691908569207990	14890
**DNPEP**	0.22088	3.2849	0.7692245524179490	14891
**CAPN11**	0.22086	2.3995	0.7692919460325030	14892
**TIMM17B**	0.22083	1.9519	0.769393043006218	14893
**VAT1L**	0.22074	0.57786	0.7696963811220050	14894
**CLASP1**	0.77937	0.15639	0.770067223915745	14895
**SLC23A1**	0.2206	2.2678	0.7701683812399110	14896
**STAT1**	0.22056	1.1304	0.7703032699327850	14897
**TTR**	0.22046	3.1944	0.7706405530014920	14898
**ZNF740**	0.22037	0.15271	0.7709441827365320	14899
**SHPK**	0.22035	2.6516	0.7710116656631280	14900
**STEAP1B**	0.22018	1.5964	0.7715854123769850	14901
**FGF10**	0.22014	1.9244	0.77172044851749	14902
**CYP2S1**	0.22011	1.1592	0.7718217348583740	14904
**PPOX**	0.22011	3.4554	0.7718217348583740	14903
**APAF1**	0.22004	0.23042	0.7720581004525680	14911
**AWAT2**	0.22004	0.59394	0.7720581004525680	14909
**CCS**	0.22004	2.0323	0.7720581004525680	14907
**FGF21**	0.22004	1.0713	0.7720581004525680	14908
**HNF1B**	0.22004	0.46279	0.7720581004525680	14910

**QPRT**	0.22004	2.7156	0.7720581004525680	14905
**RTN4RL1**	0.22004	2.6709	0.7720581004525680	14906
**TPSAB1**	0.22004	0.10623	0.7720581004525680	14912
**ZPBP2**	0.21987	3.1629	0.7726323108492980	14913
**TTC37**	0.21982	2.4185	0.7728012447449240	14914
**CAPN14**	0.21981	0.86567	0.7728350341706220	14915
**FAM183A**	0.21952	1.5393	0.77381531169879	14916
**BRS3**	0.2194	2.5284	0.7742211613337070	14921
**CCNT1**	0.2194	0.12593	0.7742211613337070	14926
**FAM228A**	0.2194	0.7383	0.7742211613337070	14924
**LAS1L**	0.2194	3.5515	0.7742211613337070	14917
**MROH7**	0.2194	1.869	0.7742211613337070	14923
**OVOS2**	0.2194	3.2493	0.7742211613337070	14918
**PHOX2B**	0.2194	2.4172	0.7742211613337070	14922
**PRKAG2**	0.2194	2.7308	0.7742211613337070	14920
**RCSD1**	0.2194	0.42341	0.7742211613337070	14925
**SFXN3**	0.2194	2.8284	0.7742211613337070	14919
**RNASE6**	0.21918	0.2657	0.7749655504187500	14927
**SEC24A**	0.78111	0.24245	0.7759474472102300	14928
**CABIN1**	0.21883	2.5783	0.7761506916750420	14930
**FAM173B**	0.21883	0.068867	0.7761506916750420	14934
**KCNS2**	0.21883	2.2729	0.7761506916750420	14931
**NQO2**	0.21883	2.9106	0.7761506916750420	14929
**PDE7B**	0.21883	2.2497	0.7761506916750420	14932
**PSMA4**	0.21883	2.1744	0.7761506916750420	14933
**KRTAP15-1**	0.21873	3.4546	0.7764895037178270	14935
**MAGEB16**	0.21871	2.8907	0.7765572768227740	14936
**TNNT3**	0.21868	1.2749	0.7766589431686560	14937
**CYP39A1**	0.21866	2.0684	0.7767267251925540	14940
**EIF4E2**	0.21866	0.079817	0.7767267251925540	14942
**PPP1R3D**	0.21866	2.4555	0.7767267251925540	14939
**SERPINB10**	0.21866	1.9555	0.7767267251925540	14941

**THAP3**	0.21866	3.7087	0.7767267251925540	14938
**EIF1B**	0.21856	1.3163	0.7770656888575310	14943
**STAP2**	0.21854	2.9627	0.7771334923044380	14944
**KIF27**	0.21814	2.6218	0.7784903124700750	14945
**DCTPP1**	0.21809	1.1394	0.7786600157536400	14946
**GRIN3B**	0.21795	1.8462	0.7791353042977820	14947
**SWI5**	0.78209	0.36126	0.7792711333538890	14949
**ZADH2**	0.21791	2.2224	0.7792711333538890	14948
**PRG3**	0.21787	2.2194	0.7794069767887020	14950
**BDH2**	0.21768	1.3951	0.7800524296339540	14955
**C12orf57**	0.21768	2.5122	0.7800524296339540	14953
**CHI3L1**	0.21768	0.96911	0.7800524296339540	14957
**DMRT3**	0.21768	0.20992	0.7800524296339540	14959
**DRAP1**	0.21768	3.3036	0.7800524296339540	14951
**EPB41L5**	0.21768	1.1355	0.7800524296339540	14956
**TM4SF19**	0.21768	2.2847	0.7800524296339540	14954
**ZBTB7C**	0.21768	0.29694	0.7800524296339540	14958
**ZNF559-ZNI**	0.21768	2.5932	0.7800524296339540	14952
**AWAT1**	0.21767	1.7073	0.7800864098377880	14960
**C16orf89**	0.7827	0.12016	0.7813443113442550	14961
**SRSF2**	0.21729	0.80502	0.7813783258379830	14963
**ZC3H14**	0.21729	2.5047	0.7813783258379830	14962
**NUP88**	0.2172	0.87887	0.7816844969750690	14964
**ADCY6**	0.21696	3.2899	0.782501311836487	14965
**KERA**	0.21696	3.1793	0.782501311836487	14966
**MLLT11**	0.21696	2.3824	0.782501311836487	14967
**BHLHE40**	0.78305	0.10438	0.7825353571176170	14968
**ATP6V1B2**	0.21689	1.205	0.782739647855433	14969
**PRMT8**	0.21679	2.9927	0.7830802050336680	14970
**SLC46A2**	0.21676	0.51735	0.7831823898979530	14971
**SLCO4A1**	0.21673	3.7505	0.7832845829406810	14972
**CCDC88C**	0.21669	3.1463	0.7834208530571190	14973

**FAM166A**	0.21665	0.83149	0.7835571377228730	14974
**COMMD4**	0.21664	2.1904	0.7835912111633040	14977
**ENPP2**	0.21664	2.2927	0.7835912111633040	14976
**NET1**	0.21664	0.801	0.7835912111633040	14981
**RNF123**	0.21664	1.3634	0.7835912111633040	14980
**SERPINB8**	0.21664	1.8143	0.7835912111633040	14979
**SLC27A1**	0.21664	2.0061	0.7835912111633040	14978
**VSIG10L2**	0.21664	2.5573	0.7835912111633040	14975
**GDF9**	0.2166	2.7361	0.783727514023636	14982
**TNIK**	0.21657	1.3088	0.7838297507248950	14983
**DPCR1**	0.21625	2.4196	0.7849207858349000	14984
**APIP**	0.21611	3.0102	0.7853984075811580	14985
**PKN2**	0.2161	2.8984	0.7854325302757470	14986
**C15orf53**	0.21602	2.7568	0.7857055447636780	14987
**NMB**	0.78398	0.54387	0.7857055447636780	14988
**DNALI1**	0.21595	0.49018	0.7859444804901890	14989
**RPL26L1**	0.2159	2.5934	0.7861151763409440	14990
**SRD5A3**	0.78412	0.03986	0.786183461094908	14991
**ROM1**	0.21583	1.6124	0.7863541890202040	14992
**PKDCC**	0.21582	0.96911	0.786388337355756	14993
**SPR**	0.2157	3.2062	0.7867981889368850	14994
**AKAP3**	0.21548	2.4182	0.7875499269861890	14999
**EGFL7**	0.21548	2.4709	0.7875499269861890	14996
**FAM19A5**	0.21548	2.521	0.7875499269861890	14995
**HCST**	0.21548	2.1985	0.7875499269861890	15001
**OR4D2**	0.21548	2.4416	0.7875499269861890	14997
**PDE1B**	0.21548	2.1776	0.7875499269861890	15002
**TCTN3**	0.21548	2.426	0.7875499269861890	14998
**TMEM132E**	0.21548	2.3196	0.7875499269861890	15000
**TMEM201**	0.21548	2.1225	0.7875499269861890	15003
**VOPP1**	0.21528	2.0499	0.7882337116189860	15004
**KIR3DL3**	0.21526	1.0688	0.7883021103514750	15005

**HOMER2**	0.21515	2.6473	0.7886783693256220	15007
**MRPL39**	0.21515	0.60042	0.7886783693256220	15008
**RPP21**	0.21515	3.1445	0.7886783693256220	15006
**ITSN1**	0.21473	2.5484	0.7901160228696870	15009
**RNASE1**	0.21471	0.92177	0.7901845232892550	15010
**ZNF341**	0.21468	0.87513	0.7902872808713110	15011
**ADM2**	0.21459	2.8201	0.7905956036997230	15012
**TSPYL4**	0.21457	2.611	0.7906641300903960	15013
**FRA10AC1**	0.21449	3.1838	0.790938272790861	15014
**ANKRD35**	0.78552	0.19982	0.7909725448079130	15015
**JPH3**	0.21444	2.4924	0.7911096421678100	15016
**FOSL2**	0.21429	2.2795	0.7916238897601630	15017
**TBC1D7**	0.21427	2.9063	0.7916924719227620	15018
**LY6G5B**	0.21414	3.1376	0.7921383467831100	15019
**C21orf33**	0.21411	1.6493	0.7922412633463700	15025
**DNHD1**	0.21411	2.4015	0.7922412633463700	15024
**NDUFA6**	0.21411	2.4418	0.7922412633463700	15023
**OTOG**	0.21411	3.0296	0.7922412633463700	15020
**PCDHGA6**	0.21411	2.7445	0.7922412633463700	15021
**TCEAL7**	0.21411	0.15801	0.7922412633463700	15026
**TIMP4**	0.21411	2.525	0.7922412633463700	15022
**DCUN1D2**	0.21406	2.7941	0.7924128096018100	15027
**C16orf90**	0.21401	1.8478	0.7925843791796280	15028
**CORO2B**	0.21394	1.3716	0.7928246157918660	15029
**GNL2**	0.78606	0.33362	0.7928246157918660	15030
**TIGD6**	0.21389	3.4745	0.7929962413898950	15031
**MYLK4**	0.78618	0.27236	0.7932365564764230	15033
**ZNF496**	0.21382	2.634	0.7932365564764230	15032
**DRD5**	0.21378	0.67218	0.7933738999528800	15035
**KDELC1**	0.21378	3.0146	0.7933738999528800	15034
**TMEM167A**	0.21366	2.8701	0.7937860202089930	15036
**USP25**	0.78647	0.24385	0.7942326360140640	15037

**DDX1**	0.21347	1.5328	0.7944388198246930	15040
**MAN2A2**	0.21347	3.4706	0.7944388198246930	15039
**OR2AE1**	0.21347	3.5984	0.7944388198246930	15038
**OTULIN**	0.78661	0.27435	0.794713784119984	15041
**SEC31B**	0.21314	0.07978	0.7955734351064710	15042
**SGO2**	0.21313	2.2279	0.7956078333736210	15043
**RCN2**	0.78688	0.0006700	0.7956422325821920	15044
**ESX1**	0.78695	0.20268	0.7958830534097270	15045
**METTL2A**	0.21302	3.005	0.7959862764663560	15046
**PSMG3**	0.21284	0.3753	0.7966057930139200	15047
**PAX6**	0.21277	0.09758	0.7968467986948870	15048
**PNP**	0.21257	0.3598	0.7975356415455650	15049
**MT1G**	0.21248	2.6617	0.7978457443158920	15051
**THUMPD1**	0.21248	2.5047	0.7978457443158920	15052
**TIMD4**	0.21248	2.797	0.7978457443158920	15050
**MELK**	0.21239	3.4222	0.7981559238289940	15053
**ATCAY**	0.21236	2.1015	0.7982593340657030	15056
**CWF19L2**	0.21236	0.17801	0.7982593340657030	15058
**ETFA**	0.21236	0.42461	0.7982593340657030	15057
**FABP2**	0.21236	2.8921	0.7982593340657030	15054
**JAKMIP3**	0.21236	2.2456	0.7982593340657030	15055
**LRRC7**	0.21217	1.8939	0.7989144639175350	15059
**RAB7B**	0.21215	3.112	0.7989834449004140	15060
**GINS2**	0.21207	2.2553	0.7992594068601190	15061
**ECI2**	0.21203	2.2082	0.7993974106673550	15062
**POLD1**	0.21195	1.177	0.7996734639665250	15063
**F11R**	0.21182	2.4601	0.8001221806000640	15064
**ASF1A**	0.21144	1.8262	0.8014347386409220	15065
**CENPT**	0.21139	3.3097	0.8016075464319730	15068
**DAZAP1**	0.21139	3.2047	0.8016075464319730	15071
**DEFB123**	0.21139	2.6998	0.8016075464319730	15074
**GADD45GIP**	0.21139	3.2733	0.8016075464319730	15069

**GZMH**	0.21139	3.7326	0.8016075464319730	15066
**INIP**	0.21139	2.8962	0.8016075464319730	15073
**IQCA1**	0.21139	2.6818	0.8016075464319730	15075
**LGALS1**	0.21139	2.9496	0.8016075464319730	15072
**OSBPL2**	0.21139	3.5509	0.8016075464319730	15067
**PLAC9**	0.21139	3.2239	0.8016075464319730	15070
**UBXN2A**	0.21139	0.90894	0.8016075464319730	15076
**SEC11A**	0.21128	1.0154	0.8019878078624000	15077
**TMA16**	0.2112	2.3254	0.8022644344834100	15078
**NFKBIA**	0.21077	2.8003	0.8037523558184720	15079
**SLC14A2**	0.21077	0.31341	0.8037523558184720	15084
**SMIM7**	0.21077	1.5038	0.8037523558184720	15083
**SOHLH1**	0.21077	1.6448	0.8037523558184720	15082
**TCP11X2**	0.21077	1.668	0.8037523558184720	15081
**WDR41**	0.21077	1.9982	0.8037523558184720	15080
**FADS2**	0.21075	1.7677	0.8038216047622820	15085
**PRKG1**	0.21069	2.9309	0.8040293747259870	15086
**OCIAD1**	0.21063	2.8326	0.8042371794041000	15087
**PM20D2**	0.21062	3.1917	0.8042718168935750	15088
**ZKSCAN4**	0.21054	1.4465	0.8045489515558050	15089
**MALL**	0.21048	2.4474	0.8047568431100060	15090
**HGC6.3**	0.78961	0.08899	0.8050687456724510	15091
**KIF21B**	0.21034	2.8557	0.8052420587196620	15092
**CYP3A7-CY**	0.2103	3.6267	0.8053807265741970	15093
**SDHC**	0.21014	0.21443	0.8059355529356810	15094
**RBMX2**	0.20997	1.0839	0.8065253279114710	15096
**SLC7A7**	0.20997	2.1993	0.8065253279114710	15095
**DPP8**	0.79007	0.052772	0.806664139266026	15097
**C20orf144**	0.20992	3.1353	0.8066988445333330	15098
**ACTRT1**	0.20967	2.2507	0.8075667922557070	15100
**BTNL2**	0.20967	1.2741	0.8075667922557070	15103
**C15orf41**	0.20967	1.8406	0.8075667922557070	15101

**CEBPG**	0.20967	0.94807	0.8075667922557070	15104
**CLEC2A**	0.20967	2.7893	0.8075667922557070	15099
**KIR2DS4**	0.20967	0.031128	0.8075667922557070	15106
**POMZP3**	0.20967	1.6767	0.8075667922557070	15102
**TMEM17**	0.20967	0.40548	0.8075667922557070	15105
**TRADD**	0.79039	0.29586	0.8077751902406700	15107
**UBQLN3**	0.20959	1.6803	0.8078446640318120	15108
**RHOT1**	0.20957	3.2823	0.8079141417223210	15109
**PLK1**	0.20955	3.1525	0.8079836233129680	15110
**MAP1S**	0.20952	3.943	0.8080878530133920	15111
**TOPORS**	0.20942	0.30677	0.8084353487714930	15112
**MDK**	0.20928	0.90749	0.8089220069042110	15113
**TNFRSF10A**	0.20926	0.68596	0.8089915451338830	15114
**LRRC18**	0.20923	3.3151	0.8090958598139150	15115
**WBSCR17**	0.20921	1.9728	0.8091654078254160	15116
**YIF1B**	0.20916	2.166	0.8093392949800180	15117
**ZFP3**	0.20913	0.13878	0.8094436390201040	15118
**KRTAP6-2**	0.2091	1.9264	0.8095479918738920	15119
**CACFD1**	0.79092	0.087602	0.8096175653408810	15120
**ZKSCAN2**	0.20873	0.1339	0.8108357361186510	15121
**TMEM230**	0.20866	0.59874	0.811079514670593	15122
**CYYR1-AS1**	0.20857	2.365	0.8113930150817300	15124
**LONP2**	0.20857	1.7162	0.8113930150817300	15126
**PLEKHM2**	0.20857	2.4433	0.8113930150817300	15123
**TAS2R46**	0.20857	0.87179	0.8113930150817300	15127
**TGFBR3L**	0.20857	2.343	0.8113930150817300	15125
**OR10G2**	0.20856	1.8564	0.8114278533832640	15128
**PGK 1.00**	0.20852	2.2217	0.8115672164390160	15129
**CARD14**	0.20852	3.5662	0.8115672164390160	15130
**TACO1**	0.20851	3.699	0.8116020596658460	15131
**DEPDC1B**	0.20817	2.0356	0.8127873163055160	15136
**GPT2**	0.20817	2.003	0.8127873163055160	15137

**INS-IGF2**	0.20817	2.6468	0.8127873163055160	15134
**NAA15**	0.20817	2.4874	0.8127873163055160	15135
**PCDHGA7**	0.20817	3.0637	0.8127873163055160	15133
**PECAM1**	0.20817	0.82399	0.8127873163055160	15138
**RAB8B**	0.20817	3.5987	0.8127873163055160	15132
**OR1J2**	0.20805	3.2977	0.8132059148587730	15139
**ANXA2R**	0.20768	2.5229	0.8144974918005530	15140
**CTRB2**	0.20749	0.99838	0.8151612622587960	15141
**USP54**	0.20747	2.8853	0.8152311537253700	15142
**CXorf38**	0.20745	2.3269	0.815301049174424	15143
**METAP2**	0.20745	2.2839	0.815301049174424	15144
**PCDHGC3**	0.20745	2.035	0.815301049174424	15145
**HDGF**	0.2074	1.3447	0.8154758052256260	15146
**CPA6**	0.2073	2.5054	0.815825392064173	15147
**GJB6**	0.20722	3.4236	0.8161051333366770	15148
**PSMA3**	0.79295	0.40137	0.8166997957025950	15149
**GCKR**	0.207	1.1507	0.8168747513658480	15151
**ZSWIM7**	0.207	2.0175	0.8168747513658480	15150
**BAG2**	0.20697	2.7572	0.816979736766681	15153
**SYT15**	0.20697	1.5529	0.816979736766681	15154
**ZC3H7B**	0.20697	3.2197	0.816979736766681	15152
**VN1R2**	0.2068	0.090258	0.8175748242207050	15155
**KYAT3**	0.79328	0.059818	0.8178549655765030	15156
**PRAMEF7**	0.20671	3.3484	0.8178899877589330	15157
**CCDC63**	0.20638	1.8745	0.8190462831836410	15158
**ERP29**	0.20638	1.245	0.8190462831836410	15160
**HNRNPM**	0.20638	0.73514	0.8190462831836410	15162
**LOC149373**	0.20638	1.8374	0.8190462831836410	15159
**SCN8A**	0.20638	0.55892	0.8190462831836410	15163
**TP53I13**	0.20638	0.77057	0.8190462831836410	15161
**C14orf93**	0.20637	2.3698	0.8190813395317630	15171
**C6orf203**	0.20637	3.1402	0.8190813395317630	15166

**CKLF-CMTM**	0.20637	2.8563	0.8190813395317630	15169
**DGCR8**	0.20637	0.47159	0.8190813395317630	15173
**DLX6**	0.20637	1.1781	0.8190813395317630	15172
**EPHA2**	0.20637	3.4659	0.8190813395317630	15164
**KIF7**	0.20637	3.1978	0.8190813395317630	15165
**POLA2**	0.20637	3.1236	0.8190813395317630	15168
**PPP1R16B**	0.20637	2.4129	0.8190813395317630	15170
**ZNF234**	0.20637	3.1337	0.8190813395317630	15167
**HPRT1**	0.20583	2.3702	0.8209758798368310	15174
**EIF3A**	0.20577	2.5424	0.8211865662017980	15175
**MRPL41**	0.79423	0.27025	0.8211865662017980	15176
**CUEDC1**	0.20553	2.525	0.8220296764587690	15177
**CDC42EP3**	0.20541	2.2864	0.8224514507736700	15179
**FBXL8**	0.20541	1.9306	0.8224514507736700	15181
**LHX1**	0.20541	3.0222	0.8224514507736700	15178
**PCDHGA11**	0.20541	1.932	0.8224514507736700	15180
**MAF 1.00**	0.20537	2.504	0.8225920747275800	15182
**SIX4**	0.20534	2.9212	0.8226975533689920	15183
**OSBPL5**	0.20532	2.216	0.8227678775486030	15184
**EMP1**	0.2053	3.0215	0.8228382057974350	15185
**ZNF287**	0.20497	0.56132	0.8239992101270140	15186
**AKAIN1**	0.20476	0.81418	0.8247386096794780	15190
**FBF1**	0.20476	0.88961	0.8247386096794780	15189
**GLI1**	0.20476	3.2039	0.8247386096794780	15187
**TDRD5**	0.20476	3.1448	0.8247386096794780	15188
**CYTIP**	0.2044	2.2191	0.8260072019578020	15194
**DBI**	0.2044	2.1689	0.8260072019578020	15195
**DCHS1**	0.2044	2.4387	0.8260072019578020	15193
**KIAA1614**	0.2044	3.6275	0.8260072019578020	15191
**MAF**	0.2044	1.8571	0.8260072019578020	15198
**PSMD8**	0.2044	2.1372	0.8260072019578020	15196
**SCRG1**	0.2044	3.1136	0.8260072019578020	15192

**SPATA3**	0.2044	1.8753	0.8260072019578020	15197
**IFT88**	0.20428	2.5742	0.826430361524861	15199
**SPACA4**	0.2042	3.3668	0.826712550133826	15200
**TOMM70**	0.2042	3.0963	0.826712550133826	15201
**PWWP2B**	0.79594	0.13505	0.827206538675326	15202
**SLC25A26**	0.20399	2.7704	0.8274536086561600	15203
**SPOCK3**	0.20388	0.66997	0.8278419635700160	15204
**VPS72**	0.20387	4.1128	0.8278772747532280	15205
**NOP10**	0.20382	1.8476	0.8280538461557650	15206
**KCNMB4**	0.79627	0.20976	0.8283717397564430	15207
**B3GNT9**	0.20321	2.3525	0.8302101002562460	15212
**CAMK2B**	0.20321	2.6802	0.8302101002562460	15208
**NR6A1**	0.20321	2.3789	0.8302101002562460	15211
**NRXN1**	0.20321	2.137	0.8302101002562460	15213
**PSTK**	0.20321	1.0256	0.8302101002562460	15215
**SAFB2**	0.20321	2.5992	0.8302101002562460	15209
**SELENON**	0.20321	0.90314	0.8302101002562460	15216
**SPRYD3**	0.20321	2.5301	0.8302101002562460	15210
**YWHAZ**	0.20321	0.48197	0.8302101002562460	15217
**ZUFSP**	0.20321	1.5098	0.8302101002562460	15214
**ALDH2**	0.20306	2.7047	0.8307409181074930	15223
**ANKRD34B**	0.20306	3.1816	0.8307409181074930	15220
**CHCHD5**	0.20306	2.0897	0.8307409181074930	15225
**FAM57A**	0.20306	1.0538	0.8307409181074930	15226
**GLOD4**	0.20306	2.5972	0.8307409181074930	15224
**OR10A6**	0.20306	3.234	0.8307409181074930	15219
**SLC35E4**	0.20306	3.24	0.8307409181074930	15218
**SPIN2A**	0.20306	3.0001	0.8307409181074930	15222
**TNNI2**	0.20306	3.1375	0.8307409181074930	15221
**ALPK2**	0.20299	3.3491	0.830988713224305	15227
**COPB2**	0.20299	3.1107	0.830988713224305	15228
**IGSF5**	0.20265	1.343	0.8321930161189800	15229

**IFNA17**	0.20242	4.1523	0.8330083764381510	15231
**NACA2**	0.20242	4.1523	0.8330083764381510	15230
**TMEM139**	0.20238	1.06	0.8331502347657400	15232
**POU4F2**	0.20224	0.9689	0.8336468710058940	15233
**ADGRE2**	0.20221	1.2163	0.8337533198096180	15234
**AK9**	0.20214	1.4648	0.8340017371003230	15235
**HOPX**	0.20212	1.8275	0.8340727229226030	15236
**TESPA1**	0.20179	0.20919	0.835244596455501	15237
**PFKM**	0.20177	3.4041	0.8353156559562000	15238
**TMEM160**	0.20174	3.7119	0.8354222531165170	15239
**ALKAL1**	0.20169	3.8137	0.8355999361489280	15240
**ATP13A3**	0.20169	2.5904	0.8355999361489280	15245
**BHLHA15**	0.20169	3.4343	0.8355999361489280	15241
**GRAMD3**	0.20169	2.7242	0.8355999361489280	15244
**KRTAP6-1**	0.20169	2.9342	0.8355999361489280	15243
**TTBK1**	0.20169	3.185	0.8355999361489280	15242
**C3orf30**	0.20166	1.4789	0.8357065586322360	15250
**ERVV-2**	0.20166	1.9751	0.8357065586322360	15249
**HINT1**	0.20166	2.009	0.8357065586322360	15248
**MRPL48**	0.20166	2.1846	0.8357065586322360	15247
**PFDN4**	0.20166	0.54223	0.8357065586322360	15251
**SFI1**	0.20166	2.8133	0.8357065586322360	15246
**ACBD3**	0.20151	0.74715	0.8362398136286100	15252
**KLHL9**	0.20111	2.819	0.8376629910078320	15253
**IL4R**	0.20105	2.8675	0.8378766139541200	15254
**HOXC12**	0.20103	0.03065	0.837947830100194	15255
**PGM2L1**	0.2008	2.7071	0.8387671214748430	15256
**GPRC5B**	0.20064	2.8081	0.8393373955098660	15257
**ABCA8**	0.20035	1.8358	0.840371713575642	15261
**CREG1**	0.20035	1.8971	0.840371713575642	15260
**EDEM1**	0.20035	2.2959	0.840371713575642	15258
**KDM1A**	0.20035	2.0498	0.840371713575642	15259

**MAPKAP1**	0.20035	0.38151	0.840371713575642	15263
**SMU1**	0.20035	1.7316	0.840371713575642	15262
**CARD10**	0.20034	3.4003	0.8404073957496580	15264
**FDX1L**	0.20034	2.8718	0.8404073957496580	15266
**IDUA**	0.20034	2.8875	0.8404073957496580	15265
**KLK7**	0.20034	1.2486	0.8404073957496580	15268
**LGALS7**	0.20034	2.6397	0.8404073957496580	15267
**IL1B**	0.19983	3.5796	0.8422286079408100	15269
**NOXRED1**	0.19968	4.2282	0.8427647903838370	15270
**C10orf126**	0.8005	0.17618	0.843408529312562	15271
**ATP1B4**	0.19941	3.0461	0.8437305298732020	15273
**HSPB7**	0.19941	2.7591	0.8437305298732020	15274
**MGP**	0.19941	3.7768	0.8437305298732020	15272
**DSP**	0.80068	0.14627	0.8440526179391950	15275
**LOC1019297**	0.1992	2.8255	0.8444822049533140	15277
**NCF1**	0.1992	3.0586	0.8444822049533140	15276
**RGMA**	0.1992	0.88652	0.8444822049533140	15279
**SAMD12**	0.1992	1.6972	0.8444822049533140	15278
**SELENOH**	0.1992	0.67942	0.8444822049533140	15280
**IFNA6**	0.19884	0.51808	0.8457719021747970	15281
**PLCD4**	0.80124	0.096892	0.8460586926272840	15282
**FBXO47**	0.19868	2.9175	0.8463455526838760	15283
**C1orf162**	0.19862	3.6099	0.8465607434385470	15285
**CNIH3**	0.19862	3.6969	0.8465607434385470	15284
**DEFB135**	0.19862	1.4604	0.8465607434385470	15291
**DHX58**	0.19862	2.9057	0.8465607434385470	15288
**DMXL2**	0.19862	3.5093	0.8465607434385470	15286
**ITGB7**	0.19862	0.78412	0.8465607434385470	15292
**LVRN**	0.19862	2.9464	0.8465607434385470	15287
**RTCA**	0.19862	2.4429	0.8465607434385470	15289
**TAS2R43**	0.19862	2.3985	0.8465607434385470	15290
**TEFM**	0.19857	4.2647	0.8467400990176110	15293

**C16orf59**	0.80148	0.19326	0.8469194818390800	15294
**TRMT61A**	0.19816	1.0649	0.8482118436228500	15295
**PLD3**	0.19812	3.7689	0.848355526825611	15296
**RASSF1**	0.80192	0.23506	0.848499227544673	15297
**DAOA**	0.19803	3.2042	0.8486788780868290	15298
**ELOVL4**	0.80198	0.041567	0.8487148114821680	15299
**PATE3**	0.19792	2.5951	0.8490742057287840	15300
**CLCN2**	0.19787	1.6757	0.849253943983771	15303
**FASTKD2**	0.19787	3.703	0.849253943983771	15301
**HMGCL**	0.19787	3.3908	0.849253943983771	15302
**NEUROG3**	0.19787	0.67837	0.849253943983771	15305
**WSB1**	0.19787	1.6173	0.849253943983771	15304
**EPAS1**	0.19779	3.0653	0.8495415822729030	15307
**FAM76B**	0.19779	2.3143	0.8495415822729030	15309
**PDHX**	0.19779	2.5699	0.8495415822729030	15308
**PRR11**	0.19779	2.3052	0.8495415822729030	15310
**SHROOM2**	0.19779	3.6784	0.8495415822729030	15306
**THEMIS**	0.19779	0.39803	0.8495415822729030	15311
**RFT1**	0.19773	0.92352	0.8497573571239640	15312
**RIBC1**	0.1977	1.1678	0.849865259387042	15313
**MFNG**	0.19763	3.1173	0.8501170698231720	15314
**POTEC**	0.19761	2.4601	0.8501890255633210	15315
**TNKS2**	0.19756	3.0632	0.8503689341754350	15316
**SNAI3**	0.19754	2.2807	0.8504409053273690	15317
**DNAJC22**	0.19725	3.5893	0.8514849825510800	15318
**FSTL4**	0.19713	3.5189	0.8519172860946580	15319
**NDUFAF8**	0.19713	3.3531	0.8519172860946580	15320
**TFIP11**	0.19713	3.3214	0.8519172860946580	15321
**TRAPPC5**	0.19713	0.44854	0.8519172860946580	15322
**YIPF3**	0.19707	3.499	0.8521334975803910	15323
**SMIM19**	0.19665	2.5668	0.8536480949562660	15324
**CAMK1D**	0.19664	3.1768	0.8536841806633530	15325

**TBCEL**	0.19658	2.9247	0.8539007182552390	15326
**AQP9**	0.19649	2.2356	0.8542255997362090	15329
**DPYSL4**	0.19649	1.1797	0.8542255997362090	15332
**HM13**	0.19649	1.499	0.8542255997362090	15331
**PCBP1**	0.19649	2.6526	0.8542255997362090	15328
**USP6NL**	0.19649	2.6875	0.8542255997362090	15327
**ZMIZ1**	0.19649	2.0713	0.8542255997362090	15330
**SEPHS1**	0.19645	2.2142	0.8543700204493380	15334
**SNX14**	0.19645	2.8998	0.8543700204493380	15333
**ZFYVE27**	0.19645	1.9308	0.8543700204493380	15335
**TLR5**	0.8036	0.19434	0.8545505714039010	15336
**EFHC2**	0.19632	1.5149	0.8548395108890720	15337
**ZSCAN16**	0.19602	4.3351	0.8559236702140230	15338
**ASCL5**	0.804	0.40499	0.8559959832715590	15339
**LYPD3**	0.19577	3.6885	0.8568279054112190	15340
**NODAL**	0.19577	1.8561	0.8568279054112190	15345
**RPL10L**	0.19577	3.1529	0.8568279054112190	15341
**SPATA31C1**	0.19577	3.0006	0.8568279054112190	15343
**VRK1**	0.19577	3.1035	0.8568279054112190	15342
**ZYX**	0.19577	2.9768	0.8568279054112190	15344
**LARGE1**	0.80435	0.04079	0.8572621872313480	15346
**CASC1**	0.19538	1.3766	0.8582399129982630	15347
**CBX2**	0.19511	2.2159	0.8592184598860190	15353
**DEF8**	0.19511	1.4399	0.8592184598860190	15356
**EGFL8**	0.19511	0.43414	0.8592184598860190	15358
**IP6K3**	0.19511	1.4715	0.8592184598860190	15355
**LEXM**	0.19511	3.0024	0.8592184598860190	15349
**OR2T3**	0.19511	1.3216	0.8592184598860190	15357
**OR5A1**	0.19511	2.8052	0.8592184598860190	15350
**PRR15**	0.19511	1.7026	0.8592184598860190	15354
**SLC17A3**	0.19511	2.6567	0.8592184598860190	15352
**TDRP**	0.19511	3.7126	0.8592184598860190	15348

**ZNF284**	0.19511	2.7036	0.8592184598860190	15351
**FADS6**	0.19504	0.69082	0.859472291549715	15359
**PTMA**	0.19498	3.2263	0.8596899056301700	15360
**USP11**	0.19496	0.55631	0.8597624527043840	15361
**TRMT12**	0.19489	2.8773	0.8600164031068770	15362
**SPAG16**	0.19482	3.1798	0.8602704089845790	15363
**RNF2**	0.19435	3.298	0.8619773167767510	15364
**PLXND1**	0.80568	0.14257	0.8620863535993890	15365
**UBE2N**	0.19432	0.12291	0.8620863535993890	15366
**ACAT1**	0.19427	1.2586	0.8622681044172050	15376
**BOLL**	0.19427	3.3058	0.8622681044172050	15371
**CD58**	0.19427	3.4962	0.8622681044172050	15368
**EPS8**	0.19427	2.7128	0.8622681044172050	15374
**HARS**	0.19427	2.8018	0.8622681044172050	15373
**RNF7**	0.19427	2.0311	0.8622681044172050	15375
**RPTOR**	0.19427	3.3375	0.8622681044172050	15370
**SLC35B3**	0.19427	3.5787	0.8622681044172050	15367
**SPON2**	0.19427	3.4334	0.8622681044172050	15369
**YBX3**	0.19427	3.2889	0.8622681044172050	15372
**ADAMTS8**	0.19411	0.9997	0.8628498985475280	15379
**DSN1**	0.19411	1.6566	0.8628498985475280	15378
**VWA8**	0.19411	2.4657	0.8628498985475280	15377
**XKR5**	0.19411	0.70126	0.8628498985475280	15380
**MEDAG**	0.19372	3.0082	0.8642692469164900	15381
**GHSR**	0.1935	3.7516	0.865070673560113	15382
**ADGRE1**	0.19344	0.25996	0.865289340905793	15388
**C2CD5**	0.19344	1.9099	0.865289340905793	15387
**CAPNS1**	0.19344	2.1501	0.865289340905793	15386
**COBL**	0.19344	2.72	0.865289340905793	15384
**HSP90B1**	0.19344	2.9066	0.865289340905793	15383
**TSSC1**	0.19344	2.6839	0.865289340905793	15385
**UBB**	0.19332	2.3879	0.8657267997691270	15389

**AGBL3**	0.19286	2.0389	0.8674052626151100	15397
**ALDH6A1**	0.19286	2.8963	0.8674052626151100	15393
**CCDC91**	0.19286	2.8071	0.8674052626151100	15394
**CPNE7**	0.19286	2.9495	0.8674052626151100	15392
**CRTAM**	0.19286	2.1044	0.8674052626151100	15396
**FCN2**	0.19286	1.7428	0.8674052626151100	15399
**FSIP2**	0.19286	2.6135	0.8674052626151100	15395
**MROH8**	0.19286	3.1155	0.8674052626151100	15390
**SCAF1**	0.19286	1.9575	0.8674052626151100	15398
**USP27X**	0.19286	3.0212	0.8674052626151100	15391
**GYG1**	0.19275	1.6873	0.8678069962958580	15400
**EPOR**	0.19265	1.2818	0.8681723303064960	15406
**FBXO48**	0.19265	2.1012	0.8681723303064960	15403
**GPM6A**	0.19265	1.395	0.8681723303064960	15404
**PAN2**	0.19265	2.8469	0.8681723303064960	15401
**PON3**	0.19265	1.3106	0.8681723303064960	15405
**RBBP8NL**	0.19265	2.5167	0.8681723303064960	15402
**ACLY**	0.19258	1.1994	0.8684281330735160	15407
**RAB11FIP1**	0.80744	0.45044	0.8685012300162120	15408
**GINS3**	0.19246	2.3277	0.8688667843617950	15409
**NAT16**	0.19238	0.25547	0.8691593114556300	15410
**FAM83C**	0.19237	3.8471	0.8691958825719410	15411
**SF3B1**	0.19237	2.594	0.8691958825719410	15412
**HNRNPA1L2**	0.19231	2.9728	0.8694153336874230	15413
**CCSER1**	0.19228	3.0452	0.8695250749477300	15414
**LARP6**	0.19213	3.0781	0.8700739384077630	15415
**ADA2**	0.19172	2.3173	0.8715755044114120	15420
**HBP1**	0.19172	1.9559	0.8715755044114120	15422
**OR13C3**	0.19172	2.4737	0.8715755044114120	15419
**RAET1L**	0.19172	2.9906	0.8715755044114120	15416
**RGS12**	0.19172	1.3871	0.8715755044114120	15423
**SMPD3**	0.19172	2.5133	0.8715755044114120	15418

**TCP1**	0.19172	2.5228	0.8715755044114120	15417
**UTP14A**	0.19172	2.2996	0.8715755044114120	15421
**AGL**	0.19167	1.538	0.8717587566603080	15427
**CDRT1**	0.19167	3.4195	0.8717587566603080	15424
**HRH4**	0.19167	2.2827	0.8717587566603080	15426
**OR5W2**	0.19167	3.3694	0.8717587566603080	15425
**TYRP1**	0.19126	1.821	0.8732625309307390	15428
**PPP2CB**	0.19108	2.2464	0.8739233486121650	15429
**TMEM263**	0.19089	3.1714	0.874621292656344	15430
**USP44**	0.19081	3.1126	0.8749152913219900	15431
**RPP40**	0.19054	0.014673	0.875908095652047	15432
**DAXX**	0.19052	0.075648	0.8759816710573350	15437
**FAM71D**	0.19052	0.85211	0.8759816710573350	15436
**HLA-DMA**	0.19052	2.1862	0.8759816710573350	15434
**RGS1**	0.19052	1.7226	0.8759816710573350	15435
**USP17L4**	0.19052	2.4403	0.8759816710573350	15433
**SLC25A3**	0.80973	0.49948	0.8769017640807430	15438
**ANKLE2**	0.19016	3.1973	0.8773068402632120	15443
**CARMIL1**	0.19016	1.251	0.8773068402632120	15447
**ELN**	0.19016	3.6047	0.8773068402632120	15439
**FGF17**	0.19016	1.3364	0.8773068402632120	15445
**KIF26A**	0.19016	3.2589	0.8773068402632120	15442
**NDUFV2**	0.19016	3.5339	0.8773068402632120	15440
**OR7A10**	0.19016	1.125	0.8773068402632120	15448
**SRSF11**	0.19016	1.2708	0.8773068402632120	15446
**TCERG1**	0.19016	3.317	0.8773068402632120	15441
**UBQLN2**	0.19016	1.475	0.8773068402632120	15444
**SLC7A1**	0.19014	4.4973	0.8773805059449130	15449
**HOXA5**	0.81008	0.031228	0.8781911429277540	15450
**EDF1**	0.18971	3.7467	0.8789654714758720	15451
**FGF5**	0.18965	3.2256	0.8791868050165310	15453
**IL15RA**	0.18965	3.2583	0.8791868050165310	15452

**TREM1**	0.18965	2.6196	0.8791868050165310	15454
**C10orf76**	0.1895	2.7854	0.8797403273966480	15455
**ANGEL2**	0.18948	0.6307	0.8798141507464460	15459
**ARPC1A**	0.18948	3.2145	0.8798141507464460	15456
**CEP85**	0.18948	2.5182	0.8798141507464460	15458
**DDHD2**	0.18948	2.747	0.8798141507464460	15457
**SERPINB4**	0.18946	2.8045	0.8798879788914370	15460
**RPL37**	0.18941	2.9924	0.8800725702396170	15461
**MGRN1**	0.18933	1.3462	0.8803679787875690	15462
**ATP6V1G1**	0.81115	0.16377	0.8821420461528890	15463
**CCDC146**	0.18879	3.1899	0.8823639998001690	15468
**CMTM5**	0.18879	3.2787	0.8823639998001690	15467
**NDUFB6**	0.18879	1.2512	0.8823639998001690	15470
**PACSIN1**	0.18879	3.3906	0.8823639998001690	15465
**RPF1**	0.18879	3.808	0.8823639998001690	15464
**SUGP2**	0.18879	3.3727	0.8823639998001690	15466
**TCL1A**	0.18879	2.9208	0.8823639998001690	15469
**ADCY10**	0.18853	2.0035	0.8833263016956310	15475
**GTPBP2**	0.18853	2.4214	0.8833263016956310	15472
**HERPUD1**	0.18853	2.1954	0.8833263016956310	15473
**LYZL6**	0.18853	2.1828	0.8833263016956310	15474
**PLCL1**	0.18853	0.81557	0.8833263016956310	15479
**RHBDD3**	0.18853	0.11325	0.8833263016956310	15481
**SIPA1L2**	0.18853	1.7785	0.8833263016956310	15477
**SLC25A41**	0.18853	2.6239	0.8833263016956310	15471
**TSC22D3**	0.18853	1.5831	0.8833263016956310	15478
**WDR11**	0.18853	0.16039	0.8833263016956310	15480
**ZNF619**	0.18853	1.9949	0.8833263016956310	15476
**DGAT2**	0.18838	3.389	0.8838818481207510	15482
**KAT2A**	0.18838	0.25702	0.8838818481207510	15483
**DUSP4**	0.18835	1.881	0.8839929901430370	15484
**TAC3**	0.18816	3.1748	0.8846971433641220	15485

**LINGO4**	0.18812	3.4184	0.8848454420480800	15486
**PIGP**	0.18806	2.7829	0.885067926568807	15487
**HOXD11**	0.18782	1.1983	0.8859583031234560	15488
**BRSK1**	0.8124	0.417	0.8867750990791240	15489
**GINS1**	0.18756	2.2611	0.886923671001735	15490
**SNTG2**	0.18754	1.7218	0.8869979643050770	15492
**VWA3B**	0.18754	2.2021	0.8869979643050770	15491
**ZBED2**	0.18754	0.27189	0.8869979643050770	15493
**C16orf72**	0.18742	3.5194	0.8874438269801280	15495
**CANX**	0.18742	2.6943	0.8874438269801280	15497
**GSTO2**	0.18742	0.96216	0.8874438269801280	15498
**TP53INP2**	0.18742	3.6509	0.8874438269801280	15494
**ZDHHC1**	0.18742	2.7953	0.8874438269801280	15496
**CYP27C1**	0.18719	2.6287	0.8882988906102020	15499
**PSD2**	0.18719	2.339	0.8882988906102020	15500
**ZNF112**	0.18686	1.1243	0.8895268567219670	15501
**CALML3**	0.18643	2.0772	0.8911289492962630	15505
**CELA3B**	0.18643	1.4091	0.8911289492962630	15506
**LCTL**	0.18643	2.6771	0.8911289492962630	15504
**SERPINB3**	0.18643	2.9165	0.8911289492962630	15502
**SLC26A6**	0.18643	2.6872	0.8911289492962630	15503
**ALOX5**	0.18629	2.8717	0.8916510545932570	15511
**CFAP43**	0.18629	3.4505	0.8916510545932570	15507
**PIGW**	0.18629	2.8775	0.8916510545932570	15510
**SFMBT1**	0.18629	3.3577	0.8916510545932570	15508
**ST3GAL5**	0.18629	3.0031	0.8916510545932570	15509
**PPTC7**	0.18593	0.062108	0.8929947285639890	15512
**INTS3**	0.18583	3.3444	0.8933682575351020	15513
**CCDC92**	0.18582	2.8879	0.8934056172878010	15514
**SLC12A2**	0.81424	0.26678	0.8936298019959900	15515
**OR4K5**	0.81437	0.31564	0.8941156896758260	15516
**TTLL8**	0.18559	0.44361	0.8942652360494680	15517

**UHRF1BP1**	0.18548	4.6151	0.8946765917389930	15518
**CYB561D2**	0.18515	2.6947	0.8959115682145320	15521
**FBRSL1**	0.18515	2.9547	0.8959115682145320	15519
**FHIT**	0.18515	2.7331	0.8959115682145320	15520
**MAMDC4**	0.18515	1.8482	0.8959115682145320	15522
**RRP1B**	0.18515	1.67	0.8959115682145320	15523
**SRM**	0.18515	0.10695	0.8959115682145320	15525
**VWC2**	0.18515	1.0532	0.8959115682145320	15524
**OR51E1**	0.18502	1.1061	0.8963984494093530	15528
**PAAF1**	0.18502	3.1754	0.8963984494093530	15527
**ZBTB3**	0.18502	3.4416	0.8963984494093530	15526
**GBP7**	0.18469	1.2607	0.8976353340440010	15529
**HCFC2**	0.18467	3.0914	0.8977103408848960	15530
**IAH1**	0.18467	2.4572	0.8977103408848960	15531
**SLC22A1**	0.18435	0.33684	0.8989111380087520	15532
**CCDC103**	0.18432	4.6431	0.8990237791937140	15533
**CFAP44**	0.18427	2.611	0.8992115398549150	15534
**UBAC2**	0.18426	1.7553	0.8992490957913420	15535
**RAB9A**	0.18425	2.9909	0.8992866529961540	15536
**AVPR1B**	0.18392	2.4226	0.9005267532279320	15537
**CTNNB1**	0.18392	1.1847	0.9005267532279320	15541
**HSD17B7**	0.18392	2.3274	0.9005267532279320	15538
**KRTAP5-11**	0.18392	1.6788	0.9005267532279320	15540
**LOC1019288**	0.18392	0.23927	0.9005267532279320	15543
**NOL6**	0.18392	0.93313	0.9005267532279320	15542
**TECTB**	0.18392	2.1429	0.9005267532279320	15539
**ATP8B3**	0.18378	2.557	0.901053274920464	15550
**EIF3H**	0.18378	2.2812	0.901053274920464	15551
**ERGIC2**	0.18378	1.1964	0.901053274920464	15552
**GPR135**	0.18378	3.4804	0.901053274920464	15546
**HRASLS2**	0.18378	3.3387	0.901053274920464	15549
**LSP1**	0.18378	3.4542	0.901053274920464	15548

**NETO2**	0.18378	3.5134	0.901053274920464	15545
**ZGRF1**	0.18378	3.8395	0.901053274920464	15544
**ZNF688**	0.18378	3.4562	0.901053274920464	15547
**ZMYND15**	0.18362	3.9594	0.9016553200357340	15553
**PTPN23**	0.18341	1.871	0.9024460005725140	15554
**CLEC18A**	0.18313	2.406	0.9035011197637460	15559
**CRYBG3**	0.18313	3.2215	0.9035011197637460	15557
**FOXK2**	0.18313	3.4536	0.9035011197637460	15556
**PAPD4**	0.18313	2.7278	0.9035011197637460	15558
**XPC**	0.18313	3.7222	0.9035011197637460	15555
**HDAC3**	0.18299	1.525	0.9040290567177420	15564
**IMMP1L**	0.18299	2.6253	0.9040290567177420	15561
**JUP**	0.18299	1.8862	0.9040290567177420	15563
**SDHAF2**	0.18299	3.0092	0.9040290567177420	15560
**SLC30A5**	0.18299	2.3123	0.9040290567177420	15562
**PTEN**	0.18297	3.8882	0.9041044968522160	15565
**GLRA4**	0.18244	3.7138	0.9061055389265610	15567
**LZTS1**	0.18244	3.5119	0.9061055389265610	15569
**MEX3B**	0.18244	3.107	0.9061055389265610	15571
**NPHP1**	0.18244	2.839	0.9061055389265610	15573
**PDCD2**	0.18244	1.0255	0.9061055389265610	15575
**PLEKHH3**	0.18244	3.02	0.9061055389265610	15572
**R3HDM2**	0.18244	3.9242	0.9061055389265610	15566
**RAB33A**	0.18244	1.0184	0.9061055389265610	15576
**SMARCA2**	0.18244	3.2033	0.9061055389265610	15570
**STT3A**	0.18244	3.5307	0.9061055389265610	15568
**VDAC3**	0.18244	2.6578	0.9061055389265610	15574
**IL17D**	0.18219	0.55736	0.9070506870294480	15577
**ADGRG7**	0.18217	2.9066	0.9071263338818350	15578
**C17orf78**	0.18207	2.556	0.9075046460366530	15584
**FBXW8**	0.18207	2.7137	0.9075046460366530	15582
**HACD3**	0.18207	2.0111	0.9075046460366530	15586

**MR1**	0.18207	2.2378	0.9075046460366530	15585
**NXF2B**	0.18207	3.0157	0.9075046460366530	15580
**SPTLC1**	0.18207	2.5594	0.9075046460366530	15583
**TRIML2**	0.18207	3.1057	0.9075046460366530	15579
**ZNF44**	0.18207	2.8302	0.9075046460366530	15581
**NXNL2**	0.18163	2.8688	0.909170764979743	15587
**NUP85**	0.18148	2.845	0.90973933718423	15588
**TNFAIP8L2**	0.1814	3.5109	0.9100426959766420	15589
**TMEM45A**	0.18135	3.1712	0.9102323377572090	15590
**CREB3L3**	0.18116	3.6826	0.9109532752776030	15591
**ACTR8**	0.18115	1.417	0.9109912324729800	15594
**IGSF23**	0.18115	2.4559	0.9109912324729800	15592
**NDUFB5**	0.18115	2.0913	0.9109912324729800	15593
**ABCG2**	0.18099	3.3966	0.9115987262052110	15597
**ALG13**	0.18099	3.3191	0.9115987262052110	15600
**BCO1**	0.18099	1.0923	0.9115987262052110	15606
**BRMS1**	0.18099	3.3229	0.9115987262052110	15599
**DNTTIP2**	0.18099	2.9834	0.9115987262052110	15602
**GDAP2**	0.18099	2.6157	0.9115987262052110	15604
**PTK2B**	0.18099	3.1031	0.9115987262052110	15601
**SYNM**	0.18099	2.7314	0.9115987262052110	15603
**TMEM8C**	0.18099	3.3409	0.9115987262052110	15598
**TUSC3**	0.18099	3.409	0.9115987262052110	15596
**ZNF773**	0.18099	1.8674	0.9115987262052110	15605
**ZXDC**	0.18099	3.7102	0.9115987262052110	15595
**DMPK**	0.18093	0.4882	0.9118266231050460	15607
**ANGPTL6**	0.18042	0.55232	0.9137656623978270	15613
**ARSA**	0.18042	2.4738	0.9137656623978270	15610
**SERPINE3**	0.18042	2.1247	0.9137656623978270	15611
**TBC1D3F**	0.18042	3.1674	0.9137656623978270	15608
**ZNF300**	0.18042	2.8464	0.9137656623978270	15609
**ZNF652**	0.18042	1.0014	0.9137656623978270	15612

**TMEM126B**	0.18034	2.5645	0.9140701372103680	15614
**DSTN**	0.18029	3.0419	0.9142604770067580	15615
**SHISA4**	0.17991	3.387	0.9157081434315990	15616
**SPRY3**	0.82016	0.23425	0.9159750281803390	15617
**ABI3BP**	0.17966	2.5237	0.916661603027261	15618
**ACOX2**	0.17965	3.3757	0.9166997587438750	15619
**SS18**	0.17965	3.019	0.9166997587438750	15620
**CRB2**	0.17964	0.67563	0.9167379157951200	15624
**METTL23**	0.17964	1.5679	0.9167379157951200	15622
**SCD**	0.17964	3.3108	0.9167379157951200	15621
**SMTNL1**	0.17964	0.043275	0.9167379157951200	15625
**TATDN2**	0.17964	1.2684	0.9167379157951200	15623
**AFAP1L2**	0.17961	1.6185	0.9168523949581240	15629
**BICD2**	0.17961	1.6067	0.9168523949581240	15630
**CETP**	0.17961	3.4233	0.9168523949581240	15628
**ENPP5**	0.17961	3.4729	0.9168523949581240	15627
**PPCDC**	0.17961	1.5273	0.9168523949581240	15631
**SYDE2**	0.17961	3.8623	0.9168523949581240	15626
**WASHC3**	0.1796	2.772	0.9168905573493790	15632
**CRTC2**	0.17953	2.919	0.9171577314870160	15633
**C12orf76**	0.17943	4.7499	0.9175395224348030	15634
**NMD3**	0.17943	4.7499	0.9175395224348030	15635
**ARVCF**	0.17928	2.4416	0.9181124597617640	15636
**ZNF672**	0.1789	3.3651	0.919565251685984	15637
**DHRS2**	0.17887	2.9735	0.9196800284480200	15638
**TMIGD3**	0.1788	0.55398	0.9199478880209180	15639
**BTK**	0.17868	1.8103	0.9204072294894220	15644
**EPN3**	0.17868	1.3181	0.9204072294894220	15645
**GTF2F2**	0.17868	2.0523	0.9204072294894220	15643
**KIF3C**	0.17868	1.2545	0.9204072294894220	15646
**LCA5**	0.17868	0.78565	0.9204072294894220	15647
**RNF39**	0.17868	2.4973	0.9204072294894220	15641

**SPOP**	0.17868	2.1606	0.9204072294894220	15642
**TMEM181**	0.17868	2.5975	0.9204072294894220	15640
**CAPN2**	0.17867	0.61921	0.920445516711238	15648
**INF2**	0.1786	1.6351	0.920713565053866	15649
**MTPN**	0.82147	0.14194	0.920981679565929	15650
**DPP9**	0.17819	0.57842	0.9222848929707170	15655
**GYS2**	0.17819	1.645	0.9222848929707170	15654
**HNF1A**	0.17819	3.7582	0.9222848929707170	15651
**LSR**	0.17819	2.2527	0.9222848929707170	15653
**SEPHS2**	0.17819	3.141	0.9222848929707170	15652
**WDFY1**	0.17819	0.54857	0.9222848929707170	15656
**CA14**	0.17805	2.5724	0.9228219657400150	15659
**DAZ3**	0.17805	2.8709	0.9228219657400150	15658
**SNAPC5**	0.17805	3.125	0.9228219657400150	15657
**STMN1**	0.17801	1.2878	0.9229754639997150	15660
**UCP1**	0.17795	3.2905	0.9232057521734580	15661
**ACSBG2**	0.17779	1.1481	0.9238200934706880	15662
**ADAMTSL1**	0.1777	2.3554	0.924165813718317	15663
**ANKS4B**	0.1777	2.0686	0.924165813718317	15665
**MED29**	0.1777	2.2939	0.924165813718317	15664
**LSM 6.00**	0.82231	0.25035	0.9242042338977190	15666
**YARS**	0.17733	3.2708	0.9255882701578240	15667
**ANKRD49**	0.17716	0.45926	0.9262424594999990	15668
**UQCRC2**	0.17698	3.5196	0.9269355629061660	15669
**C1GALT1C1**	0.17688	2.7359	0.9273208128299260	15670
**FREM1**	0.17688	0.37225	0.9273208128299260	15674
**RBM5**	0.17688	2.4223	0.9273208128299260	15671
**REN**	0.17688	1.3483	0.9273208128299260	15672
**SIRT5**	0.17688	0.58413	0.9273208128299260	15673
**EPHA4**	0.17687	1.3925	0.9273593453921150	15679
**KNOP1**	0.17687	3.5561	0.9273593453921150	15677
**PRPF40B**	0.17687	3.4598	0.9273593453921150	15678

**TOB1**	0.17687	1.1804	0.9273593453921150	15680
**USP17L22**	0.17687	3.6125	0.9273593453921150	15676
**ZNF202**	0.17687	3.619	0.9273593453921150	15675
**LCE3D**	0.17641	0.3233	0.9291333342658830	15681
**LOC1019297**	0.17621	0.77402	0.9299055464120540	15682
**SYT11**	0.17617	0.13326	0.9300600553903750	15683
**ABHD6**	0.17605	0.70255	0.9305237156046940	15689
**BBS9**	0.17605	1.2265	0.9305237156046940	15688
**EBLN2**	0.17605	2.479	0.9305237156046940	15685
**SEC24D**	0.17605	2.9883	0.9305237156046940	15684
**SELENOW**	0.17605	1.4743	0.9305237156046940	15687
**SLC22A12**	0.17605	2.2199	0.9305237156046940	15686
**HACD4**	0.17568	3.2658	0.9319545956430530	15690
**GLI3**	0.17566	1.3135	0.9320319948682210	15694
**NIPAL3**	0.17566	2.6528	0.9320319948682210	15693
**PDLIM1**	0.17566	3.4232	0.9320319948682210	15692
**RNF224**	0.17566	3.7517	0.9320319948682210	15691
**TEPSIN**	0.17563	3.2608	0.9321481041761150	15695
**KPNA7**	0.17556	1.2666	0.9324190747774130	15696
**TRIAP1**	0.17548	3.719	0.9327288393210290	15697
**DEFB112**	0.17529	0.58282	0.9334648890608250	15698
**PCBP3**	0.17529	0.16033	0.9334648890608250	15699
**THRSP**	0.17514	2.9343	0.9340463383793880	15700
**PYGL**	0.17512	2.7368	0.9341238888156910	15701
**AGXT**	0.17496	3.2504	0.9347444946720430	15702
**RAP1GAP2**	0.17479	1.4666	0.9354042831111650	15703
**KRT3**	0.17477	3.3272	0.9354819320526800	15704
**KCNA1**	0.17474	1.3517	0.935598416041777	15706
**WDR72**	0.17474	4.8494	0.935598416041777	15705
**KRBOX4**	0.17465	1.665	0.9359479442027790	15709
**LRCH2**	0.17465	2.1218	0.9359479442027790	15708
**SNRPA1**	0.17465	2.1758	0.9359479442027790	15707

**SPTBN5**	0.17465	0.52662	0.9359479442027790	15710
**MYBL1**	0.17463	3.4474	0.9360256326566600	15711
**APLNR**	0.17448	3.2488	0.9366084762367610	15713
**FKBP15**	0.17448	2.4046	0.9366084762367610	15716
**NID2**	0.17448	2.7976	0.9366084762367610	15715
**OGFRL1**	0.17448	3.6496	0.9366084762367610	15712
**SLC4A11**	0.17448	1.586	0.9366084762367610	15717
**SPRY4**	0.17448	3.2336	0.9366084762367610	15714
**CFAP57**	0.17437	1.8188	0.9370360971575740	15718
**CTSF**	0.17391	3.7756	0.9388261898089340	15719
**DNASE1L2**	0.17391	1.9093	0.9388261898089340	15721
**PCSK4**	0.17391	2.2559	0.9388261898089340	15720
**C2CD3**	0.17354	4.8733	0.940268232946484	15722
**LCN2**	0.17352	2.0525	0.9403462369268820	15723
**EEF1D**	0.17339	0.089409	0.9408534023334980	15724
**LEAP2**	0.17325	3.292	0.9413998512456300	15725
**OVCH1**	0.82675	0.19897	0.9413998512456300	15726
**DIO1**	0.17319	1.4576	0.9416341297078000	15729
**LRTM2**	0.17319	0.39055	0.9416341297078000	15731
**MUTYH**	0.17319	1.2066	0.9416341297078000	15730
**PSMB2**	0.17319	1.7201	0.9416341297078000	15728
**ZCCHC8**	0.17319	2.1681	0.9416341297078000	15727
**GABRG2**	0.17317	3.8592	0.9417122340143710	15732
**IL18**	0.17317	2.58	0.9417122340143710	15736
**LENEP**	0.17317	2.8064	0.9417122340143710	15734
**OR4C15**	0.17317	3.6392	0.9417122340143710	15733
**SETDB2**	0.17317	2.676	0.9417122340143710	15735
**TG**	0.17317	1.1776	0.9417122340143710	15737
**IFI27L2**	0.17299	1.1422	0.9424154314628770	15738
**UTP3**	0.17261	3.7715	0.9439014919598120	15739
**BRK1**	0.17254	2.9808	0.94417546735333	15740
**SMIM13**	0.17248	3.0887	0.9444103598257420	15741

**DNAJB12**	0.17247	2.9633	0.9444495136372780	15742
**DMTN**	0.17227	2.3629	0.9452328941520700	15744
**FTMT**	0.17227	1.5964	0.9452328941520700	15746
**KCNA2**	0.17227	2.5583	0.9452328941520700	15743
**SLC9A2**	0.17227	1.4645	0.9452328941520700	15747
**UTP15**	0.17227	1.7627	0.9452328941520700	15745
**CAV1**	0.17204	3.7931	0.9461344994757010	15748
**LCN8**	0.17204	2.4508	0.9461344994757010	15750
**OR10A5**	0.17204	3.1108	0.9461344994757010	15749
**IGFBPL1**	0.17164	2.6635	0.9477043433952860	15751
**MASTL**	0.17154	3.5344	0.9480971694218800	15752
**AATF**	0.17124	2.6176	0.9492765263254260	15753
**CYGB**	0.17124	1.3207	0.9492765263254260	15757
**OR8B12**	0.17124	1.7993	0.9492765263254260	15755
**SLC30A6**	0.17124	1.5858	0.9492765263254260	15756
**ZNF71**	0.17124	2.256	0.9492765263254260	15754
**ADAM23**	0.1711	0.86222	0.9498273449531940	15758
**ANKRD29**	0.17098	2.7013	0.9502997046742910	15764
**CTBP2**	0.17098	3.9883	0.9502997046742910	15759
**DMRTC2**	0.17098	2.7632	0.9502997046742910	15762
**MARC1**	0.17098	3.0641	0.9502997046742910	15761
**NUDT15**	0.17098	2.2007	0.9502997046742910	15765
**RAC1**	0.17098	3.524	0.9502997046742910	15760
**ZNF365**	0.17098	2.7141	0.9502997046742910	15763
**CNN3**	0.17047	2.8236	0.9523096036413180	15766
**GJD4**	0.17036	1.3801	0.9527436158031920	15768
**LRRC31**	0.17036	0.98201	0.9527436158031920	15769
**RHCG**	0.17036	0.076497	0.9527436158031920	15770
**SPECC1L**	0.17036	3.3996	0.9527436158031920	15767
**AKAP8L**	0.17008	2.247	0.9538491847207980	15774
**APOBEC2**	0.17008	3.623	0.9538491847207980	15771
**DXO**	0.17008	0.13454	0.9538491847207980	15775

**KRTAP21-1**	0.17008	2.5464	0.9538491847207980	15773
**TP53AIP1**	0.17008	3.4804	0.9538491847207980	15772
**FLT3**	0.17005	1.6484	0.9539677076883290	15776
**ADAMTS17**	0.16979	3.0859	0.9549954686611890	15781
**CCNL1**	0.16979	3.3546	0.9549954686611890	15777
**COMMD3-B**	0.16979	3.0089	0.9549954686611890	15782
**KHDC1L**	0.16979	1.2254	0.9549954686611890	15785
**KRI1**	0.16979	3.0898	0.9549954686611890	15780
**NDFIP2**	0.16979	1.1601	0.9549954686611890	15786
**PTPN22**	0.16979	3.3188	0.9549954686611890	15779
**SEC16B**	0.16979	2.4154	0.9549954686611890	15784
**TICAM1**	0.16979	1.058	0.9549954686611890	15787
**TM9SF4**	0.16979	2.5256	0.9549954686611890	15783
**ZNF236**	0.16979	3.3517	0.9549954686611890	15778
**NRTN**	0.16972	3.1697	0.9552723459336250	15788
**FAM157B**	0.16949	1.7353	0.9561826016150550	15793
**HAL**	0.16949	3.0387	0.9561826016150550	15789
**HECA**	0.16949	1.9446	0.9561826016150550	15792
**HFE2**	0.16949	2.8807	0.9561826016150550	15790
**PTBP1**	0.16949	2.5554	0.9561826016150550	15791
**SLC10A6**	0.16949	0.41265	0.9561826016150550	15794
**PHGR1**	0.1693	0.077579	0.9569351499516070	15795
**GPR27**	0.16909	1.2725	0.9577675449175900	15796
**NPBWR2**	0.16901	1.8469	0.9580848223694350	15797
**CENPC**	0.16852	2.7008	0.9600302552567720	15798
**IARS2**	0.16852	1.6015	0.9600302552567720	15801
**SMCHD1**	0.16852	2.6803	0.9600302552567720	15799
**TCEA1**	0.16852	1.8015	0.9600302552567720	15800
**C12orf49**	0.16847	3.179	0.9602289731411680	15808
**CMTR1**	0.16847	3.6635	0.9602289731411680	15803
**FBXO46**	0.16847	2.3044	0.9602289731411680	15810
**INA**	0.16847	1.6268	0.9602289731411680	15811

**LHX9**	0.16847	0.80592	0.9602289731411680	15812
**LMOD3**	0.16847	3.2837	0.9602289731411680	15806
**SAXO2**	0.16847	2.8681	0.9602289731411680	15809
**UTP23**	0.16847	3.6438	0.9602289731411680	15804
**ZNF225**	0.16847	3.3536	0.9602289731411680	15805
**ZNF24**	0.16847	3.6637	0.9602289731411680	15802
**ZNF577**	0.16847	3.2414	0.9602289731411680	15807
**MCM3AP**	0.16798	4.9869	0.9621784188968760	15813
**METTL8**	0.16798	4.9869	0.9621784188968760	15814
**OVCA2**	0.16766	2.9983	0.9634535024575650	15815
**ATG2B**	0.16736	1.7346	0.964650317558958	15819
**CCDC105**	0.16736	2.6762	0.964650317558958	15816
**KCTD18**	0.16736	0.81708	0.964650317558958	15822
**KSR2**	0.16736	0.9518	0.964650317558958	15821
**NT5C1A**	0.16736	1.6998	0.964650317558958	15820
**RPUSD3**	0.16736	2.1241	0.964650317558958	15817
**TINAG**	0.16736	1.7729	0.964650317558958	15818
**AK8**	0.16711	2.2011	0.965648719977347	15825
**CLDN20**	0.16711	3.2961	0.965648719977347	15823
**VCPIP1**	0.16711	3.2545	0.965648719977347	15824
**AKR1B1**	0.16702	3.0966	0.9660083805620480	15827
**ETNK1**	0.16702	1.27	0.9660083805620480	15829
**GALNT5**	0.16702	3.3932	0.9660083805620480	15826
**KEAP1**	0.16702	1.5169	0.9660083805620480	15828
**AKAP1**	0.16678	3.1397	0.9669680869381840	15830
**PCDHGA12**	0.16677	5.0086	0.9670080940317100	15831
**ZNF84**	0.16677	5.0086	0.9670080940317100	15832
**GOLGA5**	0.83332	0.077967	0.9673682275473870	15833
**SUGT1**	0.83346	0.014186	0.9679286847533770	15834
**ADAM29**	0.16644	2.7208	0.96832919754624	15836
**XIRP2**	0.16644	2.9772	0.96832919754624	15835
**PLN**	0.16637	3.5419	0.9686096489474300	15837

**NPAT**	0.1661	3.5137	0.9696921044685610	15838
**AHCTF1**	0.16593	2.3135	0.9703742338227720	15839
**BACH1**	0.16593	1.7533	0.9703742338227720	15840
**QPCTL**	0.16593	0.64289	0.9703742338227720	15841
**CA12**	0.16582	1.2527	0.9708158523418160	15842
**AOC1**	0.16576	3.218	0.9710568149910780	15849
**CLCN1**	0.16576	3.5972	0.9710568149910780	15846
**COPS7B**	0.16576	3.7623	0.9710568149910780	15843
**EXT2**	0.16576	3.6494	0.9710568149910780	15845
**HIST1H2BC**	0.16576	3.6948	0.9710568149910780	15844
**HTR1B**	0.16576	1.3752	0.9710568149910780	15850
**LAD1**	0.16576	3.2696	0.9710568149910780	15848
**LAMA4**	0.16576	3.5967	0.9710568149910780	15847
**SEMA5B**	0.16576	0.82041	0.9710568149910780	15851
**ACTL9**	0.16524	0.078816	0.9731475246334360	15859
**FAM83B**	0.16524	2.6291	0.9731475246334360	15852
**HPSE2**	0.16524	1.4311	0.9731475246334360	15857
**KLRB1**	0.16524	0.23372	0.9731475246334360	15858
**OR5B2**	0.16524	2.3243	0.9731475246334360	15855
**PCSK9**	0.16524	2.3023	0.9731475246334360	15856
**SPINK5**	0.16524	2.5512	0.9731475246334360	15853
**SULF1**	0.16524	2.3308	0.9731475246334360	15854
**MIA2**	0.16466	0.72069	0.9754844996665720	15860
**ASB10**	0.16452	3.6707	0.9760493956871090	15863
**DHODH**	0.16452	3.5197	0.9760493956871090	15865
**DUSP16**	0.16452	3.3784	0.9760493956871090	15867
**FOXD1**	0.16452	1.5359	0.9760493956871090	15872
**HCN4**	0.16452	3.5374	0.9760493956871090	15864
**LOC100129(**	0.16452	3.5084	0.9760493956871090	15866
**PCSK5**	0.16452	2.8756	0.9760493956871090	15870
**RCAN2**	0.16452	3.7109	0.9760493956871090	15862
**RENBP**	0.16452	2.8372	0.9760493956871090	15871

**SLC1A2**	0.16452	3.7344	0.9760493956871090	15861
**SLC27A2**	0.16452	3.072	0.9760493956871090	15869
**ZKSCAN7**	0.16452	3.3349	0.9760493956871090	15868
**AKAP2**	0.16428	0.41766	0.9770185141868290	15878
**COX6B1**	0.16428	2.7112	0.9770185141868290	15874
**ECD**	0.16428	2.4474	0.9770185141868290	15875
**ERCC8**	0.16428	3.8854	0.9770185141868290	15873
**IRF1**	0.16428	1.6411	0.9770185141868290	15876
**SLC26A2**	0.16428	1.4702	0.9770185141868290	15877
**NFX1**	0.83618	0.026936	0.9788785608466890	15879
**TGM1**	0.16381	3.6036	0.9789190342562820	15880
**ABCA3**	0.1637	2.7684	0.9793643476436540	15884
**ADGRE5**	0.1637	2.7721	0.9793643476436540	15883
**IRX2**	0.1637	3.4833	0.9793643476436540	15881
**TRPC1**	0.1637	3.3528	0.9793643476436540	15882
**EEF1AKMT2**	0.16353	1.4779	0.980052941504328	15886
**GCNT7**	0.16353	2.4217	0.980052941504328	15885
**SERINC4**	0.16337	1.7868	0.9807014546169660	15887
**HAPLN2**	0.1633	4.1478	0.9809853088361530	15888
**CELA1**	0.16316	3.5026	0.9815532545306480	15891
**FAF1**	0.16316	1.0156	0.9815532545306480	15897
**FGF14**	0.16316	1.1098	0.9815532545306480	15896
**FGF19**	0.16316	3.6708	0.9815532545306480	15890
**KRTAP5-5**	0.16316	1.2074	0.9815532545306480	15895
**LTF**	0.16316	3.1447	0.9815532545306480	15894
**MYLK**	0.16316	3.3188	0.9815532545306480	15893
**SPAG9**	0.16316	4.2008	0.9815532545306480	15889
**STON1**	0.16316	3.4715	0.9815532545306480	15892
**C22orf15**	0.16287	1.6822	0.9827307218220650	15901
**FAM26E**	0.16287	2.2735	0.9827307218220650	15900
**PDZD4**	0.16287	2.3959	0.9827307218220650	15898
**RPS8**	0.16287	2.3517	0.9827307218220650	15899

**LRFN2**	0.83726	0.041594	0.9832589944944230	15902
**TBL1X**	0.16263	1.4475	0.9837062088660430	15903
**DACH1**	0.16251	0.54499	0.9841943035805330	15904
**DUX4**	0.1623	3.1723	0.9850490340028810	15905
**PCDHGB6**	0.1623	2.1411	0.9850490340028810	15906
**NTSR2**	0.83771	0.086443	0.9850897534023510	15907
**NAP1L5**	0.16218	3.6143	0.9855377746453310	15908
**RNASEL**	0.16218	1.1403	0.9855377746453310	15911
**SLC22A18A**	0.16218	0.95738	0.9855377746453310	15912
**SLC30A10**	0.16218	2.231	0.9855377746453310	15909
**TRAFD1**	0.16218	2.101	0.9855377746453310	15910
**SOBP**	0.16213	1.2797	0.9857414860728380	15913
**AUP1**	0.16204	5.0922	0.9861082697643850	15914
**BTBD1**	0.16198	2.0393	0.9863528659437980	15920
**CHORDC1**	0.16198	3.1574	0.9863528659437980	15918
**FMO3**	0.16198	1.1552	0.9863528659437980	15921
**HERC4**	0.16198	0.8821	0.9863528659437980	15922
**HS3ST3B1**	0.16198	3.5927	0.9863528659437980	15917
**NKAIN3**	0.16198	3.8601	0.9863528659437980	15915
**PCDHB1**	0.16198	3.1142	0.9863528659437980	15919
**TDO2**	0.16198	3.648	0.9863528659437980	15916
**IL2RB**	0.16183	0.49515	0.9869646147210210	15923
**CEACAM20**	0.16149	1.5495	0.9883526146111080	15925
**DENND2C**	0.16149	0.67631	0.9883526146111080	15927
**EBNA1BP2**	0.16149	2.5795	0.9883526146111080	15924
**TMEM69**	0.16149	0.93384	0.9883526146111080	15926
**ZNF433**	0.16146	2.6847	0.9884751766164440	15928
**NOLC1**	0.16139	1.9096	0.9887612123887090	15929
**HIST2H3D**	0.16099	3.0232	0.990397256942993	15930
**DTD1**	0.16094	2.0234	0.9906019489421420	15934
**KDM4B**	0.16094	2.6516	0.9906019489421420	15933
**PPP1R3E**	0.16094	3.4163	0.9906019489421420	15931

**TUBB2B**	0.16094	3.2154	0.9906019489421420	15932
**DUSP26**	0.16083	1.4174	0.9910524175032750	15940
**FAM47B**	0.16083	3.3689	0.9910524175032750	15937
**GBF1**	0.16083	3.249	0.9910524175032750	15938
**PEX5**	0.16083	3.6124	0.9910524175032750	15935
**PMEPA1**	0.16083	1.8083	0.9910524175032750	15939
**TAS2R31**	0.16083	3.5405	0.9910524175032750	15936
**LOC388282**	0.16059	2.6755	0.992035956737155	15943
**MRPL4**	0.16059	2.7488	0.992035956737155	15942
**PIK3C2A**	0.16059	2.5005	0.992035956737155	15944
**RFPL3**	0.16059	1.0501	0.992035956737155	15945
**TTYH1**	0.16059	3.2471	0.992035956737155	15941
**MRM1**	0.16039	1.4119	0.9928563064229350	15946
**APCS**	0.16022	3.3426	0.9935541293644720	15947
**ZBED6CL**	0.16018	1.1373	0.9937183933216410	15948
**DMXL1**	0.16017	2.8726	0.9937594635006510	15949
**LAMB4**	0.15984	5.1323	0.995115721047217	15950
**AP3M1**	0.15964	2.4466	0.9959385868973170	15958
**APOBEC1**	0.15964	1.6028	0.9959385868973170	15962
**ARSB**	0.15964	2.0296	0.9959385868973170	15960
**C15orf62**	0.15964	3.2585	0.9959385868973170	15951
**CCNO**	0.15964	1.8947	0.9959385868973170	15961
**DDB2**	0.15964	3.1738	0.9959385868973170	15952
**GALNT15**	0.15964	1.2001	0.9959385868973170	15964
**LCE1B**	0.15964	3.043	0.9959385868973170	15953
**MCM8**	0.15964	2.0508	0.9959385868973170	15959
**MRPL53**	0.15964	2.923	0.9959385868973170	15954
**NF1**	0.15964	1.4662	0.9959385868973170	15963
**OPHN1**	0.15964	2.7085	0.9959385868973170	15956
**RAB27B**	0.15964	2.5346	0.9959385868973170	15957
**SPTLC3**	0.15964	2.7381	0.9959385868973170	15955
**TNIP3**	0.15964	1.0995	0.9959385868973170	15965

**ZNF679**	0.15964	0.22733	0.9959385868973170	15966
**ZNF608**	0.15917	3.7307	0.9978749812162820	15967
**ZNF775**	0.15884	4.0355	0.9992368161079060	15968
**ALKBH1**	0.15875	2.0928	0.9996085473686820	15974
**BRAF**	0.15875	3.6056	0.9996085473686820	15971
**FRMD7**	0.15875	2.5902	0.9996085473686820	15973
**GRP**	0.15875	5.1519	0.9996085473686820	15970
**ITM2A**	0.15875	2.0874	0.9996085473686820	15975
**PPP1R37**	0.15875	1.216	0.9996085473686820	15976
**SLC5A11**	0.15875	2.7541	0.9996085473686820	15972
**UNC79**	0.15875	5.1519	0.9996085473686820	15969
**ZNF319**	0.15873	3.8384	0.9996911730754440	15977
**RRN3**	0.15871	3.52	0.9997738056076600	15978
**SPDYE4**	0.15863	3.2017	1.0001044040249100	15979
**C7orf77**	0.15853	2.8843	1.0005178058224200	15983
**COMTD1**	0.15853	3.3366	1.0005178058224200	15982
**FER1L5**	0.15853	2.7811	1.0005178058224200	15984
**OR10G3**	0.15853	3.997	1.0005178058224200	15981
**OR51B5**	0.15853	4.0076	1.0005178058224200	15980
**EVA1B**	0.15828	1.4726	1.0015520591688000	15985
**TMEM150C**	0.15815	3.5745	1.0020902945304300	15986
**GSPT1**	0.15804	2.765	1.0025459513005200	15987
**TMEM105**	0.15798	1.0043	1.002794579112070	15988
**ASB6**	0.15797	1.4803	1.0028360231075700	15993
**FSTL5**	0.15797	2.922	1.0028360231075700	15989
**FUT5**	0.15797	1.0811	1.0028360231075700	15994
**GATB**	0.15797	0.058787	1.0028360231075700	15996
**PTPRS**	0.15797	1.8766	1.0028360231075700	15991
**PZP**	0.15797	0.66247	1.0028360231075700	15995
**TLDC2**	0.15797	1.5482	1.0028360231075700	15992
**TSNAXIP1**	0.15797	2.6207	1.0028360231075700	15990
**STYX**	0.15783	0.21389	1.0034164200094200	15997

**TMEM252**	0.15765	1.3856	1.0041631416387800	15998
**RASGRP4**	0.15751	1.0329	1.004744312455290	15999
**KDSR**	0.15747	2.7896	1.004910423602200	16000
**ARPC5**	0.15741	1.9945	1.005159642326260	16009
**CCAR1**	0.15741	3.2362	1.005159642326260	16008
**CCL3L3**	0.15741	3.9476	1.005159642326260	16001
**CTNNBL1**	0.15741	3.9469	1.005159642326260	16002
**DBNL**	0.15741	3.3651	1.005159642326260	16006
**ITPRIP**	0.15741	1.39	1.005159642326260	16011
**LAMTOR1**	0.15741	3.3212	1.005159642326260	16007
**PGBD5**	0.15741	3.6004	1.005159642326260	16005
**PIGZ**	0.15741	3.7734	1.005159642326260	16004
**SHMT1**	0.15741	1.5959	1.005159642326260	16010
**SLC9A7**	0.15741	3.9161	1.005159642326260	16003
**SAMD1**	0.15736	2.6106	1.005367372296120	16012
**ST6GALNAC**	0.15731	2.3589	1.0055751456583500	16013
**DIXDC1**	0.15725	2.9826	1.0058245310091000	16014
**CHRNA5**	0.15719	2.5539	1.0060739789307700	16016
**CPSF6**	0.15719	2.57	1.0060739789307700	16015
**PPM1F**	0.15712	0.32139	1.0063650806513400	16020
**SIX6**	0.15712	3.5569	1.0063650806513400	16017
**SLC6A19**	0.15712	2.451	1.0063650806513400	16018
**TRABD**	0.15712	2.3382	1.0063650806513400	16019
**UBQLN1**	0.15712	0.28612	1.0063650806513400	16021
**PLEKHG5**	0.157	1.1007	1.0068643106771800	16022
**P2RY10**	0.15659	0.94809	1.0085719098987400	16024
**RSAD2**	0.15659	3.0717	1.0085719098987400	16023
**POU6F1**	0.15657	3.5446	1.008655282656630	16025
**VAV3**	0.15651	1.1633	1.0089054430078900	16026
**PRSS35**	0.15649	0.6412	1.0089888438233800	16027
**ATG12**	0.15618	1.5224	1.0102824550828900	16028
**C16orf58**	0.15607	4.0495	1.0107418850095900	16029

**CD163L1**	0.15605	1.7152	1.0108254406466700	16031
**GP6**	0.15605	0.43558	1.0108254406466700	16032
**ORM2**	0.15605	2.0082	1.0108254406466700	16030
**RET**	0.15602	2.04	1.0109507873360600	16033
**RIMS2**	0.15602	1.3416	1.0109507873360600	16034
**AKT3**	0.15593	0.25202	1.0113269227432600	16035
**ANKRD66**	0.15581	1.1043	1.0118286593014300	16036
**SSH2**	0.15576	3.6618	1.0120377913936300	16037
**IL18RAP**	0.15567	2.6857	1.0124143407438700	16038
**OR13A1**	0.15558	2.464	1.0127910336983100	16040
**SRPX2**	0.15558	3.0579	1.0127910336983100	16039
**ACIN1**	0.15555	3.6903	1.012916629956680	16042
**FAM177A1**	0.15555	3.756	1.012916629956680	16041
**FAM221A**	0.15555	3.437	1.012916629956680	16045
**MTFR2**	0.15555	3.4483	1.012916629956680	16044
**OR2S2**	0.15555	3.4516	1.012916629956680	16043
**SMIM15**	0.15555	3.3747	1.012916629956680	16046
**KDM5B**	0.15546	2.015	1.0132935146371000	16047
**ATXN2L**	0.15532	2.163	1.0138800659483900	16048
**AADAT**	0.15506	3.7269	1.0149703019794300	16049
**FAM71B**	0.15506	3.3219	1.0149703019794300	16052
**GSAP**	0.15506	3.469	1.0149703019794300	16051
**MGAT2**	0.15506	3.6152	1.0149703019794300	16050
**LYN**	0.15505	3.3685	1.0150122582281900	16053
**CDKN1C**	0.15499	3.4622	1.015264033252020	16054
**ERBB2**	0.15498	0.74879	1.0153060020133200	16057
**VBP1**	0.15498	3.1195	1.0153060020133200	16055
**ZSCAN25**	0.15498	2.1333	1.0153060020133200	16056
**OR1L6**	0.15474	1.8448	1.016313789328750	16059
**RELA**	0.15474	2.6977	1.016313789328750	16058
**ZNF519**	0.15463	2.0199	1.0167760369759900	16060
**TRPA1**	0.15459	3.7306	1.0169441809043400	16061

**SCNN1D**	0.15438	1.3507	1.017827408650860	16062
**PCDHA13**	0.15423	3.8011	1.018458772047290	16063
**PPIF**	0.1542	3.6451	1.018585093450410	16064
**CHST7**	0.15405	2.6232	1.019216944426060	16065
**ARHGEF7**	0.84603	0.043136	1.0195540980507200	16066
**HIST1H2AJ**	0.15396	2.8109	1.0195962504030600	16067
**CIC**	0.15392	2.2742	1.0197648779318900	16070
**PRPSAP2**	0.15392	0.50649	1.0197648779318900	16073
**SERPINB12**	0.15392	3.3011	1.0197648779318900	16068
**SLC22A24**	0.15392	1.7236	1.0197648779318900	16071
**TES**	0.15392	2.5566	1.0197648779318900	16069
**ZNF474**	0.15392	0.70189	1.0197648779318900	16072
**C17orf58**	0.15385	3.5198	1.0200600459028100	16074
**TBKBP1**	0.15385	3.4859	1.0200600459028100	16075
**FERMT3**	0.15381	4.1404	1.0202287532232900	16076
**FAM200A**	0.15374	3.6524	1.020524060927460	16077
**CALB1**	0.15358	3.5705	1.0211993843846600	16081
**CDK3**	0.15358	3.5846	1.0211993843846600	16080
**CHPF2**	0.15358	2.6596	1.0211993843846600	16082
**CRYAA**	0.15358	1.1058	1.0211993843846600	16085
**HMCN1**	0.15358	2.045	1.0211993843846600	16084
**NRSN2**	0.15358	3.8132	1.0211993843846600	16078
**OR2L2**	0.15358	2.4393	1.0211993843846600	16083
**ZNF445**	0.15358	3.7832	1.0211993843846600	16079
**OR51F1**	0.15318	2.5259	1.022889734085210	16086
**SHH**	0.15317	1.6114	1.0229320302771100	16087
**BTBD11**	0.15315	2.4818	1.0230166281513400	16088
**SMIM24**	0.15308	0.85718	1.0233127783853900	16089
**ANKRD37**	0.15293	2.0145	1.0239476884422900	16091
**CACTIN**	0.15293	3.7685	1.0239476884422900	16090
**KLHL2**	0.15293	0.18275	1.0239476884422900	16092
**REST**	0.15291	3.0284	1.024032374306120	16093

**ATP7B**	0.15289	3.386	1.0241170675146300	16094
**DHRS4L2**	0.15279	3.1864	1.0245406437931700	16095
**ANXA9**	0.15273	3.5739	1.0247948778169500	16096
**CCDC93**	0.15273	0.73804	1.0247948778169500	16100
**FAM120C**	0.15273	0.27126	1.0247948778169500	16101
**OCIAD2**	0.15273	2.2247	1.0247948778169500	16098
**SMPDL3A**	0.15273	2.261	1.0247948778169500	16097
**TTF1**	0.15273	0.87828	1.0247948778169500	16099
**FAM49A**	0.15261	2.3258	1.0253035446797000	16102
**MRE11**	0.15245	5.2644	1.0259821801207800	16104
**SEMA4G**	0.15245	5.2644	1.0259821801207800	16103
**ZNF841**	0.15236	1.3575	1.0263641202854500	16105
**COPS9**	0.15229	1.8829	1.0266612884026200	16106
**SMG9**	0.84778	0.04634	1.0269585472106600	16107
**WDR47**	0.15204	1.9464	1.0277233440121100	16108
**RNF175**	0.15202	3.7096	1.0278083585449300	16109
**SMARCA4**	0.15183	3.9254	1.0286163674033800	16110
**SLC25A24**	0.15175	3.2254	1.0289567826417400	16111
**SBF2**	0.15174	3.7413	1.0289993429308600	16112
**SPPL2C**	0.15174	3.5282	1.0289993429308600	16113
**WNT16**	0.15174	2.6187	1.0289993429308600	16114
**NLRP13**	0.15166	2.6173	1.029339892367600	16115
**CSDE1**	0.15161	3.5733	1.0295527963998300	16116
**C7orf50**	0.84858	0.054737	1.0303622576811500	16117
**CT45A10**	0.15128	0.05641	1.0309591351059200	16123
**DIAPH3**	0.15128	0.71346	1.0309591351059200	16121
**FOLH1B**	0.15128	2.2395	1.0309591351059200	16120
**KCNK12**	0.15128	3.2899	1.0309591351059200	16118
**PSMD7**	0.15128	0.28823	1.0309591351059200	16122
**ZDHHC9**	0.15128	2.299	1.0309591351059200	16119
**KLF6**	0.15125	2.1264	1.0310870852033900	16124
**TRIM6-TRIM**	0.15125	1.6354	1.0310870852033900	16125

**MS4A12**	0.15123	1.5135	1.0311723946470700	16126
**CLDN8**	0.15104	1.9762	1.031983208959170	16127
**TGIF2-C20o**	0.15093	3.7933	1.0324529380167500	16128
**MYO16**	0.84909	0.0002704	1.032538367777300	16129
**PRAG1**	0.15089	3.7627	1.0326238050742100	16130
**RPA3**	0.15076	0.44522	1.0331793313382500	16131
**BRF1**	0.15064	1.7743	1.0336924079646700	16132
**SAV1**	0.15062	2.6545	1.0337779471941200	16133
**ZEB1**	0.15059	2.746	1.0339062702228600	16134
**TMEM14B**	0.15049	1.5945	1.0343341366646700	16135
**C2orf27B**	0.15038	3.7522	1.0348050085620100	16136
**CHRNB2**	0.15021	4.0094	1.035533171302980	16137
**JAKMIP1**	0.15021	3.381	1.035533171302980	16139
**PEA15**	0.15021	2.4859	1.035533171302980	16142
**RAD21**	0.15021	1.3151	1.035533171302980	16143
**RGS10**	0.15021	3.7398	1.035533171302980	16138
**TAC1**	0.15021	3.2244	1.035533171302980	16140
**ZNF469**	0.15021	3.1939	1.035533171302980	16141
**AAAS**	0.15013	0.32748	1.0358760261778200	16146
**LIPH**	0.15013	1.0977	1.0358760261778200	16145
**MATN2**	0.15013	2.334	1.0358760261778200	16144
**MAPRE1**	0.14991	1.3207	1.0368195055473300	16148
**PALM2-AKA**	0.14991	1.576	1.0368195055473300	16147
**WNT5B**	0.14985	2.2504	1.0370769783254900	16149
**CDH16**	0.14984	2.3511	1.037119897139550	16150
**TEKT1**	0.14984	2.198	1.037119897139550	16151
**MGMT**	0.14979	2.998	1.0373345198720700	16152
**CPNE8**	0.14976	3.6773	1.0374633164503400	16153
**TXNDC8**	0.14949	3.0377	1.0386232610177900	16154
**OR2D3**	0.14946	1.1385	1.0387522300227800	16155
**CHGB**	0.14943	1.4199	1.038881216307640	16156
**S100A13**	0.14935	1.7061	1.0392252642648100	16157

**CYP11A1**	0.14928	2.8613	1.0395264071672400	16158
**MROH9**	0.14918	0.1759	1.0399567749183200	16159
**SHBG**	0.14911	1.5183	1.040258146987390	16160
**TF**	0.14909	2.6802	1.0403442706482200	16161
**DDX39A**	0.14902	3.1014	1.040645764247480	16162
**CREBZF**	0.14896	2.8504	1.0409042626411200	16163
**FAM133B**	0.14896	1.9209	1.0409042626411200	16166
**NDN**	0.14896	2.4645	1.0409042626411200	16165
**SDR39U1**	0.14896	0.83567	1.0409042626411200	16167
**SLC13A2**	0.14896	0.1394	1.0409042626411200	16168
**TMPRSS4**	0.14896	2.4778	1.0409042626411200	16164
**UNC45A**	0.14885	2.6393	1.0413783570997300	16169
**ST7L**	0.14882	2.7204	1.0415076962172600	16170
**OMD**	0.14864	3.5969	1.042284097094020	16171
**DIS3L**	0.14859	2.4259	1.0424998755324600	16175
**LCP1**	0.14859	3.8442	1.0424998755324600	16172
**POU5F1**	0.14859	2.1786	1.0424998755324600	16176
**PSMB10**	0.14859	3.2382	1.0424998755324600	16174
**SLC34A3**	0.14859	1.5125	1.0424998755324600	16177
**STAM2**	0.14859	3.2522	1.0424998755324600	16173
**TFAP2A**	0.1485	3.3033	1.0428883990891100	16178
**GLIPR1L1**	0.14844	3.799	1.043147502275490	16179
**RNF13**	0.14831	0.98396	1.0437091328968300	16180
**HSD11B1L**	0.14828	2.1289	1.0438387867227100	16181
**ZNF22**	0.14822	1.6955	1.0440981470296300	16182
**TMEM190**	0.14815	3.8106	1.0444008228503600	16183
**TAF5L**	0.1481	5.3347	1.0446170784582800	16184
**TPM4**	0.14806	3	1.0447901181251800	16185
**IVL**	0.14787	0.75351	1.045612484121850	16186
**DGKH**	0.1476	1.653	1.0467823273855400	16193
**MPP5**	0.1476	1.8875	1.0467823273855400	16192
**NOL10**	0.1476	2.2997	1.0467823273855400	16191

**PKLR**	0.1476	3.7822	1.0467823273855400	16190
**PTGFRN**	0.1476	1.4587	1.0467823273855400	16194
**RBFOX1**	0.1476	1.0631	1.0467823273855400	16195
**SIPA1**	0.1476	4.1631	1.0467823273855400	16187
**SLC35G1**	0.1476	3.9721	1.0467823273855400	16188
**TCF3**	0.1476	3.9071	1.0467823273855400	16189
**OR10G9**	0.14756	2.0777	1.0469557593604000	16196
**CHRAC1**	0.14749	4.0926	1.0472593411153600	16197
**FAM175A**	0.14706	4.0356	1.0491263219805600	16198
**IKBKE**	0.14706	3.4558	1.0491263219805600	16199
**MT1H**	0.14706	3.1678	1.0491263219805600	16200
**ZFAND6**	0.14703	3.4387	1.049256712950040	16201
**MCMBP**	0.14701	1.9156	1.0493436501746300	16202
**HSPB1**	0.14664	0.033711	1.0509534217350900	16204
**ZNF12**	0.14664	1.4707	1.0509534217350900	16203
**UTRN**	0.14649	2.3088	1.0516068082251400	16205
**NDST1**	0.1463	2.8084	1.0524350761558500	16206
**FAR1**	0.14619	1.363	1.0529149298647100	16207
**MC1R**	0.14609	3.3163	1.0533513709909700	16208
**ATP11AUN**	0.14583	3.8075	1.0544870580298600	16209
**CCBE1**	0.14583	3.763	1.0544870580298600	16210
**DYNC1I1**	0.14583	3.5318	1.0544870580298600	16213
**JMJD4**	0.14583	3.2811	1.0544870580298600	16214
**KRT33B**	0.14583	1.6377	1.0544870580298600	16216
**NELFCD**	0.14583	3.685	1.0544870580298600	16211
**TMEM38A**	0.14583	3.5585	1.0544870580298600	16212
**TWF1**	0.14583	3.0845	1.0544870580298600	16215
**FAM60A**	0.14577	2.463	1.0547493328581900	16217
**PHGDH**	0.14575	3.5534	1.05483677392569	16218
**TEX264**	0.14565	3.1066	1.0552741002956400	16219
**ACE2**	0.14564	2.5233	1.055317844033710	16221
**ANP32A**	0.14564	2.328	1.055317844033710	16223

**IRF2BP1**	0.14564	1.4928	1.055317844033710	16224
**PIGQ**	0.14564	3.2086	1.055317844033710	16220
**POGLUT1**	0.14564	2.5197	1.055317844033710	16222
**NPTN**	0.14554	2.1687	1.0557553925291100	16225
**FOXN1**	0.14523	1.9855	1.0571130791546600	16226
**SPIN1**	0.1452	3.2343	1.0572445716130000	16227
**FHL2**	0.14506	0.3842	1.0578584449403000	16228
**CGB2**	0.14491	3.0051	1.058516609041900	16229
**EML1**	0.14463	1.5055	1.0597464104192000	16230
**PAX3**	0.1446	3.2906	1.0598782699320800	16231
**CDCA7L**	0.14435	2.8288	1.0609778166596400	16236
**DCX**	0.14435	3.7233	1.0609778166596400	16233
**HES4**	0.14435	4.0434	1.0609778166596400	16232
**LGI3**	0.14435	1.325	1.0609778166596400	16238
**MARCKS**	0.14435	1.3678	1.0609778166596400	16237
**SDF2L1**	0.14435	3.0495	1.0609778166596400	16235
**TAS2R41**	0.14435	3.3234	1.0609778166596400	16234
**KLHL31**	0.14415	0.78243	1.0618583784739200	16242
**LCAT**	0.14415	0.76787	1.0618583784739200	16243
**NEUROD6**	0.14415	2.316	1.0618583784739200	16240
**OMA1**	0.14415	0.59517	1.0618583784739200	16244
**UPK2**	0.14415	2.3476	1.0618583784739200	16239
**VRTN**	0.14415	1.1448	1.0618583784739200	16241
**ZAR1L**	0.14415	0.48499	1.0618583784739200	16245
**PRAMEF9**	0.14414	3.5661	1.061902428179370	16246
**GMDS**	0.14413	3.2985	1.0619464799454000	16247
**IL1RN**	0.14375	2.1992	1.0636219767023100	16248
**KCNJ5**	0.14375	1.3374	1.0636219767023100	16249
**C16orf92**	0.14365	3.935	1.0640633934295300	16250
**NPC2**	0.14356	1.5554	1.0644608458274700	16251
**SRD5A2**	0.14343	3.91	1.0650352407741300	16252
**DDX55**	0.14328	2.9106	1.0656984411147200	16253

**ANKRD28**	0.14326	2.6039	1.065786903246210	16254
**ZNF383**	0.14302	1.8976	1.0668491000818700	16255
**LYST**	0.14295	3.5859	1.0671591343102300	16256
**INPP5D**	0.14294	2.8165	1.0672034332883600	16258
**TYMP**	0.14294	2.8983	1.0672034332883600	16257
**SPINK2**	0.14283	1.4203	1.0676908603394200	16259
**LARP4**	0.14272	2.4212	1.0681785411897500	16260
**CLPTM1**	0.14266	3.3504	1.0684446560356200	16261
**AMY1A**	0.14261	2.0777	1.0686664762199300	16265
**CCDC115**	0.14261	2.0273	1.0686664762199300	16266
**GABRA5**	0.14261	2.1909	1.0686664762199300	16263
**NXPH3**	0.14261	2.8056	1.0686664762199300	16262
**TMEM212**	0.14261	2.0985	1.0686664762199300	16264
**TTC9B**	0.14261	1.5478	1.0686664762199300	16267
**ZNF286B**	0.14261	1.1263	1.0686664762199300	16268
**ABCC12**	0.14256	2.889	1.0688883489995200	16269
**TUBG1**	0.14255	1.5217	1.0689327298700300	16270
**TGIF2**	0.14241	2.9915	1.0695542832689200	16271
**C9orf57**	0.14235	3.987	1.0698207898245700	16272
**CATSPERE**	0.14235	2.6424	1.0698207898245700	16280
**CD28**	0.14235	1.9816	1.0698207898245700	16281
**KCNQ2**	0.14235	3.5092	1.0698207898245700	16274
**LOC1005064**	0.14235	1.9188	1.0698207898245700	16282
**MICAL3**	0.14235	3.3335	1.0698207898245700	16278
**NDUFB8**	0.14235	1.3916	1.0698207898245700	16283
**NSUN3**	0.14235	3.3704	1.0698207898245700	16277
**PDE4DIP**	0.14235	3.1813	1.0698207898245700	16279
**SNRPD1**	0.14235	3.4826	1.0698207898245700	16275
**SPDYE3**	0.14235	3.5653	1.0698207898245700	16273
**TMUB1**	0.14235	3.4079	1.0698207898245700	16276
**BPIFC**	0.14234	2.0655	1.0698652149716700	16284
**LCE3E**	0.14222	2.6185	1.0703984815224700	16285

**C8A**	0.14183	3.2826	1.0721337038155400	16286
**ZNF703**	0.14166	2.3967	1.0728910938975000	16287
**C4orf36**	0.14139	4.1306	1.0740952741438300	16288
**C6orf106**	0.14139	2.5274	1.0740952741438300	16291
**CARD9**	0.14139	1.9461	1.0740952741438300	16292
**CDHR2**	0.14139	3.4533	1.0740952741438300	16289
**PCDHA12**	0.14139	2.7556	1.0740952741438300	16290
**SPINK8**	0.14139	1.1715	1.0740952741438300	16293
**DIRAS3**	0.14131	3.5651	1.0744523675241200	16294
**FBXO8**	0.14129	3.8214	1.07454166227924	16295
**DNAJC5G**	0.14128	3.1814	1.0745863128699200	16296
**CD200R1L**	0.14111	1.274	1.0753457009510900	16300
**CYLD**	0.14111	0.074424	1.0753457009510900	16301
**LOC100130**	0.14111	1.5743	1.0753457009510900	16299
**RPL31**	0.14111	3.5832	1.0753457009510900	16298
**VEZT**	0.14111	3.6749	1.0753457009510900	16297
**MED21**	0.14106	2.6894	1.0755691684339100	16302
**IL20RA**	0.14082	1.7306	1.0766425608470400	16303
**CHGA**	0.14078	3.7887	1.0768215802128600	16304
**TMEM18**	0.14077	2.9379	1.0768663404467800	16305
**HIST1H2BH**	0.14073	1.2023	1.0770454029612300	16306
**RAB13**	0.14051	1.6501	1.0780308647111700	16307
**CEP164**	0.14043	2.6108	1.0783894741119100	16308
**NXPE3**	0.14024	4.1405	1.0792417277453200	16309
**ANTXR2**	0.14012	3.0569	1.0797803973279700	16313
**BOD1L1**	0.14012	3.471	1.0797803973279700	16311
**FOXK1**	0.14012	2.6831	1.0797803973279700	16314
**KRTAP9-4**	0.14012	3.4624	1.0797803973279700	16312
**PNPLA5**	0.14012	3.6514	1.0797803973279700	16310
**SLFN11**	0.14012	2.5556	1.0797803973279700	16315
**CLK2**	0.14004	3.6924	1.0801396845155600	16316
**RBFA**	0.13996	3.4739	1.0804991111894400	16317

**VPS26B**	0.13996	2.1583	1.0804991111894400	16318
**NOD2**	0.13987	1.3094	1.0809036331200300	16319
**USP36**	0.13986	3.7134	1.0809485909202500	16320
**NSMAF**	0.13979	3.3733	1.081263356716610	16321
**FOXN3**	0.13976	2.1733	1.081398289143240	16322
**ASCC3**	0.13968	2.994	1.081758205239950	16323
**C9orf24**	0.1396	3.6362	1.082118261521620	16324
**CLSPN**	0.1396	1.8083	1.082118261521620	16329
**COA4**	0.1396	1.4318	1.082118261521620	16330
**GBP3**	0.1396	1.8468	1.082118261521620	16328
**OBSCN**	0.1396	0.023133	1.082118261521620	16331
**PPP1R7**	0.1396	2.5356	1.082118261521620	16326
**TFF2**	0.1396	2.2018	1.082118261521620	16327
**USP18**	0.1396	2.7077	1.082118261521620	16325
**GEMIN5**	0.13957	5.4659	1.0822533188005100	16332
**DUOX2**	0.13946	2.975	1.0827486978038400	16333
**PCDH17**	0.13943	3.8393	1.0828838472903800	16334
**UGT2B15**	0.13918	1.1588	1.0840108631041300	16335
**NIPSNAP2**	0.13889	4.1508	1.0853199288240000	16336
**GPR37**	0.13862	3.1143	1.0865403882564900	16337
**EHD4**	0.13857	3.0322	1.086766576920650	16338
**FUOM**	0.13857	1.6665	1.086766576920650	16339
**APBA3**	0.13854	1.9698	1.0869023168113800	16341
**C1QC**	0.13854	2.5077	1.0869023168113800	16340
**OTX1**	0.13854	0.75603	1.0869023168113800	16342
**BEND6**	0.13849	0.86325	1.087128594476650	16343
**ACRV1**	0.13829	3.6264	1.0880342622914000	16346
**AFDN**	0.13829	3.9631	1.0880342622914000	16345
**C1orf68**	0.13829	3.5503	1.0880342622914000	16347
**GPHB5**	0.13829	1.5656	1.0880342622914000	16350
**PIK3C3**	0.13829	4.0021	1.0880342622914000	16344
**PTPRG**	0.13829	1.7077	1.0880342622914000	16349

**ST8SIA4**	0.13829	2.6868	1.0880342622914000	16348
**UGT2A2**	0.13814	0.19323	1.0887140992582200	16351
**SLITRK5**	0.13808	2.9391	1.0889861749802000	16352
**HEY1**	0.13792	2.8564	1.0897121046450300	16354
**SUCNR1**	0.13792	1.845	1.0897121046450300	16355
**TMEM244**	0.13792	2.921	1.0897121046450300	16353
**EHMT1**	0.13789	1.1319	1.0898482803989200	16356
**TRIM2**	0.13786	2.4317	1.0899844763657500	16357
**BHLHA9**	0.13772	1.3412	1.090620325055110	16358
**MRPL28**	0.13765	3.1296	1.0909384148131500	16359
**C7orf71**	0.13757	1.3636	1.091302081180380	16360
**PLAUR**	0.13744	1.8024	1.091893347046430	16361
**KLK13**	0.13743	2.7646	1.0919388448483200	16362
**TFCP2L1**	0.13732	3.5162	1.092439469930850	16363
**AFTPH**	0.13728	2.3472	1.0926215833127100	16364
**RGS8**	0.13728	2.1845	1.0926215833127100	16365
**STX4**	0.13704	2.3769	1.0937150254510100	16366
**CTDNEP1**	0.13702	0.47391	1.0938062046650000	16369
**FOXD4**	0.13702	0.018989	1.0938062046650000	16370
**PGGHG**	0.13702	0.72997	1.0938062046650000	16368
**VEGFD**	0.13702	1.5102	1.0938062046650000	16367
**SLC11A1**	0.13684	1.5033	1.0946272271487100	16371
**DAP3**	0.1368	2.5114	1.0948097768170700	16372
**IL21**	0.13678	4.0365	1.0949010653341300	16373
**EVC2**	0.1367	1.6727	1.095266310681830	16374
**EMILIN1**	0.13658	4.0927	1.0958144528266200	16375
**SLC30A9**	0.13658	3.4237	1.0958144528266200	16377
**TBRG4**	0.13658	3.5435	1.0958144528266200	16376
**TMEM240**	0.13658	2.8885	1.0958144528266200	16378
**TMTC3**	0.13655	4.0148	1.0959515398168100	16379
**ACKR4**	0.1362	2.546	1.097552412389170	16381
**PPP2R3C**	0.1362	4.0321	1.097552412389170	16380

**TNFRSF4**	0.13613	1.7119	1.0978729245847300	16382
**FBXW9**	0.13609	1.8514	1.0980561250583100	16384
**HNRNPCL4**	0.13609	3.3217	1.0980561250583100	16383
**PPM1N**	0.13609	1.3153	1.0980561250583100	16386
**SLC26A7**	0.13609	1.2604	1.0980561250583100	16387
**TMEM104**	0.13609	1.382	1.0980561250583100	16385
**LIPT2**	0.13588	3.771	1.0990185327801700	16388
**RXRG**	0.13575	1.9817	1.0996148194598600	16389
**SLC35E3**	0.13572	3.355	1.0997524796115100	16390
**AHNAK**	0.13536	3.9264	1.1014060298034900	16391
**PABPN1**	0.13536	1.4986	1.1014060298034900	16393
**PSG9**	0.13536	2.3205	1.1014060298034900	16392
**YDJC**	0.13536	1.4453	1.1014060298034900	16394
**CSRNP1**	0.13532	2.9744	1.1015899435524000	16395
**HGH1**	0.13524	2.2304	1.10195788287549	16396
**BCL6B**	0.1352	2.6284	1.1021419084923700	16397
**UFL1**	0.13505	1.8918	1.102832337218840	16398
**ADAMTS18**	0.13492	3.4024	1.10343113430668	16402
**ATG4B**	0.13492	1.2398	1.10343113430668	16405
**CES4A**	0.13492	3.531	1.10343113430668	16400
**KLHL32**	0.13492	4.1311	1.10343113430668	16399
**RASGRP2**	0.13492	3.4637	1.10343113430668	16401
**TCHP**	0.13492	3.1672	1.10343113430668	16404
**TIMM23**	0.13492	3.2601	1.10343113430668	16403
**UCHL1**	0.13491	3.7872	1.1034772120101600	16406
**ATXN7L3B**	0.13485	5.5273	1.1037537274451900	16407
**DRD2**	0.1345	1.1771	1.1053684190166900	16410
**GPD1L**	0.1345	1.8041	1.1053684190166900	16409
**IHH**	0.1345	1.8085	1.1053684190166900	16408
**FAM69C**	0.13448	1.1676	1.1054607741707200	16411
**C12orf73**	0.13438	3.1118	1.1059226914445000	16412
**SMARCC1**	0.13436	2.2386	1.1060151032189800	16413

**TSPY8**	0.13433	4.2255	1.1061537385932900	16414
**ARMCX5-GI**	0.13421	1.681	1.1067084928150200	16415
**SERPINA4**	0.13421	0.31656	1.1067084928150200	16416
**TMEM55B**	0.13418	3.6578	1.1068472345975700	16417
**GRAP2**	0.13407	3.0474	1.1073561368496700	16418
**KCNIP4**	0.13388	3.7086	1.1082358259559000	16419
**FABP3**	0.13387	2.3015	1.108282149141640	16420
**CT45A7**	0.13378	5.5423	1.1086991648787200	16421
**C1QTNF6**	0.13364	1.9449	1.1093482395246100	16423
**FAM103A1**	0.13364	0.24187	1.1093482395246100	16424
**TELO2**	0.13364	2.548	1.1093482395246100	16422
**FNDC11**	0.13362	4.0192	1.1094410026317200	16425
**ZNF827**	0.13362	2.9495	1.1094410026317200	16426
**TP53INP1**	0.13357	0.17212	1.1096729521767400	16428
**VSIR**	0.13357	0.59343	1.1096729521767400	16427
**DLL4**	0.13346	2.8034	1.110183451438330	16429
**BOP 1.00**	0.13326	4.3615	1.1111123739005400	16430
**CSF2RB**	0.13326	3.3705	1.1111123739005400	16433
**FUT8**	0.13326	3.541	1.1111123739005400	16431
**NUP155**	0.13326	3.4637	1.1111123739005400	16432
**SLC30A8**	0.13319	3.153	1.111437723364290	16434
**MATN1**	0.13314	2.9686	1.111670187886920	16435
**GMPPB**	0.13308	3.9774	1.1119492246365300	16436
**NIPSNAP3B**	0.13308	2.3558	1.1119492246365300	16437
**CCT4**	0.13294	0.5803	1.1126006473127600	16440
**FOXD3**	0.13294	2.4167	1.1126006473127600	16439
**SLC26A8**	0.13294	2.9711	1.1126006473127600	16438
**EXT1**	0.13284	0.11233	1.1130662384391300	16442
**HAMP**	0.13284	0.12646	1.1130662384391300	16441
**LOC388813**	0.13281	3.8495	1.113205962836000	16443
**TMEM191C**	0.13278	3.1265	1.1133457089692400	16444
**LOC100505**	0.13276	5.5571	1.1134388851385200	16445

**RUNDC3A**	0.13256	2.939	1.1143711790168300	16446
**COX7C**	0.13254	3.0711	1.114464461685280	16447
**NCALD**	0.13247	3.9435	1.1147910274205600	16448
**BACE2**	0.13244	0.84618	1.1149310205644000	16456
**DDR1**	0.13244	0.21918	1.1149310205644000	16458
**MAP1LC3A**	0.13244	2.2168	1.1149310205644000	16452
**MUC17**	0.13244	1.9835	1.1149310205644000	16453
**MYRIP**	0.13244	1.6653	1.1149310205644000	16454
**RGS3**	0.13244	2.8336	1.1149310205644000	16449
**SHISA6**	0.13244	1.1729	1.1149310205644000	16455
**SPINK7**	0.13244	2.2326	1.1149310205644000	16451
**XYLB**	0.13244	0.35073	1.1149310205644000	16457
**ZBED6**	0.13244	2.8019	1.1149310205644000	16450
**C6orf48**	0.13231	3.6595	1.1155379101778900	16459
**B4GALT6**	0.13229	3.0927	1.115631314283560	16460
**U2AF2**	0.13221	2.6281	1.116005028076050	16461
**NXT1**	0.13215	2.6215	1.1162854157384500	16462
**REG3G**	0.13211	3.6371	1.1164723896123400	16463
**ADAM32**	0.13185	2.5052	1.1176886723933900	16465
**CTSZ**	0.13185	3.3496	1.1176886723933900	16464
**OR2W5**	0.13181	5.5717	1.117875939618560	16466
**APLP2**	0.1317	1.3522	1.1183911267385700	16467
**INSM1**	0.13168	2.7798	1.1184848290203400	16468
**FAM231C**	0.13167	3.6314	1.118531683844080	16470
**FOXC1**	0.13167	2.1855	1.118531683844080	16475
**GATA2**	0.13167	3.2078	1.118531683844080	16474
**PSAP**	0.13167	3.6507	1.118531683844080	16469
**SHMT2**	0.13167	3.4816	1.118531683844080	16473
**TUBGCP3**	0.13167	3.5789	1.118531683844080	16471
**ZSCAN12**	0.13167	3.5009	1.118531683844080	16472
**ZNF488**	0.13165	3.1181	1.1186254008591000	16476
**KLHL41**	0.13155	3.9682	1.1190941333784900	16477

**CABP1**	0.13143	3.0356	1.1196569371604200	16478
**MEIS2**	0.13141	2.6471	1.1197507722753400	16479
**CNNM4**	0.13135	2.9632	1.1200323367944700	16481
**FURIN**	0.13135	2.2219	1.1200323367944700	16482
**RTN1**	0.13135	0.30029	1.1200323367944700	16483
**SPO11**	0.13135	3.9503	1.1200323367944700	16480
**NANOS1**	0.1309	1.892	1.1221469073304700	16485
**TECTA**	0.1309	2.2032	1.1221469073304700	16484
**CCDC30**	0.13077	0.14262	1.1227587181478100	16493
**COLEC11**	0.13077	2.601	1.1227587181478100	16487
**DNAH17**	0.13077	2.6624	1.1227587181478100	16486
**EVA1A**	0.13077	1.0273	1.1227587181478100	16492
**GALP**	0.13077	2.3844	1.1227587181478100	16488
**MYOC**	0.13077	2.296	1.1227587181478100	16490
**TNFAIP2**	0.13077	1.1861	1.1227587181478100	16491
**TSHB**	0.13077	2.3353	1.1227587181478100	16489
**MYOCD**	0.1307	3.3792	1.123088328883080	16494
**ZBTB8B**	0.13045	3.084	1.1242665073813800	16495
**CIB2**	0.13044	2.2811	1.1243136669863400	16496
**C8orf37**	0.13034	2.2055	1.124785400632000	16497
**NPY2R**	0.13016	2.7813	1.1256351525376500	16498
**ZEB2**	0.13013	3.0844	1.1257768568941100	16499
**FAM198B**	0.12997	3.5506	1.126532995553940	16504
**FAM83G**	0.12997	3.5522	1.126532995553940	16503
**FLOT2**	0.12997	3.6996	1.126532995553940	16501
**IL17RB**	0.12997	3.566	1.126532995553940	16502
**IL9**	0.12997	3.9106	1.126532995553940	16500
**OR5K3**	0.12997	1.4795	1.126532995553940	16508
**PPARGC1A**	0.12997	3.4356	1.126532995553940	16505
**TMEM155**	0.12997	2.3093	1.126532995553940	16506
**VIP**	0.12997	1.9494	1.126532995553940	16507
**EIF6**	0.12991	0.27193	1.126816713665080	16510

**EPB41L1**	0.12991	0.15401	1.126816713665080	16511
**SEC61B**	0.12991	2.5252	1.126816713665080	16509
**OR4K13**	0.12985	4.2007	1.1271005225093500	16512
**STAC**	0.12983	2.9652	1.127195145634340	16513
**ZNF729**	0.12983	1.7882	1.127195145634340	16514
**RAI2**	0.12956	1.0263	1.1284735469435700	16515
**OR56A4**	0.12955	3.3725	1.128520930563770	16516
**SECISBP2L**	0.12954	3.7855	1.1285683167178600	16517
**FAM219A**	0.1295	0.84086	1.1287578866807600	16518
**LHFP**	0.12949	2.7636	1.1288052855100000	16519
**HOXB1**	0.1294	3.3985	1.1292319891458700	16520
**CBLN3**	0.12939	0.89291	1.1292794133537600	16522
**EIF1AX**	0.12939	0.86949	1.1292794133537600	16523
**TATDN1**	0.12939	2.1651	1.1292794133537600	16521
**GRIP2**	0.12904	3.2687	1.1309408635050000	16524
**NDUFS8**	0.12898	3.3347	1.1312259971281200	16525
**YAE1D1**	0.12892	3.5024	1.1315112227508500	16526
**SEC61A1**	0.12884	1.7311	1.1318916668344400	16527
**ASNA1**	0.12868	1.5688	1.1326530468915900	16528
**BEND7**	0.12865	4.2309	1.1327958787657100	16529
**C2orf72**	0.12843	3.6466	1.1338440194363400	16530
**FRMD1**	0.12834	2.1221	1.1342731634713600	16535
**KRTAP10-9**	0.12834	3.9513	1.1342731634713600	16533
**PODNL1**	0.12834	4.1978	1.1342731634713600	16531
**RFXAP**	0.12834	3.2133	1.1342731634713600	16534
**TECRL**	0.12834	1.2963	1.1342731634713600	16536
**YAP1**	0.12834	4.0987	1.1342731634713600	16532
**DCAF10**	0.12832	2.469	1.1343685571845000	16537
**IDO1**	0.12823	3.836	1.1347979566956700	16538
**ONECUT2**	0.12818	2.894	1.13503660241722	16539
**RPL12**	0.12813	2.7459	1.1352753127985900	16540
**SAP18**	0.12811	4.3894	1.1353708150667800	16541

**ADH1A**	0.12809	1.6355	1.1354663276914400	16546
**AP5Z1**	0.12809	0.82615	1.1354663276914400	16548
**GSKIP**	0.12809	2.3021	1.1354663276914400	16544
**IKZF4**	0.12809	1.7474	1.1354663276914400	16545
**MAPK9**	0.12809	2.8683	1.1354663276914400	16542
**P2RX6**	0.12809	0.9814	1.1354663276914400	16547
**PI16**	0.12809	2.5655	1.1354663276914400	16543
**NANOGNB**	0.12807	3.7443	1.1355618506756900	16549
**ITGB3BP**	0.12804	0.98848	1.1357051545831300	16550
**CXCL2**	0.12802	2.5515	1.1358007034800000	16551
**HENMT1**	0.12797	0.47265	1.1360396211000200	16553
**RAB2A**	0.12797	1.8623	1.1360396211000200	16552
**SIDT2**	0.12795	3.3952	1.136135206307370	16554
**GALNT13**	0.1279	2.9551	1.1363742147514300	16555
**C3orf35**	0.12777	2.7469	1.1369959407146600	16556
**ZNF705G**	0.12756	5.6288	1.138001196708000	16557
**TTC34**	0.12733	3.541	1.139103512506870	16558
**ASB13**	0.12727	3.8806	1.1393913008754900	16559
**MARCH7**	0.12724	2.3957	1.1395352304535700	16560
**VGF**	0.12717	4.2051	1.1398711579715700	16561
**KCNK18**	0.12698	2.5874	1.1407836102833100	16562
**ARL 14.00**	0.12677	3.9737	1.1417932164783800	16564
**CRYGA**	0.12677	4.1462	1.1417932164783800	16563
**CYP4F12**	0.12677	3.9674	1.1417932164783800	16565
**DUOXA2**	0.12677	3.574	1.1417932164783800	16566
**TLE2**	0.12677	3.2651	1.1417932164783800	16567
**DCAF8L2**	0.12668	0.72597	1.1422262613178100	16571
**GATA1**	0.12668	2.8718	1.1422262613178100	16569
**MUC21**	0.12668	1.2428	1.1422262613178100	16570
**POLE4**	0.12668	3.0174	1.1422262613178100	16568
**BLVRB**	0.12658	1.4936	1.142707673608700	16572
**MCU**	0.12639	2.971	1.1436230873066000	16573

**C21orf2**	0.12613	1.1611	1.1448773138448800	16574
**CLSTN3**	0.12604	2.51	1.1453118890335300	16575
**TRIM41**	0.12604	1.9392	1.1453118890335300	16576
**MCOLN3**	0.12568	5.6566	1.14705235683129	16577
**TMEM131**	0.12562	3.8369	1.1473427727710800	16578
**TMEM267**	0.12547	2.4515	1.1480692363185000	16579
**MYO10**	0.12543	4.4476	1.1482630622874000	16580
**DERL2**	0.12541	3.9388	1.148359991450740	16581
**CCDC50**	0.12517	1.7203	1.1495239840057800	16583
**MEIOB**	0.12517	3.5798	1.1495239840057800	16582
**ARG2**	0.12508	2.1432	1.149960882976510	16588
**BARX1**	0.12508	3.8146	1.149960882976510	16587
**LURAP1**	0.12508	1.9457	1.149960882976510	16589
**NAALADL2**	0.12508	3.996	1.149960882976510	16585
**TBX10**	0.12508	3.8521	1.149960882976510	16586
**TLR2**	0.12508	4.2341	1.149960882976510	16584
**WASHC2A**	0.12501	2.6005	1.1503008451044500	16590
**LOC1019293**	0.12483	1.1085	1.1511756444793700	16591
**GRIK2**	0.12477	2.9923	1.1514674401172900	16593
**HAUS2**	0.12477	2.5858	1.1514674401172900	16595
**OR5V1**	0.12477	2.6258	1.1514674401172900	16594
**SPATA33**	0.12477	3.6432	1.1514674401172900	16592
**PES 1.00**	0.1247	2.8176	1.1518079923228200	16596
**TUFT1**	0.12455	2.0128	1.1525381972269400	16597
**TBX6**	0.12427	2.7435	1.1539028930845100	16598
**MPDZ**	0.12418	2.2482	1.1543420019889200	16599
**ETS2**	0.12411	3.502	1.1546836850787500	16600
**RMND5B**	0.12408	2.2858	1.1548301619636100	16601
**FUS**	0.12402	4.3111	1.1551231900896900	16602
**GZMB**	0.12387	2.7289	1.1558561946889600	16603
**CD2**	0.1238	1.3154	1.1561984761264400	16604
**RNF146**	0.12372	2.88	1.15658982085109	16605

**ASIC3**	0.12357	2.4727	1.1573240700266100	16606
**LAPTM4B**	0.1235	1.9602	1.1576669332602700	16611
**MORN2**	0.1235	2.665	1.1576669332602700	16608
**MYCT1**	0.1235	2.2978	1.1576669332602700	16610
**RAC3**	0.1235	3.8686	1.1576669332602700	16607
**WDR34**	0.1235	2.4992	1.1576669332602700	16609
**ZNF114**	0.12344	1.9974	1.1579609243856100	16612
**UCK1**	0.12341	3.9183	1.158107957486490	16613
**HTRA4**	0.12336	4.2163	1.158353068307230	16614
**SFTPC**	0.12328	3.1592	1.1587453904375500	16615
**CLCA2**	0.12326	1.5832	1.158843498841300	16620
**CPA4**	0.12326	4.0245	1.158843498841300	16618
**LYPD2**	0.12326	4.1528	1.158843498841300	16616
**PLEKHF2**	0.12326	1.2316	1.158843498841300	16621
**PVRIG**	0.12326	3.8394	1.158843498841300	16619
**SLC2A2**	0.12326	4.0435	1.158843498841300	16617
**XPO1**	0.12319	3.2444	1.159186966126300	16622
**OAS3**	0.12302	2.7283	1.1600216708067800	16623
**SGO1**	0.123	2.9012	1.1601199245031600	16624
**TOMM6**	0.12278	5.6972	1.1612014551890300	16625
**C2orf78**	0.12219	2.9193	1.1641086510327800	16626
**METAP1**	0.12213	3.9013	1.1644048495567200	16627
**C2orf50**	0.12206	0.28062	1.1647505436681400	16632
**CAMSAP1**	0.12206	2.7956	1.1647505436681400	16631
**CCDC90B**	0.12206	3.1636	1.1647505436681400	16630
**RABAC1**	0.12206	4.2677	1.1647505436681400	16628
**RADIL**	0.12206	3.7402	1.1647505436681400	16629
**CT45A1**	0.12191	1.759	1.165491785860140	16633
**CTSV**	0.12182	3.9344	1.1659368387204400	16634
**FAM107B**	0.12182	2.8703	1.1659368387204400	16637
**KRTAP9-2**	0.12182	3.7976	1.1659368387204400	16635
**ZNF283**	0.12182	3.2295	1.1659368387204400	16636

**DUS1L**	0.12171	1.1655	1.1664811060335200	16638
**EHD3**	0.12168	2.9283	1.1666296025565300	16640
**POLR2K**	0.12168	5.7105	1.1666296025565300	16639
**CYFIP2**	0.1215	4.2498	1.1675211224525800	16641
**BATF**	0.12128	3.4461	1.1686120196454700	16648
**BPHL**	0.12128	3.9874	1.1686120196454700	16643
**CRLS1**	0.12128	3.9175	1.1686120196454700	16644
**DEUP1**	0.12128	3.8677	1.1686120196454700	16645
**DUSP21**	0.12128	2.6352	1.1686120196454700	16651
**GANC**	0.12128	4.208	1.1686120196454700	16642
**NMBR**	0.12128	3.0726	1.1686120196454700	16650
**PARP15**	0.12128	2.0777	1.1686120196454700	16652
**PPY**	0.12128	3.4904	1.1686120196454700	16647
**UNC13D**	0.12128	2.0248	1.1686120196454700	16653
**WIPI2**	0.12128	3.1713	1.1686120196454700	16649
**ZNF101**	0.12128	3.544	1.1686120196454700	16646
**LANCL2**	0.12123	1.3637	1.1688601448517400	16654
**NLGN4X**	0.12081	1.9533	1.1709472440423700	16655
**AIDA**	0.12069	2.8842	1.1715444957481500	16658
**C10orf95**	0.12069	2.5922	1.1715444957481500	16659
**FBN1**	0.12069	3.6917	1.1715444957481500	16656
**MARK4**	0.12069	3.0166	1.1715444957481500	16657
**TMEM127**	0.12069	0.14926	1.1715444957481500	16660
**DHX9**	0.12063	3.1272	1.1718432783736800	16661
**MNS1**	0.12059	3.2903	1.172042524922440	16664
**TSGA13**	0.12059	3.3012	1.172042524922440	16663
**ZNF146**	0.12059	3.4205	1.172042524922440	16662
**BCL11A**	0.12055	1.8822	1.1722418180111800	16665
**GRM2**	0.12015	2.6463	1.1742373151373600	16666
**BPTF**	0.11997	2.8494	1.1751368161213700	16667
**SLCO1C1**	0.11983	1.7839	1.1758370858996200	16668
**CORO7-PAM**	0.11982	4.2939	1.1758871272362800	16669

**BLCAP**	0.11965	3.1906	1.1767382808918600	16670
**EIF2A**	0.11962	1.7386	1.1768885729955800	16674
**NKTR**	0.11962	2.2196	1.1768885729955800	16673
**NUDT16**	0.11962	2.978	1.1768885729955800	16671
**PLA2G15**	0.11962	2.4887	1.1768885729955800	16672
**POMP**	0.11946	2.6741	1.1776905802349500	16675
**C1orf43**	0.11945	3.9691	1.1777407308426100	16679
**C7orf33**	0.11945	3.7426	1.1777407308426100	16680
**CACNG4**	0.11945	4.1019	1.1777407308426100	16677
**CLGN**	0.11945	4.0463	1.1777407308426100	16678
**DHX38**	0.11945	3.7265	1.1777407308426100	16681
**KIAA1217**	0.11945	3.3224	1.1777407308426100	16682
**KRTAP4-2**	0.11945	4.1108	1.1777407308426100	16676
**SPATA31A6**	0.11945	2.3932	1.1777407308426100	16683
**SCOC**	0.11936	1.5198	1.1781922196717300	16684
**RABGGTA**	0.11929	3.7154	1.1785435437605600	16685
**RBP1**	0.11909	2.6213	1.1795481293101400	16687
**UBR2**	0.11909	3.3603	1.1795481293101400	16686
**ADGRL4**	0.11904	1.7069	1.1797994617665700	16690
**DNAH1**	0.11904	2.2457	1.1797994617665700	16689
**HMGCR**	0.11904	3.6883	1.1797994617665700	16688
**BRAT1**	0.11885	2.4055	1.1807552054875300	16691
**RRAGC**	0.11871	4.2933	1.1814601284527900	16692
**CST2**	0.11868	0.8044	1.1816112597734200	16693
**CLDN2**	0.11848	4.0171	1.1826194923803500	16694
**CSN3**	0.11844	2.1221	1.18282128320972	16696
**WRB**	0.11844	2.2057	1.18282128320972	16695
**DOCK6**	0.11834	3.5196	1.183325971118870	16697
**CNOT9**	0.11781	3.6329	1.1860058637338100	16698
**ITGA9**	0.11781	2.8095	1.1860058637338100	16701
**SLC28A3**	0.11781	3.1134	1.1860058637338100	16700
**TEKT3**	0.11781	3.4032	1.1860058637338100	16699

**PNPT1**	0.11756	4.3044	1.1872729254190700	16702
**TAPBPL**	0.11756	2.0844	1.1872729254190700	16703
**CHAC2**	0.11736	2.6937	1.188287948853880	16705
**GBE1**	0.11736	2.5785	1.188287948853880	16706
**OBP2A**	0.11736	2.4479	1.188287948853880	16707
**PARP6**	0.11736	3.7788	1.188287948853880	16704
**RDH10**	0.11736	0.8527	1.188287948853880	16708
**STK33**	0.11734	2.4885	1.1883895185468600	16709
**TBC1D14**	0.11726	2.6591	1.1887959199726900	16710
**MEP1B**	0.11718	2.4384	1.1892025178373500	16711
**IER2**	0.11715	3.1679	1.1893550427334800	16712
**ARHGEF1**	0.11712	2.2799	1.189507595303580	16714
**TAT**	0.11712	3.5394	1.189507595303580	16713
**SPATA45**	0.11711	3.4336	1.1895584523124100	16715
**ING1**	0.11689	1.5159	1.1906780858557500	16716
**MRPS22**	0.11689	1.1715	1.1906780858557500	16717
**COX4I2**	0.11687	2.7839	1.1907799447547400	16718
**RCBTB2**	0.1168	4.3392	1.1911365482322000	16719
**CCDC86**	0.11671	2.6412	1.19159526110762	16720
**IL17RA**	0.11652	2.9371	1.1925644792503800	16721
**B3GALT6**	0.11628	4.1085	1.193790358372480	16723
**C16orf70**	0.11628	3.9074	1.193790358372480	16726
**CCDC77**	0.11628	3.4707	1.193790358372480	16728
**CCK**	0.11628	4.0031	1.193790358372480	16725
**KIAA1147**	0.11628	4.3115	1.193790358372480	16722
**OR10G4**	0.11628	3.6387	1.193790358372480	16727
**SERPINI1**	0.11628	4.0049	1.193790358372480	16724
**SNRNP70**	0.11628	2.7775	1.193790358372480	16730
**URI1**	0.11628	3.066	1.193790358372480	16729
**GLT8D1**	0.11625	3.7547	1.1939437194426900	16731
**SELL**	0.11618	1.4578	1.194301671188070	16733
**ZFR**	0.11618	3.6475	1.194301671188070	16732

**NLK**	0.11605	3.67	1.1949668450080000	16734
**CLDN6**	0.11587	0.39119	1.1958887287053100	16735
**RAX**	0.11568	1.2365	1.196862932054730	16736
**TAGLN3**	0.1156	2.0835	1.1972734629573400	16737
**FAM171A2**	0.11557	3.7132	1.1974274640792300	16738
**KLRD1**	0.11549	1.903	1.1978382726391200	16739
**CRB1**	0.11548	3.2476	1.1978896379243800	16740
**CLPS**	0.11538	2.6424	1.1984034647017700	16741
**GYPC**	0.11506	3.0711	1.2000498405029100	16742
**INPP4B**	0.11506	0.3841	1.2000498405029100	16744
**ZNF677**	0.11506	0.449	1.2000498405029100	16743
**ATG9A**	0.11498	2.9152	1.2004619430306400	16745
**SLC20A1**	0.11498	1.9302	1.2004619430306400	16746
**GRK2**	0.11495	1.3406	1.2006165340508400	16747
**LTV1**	0.11486	3.2818	1.2010804793574700	16748
**C11orf88**	0.11482	3.2305	1.2012867602785900	16752
**LYPD1**	0.11482	3.5921	1.2012867602785900	16751
**PHKA1**	0.11482	4.2834	1.2012867602785900	16749
**PRLHR**	0.11482	4.0555	1.2012867602785900	16750
**TLDC1**	0.11482	1.4734	1.2012867602785900	16754
**WDR49**	0.11482	2.3229	1.2012867602785900	16753
**XPNPEP2**	0.1148	3.9212	1.2013899199106100	16755
**NOL12**	0.11474	2.677	1.2016994755437100	16756
**MYL12A**	0.11472	4.2257	1.2018026863479900	16757
**PPBP**	0.11464	3.7721	1.2022156576453700	16758
**TRMT44**	0.11454	2.6165	1.2027321602688700	16759
**COQ5**	0.11441	3.9733	1.2034040937416300	16760
**SLC16A8**	0.1144	3.9154	1.2034558034397700	16761
**SLC37A1**	0.11438	3.8649	1.2035592324909200	16762
**IRAK4**	0.11434	5.8003	1.2037661292278000	16763
**VEZF1**	0.11423	2.6272	1.2043353611191800	16764
**CDRT15L2**	0.11408	0.49011	1.2051122158881800	16767

**CLDN15**	0.11408	3.0828	1.2051122158881800	16765
**RXFP4**	0.11408	2.7907	1.2051122158881800	16766
**SLC16A3**	0.11401	2.5489	1.2054749971298200	16768
**PNN**	0.11398	4.1698	1.205630523382340	16769
**CYB5B**	0.11366	3.3302	1.2072912879113700	16770
**APOD**	0.11361	2.7967	1.207551083310890	16771
**AASDHPPT**	0.11342	3.9714	1.2085390499459100	16773
**C1D**	0.11342	3.9624	1.2085390499459100	16774
**NPFF**	0.11342	4.3134	1.2085390499459100	16772
**POU5F2**	0.11342	3.6569	1.2085390499459100	16776
**SLC25A1**	0.11342	1.814	1.2085390499459100	16777
**TFF1**	0.11342	3.7576	1.2085390499459100	16775
**RNF212**	0.11339	1.2399	1.2086951525424900	16778
**LY6D**	0.1131	3.8991	1.2102056653137600	16779
**ZCCHC2**	0.11288	2.6782	1.2113534161353100	16780
**HAPLN1**	0.11285	1.7495	1.2115100512881200	16785
**MS4A1**	0.11285	2.3657	1.2115100512881200	16784
**NRF1**	0.11285	3.1163	1.2115100512881200	16782
**RTN4RL2**	0.11285	3.2319	1.2115100512881200	16781
**SARDH**	0.11285	1.7218	1.2115100512881200	16786
**SERF2**	0.11285	2.7194	1.2115100512881200	16783
**FBXO11**	0.11284	2.9454	1.2115622696115300	16787
**SDK1**	0.11282	1.3871	1.2116667161704200	16788
**ZNF668**	0.1128	3.9788	1.2117711759491600	16789
**PAX5**	0.11272	3.7432	1.212189147352260	16790
**CDC6**	0.11269	3.6139	1.212345941237450	16791
**SP100**	0.11261	2.2234	1.2127642040454000	16792
**ATOH8**	0.11256	1.7483	1.2130257260769900	16793
**OPLAH**	0.11253	2.2793	1.2131826791264800	16794
**GPR149**	0.11251	4.0533	1.2132873310982600	16795
**SCGB1C2**	0.11249	2.6924	1.2133919963597000	16796
**OR52A1**	0.11233	2.3429	1.2142297974216000	16797

**PADI1**	0.11231	4.4082	1.2143345824936400	16798
**BCL11B**	0.11225	3.5744	1.2146490177377800	16799
**EDN3**	0.11216	1.8711	1.215120895900410	16800
**ROR2**	0.1118	3.0254	1.217011119966170	16801
**ELF4**	0.11178	0.075106	1.2171162599573700	16804
**QKI**	0.11178	4.1305	1.2171162599573700	16802
**ZFP30**	0.11178	3.4451	1.2171162599573700	16803
**AHSG**	0.11166	3.4791	1.2177473826466900	16806
**BBS4**	0.11166	1.2202	1.2177473826466900	16809
**HCK**	0.11166	3.2786	1.2177473826466900	16807
**KALRN**	0.11166	1.5247	1.2177473826466900	16808
**NGRN**	0.11166	3.8608	1.2177473826466900	16805
**C10orf111**	0.11158	4.1467	1.2181684007229000	16810
**MRPL36**	0.11134	2.1769	1.2194327523704500	16811
**ZIC2**	0.11131	1.3341	1.2195909334377900	16812
**SMNDC1**	0.11124	2.4541	1.219960141317070	16813
**TMEM207**	0.11123	3.9034	1.220012898876630	16814
**CPT1C**	0.11101	2.6384	1.2211744253985300	16815
**COG4**	0.11089	0.28077	1.2218086803238800	16823
**DDX39B**	0.11089	4.3112	1.2218086803238800	16816
**FKBP3**	0.11089	2.7358	1.2218086803238800	16818
**FZD8**	0.11089	4.2199	1.2218086803238800	16817
**HIF1AN**	0.11089	2.5702	1.2218086803238800	16819
**IPO8**	0.11089	2.3722	1.2218086803238800	16820
**MOGS**	0.11089	0.8849	1.2218086803238800	16821
**RHAG**	0.11089	0.62506	1.2218086803238800	16822
**HPSE**	0.11085	2.8633	1.2220202078907900	16824
**C19orf81**	0.11083	1.7922	1.2221259921814100	16825
**SYNJ2BP-C**	0.11082	3.4092	1.2221788894555700	16826
**GPR50**	0.11063	2.9155	1.2231845881430900	16827
**PNLIPRP1**	0.11049	3.8513	1.2239264216763900	16828
**SCARA5**	0.11041	4.202	1.224350629131540	16829

**VGLL3**	0.11037	3.5595	1.2245628155045200	16830
**SCRN2**	0.11029	4.4246	1.2249873537323200	16831
**ABCG4**	0.1102	0.95285	1.225465223295490	16832
**BARHL2**	0.11011	3.3933	1.2259433728689100	16833
**DDIT4L**	0.11005	4.1607	1.226262295028590	16835
**RIPK2**	0.11005	3.4702	1.226262295028590	16839
**RNF219**	0.11005	1.8837	1.226262295028590	16841
**ST6GALNAC**	0.11005	3.8604	1.226262295028590	16837
**TNFRSF18**	0.11005	3.16	1.226262295028590	16840
**TNFRSF9**	0.11005	4.4762	1.226262295028590	16834
**TOLLIP**	0.11005	3.8774	1.226262295028590	16836
**ZIC4**	0.11005	3.6883	1.226262295028590	16838
**ABCA13**	0.10967	1.7264	1.2282850382748300	16843
**FMC1**	0.10967	0.46327	1.2282850382748300	16845
**GDI1**	0.10967	2.8748	1.2282850382748300	16842
**TMPO**	0.10967	0.99992	1.2282850382748300	16844
**IRX5**	0.1096	4.3213	1.2286581973915900	16846
**DDI1**	0.10957	3.2799	1.2288181751130000	16847
**MAPKAPK5**	0.10956	3.3742	1.2288715080093500	16848
**OR3A3**	0.10949	2.7025	1.2292449361955500	16849
**ATG101**	0.10905	3.5009	1.2315961344491800	16852
**CEP95**	0.10905	3.938	1.2315961344491800	16850
**DYNC1LI1**	0.10905	3.6768	1.2315961344491800	16851
**ETV3**	0.10905	2.0123	1.2315961344491800	16854
**MIA**	0.10905	2.7549	1.2315961344491800	16853
**SHKBP1**	0.10894	4.0313	1.2321849988621400	16855
**NXPH4**	0.10882	1.0623	1.2328278840900600	16856
**ARMCX3**	0.10872	0.87463	1.2333640112668800	16862
**ETV3L**	0.10872	3.5533	1.2333640112668800	16857
**FBXL6**	0.10872	2.3176	1.2333640112668800	16859
**LCNL1**	0.10872	0.88492	1.2333640112668800	16861
**NONO**	0.10872	3.3084	1.2333640112668800	16858

**PDE6A**	0.10872	1.7049	1.2333640112668800	16860
**KCNH3**	0.10866	2.3414	1.233685857809770	16863
**NRAS**	0.10852	3.4578	1.2344373304743500	16864
**CERKL**	0.1085	2.3576	1.2345447406310100	16867
**CXorf67**	0.1085	1.9771	1.2345447406310100	16869
**LGR6**	0.1085	2.0483	1.2345447406310100	16868
**SLC18A1**	0.1085	3.7692	1.2345447406310100	16866
**SPC25**	0.1085	3.934	1.2345447406310100	16865
**CTSB**	0.10848	1.473	1.2346521650324300	16870
**KIAA0391**	0.10821	4.3833	1.2361037911988500	16871
**ANKRA2**	0.10811	3.9808	1.2366420920562100	16872
**CD177**	0.10806	0.15548	1.2369113769118900	16874
**HIF3A**	0.10806	3.2159	1.2369113769118900	16873
**GLRB**	0.10803	4.199	1.237072990887570	16875
**CCDC71**	0.108	3.8922	1.2372346371809000	16876
**GBA2**	0.10781	4.0111	1.2382591486339800	16877
**ZNF667**	0.10779	2.9504	1.2383670675710100	16878
**CLASRP**	0.1077	2.047	1.238852881365440	16881
**DAZAP2**	0.1077	0.62156	1.238852881365440	16882
**NDUFA5**	0.1077	2.1115	1.238852881365440	16880
**SENP7**	0.1077	2.5863	1.238852881365440	16879
**PRDM7**	0.10753	3.8733	1.2397713282516400	16883
**HS3ST1**	0.10745	2.9495	1.2402039006397000	16884
**EPHX2**	0.1074	2.8693	1.2404743762702700	16885
**SEC23IP**	0.10737	3.48	1.2406367052177900	16886
**AP2M1**	0.10725	3.1996	1.241286348164500	16887
**BANF2**	0.1071	3.5457	1.2420991392622700	16890
**BLID**	0.1071	4.157	1.2420991392622700	16888
**GNL3L**	0.1071	1.8755	1.2420991392622700	16892
**NCF2**	0.1071	3.5649	1.2420991392622700	16889
**OR2M5**	0.1071	3.5099	1.2420991392622700	16891
**GPR26**	0.10694	2.2911	1.2429670218483000	16893

**PCDHA9**	0.10687	2.4404	1.2433470150393700	16895
**WDR75**	0.10687	3.5938	1.2433470150393700	16894
**SUN1**	0.10675	1.4834	1.2439988499488900	16896
**BMP4**	0.10674	2.8297	1.244053193387040	16897
**ABCF3**	0.10668	1.8252	1.2443793311970600	16898
**PANK2**	0.10659	1.6322	1.2448687862312600	16899
**CCDC117**	0.10643	3.6126	1.2457396656380000	16901
**CXorf58**	0.10643	2.5775	1.2457396656380000	16902
**EHBP1L1**	0.10643	1.4409	1.2457396656380000	16904
**FAM46C**	0.10643	1.2845	1.2457396656380000	16905
**LCN6**	0.10643	4.3046	1.2457396656380000	16900
**TPPP3**	0.10643	2.0928	1.2457396656380000	16903
**STX5**	0.10638	3.9037	1.2460120092947200	16906
**SLC2A10**	0.10622	2.8761	1.2468841306653300	16907
**TBCD**	0.10611	4.2038	1.2474842646727200	16908
**SP4**	0.10606	1.013	1.2477572014428900	16909
**BACE1**	0.10605	3.3587	1.24781179995216	16910
**OR2T1**	0.10604	1.8614	1.2478664021814100	16911
**OR4K1**	0.10582	4.3985	1.249068593736150	16912
**AGTPBP1**	0.10575	1.2127	1.2494514880351500	16924
**C19orf18**	0.10575	3.2575	1.2494514880351500	16922
**ERRFI1**	0.10575	4.1612	1.2494514880351500	16914
**FGF6**	0.10575	3.192	1.2494514880351500	16923
**KRTAP4-3**	0.10575	3.6059	1.2494514880351500	16919
**LUZP6**	0.10575	3.5951	1.2494514880351500	16920
**MMP9**	0.10575	4.1902	1.2494514880351500	16913
**OR1C1**	0.10575	3.9974	1.2494514880351500	16917
**RAPSN**	0.10575	3.8844	1.2494514880351500	16918
**RBM15**	0.10575	4.0195	1.2494514880351500	16916
**TCEAL8**	0.10575	4.1295	1.2494514880351500	16915
**TXLNB**	0.10575	3.4727	1.2494514880351500	16921
**IL4**	0.10573	2.8077	1.2495609200575000	16925

**OCEL1**	0.10548	3.6691	1.2509300848431600	16926
**SPANXB1**	0.10545	4.6314	1.2510945422629200	16927
**GSC**	0.10526	2.9244	1.2521368926222600	16928
**APOL2**	0.10523	4.9575	1.252301598661540	16929
**ZNF443**	0.10523	4.3594	1.252301598661540	16930
**ZNF512B**	0.10523	3.2972	1.252301598661540	16931
**DGCR14**	0.1051	3.4168	1.2530157177217000	16932
**DENND2A**	0.10505	1.7644	1.2532905491152500	16936
**HTR7**	0.10505	2.446	1.2532905491152500	16933
**IP6K2**	0.10505	1.8093	1.2532905491152500	16935
**RAB6B**	0.10505	0.093843	1.2532905491152500	16938
**SMC5**	0.10505	1.9512	1.2532905491152500	16934
**SPDYE16**	0.10505	1.0539	1.2532905491152500	16937
**SYTL3**	0.10503	3.9802	1.2534005081828800	16939
**TRPV5**	0.10503	3.8674	1.2534005081828800	16940
**COL26A1**	0.10492	3.4317	1.2540055541329700	16941
**NOS2**	0.10492	2.481	1.2540055541329700	16942
**EEF1AKMT3**	0.10464	4.1317	1.25554774630279	16943
**LPCAT4**	0.1045	2.4717	1.2563199634076800	16944
**MTOR**	0.10449	4.5462	1.2563751504445800	16945
**MYO15A**	0.10449	3.5716	1.2563751504445800	16946
**TLE4**	0.10442	2.9892	1.2567615668893700	16947
**CCNJ**	0.10435	3.3101	1.257148171081980	16948
**ZMAT3**	0.10431	3.0314	1.257369172150890	16949
**BABAM2**	0.10426	4.072	1.2576455098765600	16952
**DBNDD1**	0.10426	2.165	1.2576455098765600	16954
**LRRC42**	0.10426	4.4414	1.2576455098765600	16950
**PAQR7**	0.10426	4.0535	1.2576455098765600	16953
**ZNF747**	0.10426	4.281	1.2576455098765600	16951
**PRAMEF27**	0.10412	3.3223	1.2584197668835800	16955
**TMEM225B**	0.10411	2.0316	1.2584750998276700	16956
**GPR155**	0.10399	2.8717	1.2591393959228100	16957

**HIVEP1**	0.10387	1.5374	1.259804248127720	16958
**IFI30**	0.10387	1.4784	1.259804248127720	16959
**TNFSF13B**	0.10387	0.50996	1.259804248127720	16960
**CBWD6**	0.10386	5.9423	1.2598596776187100	16961
**PLAGL1**	0.10371	3.7431	1.2606915849139200	16962
**PCDH10**	0.10344	3.5189	1.2621912206416200	16963
**MEX3A**	0.10341	4.6837	1.2623580221261500	16964
**CAPZA3**	0.10326	3.7925	1.2631925568825000	16965
**THSD4**	0.10326	3.6747	1.2631925568825000	16966
**FAM131C**	0.10322	1.544	1.263415248119930	16967
**HCFC1R1**	0.10316	1.3539	1.2637494025007700	16968
**EPPK1**	0.10315	5.9535	1.2638051086180900	16969
**FCRL3**	0.10314	4.2372	1.2638608186574800	16970
**ZNF804A**	0.10311	3.7774	1.2640279723154000	16971
**SLFN5**	0.10308	2.9462	1.2641951612981400	16972
**TWIST1**	0.10297	1.5018	1.2648084901088000	16973
**ANKMY1**	0.10287	1.2367	1.2653664749217600	16976
**PNMT**	0.10287	2.5101	1.2653664749217600	16974
**SLC1A7**	0.10287	2.2467	1.2653664749217600	16975
**ACADVL**	0.10286	3.9305	1.2654222950745500	16977
**CBS**	0.10286	2.1035	1.2654222950745500	16980
**FAAP100**	0.10286	3.8023	1.2654222950745500	16978
**LRP6**	0.10286	2.1381	1.2654222950745500	16979
**LRRC75B**	0.10282	1.4852	1.2656456151249400	16981
**XBP1**	0.10264	2.3613	1.2666513373603500	16982
**MKNK1**	0.1026	3.1323	1.2668750052499200	16983
**CHMP7**	0.10259	2.1797	1.2669309321261500	16984
**JMJD7**	0.10229	1.634	1.2686105845283000	16985
**HSD17B14**	0.10226	2.0713	1.2687787467054900	16986
**BTBD2**	0.10213	3.2001	1.2695078644234900	16987
**TUBA4A**	0.10209	2.593	1.2697323441623400	16988
**MAGOHB**	0.10205	3.3719	1.2699568879026800	16989

**IZUMO4**	0.10203	2.3634	1.2700691837883600	16990
**BAALC**	0.10191	3.5694	1.2707432956967400	16991
**GCH1**	0.10191	2.3801	1.2707432956967400	16992
**DDX3Y**	0.1017	4.0121	1.2719243830219100	16993
**PISD**	0.10167	5.9757	1.2720932546776600	16994
**SUPT20H**	0.1016	4.348	1.2724874296842100	16995
**AIPL1**	0.10151	3.6841	1.2729945168179700	16998
**CD72**	0.10151	1.6298	1.2729945168179700	17000
**CHI3L2**	0.10151	3.3193	1.2729945168179700	16999
**DYNLL2**	0.10151	3.8813	1.2729945168179700	16997
**ZNF157**	0.10151	4.0967	1.2729945168179700	16996
**GCSAM**	0.10132	4.281	1.2740661104187400	17001
**DDIT3**	0.10131	4.7789	1.274122550618590	17002
**UQCR10**	0.10126	3.8477	1.2744048125181700	17003
**DNLZ**	0.10107	0.22712	1.2754783348752000	17006
**PGPEP1L**	0.10107	0.25965	1.2754783348752000	17005
**TRMU**	0.10107	1.9923	1.2754783348752000	17004
**CEP170**	0.10103	4.3021	1.2757045269654000	17007
**MYH2**	0.10103	2.5779	1.2757045269654000	17008
**ZNF189**	0.10103	1.906	1.2757045269654000	17009
**CATSPER4**	0.10098	4.0889	1.275987358894500	17010
**CD276**	0.10087	3.9602	1.2766099486945000	17011
**TOX4**	0.10082	5.9867	1.2768931077088600	17012
**ACKR1**	0.10081	2.4114	1.2769497517986700	17013
**MARCH11**	0.10073	4.1772	1.277403052090730	17014
**NUAK1**	0.10019	1.8876	1.2804697185928200	17015
**ARNTL2**	0.10007	4.126	1.2811528374631100	17016
**CSNK1G2**	0.10007	1.7856	1.2811528374631100	17017
**LOC100130[illegible]**	0.099989	1.4877	1.2816142808858500	17018
**ALDH5A1**	0.099979	4.2823	1.2816712681372900	17019
**APBB3**	0.099979	1.5232	1.2816712681372900	17023
**CER1**	0.099979	1.1642	1.2816712681372900	17025

**IGLL1**	0.099979	1.7606	1.2816712681372900	17022
**MRPS18A**	0.099979	2.0417	1.2816712681372900	17021
**OR52R1**	0.099979	3.5376	1.2816712681372900	17020
**PTH2R**	0.099979	1.2671	1.2816712681372900	17024
**IFFO2**	0.099938	2.5214	1.2819049593966400	17028
**MAN1B1**	0.099938	2.8107	1.2819049593966400	17027
**PTPRU**	0.099938	3.3811	1.2819049593966400	17026
**ACBD7**	0.099923	3.6916	1.2819904736924900	17029
**STAU1**	0.099887	4.5375	1.2821957462610400	17030
**C12orf77**	0.099856	2.9477	1.2823725520536300	17031
**SYTL2**	0.099761	3.4742	1.282914626074390	17032
**CLCA4**	0.099739	3.2798	1.2830402127763100	17033
**SCP2**	0.099662	3.0385	1.2834799256740500	17034
**MT1E**	0.099599	4.3666	1.2838398754399000	17035
**CDH7**	0.0995	1.7805	1.2844058470918600	17036
**MYO3B**	0.099262	4.2725	1.2857681519736700	17037
**RAP2A**	0.099228	4.4768	1.2859629618378300	17038
**C8orf89**	0.099166	2.1291	1.2863183290480700	17040
**IFT52**	0.099166	0.10649	1.2863183290480700	17041
**TRIM64C**	0.099166	2.7446	1.2863183290480700	17039
**TUBG2**	0.099082	2.7834	1.2868000535881600	17042
**EPHX1**	0.098704	1.1093	1.2889715186293400	17043
**WASHC1**	0.098653	2.7535	1.2892649597285000	17044
**TRIM47**	0.09865	2.8293	1.2892822244271000	17045
**GPR150**	0.098566	1.4903	1.289765792096920	17047
**ZCCHC18**	0.098566	4.1632	1.289765792096920	17046
**GJC1**	0.098487	2.8481	1.2902208513416300	17048
**AZU1**	0.098419	4.283	1.2906127619818000	17050
**MARK1**	0.098419	4.7595	1.2906127619818000	17049
**OR13C9**	0.098419	2.1327	1.2906127619818000	17052
**OSTC**	0.098419	3.6246	1.2906127619818000	17051
**CEBPA**	0.098392	4.1565	1.2907684285534000	17053

**ADCK2**	0.098307	2.0264	1.2912586943294600	17054
**CSF1**	0.098307	1.8086	1.2912586943294600	17055
**C6orf229**	0.098171	3.8195	1.2920437657575400	17056
**UGT3A2**	0.098066	4.3444	1.2926504322704800	17057
**RGMB**	0.097818	1.8241	1.2940852128873400	17058
**CCDC87**	0.097682	4.6155	1.294873160365630	17059
**BRD9**	0.097589	1.5338	1.2954124405760200	17060
**TMEM268**	0.09741	4.2874	1.2964514717322500	17061
**SLX1A**	0.097352	3.0261	1.2967884414431300	17062
**ANO4**	0.097305	3.8753	1.2970616111518700	17063
**MAPK15**	0.097305	2.6803	1.2970616111518700	17064
**C9orf153**	0.097272	2.6952	1.2972534690120900	17065
**CELF2**	0.097272	1.9954	1.2972534690120900	17067
**MPLKIP**	0.097272	1.7091	1.2972534690120900	17068
**XIRP1**	0.097272	2.3759	1.2972534690120900	17066
**RALA**	0.097203	3.298	1.2976547807341900	17069
**HOMER1**	0.097172	1.4955	1.2978351482608500	17070
**EGFL6**	0.097143	4.0773	1.298003917404970	17071
**TRAPPC3L**	0.097068	4.1447	1.298440560865810	17072
**IL17RC**	0.096667	2.5117	1.3007793595130700	17077
**OR8U1**	0.096667	3.6812	1.3007793595130700	17075
**SAT1**	0.096667	4.2001	1.3007793595130700	17073
**TMPRSS15**	0.096667	3.8159	1.3007793595130700	17074
**VPS37D**	0.096667	3.5461	1.3007793595130700	17076
**ZNF844**	0.096667	1.9725	1.3007793595130700	17078
**GSTM3**	0.096652	3.4738	1.3008669838734500	17080
**MGA**	0.096652	3.6171	1.3008669838734500	17079
**KCNH8**	0.096622	4.6795	1.3010422625648500	17081
**EML4**	0.096576	1.6407	1.3013111008842300	17082
**HOXD12**	0.096404	3.2439	1.3023171567111200	17083
**DGUOK**	0.096355	3.1509	1.3026040070067700	17084
**CHCHD7**	0.096249	2.7588	1.3032249072973900	17085

**GOLGA8R**	0.096178	6.0508	1.3036410744692100	17086
**FLT1**	0.096122	3.181	1.303969478341500	17087
**FZD6**	0.096002	4.1499	1.304673674884520	17088
**OR2D2**	0.095979	4.4699	1.3048087197949600	17089
**DR1**	0.095822	2.4187	1.3057311843806900	17092
**OR2A12**	0.095822	2.5474	1.3057311843806900	17091
**WBP11**	0.095822	2.2547	1.3057311843806900	17093
**WBP2**	0.095822	3.1241	1.3057311843806900	17090
**TP53I3**	0.095675	2.3722	1.3065959016060800	17094
**IDH3A**	0.095635	4.829	1.3068313679747600	17095
**ANAPC7**	0.095578	4.2653	1.3071670327940000	17096
**PPIE**	0.095563	4.2832	1.307255390123690	17097
**CTXN2**	0.095145	2.5107	1.3097217307143100	17100
**GOLPH3L**	0.095145	2.8073	1.3097217307143100	17099
**RPAIN**	0.095145	3.5598	1.3097217307143100	17098
**SPPL2B**	0.095083	3.2251	1.3100882306295900	17101
**ISM1**	0.095053	4.4107	1.3102656324899400	17102
**MAB21L3**	0.095053	3.9526	1.3102656324899400	17103
**ZNF611**	0.095053	3.6674	1.3102656324899400	17104
**CNTD1**	0.094993	1.6938	1.3106205599719100	17107
**LRRC61**	0.094993	1.9188	1.3106205599719100	17106
**RABIF**	0.094993	2.1771	1.3106205599719100	17105
**OR5A2**	0.094905	1.585	1.3111414191274700	17108
**IQCK**	0.094875	1.8354	1.3113190660741100	17109
**FOXH1**	0.094421	1.5192	1.314012523951200	17112
**VGLL2**	0.094421	2.1266	1.314012523951200	17111
**WDR6**	0.094421	3.315	1.314012523951200	17110
**ZER1**	0.094409	6.0716	1.3140838460588500	17113
**GGH**	0.094219	2.9236	1.3152140047205600	17115
**SEC14L4**	0.094219	3.1934	1.3152140047205600	17114
**TNFSF4**	0.094174	4.1721	1.3154819200503500	17116
**RFPL4B**	0.094072	3.9005	1.3160895446320500	17117

**MAP4K2**	0.094052	1.4673	1.316208743700080	17118
**CCDC124**	0.093981	2.8337	1.316632051503130	17119
**RNF185**	0.093916	2.3003	1.3170197937855900	17120
**BNC2**	0.093852	4.2365	1.317401764359860	17121
**GRIK3**	0.093782	3.4819	1.3178197648804300	17122
**TMSB4Y**	0.093765	3.0686	1.3179213140490100	17123
**NPRL2**	0.093739	3.5975	1.3180766508351700	17124
**PRR25**	0.093739	3.2988	1.3180766508351700	17125
**TAS2R19**	0.093739	1.7644	1.3180766508351700	17127
**UBR4**	0.093739	0.6404	1.3180766508351700	17128
**ZNF281**	0.093739	2.0965	1.3180766508351700	17126
**KRT7**	0.093689	4.1744	1.3183754648482400	17129
**MYRFL**	0.093689	4.1093	1.3183754648482400	17130
**RBL1**	0.093689	3.8248	1.3183754648482400	17131
**RAF1**	0.093536	3.9579	1.3192905677641800	17132
**CD79B**	0.09348	3.5059	1.31962578373168	17133
**TRAPPC12**	0.093465	4.1098	1.3197155989064200	17134
**DNAH5**	0.093447	4.3286	1.3198233911705500	17136
**PSMB5**	0.093447	5.1366	1.3198233911705500	17135
**DERL1**	0.093427	2.1977	1.3199431783415200	17139
**ECM2**	0.093427	3.3168	1.3199431783415200	17138
**MYADML2**	0.093427	3.8655	1.3199431783415200	17137
**HIST1H3A**	0.093236	2.5806	1.3210881012006100	17140
**NANOS2**	0.093186	3.0627	1.321388105390880	17141
**MESP1**	0.09311	3.3057	1.3218443396620900	17142
**SLC17A1**	0.09282	6.092	1.3235877668149200	17143
**FRMD3**	0.09275	3.3174	1.3240091972923800	17144
**C16orf71**	0.092694	2.9949	1.324346511059820	17147
**SPNS3**	0.092694	3.1492	1.324346511059820	17146
**ZNF596**	0.092694	4.0913	1.324346511059820	17145
**FBXO22**	0.09268	2.0297	1.324430863049950	17148
**TPD52**	0.09268	0.67341	1.324430863049950	17151

**TRMT10A**	0.09268	0.91518	1.324430863049950	17149
**USF1**	0.09268	0.75507	1.324430863049950	17150
**SMYD5**	0.092665	4.1068	1.3245212506408600	17152
**NO-TARGET**	0.092568	0.096322	1.325106018488410	17153
**AVIL**	0.092535	3.9029	1.3253050634623300	17154
**CUL3**	0.09236	4.7582	1.3263614836063600	17155
**DCST1**	0.09234	4.1029	1.3264823116217000	17157
**JADE1**	0.09234	3.8599	1.3264823116217000	17160
**KRTAP13-1**	0.09234	4.0111	1.3264823116217000	17158
**OTUB2**	0.09234	1.4161	1.3264823116217000	17163
**PP2D1**	0.09234	1.8925	1.3264823116217000	17162
**SDHAF1**	0.09234	3.0266	1.3264823116217000	17161
**TNFSF11**	0.09234	4.4188	1.3264823116217000	17156
**TTLL3**	0.09234	3.9223	1.3264823116217000	17159
**RNF180**	0.092079	2.5597	1.3280608958376100	17164
**LUZP4**	0.091981	1.5288	1.3286544763806800	17165
**CMIP**	0.091843	2.3821	1.3294911292857000	17166
**HACL1**	0.09183	3.2902	1.3295699923896500	17168
**TAF1D**	0.09183	3.6734	1.3295699923896500	17167
**UPK1A**	0.09183	0.14498	1.3295699923896500	17169
**PLEKHG4**	0.091734	2.584	1.3301526222538900	17170
**FAM58A**	0.091705	3.4562	1.3303287138550800	17171
**MAGEB4**	0.091604	3.0617	1.3309423206470200	17172
**CD24**	0.0916	3.5924	1.3309666322237300	17173
**OSBPL7**	0.0916	1.3009	1.3309666322237300	17175
**PDCL**	0.0916	1.7801	1.3309666322237300	17174
**TMEM30A**	0.0916	0.70194	1.3309666322237300	17176
**FBXO32**	0.091547	4.0973	1.331288834909360	17177
**OR10P1**	0.091488	3.329	1.3316476760091600	17178
**IER3**	0.091438	6.1122	1.3319519129239400	17179
**RTTN**	0.091322	3.6053	1.332658217773510	17180
**SOX17**	0.091241	4.1861	1.3331518078693500	17181

**SNX29**	0.091194	4.2264	1.3334383609706600	17182
**KIF9**	0.090891	4.3406	1.3352883478921000	17183
**ANKRD13C**	0.090828	4.3186	1.3356735729743500	17185
**B3GALNT2**	0.090828	4.1845	1.3356735729743500	17186
**LCE2D**	0.090828	4.1409	1.3356735729743500	17187
**LRRC4**	0.090828	1.5575	1.3356735729743500	17191
**MZT2B**	0.090828	2.0862	1.3356735729743500	17190
**PPARGC1B**	0.090828	3.7292	1.3356735729743500	17189
**PTPRH**	0.090828	4.0797	1.3356735729743500	17188
**SLC35G2**	0.090828	4.4559	1.3356735729743500	17184
**RNF217**	0.090675	1.5604	1.336609945754340	17192
**PREX1**	0.090325	2.7066	1.3387563934216000	17193
**ROCK1**	0.090299	5.8294	1.3389160900832100	17194
**ABCB9**	0.090208	3.4906	1.33947529748198	17196
**HIST1H4B**	0.090208	3.8254	1.33947529748198	17195
**LRRCC1**	0.090208	2.6713	1.33947529748198	17197
**PHF11**	0.090208	0.79927	1.33947529748198	17198
**SIRPB2**	0.090208	0.21935	1.33947529748198	17200
**SULT1C4**	0.090208	0.62577	1.33947529748198	17199
**PSMB7**	0.090044	2.8884	1.3404841588367800	17201
**HIGD1A**	0.089964	3.3464	1.340976781797480	17202
**SNX3**	0.089931	3.6963	1.3411800836054200	17203
**RAB4A**	0.089902	4.9217	1.3413587885427800	17204
**KCNC2**	0.089848	6.1321	1.3416916636549400	17205
**PPP3CB**	0.089732	4.4224	1.3424072318504100	17206
**FAM111A**	0.089626	2.4338	1.3430617147021600	17207
**ZNF823**	0.089587	3.2512	1.3433026598270600	17208
**CDC23**	0.089545	4.3127	1.3435622264362200	17209
**CHMP1A**	0.089545	4.2799	1.3435622264362200	17211
**OSR2**	0.089545	4.041	1.3435622264362200	17212
**SOX13**	0.089545	4.2928	1.3435622264362200	17210
**SULT1A1**	0.089545	3.9402	1.3435622264362200	17213

**TSNAX**	0.089528	2.8263	1.3436673148509900	17214
**EPHB3**	0.089517	1.6277	1.3437353211450300	17215
**GPR19**	0.089514	4.1788	1.3437538693947300	17216
**AQP8**	0.089444	4.1423	1.3441867931956300	17217
**SUOX**	0.089422	1.7043	1.3443229070058000	17218
**C2orf88**	0.089267	4.1321	1.3452825973540200	17219
**LNP1**	0.089267	2.7217	1.3452825973540200	17220
**MAS1L**	0.089206	3.9789	1.3456606219101600	17221
**ABCB4**	0.089174	2.2971	1.3458590067960000	17222
**IRS1**	0.089168	2.7892	1.3458962098594500	17223
**KDM2A**	0.089114	0.6844	1.3462311212898200	17224
**OTUD1**	0.08901	4.2319	1.3468765616934900	17225
**FAM214B**	0.088944	6.1419	1.3472864593069900	17227
**GOLGA6L9**	0.088944	6.1419	1.3472864593069900	17226
**ACTN4**	0.088923	4.438	1.347416928757270	17228
**IBTK**	0.08855	3.4184	1.3497381466815600	17229
**RLN2**	0.088464	4.5757	1.3502743664556700	17230
**HCRTR2**	0.088415	2.2525	1.3505800607287700	17231
**AFAP1L1**	0.088406	4.1383	1.3506362223778800	17232
**CDK5**	0.088388	3.0467	1.3507485584581300	17235
**CKAP4**	0.088388	4.2225	1.3507485584581300	17233
**CLEC4C**	0.088388	1.8284	1.3507485584581300	17237
**FASTKD1**	0.088388	3.8262	1.3507485584581300	17234
**TMEM161A**	0.088388	1.7563	1.3507485584581300	17238
**ZNF493**	0.088388	2.6433	1.3507485584581300	17236
**ARL 6.00**	0.08838	3.1328	1.350798491076790	17239
**ZNF816-ZNI**	0.088364	2.737	1.3508983664190600	17240
**ASMT**	0.088345	4.2101	1.3510169858928700	17241
**C19orf67**	0.088345	3.3479	1.3510169858928700	17245
**CDC42EP2**	0.088345	3.8137	1.3510169858928700	17243
**DNAAF2**	0.088345	2.8166	1.3510169858928700	17246
**EIF3I**	0.088345	2.2001	1.3510169858928700	17247

**PCDHB9**	0.088345	3.5798	1.3510169858928700	17244
**PIK3CG**	0.088345	4.0028	1.3510169858928700	17242
**PROB1**	0.088345	2.049	1.3510169858928700	17248
**CYP3A5**	0.088161	3.5691	1.3521667066153200	17249
**PIK3CA**	0.088137	6.1517	1.3523168020136500	17250
**EGLN1**	0.088129	3.0169	1.3523668405838800	17251
**EFR3A**	0.088121	4.2863	1.3524168825404700	17252
**CASP10**	0.088004	4.4229	1.353149133444100	17253
**CLN3**	0.088004	1.4926	1.353149133444100	17256
**GP1BA**	0.088004	2.3597	1.353149133444100	17255
**SPIDR**	0.088004	3.2658	1.353149133444100	17254
**LEFTY1**	0.087967	2.5905	1.353380851035210	17257
**TMEM215**	0.087603	3.348	1.355664335396260	17258
**MTCL1**	0.087545	2.0584	1.3560288407943100	17259
**HSPA13**	0.087507	3.8036	1.356267752398980	17260
**DNMT3B**	0.087464	3.6638	1.3565381931380400	17261
**GALT**	0.087464	1.4126	1.3565381931380400	17262
**TMEM35A**	0.087464	0.02522	1.3565381931380400	17263
**SDC2**	0.087355	2.8164	1.357224173848410	17264
**LRRC34**	0.087325	4.2899	1.357413087999120	17265
**LUM**	0.087258	2.3602	1.357835171270990	17266
**ELL3**	0.08705	4.0878	1.3591470630047100	17267
**PCDHB6**	0.08705	2.1975	1.3591470630047100	17268
**PCM1**	0.08705	1.4267	1.3591470630047100	17271
**RNF133**	0.08705	1.5327	1.3591470630047100	17270
**WDR33**	0.08705	2.1783	1.3591470630047100	17269
**LARP1**	0.086978	6.1711	1.3596017248076500	17272
**MMP3**	0.08686	3.4276	1.3603474733203100	17273
**PDE6H**	0.086754	4.2864	1.3610180286515200	17274
**PIP4K2C**	0.086639	1.6889	1.3617462107239200	17275
**C15orf56**	0.086619	0.83707	1.3618729248142500	17280
**COPS5**	0.086619	3.0436	1.3618729248142500	17277

**CSF2RA**	0.086619	2.4389	1.3618729248142500	17278
**LRRC26**	0.086619	1.8649	1.3618729248142500	17279
**P2RY14**	0.086619	3.375	1.3618729248142500	17276
**WNK4**	0.086467	3.1547	1.3628366673229300	17281
**ARHGAP35**	0.086113	2.5969	1.3650860934859700	17282
**ELOC**	0.085831	3.9481	1.3668829646509600	17286
**FBP1**	0.085831	4.1202	1.3668829646509600	17285
**KCNJ11**	0.085831	4.1659	1.3668829646509600	17284
**UBE2S**	0.085831	4.3802	1.3668829646509600	17283
**CHD9**	0.085785	1.7154	1.3671764902629800	17289
**IFIT1B**	0.085785	0.3861	1.3671764902629800	17290
**MDFIC**	0.085785	3.491	1.3671764902629800	17287
**PLXDC2**	0.085785	2.1476	1.3671764902629800	17288
**E2F7**	0.085737	3.7397	1.3674829034960400	17291
**MYCBPAP**	0.085672	3.5917	1.3678980428460900	17292
**ADGRF4**	0.085457	4.1277	1.369272878404500	17293
**DTX1**	0.085454	1.1165	1.3692920804711500	17295
**MTX1**	0.085454	3.6862	1.3692920804711500	17294
**CDPF1**	0.085081	2.3799	1.3716834821696800	17297
**OR6M1**	0.085081	2.9307	1.3716834821696800	17296
**ZNF526**	0.085081	1.7631	1.3716834821696800	17298
**FNDC10**	0.084921	3.3242	1.3727116927335400	17299
**TSPY10**	0.08492	6.1996	1.3727181236141200	17300
**C4orf51**	0.084895	2.4851	1.3728789140824400	17301
**C5orf30**	0.084888	4.3957	1.3729239417746100	17302
**DDX46**	0.084743	3.262	1.3738572849630300	17303
**ALOX12**	0.084666	0.80165	1.374353409279280	17305
**DFFB**	0.084666	2.793	1.374353409279280	17304
**FAU**	0.084666	0.2253	1.374353409279280	17308
**GATS**	0.084666	0.42336	1.374353409279280	17307
**OR11H12**	0.084666	0.67003	1.374353409279280	17306
**ANGPTL5**	0.084647	4.3037	1.3744758816127300	17311

**CADM3**	0.084647	4.5532	1.3744758816127300	17309
**CENPV**	0.084647	1.8199	1.3744758816127300	17315
**CHIC1**	0.084647	3.8704	1.3744758816127300	17313
**GPR20**	0.084647	4.2685	1.3744758816127300	17312
**NBEAL2**	0.084647	1.5537	1.3744758816127300	17317
**TERF1**	0.084647	1.804	1.3744758816127300	17316
**UGT1A7**	0.084647	4.3312	1.3744758816127300	17310
**ZNF326**	0.084647	1.9181	1.3744758816127300	17314
**C2orf40**	0.084406	3.6452	1.376031138979450	17318
**HAPLN4**	0.084392	3.511	1.3761215882164400	17319
**RNF223**	0.084252	3.9113	1.3770267004463800	17320
**HMCN2**	0.084162	2.9545	1.377609154533420	17321
**NDUFB11**	0.084023	4.5935	1.378509641888730	17322
**FLG2**	0.083962	1.073	1.3789051729351500	17328
**KRTAP22-1**	0.083962	2.8498	1.3789051729351500	17324
**MRTO4**	0.083962	1.9931	1.3789051729351500	17325
**SERPING1**	0.083962	1.9689	1.3789051729351500	17326
**SPTBN1**	0.083962	3.1355	1.3789051729351500	17323
**STX3**	0.083962	1.122	1.3789051729351500	17327
**EPYC**	0.083944	4.2144	1.3790219282433300	17329
**CLDN17**	0.083857	4.3714	1.379586510754070	17330
**HIST1H2AL**	0.083791	2.685	1.3800151082494700	17332
**SLA**	0.083791	3.1924	1.3800151082494700	17331
**BHLHE22**	0.083679	3.9952	1.3807430058568900	17333
**SALL1**	0.083528	3.262	1.3817255273477400	17334
**APBB1IP**	0.083446	4.6317	1.382259641377610	17335
**APOL3**	0.083446	4.0605	1.382259641377610	17337
**GLYATL2**	0.083446	3.611	1.382259641377610	17341
**GPR142**	0.083446	1.2849	1.382259641377610	17345
**MFAP3**	0.083446	4.3195	1.382259641377610	17336
**MITF**	0.083446	1.7354	1.382259641377610	17343
**MRPL16**	0.083446	1.9674	1.382259641377610	17342

**MRPL3**	0.083446	3.8798	1.382259641377610	17339
**POLI**	0.083446	3.7092	1.382259641377610	17340
**SEPT6**	0.083446	1.6417	1.382259641377610	17344
**ZNF708**	0.083446	4.0191	1.382259641377610	17338
**CCDC40**	0.083428	4.7028	1.3823769387145000	17346
**C19orf57**	0.083263	4.1504	1.3834530516985700	17347
**SBK1**	0.083223	2.9037	1.383714168969940	17348
**ZDHHC19**	0.083179	1.2024	1.3840015069793000	17349
**MAP2K1**	0.083083	2.5616	1.3846288232181500	17350
**LIPE**	0.083022	3.4189	1.3850277136976300	17352
**SHISA7**	0.083022	3.9977	1.3850277136976300	17351
**MAGEC2**	0.082929	0.23163	1.3856362826483300	17354
**NAXE**	0.082929	2.5798	1.3856362826483300	17353
**CRISP3**	0.082899	5.1451	1.3858327047239	17355
**DPRX**	0.082899	4.6864	1.3858327047239	17356
**PDK2**	0.082652	2.0611	1.3874519494068300	17357
**NEU4**	0.082556	2.7799	1.388082274256830	17358
**ZNF267**	0.082538	4.792	1.388200521580530	17359
**GORASP2**	0.082503	2.9672	1.3884305025138400	17360
**MRC2**	0.082377	3.0611	1.3892590425408900	17361
**EXOC7**	0.082363	4.9607	1.3893511614312600	17362
**ATP12A**	0.082263	1.7689	1.3900094966446800	17365
**KPNA3**	0.082263	3.9253	1.3900094966446800	17364
**TTLL10**	0.082263	3.9761	1.3900094966446800	17363
**TAF4**	0.082221	4.2849	1.3902861771655800	17366
**ADAMTS13**	0.082182	3.0022	1.3905431901243	17367
**CALCOCO2**	0.082182	1.4799	1.3905431901243	17370
**DOC2B**	0.082182	0.52599	1.3905431901243	17371
**KIF13B**	0.082182	2.8467	1.3905431901243	17368
**TIMM9**	0.082182	2.4848	1.3905431901243	17369
**ZNF771**	0.082034	1.6763	1.3915193580300600	17372
**ZNF780B**	0.081928	3.0806	1.3922193210962100	17373

**HSD17B6**	0.081795	4.3596	1.3930985426473700	17374
**C3orf38**	0.081698	4.3573	1.393740459178800	17375
**PLGRKT**	0.081698	4.0582	1.393740459178800	17376
**HDAC2**	0.08167	3.5585	1.3939258615263400	17377
**RUNX3**	0.081469	2.8121	1.395258194322850	17378
**TAB3**	0.081408	1.4958	1.395663024385470	17379
**TARM1**	0.081311	4.0969	1.3963072419968600	17380
**CLNK**	0.081172	3.8376	1.397231410614390	17383
**CPLX4**	0.081172	4.2111	1.397231410614390	17382
**FSHB**	0.081172	4.2291	1.397231410614390	17381
**MACROD1**	0.081172	1.955	1.397231410614390	17386
**MED24**	0.081172	3.3536	1.397231410614390	17384
**ZNF558**	0.081172	2.1325	1.397231410614390	17385
**CCNB3**	0.081143	3.46	1.397424373296660	17387
**CTXN3**	0.081143	3.0735	1.397424373296660	17388
**MAP3K9**	0.081143	2.8806	1.397424373296660	17389
**SORL1**	0.081143	0.81999	1.397424373296660	17390
**PAIP2**	0.081129	3.1421	1.3975175463206600	17391
**TRAPPC1**	0.080992	4.4247	1.3984299518595300	17392
**C20orf27**	0.080511	2.863	1.4016426166437200	17393
**HTR1F**	0.080366	5.0807	1.4026139346532500	17394
**CHN2**	0.080294	6.2642	1.4030967363591500	17395
**KEL**	0.080243	1.0509	1.4034389188839000	17396
**BEND3**	0.080175	2.8063	1.4038954180272400	17398
**LAMB3**	0.080175	2.3537	1.4038954180272400	17400
**MYCBP**	0.080175	2.642	1.4038954180272400	17399
**RASD2**	0.080175	1.7273	1.4038954180272400	17401
**VCY1B**	0.080175	3.5196	1.4038954180272400	17397
**LPCAT2**	0.080146	4.5274	1.404090190504190	17402
**GRIN2B**	0.080107	4.8401	1.404352209918050	17403
**DNAJA2**	0.079995	3.5263	1.405105212450640	17404
**CCNG2**	0.079986	3.8798	1.4051657561742600	17409

**IL37**	0.079986	4.1979	1.4051657561742600	17407
**SAFB**	0.079986	4.0519	1.4051657561742600	17408
**TNFRSF10B**	0.079986	2.7748	1.4051657561742600	17410
**TPST2**	0.079986	4.5921	1.4051657561742600	17405
**ZBTB18**	0.079986	4.4965	1.4051657561742600	17406
**GPR32**	0.079956	3.9652	1.405367605795170	17411
**HOXA10**	0.079746	4.5193	1.4067821591410800	17412
**SELENOM**	0.079511	3.6486	1.4083684565432700	17413
**SLC45A4**	0.079459	4.2407	1.4087199462630400	17414
**FAM110C**	0.079336	0.20961	1.4095520480913500	17417
**PSPH**	0.079336	1.3572	1.4095520480913500	17416
**SKIL**	0.079336	0.12235	1.4095520480913500	17418
**SYCN**	0.079336	4.5342	1.4095520480913500	17415
**C19orf48**	0.079288	4.0199	1.4098770356557800	17420
**GAB3**	0.079288	4.8626	1.4098770356557800	17419
**SNAPC2**	0.07926	2.1821	1.410066680521610	17421
**FRG1**	0.079188	2.6819	1.4105545717592200	17422
**IFT46**	0.079108	4.7859	1.4110970672296800	17423
**CST9**	0.078901	1.7697	1.4125027051827900	17427
**MED18**	0.078901	4.1814	1.4125027051827900	17425
**STOX2**	0.078901	4.3495	1.4125027051827900	17424
**VSIG1**	0.078901	2.3816	1.4125027051827900	17426
**KIF20B**	0.078848	4.1979	1.4128630519221900	17428
**DDX17**	0.0787	4.4813	1.4138702758729400	17429
**LSM 3.00**	0.078573	6.291	1.4147357278721800	17430
**SFRP4**	0.07856	3.7434	1.4148243772507800	17431
**ATP6V0D2**	0.078557	2.334	1.41484483637874	17433
**HAUS6**	0.078557	3.4441	1.41484483637874	17432
**GUCY2C**	0.078465	3.572	1.415472537406270	17434
**TRPM6**	0.078465	2.8267	1.415472537406270	17435
**ZNF207**	0.078321	0.79732	1.41645614707037	17436
**TMEM42**	0.077939	4.5122	1.4190721066143000	17437

**CNKSR1**	0.077911	4.3557	1.4192642346542100	17438
**PABPC1L**	0.077911	1.9502	1.4192642346542100	17440
**XYLT1**	0.077911	4.2883	1.4192642346542100	17439
**PCDHA7**	0.077865	4.0476	1.419579987358010	17441
**TRIQK**	0.077843	3.2927	1.4197310495644800	17442
**RFC1**	0.07779	2.9328	1.420095105253990	17443
**ACTR3B**	0.077749	2.1458	1.4203768624113100	17448
**AMZ2**	0.077749	2.4077	1.4203768624113100	17446
**BMPR1B**	0.077749	2.919	1.4203768624113100	17445
**CHRNB3**	0.077749	2.3638	1.4203768624113100	17447
**GMEB1**	0.077749	3.9561	1.4203768624113100	17444
**ZZEF1**	0.077749	1.5985	1.4203768624113100	17449
**TMC3**	0.077574	2.8138	1.4215807541052800	17450
**CCL5**	0.077349	4.4459	1.4231316487742300	17451
**CIART**	0.077328	4.6867	1.4232765736884900	17452
**ARHGAP19**	0.077154	1.45	1.4244785317785800	17453
**KRT6C**	0.077106	1.4435	1.4248104688311	17454
**MAGEA12**	0.077071	3.7631	1.4250526052712200	17455
**TGIF1**	0.077005	4.2466	1.4255094328784000	17456
**LATS1**	0.076938	4.0008	1.4259734866037900	17457
**MYOM1**	0.076847	1.944	1.4266042607275000	17458
**C10orf25**	0.07669	4.3007	1.4276938557195400	17460
**CLEC5A**	0.07669	4.4846	1.4276938557195400	17459
**M6PR**	0.07669	3.2878	1.4276938557195400	17467
**OR2AT4**	0.07669	3.8603	1.4276938557195400	17466
**RIPPLY3**	0.07669	4.0984	1.4276938557195400	17463
**RTCB**	0.07669	3.8895	1.4276938557195400	17465
**THAP8**	0.07669	4.1856	1.4276938557195400	17462
**UBE2NL**	0.07669	3.9337	1.4276938557195400	17464
**UGT1A5**	0.07669	4.2809	1.4276938557195400	17461
**ZNF540**	0.076392	3.3862	1.429766678784220	17468
**ZNF331**	0.076359	3.307	1.4299965976932000	17469

**GLIS3**	0.076325	2.2779	1.4302335629327700	17472
**MIEN1**	0.076325	2.492	1.4302335629327700	17471
**TRPC7**	0.076325	0.69233	1.4302335629327700	17473
**ZDHHC16**	0.076325	3.3401	1.4302335629327700	17470
**WNT6**	0.076315	2.4611	1.4303032738777500	17474
**EIF2S3**	0.076281	2.5968	1.430540343097640	17475
**MAP3K4**	0.076256	4.0721	1.4307147100066800	17476
**MAP4K3**	0.076147	6.3259	1.4314754584902000	17477
**NDUFS6**	0.076147	6.3259	1.4314754584902000	17478
**GATD1**	0.076139	4.2669	1.4315313258826600	17479
**DGKD**	0.076116	4.3848	1.4316919695294900	17481
**SEC62**	0.076116	5.1545	1.4316919695294900	17480
**DPM2**	0.07599	2.3053	1.4325726736590100	17482
**SHISA5**	0.075876	3.0293	1.4333704597814200	17483
**METTL26**	0.075646	3.0721	1.4349828111565400	17486
**STX1A**	0.075646	3.6499	1.4349828111565400	17485
**TSC22D4**	0.075646	4.5882	1.4349828111565400	17484
**RND3**	0.075572	4.1867	1.4355023616962000	17487
**ASPRV1**	0.075523	2.4632	1.4358466017588100	17491
**CALCA**	0.075523	2.5447	1.4358466017588100	17490
**FAM57B**	0.075523	3.0936	1.4358466017588100	17489
**GPX2**	0.075523	4.1594	1.4358466017588100	17488
**CHRD**	0.075508	3.2778	1.4359520153927100	17492
**KIAA2012**	0.075373	4.1925	1.4369014569635500	17493
**PARP9**	0.075301	3.3416	1.4374083558704800	17494
**VLDLR**	0.07523	4.2429	1.437908576453720	17495
**CASP3**	0.075074	4.3779	1.4390089182191000	17496
**VKORC1**	0.074957	2.6962	1.4398353193543700	17497
**ZSCAN32**	0.074957	2.0321	1.4398353193543700	17498
**ATG9B**	0.074752	1.6608	1.4412856625852300	17502
**DDX49**	0.074752	3.5494	1.4412856625852300	17499
**EZH2**	0.074752	1.8978	1.4412856625852300	17501

**TPD52L2**	0.074752	3.276	1.4412856625852300	17500
**PIK3IP1**	0.074632	3.1533	1.4421360526792100	17503
**OR2T4**	0.074549	3.8364	1.4427248498516500	17504
**DNAJC17**	0.074499	4.2653	1.4430797884885600	17505
**DPP10**	0.074499	1.636	1.4430797884885600	17508
**LCE2B**	0.074499	2.303	1.4430797884885600	17506
**OR2W1**	0.074499	1.4211	1.4430797884885600	17509
**ZDHHC12**	0.074499	1.873	1.4430797884885600	17507
**DLEU7**	0.074429	2.9461	1.4435770082147200	17510
**FAM214A**	0.074401	2.062	1.4437759960710800	17511
**GDNF-AS1**	0.074074	4.1715	1.4461041343171700	17513
**PITRM1**	0.074074	5.1471	1.4461041343171700	17512
**MED6**	0.073922	3.1396	1.4471890005633500	17514
**C17orf99**	0.073819	4.0185	1.4479251094470000	17516
**MCIDAS**	0.073819	4.8668	1.4479251094470000	17515
**SMARCA1**	0.073819	3.8061	1.4479251094470000	17517
**CAGE1**	0.073715	2.2217	1.4486691619053000	17519
**DAAM1**	0.073715	2.8066	1.4486691619053000	17518
**PSME3**	0.073715	1.5879	1.4486691619053000	17521
**SLCO2B1**	0.073715	2.0501	1.4486691619053000	17520
**SH3GLB2**	0.07365	1.586	1.4491346022626000	17522
**ZNF248**	0.073566	3.1217	1.4497365597985700	17523
**MRPS16**	0.073359	4.1712	1.451222201796290	17524
**RNASE13**	0.07331	4.6699	1.4515743447237300	17525
**TIMM23B**	0.073168	3.3757	1.452595858552170	17526
**ELFN1**	0.073132	5.1614	1.4528550748778100	17527
**ZNF382**	0.073017	2.9665	1.4536837817357000	17528
**AKR1C4**	0.072996	1.2264	1.4538352186529000	17530
**DEPDC4**	0.072996	3.7047	1.4538352186529000	17529
**LCE1C**	0.072914	3.2274	1.4544268632692500	17531
**MUC3A**	0.07279	2.1124	1.455322513598430	17536
**ST8SIA5**	0.07279	3.6019	1.455322513598430	17534

**TICAM2**	0.07279	3.3101	1.455322513598430	17535
**TRIM15**	0.07279	3.7888	1.455322513598430	17532
**ZNF333**	0.07279	3.606	1.455322513598430	17533
**EFCAB2**	0.072731	4.3991	1.4557490799959400	17537
**RSPH10B2**	0.072685	3.1753	1.4560818410499800	17538
**HAUS5**	0.072493	3.0549	1.4574725007475600	17539
**KCNV2**	0.072493	2.6	1.4574725007475600	17541
**TMEM114**	0.072493	2.6078	1.4574725007475600	17540
**GPR62**	0.072262	1.9098	1.4591493826741400	17542
**DPY19L4**	0.072169	4.9285	1.4598256508786400	17543
**SPATA5L1**	0.072156	4.3886	1.4599202361939700	17544
**ALPK3**	0.072129	2.5488	1.4601167243623700	17545
**RARRES1**	0.071789	2.2484	1.462595858908270	17546
**ADRA1D**	0.071666	1.7375	1.4634949403397200	17550
**CRCP**	0.071666	3.9259	1.4634949403397200	17548
**ENC1**	0.071666	4.3464	1.4634949403397200	17547
**SPATA12**	0.071666	3.7994	1.4634949403397200	17549
**GALK2**	0.071582	6.3852	1.464109627660540	17551
**IK**	0.071582	6.3852	1.464109627660540	17552
**CCDC28A**	0.071544	1.3045	1.4643878823204600	17554
**CLEC4E**	0.071544	1.1483	1.4643878823204600	17556
**GPAA1**	0.071544	1.6027	1.4643878823204600	17553
**LRRC27**	0.071544	1.1822	1.4643878823204600	17555
**TNIP2**	0.071509	3.595	1.4646442698532900	17557
**BCL6**	0.071497	2.7622	1.4647321963176200	17558
**OR5T3**	0.071379	5.2778	1.4655974103497500	17559
**RBM28**	0.071294	2.927	1.4662213382130200	17560
**TRIP6**	0.071239	2.8747	1.4666253605922800	17561
**MKRN1**	0.07112	3.2251	1.4675003384075500	17562
**ARAP2**	0.070904	6.3934	1.4690914100332600	17563
**VPS45**	0.070904	6.3934	1.4690914100332600	17564
**KISS1R**	0.070802	3.7162	1.4698440438875000	17565

**MAP3K5**	0.070629	4.0705	1.4711224769013900	17566
**DACT3**	0.070604	3.3317	1.4713074205007300	17569
**GTF3C3**	0.070604	3.7016	1.4713074205007300	17568
**PRR14L**	0.070604	4.6255	1.4713074205007300	17567
**AADACL3**	0.070475	3.1117	1.4722625302373400	17571
**PDSS2**	0.070475	0.77503	1.4722625302373400	17572
**SLC4A8**	0.070475	3.7476	1.4722625302373400	17570
**TPRN**	0.070474	2.8412	1.472269939436130	17573
**AFAP1**	0.070432	0.17423	1.4725811987951400	17576
**CRIPT**	0.070432	4.6337	1.4725811987951400	17574
**GAL**	0.070432	3.2124	1.4725811987951400	17575
**NDST2**	0.070145	6.4017	1.474711966234550	17578
**NPDC1**	0.070145	6.4017	1.474711966234550	17577
**C1orf87**	0.07012	3.2243	1.4748978904201900	17579
**DIAPH2**	0.070087	2.7391	1.4751433884389500	17580
**KHDRBS2**	0.069971	2.8978	1.4760070574868700	17582
**SAMD15**	0.069971	3.3277	1.4760070574868700	17581
**SLC35F4**	0.069848	2.9669	1.4769240487588500	17583
**COL1A1**	0.069776	0.99336	1.4774614009094500	17585
**GEMIN7**	0.069776	1.2075	1.4774614009094500	17584
**KRTAP19-8**	0.069776	0.41998	1.4774614009094500	17586
**EID1**	0.069714	4.4512	1.477924462907700	17589
**EXOC3L2**	0.069714	3.6818	1.477924462907700	17590
**NMUR2**	0.069714	4.5979	1.477924462907700	17587
**POLG2**	0.069714	4.4831	1.477924462907700	17588
**C1orf94**	0.069655	4.7136	1.4783654131496200	17592
**CFHR4**	0.069655	5.765	1.4783654131496200	17591
**ZC3H12C**	0.069655	4.65	1.4783654131496200	17593
**UBR3**	0.069561	3.9882	1.4790685384277400	17594
**HIST1H3D**	0.069519	6.4098	1.4793829372709300	17595
**FKBP6**	0.069518	1.7526	1.4793904247401500	17596
**PDCL3**	0.069295	3.2072	1.4810622060363000	17597

**MMS19**	0.069266	3.8592	1.4812799169417100	17598
**BRMS1L**	0.069208	1.7201	1.4817155495054600	17601
**MEPCE**	0.069208	0.034322	1.4817155495054600	17602
**RASSF3**	0.069208	2.2062	1.4817155495054600	17600
**TKFC**	0.069208	2.2781	1.4817155495054600	17599
**RPS24**	0.069127	3.6872	1.4823244038809800	17603
**ADAM8**	0.069074	2.0938	1.4827230876766900	17604
**BHLHB9**	0.069056	2.1278	1.4828585433476200	17605
**MAPK10**	0.068949	2.4041	1.4836643142097600	17607
**OR10J1**	0.068949	4.3304	1.4836643142097600	17606
**CYP2A7**	0.068606	2.719	1.486253814503110	17608
**TOMM34**	0.068606	1.3761	1.486253814503110	17609
**ASCL1**	0.068587	4.3345	1.486397547784700	17610
**STAC2**	0.068587	3.2302	1.486397547784700	17611
**ERGIC1**	0.068043	2.2471	1.490525950879640	17613
**QSOX1**	0.068043	4.3263	1.490525950879640	17612
**TLR4**	0.068026	4.6014	1.4906553733455200	17614
**ANXA3**	0.067909	2.9201	1.4915467824599300	17617
**CPO**	0.067909	3.7833	1.4915467824599300	17615
**INSIG2**	0.067909	3.3086	1.4915467824599300	17616
**NAGLU**	0.067772	0.99709	1.492592077898960	17618
**ZNF23**	0.067772	0.37743	1.492592077898960	17619
**PPM1A**	0.067553	4.1473	1.4942664197227400	17620
**SPTAN1**	0.06741	2.5651	1.4953619761778800	17621
**SMTN**	0.067237	3.363	1.496689773357920	17622
**GTF2H5**	0.067196	2.6624	1.4970048406910800	17623
**GSTA4**	0.067134	2.8623	1.4974815664319800	17624
**AKAP13**	0.067024	3.3378	1.4983282086843800	17627
**CKS1B**	0.067024	4.2016	1.4983282086843800	17626
**OSBPL11**	0.067024	4.264	1.4983282086843800	17625
**PROM2**	0.067024	1.3999	1.4983282086843800	17629
**SYNGR4**	0.067024	1.7289	1.4983282086843800	17628

**PNMA8A**	0.066865	2.8554	1.4995538930611400	17630
**KRT28**	0.066843	4.8277	1.4997236621247900	17631
**SCN7A**	0.066843	2.375	1.4997236621247900	17632
**CLEC16A**	0.066758	5.0607	1.5003799945188500	17633
**ACP2**	0.066311	0.49783	1.5038422143558600	17636
**ASGR2**	0.066311	1.3454	1.5038422143558600	17635
**CKMT2**	0.066311	1.6361	1.5038422143558600	17634
**ZNF750**	0.066311	0.48148	1.5038422143558600	17637
**MCOLN2**	0.066267	4.7081	1.5041839904119800	17638
**RAP2B**	0.066267	2.7478	1.5041839904119800	17640
**ST18**	0.066267	4.4963	1.5041839904119800	17639
**GPR151**	0.066237	3.6821	1.5044171203195400	17641
**DUS2**	0.066207	4.4044	1.504650332020210	17642
**MED10**	0.066083	4.4674	1.50561514297236	17643
**ALG6**	0.065925	4.183	1.506846533111110	17645
**C11orf96**	0.065925	4.6822	1.506846533111110	17644
**CNTNAP5**	0.065925	4.0046	1.506846533111110	17646
**PER1**	0.065925	1.7368	1.506846533111110	17649
**POLR2E**	0.065925	3.417	1.506846533111110	17647
**RAPGEF2**	0.065925	2.256	1.506846533111110	17648
**REXO5**	0.065913	0.87687	1.506940149812700	17650
**FAM69B**	0.065871	2.9053	1.5072679123176200	17651
**ATAD5**	0.065719	4.5471	1.5084554559263300	17652
**FXYD5**	0.065719	1.4689	1.5084554559263300	17655
**IL9R**	0.065719	2.7088	1.5084554559263300	17654
**LRCH1**	0.065719	4.0696	1.5084554559263300	17653
**PLA2G6**	0.065648	4.8986	1.5090108934533900	17657
**RPS10-NUD**	0.065648	4.7947	1.5090108934533900	17658
**ZKSCAN3**	0.065648	5.8196	1.5090108934533900	17656
**LAYN**	0.065542	2.9921	1.509841005584600	17659
**GRK6**	0.065153	2.8616	1.512896310999850	17660
**CDKL3**	0.065141	2.7657	1.5129907868383900	17662

**GIF**	0.065141	4.21	1.5129907868383900	17661
**CCDC78**	0.064825	4.5549	1.5154835257427700	17664
**FAM159A**	0.064825	4.1391	1.5154835257427700	17666
**GPR101**	0.064825	1.8606	1.5154835257427700	17669
**HBEGF**	0.064825	4.0656	1.5154835257427700	17667
**PCED1A**	0.064825	4.1447	1.5154835257427700	17665
**RHOXF1**	0.064825	4.5975	1.5154835257427700	17663
**TMEM238**	0.064825	3.7406	1.5154835257427700	17668
**ANKK1**	0.064807	5.8936	1.5156258009286400	17670
**FAM172A**	0.064767	3.6247	1.5159420778962900	17671
**APBB2**	0.064508	3.6951	1.5179936506396500	17672
**ZNF791**	0.064431	3.4652	1.5186048114185600	17673
**ACP6**	0.064421	4.0375	1.518684224575970	17675
**CDH4**	0.064421	1.768	1.518684224575970	17677
**DPT**	0.064421	0.34445	1.518684224575970	17679
**LRPAP1**	0.064421	1.038	1.518684224575970	17678
**RASGRP1**	0.064421	3.9119	1.518684224575970	17676
**S100A1**	0.064421	4.0892	1.518684224575970	17674
**MT1A**	0.064391	3.3682	1.5189225215314100	17680
**NSUN2**	0.06429	0.51882	1.5197254226241900	17681
**CHRND**	0.064217	2.8039	1.5203063477951500	17682
**ERVFRD-1**	0.06419	5.8808	1.5205213405607200	17683
**RNF25**	0.06419	3.097	1.5205213405607200	17684
**IGFLR1**	0.063777	2.0136	1.5238187294592500	17691
**IZUMO2**	0.063777	3.782	1.5238187294592500	17689
**MUC13**	0.063777	4.4317	1.5238187294592500	17685
**PRELP**	0.063777	1.8116	1.5238187294592500	17692
**PSMB3**	0.063777	3.7176	1.5238187294592500	17690
**SFN**	0.063777	4.2609	1.5238187294592500	17687
**TAGAP**	0.063777	1.6925	1.5238187294592500	17693
**TSHZ1**	0.063777	4.3689	1.5238187294592500	17686
**YIPF6**	0.063777	3.9409	1.5238187294592500	17688

**PPFIA3**	0.063732	2.5638	1.5241790114920300	17694
**PMS1**	0.063632	6.4968	1.5249803473344000	17695
**COX8A**	0.063586	2.7598	1.5253492908802100	17696
**CAMKV**	0.063472	2.1405	1.526264525228500	17700
**CCDC57**	0.063472	2.6874	1.526264525228500	17698
**CCNC**	0.063472	2.6565	1.526264525228500	17699
**IFNL3**	0.063472	3.59	1.526264525228500	17697
**PLXNB3**	0.063472	0.061923	1.526264525228500	17701
**ZNF578**	0.063432	4.503	1.526585963283510	17702
**MRPL13**	0.063404	4.491	1.5268110638066100	17703
**PPIL2**	0.063265	5.4982	1.5279296740890200	17704
**PTPRB**	0.063045	4.0562	1.529704053941850	17705
**FAM92A**	0.063011	4.9622	1.5299787063243300	17706
**DEFB104A**	0.062919	2.0152	1.5307224625977600	17707
**OTOS**	0.062855	4.8293	1.5312403581047100	17708
**COL4A2-AS**	0.062853	4.183	1.5312565489582100	17710
**IGF2R**	0.062853	4.1521	1.5312565489582100	17711
**MEIKIN**	0.062853	4.3888	1.5312565489582100	17709
**SERINC5**	0.062853	4.1149	1.5312565489582100	17712
**XIAP**	0.062835	3.0521	1.531402284706470	17713
**CCDC65**	0.062742	2.0035	1.5321557713914700	17714
**CD44**	0.062678	3.5381	1.5326748055863100	17715
**FANCI**	0.06267	2.5134	1.532739713899260	17717
**FIBIN**	0.06267	1.239	1.532739713899260	17718
**MIIP**	0.06267	4.4757	1.532739713899260	17716
**RFXANK**	0.06267	0.15341	1.532739713899260	17719
**LIN28B**	0.062634	5.4486	1.533031881250440	17720
**ECM1**	0.062295	3.659	1.5357895644966500	17721
**TOGARAM1**	0.062265	3.6625	1.5360341704109100	17722
**SLCO2A1**	0.062255	0.81486	1.536115726142340	17723
**AKR1C3**	0.062055	0.14058	1.5377489907938600	17724
**PEBP4**	0.061975	3.4981	1.5384034467113200	17725

**AP3D1**	0.061842	3.8055	1.5394929402506100	17727
**KRIT1**	0.061842	3.8062	1.5394929402506100	17726
**LRFN4**	0.061842	3.6973	1.5394929402506100	17728
**POLR3H**	0.061842	2.5889	1.5394929402506100	17730
**RPS5**	0.061842	3.4132	1.5394929402506100	17729
**ZNF483**	0.061842	2.4396	1.5394929402506100	17731
**TBX5**	0.061788	1.5989	1.5399358130566100	17732
**SOWAHA**	0.061597	3.1758	1.5415046991223400	17733
**GPR15**	0.061537	3.8474	1.541998326929240	17734
**THEM5**	0.061537	3.6037	1.541998326929240	17735
**ELP4**	0.061492	6.5347	1.5423687945113300	17736
**ICMT**	0.061404	2.0277	1.5430938768110600	17737
**ZPR1**	0.061278	3.789	1.5441334773602600	17738
**C9orf116**	0.061078	3.768	1.5457870719365900	17739
**ACPP**	0.060959	4.7708	1.546772969820780	17740
**PRSS21**	0.060959	4.1701	1.546772969820780	17742
**PURB**	0.060959	3.1579	1.546772969820780	17743
**SDR16C5**	0.060959	4.7117	1.546772969820780	17741
**AZIN1**	0.060921	4.3735	1.5470881112746900	17744
**RPRD1A**	0.060529	3.03	1.550348049297350	17746
**SEMA4D**	0.060529	2.0909	1.550348049297350	17748
**SETBP1**	0.060529	2.4023	1.550348049297350	17747
**SPATA2**	0.060529	4.7997	1.550348049297350	17745
**RCVRN**	0.060468	5.4853	1.550856819374920	17749
**TUBB8**	0.060468	5.2853	1.550856819374920	17750
**PITX1**	0.060317	3.8074	1.5521179636007800	17752
**ZSCAN26**	0.060317	4.3176	1.5521179636007800	17751
**DDX52**	0.060204	2.916	1.5530633512350800	17753
**ATP6V0E1**	0.060141	1.7008	1.5535910289914700	17755
**PSMD13**	0.060141	4.7212	1.5535910289914700	17754
**TMEM234**	0.060021	5.4504	1.5545973279065700	17756
**FOXR1**	0.059887	3.1508	1.5557228920021600	17757

**MCF 2.00**	0.059834	2.0312	1.5561686222305900	17758
**ACAD9**	0.059834	3.3416	1.5561686222305900	17760
**LMAN1L**	0.059834	1.4596	1.5561686222305900	17759
**DYNC1H1**	0.059781	3.7617	1.556614661846200	17761
**PLCXD1**	0.05974	4.3511	1.5569599239315400	17762
**LILRB3**	0.05973	2.4653	1.5570441623566900	17763
**SSX7**	0.059706	3.2963	1.5572463796693900	17764
**SLC5A5**	0.05965	3.2604	1.5577184678667400	17765
**CCDC59**	0.059607	5.0293	1.558081199933450	17768
**KLHL40**	0.059607	5.2106	1.558081199933450	17767
**NHLRC2**	0.059607	5.7303	1.558081199933450	17766
**CAPN6**	0.05949	3.3758	1.5590692077501300	17769
**ZNF529**	0.059408	6.5643	1.55976256582794	17770
**STAT6**	0.059386	3.6679	1.5599487163606300	17771
**SOX21**	0.059295	4.4271	1.5607192774695100	17772
**ZPLD1**	0.059295	1.9936	1.5607192774695100	17773
**FAM212A**	0.059047	0.45261	1.5628239858419200	17775
**PRSS2**	0.059047	3.5709	1.5628239858419200	17774
**C17orf105**	0.059034	0.7309	1.562934504407970	17781
**GATA5**	0.059034	1.2645	1.562934504407970	17779
**KCNS3**	0.059034	2.9919	1.562934504407970	17777
**SLC16A1**	0.059034	0.89733	1.562934504407970	17780
**TIMP3**	0.059034	3.2816	1.562934504407970	17776
**ZNF449**	0.059034	1.5716	1.562934504407970	17778
**MARCH4**	0.059023	4.201	1.56302803503068	17782
**SIKE1**	0.058979	3.7474	1.5634022943230900	17783
**ANKRD61**	0.058909	4.2941	1.5639981585424800	17784
**KLHL5**	0.058799	5.8404	1.5649356400704400	17785
**LATS2**	0.058799	5.4004	1.5649356400704400	17786
**UCK2**	0.058799	5.2033	1.5649356400704400	17787
**ZNF324B**	0.058782	2.9537	1.5650806463413300	17788
**CALM2**	0.058737	3.757	1.5654646454037500	17789

**XK**	0.058671	2.9161	1.566028261930830	17790
**KCTD4**	0.058661	3.2056	1.5661137017838700	17791
**TMEM225**	0.058545	0.2873	1.567105640636710	17792
**C14orf178**	0.058449	3.7497	1.5679277231963100	17793
**C1orf109**	0.058313	4.2115	1.5690941571738300	17795
**KLF8**	0.058313	3.7778	1.5690941571738300	17796
**SLC35F2**	0.058313	3.2984	1.5690941571738300	17797
**SSUH2**	0.058313	4.6149	1.5690941571738300	17794
**C11orf68**	0.058223	3.5724	1.5698672374298200	17798
**METTL6**	0.058085	4.3048	1.5710544525828700	17799
**MS4A6A**	0.058085	2.3887	1.5710544525828700	17800
**TNFSF18**	0.058085	2.1966	1.5710544525828700	17801
**GOLGA6L6**	0.057986	5.7528	1.571907516479210	17802
**STEAP2**	0.057683	3.3261	1.5745255425739400	17803
**GABRP**	0.057603	6.5933	1.5752185744610200	17804
**ADRB3**	0.057512	3.2307	1.5760078191096700	17805
**ARPC1B**	0.057307	4.1266	1.577789393598380	17806
**MAGEH1**	0.05727	3.503	1.57811148008576	17807
**MEPE**	0.05727	3.4905	1.57811148008576	17808
**CDC45**	0.057243	4.2245	1.5783466195343900	17812
**DENND4B**	0.057243	4.5678	1.5783466195343900	17810
**GABBR1**	0.057243	2.3399	1.5783466195343900	17814
**IGF1R**	0.057243	4.4815	1.5783466195343900	17811
**KLK15**	0.057243	4.6308	1.5783466195343900	17809
**PARPBP**	0.057243	3.323	1.5783466195343900	17813
**ACTL7B**	0.057146	3.5587	1.5791921005270600	17815
**CDH10**	0.057146	1.673	1.5791921005270600	17817
**INTS12**	0.057146	0.91799	1.5791921005270600	17818
**KNL1**	0.057146	3.329	1.5791921005270600	17816
**ADGRF5**	0.056973	4.4879	1.5807028284099200	17819
**ACOT1**	0.056936	4.4823	1.5810264007737100	17820
**ARFGAP2**	0.056772	2.0902	1.58246261015486	17821

**NDUFS4**	0.056708	4.0735	1.5830239685952000	17822
**TXNRD1**	0.056673	3.5988	1.583331172611400	17823
**GP1BB**	0.056558	5.1687	1.5843416107651900	17824
**MAGEB18**	0.056558	5.08	1.5843416107651900	17825
**BSCL2**	0.056452	3.3271	1.585274405956790	17826
**NPR 3.00**	0.056355	1.3623	1.586129211882720	17829
**FANCD2OS**	0.056355	1.4728	1.586129211882720	17828
**HMSD**	0.056355	4.3118	1.586129211882720	17830
**PCDH7**	0.056355	4.5134	1.586129211882720	17827
**BIVM-ERCC**	0.056293	6.6146	1.5866761904075100	17831
**HDDC2**	0.056287	3.6185	1.5867291490119800	17832
**ZZZ3**	0.056287	1.696	1.5867291490119800	17833
**CDC5L**	0.056245	4.3432	1.5870999839085600	17834
**ARHGAP23**	0.055935	4.6137	1.5898438735367500	17835
**VSNL1**	0.055907	2.4932	1.5900922988315300	17836
**LGALS2**	0.0559	5.6173	1.5901544204908900	17838
**SYK**	0.0559	5.7495	1.5901544204908900	17837
**CRYGS**	0.055709	4.3849	1.591851827584930	17840
**OR1M1**	0.055709	3.6351	1.591851827584930	17843
**RAB42**	0.055709	4.4821	1.591851827584930	17839
**ROBO1**	0.055709	4.1037	1.591851827584930	17841
**SP5**	0.055709	3.6821	1.591851827584930	17842
**ARSI**	0.055672	2.5104	1.5921811754685000	17844
**CD6**	0.055664	3.3423	1.5922524085337800	17845
**CYTH1**	0.055556	4.0902	1.5932148466652300	17846
**NDRG3**	0.055556	3.659	1.5932148466652300	17847
**CHRNA10**	0.055541	5.4725	1.5933486354022400	17848
**KLHL11**	0.055526	3.6406	1.5934824526653700	17849
**GOT2**	0.055288	3.4064	1.5956095152616300	17850
**NACC1**	0.055205	1.7306	1.5963530065725800	17853
**NCKAP5L**	0.055205	1.7906	1.5963530065725800	17852
**SMIM14**	0.055205	2.0206	1.5963530065725800	17851

**SERPINI2**	0.055181	5.5821	1.5965681565676100	17854
**SLC25A10**	0.055181	5.4977	1.5965681565676100	17855
**NOL9**	0.055147	4.1289	1.596873078961780	17856
**C17orf75**	0.055035	4.4556	1.597878580754170	17857
**EMP2**	0.055035	3.7611	1.597878580754170	17858
**C11orf24**	0.054993	4.0424	1.5982560607609600	17861
**IGIP**	0.054993	4.9599	1.5982560607609600	17859
**OSGEPL1**	0.054993	4.5239	1.5982560607609600	17860
**SYT17**	0.054977	3.6449	1.5983999225903400	17862
**COIL**	0.054933	3.3432	1.5987957132882900	17863
**CHD6**	0.054918	4.3048	1.5989306991968100	17864
**CTDSPL2**	0.054727	2.4902	1.600652072857330	17865
**PNRC2**	0.054659	0.62392	1.6012660643465000	17866
**C19orf33**	0.054618	6.6357	1.6016365570032300	17868
**CYTH4**	0.054618	6.6357	1.6016365570032300	17867
**PDZD7**	0.054562	5.0046	1.6021429512364900	17869
**EIF4E3**	0.054472	5.2829	1.6029576608813800	17870
**NR3C2**	0.054472	4.1669	1.6029576608813800	17871
**PABPC3**	0.054472	2.1627	1.6029576608813800	17873
**TTC4**	0.054472	3.7271	1.6029576608813800	17872
**RHEBL1**	0.054363	4.2126	1.6039457915601800	17874
**ICAM3**	0.05432	1.6389	1.604336035706290	17876
**IRGQ**	0.05432	2.2061	1.604336035706290	17875
**BMP5**	0.054102	3.9283	1.6063182520086700	17881
**EIF4A2**	0.054102	4.195	1.6063182520086700	17879
**KRR1**	0.054102	4.1579	1.6063182520086700	17880
**LPXN**	0.054102	4.2031	1.6063182520086700	17878
**SERPINB11**	0.054102	4.9071	1.6063182520086700	17877
**UFC1**	0.054102	3.6751	1.6063182520086700	17882
**ZBBX**	0.054102	1.5835	1.6063182520086700	17883
**PARD6B**	0.054055	3.5349	1.6067464386150300	17884
**DTX4**	0.053862	4.0455	1.6085078327684600	17886

**GTF2E2**	0.053862	3.3704	1.6085078327684600	17887
**KIF25**	0.053862	4.3138	1.6085078327684600	17885
**C6orf89**	0.05384	1.973	1.6087089305637000	17890
**DEDD2**	0.05384	3.0714	1.6087089305637000	17888
**TBX1**	0.05384	2.6106	1.6087089305637000	17889
**ASPH**	0.053808	3.5364	1.6090015526331800	17893
**ATOX1**	0.053808	5.5395	1.6090015526331800	17891
**DEFB106B**	0.053808	3.7982	1.6090015526331800	17892
**RBMY1B**	0.053621	3.3198	1.6107143241944900	17894
**PCCB**	0.053515	4.5609	1.6116873022049900	17895
**SEPT5**	0.053385	4.3973	1.612882664040730	17896
**SPATA21**	0.053343	2.9654	1.6132693508677400	17897
**CARD18**	0.053305	4.1989	1.6136194183577400	17900
**EXOC5**	0.053305	2.3696	1.6136194183577400	17902
**FAM129A**	0.053305	4.3629	1.6136194183577400	17899
**MYH13**	0.053305	4.0104	1.6136194183577400	17901
**SCGB1D2**	0.053305	2.1766	1.6136194183577400	17903
**ZACN**	0.053305	4.3889	1.6136194183577400	17898
**PRAC2**	0.053304	3.5674	1.613628633330830	17904
**TYW3**	0.053128	3.9805	1.615252607311840	17905
**ACAN**	0.053078	3.091	1.615714741437810	17906
**DAB2**	0.052876	4.5105	1.6175852853286500	17907
**CNST**	0.052776	2.3875	1.618513395445100	17909
**CRTAC1**	0.052776	0.2398	1.618513395445100	17910
**SUN5**	0.052776	3.7833	1.618513395445100	17908
**LOX**	0.052636	4.3324	1.6198150964586800	17911
**H1FOO**	0.052613	3.5469	1.6200292100187400	17913
**KRT81**	0.052613	4.1559	1.6200292100187400	17912
**SDCCAG8**	0.052613	2.4415	1.6200292100187400	17914
**EIF2B4**	0.052461	6.6701	1.6214460921816100	17915
**EXOSC8**	0.05246	3.0534	1.6214554245581800	17916
**RGS20**	0.052318	2.8879	1.6227820582703700	17917

**STRN4**	0.052278	4.194	1.623156273954850	17918
**MTFMT**	0.052136	2.7447	1.624486578765180	17919
**PNCK**	0.052024	3.1669	1.6255378648993000	17920
**ARFGEF2**	0.051909	2.4981	1.6266191831289200	17923
**ASTN1**	0.051909	4.0609	1.6266191831289200	17921
**CALCB**	0.051909	3.6881	1.6266191831289200	17922
**NR1H4**	0.051909	1.9688	1.6266191831289200	17924
**NMRAL1**	0.051852	4.3593	1.6271558465753400	17925
**MRPL46**	0.051824	5.4417	1.6274196424587200	17926
**SCUBE3**	0.051824	5.1861	1.6274196424587200	17927
**CYBRD1**	0.05177	1.87	1.6279287117147600	17932
**GNPTG**	0.05177	4.6111	1.6279287117147600	17928
**MIR1-1HG**	0.05177	3.5314	1.6279287117147600	17930
**RBM4B**	0.05177	2.5176	1.6279287117147600	17931
**ZYG11A**	0.05177	3.9707	1.6279287117147600	17929
**GPX3**	0.051585	2.804	1.6296759515087800	17933
**C5orf24**	0.051458	5.0729	1.6308782941870400	17935
**HIST1H2BK**	0.051458	3.7819	1.6308782941870400	17937
**MGME1**	0.051458	4.7245	1.6308782941870400	17936
**THSD7A**	0.051458	5.8663	1.6308782941870400	17934
**AARSD1**	0.051208	2.7584	1.6332520229091500	17938
**FBXO33**	0.051129	5.3791	1.6340040380929500	17940
**KIAA1161**	0.051129	5.4583	1.6340040380929500	17939
**KRTAP6-3**	0.051129	5.1717	1.6340040380929500	17941
**FGFBP1**	0.051088	3.6636	1.6343946889713600	17944
**POLG**	0.051088	4.4154	1.6343946889713600	17942
**TMEM184C**	0.051088	3.7325	1.6343946889713600	17943
**FN3K**	0.051	4.6052	1.63523400236505	17945
**FAM35A**	0.050945	3.141	1.6357591587688200	17946
**LEPROTL1**	0.050803	4.4127	1.637117107076290	17947
**RPA1**	0.050731	2.7218	1.637806799228800	17949
**ZNF518A**	0.050731	3.2436	1.637806799228800	17948

**MRPL17**	0.050691	3.3957	1.6381902984153100	17950
**ARRDC1**	0.050637	4.3577	1.638708404801340	17951
**MMD2**	0.050612	3.9356	1.6389484179084800	17952
**PHLDA3**	0.050486	3.5962	1.640159523397320	17953
**FAHD2B**	0.050371	4.2679	1.6412670012238200	17954
**KRTAP9-9**	0.050348	3.1777	1.6414887385138000	17956
**TTF2**	0.050348	4.8734	1.6414887385138000	17955
**CCDC58**	0.050293	2.2442	1.6420193073653500	17961
**FAM170B**	0.050293	2.272	1.6420193073653500	17960
**GON4L**	0.050293	1.7596	1.6420193073653500	17963
**ITGB4**	0.050293	1.6146	1.6420193073653500	17964
**JAGN1**	0.050293	2.1975	1.6420193073653500	17962
**LDHD**	0.050293	3.8499	1.6420193073653500	17959
**OR13G1**	0.050293	4.9731	1.6420193073653500	17957
**RPL39**	0.050293	4.4616	1.6420193073653500	17958
**RARA**	0.050007	0.13291	1.6447857447677800	17965
**CECR6**	0.049943	2.5387	1.645406534324350	17968
**NSUN5**	0.049943	3.0645	1.645406534324350	17967
**SERINC2**	0.049943	3.9823	1.645406534324350	17966
**RINL**	0.049933	4.7663	1.645503589992210	17969
**LDLRAD2**	0.049903	4.0952	1.6457948500360600	17970
**STIM2**	0.049903	0.20499	1.6457948500360600	17971
**TRIP13**	0.049676	5.5188	1.6480032562231500	17972
**MAGEE1**	0.049667	3.5231	1.6480909799725900	17973
**C2orf54**	0.04955	3.3395	1.6492325446021100	17976
**CITED1**	0.04955	4.6681	1.6492325446021100	17974
**TMX2**	0.04955	4.4443	1.6492325446021100	17975
**POR**	0.049489	4.6716	1.6498285726992400	17977
**UNG**	0.049489	4.4889	1.6498285726992400	17978
**GTF2A1**	0.049342	4.7378	1.6512673157568700	17979
**UBR1**	0.049342	3.8522	1.6512673157568700	17980
**MGST3**	0.049328	3.6633	1.651404517294410	17981

**RGPD4**	0.049032	2.3502	1.6543126545481400	17982
**MMP27**	0.049008	5.261	1.6545490634640800	17984
**SAMSN1**	0.049008	5.6451	1.6545490634640800	17983
**DYNAP**	0.048996	0.13289	1.654667302607050	17986
**RPLP2**	0.048996	2.628	1.654667302607050	17985
**ARL6IP5**	0.048767	3.2592	1.6569281458192500	17989
**LRRC8C**	0.048767	4.8953	1.6569281458192500	17987
**SRRD**	0.048767	4.2472	1.6569281458192500	17988
**MORC2**	0.048714	2.8537	1.657452606419630	17990
**AGAP9**	0.048539	5.0518	1.659187561752510	17991
**FCER1G**	0.048351	1.8735	1.6610569810550400	17992
**PYDC2**	0.048351	1.0159	1.6610569810550400	17993
**BDKRB1**	0.048348	5.2048	1.6610868593021400	17994
**GSTP1**	0.048121	2.3096	1.6633519606182600	17995
**ESD**	0.048109	3.9734	1.66347193938113	17996
**DNAJB9**	0.04802	4.4841	1.6643625301593300	17997
**VAV1**	0.048012	1.5427	1.6644426479532400	17998
**IRF6**	0.048007	1.5659	1.664492727000320	18002
**MSL3**	0.048007	4.59	1.664492727000320	18000
**THSD1**	0.048007	4.6616	1.664492727000320	17999
**USH1G**	0.048007	4.343	1.664492727000320	18001
**SPRR2E**	0.047685	4.5944	1.6677266458961400	18003
**PMFBP1**	0.047683	5.1223	1.6677467869156000	18004
**PRAMEF14**	0.047681	3.5194	1.6677669286116200	18005
**BOLA1**	0.04752	3.8379	1.6693905593687200	18006
**KLF17**	0.047501	4.6316	1.6695824585287300	18007
**SLC26A10**	0.047466	4.4209	1.6699361180125900	18008
**TSPAN9**	0.047466	0.59608	1.6699361180125900	18009
**PCDHB4**	0.047392	5.8494	1.6706845436124600	18010
**DONSON**	0.047351	4.5542	1.6710996150617800	18011
**BMP15**	0.047291	4.7399	1.6717075561336600	18012
**SLC12A1**	0.0472	2.2504	1.67263078093103	18013

**USP17L19**	0.047109	5.3069	1.6735554335911800	18014
**MXI1**	0.047039	3.753	1.6742676797664200	18015
**TXLNG**	0.046941	2.5716	1.675266253555490	18016
**SYN1**	0.046932	3.6202	1.6753580431025000	18017
**CUBN**	0.04688	1.9159	1.6758886592933000	18019
**GPR182**	0.04688	3.3602	1.6758886592933000	18018
**OR4A5**	0.04688	1.64	1.6758886592933000	18020
**NEMP1**	0.046847	3.7785	1.6762256413852000	18021
**PRMT1**	0.046761	4.1017	1.6771047326880900	18022
**ENDOG**	0.046607	3.6003	1.6786821656216300	18023
**DCDC1**	0.046575	4.3722	1.679010468576460	18024
**SEPT8**	0.046511	4.0012	1.6796676179265600	18025
**ZBTB1**	0.046511	3.5855	1.6796676179265600	18026
**GTSF1**	0.046498	6.1943	1.679801190058020	18027
**PRR23C**	0.046498	6.0987	1.679801190058020	18028
**RPL30**	0.046498	5.797	1.679801190058020	18029
**CD1E**	0.046463	3.2304	1.6801609563772400	18030
**FLNA**	0.046463	2.6691	1.6801609563772400	18031
**DPPA3**	0.0463	4.2237	1.6818393109378700	18032
**NKX6-1**	0.046274	1.8621	1.6821074624656400	18035
**OLR1**	0.046274	0.25697	1.6821074624656400	18036
**PSMD12**	0.046274	2.559	1.6821074624656400	18033
**SPRY1**	0.046274	1.9983	1.6821074624656400	18034
**XRCC4**	0.045998	6.756	1.6849614797527100	18037
**RSRC1**	0.045856	5.3341	1.6864352140614900	18038
**PRCD**	0.045694	1.146	1.6881210018763600	18039
**ATP5S**	0.045608	4.7428	1.6890178791810800	18040
**C3orf80**	0.045501	4.9533	1.6901356616091900	18041
**CTAGE1**	0.045501	4.4061	1.6901356616091900	18043
**OR10H4**	0.045501	4.9381	1.6901356616091900	18042
**STRC**	0.045415	0.55745	1.691035599344760	18044
**ATE1**	0.045237	3.1765	1.6929026242361400	18045

**PTN**	0.045188	5.1998	1.6934176177644000	18046
**PRR5L**	0.045164	5.926	1.6936700234409400	18047
**PPT2**	0.045122	4.2997	1.6941119931889900	18048
**AURKAIP1**	0.044741	4.411	1.698136490501740	18050
**FKBP1A**	0.044741	1.9455	1.698136490501740	18052
**POU3F2**	0.044741	4.2801	1.698136490501740	18051
**SEZ6**	0.044741	4.7089	1.698136490501740	18049
**FGF7**	0.044678	6.7751	1.6988046148204100	18053
**ATRNL1**	0.044573	3.4442	1.6999198434448300	18054
**ABCC2**	0.044568	5.5529	1.6999730023317300	18057
**ACTL6B**	0.044568	6.3256	1.6999730023317300	18055
**GIMAP1-GIM**	0.044568	5.5411	1.6999730023317300	18058
**SLC16A12**	0.044568	5.511	1.6999730023317300	18059
**TAS2R38**	0.044568	5.6607	1.6999730023317300	18056
**TSC22D1**	0.044568	5.4922	1.6999730023317300	18060
**DRG1**	0.044213	3.6996	1.7037596242792700	18061
**EIF2S2**	0.044114	4.461	1.7048199803966500	18062
**NAB1**	0.044114	4.3451	1.7048199803966500	18064
**NUDCD3**	0.044114	1.4409	1.7048199803966500	18065
**SLC9A4**	0.044114	4.3532	1.7048199803966500	18063
**NDUFS7**	0.044075	5.8106	1.7052382232346500	18067
**PANK4**	0.044075	6.0765	1.7052382232346500	18066
**CPSF2**	0.044052	4.6918	1.7054850191658200	18068
**VPS54**	0.044015	3.0881	1.7058822568100200	18069
**COX8C**	0.043955	4.927	1.7065269987285200	18070
**APOL6**	0.043885	4.2911	1.7072800953402000	18071
**ADAM7**	0.043792	3.3636	1.708282138195480	18073
**CUL2**	0.043792	0.35876	1.708282138195480	18076
**HTR1E**	0.043792	2.6759	1.708282138195480	18074
**OSBPL6**	0.043792	2.4832	1.708282138195480	18075
**ZNF620**	0.043792	3.5658	1.708282138195480	18072
**SPRR2F**	0.043738	4.7675	1.7088647575700500	18077

**TAAR5**	0.043617	4.5111	1.710172366799080	18078
**PCK1**	0.043578	2.0586	1.7105944514136100	18079
**C5AR2**	0.043521	6.0169	1.711211893043300	18081
**DEFB115**	0.043521	6.3157	1.711211893043300	18080
**POTEG**	0.043492	6.7877	1.7115262805634400	18083
**TOR1A**	0.043492	6.7877	1.7115262805634400	18082
**AKR1C2**	0.043381	3.4552	1.7127311915307200	18084
**TRIM36**	0.043267	0.16047	1.7139712614032600	18085
**PUS10**	0.043199	3.4701	1.714712209014480	18086
**IL4I1**	0.043171	3.8985	1.71501757889794	18087
**ASB14**	0.043027	4.3285	1.7165905843868400	18088
**CASR**	0.043027	1.8938	1.7165905843868400	18090
**NPHS2**	0.043027	1.5667	1.7165905843868400	18091
**PI4KB**	0.043027	4.1788	1.7165905843868400	18089
**TMED2**	0.042967	5.0807	1.7172472587735900	18092
**UBA3**	0.042967	4.5245	1.7172472587735900	18093
**BPIFB3**	0.042962	5.4673	1.717302015075870	18095
**PARD3B**	0.042962	5.647	1.717302015075870	18094
**KRTAP4-9**	0.042954	6.7939	1.7173896358707300	18096
**RCC1L**	0.042918	2.9921	1.717784092704790	18098
**TMPRSS6**	0.042918	3.1559	1.717784092704790	18097
**MARCH1**	0.042813	1.1631	1.7189361210451200	18099
**NUP205**	0.042685	3.2771	1.7203435912510900	18100
**ZNF585A**	0.04254	5.6922	1.7219421190678100	18101
**C8G**	0.04246	3.1648	1.7228259523297900	18102
**HIRIP3**	0.042398	4.2531	1.7235118499336200	18103
**MAT2A**	0.042379	1.5859	1.723722206763720	18106
**MSN**	0.042379	4.5571	1.723722206763720	18105
**SLC6A12**	0.042379	4.806	1.723722206763720	18104
**CUX2**	0.042329	2.4501	1.7242761421402400	18107
**HACE1**	0.042045	6.0745	1.7274325772098400	18108
**PRSS22**	0.041975	1.5109	1.7282132215481500	18109

**FAM181B**	0.041964	3.5333	1.7283359900280300	18110
**EXOG**	0.041941	4.6253	1.7285927719687300	18111
**ARMC7**	0.041718	3.2978	1.7310883706517200	18112
**CFHR5**	0.041704	4.2231	1.7312454051007800	18113
**MRM2**	0.041647	2.8396	1.731885200803430	18115
**STRBP**	0.041647	1.518	1.731885200803430	18117
**SYS1**	0.041647	5.1979	1.731885200803430	18114
**VPS53**	0.041647	1.9829	1.731885200803430	18116
**FAXDC2**	0.041623	4.3483	1.732154800686100	18118
**ANTXRL**	0.041567	3.6028	1.7327843571569400	18119
**ZMYM1**	0.041567	2.0925	1.7327843571569400	18121
**ZNF416**	0.041567	2.9089	1.7327843571569400	18120
**KCNQ4**	0.041517	4.7147	1.7333470420715600	18122
**NME1-NME2**	0.041502	5.1532	1.733515954610040	18123
**ZNF765**	0.041431	3.1133	1.7343161458404600	18124
**DHX57**	0.041373	5.54	1.7349706482792500	18127
**POC1B**	0.041373	5.7679	1.7349706482792500	18126
**TEX22**	0.041373	5.8994	1.7349706482792500	18125
**FOXD4L5**	0.041326	5.1016	1.735501566623470	18128
**PAK1**	0.041321	4.5897	1.7355580760955900	18129
**OR4A47**	0.040965	4.7971	1.7395958738497300	18130
**RNF34**	0.040965	2.3616	1.7395958738497300	18132
**VAMP5**	0.040965	3.8811	1.7395958738497300	18131
**CREM**	0.040932	2.2934	1.7399716034588800	18136
**GPKOW**	0.040932	2.2594	1.7399716034588800	18137
**ISOC2**	0.040932	2.4139	1.7399716034588800	18135
**NLRC3**	0.040932	2.0006	1.7399716034588800	18138
**PURG**	0.040932	5.0477	1.7399716034588800	18133
**TK1**	0.040932	3.6154	1.7399716034588800	18134
**EBAG9**	0.040739	4.64	1.7421739870428200	18139
**VTN**	0.040611	6.2553	1.743639309137360	18140
**TMEM178B**	0.040598	4.3299	1.7437883405029200	18141

**PYCARD**	0.040504	3.6939	1.7448671061753100	18142
**TUBB2A**	0.040504	2.9575	1.7448671061753100	18143
**CYBA**	0.040388	4.0923	1.7462011541967300	18144
**DCN**	0.040388	4.0888	1.7462011541967300	18145
**OR8B4**	0.040388	3.7525	1.7462011541967300	18146
**TFEB**	0.040251	0.32882	1.747780724088720	18147
**ALYREF**	0.040023	4.3343	1.7504192056425600	18148
**INPP5F**	0.039767	4.3059	1.753396307460560	18149
**MLANA**	0.039767	1.2403	1.753396307460560	18150
**CAPN9**	0.039737	4.3507	1.7537462055488000	18151
**MAGEA2**	0.039719	4.5045	1.753956247505660	18152
**PFKP**	0.039696	6.0986	1.754224747108570	18153
**UXS1**	0.039569	4.3517	1.7557096138147400	18154
**MAGEA6**	0.03954	4.3428	1.7560492213267500	18155
**GNG2**	0.039486	3.7261	1.7566821341539100	18156
**ST6GALNAC**	0.039486	1.7168	1.7566821341539100	18157
**TIGAR**	0.039486	0.84164	1.7566821341539100	18158
**OR10V1**	0.039413	5.6992	1.7575388587562800	18159
**IBA57**	0.039362	3.7416	1.7581381586940700	18160
**SPIN2B**	0.039233	3.2337	1.7596568601126500	18161
**ARX**	0.039205	1.2111	1.7599870373648300	18162
**CEACAM18**	0.039067	3.7501	1.761617149279630	18163
**SULT4A1**	0.03904	4.5458	1.7619366323409900	18164
**TFAP2D**	0.03904	3.2604	1.7619366323409900	18165
**CHML**	0.03893	4.5235	1.7632400923815500	18166
**LEKR1**	0.03893	3.9337	1.7632400923815500	18167
**E2F6**	0.038859	5.6292	1.7640830100952200	18169
**GOPC**	0.038859	6.1666	1.7640830100952200	18168
**PRR4**	0.038823	4.3637	1.764510884315840	18170
**CAST**	0.038767	4.3043	1.7651771092232700	18171
**GPR173**	0.038594	4.1105	1.7672402324602100	18172
**CHRNA9**	0.038579	3.8374	1.7674194707406000	18173

**C4orf46**	0.038399	2.1057	1.7695747723909000	18177
**RASA1**	0.038399	3.8591	1.7695747723909000	18176
**RFLNB**	0.038399	4.0691	1.7695747723909000	18175
**ZNF680**	0.038399	4.5364	1.7695747723909000	18174
**FAM151B**	0.038317	4.7436	1.7705593634341400	18178
**NRP2**	0.038317	4.2117	1.7705593634341400	18179
**C1orf167**	0.038295	5.6777	1.7708238142534600	18182
**DHRS1**	0.038295	5.6943	1.7708238142534600	18181
**DNAJC9**	0.038295	5.8007	1.7708238142534600	18180
**DCBLD1**	0.038234	3.6084	1.7715577127081700	18184
**OR5L2**	0.038234	4.0929	1.7715577127081700	18183
**RHOB**	0.038225	5.0907	1.7716660736191200	18185
**AMER3**	0.038028	4.0998	1.7740432020721600	18186
**IGFL1**	0.038021	4.2479	1.7741278531953400	18187
**CAMP**	0.03795	0.089058	1.774987176734160	18188
**ESYT1**	0.03787	1.6032	1.7759570017116700	18190
**SCNN1A**	0.03787	4.3851	1.7759570017116700	18189
**ZNF662**	0.037863	4.594	1.776041940911910	18191
**TWNK**	0.037801	4.2758	1.7767948194907300	18192
**APOPT1**	0.037735	5.5709	1.777597379132490	18194
**DOCK1**	0.037735	5.4575	1.777597379132490	18195
**UGGT2**	0.037735	5.5839	1.777597379132490	18193
**FBXO40**	0.037727	6.304	1.777694736926970	18196
**MT1M**	0.037714	3.8016	1.7778529792900300	18197
**ZNF681**	0.037714	2.2047	1.7778529792900300	18198
**ISYNA1**	0.037601	2.7843	1.779230349582200	18199
**CYP 20.00**	0.037555	5.0376	1.7797920170021400	18200
**OR2B6**	0.037374	0.55039	1.7820075251907200	18201
**ACOX1**	0.037219	0.0583	1.783911760577070	18204
**HIBCH**	0.037219	2.5994	1.783911760577070	18202
**IDNK**	0.037219	0.063884	1.783911760577070	18203
**MARCH10**	0.037184	4.6729	1.784342645686380	18205

**ADCY9**	0.037156	4.76	1.7846875924540200	18206
**ADPRHL2**	0.036956	4.2428	1.7871576955295500	18208
**RNF181**	0.036956	4.8489	1.7871576955295500	18207
**INPP5J**	0.036942	4.8954	1.7873310116169000	18209
**PPP5C**	0.036753	2.9571	1.789676050959320	18210
**C1orf56**	0.03675	2.6824	1.7897133532358700	18211
**MTRNR2L6**	0.03675	1.6475	1.7897133532358700	18212
**LRRC56**	0.036741	4.5526	1.7898252750099600	18213
**CACNG5**	0.036623	4.2589	1.7912947721386300	18214
**AGER**	0.036305	4.5105	1.795274318687200	18215
**FAM118B**	0.036294	5.0924	1.795412485638520	18216
**PTGES3**	0.036294	4.487	1.795412485638520	18217
**PNOC**	0.036162	4.4755	1.797073168773140	18218
**SRGAP3**	0.036162	3.5254	1.797073168773140	18219
**VPS4B**	0.036152	4.665	1.7971991802282600	18220
**CSMD3**	0.036102	5.6454	1.7978296659627300	18221
**MUM1L1**	0.035955	4.9187	1.799687444924480	18222
**CACNG1**	0.035934	3.3593	1.7999533495446500	18223
**CLCA1**	0.035905	6.9137	1.8003207605170800	18224
**BTRC**	0.035747	4.7746	1.8023267983897000	18225
**NT5C3A**	0.035747	2.0989	1.8023267983897000	18226
**QDPR**	0.035597	3.7986	1.8042379994860300	18227
**POU3F4**	0.035586	5.8117	1.8043784138560500	18228
**C2CD6**	0.035583	1.4307	1.8044167148591800	18230
**GSDMC**	0.035583	0.25261	1.8044167148591800	18231
**TSLP**	0.035583	4.4873	1.8044167148591800	18229
**PLEKHA1**	0.035582	4.5988	1.8044294824484700	18232
**KLK3**	0.035243	4.5186	1.8087747492653700	18234
**OR5H2**	0.035243	4.294	1.8087747492653700	18235
**RAG2**	0.035243	4.6709	1.8087747492653700	18233
**EML2**	0.035181	3.9819	1.8095731638907600	18236
**HIST1H2AE**	0.035163	3.0593	1.8098051778912100	18237

**ZNF581**	0.035157	5.0511	1.809882537545290	18238
**RANGRF**	0.03511	4.299	1.8104888965625600	18239
**TSPAN15**	0.035034	4.3605	1.8114708027517800	18240
**SOX15**	0.035001	3.8501	1.8118977011218400	18241
**PDIA6**	0.034887	3.7651	1.8133749872944100	18242
**YTHDC1**	0.034887	2.3126	1.8133749872944100	18243
**P4HB**	0.034682	5.6465	1.8160415107674000	18244
**XPO5**	0.034682	4.9557	1.8160415107674000	18245
**PRR7**	0.034647	4.3424	1.8164980636472300	18246
**RCBTB1**	0.034647	2.5076	1.8164980636472300	18247
**CXorf57**	0.034593	6.414	1.8172032031295900	18248
**GJA3**	0.03458	2.6533	1.8173730939473000	18249
**ACTR2**	0.034529	3.0073	1.818040095745950	18250
**DDIT4**	0.034529	2.5243	1.818040095745950	18251
**GOLGA6L10**	0.034431	4.7465	1.8193240605740000	18252
**ANKS3**	0.034357	6.1896	1.8202955762562200	18253
**NFRKB**	0.03426	2.7055	1.8215716572814800	18254
**PIFO**	0.034226	4.8655	1.8220196463026000	18255
**AMFR**	0.0342	2.5757	1.822362473038990	18257
**KRTAP4-12**	0.0342	4.684	1.822362473038990	18256
**ERCC3**	0.034111	3.3691	1.8235376196502700	18259
**UBALD2**	0.034111	4.2703	1.8235376196502700	18258
**ANKZF1**	0.033994	6.9422	1.8250863167940300	18260
**DKK 1.00**	0.033982	4.1519	1.8252454052510700	18261
**CAMK2N1**	0.033888	4.1004	1.8264931992986100	18262
**PEX7**	0.033789	4.7917	1.827810447440440	18263
**C2orf71**	0.033698	2.2474	1.8290240550645300	18266
**KIAA0040**	0.033698	2.7968	1.8290240550645300	18265
**LOC284570**	0.033698	4.4901	1.8290240550645300	18264
**ZSCAN9**	0.033663	3.6067	1.8294915453718500	18267
**ACADM**	0.033644	3.6956	1.8297454933566400	18270
**CLCNKB**	0.033644	5.075	1.8297454933566400	18268

**TM9SF1**	0.033644	4.1451	1.8297454933566400	18269
**LRRTM3**	0.033261	5.487	1.8348898983082400	18271
**AHDC1**	0.033242	0.54704	1.8351463712781100	18274
**ATP1A3**	0.033242	4.9435	1.8351463712781100	18272
**NUDT17**	0.033242	3.7864	1.8351463712781100	18273
**BNC1**	0.033211	3.419	1.835565086505830	18275
**GOLIM4**	0.033192	4.2197	1.835821877571770	18276
**NOS1**	0.033192	3.3283	1.835821877571770	18277
**RNF216**	0.033192	1.6162	1.835821877571770	18278
**AP3S2**	0.033168	4.199	1.8361464183666800	18279
**DGKZ**	0.033114	4.8932	1.8368773430826700	18281
**SSPN**	0.033114	5.2363	1.8368773430826700	18280
**MEIS3**	0.032975	5.332	1.8387633250475200	18282
**KLHL21**	0.032917	6.9645	1.8395522191814700	18283
**BCAR3**	0.032843	3.066	1.8405604043574100	18284
**SON**	0.032787	4.637	1.8413246007484800	18285
**AQP12A**	0.032769	6.057	1.8415704638271600	18286
**FOXE1**	0.032752	1.8252	1.841802770105810	18287
**ELOVL2**	0.032641	3.4711	1.8433220421989800	18289
**TSR1**	0.032641	3.5784	1.8433220421989800	18288
**THNSL1**	0.032631	6.9701	1.8434591226576500	18290
**RNF139**	0.03244	4.5818	1.8460840338919800	18291
**KCTD15**	0.03235	4.4325	1.8473253247549600	18292
**MMP25**	0.03235	3.9005	1.8473253247549600	18293
**OSGIN1**	0.032304	2.9016	1.847960863077180	18294
**BRCA2**	0.032302	5.9867	1.8479885121152500	18295
**PSKH2**	0.032302	5.7283	1.8479885121152500	18296
**OTC**	0.032281	2.9199	1.8482789123446200	18297
**IL17C**	0.032179	3.5919	1.8496916496201400	18298
**SLC5A2**	0.032006	5.1096	1.8520962373380000	18299
**CILP2**	0.031871	4.1159	1.8539801162806400	18301
**UBTF**	0.031871	3.8655	1.8539801162806400	18302

**ZFP1**	0.031871	4.6337	1.8539801162806400	18300
**LAMTOR2**	0.031852	6.2025	1.8542457836206800	18303
**UBE2Z**	0.031805	3.8667	1.8549035235888100	18304
**CD14**	0.031723	0.93731	1.85605299495706	18306
**ZNF229**	0.031723	2.1648	1.85605299495706	18305
**CLEC18C**	0.031377	4.0116	1.8609304096653700	18308
**TIMM13**	0.031377	4.3719	1.8609304096653700	18307
**TMEM163**	0.031377	3.4241	1.8609304096653700	18309
**UBQLNL**	0.031377	2.1551	1.8609304096653700	18310
**OR5F1**	0.031315	5.9929	1.8618090903138300	18311
**RETREG1**	0.031292	2.1477	1.8621354183377100	18312
**FAM231A**	0.031063	4.6149	1.8653953782575200	18313
**AHSA1**	0.030937	1.879	1.8671975565338700	18315
**PRSS33**	0.030937	1.6215	1.8671975565338700	18316
**ZNF781**	0.030937	2.534	1.8671975565338700	18314
**EIF2B3**	0.030908	4.9	1.8676132033008700	18317
**LZTFL1**	0.030908	4.8696	1.8676132033008700	18318
**HAT1**	0.030791	6.3431	1.86929340825829	18319
**MKX**	0.030759	4.4504	1.8697538718293900	18320
**PBRM1**	0.030748	7.0029	1.8699122477814900	18321
**GNA15**	0.030622	2.0266	1.8717297268467800	18322
**PPID**	0.030597	2.9161	1.8720910738158600	18323
**TAF15**	0.030592	3.883	1.8721633725504800	18324
**CSRP3**	0.030452	0.040698	1.8741917218738600	18325
**SH3TC1**	0.030433	3.8133	1.8744675928696200	18326
**LINC01862**	0.030391	4.3629	1.875077919680750	18327
**IFNAR2**	0.030321	5.702	1.8760966858593200	18329
**PVALB**	0.030321	6.1426	1.8760966858593200	18328
**PLEK2**	0.03032	3.2263	1.8761112537756600	18330
**SRP68**	0.030316	5.4209	1.8761695294229500	18331
**KRTAP11-1**	0.030255	4.0633	1.8770590235457500	18332
**NAPRT**	0.030185	3.9815	1.878081587881790	18333

**ANKRD54**	0.030148	5.1532	1.8786228804844100	18334
**CORO1B**	0.030148	5.0109	1.8786228804844100	18335
**CRABP2**	0.030148	4.5713	1.8786228804844100	18337
**ZNF273**	0.030148	4.7515	1.8786228804844100	18336
**ALDH1A3**	0.030119	5.0948	1.8790475219557400	18338
**SRRM4**	0.030094	4.0196	1.8794138643348900	18339
**SETDB1**	0.02997	5.2046	1.8812346610335100	18340
**RSAD1**	0.029909	5.852	1.8821326690866400	18341
**NUFIP1**	0.029902	0.57859	1.8822358163243200	18343
**SLC26A1**	0.029902	1.0286	1.8822358163243200	18342
**KRT222**	0.029829	3.1822	1.8833126899299100	18344
**KCNK16**	0.029776	4.8712	1.884095900302250	18345
**RHBDD1**	0.029776	2.4666	1.884095900302250	18346
**SVIP**	0.029643	5.1719	1.8860664203227600	18347
**ZNF215**	0.02961	3.5743	1.8865564821362200	18348
**RAB35**	0.02955	4.6674	1.8874486661338300	18349
**CHCHD3**	0.029478	3.1872	1.888521273948280	18351
**LDB1**	0.029478	5.1537	1.888521273948280	18350
**DNAJC2**	0.029358	4.6294	1.8903137966964700	18352
**PLD5**	0.029358	2.2997	1.8903137966964700	18354
**RARB**	0.029358	2.8858	1.8903137966964700	18353
**NAT8L**	0.029296	4.9248	1.8912423183277600	18355
**PRPF18**	0.029279	4.019	1.8914971980285100	18356
**HIST1H3I**	0.029067	3.0593	1.8946860673856700	18357
**ARHGEF37**	0.02906	5.3153	1.8947916893570400	18358
**C5orf42**	0.02906	4.7497	1.8947916893570400	18359
**H2AFJ**	0.02906	4.3089	1.8947916893570400	18360
**IREB2**	0.029037	3.6023	1.8951388818896900	18361
**SFTPA1**	0.029037	2.1442	1.8951388818896900	18362
**GNAT3**	0.028852	4.8562	1.8979398610245600	18363
**KIAA1109**	0.028653	5.7295	1.9009695233500100	18364
**ARHGAP26**	0.028611	0.88287	1.9016111846771900	18365

**AVL9**	0.028543	4.6344	1.9026517278986700	18367
**NCOR1**	0.028543	4.6597	1.9026517278986700	18366
**SPG21**	0.028481	5.1762	1.9036022572299300	18368
**VANGL1**	0.028384	3.8015	1.9050928349912200	18369
**CDK2AP1**	0.028236	5.7851	1.9073753071831800	18371
**MROH2B**	0.028236	6.3392	1.9073753071831800	18370
**GRN**	0.028172	4.1645	1.9083654074193500	18372
**TP53**	0.028127	4.8218	1.9090626934222300	18373
**USH1C**	0.02811	1.7911	1.909326354310390	18374
**FLYWCH2**	0.028022	2.6183	1.9106933134599100	18375
**MTHFD1L**	0.027891	3.97	1.9127348556880200	18376
**ZFYVE21**	0.027803	4.6041	1.9141107629115700	18377
**GPR22**	0.027781	7.0663	1.9144553065562700	18378
**CTTNBP2NL**	0.027674	4.5634	1.9161342906240500	18380
**GART**	0.027674	4.7159	1.9161342906240500	18379
**OR5P2**	0.027674	1.4436	1.9161342906240500	18381
**SCUBE1**	0.027674	0.27446	1.9161342906240500	18382
**RLF**	0.027665	4.8057	1.9162757601018500	18384
**SELENOT**	0.027665	5.3579	1.9162757601018500	18383
**PRAMEF19**	0.027507	4.9458	1.918765604982570	18385
**GAST**	0.027382	5.1177	1.9207438809125000	18386
**HMGXB3**	0.027377	7.0766	1.9208231684652700	18387
**TEX10**	0.027375	0.8549	1.9208548868677400	18388
**LCE1F**	0.02727	6.0334	1.9225228235683500	18389
**TREX1**	0.02727	5.8872	1.9225228235683500	18390
**C16orf62**	0.027218	5.0209	1.9233508335688100	18391
**SPATA31C2**	0.027218	4.1352	1.9233508335688100	18393
**TMEM254**	0.027218	4.5381	1.9233508335688100	18392
**CHRDL1**	0.027092	2.399	1.9253626523457400	18395
**LUZP1**	0.027092	3.1197	1.9253626523457400	18394
**FAM184A**	0.026867	4.6164	1.9289746889133500	18396
**ZDHHC7**	0.026843	7.0868	1.929361461587260	18397

**ADAMTS12**	0.02684	4.1152	1.9294098284701600	18398
**YARS2**	0.026814	4.53	1.9298291973106900	18399
**BCL2L12**	0.026682	4.9734	1.9319635536113000	18400
**SLC10A5**	0.026615	7.0919	1.9330502771766400	18401
**KCNJ10**	0.026551	4.1589	1.934090477647220	18402
**FGD3**	0.026503	4.4936	1.9348720035922200	18404
**KRTAP20-1**	0.026503	3.7876	1.9348720035922200	18405
**SDC4**	0.026503	4.9315	1.9348720035922200	18403
**PRDX6**	0.026369	2.3606	1.9370600405262800	18406
**RPUSD1**	0.026369	1.3414	1.9370600405262800	18407
**SCPEP1**	0.026285	5.7624	1.9384363889847700	18408
**FKRP**	0.026266	5.1179	1.938748215689210	18409
**ADGRG4**	0.02609	7.102	1.941645716153980	18410
**OR4C46**	0.025873	5.8854	1.9452407916644900	18411
**C12orf45**	0.025849	2.6635	1.9456399513326900	18414
**CX3CR1**	0.025849	4.9691	1.9456399513326900	18412
**USP32**	0.025849	4.5449	1.9456399513326900	18413
**IL10RB**	0.025564	4.3078	1.9504038409919800	18415
**ARHGEF6**	0.025531	7.1121	1.9509583175003300	18416
**OTOP1**	0.025468	6.1587	1.9520185321960900	18417
**F3**	0.025408	4.4587	1.9530303044950800	18418
**DGKQ**	0.02527	3.1227	1.9553649978937600	18420
**NCKAP1**	0.02527	4.8725	1.9553649978937600	18419
**CDCP1**	0.025011	2.1907	1.959775770962210	18424
**LRRN4**	0.025011	4.2789	1.959775770962210	18422
**PFDN2**	0.025011	4.2944	1.959775770962210	18421
**RCN1**	0.025011	2.5001	1.959775770962210	18423
**OR2B2**	0.025007	5.9088	1.959844190643610	18425
**FCGR3A**	0.024998	5.1347	1.9599981684798500	18426
**FTO**	0.02493	3.013	1.9611630612282500	18427
**H1F0**	0.02493	1.9962	1.9611630612282500	18428
**CARS2**	0.024867	4.1664	1.9622446801597500	18429

**UBE2F**	0.024764	3.3655	1.9640180014956500	18430
**TNFAIP8L1**	0.02471	5.1602	1.9649501772695300	18431
**B3GNT2**	0.024652	4.833	1.9659533086346000	18432
**MAFA**	0.024652	3.7634	1.9659533086346000	18433
**TNNT1**	0.024567	3.9817	1.9674269985212200	18434
**RPS6**	0.024552	1.5363	1.9676875054731800	18435
**GNG8**	0.024515	4.8657	1.9683306608405100	18436
**YBX1**	0.024497	4.6162	1.9686438418601100	18437
**AK3**	0.024388	4.8203	1.9705444640847600	18438
**ARG1**	0.02433	0.29893	1.9715587130232000	18440
**BACH2**	0.02433	2.2733	1.9715587130232000	18439
**WDR46**	0.024261	6.0294	1.9727679671630100	18441
**SOAT2**	0.024211	5.1447	1.9736460442983100	18442
**UPF3B**	0.024211	5.1274	1.9736460442983100	18443
**CPSF1**	0.024192	4.7736	1.9739801129739700	18444
**CCDC36**	0.024018	2.33	1.9770497739195500	18446
**WDR89**	0.024018	5.9508	1.9770497739195500	18445
**CEACAM8**	0.023808	4.7575	1.980779522186800	18447
**FEM1A**	0.023738	3.8895	1.9820289175239100	18448
**GYG2**	0.023654	0.37682	1.9835322875816700	18449
**GPR161**	0.023509	6.0986	1.9861379879254900	18450
**FOSL1**	0.023443	4.8292	1.987328510540150	18451
**MIEF1**	0.023382	2.1943	1.9884313523797400	18452
**PRDX4**	0.023381	3.7877	1.988449451914040	18453
**FAM72A**	0.02327	4.9317	1.990462561566020	18454
**STX1B**	0.02327	3.3731	1.990462561566020	18455
**CHRM5**	0.023183	4.7629	1.992046062205270	18456
**NOP58**	0.023143	6.1919	1.9927757878462400	18457
**TSEN15**	0.023143	5.9655	1.9927757878462400	18458
**SNF8**	0.02312	4.6268	1.993195861069180	18459
**PAGE4**	0.023107	3.2497	1.993433449430540	18460
**ARL 11.00**	0.023016	7.171	1.99509972691977	18461

**NHP2**	0.022978	5.5693	1.9957971777083400	18462
**RFFL**	0.022978	4.7603	1.9957971777083400	18463
**GCAT**	0.022954	5.549	1.996238173654110	18464
**CLN8**	0.022847	4.4398	1.9982090193039100	18465
**CMC1**	0.022847	4.0009	1.9982090193039100	18468
**PRAMEF25**	0.022847	4.1196	1.9982090193039100	18467
**RPL37A**	0.022847	4.2803	1.9982090193039100	18466
**TRPM3**	0.022847	3.7534	1.9982090193039100	18469
**GPR137B**	0.022823	4.5731	1.9986521455894200	18470
**CPEB1**	0.022776	6.4611	1.999521073130250	18471
**PDILT**	0.022776	6.4401	1.999521073130250	18472
**OR4E2**	0.022759	5.2869	1.9998357381964200	18473
**C17orf100**	0.022742	0.32999	2.0001506013992700	18474
**CD3E**	0.022668	0.44755	2.001523497738750	18475
**ADAMTS16**	0.022551	5.0005	2.0037018863955400	18476
**ALG2**	0.022551	4.1211	2.0037018863955400	18477
**LYSMD3**	0.022528	5.1551	2.004131236887810	18479
**TMEM213**	0.022528	5.7233	2.004131236887810	18478
**JPT2**	0.022331	5.1696	2.0078239386371600	18480
**ZMYM4**	0.022264	5.2878	2.009086094821150	18481
**HELLS**	0.022229	1.1221	2.00974670488021	18482
**E2F2**	0.022197	4.2939	2.0103514596961800	18483
**PCMTD2**	0.022092	5.735	2.012340992800380	18484
**FYN**	0.022068	3.9055	2.0127968635155800	18485
**PGM1**	0.02195	4.6584	2.015044334085550	18486
**CSHL1**	0.021926	3.9206	2.0155026946229900	18487
**KATNA1**	0.021916	4.7963	2.015693803220660	18488
**SLC39A5**	0.021916	4.7743	2.015693803220660	18489
**MMGT1**	0.021805	0.89413	2.0178200688518500	18490
**RNPC3**	0.021725	6.4193	2.019358188542970	18491
**SEPT9**	0.021725	5.9361	2.019358188542970	18492
**ITCH**	0.021659	5.0876	2.0206307435293900	18493

**ADAMTSL2**	0.021506	0.28856	2.023593407161080	18497
**GIT1**	0.021506	1.0938	2.023593407161080	18496
**LOC1001296**	0.021506	1.9655	2.023593407161080	18494
**TSTA3**	0.021506	1.3438	2.023593407161080	18495
**TMPRSS13**	0.021453	5.5723	2.0246238441427700	18498
**FOPNL**	0.021312	4.8845	2.027375711683730	18500
**SEC14L3**	0.021312	5.3328	2.027375711683730	18499
**GARS**	0.021183	4.5666	2.0299068969317000	18501
**DPPA4**	0.021176	5.4838	2.03004462063713	18502
**SMYD4**	0.021155	5.0259	2.03045802294787	18503
**AKTIP**	0.02114	3.7789	2.030753522907110	18505
**NDUFV1**	0.02114	3.9886	2.030753522907110	18504
**PRR5-ARHG**	0.021133	5.4346	2.0308914835948500	18506
**CAMTA2**	0.021078	6.0501	2.0319768077509800	18507
**IL1R2**	0.02105	2.9146	2.032530257127570	18508
**USP51**	0.020948	5.1824	2.0345516767342000	18509
**GOLGA6D**	0.020811	7.2229	2.0372798705887800	18510
**EMC4**	0.020784	6.5724	2.0378193365179200	18511
**ASPM**	0.020734	2.3832	2.0388199163395600	18516
**NYNRIN**	0.020734	2.8403	2.0388199163395600	18515
**SEH1L**	0.020734	4.4056	2.0388199163395600	18514
**SIK1**	0.020734	5.5036	2.0388199163395600	18512
**TTLL2**	0.020734	4.8321	2.0388199163395600	18513
**NKX6-2**	0.020718	6.7798	2.0391405334140500	18517
**MPG**	0.020677	4.8594	2.0399630727989000	18518
**RIPOR2**	0.020677	4.4778	2.0399630727989000	18519
**NPPA**	0.020505	5.142	2.0434288517861200	18521
**SAMD14**	0.020505	5.6076	2.0434288517861200	18520
**TSC2**	0.020505	4.7209	2.0434288517861200	18522
**DDR2**	0.020408	6.559	2.045394266178660	18523
**PFN2**	0.020383	4.2405	2.045902099174680	18524
**TCAF2**	0.020383	4.1553	2.045902099174680	18525

**JAK3**	0.020328	2.7646	2.047021192677950	18526
**OGFOD3**	0.020241	5.1357	2.0487966472806900	18527
**ADM5**	0.020116	5.2039	2.051358948281430	18529
**MRPS15**	0.020116	5.3797	2.051358948281430	18528
**ACKR3**	0.020016	5.3088	2.0534185296033500	18530
**HYPM**	0.019981	3.8292	2.0541414453797800	18531
**MTERF1**	0.019801	5.1691	2.0578763587083200	18532
**TJP1**	0.019801	4.3538	2.0578763587083200	18533
**SH3KBP1**	0.019661	1.887	2.060801265972520	18534
**GSTM1**	0.019599	5.4972	2.062102234687340	18535
**RBP7**	0.019599	3.1276	2.062102234687340	18538
**SIM2**	0.019599	4.7772	2.062102234687340	18537
**SRP54**	0.019599	4.8573	2.062102234687340	18536
**HAAO**	0.019532	6.0924	2.063512055122870	18540
**KIAA1683**	0.019532	6.2552	2.063512055122870	18539
**ITGAV**	0.019412	5.7753	2.0660474055777600	18541
**LPA**	0.019412	5.7495	2.0660474055777600	18542
**ZBTB45**	0.019355	3.6332	2.067256364281520	18543
**TACR1**	0.019277	3.0886	2.068915640752330	18544
**ACVRL1**	0.019144	3.7411	2.071758126028040	18546
**CPNE5**	0.019144	4.5902	2.071758126028040	18545
**CYP2A13**	0.019058	4.0969	2.073605074265360	18547
**ZFP14**	0.019058	2.7353	2.073605074265360	18548
**TMEM179B**	0.018903	6.6639	2.076951849485880	18549
**PRKCG**	0.0188	4.0645	2.0791887690906000	18550
**RUSC1**	0.018688	4.7053	2.081633013703470	18551
**HOXB4**	0.018662	6.4287	2.082202210128700	18552
**SHC2**	0.018461	4.473	2.0866254660054000	18553
**ITM2C**	0.018389	5.1375	2.088219895248800	18554
**KRTAP4-5**	0.018375	4.0858	2.0885305404610900	18555
**HAP1**	0.018219	4.6139	2.09200572676998	18556
**CHAMP1**	0.018115	4.6662	2.09433663099349	18557

**CAPN8**	0.018064	5.9379	2.095483841376440	18558
**ACTL6A**	0.017845	3.5364	2.1004417078137500	18559
**LIMS1**	0.017845	2.6261	2.1004417078137500	18560
**A1CF**	0.017769	3.0053	2.102174378567820	18561
**TRH**	0.017607	4.9229	2.1058889090734500	18562
**MAP3K10**	0.017498	2.9644	2.108404641766550	18563
**ADPRM**	0.01743	4.4891	2.109980874640230	18565
**PUS7L**	0.01743	2.7255	2.109980874640230	18566
**SLC35D3**	0.01743	5.457	2.109980874640230	18564
**NGDN**	0.017405	2.4308	2.1105616922887900	18567
**PUM2**	0.017339	4.8411	2.1120984812033000	18568
**KLF9**	0.017301	5.4988	2.1129855668867900	18569
**OR52D1**	0.017126	4.6434	2.1170924397414000	18570
**PROKR1**	0.017122	4.4531	2.1171867295396900	18571
**PTGR1**	0.017122	3.5791	2.1171867295396900	18572
**DEFB103A**	0.017069	4.5996	2.118437849958330	18573
**PRRG3**	0.016925	6.1084	2.121853966144100	18574
**ATG13**	0.01688	4.7651	2.1229265986678000	18576
**FABP4**	0.01688	5.8775	2.1229265986678000	18575
**FGF16**	0.016823	2.3173	2.1242887824861600	18578
**OR8J3**	0.016823	2.9528	2.1242887824861600	18577
**ITGA2B**	0.016754	5.7979	2.1259430333689500	18579
**OR2H2**	0.016646	6.6094	2.128544033695950	18580
**SHARPIN**	0.016558	5.4291	2.130674065373880	18581
**CCT6B**	0.016524	3.4653	2.131499627592	18582
**SLC39A11**	0.016354	5.1058	2.1356493827211	18583
**CCNT2**	0.016345	6.3878	2.1358701034084900	18585
**FAM240A**	0.016345	6.4765	2.1358701034084900	18584
**DBN1**	0.016298	5.1555	2.1370244497455300	18586
**PHAX**	0.016298	4.9216	2.1370244497455300	18587
**CXCR1**	0.016144	0.068551	2.1408268605338400	18588
**CRY1**	0.015949	2.5403	2.1456864498142500	18590

**EBF4**	0.015949	3.6496	2.1456864498142500	18589
**SDS**	0.015933	2.9135	2.14608744231543	18591
**ABCA2**	0.015902	5.8231	2.146865348715060	18593
**CYTH3**	0.015902	5.8709	2.146865348715060	18592
**DHRS7B**	0.015568	2.9092	2.155330216162710	18596
**JDP2**	0.015568	3.3832	2.155330216162710	18595
**RAPH1**	0.015568	3.9089	2.155330216162710	18594
**CASP9**	0.015567	5.3288	2.155355793082440	18597
**BEGAIN**	0.015548	3.4599	2.155842022666230	18598
**TARBP1**	0.015356	4.2511	2.160784332971240	18599
**RNF148**	0.01533	6.9078	2.161457677485730	18600
**PPP1CB**	0.015241	5.1707	2.163770036584090	18601
**ANO9**	0.015165	5.3812	2.1657538346361200	18602
**CD37**	0.015165	4.4526	2.1657538346361200	18603
**CRP**	0.015102	3.5987	2.1674047842068800	18604
**TFCP2**	0.014962	2.9153	2.1710948565875900	18605
**RAB9B**	0.014823	1.0682	2.1747880535322200	18606
**ZNF599**	0.014802	6.1614	2.1753486056089400	18607
**AIF1L**	0.014749	3.4029	2.1767663796555700	18608
**CBFB**	0.014749	1.7875	2.1767663796555700	18609
**GPR158**	0.014607	6.388	2.180586660087230	18610
**ODF3L1**	0.014594	4.1624	2.1809379985882000	18611
**KRT26**	0.014522	3.6932	2.1828887641437100	18612
**KRT82**	0.014465	5.789	2.1844390328348500	18613
**LRWD1**	0.014374	2.0198	2.1869249577518000	18614
**INTS5**	0.014218	5.4367	2.191218249312120	18615
**USP6**	0.014143	7.3934	2.1932967926137400	18616
**TAS2R60**	0.014097	4.2292	2.1945763360525200	18617
**LSM14B**	0.013977	5.8981	2.197931292279990	18618
**SCN2B**	0.013977	5.6174	2.197931292279990	18619
**ZNF839**	0.01396	6.6334	2.198408583816040	18620
**PCDHA5**	0.013901	3.5841	2.200068962933750	18621

**RASA4B**	0.013829	3.7887	2.202103440488290	18622
**CRISP1**	0.013807	1.7863	2.2027269084235900	18623
**THUMPD2**	0.013767	6.6732	2.2038626849647000	18624
**ZNF174**	0.013685	7.4057	2.2061999542956000	18625
**KLHL4**	0.013663	3.9884	2.2068290819567400	18626
**FAM120A**	0.013457	4.7016	2.2127628112565600	18627
**PTGER4**	0.013447	2.1372	2.213052847025460	18629
**TAAR8**	0.013447	4.9987	2.213052847025460	18628
**ANK2**	0.01344	5.6194	2.213255982886520	18630
**OR4D1**	0.013375	7.0246	2.2151466197898000	18631
**ELMO1**	0.013244	4.1276	2.2189812173950500	18632
**C11orf44**	0.013195	7.4219	2.220423955995040	18633
**RMDN2**	0.013156	1.6506	2.221575570380950	18634
**TRPC6**	0.013153	5.7333	2.2216642782392400	18635
**PHKB**	0.013069	4.3892	2.224155225418530	18636
**RPL7**	0.012918	0.44945	2.2286680091277000	18638
**TBC1D3E**	0.012918	3.1771	2.2286680091277000	18637
**PLCB2**	0.012787	6.4248	2.2326201785240200	18639
**ZNF404**	0.012755	6.4946	2.2335909145627500	18640
**LOXL3**	0.012635	4.7457	2.2372500439359900	18641
**PABPC4L**	0.012635	4.7425	2.2372500439359900	18642
**TMEM27**	0.012635	4.5884	2.2372500439359900	18643
**GABRA3**	0.012615	6.0673	2.2378628204268600	18644
**ENPEP**	0.012613	2.4185	2.237924144314360	18646
**FEM1C**	0.012613	5.6733	2.237924144314360	18645
**MRPL35**	0.012613	2.2368	2.237924144314360	18647
**LOC100287(**	0.012194	1.0671	2.2509610863944000	18649
**TEX48**	0.012194	4.4515	2.2509610863944000	18648
**TRABD2B**	0.012166	6.1175	2.2518460548911300	18650
**ERCC6L2**	0.011793	5.9536	2.2638067685765900	18651
**OR9A2**	0.011793	5.6681	2.2638067685765900	18652
**ADGRG5**	0.011535	0.39102	2.2722734065846700	18653

**CCSAP**	0.011532	3.0264	2.2723728190095100	18655
**TUBA1A**	0.011532	4.9277	2.2723728190095100	18654
**ZNF622**	0.011514	6.2751	2.2729697656621600	18656
**FAM114A2**	0.011365	4.3359	2.2779425291275600	18657
**ZRANB2**	0.01132	2.1731	2.2794555073968500	18658
**BRINP1**	0.011309	5.2969	2.279826141394410	18662
**HSF2BP**	0.011309	6.3052	2.279826141394410	18659
**ITGAL**	0.011309	5.9725	2.279826141394410	18660
**MRPL45**	0.011309	5.8973	2.279826141394410	18661
**MBD2**	0.011195	5.4385	2.28368582183119	18663
**TNK2**	0.011195	2.7414	2.28368582183119	18664
**VNN 1.00**	0.011095	4.8718	2.2870997432099700	18666
**SPRED2**	0.011095	0.17101	2.2870997432099700	18665
**PELI3**	0.011029	3.1407	2.2893676196381100	18667
**TCF7L2**	0.010986	5.6573	2.2908515348957400	18668
**SC5D**	0.010871	4.8607	2.2948451202042000	18669
**FNDC8**	0.010858	5.6964	2.295298878975750	18671
**SLC25A28**	0.010858	5.9837	2.295298878975750	18670
**C2**	0.010823	6.7449	2.2965228916841800	18672
**FNDC7**	0.010727	0.75502	2.2998979584732800	18674
**PCDHB14**	0.010727	3.7363	2.2998979584732800	18673
**C8orf44-SGI**	0.01065	6.9936	2.3026241037742000	18676
**CCDC73**	0.01065	7.0538	2.3026241037742000	18675
**TMEM182**	0.010624	6.4953	2.303548497687210	18677
**ZFHX3**	0.010561	3.2658	2.3057965741451000	18678
**BSPH1**	0.010479	2.7235	2.3087402078612300	18679
**RTL9**	0.010477	1.6224	2.3088122542123100	18680
**TBX22**	0.010403	5.7661	2.31148643255388	18681
**CASKIN1**	0.010174	3.3189	2.3198683229085200	18682
**PTPRN**	0.010174	2.1288	2.3198683229085200	18683
**AADAC**	0.01013	6.5336	2.321497633977090	18684
**HLA-F**	0.010073	5.9745	2.323617537736140	18685

**SNAP29**	0.010073	3.5295	2.323617537736140	18686
**C1orf229**	0.009854	2.8395	2.3318610600463100	18688
**GCM1**	0.009854	4.4672	2.3318610600463100	18687
**NBPF15**	0.0096904	5.7434	2.338124289372780	18689
**SPACA7**	0.0096306	2.2467	2.340436732729330	18690
**MLYCD**	0.00963	5.5498	2.3404599980136700	18691
**SMYD1**	0.00963	4.8822	2.3404599980136700	18692
**KLHL1**	0.0093919	6.4139	2.349794040165870	18693
**SSX2IP**	0.0092602	5.3812	2.3550462295725100	18694
**TMEM26**	0.0092392	4.8867	2.3558897428775000	18695
**TNKS**	0.009235	1.9808	2.356058646855990	18696
**MIOS**	0.0090798	3.8176	2.362347698356610	18697
**MAGEB10**	0.009054	6.0233	2.3634022827680300	18699
**SH3GL1**	0.009054	6.2645	2.3634022827680300	18698
**C19orf25**	0.0089638	3.8229	2.3671100450352600	18700
**SCARF1**	0.0089539	5.6234	2.367518982556280	18701
**ATP1A4**	0.0088857	0.71696	2.370346921250820	18702
**ASB15**	0.00886	5.6545	2.3714175171367400	18703
**GUCA1B**	0.00886	5.4561	2.3714175171367400	18705
**RRP8**	0.00886	5.4972	2.3714175171367400	18704
**ALG5**	0.0088185	3.6268	2.3731520597969100	18706
**RAB8A**	0.0087403	3.8893	2.376440058540840	18707
**KRTAP13-2**	0.0086727	5.6282	2.379303220334090	18709
**SOCS4**	0.0086727	6.5559	2.379303220334090	18708
**CDC34**	0.0085735	2.218	2.3835404210387100	18711
**VCX**	0.0085735	2.4296	2.3835404210387100	18710
**CDK5RAP3**	0.0084996	3.7273	2.3867250101015900	18713
**CITED2**	0.0084996	4.7087	2.3867250101015900	18712
**POLR2I**	0.0083784	6.5367	2.392000901525080	18715
**ZBTB22**	0.0083784	6.9927	2.392000901525080	18714
**C17orf74**	0.008307	5.8303	2.395140420380300	18717
**GRIPAP1**	0.008307	6.3488	2.395140420380300	18716

**FBXW5**	0.0081985	4.2713	2.3999569264315000	18718
**NFATC4**	0.0081985	2.2255	2.3999569264315000	18719
**B4GALT4**	0.0081539	5.2106	2.4019530535759700	18720
**CPXCR1**	0.0081539	2.6768	2.4019530535759700	18722
**TAS2R13**	0.0081539	4.2458	2.4019530535759700	18721
**ATG4C**	0.008147	5.9505	2.402262727908900	18723
**LCMT2**	0.0080096	6.6751	2.408477786108010	18724
**NEK9**	0.0079881	4.4656	2.409458769449560	18725
**CRH**	0.007634	1.7132	2.425958857612770	18727
**LBX2**	0.007634	5.1473	2.425958857612770	18726
**C6orf223**	0.0076204	6.221	2.4266059127217100	18728
**HOOK1**	0.0075606	3.5176	2.4294631730558000	18729
**CTRC**	0.0075391	2.7363	2.4304953144359400	18730
**REPIN1**	0.0075359	5.5438	2.430649157059430	18731
**METTL21C**	0.0074137	4.6755	2.436567520767540	18732
**DEFB126**	0.0072647	6.1383	2.4439012970195700	18733
**LCE3C**	0.007261	5.2786	2.444085092355260	18734
**DCAF4**	0.0072579	4.8077	2.44423914663279	18735
**NUBPL**	0.0072579	4.7631	2.44423914663279	18736
**CCDC9**	0.0072267	7.2561	2.4457928674856900	18737
**CCDC14**	0.0070743	5.7613	2.453468300726440	18738
**KCNJ1**	0.0070475	5.6388	2.454833107292810	18739
**ANKRD17**	0.0070213	0.54471	2.456171793310600	18740
**YWHAE**	0.0068807	6.1186	2.4634319681055600	18741
**MYO1A**	0.0067915	6.1431	2.4681062165752900	18743
**PLAA**	0.0067915	6.5716	2.4681062165752900	18742
**TXNDC17**	0.0065979	1.7541	2.478440798035090	18744
**SLC25A31**	0.0065764	2.5456	2.4796049736956200	18745
**TEN1**	0.0065764	1.6001	2.4796049736956200	18746
**APOBEC3H**	0.0065497	3.0261	2.481055411457950	18747
**POPDC2**	0.0065497	2.51	2.481055411457950	18748
**DNM1**	0.0064086	4.5253	2.48880843263207	18749

**UBFD1**	0.0063456	5.2455	2.4923189702710100	18750
**HES1**	0.006332	0.71263	2.4930808469710700	18751
**NRK**	0.0063299	3.9623	2.4931986187710100	18752
**RNASE3**	0.00615	6.0639	2.5034184954898600	18753
**SPATS1**	0.006129	3.3324	2.5046286796509300	18754
**RWDD2B**	0.0060797	7.6903	2.5074842315489400	18755
**MCHR2**	0.0059674	6.9708	2.5140662656316100	18756
**HDHD3**	0.0059417	3.7257	2.515588001345190	18757
**ZNF597**	0.0059417	2.0321	2.515588001345190	18758
**SLC44A2**	0.0057308	6.7607	2.5283011085612400	18759
**MYSM1**	0.0057214	3.0495	2.5288773518834800	18760
**CACNB2**	0.0056207	6.2943	2.5351038157976600	18761
**CSTF2T**	0.0055734	1.2453	2.538062720048720	18762
**PLEKHF1**	0.005501	5.8855	2.5426352718144500	18763
**SH3BGRL3**	0.0054103	4.6012	2.5484396559488800	18764
**TPPP2**	0.0053751	6.1091	2.550715614526530	18765
**INSL5**	0.0053647	4.04	2.5513905926704900	18766
**ALOX15B**	0.005287	7.0392	2.556470603689450	18767
**PCDHB15**	0.0052563	0.18435	2.5584960814261200	18768
**SDR9C7**	0.0052288	5.4491	2.560319389683450	18769
**SIGLECL1**	0.0051223	6.5115	2.5674620428540400	18770
**AKNA**	0.0050714	5.3322	2.570922559954800	18771
**EFCAB5**	0.0049738	6.7731	2.577645458110500	18772
**C10orf88**	0.0049376	4.7626	2.5801688956901600	18773
**SPATA17**	0.0047929	7.1698	2.5904232141945900	18774
**MAP4K4**	0.0047509	7.0939	2.593451268047610	18775
**HNF4G**	0.0047231	5.2566	2.5954687087312800	18776
**JUN**	0.0047231	5.1829	2.5954687087312800	18777
**ABCA7**	0.0047026	6.4044	2.596963185756020	18779
**HAND2**	0.0047026	6.3599	2.596963185756020	18780
**HNRNPLL**	0.0047026	6.9096	2.596963185756020	18778
**KNTC1**	0.0047026	6.276	2.596963185756020	18781

**OPN1SW**	0.0045479	2.3758	2.608432294544060	18782
**SSC4D**	0.0044141	7.0609	2.61863634789595	18783
**B3GALT4**	0.0043417	6.5204	2.624273589971970	18785
**FGFRL1**	0.0043417	6.0332	2.624273589971970	18786
**HIST1H2BD**	0.0043417	6.627	2.624273589971970	18784
**RASA3**	0.0042882	3.2394	2.6284934664058500	18787
**ADAMTS1**	0.0042772	4.7893	2.6293669337221900	18788
**FAM104A**	0.0041613	5.4245	2.638694257784040	18789
**OR52B4**	0.0041314	6.1099	2.6411382166545400	18790
**RGPD5**	0.0041119	5.089	2.6427406433246600	18791
**ITPR3**	0.0040228	6.6882	2.6501501627564000	18792
**TARSL2**	0.0040018	4.8003	2.651917891815920	18793
**CT55**	0.0038565	6.6343	2.66438164579624	18794
**MS4A5**	0.0038565	6.2687	2.66438164579624	18795
**OR7D4**	0.0038061	5.3759	2.6688033594592700	18796
**FAM20C**	0.0038045	4.3948	2.668944588982440	18797
**ACYP2**	0.0037563	4.9352	2.673224292459700	18798
**SLC45A2**	0.0037227	7.1148	2.6762368787242900	18799
**CCDC68**	0.0036823	6.2566	2.679891614301220	18800
**ADAMTS10**	0.0036487	2.9388	2.6829586987393500	18801
**ZNF736**	0.0035795	5.3318	2.6893561163582700	18802
**KRTAP2-1**	0.0035186	6.2181	2.695078728170600	18804
**ROBO2**	0.0035186	6.6645	2.695078728170600	18803
**HSPA2**	0.0034977	3.6132	2.6970631562083200	18805
**C14orf37**	0.0034924	7.1419	2.697568076782220	18806
**KLHDC7B**	0.0034232	5.519	2.70422457168188	18807
**SUMO3**	0.0034232	5.0033	2.70422457168188	18808
**OR8B8**	0.003377	6.4682	2.708736280228370	18809
**PATE1**	0.0033691	7.0851	2.7095133108756900	18810
**FOSB**	0.0033471	0.26804	2.7116858565269100	18811
**PDZK1**	0.0033366	1.8389	2.7127272821245600	18812
**MTHFD1**	0.0032842	6.2938	2.717968953062900	18813

**CSTL1**	0.0031834	0.86411	2.7282672594052700	18815
**DLX5**	0.0031834	4.1686	2.7282672594052700	18814
**FAM71E1**	0.0030276	3.0655	2.7447771504934200	18816
**ALDOC**	0.002961	4.6727	2.7520692273492300	18817
**C10orf90**	0.0029348	6.2008	2.7549784536959500	18819
**ERLEC1**	0.0029348	6.7232	2.7549784536959500	18818
**NFATC2IP**	0.0028587	6.2568	2.7635633049961200	18820
**HBB**	0.0028566	5.3033	2.7638031153393800	18821
**ZNF484**	0.0027533	6.4527	2.775800414991490	18822
**RAB27A**	0.0026714	4.3172	2.785604592338070	18823
**GZMA**	0.0026074	5.0341	2.7934567603773800	18824
**PAX2**	0.0026074	4.4108	2.7934567603773800	18825
**HMG20A**	0.0025413	5.2291	2.8017515452673900	18826
**WNK2**	0.0025387	2.9456	2.8020817848858	18827
**ABHD17B**	0.0024501	6.9929	2.8135222159855800	18828
**CNTRL**	0.002309	6.3942	2.832538533151260	18829
**RAB10**	0.0021799	6.7439	2.8508830648989500	18830
**KRT8**	0.0020624	2.4735	2.868456437253050	18831
**PTPA**	0.0020398	6.2632	2.8719403252830500	18832
**FUT4**	0.0020204	4.2795	2.874958976330010	18833
**ZNRF4**	0.0019512	2.9726	2.8859452835605500	18834
**SETD9**	0.0019155	3.2217	2.8917521853274500	18835
**PRAP1**	0.0018704	6.7968	2.8992303180930400	18836
**IL1RAPL1**	0.0018279	6.5906	2.9064288793023000	18837
**SLC6A16**	0.0017492	3.0262	2.92017044172401	18838
**PLVAP**	0.0017109	6.5961	2.9270626051745100	18839
**MUC7**	0.0016994	3.835	2.9291594758273000	18840
**RAPGEF5**	0.0016669	7.0575	2.935156039297870	18841
**GAN**	0.0016286	7.0459	2.9423609833056300	18842
**PINK1**	0.0015981	5.8599	2.9482098748109900	18843
**KCNU1**	0.0014948	7.1231	2.9688052626675500	18844
**CDAN1**	0.001436	0.29716	2.9811159887517200	18845

**COX6A1**	0.0013558	6.7783	2.998670944970330	18846
**NUDT4**	0.0013091	7.452	3.0093352991065400	18847
**CLDN18**	0.001274	0.94108	3.017582123481480	18848
**CCDC96**	0.0012614	5.2364	3.0205932741328000	18849
**C1QL2**	0.0012336	4.5882	3.02733545947365	18850
**MYCN**	0.0011764	6.8675	3.0416567945750900	18851
**HECTD3**	0.0011071	5.136	3.059887709865830	18852
**HIST1H2BM**	0.0010898	0.91179	3.0646017883525900	18853
**ABO**	0.0010704	6.9061	3.069970393940090	18854
**PTTG1**	0.00099802	4.7013	3.0908208991889000	18855
**C12orf74**	0.00092091	6.9531	3.114620047906410	18856
**ARID3A**	0.0007672	2.7216	3.1680976148991200	18857
**PEF1**	0.00075986	7.3219	3.1708913144387500	18858
**OR6C75**	0.00073993	4.1069	3.178604247989730	18859
**PRDX3**	0.00070793	5.7716	3.1913982079573200	18860
**LTBP2**	0.00062504	6.8222	3.227200132602160	18862
**UCKL1**	0.00062504	7.0672	3.227200132602160	18861
**SRPK3**	0.00059304	2.9872	3.242206630746440	18863
**BASP1**	0.00055842	4.8319	3.259308900728310	18864
**PRAMEF13**	0.00055265	6.8807	3.262254169057560	18865
**ZC3H12D**	0.00050753	5.1716	3.2863189779037400	18866
**ZNF564**	0.00043881	2.369	3.327077398583830	18867
**BRCA1**	0.00042413	4.0782	3.3365472976425400	18868
**ETNPPL**	0.00025783	2.9429	3.4724870144989400	18869
**BAZ2B**	0.00022111	2.8405	3.513514688594850	18870
**CEACAM5**	0.0001419	7.4955	3.6296552736782400	18871
**CAPRIN1**	9.2589E-05	0.21723	3.7384232801889700	18873
**ZNF511-PR/**	9.2589E-05	4.8208	3.7384232801889700	18872
**NBAS**	6.2163E-05	7.419	3.8374352740295500	18874
